# Supplementary material for: Cobalt-catalyzed aminoalkylative carbonylation of alkenes toward direct synthesis of γ-amino acid derivatives and peptides
Source: Nat Commun. 2023 Nov 17;14:7439. doi: 10.1038/s41467-023-43306-y (PMC10656502; doi:10.1038/s41467-023-43306-y)
Supplement: Supplementary file 1 — Supplementary Information [file 41467_2023_43306_MOESM1_ESM.pdf]

Supplementary Information for

Cobalt-Catalyzed Aminoalkylative Carbonylation of Alkenes Toward Direct Synthesis of γ-Amino Acid Derivatives and Peptides

Le-Cheng Wang[1,2], Yang Yuan[1], Youcan Zhang[1], Xiao-Feng Wu[1,2,3*]

[1]Dalian National Laboratory for Clean Energy, Dalian Institute of Chemical Physics, Chinese Academy of Sciences, 116023 Dalian, Liaoning, China.

[2]Leibniz-Institut für Katalyse e.V., 18059 Rostock, Germany.

[3]University of Chinese Academy of Sciences, Beijing 100049, China.

*Corresponding author e-mail: xwu2020@dicp.ac.cn

Table of Contents

1. Supplementary Note1 1
2. Supplementary Note 2 2
    2.1 Optimization for carbonylationn of unactivated alkenes 2
    2.2 Optimization for carbonylationn of ethylene 4
3. Supplementary Methods 5
    3.1 General Carbonylation 5
        3.1.1 General Procedure I 5
        3.1.2 General Procedure II 5
        3.1.3 General Procedure III 6
        3.1.4 General Procedure IV 6
        3.1.5 General Procedure V 7
        3.1.6 General Procedure VI 8
        3.1.7 General Procedure VII 8
    3.2 Typical Procedures for the Synthesis of **10** 9
    3.3 Typical Procedures for the Synthesis of **11** and **12** 10
    3.4 Typical Procedures for the Synthesis of **13** 10
    3.5 Typical Procedures for the Radical Trapping Experiments 11
    3.6 Typical Procedures for the Radical Clock Experiments 11
    3.7 Typical Procedures for the Competitive Experiments 12
    3.8 Typical Procedures for the Background Experiments 13
4. Supplementary Note 3 15
    4.1 Radical-Trapping Products by GC-MS 15
    4.2 Spectroscopic Data of Products 16
    4.3 The NMR Spectrum 80
5. Supplementary References 192

1. Supplementary Note 1

Reagents, solvents, and analytical methods:

Unless otherwise noted, all reactions were carried out under carbon monoxide or nitrogen atmosphere. All reagents were from commercial sources and used as received without further purification. Column chromatography was performed on silica gel (200-300 meshes) using petroleum ether (bp. 60~90 °C), dichloromethane and ethyl acetate as eluent. All NMR spectra were recorded at ambient temperature using Bruker Avance III 400 MHz NMR (^{1}H, 400 MHz; ^{13}C {1H}, 101 MHz, ^{19}F 376 MHz), Bruker AVANCE III HD 700 MHz NMR spectrometers (^{1}H, 700 MHz; ^{13}C{^{1}H}, 176 MHz). 1H NMR chemical shifts are reported relative to TMS and were referenced via residual proton resonances of the corresponding deuterated solvent ($CDCl_3$: 7.26 ppm; d_6-DMSO: 2.50 ppm) whereas 13C{1H} NMR spectra are reported relative to TMS via the carbon signals of the deuterated solvent ($CDCl_3$: 77.0 ppm; d_6-DMSO: 39.5 ppm. Data for ^{1}H are reported as follows: chemical shift (δ ppm), multiplicity (s = singlet, d = doublet, t = triplet, q = quartet, quint = quintet, m = multiplet, br = broad), coupling constant (Hz), and integration. All ^{13}C NMR spectra were broad-band ^{1}H decoupled. All reactions were monitored by GC-FID or NMR analysis. HRMS data was obtained with Micromass HPLC-Q-TOF mass spectrometer (ESI-TOF) or Agilent 6540 Accurate-MS spectrometer (Q-TOF).

NOTE: Because of the high toxicity of carbon monoxide, all the reactions should be performed in an autoclave. The laboratory should be well-equipped with a CO detector and alarm system.

2. Supplementary Note 2

2.1 Optimization for carbonylation of unactivated alkenes

Supplementary Table 1. Optimization of catalysts

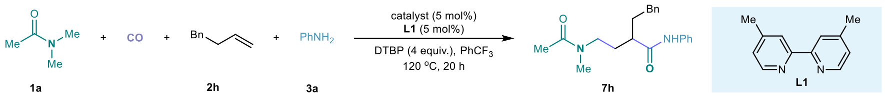

entry	catalyst	**7h** [%]
1	$Co(acac)_2$	43
2	$Co(acac)_3$	19
3	$Co(hfacac)_2$	25
4	$CoCl(PPh_3)_3$	trace
5	$Ni(acac)_2$	8
6	$Ni(TMHD)_2$	trace
7	CuBr·DME	trace
8	$Fe_3(CO)_{12}$	trace

[a] Reaction condition: **1a** (2 mmol), **2h** (0.4 mmol), **3a** (0.2 mmol), catalyst (5 mol%), **L1** (5 mol%), DTBP (0.8 mmol), $PhCF_3$ (0.2 M), CO (50 bar), 120 °C, 20 h. determined by gas chromatography (GC) using hexadecane as the internal standard. [b] isolated yield.

Supplementary Table 2. Optimization of ligands

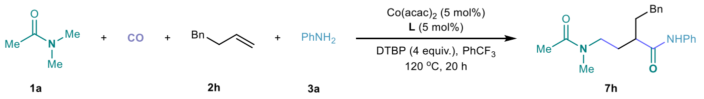

entry	ligand	**7h** [%]
1	**L2**	45
2	**L3**	50
3	**L4**	39
4	**L5**	75 (72[b])
5	**L6**	45
6	**L7**	54
7	**L8**	29
8	**L9**	43
9	**L10**	25
10	**L 11**	47
11	**L12**	65
12	**L13**	83
13	**L14**	54
14	**L15**	76

[a] Reaction condition: **1a** (2 mmol), **2h** (0.4 mmol), **3a** (0.2 mmol), $Co(acac)_2$ (5 mol%), **L** (5 mol%), DTBP (0.8 mmol), $PhCF_3$ (0.2 M), CO (50 bar), 120 °C, 20 h. determined by gas chromatography (GC) using hexadecane as the internal standard. [b] isolated yield.

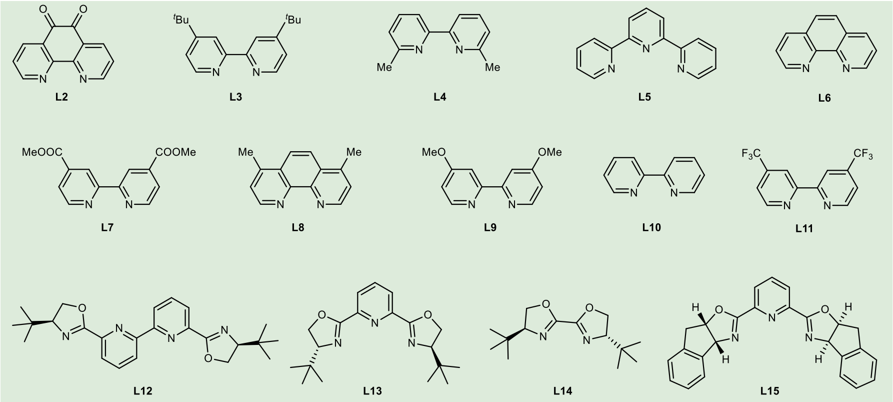

Supplementary Figure 1. Optimization of ligands

Supplementary Table 3. Optimization of other conditions

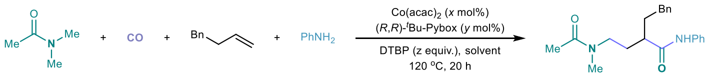

entry	initiator (mmol)	CO (bar)	$Co(acac)_2$/**L13** (mol%)	solvent (M)	**7h** [%]
1	TBHP (0.8)	50	5 / 5	$PhCF_3$ (0.2)	trace
2	BPO (0.8)	50	5 / 5	$PhCF_3$ (0.2)	trace
3	$K_2S_2O_8$	50	5 / 5	$PhCF_3$ (0.2)	trace
4	*m*-CPBA	50	5 / 5	$PhCF_3$ (0.2)	trace
5	H_2O_2 (0.8)	50	5 / 5	$PhCF_3$ (0.2)	50
6	DTBP (0.6)	50	5 / 5	$PhCF_3$ (0.2)	80
7	DTBP (0.4)	50	5 / 5	$PhCF_3$ (0.2)	74
8	DTBP (0.2)	50	5 / 5	$PhCF_3$ (0.2)	19
9	DTBP (0.6)	40	5 / 5	$PhCF_3$ (0.2)	79
10	DTBP (0.6)	20	5 / 5	$PhCF_3$ (0.2)	50
11	DTBP (0.6)	10	5 / 5	$PhCF_3$ (0.2)	38
12	DTBP (0.6)	40	3 / 3	$PhCF_3$ (0.2)	63
13	DTBP (0.6)	40	1 / 1	$PhCF_3$ (0.2)	37
14	DTBP (0.6)	40	3 / 5	$PhCF_3$ (0.2)	71
15	DTBP (0.6)	40	5 / 5	PhH (0.2)	56
16	DTBP (0.6)	40	5 / 5	PhCl (0.2)	72
17	DTBP (0.6)	40	5 / 5	DCE (0.2)	32
18	DTBP (0.6)	40	5 / 5	DMAc (0.2)	82

[a] Reaction condition: **1a** (2 mmol), **2h** (0.4 mmol), **3a** (0.2 mmol), $Co(acac)_2$ (*x* mol%), **L13** (*y* mol%), initiator (*z* mmol), $PhCF_3$ (0.2 M), CO (*n* bar), 120 °C, 20 h. determined by gas chromatography (GC) using hexadecane as the internal standard. [b] isolated yield.

2.2 Optimization for carbonylation of ethylene

Supplementary Table 4. Optimization of conditions for ethylene

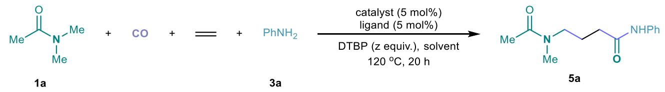

entry	ethylene (bar)	CO (bar)	catalyst (mol%)	ligand (mol%)	**5a** [%]
1	10	40	$Co(acac)_2$ (5)	**L1** (5)	77
2	10	40	$Co(acac)_3$ (5)	**L1** (5)	75
3	10	40	$CoCl(PPh_3)_3$ (5)	**L1** (5)	trace
4	10	40	$Ni(acac)_2$ (5)	**L1** (5)	59
5	10	40	$Co(acac)_2$ (5)	**L2** (5)	48
6	10	40	$Co(acac)_2$ (5)	**L3** (5)	78
7	10	40	$Co(acac)_2$ (5)	**L4** (5)	71
8	10	40	$Co(acac)_2$ (5)	**L5** (5)	76
9	10	40	$Co(acac)_2$ (5)	**L6** (5)	83
10	10	40	$Co(acac)_2$ (5)	**L7** (5)	36
11	10	40	$Co(acac)_2$ (5)	**L8** (5)	66
12	10	40	$Co(acac)_2$ (5)	**L9** (5)	27
13	10	40	$Co(acac)_2$ (5)	**L10** (5)	52
14	10	40	$Co(acac)_2$ (5)	**L11** (5)	23
15	10	40	$Co(acac)_2$ (5)	**L12** (5)	53
16	10	40	$Co(acac)_2$ (5)	**L13** (5)	66
17	10	40	$Co(acac)_2$ (5)	**L14** (5)	71
18	10	40	$Co(acac)_2$ (5)	**L15** (5)	59
19	10	20	$Co(acac)_2$ (5)	**L6** (5)	81 (78%[b])
20	10	10	$Co(acac)_2$ (5)	**L6** (5)	75
21	5	5	$Co(acac)_2$ (5)	**L6** (5)	72

[a] Reaction condition: **1a** (2 mmol), **3a** (0.2 mmol), $Co(acac)_2$ (5 mol%), **L13** (5 mol%), DTBP (0.6 mmol), $PhCF_3$ (0.2 M), CO (*x* bar), ethylene (*y* bar), 120 °C, 20 h. determined by gas chromatography (GC) using hexadecane as the internal standard. [b] isolated yield.

3. Supplementary methods

3.1 General carbonylation

3.1.1 General Procedure I

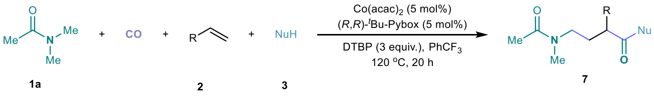

A 4 mL screw-cap vial was charged with Co(acac)$_2$ (3.9 mg, 5 mol%), (*R*,*R*)-^{t}Bu-Pybox (4.9 mg, 5 mol%), and an oven-dried stirring bar. The vial was closed with a Teflon septum and cap and connected to the atmosphere via a needle. After **1a** (258 mg, 10 equiv.), PhCF$_3$ (1.5 mL, 0.2 M), NuH (1 equiv., 0.3 mmol), and DTBP (131.6 mg, 3 equiv.) were added with a syringe under nitrogen atmosphere, the vial was moved to an alloy plate and put into a Parr 4560 series autoclave (300 mL) under an argon atmosphere. At room temperature, the autoclave was flushed with CO three times, then charged with 40 bar of CO. The autoclave was placed on a heating plate equipped with a magnetic stirrer and an aluminum block. The reaction mixture was heated to 120 ^{o}C for 20 h. After the reaction was complete, the autoclave was cooled down with ice water to room temperature and the pressure was released carefully. After cooling to room temperature, the reaction mixture was directly purified by column chromatographyon silica gel using petroleum ether, ethyl acetate, and methanol to afford the corresponding product.

3.1.2 General Procedure I

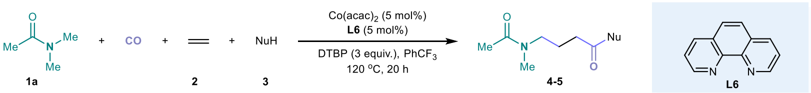

A 4 mL screw-cap vial was charged with Co(acac)$_2$ (3.9 mg, 5 mol%), **L6** (2.7 mg, 5 mol%), and an oven-dried stirring bar. The vial was closed with a Teflon septum and cap and connected to the atmosphere via a needle. After **1a** (258 mg, 10 equiv.), PhCF$_3$ (1.5 mL, 0.2 M), NuH (1 equiv., 0.3 mmol), and DTBP (131.6 mg, 3 equiv.) were added with a syringe under nitrogen atmosphere, the vial was moved to an alloy plate and put

into a Parr 4560 series autoclave (300 mL) under an argon atmosphere. At room temperature, the autoclave was flushed with CO two times and ethylene two times, then charged with 40 bar of CO and 10 bar ethylene. The autoclave was placed on a heating plate equipped with a magnetic stirrer and an aluminum block. The reaction mixture was heated to 120 °C for 20 h. After the reaction was complete, the autoclave was cooled down with ice water to room temperature and the pressure was released carefully. After cooling to room temperature, the reaction mixture was directly purified by column chromatographyon silica gel using petroleum ether, ethyl acetate, and methanol to afford the corresponding product.

3.1.3 General carbonylation III

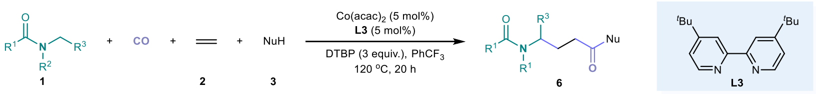

A 4 mL screw-cap vial was charged with $Co(acac)_2$ (3.9 mg, 5 mol%), **L3** (2.8 mg, 5 mol%), and an oven-dried stirring bar. The vial was closed with a Teflon septum and cap and connected to the atmosphere via a needle. After **1** (10 equiv.), $PhCF_3$ (1.5 mL, 0.2 M), NuH (1 equiv., 0.3 mmol), and DTBP (131.6 mg, 3 equiv.) were added with a syringe under nitrogen atmosphere, the vial was moved to an alloy plate and put into a Parr 4560 series autoclave (300 mL) under an argon atmosphere. At room temperature, the autoclave was flushed with CO two times and ethylene two times, then charged with 40 bar of CO and 10 bar ethylene. The autoclave was placed on a heating plate equipped with a magnetic stirrer and an aluminum block. The reaction mixture was heated to 120 °C for 20 h. After the reaction was complete, the autoclave was cooled down with ice water to room temperature and the pressure was released carefully. After cooling to room temperature, the reaction mixture was directly purified by column chromatographyon silica gel using petroleum ether, ethyl acetate, and methanol to afford the corresponding product.

3.1.4 General carbonylation IV

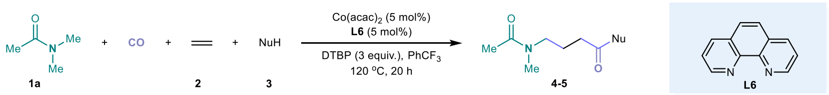

A 4 mL screw-cap vial was charged with Co(acac)$_2$ (3.9 mg, 5 mol%), **L6** (2.7 mg, 5 mol%), and an oven-dried stirring bar. The vial was closed with a Teflon septum and cap and connected to the atmosphere via a needle. After **1a** (258 mg, 10 equiv.), PhCF$_3$ (1.5 mL, 0.2 M), NuH (1 equiv., 0.3 mmol), and DTBP (131.6 mg, 3 equiv.) were added with a syringe under nitrogen atmosphere, the vial was moved to an alloy plate and put into a Parr 4560 series autoclave (300 mL) under an argon atmosphere. At room temperature, the autoclave was flushed with CO two times and ethylene two times, then charged with 5 bar of CO and 5 bar ethylene. The autoclave was placed on a heating plate equipped with a magnetic stirrer and an aluminum block. The reaction mixture was heated to 120 ^{o}C for 20 h. After the reaction was complete, the autoclave was cooled down with ice water to room temperature and the pressure was released carefully. After cooling to room temperature, the reaction mixture was directly purified by column chromatographyon silica gel using petroleum ether, ethyl acetate, and methanol to afford the corresponding product.

3.1.5 General carbonylation V

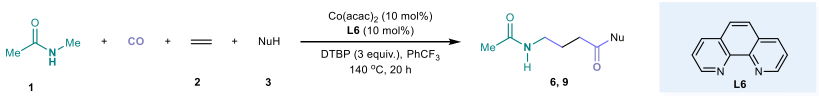

A 4 mL screw-cap vial was charged with Co(acac)$_2$ (3.9 mg, 5 mol%), **L6** (2.7 mg, 5 mol%), and an oven-dried stirring bar. The vial was closed with a Teflon septum and cap and connected to the atmosphere via a needle. After **1a** (258 mg, 10 equiv.), PhCF$_3$ (1.5 mL, 0.2 M), NuH (1 equiv., 0.3 mmol), and DTBP (131.6 mg, 3 equiv.) were added with a syringe under nitrogen atmosphere, the vial was moved to an alloy plate and put into a Parr 4560 series autoclave (300 mL) under an argon atmosphere. At room temperature, the autoclave was flushed with CO two times and ethylene two times, then charged with 40 bar of CO and 10 bar ethylene. The autoclave was placed on a heating plate equipped with a magnetic stirrer and an aluminum block. The reaction mixture

was heated to 140 °C for 20 h. After the reaction was complete, the autoclave was cooled down with ice water to room temperature and the pressure was released carefully. After cooling to room temperature, the reaction mixture was directly purified by column chromatographyon silica gel using petroleum ether, ethyl acetate, and methanol to afford the corresponding product. In order to get more pure products, the column chromatographyon silica gel products need to be required to be crystallized. At this time, the product production rate will decrease by about 10%-20% yields.

3.1.6 General scale-up carbonylation VI

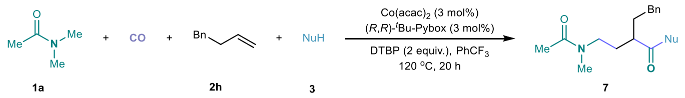

A 25 mL screw-cap vial was charged with $Co(acac)_2$ (25.7 mg, 5 mol%), (*R*,*R*)-^{t}Bu-Pybox (32.8 mg, 5 mol%), and an oven-dried stirring bar. The vial was closed with a Teflon septum and cap and connected to the atmosphere via a needle. After **1a** (860 mg, 5 equiv.), $PhCF_3$ (10 mL), amines **3a** (186 mg, 2 mmol), and DTBP (584 mg, 2 equiv.) were added with a syringe under nitrogen atmosphere, the vial was moved to an alloy plate and put into a Parr 4560 series autoclave (300 mL) under an argon atmosphere. At room temperature, the autoclave was flushed with CO two times and ethylene two times, then charged with 40 bar of CO. The autoclave was placed on a heating plate equipped with a magnetic stirrer and an aluminum block. The reaction mixture was heated to 120 °C for 20 h. After the reaction was complete, the autoclave was cooled down with ice water to room temperature and the pressure was released carefully. After cooling to room temperature, the reaction mixture was directly purified by column chromatographyon silica gel using petroleum ether, ethyl acetate, and methanol to afford the corresponding product.

3.1.7 General scale-up carbonylation VII

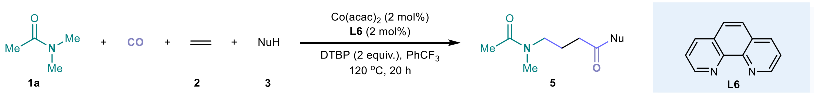

A 25 mL screw-cap vial was charged with Co(acac)$_2$ (35.7 mg, 5 mol%), **L6** (32.8 mg, 5 mol%), and an oven-dried stirring bar. The vial was closed with a Teflon septum and cap and connected to the atmosphere via a needle. After **1a** (860 mg, 5 equiv.), PhCF$_3$ (10 mL), amines **3** (2 mmol), and DTBP (584 mg, 2 equiv.) were added with a syringe under nitrogen atmosphere, the vial was moved to an alloy plate and put into a Parr 4560 series autoclave (300 mL) under an argon atmosphere. At room temperature, the autoclave was flushed with CO two times and ethylene two times, then charged with 5 bar of CO and 5 bar ethylene. The autoclave was placed on a heating plate equipped with a magnetic stirrer and an aluminum block. The reaction mixture was heated to 120 ^{o}C for 20 h. After the reaction was complete, the autoclave was cooled down with ice water to room temperature and the pressure was released carefully. After cooling to room temperature, the reaction mixture was directly purified by column chromatographyon silica gel using petroleum ether, ethyl acetate, and methanol to afford the corresponding product.

3.2 Typical Procedures for the Synthesis of 10

A 25 mL screw-cap vial was charged with Co(acac)$_2$ (3.9 mg, 5 mol%), **L3** (2.8 mg, 5 mol%), and an oven-dried stirring bar. The vial was closed with a Teflon septum and cap and connected to the atmosphere via a needle. After *N*,*N*-dimethylpent-4-enamide (381 mg, 10 equiv.), PhCF$_3$ (1.5 mL, 0.2 M), NuH (1 equiv., 0.3 mmol), and DTBP (131.6 mg, 3 equiv.) were added with a syringe under nitrogen atmosphere, the vial was moved to an alloy plate and put into a Parr 4560 series autoclave (300 mL) under an argon atmosphere. At room temperature, the autoclave was flushed with CO two times, then charged with 40 bar of CO. The autoclave was placed on a heating plate equipped with a magnetic stirrer and an aluminum block. The reaction mixture was heated to 120 ^{o}C for 20 h. After the reaction was complete, the autoclave was cooled down with ice water to room temperature and the pressure was released carefully. After cooling to room temperature, the reaction mixture was directly purified by column chromatographyon silica gel using petroleum ether and ethyl acetate to afford the corresponding product.

3.3 Typical Procedures for the Synthesis of 11 and 12

6j (0.3 mmol) and LiOH-H_2O (5.1 mmol) were stirred in a THF/H_2O (2.5:1) mixture at room temperature for 6 h. The mixture was then acidified to pH 1 with a 1M HCl solution and extracted with EtOAc (3× 10 mL), and the combined organic layer was dried over Na_2SO_4, and recrystallization (MeOH) to afford the product Ac-Glycine (41.3 mg, 95%).

To a stirred solution of **11** (0.2 mmol) in anhydrous 1,4-dioxane (1 mL) was added Schwartz reagent (1.5-2 equiv) at room temperature and the reaction mixture was stirred for 2-5 min. After which, the reaction was quenched with HCl in 1,4-dioxane to make a stable salt.[2]

3.4 Typical Procedures for the Synthesis of 13

A 4 mL screw-cap vial was charged with Co(acac)$_2$ (7.8 mg, 10 mol%), **L6** (5.4 mg, 10 mol%), and an oven-dried stirring bar. The vial was closed with a Teflon septum and cap and connected to the atmosphere via a needle. After *N*-Methylacetamide (10 equiv.), $PhCF_3$ (1.5 mL, 0.2 M), 1,6-hexanediol (1 equiv., 0.3 mmol), and DTBP (263 mg, 3 equiv.) were added with a syringe under nitrogen atmosphere, the vial was moved to an alloy plate and put into a Parr 4560 series autoclave (300 mL) under an argon atmosphere. At room temperature, the autoclave was flushed with CO two times and ethylene two times, then charged with 40 bar of CO and 10 bar ethylene. The autoclave was placed on a heating plate equipped with a magnetic stirrer and an aluminum block. The reaction mixture was heated to 120 ^{o}C for 20 h. After the reaction was complete, the autoclave was cooled down with ice water to room temperature and the pressure was released carefully. After cooling to room temperature, the reaction mixture was directly purified by column chromatographyon silica gel using petroleum ether, ethyl acetate, and methanol to afford the corresponding product.

3.5 Typical Procedures for the Radical Trapping Experiments.

A 4 mL screw-cap vial was charged with Co(acac)$_2$ (7.8 mg, 10 mol%), **L6** (5.4

mg, 10 mol%), radical scavengers (2 equiv.), and an oven-dried stirring bar. The vial was closed with a Teflon septum and cap and connected to the atmosphere via a needle. After **1a** (10 equiv.), $PhCF_3$ (1.5 mL, 0.2 M), NuH (1 equiv., 0.3 mmol), and DTBP (161 mg, 3 equiv.) were added with a syringe under nitrogen atmosphere, the vial was moved to an alloy plate and put into a Parr 4560 series autoclave (300 mL) under an argon atmosphere. At room temperature, the autoclave was flushed with CO two times and ethylene two times, then charged with 40 bar of CO and 10 bar ethylene. The autoclave was placed on a heating plate equipped with a magnetic stirrer and an aluminum block. The reaction mixture was heated to 120 ^{o}C for 20 h. After the reaction was complete, the autoclave was cooled down with ice water to room temperature and the pressure was released carefully. After cooling to room temperature, the reaction mixture was directly purified by column chromatographyon silica gel using petroleum ether, ethyl acetate, and methanol to afford the corresponding product.

3.6 Typical Procedures for the Radical Clock Experiments.

A 4 mL screw-cap vial was charged with $Co(acac)_2$ (7.8 mg, 10 mol%), **L6** (5.4 mg, 10 mol%), radical scavengers (2 equiv.), and an oven-dried stirring bar. The vial was closed with a Teflon septum and cap and connected to the atmosphere via a needle. After **1a** (10 equiv.), $PhCF_3$ (1.5 mL, 0.2 M), NuH (1 equiv., 0.3 mmol), corresponding alkenes (2 equiv.), and DTBP (161 mg, 3 equiv.) were added with a syringe under nitrogen atmosphere, the vial was moved to an alloy plate and put into a Parr 4560 series autoclave (300 mL) under an argon atmosphere. At room temperature, the autoclave was flushed with CO two times, then charged with 40 bar of CO. The autoclave was placed on a heating plate equipped with a magnetic stirrer and an aluminum block. The reaction mixture was heated to 120 ^{o}C for 20 h. After the reaction was complete, the autoclave was cooled down with ice water to room temperature and the pressure was released carefully. After cooling to room temperature, the reaction mixture was directly purified by column chromatographyon silica gel using petroleum ether, ethyl acetate, and methanol to afford the corresponding product.

3.7 Typical Procedures for the Competitive Experiments

A 4 mL screw-cap vial was charged with $Co(acac)_2$ (7.8 mg, 10 mol%), **L6** (5.4 mg, 10 mol%), and an oven-dried stirring bar. The vial was closed with a Teflon septum and cap and connected to the atmosphere via a needle. After **1** (10 equiv.), $PhCF_3$ (1.5 mL, 0.2 M), NuH (1 equiv., 0.3 mmol), 4-phenyl-1-butene (0.6 mmol, 2 equiv.), and DTBP (161 mg, 3 equiv.) were added with a syringe under nitrogen atmosphere, the vial was moved to an alloy plate and put into a Parr 4560 series autoclave (300 mL) under an argon atmosphere. At room temperature, the autoclave was flushed with CO two times and ethylene two times, then charged with 40 bar of CO and 10 bar ethylene. The autoclave was placed on a heating plate equipped with a magnetic stirrer and an aluminum block. The reaction mixture was heated to 120 ^{o}C for 20 h. After the reaction was complete, the autoclave was cooled down with ice water to room temperature and the pressure was released carefully. After cooling to room temperature, the reaction mixture was directly purified by column chromatographyon silica gel using petroleum ether, ethyl acetate, and methanol to afford the corresponding product.

A 4 mL screw-cap vial was charged with $Co(acac)_2$ (7.8 mg, 10 mol%), **L6** (5.4 mg, 10 mol%), and an oven-dried stirring bar. The vial was closed with a Teflon septum and cap and connected to the atmosphere via a needle. After dimethylacetamide and diethylacetamide (10 equiv.), $PhCF_3$ (1.5 mL, 0.2 M), NuH (1 equiv., 0.3 mmol), and DTBP (161 mg, 3 equiv.) were added with a syringe under nitrogen atmosphere, the vial was moved to an alloy plate and put into a Parr 4560 series autoclave (300 mL) under an argon atmosphere. At room temperature, the autoclave was flushed with CO two times and ethylene two times, then charged with 40 bar of CO and 10 bar ethylene. The autoclave was placed on a heating plate equipped with a magnetic stirrer and an aluminum block. The reaction mixture was heated to 120 ^{o}C for 20 h. After the reaction was complete, the autoclave was cooled down with ice water to room temperature and the pressure was released carefully. After cooling to room temperature, the reaction mixture was directly purified by column chromatographyon silica gel using petroleum ether, ethyl acetate, and methanol to afford the corresponding product.

3.8 Typical Procedures for the Background Experiments

A 4 mL screw-cap vial was charged with (*R,R*)-^{t}Bu-Pybox (32.8 mg, 5 mol%), and an oven-dried stirring bar. The vial was closed with a Teflon septum and cap and connected to the atmosphere via a needle. After **1** (10 equiv.), $PhCF_3$ (1.5 mL, 0.2 M), NuH (1 equiv., 0.3 mmol), 4-phenyl-1-butene (0.6 mmol, 2 equiv.), and DTBP (161 mg, 3 equiv.) were added with a syringe under nitrogen atmosphere, the vial was moved to an alloy plate and put into a Parr 4560 series autoclave (300 mL) under an argon atmosphere. At room temperature, the autoclave was flushed with CO two times, then charged with 40 bar of CO. The autoclave was placed on a heating plate equipped with a magnetic stirrer and an aluminum block. The reaction mixture was heated to 120 ^oC for 20 h. After the reaction was complete, the autoclave was cooled down with ice water to room temperature and the pressure was released carefully. After cooling to room temperature, the reaction mixture was directly purified by column chromatographyon silica gel using petroleum ether, ethyl acetate, and methanol to afford the corresponding product.

A 4 mL screw-cap vial was charged with $Co(acac)_2$ (3.9 mg, 5mol%), and an oven-dried stirring bar. The vial was closed with a Teflon septum and cap and connected to the atmosphere via a needle. After **1** (10 equiv.), $PhCF_3$ (1.5 mL, 0.2 M), NuH (1 equiv., 0.3 mmol), 4-phenyl-1-butene (0.6 mmol, 2 equiv.), and DTBP (161 mg, 3 equiv.) were added with a syringe under nitrogen atmosphere, the vial was moved to an alloy plate and put into a Parr 4560 series autoclave (300 mL) under an argon atmosphere. At room temperature, the autoclave was flushed with CO two times, then charged with 40 bar of CO. The autoclave was placed on a heating plate equipped with a magnetic stirrer and an aluminum block. The reaction mixture was heated to 120 ^oC for 20 h. After the reaction was complete, the autoclave was cooled down with ice water to room temperature and the pressure was released carefully. After cooling to room temperature, the reaction mixture was directly purified by column chromatographyon silica gel using petroleum ether, ethyl acetate, and methanol to afford the corresponding product.

A 4 mL screw-cap vial was charged with **1** (10 equiv.), $PhCF_3$ (1.5 mL, 0.2 M),

NuH (1 equiv., 0.3 mmol), 4-phenyl-1-butene (0.6 mmol, 2 equiv.), and DTBP (161 mg, 3 equiv.) were added with a syringe under nitrogen atmosphere, the vial was moved to an alloy plate and put into a Parr 4560 series autoclave (300 mL) under an argon atmosphere. At room temperature, the autoclave was flushed with CO two times, then charged with 40 bar of CO. The autoclave was placed on a heating plate equipped with a magnetic stirrer and an aluminum block. The reaction mixture was heated to 120 ^{o}C for 20 h. After the reaction was complete, the autoclave was cooled down with ice water to room temperature and the pressure was released carefully. After cooling to room temperature, the reaction mixture was directly purified by column chromatographyon silica gel using petroleum ether, ethyl acetate, and methanol to afford the corresponding product.

4 Supplementary Note 3

4.1 Radical-Trapping Products by GC-MS

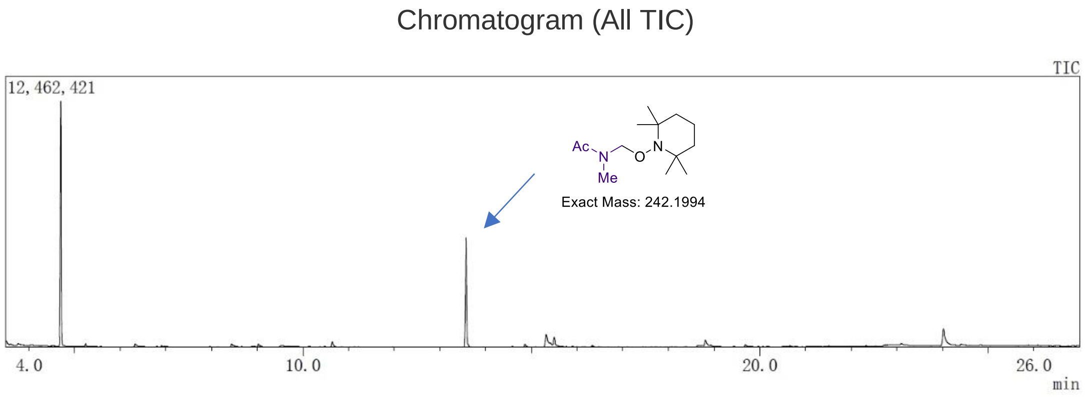

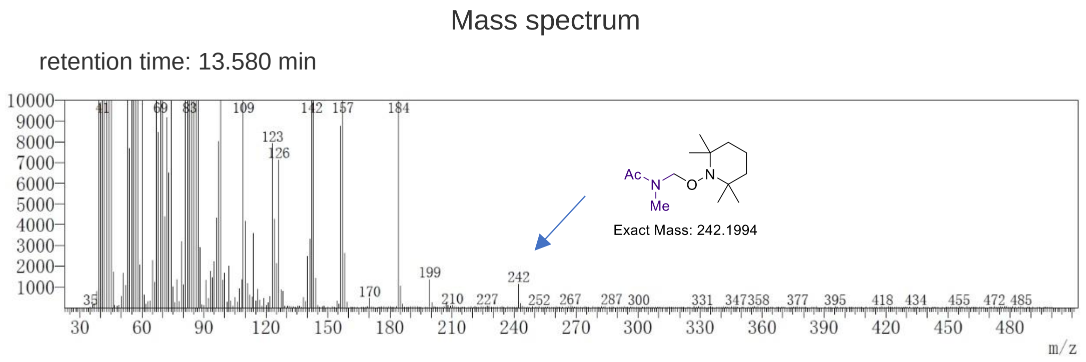

Supplementary Figure 2. Radical-Trapping Products by GC-MS

4.2 Spectroscopic Data of Products

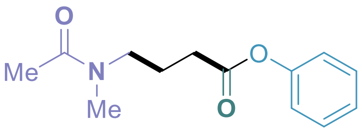

phenyl 4-(*N*-methylacetamido)butanoate (4a)

This reaction was conducted on a 0.3 mmol scale with the general procedure II. The crude product was purified by silica gel chromatography (PE/EA = 1:1) to afford the title compound as a colorless liquid (55.6 mg, 79% yield). The identity of the product was confirmed by ^{1}H NMR, ^{13}C NMR, and HRMS. Analysis of NMR spectra revealed the presence of two amide bond rotamers. These conformational isomers have been observed and studied in similar compounds.[1] The minor distinguishable rotamer peak is denoted with *.

^{1}H NMR (400 MHz, CDCl$_3$) δ 7.42 – 7.31 (m, 2H), 7.25 – 7.17 (m, 1H), 7.12 – 7.00 (m, 2H), 3.46/3.39* (t, J = 7.2 Hz, 2H), 2.99/2.93* (s, 3H), 2.62 – 2.50 (m, 2H), 2.11/2.06* (s, 3H), 2.04 – 1.88 (m, 2H).

^{13}C NMR (101 MHz, CDCl$_3$) δ 171.7/170.7*, 171.2/170.5*, 150.6/150.4*, 129.4*/129.3, 125.9/125.7*, 121.5/121.3*, 49.7*/46.5, 36.0/33.1*, 31.4/30.8*, 23.2*/22.3, 21.8/21.1*.

HRMS (ESI-TOF) m/z: [M + H]+ Calcd for $C_{13}H_{18}NO_3$ 236.1281; Found: 236.1287.

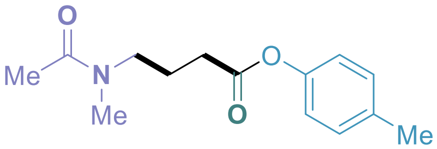

***p*-tolyl 4-(*N*-methylacetamido)butanoate (4b)**

This reaction was conducted on a 0.3 mmol scale with the general procedure II. The crude product was purified by silica gel chromatography (PE/EA = 1:1) to afford the title compound as a colorless liquid (53.8 mg, 72% yield). The identity of the product was confirmed by ^{1}H NMR, ^{13}C NMR, and HRMS. Analysis of NMR spectra revealed the presence of two amide bond rotamers. These conformational isomers have been observed and studied in similar compounds.[1] The minor distinguishable rotamer peak is denoted with *.

^{1}H NMR (400 MHz, CDCl$_3$) δ 7.20 – 7.11 (m, 2H), 7.00 – 6.90 (m, 2H), 3.46/3.39* (t, J = 7.2 Hz, 2H), 3.00/2.93* (s, 3H), 2.62 – 2.50 (m, 2H), 2.33*/2.32 (s, 3H), 2.11*/2.07 (s, 3H), 2.03 – 1.91 (m, 2H).

^{13}C NMR (101 MHz, CDCl$_3$) δ 171.9/170.8*, 171.4/170.5*, 148.4/148.1*, 135.6*/135.4, 130.0*/129.9, 121.1/121.0*, 49.7*/46.6, 36.1/33.1*, 31.4/30.8*, 23.2*/22.4, 21.8/21.1*, 20.8.

HRMS (ESI-TOF) m/z: [M + H]+ Calcd for $C_{14}H_{20}NO_3$ 250.1438; Found: 250.1433.

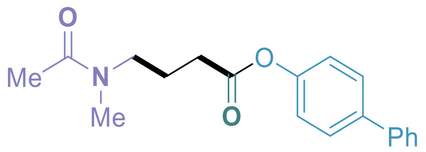

[1,1'-biphenyl]-4-yl 4-(*N*-methylacetamido)butanoate (4c)

This reaction was conducted on a 0.3 mmol scale with the general procedure II. The crude product was purified by silica gel chromatography (PE/EA = 1:1) to afford the title compound as a colorless liquid (69.1 mg, 74% yield). The identity of the product was confirmed by ^{1}H NMR, ^{13}C NMR, and HRMS. Analysis of NMR spectra revealed the presence of two amide bond rotamers. These conformational isomers have been observed and studied in similar compounds.[1] The minor distinguishable rotamer peak is denoted with *.

^{1}H NMR (400 MHz, CDCl$_3$) δ 7.63 – 7.52 (m, 4H), 7.50 – 7.38 (m, 2H), 7.38 – 7.30 (m, 1H), 7.18 – 7.11 (m, 2H), 3.49/3.41* (t, J = 7.1 Hz, 2H), 3.01/2.95* (s, 3H), 2.69 – 2.55 (m, 2H), 2.13*/2.09 (s, 3H), 2.07 – 1.91 (m, 2H).

^{13}C NMR (101 MHz, CDCl$_3$) δ 171.8/171.3*, 170.7/170.4*, 150.0/149.8*, 140.3/140.1*, 139.1*/138.9, 128.7*/128.7, 128.2*/128.1, 127.3*/127.2, 127.1*/127.0, 121.7/121.6*, 49.7*/46.5, 36.0/33.1*, 31.5/30.8*, 28.0*/23.2, 22.3/21.8*.

HRMS (ESI-TOF) m/z: [M + H]+ Calcd for $C_{19}H_{22}NO_3$ 312.1594; Found: 312.1598.

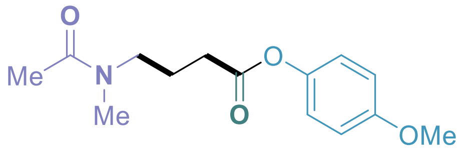

4-methoxyphenyl 4-(*N*-methylacetamido)butanoate (4d)

This reaction was conducted on a 0.3 mmol scale with the general procedure II. The crude product was purified by silica gel chromatography (PE/EA = 1:3) to afford the title compound as a colorless liquid (42.2 mg, 53% yield). The identity of the product was confirmed by ^{1}H NMR, ^{13}C NMR, and HRMS. Analysis of NMR spectra revealed the presence of two amide bond rotamers. These conformational isomers have been observed and studied in similar compounds.[1] The minor distinguishable rotamer peak is denoted with *.

^{1}H NMR (400 MHz, CDCl$_3$) δ 7.09 – 6.95 (m, 2H), 6.94 – 6.75 (m, 2H), 3.78*/3.77 (s, 3H), 3.46/3.39* (t, J = 7.2 Hz, 2H), 3.00/2.93* (s, 3H), 2.62 – 2.46 (m, 2H), 2.11*/2.07 (s, 3H), 2.02 – 1.84 (m, 2H).

^{13}C NMR (101 MHz, CDCl$_3$) δ 172.1/170.7*, 171.6/170.5*, 157.3*/157.1, 144.1/143.8*, 122.2/122.1*, 114.5*/114.4, 55.5*/55.5, 49.7*/46.5, 36.1/33.1*, 31.4/30.7*, 23.2/22.4*, 21.8/21.2*.

HRMS (ESI-TOF) m/z: [M + H]+ Calcd for $C_{14}H_{20}NO_4$ 266.1387; Found: 266.1392.

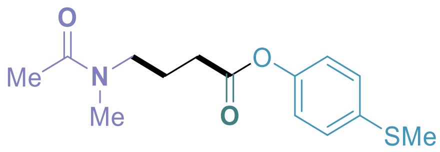

4-(methylthio)phenyl 4-(*N*-methylacetamido)butanoate (4e)

This reaction was conducted on a 0.3 mmol scale with the general procedure II. The crude product was purified by silica gel chromatography (PE/EA = 1:3) to afford the title compound as a colorless liquid (76.7 mg, 91% yield). The identity of the product was confirmed by ^{1}H NMR, ^{13}C NMR, and HRMS. Analysis of NMR spectra revealed the presence of two amide bond rotamers. These conformational isomers have been observed and studied in similar compounds.[1] The minor distinguishable rotamer peak is denoted with *.

^{1}H NMR (400 MHz, CDCl$_3$) δ 7.69 – 7.15 (m, 2H), 7.07 – 7.00 (m, 2H), 3.47/3.40* (t, J = 7.1 Hz, 2H), 3.01/2.94* (s, 3H), 2.61 – 2.55 (m, 2H), 2.47*/2.47 (s, 3H), 2.12*/2.08 (s, 3H), 2.06 – 1.90 (m, 2H).

^{13}C NMR (101 MHz, CDCl$_3$) δ 171.7/171.2*, 170.7/170.4*, 148.3/148.0*, 135.9*/135.5, 127.8/127.8*, 121.9/121.8*, 49.6*/46.4, 36.0/33.0*, 31.3/30.7*, 23.1*/22.2, 21.8/21.1*, 16.3/16.3*.

HRMS (ESI-TOF) m/z: [M + H]+ Calcd for $C_{14}H_{20}NO_3S$ 282.1158; Found: 282.1160.

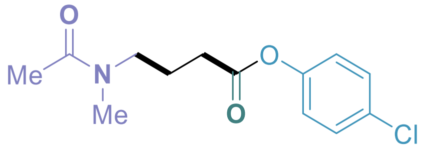

4-chlorophenyl 4-(*N*-methylacetamido)butanoate (4f)

This reaction was conducted on a 0.3 mmol scale with the general procedure II. The crude product was purified by silica gel chromatography (PE/EA = 1:1) to afford the title compound as a colorless liquid (62.2 mg, 77% yield). The identity of the product was confirmed by ^{1}H NMR, ^{13}C NMR, and HRMS. Analysis of NMR spectra revealed the presence of two amide bond rotamers. These conformational isomers have been observed and studied in similar compounds.[1] The minor distinguishable rotamer peak is denoted with *.

^{1}H NMR (400 MHz, CDCl$_3$) δ 7.36 – 7.28 (m, 2H), 7.07 – 6.98 (m, 2H), 3.45/3.38* (t, J = 7.1 Hz, 2H), 2.98/2.92* (s, 3H), 2.63 – 2.48 (m, 2H), 2.10*/2.05 (s, 3H), 2.01 – 1.86 (m, 2H).

^{13}C NMR (101 MHz, CDCl$_3$) δ 171.5/171.0*, 170.7/170.4*, 149.1/148.8*, 131.3*/131.0, 129.5*/129.3, 122.9/122.7*, 49.6*/46.4, 36.0/33.1*, 31.3/30.7*, 23.1*/22.2, 21.8/21.1*.

HRMS (ESI-TOF) m/z: [M + H]+ Calcd for $C_{13}H_{17}NO_3Cl$ 270.0891; Found: 270.0888.

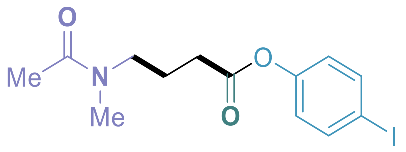

4-iodophenyl 4-(*N*-methylacetamido)butanoate (4g)

This reaction was conducted on a 0.3 mmol scale with the general procedure II. The crude product was purified by silica gel chromatography (PE/EA = 1:1) to afford the title compound as a colorless liquid (68.2 mg, 63% yield). The identity of the product was confirmed by ^{1}H NMR, ^{13}C NMR, and HRMS. Analysis of NMR spectra revealed the presence of two amide bond rotamers. These conformational isomers have been observed and studied in similar compounds.[1] The minor distinguishable rotamer peak is denoted with *.

^{1}H NMR (400 MHz, $CDCl_3$) δ 7.75 – 7.60 (m, 2H), 7.05 – 6.80 (m, 2H), 3.46/3.39* (t, J = 7.1 Hz, 2H), 3.00/2.93* (s, 3H), 2.66 – 2.45 (m, 2H), 2.11*/2.07 (s, 3H), 2.03 – 1.80 (m, 2H).

^{13}C NMR (101 MHz, $CDCl_3$) δ 171.4/170.9*, 170.8/170.4*, 150.5/150.2*, 138.5*/138.4, 123.7/123.5*, 90.0*/89.8, 49.6*/46.5, 36.0/33.1*, 31.4/30.8*, 23.1*/22.3, 21.8/21.2*.

HRMS (ESI-TOF) m/z: [M + H]+ Calcd for $C_{13}H_{17}NO_3I$ 362.0248; Found: 262.0252

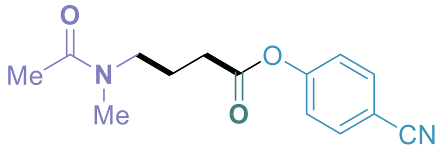

4-cyanophenyl 4-(*N*-methylacetamido)butanoate (4h)

This reaction was conducted on a 0.3 mmol scale with the general procedure II. The crude product was purified by silica gel chromatography (PE/EA = 1:5) to afford the title compound as a colorless liquid (62.4 mg, 80% yield). The identity of the product was confirmed by ^{1}H NMR, ^{13}C NMR, and HRMS. Analysis of NMR spectra revealed the presence of two amide bond rotamers. These conformational isomers have been observed and studied in similar compounds.[1] The minor distinguishable rotamer peak is denoted with *.

^{1}H NMR (400 MHz, $CDCl_3$) δ 7.85 – 7.59 (m, 2H), 7.47 – 7.05 (m, 2H), 3.49/3.42* (t, J = 7.0 Hz, 2H), 3.02/2.95* (s, 3H), 2.72 – 2.49 (m, 2H), 2.13*/2.09 (s, 3H), 2.07 – 1.85 (m, 2H).

^{13}C NMR (101 MHz, $CDCl_3$) δ 170.9/170.9*, 170.5/170.5*, 154.0/153.7*, 133.8*/133.7, 122.8/122.7*, 118.3/118.1*, 109.9*/109.6, 49.7*/46.3, 36.1/33.2*, 31.4/30.9*, 23.1*/22.2, 21.9/21.2*.

HRMS (ESI-TOF) m/z: [M + H]+ Calcd for $C_{14}H_{17}N_2O_3I$ 261.1234; Found: 261.1238.

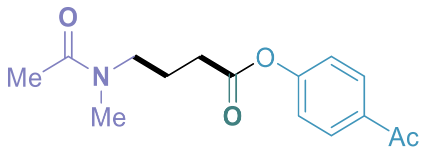

4-acetylphenyl 4-(*N*-methylacetamido)butanoate (4i)

This reaction was conducted on a 0.3 mmol scale with the general procedure II. The crude product was purified by silica gel chromatography (PE/EA = 1:3) to afford the title compound as a colorless liquid (70.6 mg, 85% yield). The identity of the product was confirmed by ^{1}H NMR, ^{13}C NMR, and HRMS. Analysis of NMR spectra revealed the presence of two amide bond rotamers. These conformational isomers have been observed and studied in similar compounds.[1] The minor distinguishable rotamer peak is denoted with *.

^{1}H NMR (400 MHz, $CDCl_3$) δ 8.07 – 7.91 (m, 2H), 7.25 – 7.09 (m, 2H), 3.46/3.39* (t, J = 7.1 Hz, 2H), 3.00/2.92* (s, 3H), 2.72 – 2.54 (m, 5H), 2.10*/2.06 (s, 3H), 2.04 – 1.89 (m, 2H).

^{13}C NMR (101 MHz, $CDCl_3$) δ 196.8/196.7*, 171.2/170.8*, 170.7/170.4*, 154.3*/154.0, 134.8*/134.6, 129.9/129.8*, 121.7/121.6*, 49.6*/46.4, 36.0/33.1*, 31.4/30.8*, 26.5, 23.1*/22.2, 21.8/21.1*.

HRMS (ESI-TOF) m/z: [M + H]+ Calcd for $C_{15}H_{20}NO_4$ 278.1387; Found: 278.1390.

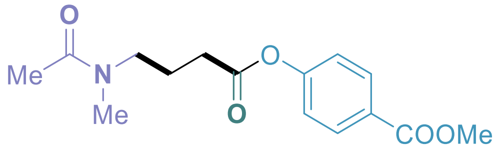

methyl 4-((4-(*N*-methylacetamido)butanoyl)oxy)benzoate (4j)

This reaction was conducted on a 0.3 mmol scale with the general procedure II. The crude product was purified by silica gel chromatography (PE/EA = 1:3) to afford the title compound as a colorless liquid (77.4 mg, 88% yield). The identity of the product was confirmed by ^{1}H NMR, ^{13}C NMR, and HRMS. Analysis of NMR spectra revealed the presence of two amide bond rotamers. These conformational isomers have been observed and studied in similar compounds.[1] The minor distinguishable rotamer peak is denoted with *.

^{1}H NMR (400 MHz, $CDCl_3$) δ 8.18 – 7.98 (m, 2H), 7.19 – 7.10 (m, 2H), 3.89*/3.88 (s, 3H), 3.46/3.39* (t, J = 7.1 Hz, 2H), 2.99/2.92* (s, 3H), 2.72 – 2.50 (m, 2H), 2.10*/2.06 (s, 3H), 2.03 – 1.88 (m, 2H).

^{13}C NMR (101 MHz, $CDCl_3$) δ 171.2/170.8*, 170.7/170.4*, 166.2/166.1*, 154.2*/153.9, 131.2*/131.1, 127.8/127.6*, 121.5/121.4*, 52.1*/52.1, 49.6*/46.4, 36.0/33.1*, 31.4/30.8*, 23.1*/22.2, 21./21.1*.

HRMS (ESI-TOF) m/z: [M + H]+ Calcd for $C_{15}H_{20}NO_5$ 294.1336; Found: 294.1343.

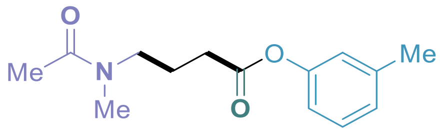

***m*-tolyl 4-(*N*-methylacetamido)butanoate (4k)**

This reaction was conducted on a 0.3 mmol scale with the general procedure II. The crude product was purified by silica gel chromatography (PE/EA = 1:1) to afford the title compound as a colorless liquid (49.3 mg, 66% yield). The identity of the product was confirmed by ^{1}H NMR, ^{13}C NMR, and HRMS. Analysis of NMR spectra revealed the presence of two amide bond rotamers. These conformational isomers have been observed and studied in similar compounds.[1] The minor distinguishable rotamer peak is denoted with *.

^{1}H NMR (400 MHz, CDCl$_3$) δ 7.31 – 7.20 (m, 1H), 7.04 (t, J = 8.2 Hz, 1H), 6.96 – 6.84 (m, 2H), 3.48/3.41* (t, J = 7.2 Hz, 2H), 3.02/2.95* (s, 3H), 2.66 – 2.51 (m, 2H), 2.36*/2.35 (s, 3H), 2.13*/2.09 (s, 3H), 2.04 – 1.92 (m, 2H).

^{13}C NMR (101 MHz, CDCl$_3$) δ 171.9/171.4*, 170.7/170.5*, 150.6/150.4*, 139.7*/139.6, 129.2*/129.1, 126.8*/126.6, 122.1/ 121.9*, 118.4/118.3*, 49.7*/46.6, 36.1/33.2*, 31.5/30.8*, 23.2*/22.4, 21.9/21.3*, 21.3*/21.2.

HRMS (ESI-TOF) m/z: [M + H]+ Calcd for $C_{14}H_{20}NO_3$ 250.1438; Found: 250.1440.

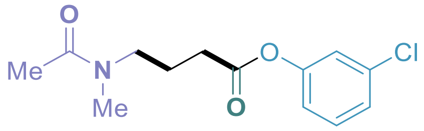

3-chlorophenyl 4-(*N*-methylacetamido)butanoate (4l)

This reaction was conducted on a 0.3 mmol scale with the general procedure II. The crude product was purified by silica gel chromatography (PE/EA = 1:1) to afford the title compound as a colorless liquid (69.4 mg, 86% yield). The identity of the product was confirmed by ^{1}H NMR, ^{13}C NMR, and HRMS. Analysis of NMR spectra revealed the presence of two amide bond rotamers. These conformational isomers have been observed and studied in similar compounds.[1] The minor distinguishable rotamer peak is denoted with *.

^{1}H NMR (400 MHz, CDCl$_3$) δ 7.34 – 7.27 (m, 1H), 7.25 – 7.19 (m, 1H), 7.17 – 7.11 (m, 1H), 7.07 – 6.95 (m, 1H), 3.48/3.40* (t, J = 7.1 Hz, 2H), 3.01/2.94* (s, 3H), 2.64 – 2.53 (m, 2H), 2.12*/2.08 (s, 3H), 2.04 – 1.88 (m, 2H).

^{13}C NMR (101 MHz, CDCl$_3$) δ 171.3/170.8*, 170.7/170.4*, 151.0/150.8*, 134.6*/134.5, 130.2*/130.0, 126.2*/125.9, 122.1/122.0*, 119.9/119.7*, 49.6*/46.4, 36.0/33.1*, 31.3/30.7*, 23.1*/22.2, 21.8/21.1*.

HRMS (ESI-TOF) m/z: [M + H]+ Calcd for $C_{13}H_{17}NO_3Cl$ 270.0891; Found: 270.0895.

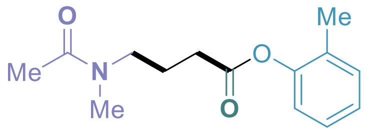

o-tolyl 4-(N-methylacetamido)butanoate (4m)

This reaction was conducted on a 0.3 mmol scale with the general procedure II. The crude product was purified by silica gel chromatography (PE/EA = 1:1) to afford the title compound as a colorless liquid (30.6 mg, 41% yield). The identity of the product was confirmed by ^{1}H NMR, ^{13}C NMR, and HRMS. Analysis of NMR spectra revealed the presence of two amide bond rotamers. These conformational isomers have been observed and studied in similar compounds.[1] The minor distinguishable rotamer peak is denoted with *.

^{1}H NMR (400 MHz, CDCl$_3$) δ 7.36 – 7.07 (m, 3H), 6.99 (t, J = 7.2 Hz, 1H), 3.48/3.41* (t, J = 7.2 Hz, 2H), 3.02/2.95* (s, 3H), 2.76 – 2.51 (m, 2H), 2.17 (s, 3H), 2.12*/2.08 (s, 3H), 2.07 – 1.92 (m, 2H).

^{13}C NMR (101 MHz, CDCl$_3$) δ 171.5/171.0*, 170.7/170.5*, 149.2/149.1*, 131.2*/131.1, 130.0/129.8*, 127.0*/126.9, 126.2*/126.0, 121.8/121.7*, 49.8*/46.7, 36.1/33.2*, 31.3/30.6*, 23.3/22.5*, 21.8/21.2*, 16.1.

HRMS (ESI-TOF) m/z: [M + H]+ Calcd for $C_{14}H_{20}NO_3$ 250.1438; Found: 250.1438.

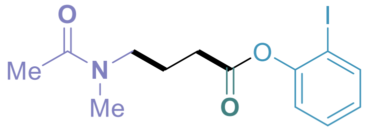

2-iodophenyl 4-(N-methylacetamido)butanoate (4n)

This reaction was conducted on a 0.3 mmol scale with the general procedure II. The crude product was purified by silica gel chromatography (PE/EA = 1:1) to afford the title compound as a colorless liquid (87.7 mg, 81% yield). The identity of the product was confirmed by ^{1}H NMR, ^{13}C NMR, and HRMS. Analysis of NMR spectra revealed the presence of two amide bond rotamers. These conformational isomers have been observed and studied in similar compounds.[1] The minor distinguishable rotamer peak is denoted with *.

^{1}H NMR (400 MHz, CDCl$_3$) δ 7.79 (t, J = 6.4 Hz, 1H), 7.42 – 7.28 (m, 1H), 7.07 (t, J = 8.4 Hz, 1H), 7.01 – 6.89 (m, 1H), 3.48/3.41* (t, J = 7.2 Hz, 2H), 2.99/2.92* (s, 3H), 2.73 – 2.58 (m, 2H), 2.10*/2.06 (s, 3H), 2.05 – 1.87 (m, 2H).

^{13}C NMR (101 MHz, CDCl$_3$) δ 170.8/170.6*, 170.4*/170.4, 151.0/150.8*, 139.3*/139.2, 129.5*/129.4, 127.7*/127.5, 123.0/122.8*, 90.3/90.3*, 49.6*/46.5, 36.0/33.1*, 31.5/30.9*, 23.1*/22.2, 21.8/1.2*.

HRMS (ESI-TOF) m/z: [M + H]+ Calcd for $C_{13}H_{17}NO_3I$ 362.0248; Found: 362.0251.

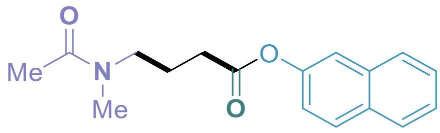

naphthalen-2-yl 4-(*N*-methylacetamido)butanoate (4o)

This reaction was conducted on a 0.3 mmol scale with the general procedure II. The crude product was purified by silica gel chromatography (PE/EA = 1:1) to afford the title compound as a colorless liquid (63.3 mg, 74% yield). The identity of the product was confirmed by ^{1}H NMR, ^{13}C NMR, and HRMS. Analysis of NMR spectra revealed the presence of two amide bond rotamers. These conformational isomers have been observed and studied in similar compounds.[1] The minor distinguishable rotamer peak is denoted with *.

^{1}H NMR (400 MHz, CDCl$_3$) δ 7.87 – 7.75 (m, 3H), 7.63 – 7.53 (m, 1H), 7.52 – 7.42 (m, 2H), 7.34 – 7.18 (m, 1H), 3.50/3.41* (t, J = 7.1 Hz, 2H), 3.00/2.95* (s, 3H), 2.67 – 2.48 (m, 2H), 2.13*/2.08 (s, 3H), 2.08 – 1.90 (m, 2H).

^{13}C NMR (101 MHz, CDCl$_3$) δ 171.9/170.8*, 171.4/170.5*, 148.2/148.0*, 133.7/133.6*, 131.4/131.5*, 129.5*/129.3, 127.7*/127.7, 127.5, 126.6*/126.5, 125.8*/125.6, 121.1/120.8*, 118.4/118.3*, 49.7*/46.5, 36.0/33.1*, 31.5/30.9*, 23.2*/22.3, 21.8/21.2*.

HRMS (ESI-TOF) m/z: [M + H]+ Calcd for $C_{17}H_{20}NO_3$ 286.1438; Found: 186.1444.

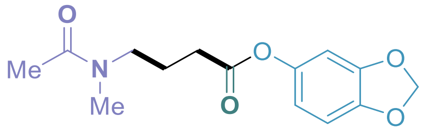

benzo[d][1,3]dioxol-5-yl 4-(*N*-methylacetamido)butanoate (4p)

This reaction was conducted on a 0.3 mmol scale with the general procedure II. The crude product was purified by silica gel chromatography (PE/EA = 1:3) to afford the title compound as a colorless liquid (39.3 mg, 47% yield). The identity of the product was confirmed by ^{1}H NMR, ^{13}C NMR, and HRMS. Analysis of NMR spectra revealed the presence of two amide bond rotamers. These conformational isomers have been observed and studied in similar compounds.[1] The minor distinguishable rotamer peak is denoted with *.

^{1}H NMR (400 MHz, CDCl$_3$) δ 6.80 – 6.71 (m, 1H), 6.63 – 6.56 (m, 1H), 6.53 – 6.43 (m, 1H), 5.97*/5.96 (s, 2H), 3.46/3.38* (t, J = 7.2 Hz, 2H), 3.00/2.93* (s, 3H), 2.60 – 2.43 (m, 2H), 2.11*/2.07 (s, 3H), 2.04 – 1.84 (m, 2H).

^{13}C NMR (101 MHz, CDCl$_3$) δ 172.0/171.6*, 170.8/170.5*, 148.0*/147.9, 145.4*/145.3, 144.9/144.7*, 113.8/113.7*, 108.0*/107.9, 103.7/103.5*, 101.7*/101.6, 49.7*/46.5, 36.1/33.1*, 31.4/30.7*, 23.2*/22.3, 21.8/21.2*.

HRMS (ESI-TOF) m/z: [M + H]+ Calcd for $C_{14}H_{18}NO_5$ 280.1179; Found: 280.1181.

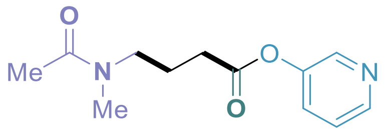

pyridin-3-yl 4-(*N*-methylacetamido)butanoate (4q)

This reaction was conducted on a 0.3 mmol scale with the general procedure II. The crude product was purified by silica gel chromatography (PE/EA = 1:3) to afford the title compound as a colorless liquid (50.3 mg, 71% yield). The identity of the product was confirmed by ^{1}H NMR, ^{13}C NMR, and HRMS. Analysis of NMR spectra revealed the presence of two amide bond rotamers. These conformational isomers have been observed and studied in similar compounds.[1] The minor distinguishable rotamer peak is denoted with *.

^{1}H NMR (400 MHz, CDCl$_3$) δ 8.57 – 8.46 (m, 1H), 8.44*/8.43 (s, 1H), 7.59 – 7.42 (m, 1H), 7.42 – 7.18 (m, 1H), 3.50/3.42* (t, J = 7.0 Hz, 2H), 3.03/2.95* (s, 3H), 2.86 – 2.56 (m, 2H), 2.14*/2.10 (s, 3H), 2.06 – 1.93 (m, 2H).

^{13}C NMR (101 MHz, CDCl$_3$) δ 171.2/170.9*, 170.7/170.5*, 147.3*/147.1, 146.9*/146.6, 143.2/143.0*, 129.4/129.2*, 123.9*/123.9, 49.6*/46.4, 36.0/33.1*, 31.2/30.7*, 23.0*/22.1, 21.7/21.1*.

HRMS (ESI-TOF) m/z: [M + H]+ Calcd for $C_{12}H_{17}N_2O_3$ 237.1234; Found: 237.1238.

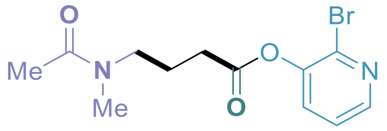

2-bromopyridin-3-yl 4-(*N*-methylacetamido)butanoate (4r)

This reaction was conducted on a 0.3 mmol scale with the general procedure II. The crude product was purified by silica gel chromatography (PE/EA = 1:3) to afford the title compound as a colorless liquid (59.3 mg, 63% yield). The identity of the product was confirmed by ^{1}H NMR, ^{13}C NMR, and HRMS. Analysis of NMR spectra revealed the presence of two amide bond rotamers. These conformational isomers have been observed and studied in similar compounds.[1] The minor distinguishable rotamer peak is denoted with *.

^{1}H NMR (400 MHz, CDCl$_3$) δ 8.32 – 8.25 (m, 1H), 7.58 – 7.47 (m, 1H), 7.42 – 7.28 (m, 1H), 3.51/3.45* (t, J = 7.1 Hz, 2H), 3.04/2.95* (s, 3H), 2.78 – 2.54 (m, 2H), 2.14*/2.10 (s, 3H), 2.09 – 1.97 (m, 2H).

^{13}C NMR (101 MHz, CDCl$_3$) δ 170.7/170.3*, 170.2/169.9*, 147.1*/146.9, 145.3/136.3*, 131.7/131.5*, 123.5*/123.5, 49.5*/46.2, 36.0/33.0*, 31.1/30.6*, 22.9*/22.0, 21.7/21.1*.

HRMS (ESI-TOF) m/z: [M + H]+ Calcd for $C_{12}H_{16}N_2O_3Br$ 315.0339; Found: 315.0342.

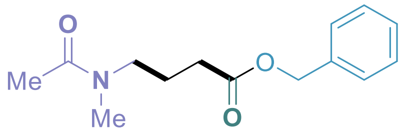

benzyl 4-(*N*-methylacetamido)butanoate (4s)

This reaction was conducted on a 0.3 mmol scale with the general procedure II. The crude product was purified by silica gel chromatography (PE/EA = 1:1) to afford the title compound as a colorless liquid (59.8 mg, 80% yield). The identity of the product was confirmed by ^{1}H NMR, ^{13}C NMR, and HRMS. Analysis of NMR spectra revealed the presence of two amide bond rotamers. These conformational isomers` have been observed and studied in similar compounds.[1] The minor distinguishable rotamer peak is denoted with *.

^{1}H NMR (400 MHz, CDCl$_3$) δ 7.37 – 7.21 (m, 4H), 5.11/5.10* (s, 2H), 3.53 – 3.33/3.33* – 3.21* (m, 2H), 2.94/2.87* (s, 3H), 2.49 – 2.26 (m, 2H), 2.03*/2.02 (s, 3H), 1.92 – 1.81 (m, 2H).

^{13}C NMR (101 MHz, CDCl$_3$) δ 172.9/172.4*, 170.6/170.4*, 135.8/135.6*, 128.5*/128.5, 128.4*/128.3, 128.2*/128.1, 66.5*/66.2, 49.7*/46.6, 36.0/33.0*, 31.4/30.8*, 23.2*/22.4, 21.8/21.0*.

HRMS (ESI-TOF) m/z: [M + H]+ Calcd for $C_{14}H_{20}NO_3$ 250.1438; Found: 250.1435.

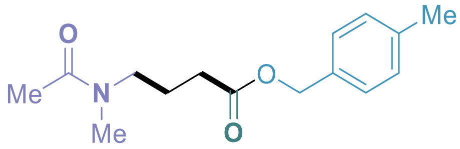

4-methylbenzyl 4-(*N*-methylacetamido)butanoate (4t)

This reaction was conducted on a 0.3 mmol scale with the general procedure II. The crude product was purified by silica gel chromatography (PE/EA = 1:1) to afford the title compound as a colorless liquid (45.0 mg, 57% yield). The identity of the product was confirmed by ^{1}H NMR, ^{13}C NMR, and HRMS. Analysis of NMR spectra revealed the presence of two amide bond rotamers. These conformational isomers have been observed and studied in similar compounds.[1] The minor distinguishable rotamer peak is denoted with *.

^{1}H NMR (400 MHz, CDCl$_3$) δ 7.22 (d, J = 6.6 Hz, 2H), 7.19 – 7.12 (m, 2H), 5.06*/5.05 (s, 2H), 3.70 – 3.33/3.32* – 3.19* (m, 2H), 2.94/2.86* (s, 3H), 2.52 – 2.28 (m, 5H), 2.02/2.01* (s, 3H), 1.94 – 1.79 (m, 2H).

^{13}C NMR (101 MHz, CDCl$_3$) δ 172.9/172.4*, 170.5/170.4*, 138.2*/138.0, 132.8/132.5*, 129.2*/129.1, 128.5*/128.3, 66.4*/66.1, 49.7*/46.5, 36.0/33.0*, 31.4/30.8*, 28.0*/23.2, 22.4*/21.7, 21.1/21.0*.

HRMS (ESI-TOF) m/z: [M + H]+ Calcd for $C_{15}H_{22}NO_3$ 264.1594; Found: 264.1593.

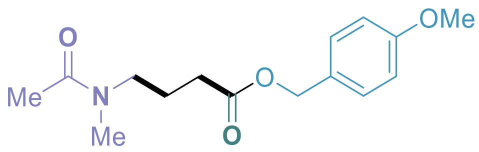

4-methoxybenzyl 4-(*N*-methylacetamido)butanoate (4u)

This reaction was conducted on a 0.3 mmol scale with the general procedure II. The crude product was purified by silica gel chromatography (PE/EA = 1:3) to afford the title compound as a colorless liquid (61.1 mg, 73% yield). The identity of the product was confirmed by ^{1}H NMR, ^{13}C NMR, and HRMS. Analysis of NMR spectra revealed the presence of two amide bond rotamers. These conformational isomers have been observed and studied in similar compounds.[1] The minor distinguishable rotamer peak is denoted with *.

^{1}H NMR (400 MHz, $CDCl_3$) δ 7.29 (d, J = 8.6 Hz, 2H), 7.04 – 6.85 (m, 2H), 5.06*/5.05 (s, 2H), 3.80 (s, 3H), 3.47 – 3.34/3.34* – 3.18* (m, 2H), 2.96/2.88* (s, 3H), 2.60 – 2.27 (m, 2H), 2.05/2.03* (s, 3H), 1.96 – 1.79 (m, 2H).

^{13}C NMR (101 MHz, $CDCl_3$) δ 173.0/172.4*, 170.6/170.4*, 159.7*/159.5, 130.2*/130.0, 128.0/127.7*, 113.9*/113.8, 66.3*/66.0, 55.2, 49.7*/46.5, 36.0/33.0*, 31.4/30.8*, 23.2*/22.4, 21.7/21.0*.

HRMS (ESI-TOF) m/z: [M + H]+ Calcd for $C_{15}H_{22}NO_4$ 302.1363; Found: 302.1363.

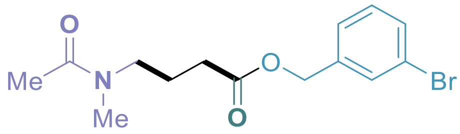

3-bromobenzyl 4-(*N*-methylacetamido)butanoate (4v)

This reaction was conducted on a 0.3 mmol scale with the general procedure II. The crude product was purified by silica gel chromatography (PE/EA = 1:1) to afford the title compound as a colorless liquid (70.6 mg, 72% yield). The identity of the product was confirmed by ^{1}H NMR, ^{13}C NMR, and HRMS. Analysis of NMR spectra revealed the presence of two amide bond rotamers. These conformational isomers have been observed and studied in similar compounds.[1] The minor distinguishable rotamer peak is denoted with *.

^{1}H NMR (400 MHz, $CDCl_3$) δ 7.50 (s, 1H), 7.49 – 7.38 (m, 1H), 7.37 – 7.01 (m, 2H), 5.09*/5.07 (s, 2H), 3.59 – 3.35/3.35* – 3.23* (m, 2H), 2.98/2.90* (s, 3H), 2.52 – 2.32 (m, 2H), 2.06 (s, 3H), 1.99 – 1.74 (m, 2H).

^{13}C NMR (101 MHz, CDCl$_3$) δ 172.7/172.2*, 170.6/170.3*, 138.1/137.8*, 131.3*/131.1, 131.1*/130.9, 130.1*/130.0, 126.7*/126.5, 122.5*/122.4, 65.4*/65.1, 49.6*/46.4, 36.0/33.0*, 31.2/30.6*, 23.2*/22.3, 21.7/21.0*.
HRMS (ESI-TOF) m/z: [M + H]+ Calcd for $C_{14}H_{19}NO_3Br$ 328.0543; Found: 328.0546.

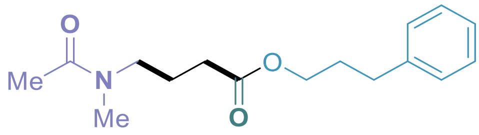

3-phenylpropyl 4-(*N*-methylacetamido)butanoate (4w)

This reaction was conducted on a 0.3 mmol scale with the general procedure II. The crude product was purified by silica gel chromatography (PE/EA = 1:1) to afford the title compound as a colorless liquid (53.8 mg, 79% yield). The identity of the product was confirmed by ^{1}H NMR, ^{13}C NMR, and HRMS. Analysis of NMR spectra revealed the presence of two amide bond rotamers. These conformational isomers have been observed and studied in similar compounds.[1] The minor distinguishable rotamer peak is denoted with *.

^{1}H NMR (400 MHz, CDCl$_3$) δ 7.32 – 7.24 (m, 2H), 7.24 – 7.15 (m, 3H), 4.19 – 3.94 (m, 2H), 3.54 – 3.36/3.36* – 3.24* (m, 2H), 2.98/2.91* (s, 3H), 2.68 (t, J = 6.5 Hz, 2H), 2.32 (t, J = 7.5 Hz, 2H), 2.09*/2.06 (s, 3H), 2.04 – 1.90 (m, 2H), 1.91 – 1.74 (m, 2H).
^{13}C NMR (101 MHz, CDCl$_3$) δ 173.0/172.5*, 170.4/170.3*, 141.0/140.8*, 128.3*/128.2, 128.2/128.1*, 125.9*/125.8, 63.9*/63.6, 49.6*/46.5, 35.9/32.9*, 32.0*/32.0, 31.2/30.5*, 30.0/29.9*, 23.1*/22.3, 21.7/21.0*.
HRMS (ESI-TOF) m/z: [M + H]+ Calcd for $C_{16}H_{24}NO_3$ 278.1751; Found: 278.1754.

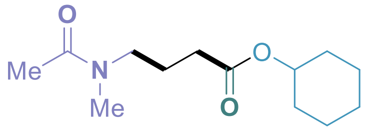

cyclohexyl 4-(*N*-methylacetamido)butanoate (4x)

This reaction was conducted on a 0.3 mmol scale with the general procedure II. The crude product was purified by silica gel chromatography (PE/EA = 1:1) to afford the title compound as a colorless liquid (60.0 mg, 83% yield). The identity of the product was confirmed by ^{1}H NMR, ^{13}C NMR, and HRMS. Analysis of NMR spectra revealed the presence of two amide bond rotamers. These conformational isomers have been observed and studied in similar compounds.[1] The minor distinguishable rotamer peak is denoted with *.

^{1}H NMR (400 MHz, CDCl$_3$) δ 5.03 – 4.56 (m, 1H), 3.42 – 3.30/3.30* – 3.21* (m, 2H), 2.94/2.86* (s, 3H), 2.31 – 2.13 (m, 2H), 2.04*/2.01 (s, 3H), 1.86 – 1.73 (m, 4H), 1.73 – 1.59 (m, 2H), 1.56 – 1.43 (m, 1H), 1.38 – 1.20 (m, 5H).
^{13}C NMR (101 MHz, CDCl$_3$) δ 172.5/172.0*, 170.5/170.4*, 72.9*/72.5, 49.7*/46.6, 36.0/33.0*, 31.7*/31.5, 31.5/31.0*, 25.2/25.2*, 23.6*/23.6, 23.3*/22.5, 21.7/21.0*.
HRMS (ESI-TOF) m/z: [M + H]+ Calcd for $C_{13}H_{24}NO_3$ 242.1751; Found: 242.1751.

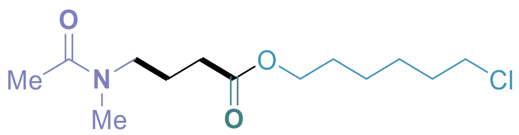

6-chlorohexyl 4-(*N*-methylacetamido)butanoate (4y)

This reaction was conducted on a 0.3 mmol scale with the general procedure II. The crude product was purified by silica gel chromatography (PE/EA = 1:1) to afford the title compound as a colorless liquid (64.0 mg, 77% yield). The identity of the product was confirmed by ^{1}H NMR, ^{13}C NMR, and HRMS. Analysis of NMR spectra revealed the presence of two amide bond rotamers. These conformational isomers have been observed and studied in similar compounds.[1] The minor distinguishable rotamer peak is denoted with *.

^{1}H NMR (400 MHz, CDCl$_3$) δ 4.18 – 3.92 (m, 2H), 3.49 (t, J = 6.7 Hz, 2H), 3.44 – 3.32/3.31* – 3.19* (m, 2H), 2.95/2.87* (s, 3H), 2.28 (q, J = 7.4 Hz, 2H), 2.05*/2.02 (s, 3H), 1.94 – 1.67 (m, 4H), 1.65 – 1.50 (m, 2H), 1.49 – 1.38 (m, 2H), 1.37 – 1.26 (m, 2H).
^{13}C NMR (101 MHz, CDCl$_3$) δ 173.1/172.6*, 170.5/170.4*, 64.5*/64.2, 49.7*/46.6, 44.8/44.8*, 36.0/33.0*, 32.3/32.3*, 31.3/30.7*, 28.3/28.3*, 28.0*/25.1, 26.4/26.3*, 23.2*/22.4, 21.7/21.1*.
HRMS (ESI-TOF) m/z: [M + H]+ Calcd for $C_{13}H_{25}NO_3Cl$ 278.1514; Found: 278.1522.

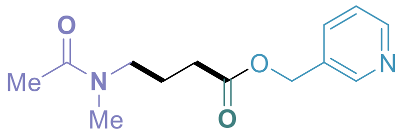

pyridin-3-ylmethyl 4-(*N*-methylacetamido)butanoate (4z)

This reaction was conducted on a 0.3 mmol scale with the general procedure II. The crude product was purified by silica gel chromatography (EA/MeOH = 10:1) to afford the title compound as a colorless liquid (52.5 mg, 70% yield). The identity of the product was confirmed by ^{1}H NMR, ^{13}C NMR, and HRMS. Analysis of NMR spectra revealed the presence of two amide bond rotamers. These conformational isomers have

been observed and studied in similar compounds.[1] The minor distinguishable rotamer peak is denoted with *.

^{1}H NMR (400 MHz, $CDCl_3$) δ 8.90 – 8.35 (m, 2H), 7.72 (dd, J = 7.9, 2.1 Hz, 1H), 7.50 – 7.04 (m, 1H), 5.16*/5.14 (s, 2H), 3.82 – 3.35/3.35* – 3.22* (m, 2H), 2.98/2.90* (s, 3H), 2.56 – 2.25 (m, 2H), 2.06 (s, 3H), 2.00 – 1.74 (m, 2H).
^{13}C NMR (101 MHz, $CDCl_3$) δ 172.6/172.1*, 170.6/170.3*, 149.5, 149.3/149.3*, 136.1*/136.0, 131.5*/131.2' 123.4*/123.4, 63.7*/63.5, 49.6*/46.4, 35.9/32.9*, 31.1/30.5*, 23.1*/22.2, 21.6/20.9*.
HRMS (ESI-TOF) m/z: [M + H]+ Calcd for $C_{13}H_{19}N_2O_3$ 251.1390; Found: 250.1391.

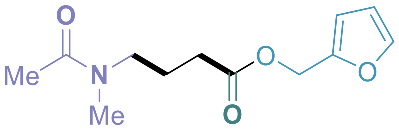

furan-2-ylmethyl 4-(*N*-methylacetamido)butanoate (4aa)

This reaction was conducted on a 0.3 mmol scale with the general procedure II. The crude product was purified by silica gel chromatography (PE/EA = 1:2) to afford the title compound as a colorless liquid (46.6 mg, 65% yield). The identity of the product was confirmed by ^{1}H NMR, ^{13}C NMR, and HRMS. Analysis of NMR spectra revealed the presence of two amide bond rotamers. These conformational isomers have been observed and studied in similar compounds.[1] The minor distinguishable rotamer peak is denoted with *.

^{1}H NMR (400 MHz, $CDCl_3$) δ 7.47 – 7.32 (m, 1H), 6.37 (dd, J = 6.0, 3.3 Hz, 1H), 6.36 – 6.08 (m, 1H), 5.05/5.03* (s, 2H), 3.52 – 3.31/3.31* – 3.19* (m, 2H), 2.93/2.86* (s, 3H), 2.56 – 2.25 (m, 2H), 2.02 (s, 3H), 1.97 – 1.75 (m, 2H).
^{13}C NMR (101 MHz, $CDCl_3$) δ 172.7/172.1*, 170.5/170.4*, 149.4/149.1*, 143.3*/143.1, 110.8, 110.5/110.5*, 58.1*/57.9, 49.7*/46.5, 36.0/33.0*, 31.2/30.6*, 23.2*/22.3, 21.7/21.0*.
HRMS (ESI-TOF) m/z: [M + H]+ Calcd for $C_{12}H_{18}NO_4$ 240.1230; Found: 240.1230.

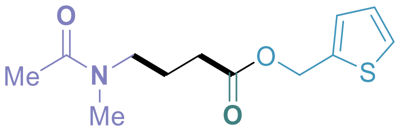

thiophen-2-ylmethyl 4-(*N*-methylacetamido)butanoate (4ab)

This reaction was conducted on a 0.3 mmol scale with the general procedure II. The crude product was purified by silica gel chromatography (PE/EA = 1:2) to afford the title compound as a colorless liquid (50.5 mg, 66% yield). The identity of the product

was confirmed by ^{1}H NMR, ^{13}C NMR, and HRMS. Analysis of NMR spectra revealed the presence of two amide bond rotamers. These conformational isomers have been observed and studied in similar compounds.[1] The minor distinguishable rotamer peak is denoted with *.

^{1}H NMR (400 MHz, $CDCl_3$) δ 7.43 – 7.29 (m, 1H), 7.14 – 7.02 (m, 1H), 6.98 (d, J = 5.1 Hz, 1H), 5.29/5.27* (s, 2H), 3.47 – 3.34/3.34* – 3.18* (m, 2H), 2.96/2.89* (s, 3H), 2.44 – 2.29 (m, 2H), 2.05/2.04* (s, 3H), 1.96 – 1.81 (m, 2H).

^{13}C NMR (101 MHz, $CDCl_3$) δ 172.8/172.2*, 170.6/170.5*, 137.8/137.5*, 128.4*/128.1, 127.0, 126.8*/126.8, 60.5*/60.4, 49.7*/46.6, 36.0/33.1*, 31.3/30.8*, 23.2*/22.4, 21.8/21.0*.

HRMS (ESI-TOF) m/z: [M + H]+ Calcd for $C_{12}H_{18}NO_3S$ 256.1002; Found: 256.1006.

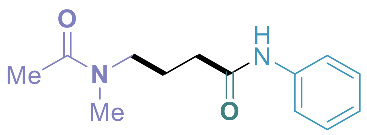

4-(*N*-methylacetamido)-*N*-phenylbutanamide (5a)

This reaction was conducted on a 0.3 mmol scale with the general procedure II. The crude product was purified by silica gel chromatography (EA/MeOH = 20:1) to afford the title compound as a colorless oil (54.0 mg, 77% yield). The identity of the product was confirmed by ^{1}H NMR, ^{13}C NMR, and HRMS. Analysis of NMR spectra revealed the presence of two amide bond rotamers. These conformational isomers have been observed and studied in similar compounds.[1] The minor distinguishable rotamer peak is denoted with *.

^{1}H NMR (400 MHz, $CDCl_3$) δ 9.32/8.41* (s, 1H), 7.64/7.53* (d, J = 7.6 Hz, 2H), 7.29* (t, J = 7.3 Hz, 2H), 7.10 – 7.04 (m, 1H), 3.50/3.37* (t, J = 7.2 Hz, 2H), 3.00/2.91* (s, 3H), 2.38*/2.31 (t, J = 7.0 Hz, 2H), 2.11/2.09* (s, 3H), 1.99 – 1.89 (m, 2H).

^{13}C NMR (101 MHz, $CDCl_3$) δ 172.0*/171.2, 170.8/170.2*, 138.6/138.1*, 128.9*/128.7, 124.1*/123.7, 119.8*/119.6, 49.8*/46.4, 36.0/33.1*, 34.5/33.0*, 23.6/23.4*, 21.9/21.2*.

HRMS (ESI-TOF) m/z: [M + H]+ Calcd for $C_{13}H_{19}N_2O_2$ 235.1441; Found: 235.1442.

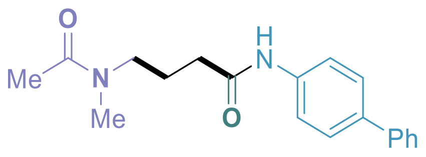

***N*-([1,1'-biphenyl]-4-yl)-4-(*N*-methylacetamido)butanamide (5b)**

This reaction was conducted on a 0.3 mmol scale with the general procedure II. The crude product was purified by silica gel chromatography (EA/MeOH = 20:1) to afford

the title compound as a colorless oil (77.2 mg, 83% yield). The identity of the product was confirmed by ^{1}H NMR, ^{13}C NMR, and HRMS. Analysis of NMR spectra revealed the presence of two amide bond rotamers. These conformational isomers have been observed and studied in similar compounds.[1] The minor distinguishable rotamer peak is denoted with *.

^{1}H NMR (400 MHz, CDCl$_3$) δ 9.44/8.30* (s, 1H), 7.73 (d, J = 8.6 Hz, 2H), 7.55 (t, J = 8.7 Hz, 4H), 7.41 (t, J = 7.7 Hz, 2H), 7.31 (d, J = 7.4 Hz, 1H), 3.52/3.40* (t, J = 7.2 Hz, 2H), 3.02/2.93* (s, 3H), 2.41*/2.33 (t, J = 7.0 Hz, 2H), 2.12/2.11* (s, 3H), 2.02 – 1.91 (m, 2H).

^{13}C NMR (101 MHz, CDCl$_3$) δ 172.1, 171.2, 140.7, 138.0, 136.5, 128.7*/128.7, 127.5*/127.4, 126.9*/126.7, 120.1*/119.9, 49.8*/46.4, 36.1/33.2*, 34.5/33.1*, 23.7/23.7*, 21.9/21.2*.

HRMS (ESI-TOF) m/z: [M + H]+ Calcd for $C_{19}H_{23}N_2O_2$ 311.1754; Found: 311.1753.

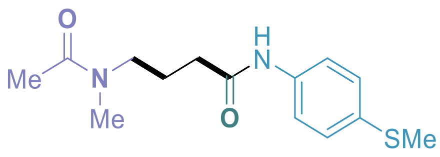

4-(*N*-methylacetamido)-*N*-(4-(methylthio)phenyl)butanamide (5c)

This reaction was conducted on a 0.3 mmol scale with the general procedure II. The crude product was purified by silica gel chromatography (EA/MeOH = 20:1) to afford the title compound as a colorless oil (68.0 mg, 81% yield). The identity of the product was confirmed by ^{1}H NMR, ^{13}C NMR, and HRMS. Analysis of NMR spectra revealed the presence of two amide bond rotamers. These conformational isomers have been observed and studied in similar compounds.[1] The minor distinguishable rotamer peak is denoted with *.

^{1}H NMR (400 MHz, CDCl$_3$) δ 9.38/8.27* (s, 1H), 7.59/7.46* (d, J = 8.7 Hz, 2H), 7.34 – 7.12 (m, 2H), 3.49/3.38* (t, J = 7.3 Hz, 2H), 3.01/2.92* (s, 3H), 2.45 (s, 3H), 2.37*/2.30 (t, J = 7.0 Hz, 2H), 2.11/2.09* (s, 3H), 2.04 – 1.85 (m, 2H).

^{13}C NMR (101 MHz, CDCl$_3$) δ 172.0, 171.9, 136.4, 132.6, 128.1*/127.9, 120.4/120.2*, 49.7*/46.4, 36.1, 34.4/33.0*, 23.6/23.4*, 21.9/21.2*, 16.8.

HRMS (ESI-TOF) m/z: [M + H]+ Calcd for $C_{14}H_{21}N_2O_2S$ 281.1318; Found: 281.1324.

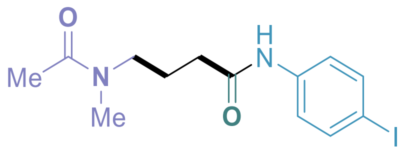

***N*-(4-iodophenyl)-4-(*N*-methylacetamido)butanamide (5d)**

This reaction was conducted on a 0.3 mmol scale with the general procedure II. The crude product was purified by silica gel chromatography (EA/MeOH = 20:1) to afford the title compound as a colorless oil (76.7 mg, 71% yield). The identity of the product was confirmed by ^{1}H NMR, ^{13}C NMR, and HRMS. Analysis of NMR spectra revealed the presence of two amide bond rotamers. These conformational isomers have been observed and studied in similar compounds.[1] The minor distinguishable rotamer peak is denoted with *.

^{1}H NMR (400 MHz, $CDCl_3$) δ 9.62/8.77* (s, 1H), 7.58 (d, J = 8.8 Hz, 2H), 7.45/7.34* (d, J = 8.4 Hz, 2H), 3.48/3.38 (t, J = 7.1 Hz, 2H), 3.01/2.91* (s, 3H), 2.37*/2.29 (t, J = 7.1 Hz, 2H), 2.11/2.09* (s, 3H), 2.03 – 1.85 (m, 2H).

^{13}C NMR (101 MHz, $CDCl_3$) δ 172.1, 171.3, 138.5/138.0*, 137.7*/137.6, 121.6*/121.5, 86.6, 49.7*/46.4, 36.1, 34.4/33.0*, 23.5/23.3*, 21.8/21.2*.

HRMS (ESI-TOF) m/z: [M + H]+ Calcd for $C_{13}H_{18}N_2O_2I$ 361.0401; Found: 361.0407.

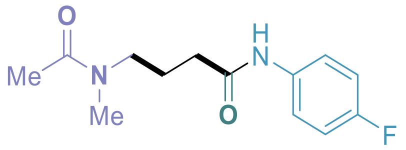

***N*-(4-fluorophenyl)-4-(*N*-methylacetamido)butanamide (5e)**

This reaction was conducted on a 0.3 mmol scale with the general procedure II. The crude product was purified by silica gel chromatography (EA/MeOH = 20:1) to afford the title compound as a colorless oil (52.9 mg, 70% yield). The identity of the product was confirmed by ^{1}H NMR, ^{13}C NMR, and HRMS. Analysis of NMR spectra revealed the presence of two amide bond rotamers. These conformational isomers have been observed and studied in similar compounds.[1] The minor distinguishable rotamer peak is denoted with *.

^{1}H NMR (400 MHz, $CDCl_3$) δ 9.52/8.72* (s, 1H), 7.58/7.47* (dd, J = 9.1, 4.8 Hz, 2H), 6.94 (t, J = 8.7 Hz, 2H), 3.46/3.35* (t, J = 7.2 Hz, 2H), 2.98/2.88* (s, 3H), 2.34*/2.27 (t, J = 7.1 Hz, 2H), 2.08/2.06* (s, 3H), 2.04 – 1.81 (m, 2H).

^{13}C NMR (101 MHz, CDCl$_3$) δ 172.0/170.8*, 171.1/170.2*, 158.9 (d, J = 242.5 Hz), 134.7 (d, J = 2.8 Hz), 121.5*/121.3 (d, J = 7.8 Hz), 115.4*/115.2 (d, J = 22.4 Hz), 49.7*/46.4, 36.0/33.0*, 34.2/32.8*, 23.5/23.4*, 21.8/21.2*.
HRMS (ESI-TOF) m/z: [M + H]+ Calcd for $C_{13}H_{18}N_2O_2F$ 253.1347; Found: 253.1342.

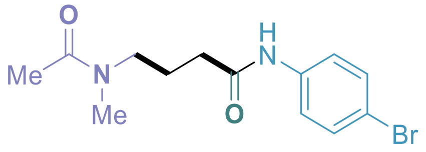

***N*-(4-bromophenyl)-4-(*N*-methylacetamido)butanamide (5f)**

This reaction was conducted on a 0.3 mmol scale with the general procedure II. The crude product was purified by silica gel chromatography (EA/MeOH = 20:1) to afford the title compound as a colorless oil (82.4 mg, 88% yield). The identity of the product was confirmed by ^{1}H NMR, ^{13}C NMR, and HRMS. Analysis of NMR spectra revealed the presence of two amide bond rotamers. These conformational isomers have been observed and studied in similar compounds.[1] The minor distinguishable rotamer peak is denoted with *.

^{1}H NMR (400 MHz, CDCl$_3$) δ 9.65/8.78* (s, 1H), 7.57 (d, J = 8.8 Hz, 2H), 7.39 (d, J = 8.8 Hz, 2H), 3.48/3.38* (t, J = 6.2 Hz, 2H), 3.01/2.91* (s, 3H), 2.38/2.29* (t, J = 7.1 Hz, 2H), 2.12/2.09* (s, 3H), 1.99 – 1.81 (m, 2H).
^{13}C NMR (101 MHz, CDCl$_3$) δ 172.1/170.8*, 171.3/170.3*, 137.8/137.3*, 131.7*/131.6, 121.3*/121.2, 116.4*/116.0, 49.7*/46.4, 36.1/33.0*, 34.4/33.0*, 23.6/23.3*, 21.8/21.2*.
HRMS (ESI-TOF) m/z: [M + H]+ Calcd for $C_{13}H_{18}N_2O_2Br$ 313.0546; Found: 313.0549.

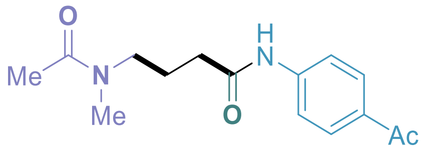

***N*-(4-acetylphenyl)-4-(*N*-methylacetamido)butanamide (5g)**

This reaction was conducted on a 0.3 mmol scale with the general procedure II. The crude product was purified by silica gel chromatography (EA/MeOH = 20:1) to afford the title compound as a colorless oil (53.8 mg, 65% yield). The identity of the product was confirmed by ^{1}H NMR, ^{13}C NMR, and HRMS. Analysis of NMR spectra revealed the presence of two amide bond rotamers. These conformational isomers have been observed and studied in similar compounds.[1] The minor distinguishable rotamer peak is denoted with *.

^{1}H NMR (400 MHz, CDCl$_3$) δ 9.93/9.35* (s, 1H), 7.91 (d, J = 8.8 Hz, 2H), 7.77/7.69* (d, J = 8.7 Hz, 2H), 3.50/3.45* (t, J = 6.3 Hz, 2H), 3.03/2.93* (s, 3H), 2.57 (s, 3H), 2.46/2.36* (t, J = 7.0 Hz, 2H), 2.12/2.09* (s, 3H), 2.02 – 1.85 (m, 2H).
^{13}C NMR (101 MHz, CDCl$_3$) δ 197.1/197.0*, 175.2*/172.1, 174.8*/171.7, 143.2/142.9*, 132.3*/132.2, 129.5*/129.5, 118.8, 49.7*/46.4, 36.0/36.0*, 34.4/33.0*, 26.3, 23.4/23.2*, 21.7/21.7*.
HRMS (ESI-TOF) m/z: [M + H]+ Calcd for $C_{15}H_{21}N_2O_3$ 277.1547; Found: 277.1554.

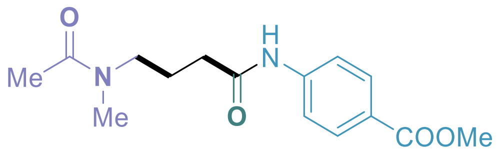

methyl 4-(4-(*N*-methylacetamido)butanamido)benzoate (5h)

This reaction was conducted on a 0.3 mmol scale with the general procedure II. The crude product was purified by silica gel chromatography (EA/MeOH = 20:1) to afford the title compound as a colorless oil (68.3 mg, 78% yield). The identity of the product was confirmed by ^{1}H NMR, ^{13}C NMR, and HRMS. Analysis of NMR spectra revealed the presence of two amide bond rotamers. These conformational isomers have been observed and studied in similar compounds.[1] The minor distinguishable rotamer peak is denoted with *.

^{1}H NMR (700 MHz, CDCl$_3$) δ 9.89/9.08* (s, 1H), 7.98 (d, J = 8.7 Hz, 2H), 7.75/7.66* (d, J = 8.4 Hz, 2H), 3.89 (s, 3H), 3.50/3.39* (t, J = 6.2 Hz, 2H), 3.02/2.92* (s, 3H), 2.43*/2.34 (t, J = 7.1 Hz, 2H), 2.12/2.10* (s, 3H), 2.00 – 1.86 (m, 2H).
^{13}C NMR (176 MHz, CDCl$_3$) δ 172.1/170.8*, 171.6/170.7*, 166.7/166.6*, 142.9/142.6*, 130.6*/130.6, 125.1*/124.8, 118.7, 51.9*/51.8, 49.7*/46.4, 36.0/33.1*, 34.5/33.0*, 23.5/23.2*, 21.8/21.1*.
HRMS (ESI-TOF) m/z: [M + H]+ Calcd for $C_{15}H_{21}N_2O_4$ 293.1496; Found: 193.1498.

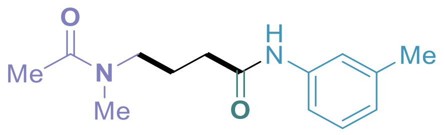

4-(*N*-methylacetamido)-*N*-(*m*-tolyl)butanamide (5i)

This reaction was conducted on a 0.3 mmol scale with the general procedure II. The crude product was purified by silica gel chromatography (EA/MeOH = 20:1) to afford the title compound as a colorless oil (52.1 mg, 70% yield). The identity of the product was confirmed by ^{1}H NMR, ^{13}C NMR, and HRMS. Analysis of NMR spectra revealed the presence of two amide bond rotamers. These conformational isomers have been

observed and studied in similar compounds.[1] The minor distinguishable rotamer peak is denoted with *.

^{1}H NMR (400 MHz, $CDCl_3$) δ 9.23/8.45* (s, 1H), 7.52 – 7.26 (m, 2H), 7.17 (t, J = 7.8 Hz, 1H), 6.88 (d, J = 7.6 Hz, 1H), 3.49/3.36* (t, J = 6.3 Hz, 2H), 3.00/2.91* (s, 3H), 2.47 – 2.24 (m, 5H), 2.10/2.09* (s, 3H), 2.02 – 1.87 (m, 2H).

^{13}C NMR (101 MHz, $CDCl_3$) δ 171.9*/171.2, 170.8*/170.2, 138.7*/138.5, 138.5/138.0*, 128.6*/128.5, 124.8*/124.5, 120.4*/120.2, 116.9*/116.8, 49.8*/46.4, 36.0/33.1*, 34.4/33.0*, 23.6/23.4*, 21.8, 21.4/21.2*.

HRMS (ESI-TOF) m/z: [M + H]+ Calcd for $C_{14}H_{21}N_2O_2$ 249.1598; Found: 249.1596.

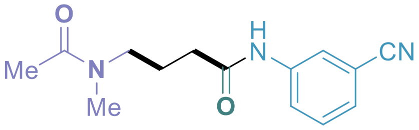

N-(3-cyanophenyl)-4-(N-methylacetamido)butanamide (5j)

This reaction was conducted on a 0.3 mmol scale with the general procedure II. The crude product was purified by silica gel chromatography (EA/MeOH = 20:1) to afford the title compound as a colorless oil (68.4 mg, 88% yield). The identity of the product was confirmed by ^{1}H NMR, ^{13}C NMR, and HRMS. Analysis of NMR spectra revealed the presence of two amide bond rotamers. These conformational isomers have been observed and studied in similar compounds.[1] The minor distinguishable rotamer peak is denoted with *.

^{1}H NMR (400 MHz, DMSO-d_6) δ 10.29*/10.26 (s, 1H), 8.11 (s, 1H), 7.79 (d, J = 7.7 Hz, 1H), 7.61 – 7.45 (m, 2H), 3.39 – 3.21 (m, 2H), 2.95/2.80* (s, 3H), 2.39/2.31* (t, J = 7.2 Hz, 2H), 2.01*/1.97 (s, 3H), 1.94 – 1.71 (m, 2H).

^{13}C NMR (101 MHz, DMSO-d_6) δ 171.4/171.2*, 169.7/169.3*, 140.1*/139.9, 130.2*/130.2, 126.6*/126.5, 123.6*/123.5, 121.7/121.6*, 118.7*/118.7, 111.5*/111.5, 49.1*/45.8, 35.5/33.6*, 32.9*/32.4, 23.2*/22.5, 21.7/21.0*.

HRMS (ESI-TOF) m/z: [M + H]+ Calcd for $C_{14}H_{18}N_3O_2$ 260.1394; Found: 260.1386.

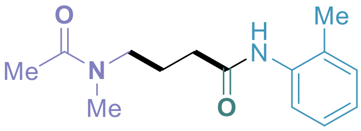

4-(N-methylacetamido)-N-(o-tolyl)butanamide (5k)

This reaction was conducted on a 0.3 mmol scale with the general procedure II. The crude product was purified by silica gel chromatography (EA/MeOH = 20:1) to afford the title compound as a colorless oil (46.1 mg, 62% yield). The identity of the product

was confirmed by ^{1}H NMR, ^{13}C NMR, and HRMS. Analysis of NMR spectra revealed the presence of two amide bond rotamers. These conformational isomers have been observed and studied in similar compounds.[1] The minor distinguishable rotamer peak is denoted with *.

^{1}H NMR (400 MHz, CDCl$_3$) δ 8.49/7.97* (s, 1H), 7.64/7.54* (d, J = 7.7 Hz, 1H), 7.24 – 6.94 (m, 3H), 3.48/3.34* (t, J = 6.5 Hz, 2H), 2.98/2.89* (s, 3H), 2.34 (t, J = 6.7 Hz, 2H), 2.27/2.21* (s, 3H), 2.07*/2.05 (s, 3H), 2.01 – 1.72 (m, 2H).
^{13}C NMR (101 MHz, CDCl$_3$) δ 171.4/170.5*, 171.2/170.4*, 135.8/135.4*, 130.6*/130.4, 130.4*/130.3, 126.3*/126.1, 125.4*/125.1, 124.2*/123.9, 49.8*/46.4, 35.9/32.9*, 33.9/32.6*, 23.5*/23.4, 21.7/21.1*, 17.9/17.7*.
HRMS (ESI-TOF) m/z: [M + H]+ Calcd for $C_{14}H_{21}N_2O_2$ 249.1598; Found: 249.1596.

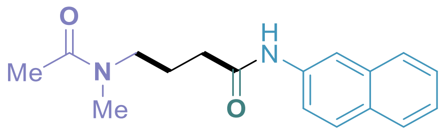

4-(*N*-methylacetamido)-*N*-(naphthalen-2-yl)butanamide (5l)

This reaction was conducted on a 0.3 mmol scale with the general procedure II. The crude product was purified by silica gel chromatography (EA/MeOH = 20:1) to afford the title compound as a colorless oil (66.5 mg, 78% yield). The identity of the product was confirmed by ^{1}H NMR, ^{13}C NMR, and HRMS. Analysis of NMR spectra revealed the presence of two amide bond rotamers. These conformational isomers have been observed and studied in similar compounds.[1] The minor distinguishable rotamer peak is denoted with *.

^{1}H NMR (400 MHz, CDCl$_3$) δ 9.53/8.40* (s, 1H), 8.34/8.23* (s, 1H), 7.84 – 7.69 (m, 3H), 7.61 (dd, J = 8.8, 2.1 Hz, 1H), 7.57 – 7.32 (m, 2H), 3.53/3.37* (t, J = 6.1 Hz, 2H), 3.00/2.92* (s, 3H), 2.41*/2.36 (t, J = 6.9 Hz, 2H), 2.12/2.09* (s, 3H), 2.06 – 1.87 (m,2H).
^{13}C NMR (101 MHz, CDCl$_3$) δ 172.1, 171.4, 136.1, 133.9, 130.4, 128.5, 127.6/127.6*, 127.4, 126.4*/126.2, 124.6, 120.0, 116.8, 116.1, 49.7*/46.4, 36.1, 34.6/33.0*, 23.7, 21.9.
HRMS (ESI-TOF) m/z: [M + H]+ Calcd for $C_{17}H_{21}N_2O_2$ 285.1598; Found: 285.1601.

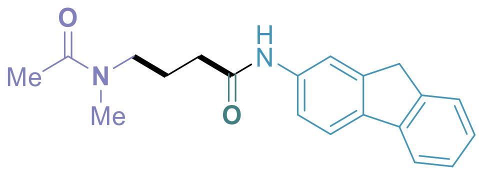

***N*-(9*H*-fluoren-2-yl)-4-(*N*-methylacetamido)butanamide (5m)**

This reaction was conducted on a 0.3 mmol scale with the general procedure II. The crude product was purified by silica gel chromatography (EA/MeOH = 20:1) to afford the title compound as a colorless oil (78.2 mg, 81% yield). The identity of the product was confirmed by ^{1}H NMR, ^{13}C NMR, and HRMS. Analysis of NMR spectra revealed the presence of two amide bond rotamers. These conformational isomers have been observed and studied in similar compounds.[1] The minor distinguishable rotamer peak is denoted with *.

^{1}H NMR (400 MHz, CDCl$_3$) δ 9.42/8.16* (s, 1H), 7.96/7.89* (s, 1H), 7.76 – 7.65 (m, 2H), 7.58 (d, J = 8.2 Hz, 1H), 7.50 (d, J = 7.4 Hz, 1H), 7.34 (t, J = 7.5 Hz, 1H), 7.28 – 7.19 (m, 1H), 3.86/3.78* (s, 2H), 3.53/3.39 (t, J = 6.1 Hz, 2H), 3.01/2.94* (s, 3H), 2.40*/2.33 (t, J = 6.9 Hz, 2H), 2.13/2.08* (s, 3H), 2.05 – 1.85 (m, 2H).
^{13}C NMR (101 MHz, CDCl$_3$) δ 172.2/170.1*, 171.2/169.3*, 144.1, 143.1/143.1*, 141.5, 137.6, 137.4, 126.6, 126.2*/126.0, 124.9*/124.9, 120.0*/120.0, 119.4*/119.4, 118.6*/118.5, 116.8*/116.5, 49.8*/46.4, 38.0*/37.0, 36.1/35.2*, 34.6/33.1*, 23.8/23.5*, 21.9/21.5*.
HRMS (ESI-TOF) m/z: [M + H]+ Calcd for $C_{20}H_{23}N_2O_2$ 323.1754; Found: 323.1757.

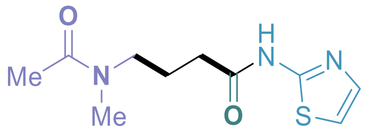

4-(*N*-methylacetamido)-*N*-(thiazol-2-yl)butanamide (5n)

This reaction was conducted on a 0.3 mmol scale with the general procedure II. The crude product was purified by silica gel chromatography (EA/MeOH = 10:1) to afford the title compound as a colorless oil (56.4 mg, 78% yield). The identity of the product was confirmed by ^{1}H NMR, ^{13}C NMR, and HRMS. Analysis of NMR spectra revealed the presence of two amide bond rotamers. These conformational isomers have been observed and studied in similar compounds.[1] The minor distinguishable rotamer peak is denoted with *.

^{1}H NMR (400 MHz, DMSO-d$_6$) δ 12.10/12.05* (s, 1H), 7.45 (t, J = 3.2 Hz, 1H), 7.28 – 7.12 (m, 1H), 3.30 (t, J = 7.3 Hz, 2H), 2.94/2.78* (s, 3H), 2.46*/2.39 (t, J = 7.4 Hz, 2H), 1.99*/1.95 (s, 3H), 1.90 – 1.67 (m, 2H).
^{13}C NMR (101 MHz, DMSO-d$_6$) δ 170.8/170.6*, 169.7/169.3*, 158.0/57.9*, 137.5*/137.5, 113.2*/113.2, 49.1*/45.8, 35.5/32.4*, 32.2/31.7*, 23.1*/22.4, 21.7/21.0*.
HRMS (ESI-TOF) m/z: [M + H]+ Calcd for $C_{10}H_{16}N_3O_2S$ 242.0958; Found: 242.0966.

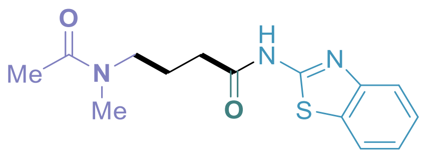

***N*-(benzo[d]thiazol-2-yl)-4-(*N*-methylacetamido)butanamide (5o)**

This reaction was conducted on a 0.3 mmol scale with the general procedure II. The crude product was purified by silica gel chromatography (EA/MeOH = 10:1) to afford the title compound as a colorless oil (70.7 mg, 81% yield). The identity of the product was confirmed by ^{1}H NMR, ^{13}C NMR, and HRMS. Analysis of NMR spectra revealed the presence of two amide bond rotamers. These conformational isomers have been observed and studied in similar compounds.[1] The minor distinguishable rotamer peak is denoted with *.

^{1}H NMR (400 MHz, DMSO-d$_6$) δ12.38*/12.32 (s, 1H), 7.97 (d, J = 8.0 Hz, 1H), 7.74 (d, J = 8.2 Hz, 1H), 7.50 – 7.40 (m, 1H), 7.30 (t, J = 7.4 Hz, 1H), 3.44 – 3.20 (m, 2H), 2.95/2.81* (s, 3H), 2.66 – 2.39 (m, 2H), 2.01*/1.97 (s, 3H), 1.93 – 1.71 (m, 2H).
^{13}C NMR (101 MHz, DMSO-d$_6$) δ 171.9/171.7*, 169.7/169.3*, 157.9/157.9*, 148.6/148.5*, 131.4, 126.0*/126.0, 123.4*/123.4, 121.6*/121.6, 120.5*/120.4, 49.1*/45.8, 35.5/32.5*, 32.4/32.0*, 22.9*/22.2, 21.7/21.0*.
HRMS (ESI-TOF) m/z: [M + H]+ Calcd for $C_{14}H_{18}N_3O_2S$ 292.1114; Found: 292.1111.

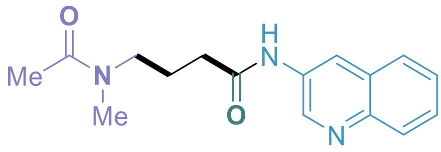

4-(*N*-methylacetamido)-*N*-(quinolin-3-yl)butanamide (5p)

This reaction was conducted on a 0.3 mmol scale with the general procedure II. The crude product was purified by silica gel chromatography (EA/MeOH = 10:1) to afford the title compound as a colorless oil (52.2 mg, 61% yield). The identity of the product was confirmed by ^{1}H NMR, ^{13}C NMR, and HRMS. Analysis of NMR spectra revealed the presence of two amide bond rotamers. These conformational isomers have been

observed and studied in similar compounds.[1] The minor distinguishable rotamer peak is denoted with *.

^{1}H NMR (400 MHz, CDCl$_3$) δ 10.29/10.05* (s, 1H), 8.94 (s, 1H), 8.78 (d, J = 22.0 Hz, 1H), 7.96 (t, J = 9.2 Hz, 1H), 7.73 (d, J = 8.2 Hz, 1H), 7.54 (t, J = 7.7 Hz, 1H), 7.45 (t, J = 7.4 Hz, 1H), 3.47/3.35* (t, J = 6.5 Hz, 2H), 2.97/2.88* (s, 3H), 2.46*/2.40 (t, J = 6.8 Hz, 2H), 2.07/2.06* (s, 3H), 2.03 – 1.86 (m, 2H).

^{13}C NMR (101 MHz, CDCl$_3$) δ 172.0/171.3*, 171.8/170.8*, 144.7, 144.4/144.0*, 132.5/132.4*, 128.6/128.5*, 128.3/128.0*, 127.8/127.7*, 127.6, 127.1*/126.9, 123.6*/123.3, 49.7*/46.6, 36.0/34.3*, 33.0/32.9*, 23.4/23.2*, 21.8/21.2*.

HRMS (ESI-TOF) m/z: [M + H]+ Calcd for $C_{16}H_{20}N_3O_2$ 286.1550; Found: 286.1559.

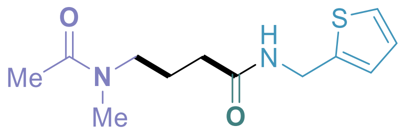

4-(*N*-methylacetamido)-*N*-(thiophen-2-ylmethyl)butanamide (5q)

This reaction was conducted on a 0.3 mmol scale with the general procedure II. The crude product was purified by silica gel chromatography (EA/MeOH = 10:1) to afford the title compound as a colorless oil (44.2 mg, 58% yield). The identity of the product was confirmed by ^{1}H NMR, ^{13}C NMR, and HRMS. Analysis of NMR spectra revealed the presence of two amide bond rotamers. These conformational isomers have been observed and studied in similar compounds.[1] The minor distinguishable rotamer peak is denoted with *.

^{1}H NMR (700 MHz, CDCl$_3$) δ 7.35 (s, 1H), 7.20*/7.18 (d, J = 5.1 Hz, 1H), 7.00 – 6.89 (m, 2H), 4.59/4.58* (s, 2H), 3.39/3.32* (t, J = 6.7 Hz, 2H), 2.98/2.88* (s, 3H), 2.22/2.18* (t, J = 7.0 Hz, 2H), 2.05*/2.04 (s, 3H), 1.97 – 1.82 (m, 2H).

^{13}C NMR (101 MHz, CDCl$_3$) δ 172.4/171.5*, 171.4/170.8*, 141.3/141.0*, 126.7*/126.7, 125.9*/125.6, 125.0*/124.7, 49.8*/46.5, 38.1/38.1*, 36.0/33.1*, 33.0/32.2*, 23.6*/23.2, 21.7/21.0*.

HRMS (ESI-TOF) m/z: [M + H]+ Calcd for $C_{12}H_{19}N_2O_2S$ 255.1162; Found: 255.1159.

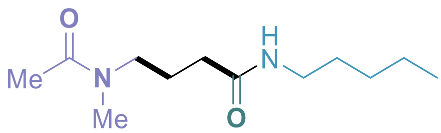

4-(*N*-methylacetamido)-*N*-pentylbutanamide (5r)

This reaction was conducted on a 0.3 mmol scale with the general procedure II. The crude product was purified by silica gel chromatography (EA/MeOH = 20:1) to afford

the title compound as a colorless oil (56.8 mg, 83% yield). The identity of the product was confirmed by ^{1}H NMR, ^{13}C NMR, and HRMS. Analysis of NMR spectra revealed the presence of two amide bond rotamers. These conformational isomers have been observed and studied in similar compounds.[1] The minor distinguishable rotamer peak is denoted with *.

^{1}H NMR (400 MHz, CDCl$_3$) δ 6.81/6.00* (s, 1H), 3.42/3.35* (t, J = 6.6 Hz, 2H), 3.31 – 3.16 (m, 2H), 3.00/2.91* (s, 3H), 2.41 – 2.03 (m, 5H), 1.95 – 1.76 (m, 2H), 1.66 – 1.43 (m, 2H), 1.39 – 1.19 (m, 4H), 1.05 – 0.79 (m, 3H).

^{13}C NMR (101 MHz, CDCl$_3$) δ 172.5/171.4*, 171.6/170.7*, 49.9, 46.7*/46.5, 39.5/39.5*, 36.1*/36.0, 33.3/33.1*, 32.4/31.3*, 29.2*/29.1, 29.0/29.0*, 23.7*/23.3, 22.2/22.2*, 21.8/21.7*, 21.1/21.0*, 13.9/13.9*.

HRMS (ESI-TOF) m/z: [M + H]+ Calcd for $C_{12}H_{25}N_2O_2$ 229.1911; Found: 229.1916

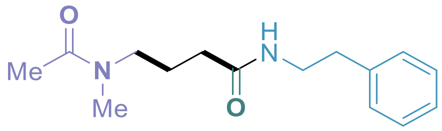

4-(*N*-methylacetamido)-*N*-phenethylbutanamide (5s)

This reaction was conducted on a 0.3 mmol scale with the general procedure II. The crude product was purified by silica gel chromatography (EA/MeOH = 20:1) to afford the title compound as a colorless oil (55.8 mg, 71% yield). The identity of the product was confirmed by ^{1}H NMR, ^{13}C NMR, and HRMS. Analysis of NMR spectra revealed the presence of two amide bond rotamers. These conformational isomers have been observed and studied in similar compounds.[1] The minor distinguishable rotamer peak is denoted with *.

^{1}H NMR (400 MHz, CDCl$_3$) δ 7.37 – 7.24 (m, 2H), 7.25 – 7.14 (m, 3H), 6.82/6.09* (s, 1H), 3.58 – 3.46 (m, 2H), 3.34/3.29* (t, J = 6.7 Hz, 2H), 3.01 – 2.69 (m, 5H), 2.20 – 2.01 (m, 5H), 1.98 – 1.75 (m, 2H).

^{13}C NMR (101 MHz, CDCl$_3$) δ 172.5/171.6*, 171.3/170.6*, 138.9/138.7*, 128.6/128.6*, 128.5*/128.3, 126.4*/126.2, 49.8*/46.4, 40.6/40.5*, 35.9/35.5*, 35.5/33.0*, 33.2,/32.3*, 23.6*/23.2, 21.7/21.0*.

HRMS (ESI-TOF) m/z: [M + H]+ Calcd for $C_{15}H_{23}N_2O_2$ 263.1754; Found: 263.1759.

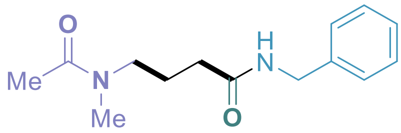

***N*-benzyl-4-(*N*-methylacetamido)butanamide (5t)**

This reaction was conducted on a 0.3 mmol scale with the general procedure II. The crude product was purified by silica gel chromatography (EA/MeOH = 20:1) to afford the title compound as a colorless oil (49.1 mg, 66% yield). The identity of the product was confirmed by ^{1}H NMR, ^{13}C NMR, and HRMS. Analysis of NMR spectra revealed the presence of two amide bond rotamers. These conformational isomers have been observed and studied in similar compounds.[1] The minor distinguishable rotamer peak is denoted with *.

^{1}H NMR (400 MHz, CDCl$_3$) δ 7.43 – 6.70 (m, 6H), 4.42/4.40* (s, 2H), 3.38/3.31* (t, J = 6.7 Hz, 2H), , 2.96/2.86* (s, 3H), 2.42 – 2.11 (m, 2H), 2.03*/2.02 (s, 3H), 1.95 – 1.81 (m, 2H).

^{13}C NMR (101 MHz, CDCl$_3$) δ 172.5/171.6*, 171.3/170.6*, 138.5/138.2*, 128.5*/128.4, 127.7*/127.6, 127.3*/127.1, 49.8*/46.5, 43.4*/43.3, 35.9/32.9*, 33.2/32.2*, 23.6*/23.2, 21.7/21.0*.

HRMS (ESI-TOF) m/z: [M + H]+ Calcd for $C_{14}H_{21}N_2O_2$ 249.1598; Found: 249.1589.

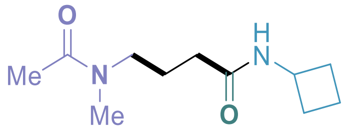

***N*-cyclobutyl-4-(*N*-methylacetamido)butanamide (5u)**

This reaction was conducted on a 0.3 mmol scale with the general procedure II. The crude product was purified by silica gel chromatography (EA/MeOH = 20:1) to afford the title compound as a colorless oil (49.0 mg, 77% yield). The identity of the product was confirmed by ^{1}H NMR, ^{13}C NMR, and HRMS. Analysis of NMR spectra revealed the presence of two amide bond rotamers. These conformational isomers have been observed and studied in similar compounds.[1] The minor distinguishable rotamer peak is denoted with *.

^{1}H NMR (700 MHz, CDCl$_3$) δ 7.06/6.32* (s, 1H), 4.43 – 4.20 (m, 1H), 3.51 – 3.23 (m, 2H), 2.94/2.85* (s, 3H), 2.46 – 2.19 (m, 2H), 2.16 – 1.96 (m, 5H), 1.93 – 1.71 (m, 2H), 1.71 – 1.48 (m, 4H).

^{13}C NMR (176 MHz, CDCl$_3$) δ 171.6, 171.4/170.7*, 49.8*/46.5, 44.5*/44.5, 36.0/32.9*, 33.2/32.2*, 31.0*/30.9, 23.6*/23.2, 21.8/21.1*, 15.0/15.0*.
HRMS (ESI-TOF) m/z: [M + H]+ Calcd for $C_{11}H_{21}N_2O_2$ 213.1598; Found: 213.1596.

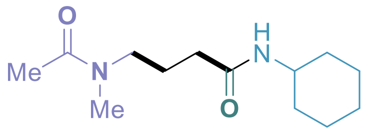

***N*-cyclohexyl-4-(*N*-methylacetamido)butanamide (5v)**

This reaction was conducted on a 0.3 mmol scale with the general procedure II. The crude product was purified by silica gel chromatography (EA/MeOH = 20:1) to afford the title compound as a colorless oil (59.0 mg, 82% yield). The identity of the product was confirmed by ^{1}H NMR, ^{13}C NMR, and HRMS. Analysis of NMR spectra revealed the presence of two amide bond rotamers. These conformational isomers have been observed and studied in similar compounds.[1] The minor distinguishable rotamer peak is denoted with *.

^{1}H NMR (400 MHz, CDCl$_3$) δ 6.65/5.72* (s, 1H), 3.84 – 3.57 (m, 1H), 3.37/3.30* (t, J = 6.5 Hz, 2H), 2.95/2.86* (s, 3H), 2.39 – 1.98 (m, 5H), 1.95 – 1.73 (m, 4H), 1.73 – 1.62 (m, 2H), 1.62 – 1.50 (m, 1H), 1.45 – 1.23 (m, 2H), 1.23 – 0.97 (m, 3H).
^{13}C NMR (101 MHz, CDCl$_3$) δ 171.5/170.6*, 171.3/170.6*, 49.9*/48.1, 48.2*/46.4, 36.0/33.1*, 33.4/33.0*, 32.9/32.5*, 25.4/25.4*, 24.8/24.8*, 23.7*/23.4, 21.8/21.1*.
HRMS (ESI-TOF) m/z: [M + H]+ Calcd for $C_{13}H_{25}N_2O_2$ 241.1911; Found: 241.1919.

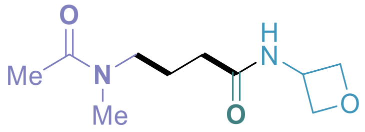

4-(*N*-methylacetamido)-*N*-(oxetan-3-yl)butanamide (5w)

This reaction was conducted on a 0.3 mmol scale with the general procedure II. The crude product was purified by silica gel chromatography (EA/MeOH = 20:1) to afford the title compound as a colorless oil (44.3 mg, 69% yield). The identity of the product was confirmed by ^{1}H NMR, ^{13}C NMR, and HRMS. Analysis of NMR spectra revealed the presence of two amide bond rotamers. These conformational isomers have been observed and studied in similar compounds.[1] The minor distinguishable rotamer peak is denoted with *.

¹H NMR (700 MHz, CDCl₃) δ 8.03/7.27* (s, 1H), 5.12 – 4.99 (m, 1H), 4.97 – 4.81 (m, 2H), 4.59/4.51* (t, J = 6.4 Hz, 2H), 3.43/3.35* (t, J = 7.3 Hz, 2H), 3.01/2.91* (s, 3H), 2.33 – 2.14 (m, 2H), 2.12/2.10* (s, 3H), 1.97 – 1.73 (m, 2H).
¹³C NMR (176 MHz, CDCl₃) δ 172.4/171.4*, 171.6/170.7*, 78.3*/78.3, 49.8*/46.5, 44.5, 36.0/33.0*, 33.0/32.0*, 23.4*/23.2, 21.8/21.1*.
HRMS (ESI-TOF) m/z: [M + H]+ Calcd for $C_{10}H_{19}N_2O_3$ 215.1390; Found: 215.1389.

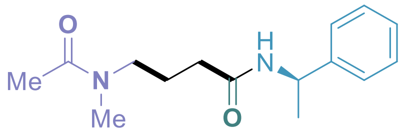

(*R*)-4-(*N*-methylacetamido)-*N*-(1-phenylethyl)butanamide (5x)

This reaction was conducted on a 0.3 mmol scale with the general procedure II. The crude product was purified by silica gel chromatography (EA/MeOH = 20:1) to afford the title compound as a colorless oil (56.6 mg, 72% yield). The identity of the product was confirmed by ¹H NMR, ¹³C NMR, and HRMS. Analysis of NMR spectra revealed the presence of two amide bond rotamers. These conformational isomers have been observed and studied in similar compounds.[1] The minor distinguishable rotamer peak is denoted with *.

¹H NMR (700 MHz, CDCl₃) δ 7.35 – 7.29 (m, 4H), 7.27 – 7.14 (m, 2H), 5.52 – 4.68 (m, 1H), 3.73 – 3.24 (m, 2H), 3.14 – 2.70 (m, 3H), 2.26 – 2.12 (m, 2H), 2.10 – 1.96 (m, 3H), 1.94 – 1.75 (m, 2H), 1.55 – 1.36 (m, 3H).
¹³C NMR (176 MHz, CDCl₃) δ 171.7, 171.4/170.7*, 143.7, 128.6*/128.4, 127.3*/127.0, 126.1*/126.1, 48.8*/46.5, 36.0/33.0*, 33.3/32.4*, 23.6*/23.3, 22.2/21.7*, 21.8/21.1*.
HRMS (ESI-TOF) m/z: [M + H]+ Calcd for $C_{15}H_{23}N_2O_2$ 263.1754; Found: 263.1761.

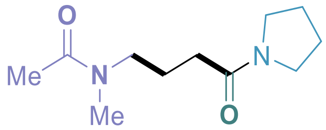

***N*-methyl-*N*-(4-oxo-4-(pyrrolidin-1-yl)butyl)acetamide (5y)**

This reaction was conducted on a 0.3 mmol scale with the general procedure II. The crude product was purified by silica gel chromatography (EA/MeOH = 20:1) to afford the title compound as a colorless oil (45.2 mg, 71% yield). The identity of the product was confirmed by ¹H NMR, ¹³C NMR, and HRMS. Analysis of NMR spectra revealed the presence of two amide bond rotamers. These conformational isomers have been

observed and studied in similar compounds.[1] The minor distinguishable rotamer peak is denoted with *.

^{1}H NMR (400 MHz, $CDCl_3$) δ 3.52 – 3.27 (m, 6H), 2.97/2.89* (s, 3H), 2.43 – 2.18 (m, 2H), 2.07*/2.02 (s, 3H), 1.99 – 1.69 (m, 6H).

^{13}C NMR (101 MHz, $CDCl_3$) δ 170.8/170.6*, 170.6*/170.0, 50.0*/46.8, 46.5/46.4*, 45.6*/45.6, 35.9/33.0*, 31.6/30.6*, 26.0*/26.0, 24.3/24.3*, 22.9*/22.2, 21.8/21.1*.

HRMS (ESI-TOF) m/z: [M + H]+ Calcd for $C_{11}H_{21}N_2O_2$ 213.1598; Found: 213.1599.

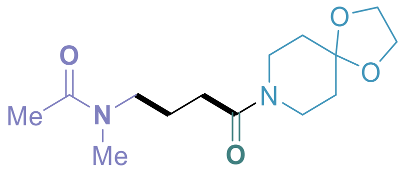

_N_-methyl-_N_-(4-oxo-4-(1,4-dioxa-8-azaspiro[4.5]decan-8-yl)butyl)acetamide (5z)

This reaction was conducted on a 0.3 mmol scale with the general procedure II. The crude product was purified by silica gel chromatography (EA/MeOH = 20:1) to afford the title compound as a colorless oil (66.5 mg, 78% yield). The identity of the product was confirmed by ^{1}H NMR, ^{13}C NMR, and HRMS. Analysis of NMR spectra revealed the presence of two amide bond rotamers. These conformational isomers have been observed and studied in similar compounds.[1] The minor distinguishable rotamer peak is denoted with *.

^{1}H NMR (400 MHz, $CDCl_3$) δ 3.94*/3.94 (s, 4H), 3.65 (q, J = 5.6 Hz, 2H), 3.55 – 3.45 (m, 2H), 3.44 – 3.24 (m, 2H), 2.97/2.88* (s, 3H), 2.31 (t, J = 7.4 Hz, 2H), 2.07*/2.03 (s, 3H), 1.95 – 1.78 (m, 2H), 1.76 – 1.59 (m, 4H).

^{13}C NMR (101 MHz, $CDCl_3$) δ 170.7/170.7*, 170.6/169.7*, 106.8/106.7*, 64.4*/64.4, 50.1*/46.8, 43.4/43.3*, 39.8*/39.7, 35.9/35.4*, 35.4/34.6*, 34.6/33.1*, 30.1/29.1*, 23.3*/22.6, 21.8/21.1*.

HRMS (ESI-TOF) m/z: [M + H]+ Calcd for $C_{14}H_{25}N_2O_4$ 285.1809; Found: 285.1807.

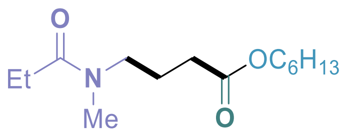

hexyl 4-(_N_-methylpropionamido)butanoate (6a)

This reaction was conducted on a 0.3 mmol scale with the general procedure III. The crude product was purified by silica gel chromatography (PE/EA = 1:1) to afford the title compound as a colorless oil (64.0 mg, 83% yield). The identity of the product was

confirmed by ^{1}H NMR, ^{13}C NMR, and HRMS. Analysis of NMR spectra revealed the presence of two amide bond rotamers. These conformational isomers have been observed and studied in similar compounds.[1] The minor distinguishable rotamer peak is denoted with *.

^{1}H NMR (700 MHz, $CDCl_3$) δ 4.12 – 3.94 (m, 2H), 3.39/3.31* (t, J = 7.3 Hz, 2H), 2.97/2.91* (s, 3H), 2.38 – 2.24 (m, 4H), 1.97 – 1.80 (m, 2H), 1.60 (q, J = 7.8 Hz, 2H), 1.38 – 1.23 (m, 6H), 1.19 – 1.07 (m, 3H), 0.95 – 0.84 (m, 3H).

^{13}C NMR (176 MHz, $CDCl_3$) δ 173.7/173.7*, 173.3/172.8*, 64.9*/64.6, 48.8*/46.9, 35.2*/33.3, 31.5/31.4*, 31.4/30.9*, 28.5/28.1*, 26.7*/25.5, 25.5/23.4*, 22.6*/22.5, 14.0, 9.6*/9.2.

HRMS (ESI-TOF) m/z: [M + H]+ Calcd for$C_{14}H_{28}NO_3$ 258.2064; Found: 258.2065.

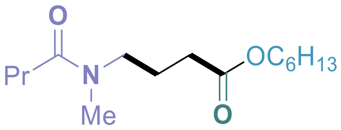

hexyl 4-(*N*-methylbutyramido)butanoate (6b)

This reaction was conducted on a 0.3 mmol scale with the general procedure III. The crude product was purified by silica gel chromatography (PE/EA = 1:1) to afford the title compound as a colorless oil (64.2 mg, 79% yield). The identity of the product was confirmed by ^{1}H NMR, ^{13}C NMR, and HRMS. Analysis of NMR spectra revealed the presence of two amide bond rotamers. These conformational isomers have been observed and studied in similar compounds.[1] The minor distinguishable rotamer peak is denoted with *.

^{1}H NMR (700 MHz, $CDCl_3$) δ 4.07/4.04* (t, J = 6.8 Hz, 2H), 3.39/3.31* (t, J = 7.2 Hz, 2H), 2.97/2.91* (s, 3H), 2.51 – 2.15 (m, 4H), 1.97 – 1.79 (m, 2H), 1.70 – 1.52 (m, 4H), 1.37 – 1.16 (m, 6H), 0.95 (t, J = 7.4 Hz, 3H), 0.91 – 0.78 (m, 3H).

^{13}C NMR (176 MHz, $CDCl_3$) δ 173.3/173.0*, 172.9/172.8*, 64.9*/64.6, 48.9/48.9*, 46.8*/46.8, 35.5*/35.5, 35.3*/35.3, 34.8*/34.8, 31.5*/31.4, 30.9*/28.5, 28.1/25.5*, 23.6*/23.5, 22.6*/22.5, 18.8*/18.4, 14.0*/14.0.

HRMS (ESI-TOF) m/z: [M + H]+ Calcd for $C_{15}H_{30}NO_3$ 272.2220; Found: 272.2225.

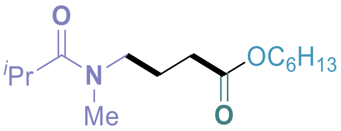

hexyl 4-(*N*-methylisobutyramido)butanoate (6c)

This reaction was conducted on a 0.3 mmol scale with the general procedure III. The crude product was purified by silica gel chromatography (PE/EA = 1:1) to afford the title compound as a colorless oil (61.0 mg, 75% yield). The identity of the product was confirmed by ^{1}H NMR, ^{13}C NMR, and HRMS. Analysis of NMR spectra revealed the presence of two amide bond rotamers. These conformational isomers have been observed and studied in similar compounds.[1] The minor distinguishable rotamer peak is denoted with *.

^{1}H NMR (400 MHz, CDCl$_3$) δ 4.21 – 3.97 (m, 2H), 3.59 – 3.30 (m, 2H), 3.03/2.92* (s, 3H), 2.88 – 2.66 (m, 1H), 2.45 – 2.25 (m, 2H), 1.96 – 1.74 (m, 2H), 1.69 – 1.56 (m, 2H), 1.45 – 1.23 (m, 6H), 1.19 – 1.01 (m, 6H), 0.98 – 0.81 (m, 3H).

^{13}C NMR (101 MHz, CDCl$_3$) δ 177.2*/176.9, 173.4/172.8*, 64.9*/64.6, 48.8*/47.0, 35.1/33.5*, 31.4*/31.4, 30.9*/30.4, 29.9/28.5*, 28.1/28.0*, 25.5/23.9*, 22.5*/22.5, 19.7*/19.2, 13.9.

HRMS (ESI-TOF) m/z: [M + H]+ Calcd for $C_{15}H_{30}NO_3$ 272.2220; Found: 272.2230.

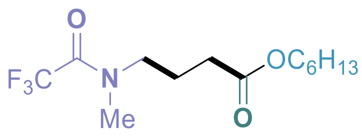

hexyl 4-(2,2,2-trifluoro-*N*-methylacetamido)butanoate (6d)

This reaction was conducted on a 0.3 mmol scale with the general procedure III. The crude product was purified by silica gel chromatography (PE/EA = 2:1) to afford the title compound as a colorless oil (45.4 mg, 51% yield). The identity of the product was confirmed by ^{1}H NMR, ^{13}C NMR, and HRMS. Analysis of NMR spectra revealed the presence of two amide bond rotamers. These conformational isomers have been observed and studied in similar compounds.[1] The minor distinguishable rotamer peak is denoted with *.

^{1}H NMR (700 MHz, CDCl$_3$) δ 4.07 (q, J = 6.9 Hz, 2H), 3.47/3.44* (t, J = 8.4 Hz, 2H), 3.13/3.03* (s, 3H), 2.54 – 2.24 (m, 2H), 2.12 – 1.81 (m, 2H), 1.72 – 1.50 (m, 2H), 1.42 – 1.22 (m, 6H), 0.88 (t, J = 7.0 Hz, 3H).

^{13}C NMR (176 MHz, CDCl$_3$) δ 172.8/172.4*, 157.0/156.9* (q, J = 35.8 Hz), 116.5/116.4* (q, J = 288.0 Hz), 65.0*/64.8, 48.7/48.6*, 34.8/34.3*, 31.4/31.4*, 31.1/30.7*, 28.5*/28.5, 25.5/23.2*, 22.5/21.7*, 14.0.

HRMS (ESI-TOF) m/z: [M + H]+ Calcd for $C_{13}H_{23}NO_3F_3$ 298.1625; Found: 298.1630.

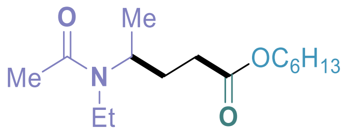

hexyl-4-(*N*-ethylacetamido)pentanoate (6e)

This reaction was conducted on a 0.3 mmol scale with the general procedure III. The crude product was purified by silica gel chromatography (PE/EA = 1:1) to afford the title compound as a colorless oil (45.5 mg, 56% yield). The identity of the product was confirmed by ^{1}H NMR, ^{13}C NMR, and HRMS. Analysis of NMR spectra revealed the presence of two amide bond rotamers. These conformational isomers have been observed and studied in similar compounds.[1] The minor distinguishable rotamer peak is denoted with *.

^{1}H NMR (400 MHz, CDCl$_3$) δ 4.12 – 3.98 (m, 2H), 3.70 – 3.01 (m, 3H), 2.53 – 2.15 (m, 2H), 2.10/2.08* (s, 3H), 1.96 – 1.72 (m, 2H), 1.61 (td, J = 6.9, 3.4 Hz, 2H), 1.36 – 1.26 (m, 6H), 1.25 – 1.12 (m, 6H), 0.89 (t, J = 6.2 Hz, 3H).
^{13}C NMR (101 MHz, CDCl$_3$) δ 173.4/173.0*, 170.7/170.1*, 64.8*/64.6, 53.0/49.2*, 38.7*/35.4, 32.7*/31.5, 31.9/31.5*, 31.5/30.8*, 29.9*/29.5, 28.5*/28.0, 25.5/22.5*, 22.2/22.0*, 19.7*/19.0, 16.2, 14.6*/13.9.
HRMS (ESI-TOF) m/z: [M + H]+ Calcd for $C_{15}H_{30}NO_3$ 272.2220; Found: 272.2220.

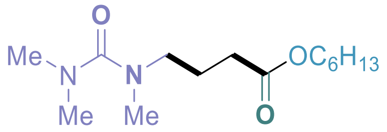

hexyl 4-(1,3,3-trimethylureido)butanoate (6f)

This reaction was conducted on a 0.3 mmol scale with the general procedure III. The crude product was purified by silica gel chromatography (EA/MeOH = 40:1) to afford the title compound as a colorless oil (43.2 mg, 53% yield). The identity of the product was confirmed by ^{1}H NMR, ^{13}C NMR, and HRMS.

^{1}H NMR (400 MHz, CDCl$_3$) δ 4.05 (t, J = 6.8 Hz, 2H), 3.57 – 2.99 (m, 2H), 2.80 (s, 3H), 2.79 (s, 6H), 2.30 (t, J = 7.4 Hz, 2H), 2.03 – 1.78 (m, 2H), 1.71 – 1.55 (m, 2H), 1.38 – 1.23 (m, 6H), 1.06 – 0.83 (m, 3H).
^{13}C NMR (101 MHz, CDCl$_3$) δ 173.3, 165.5, 64.6, 49.5, 38.8, 36.7, 31.5, 31.4, 28.6, 25.6, 22.8, 22.5, 14.0.
HRMS (ESI-TOF) m/z: [M + H]+ Calcd for $C_{14}H_{29}N_2O_3$ 273.2189; Found: 273.2189.

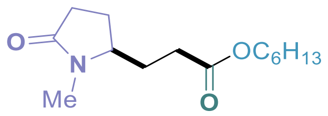

hexyl-3-(1-methyl-5-oxopyrrolidin-2-yl)propanoate (6g)

This reaction was conducted on a 0.3 mmol scale with the general procedure III. The crude product was purified by silica gel chromatography (PE/EA = 1:2) to afford the title compound as a colorless oil (49.0 mg, 64% yield). The identity of the product was confirmed by ^{1}H NMR, ^{13}C NMR, and HRMS. Analysis of NMR spectra revealed the presence of two amide bond rotamers. These conformational isomers have been observed and studied in similar compounds.[1] The minor distinguishable rotamer peak is denoted with *.

^{1}H NMR (400 MHz, $CDCl_3$) δ 4.03 (t, J = 6.8 Hz, 2H), 2.78 – 2.74 (m, 4H), 2.50 – 2.19 (m, 4H), 2.20 – 1.95 (m, 2H), 1.75 – 1.51 (m, 4H), 1.34 – 1.13 (m, 6H), 0.84 (t, J = 6.8 Hz, 3H).

^{13}C NMR (101 MHz, $CDCl_3$) δ 174.8, 172.7/171.9*, 64.8, 59.0*/58.9, 31.3/30.4*, 29.9*/29.8, 29.2, 28.4, 28.1*/28.1, 27.9/27.6*, 25.4, 23.4*/23.4, 22.4, 13.9.

HRMS (ESI-TOF) m/z: [M + H]+ Calcd for $C_{14}H_{26}NO_3$ 256.1907; Found: 256.1905.

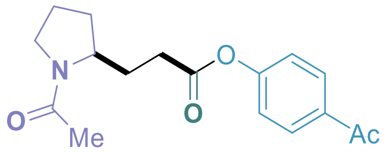

4-acetylphenyl-3-(1-acetylpyrrolidin-2-yl)propanoate (6h)

This reaction was conducted on a 0.3 mmol scale with the general procedure III. The crude product was purified by silica gel chromatography (PE/EA = 1:2) to afford the title compound as a colorless oil (64.5 mg, 71% yield). The identity of the product was confirmed by ^{1}H NMR, ^{13}C NMR, and HRMS. Analysis of NMR spectra revealed the presence of two amide bond rotamers. These conformational isomers have been observed and studied in similar compounds.[1] The minor distinguishable rotamer peak is denoted with *.

^{1}H NMR (400 MHz, $CDCl_3$) δ 7.98 (d, J = 8.7 Hz, 2H), 7.22 (d, J = 8.6 Hz, 2H), 4.35 – 4.11 (m, 1H), 3.73 – 3.23 (m, 2H), 2.75 – 2.53 (m, 6H), 2.18 – 2.08 (m, 1H), 2.05 (s, 3H), 2.00 – 1.89 (m, 2H), 1.89 – 1.77 (m, 1H), 1.76 – 1.66 (m, 1H).

^{13}C NMR (101 MHz, $CDCl_3$) δ 196.8/196.7*, 171.3/170.7*, 169.7/169.2*, 154.4/154.0*, 134.8*/134.5, 129.9*/129.8, 121.7/121.6*, 57.3*/55.8, 47.5/45.3*, 31.6/30.8*, 29.9*/29.8, 29.2*/28.9, 26.5, 23.9*/23.9, 22.9*/22.8, 22.0.

HRMS (ESI-TOF) m/z: [M + H]+ Calcd for $C_{17}H_{22}NO_4$ 304.1543; Found: 304.1544.

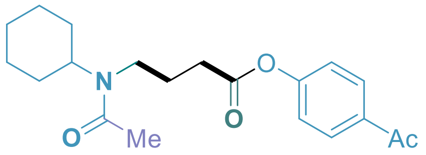

4-acetylphenyl 4-(*N*-cyclohexylacetamido)butanoate (6i)

This reaction was conducted on a 0.3 mmol scale with the general procedure III. The crude product was purified by silica gel chromatography (PE/EA = 1:2) to afford the title compound as a colorless oil (68.0 mg, 56% yield). The identity of the product was confirmed by ^{1}H NMR, ^{13}C NMR, and HRMS. Analysis of NMR spectra revealed the presence of two amide bond rotamers. These conformational isomers have been observed and studied in similar compounds.[1] The minor distinguishable rotamer peak is denoted with *.

^{1}H NMR (400 MHz, CDCl$_3$) δ 8.00 (d, J = 8.7 Hz, 2H), 7.21 (d, J = 8.7 Hz, 2H), 3.55 – 3.41 (m, 1H), 3.40 – 3.19 (m, 2H), 2.68 – 2.53 (m, 5H), 2.14/2.13* (s, 3H), 2.03 – 1.91 (m, 2H), 1.90 – 1.73 (m, 4H), 1.72 – 1.60 (m, 2H), 1.59 – 1.27 (m, 4H).

^{13}C NMR (101 MHz, CDCl$_3$) δ 196.8, 171.4/170.7*, 170.4, 154.4/154.1*, 134.9*/134.6, 130.0*/129.9, 121.8/121.6*, 58.2/53.5*, 43.7*/40.7, 32.0*/31.7, 31.4*/31.0, 28.0*/26.6, 26.0/25.9*, 25.8*/25.5, 25.2/24.7*, 22.1.

HRMS (ESI-TOF) m/z: [M + H]+ Calcd for $C_{20}H_{28}NO_4$ 346.2013; Found: 346.2009.

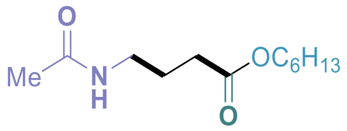

hexyl 4-acetamidobutanoate (6j)

This reaction was conducted on a 0.3 mmol scale with the general procedure V. The crude product was purified by silica gel chromatography (EA/MeOH = 20:1) to afford the title compound as a white solid (39.2 mg, 57% yield). The identity of the product was confirmed by ^{1}H NMR, ^{13}C NMR, and HRMS.

^{1}H NMR (400 MHz, CDCl$_3$) δ 5.93 (s, 1H), 4.04 (t, J = 6.8 Hz, 2H), 3.61 – 3.03 (m, 2H), 2.34 (t, J = 7.2 Hz, 2H), 1.94 (s, 3H), 1.90 – 1.68 (m, 2H), 1.68 – 1.46 (m, 2H), 1.44 – 1.10 (m, 6H), 1.02 – 0.56 (m, 3H).

^{13}C NMR (101 MHz, CDCl$_3$) δ 173.5, 170.2, 64.7, 39.0, 31.7, 31.3, 28.5, 25.5, 24.6, 23.2, 22.4, 13.9.

HRMS (ESI-TOF) m/z: [M + H]+ Calcd for $C_{12}H_{24}NO_3$ 230.1751; Found: 230.1760.

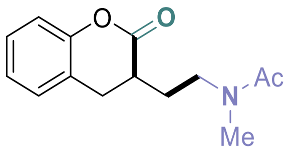

***N*-methyl-*N*-(2-(2-oxochroman-3-yl)ethyl)acetamide (7a)**

This reaction was conducted on a 0.3 mmol scale with the general procedure I. The crude product was purified by silica gel chromatography (PE/EA = 1:1) to afford the title compound as a colorless oil (51.1 mg, 69% yield). The identity of the product was confirmed by ^{1}H NMR, ^{13}C NMR, and HRMS. Analysis of NMR spectra revealed the presence of two amide bond rotamers. These conformational isomers have been observed and studied in similar compounds.[1] The minor distinguishable rotamer peak is denoted with *.

^{1}H NMR (400 MHz, CDCl$_3$) δ 7.36 – 7.16 (m, 2H), 7.16 – 6.98 (m, 2H), 3.77 – 3.54 (m, 1H), 3.54 – 3.31 (m, 1H), 3.23 – 2.81 (m, 5H), 2.76 – 2.53 (m, 1H), 2.14*/2.07 (s, 3H), 2.00 – 1.63 (m, 2H).
^{13}C NMR (101 MHz, CDCl$_3$) δ 171.4*/171,0 170.9/170.3*, 151.5, 128.6*/128.2, 128.2/127.9*, 124.6*/124.4, 122.7/122.2*, 116.7*/116.5, 48.6*/44.9, 36.9/36.6*, 36.1/33.2*, 30.1*/29.3, 29.0*/27.5, 21.8/21.2*.
HRMS (ESI-TOF) m/z: [M + H]+ Calcd for $C_{14}H_{18}NO_3$ 248.1281; Found: 248.1276.

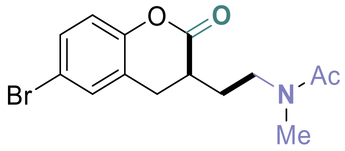

***N*-(2-(6-bromo-2-oxochroman-3-yl)ethyl)-*N*-methylacetamide (7b)**

This reaction was conducted on a 0.3 mmol scale with the general procedure I. The crude product was purified by silica gel chromatography (PE/EA = 1:1) to afford the title compound as a colorless oil (66.3 mg, 68% yield,). The identity of the product was confirmed by ^{1}H NMR, ^{13}C NMR, and HRMS. Analysis of NMR spectra revealed the presence of two amide bond rotamers. These conformational isomers have been observed and studied in similar compounds.[1] The minor distinguishable rotamer peak is denoted with *.

^{1}H NMR (400 MHz, CDCl$_3$) δ 7.35 (d, J = 7.0 Hz, 2H), 7.00 – 6.85 (m, 1H), 3.86 – 3.54 (m, 1H), 3.47 – 3.30 (m, 1H), 3.21 – 3.07 (m, 1H), 3.07 – 2.76 (m, 4H), 2.73 – 2.59 (m, 1H), 2.14*/2.07 (s, 3H), 1.86 – 1.68 (m, 2H).

^{13}C NMR (101 MHz, CDCl$_3$) δ 171.0/170.5*, 170.2/169.5*, 150.5, 131.6*/131.2, 131.0/130.7*, 124.8/124.3*, 118.4*/118.2, 117.1*/116.9, 48.4*/44.7, 36.4/36.2*, 36.1/33.1*, 29.9*/29.0, 29.0/27.5*, 21.8/21.1*.
HRMS (ESI-TOF) m/z: [M + H]+ Calcd for $C_{14}H_{17}BrNO_3$ 326.0386; Found: 326.0390.

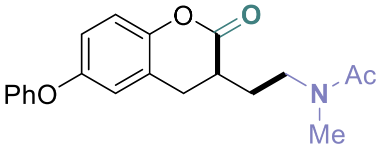

***N*-methyl-*N*-(2-(2-oxo-6-phenoxychroman-3-yl)ethyl)acetamide (7c)**

This reaction was conducted on a 0.3 mmol scale with the general procedure I. The crude product was purified by silica gel chromatography (PE/EA = 1:2) to afford the title compound as a colorless oil (53.9 mg, 53% yield,). The identity of the product was confirmed by ^{1}H NMR, ^{13}C NMR, and HRMS. Analysis of NMR spectra revealed the presence of two amide bond rotamers. These conformational isomers have been observed and studied in similar compounds.[1] The minor distinguishable rotamer peak is denoted with *.

^{1}H NMR (700 MHz, CDCl$_3$) δ 7.40 – 7.31 (m, 2H), 7.16 – 7.07 (m, 1H), 7.03 – 6.95 (m, 3H), 6.94 – 6.86 (m, 1H), 6.84 (dd, J = 12.0, 2.9 Hz, 1H), 3.79 – 3.54 (m, 1H), 3.52 – 3.36 (m, 1H), 3.17 – 2.92 (m, 4H), 2.91 – 2.81 (m, 1H), 2.76 – 2.58 (m, 1H), 2.29 – 2.14 (m, 1H), 2.14*/2.07 (s, 3H), 1.94 – 1.68 (m, 1H).
^{13}C NMR (176 MHz, CDCl$_3$) δ 170.8*/170.8, 170.6*/170.1, 157.2/157.1*, 153.6*/153.4, 147.1/147.0*, 129.8*/129.8, 123.5*/123.3, 119.0*/118.9, 118.7*/118.6, 118.6/118.5*, 118.3/118.0*, 117.8*/117.5, 48.5*/44.9, 36.6/36.2*, 36.1/33.1*, 30.2*/29.4, 29.0*/27.5, 21.8/21.2*.
HRMS (ESI-TOF) m/z: [M + H]+ Calcd for $C_{20}H_{22}NO_4$ 340.1543; Found: 340.1546.

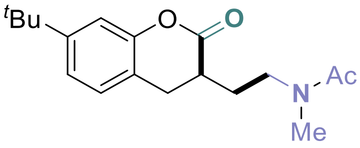

***N*-(2-(7-(*tert*-butyl)-2-oxochroman-3-yl)ethyl)-*N*-methylacetamide (7d)**

This reaction was conducted on a 0.3 mmol scale with the general procedure I. The crude product was purified by silica gel chromatography (PE/EA = 1:1) to afford the title compound as a colorless oil (73.6 mg, 81% yield). The identity of the product was confirmed by ^{1}H NMR, ^{13}C NMR, and HRMS. Analysis of NMR spectra revealed the presence of two amide bond rotamers. These conformational isomers have been

observed and studied in similar compounds.[1] The minor distinguishable rotamer peak is denoted with *.

^{1}H NMR (400 MHz, $CDCl_3$) δ 7.31 – 7.22 (m, 1H), 7.18 (t, J = 2.8 Hz, 1H), 7.03 – 6.93 (m, 1H), 3.78 – 3.57 (m, 1H), 3.57 – 3.35 (m, 1H), 3.21 – 2.84 (m, 5H), 2.75 – 2.58 (m, 1H), 2.14*/2.07 (s, 3H), 1.95 – 1.67 (m, 2H), 1.43 – 1.19 (m, 9H).

^{13}C NMR (101 MHz, $CDCl_3$) δ 171.2/170.9*, 170.6/170.2*, 149.2*/147.7, 148.7*/147.4,125.4*/125.1,125.0/124.8*, 121.8/121.5*, 116.1*/115.9, 48.6*/45.0, 37.0/36.7*, 36.1/34.4*, 33.2*/31.6, 31.4/31.4*, 30.4*/29.7, 29.1*/27.6, 21.9/21.2*.

HRMS (ESI-TOF) m/z: [M + H]+ Calcd for $C_{18}H_{26}NO_3$ 304.1907; Found: 304.1908.

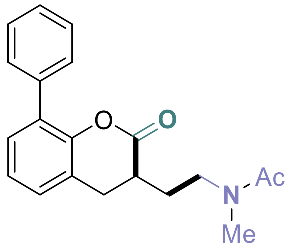

***N*-methyl-*N*-(2-(2-oxo-8-phenylchroman-3-yl)ethyl)acetamide (7e)**

This reaction was conducted on a 0.3 mmol scale with the general procedure I. The crude product was purified by silica gel chromatography (PE/EA = 1:1) to afford the title compound as a colorless oil (58.1 mg, 60% yield). The identity of the product was confirmed by ^{1}H NMR, ^{13}C NMR, and HRMS. Analysis of NMR spectra revealed the presence of two amide bond rotamers. These conformational isomers have been observed and studied in similar compounds.[1] The minor distinguishable rotamer peak is denoted with *.

^{1}H NMR (400 MHz, $CDCl_3$) δ 7.51 (dd, J = 8.3, 1.5 Hz, 2H), 7.47 – 7.39 (m, 2H), 7.39 – 7.28 (m, 2H), 7.23 – 7.12 (m, 2H), 3.82 – 3.54 (m, 1H), 3.49 – 3.36 (m, 1H), 3.32 – 3.13 (m, 1H), 3.07 – 2.89 (m, 4H), 2.75 – 2.54 (m, 1H), 2.13*/2.07 (s, 3H), 1.97 – 1.58 (m, 2H).

^{13}C NMR (101 MHz, $CDCl_3$) δ 172.3*/170.7, 168.9/166.6*, 148.2/148.1*, 136.5/136.3*, 130.2*/130.1, 130.0*/129.8, 129.4/129.4*, 128.3*/128.2, 127.7*/127.5, 127.3/127.0*, 124.5*/124.3, 123.4/123.0*, 48.6*/44.9, 36.7/36.5*, 36.1/33.2*, 30.6*/29.7, 29.0*/27.5, 21.9/21.2*.

HRMS (ESI-TOF) m/z: [M + H]+ Calcd for $C_{20}H_{22}NO_3$ 324.1594; Found: 324.1597.

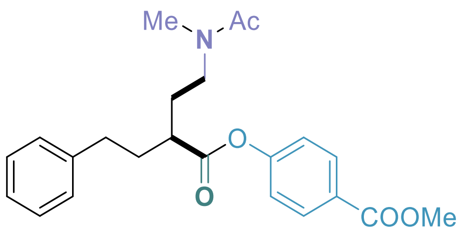

methyl-4-((4-(*N*-methylacetamido)-2-phenethylbutanoyl)oxy)benzoate (7f)

This reaction was conducted on a 0.3 mmol scale with the general procedure I. The crude product was purified by silica gel chromatography (PE/EA = 1:2) to afford the title compound as a colorless oil (61.9 mg, 52% yield). The identity of the product was confirmed by ^{1}H NMR, ^{13}C NMR, and HRMS. Analysis of NMR spectra revealed the presence of two amide bond rotamers. These conformational isomers have been observed and studied in similar compounds.[1] The minor distinguishable rotamer peak is denoted with *.

^{1}H NMR (400 MHz, CDCl$_3$) δ 8.17 – 7.83 (m, 2H), 7.53 – 7.24 (m, 2H), 7.27 – 6.96 (m, 5H), 3.92/3.91* (s, 3H), 3.47 – 3.28 (m, 2H), 2.98/2.92* (s, 3H), 2.84 – 2.71 (m, 2H), 2.18 – 1.95 (m, 8H).

^{13}C NMR (101 MHz, CDCl$_3$) δ 173.5/171.8*,173.0/171.7*, 166.3/165.3*, 154.3/154.0*, 141.0/140.5*, 131.3*/131.1, 128.6*/128.5, 128.5*/128.4, 128.3/128.3*, 126.4*/126.1, 121.6/121.4*, 52.2*/52.2, 48.7*/45.5, 42.6/42.4*, 36.1, 34.1*/34.0, 33.4/33.2*, 30.3*/29.2, 21.8/21.2*.

HRMS (ESI-TOF) m/z: [M + H]+ Calcd for $C_{23}H_{28}NO_5$ 398.1962; Found: 398.1270.

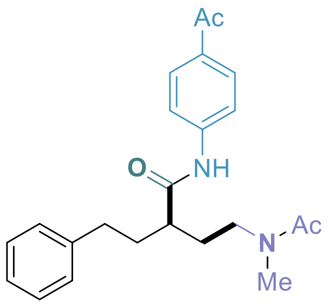

***N*-(4-acetylphenyl)-4-(*N*-methylacetamido)-2-phenethylbutanamide (7g)**

This reaction was conducted on a 0.3 mmol scale with the general procedure I. The crude product was purified by silica gel chromatography (EA/MeOH = 10:1) to afford the title compound as a colorless oil (88.9 mg, 78% yield). The identity of the product was confirmed by ^{1}H NMR, ^{13}C NMR, and HRMS. Analysis of NMR spectra revealed the presence of two amide bond rotamers. These conformational isomers have been

observed and studied in similar compounds.[1] The minor distinguishable rotamer peak is denoted with *.

^{1}H NMR (400 MHz, CDCl$_3$) δ 10.07 (s, 1H), 7.94 (d, J = 8.8 Hz, 2H), 7.82 (d, J = 8.7 Hz, 2H), 7.58 – 7.24 (m, 2H), 7.24 – 6.91 (m, 3H), 3.08 – 2.91 (m, 5H), 2.69 – 2.48 (m, 4H), 2.37 – 2.14 (m, 2H), 2.08*/2.06 (s, 3H), 2.00 – 1.78 (m, 2H), 1.77 – 1.50 (m, 2H).
^{13}C NMR (101 MHz, CDCl$_3$) δ 197.1, 173.9, 172.5, 143.2, 141.6, 132.3, 129.6, 128.4, 128.3, 125.9, 118.9, 45.9, 43.5, 36.1, 34.4, 33.6, 31.5, 26.4, 21.9.
HRMS (ESI-TOF) m/z: [M + H]+ Calcd for $C_{23}H_{29}N_2O_3$ 398.1962; Found: 398.1968.

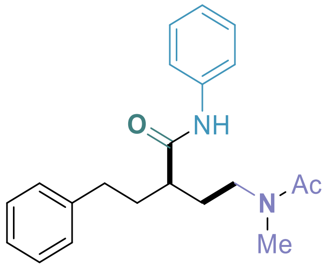

4-(*N*-methylacetamido)-2-phenethyl-*N*-phenylbutanamide (7h)

This reaction was conducted on a 0.3 mmol scale with the general procedure I. The crude product was purified by silica gel chromatography (EA/MeOH = 20:1) to afford the title compound as a colorless oil (73.0 mg, 72% yield). The identity of the product was confirmed by ^{1}H NMR, ^{13}C NMR, and HRMS.

^{1}H NMR (400 MHz, CDCl$_3$) δ 9.39 (s, 1H), 7.64 (d, J = 7.6 Hz, 2H), 7.27 – 7.16 (m, 4H), 7.13 – 7.06 (m, 3H), 7.00 (t, J = 7.4 Hz, 1H), 4.04 – 3.85 (m, 1H), 2.99 – 2.74 (m, 4H), 2.70 – 2.58 (m, 1H), 2.58 – 2.42 (m, 1H), 2.28 – 2.06 (m, 2H), 1.95 (s, 3H), 1.88 – 1.76 (m, 1H), 1.62 – 1.46 (m, 2H).
^{13}C NMR (101 MHz, CDCl$_3$) δ 173.4, 172.3, 141.8, 138.8, 128.9, 128.5, 128.4, 125.9, 123.7, 119.7, 46.0, 43.7, 36.1, 34.7, 33.7, 31.4, 21.9.
HRMS (ESI-TOF) m/z: [M + H]+ Calcd for $C_{21}H_{27}N_2O_2$ 339.2067; Found: 339.2071.

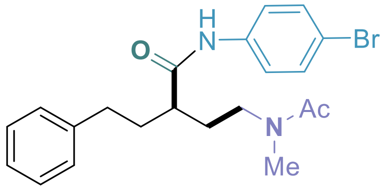

***N*-(4-bromophenyl)-4-(*N*-methylacetamido)-2-phenethylbutanamide (7i)**

This reaction was conducted on a 0.3 mmol scale with the general procedure I. The crude product was purified by silica gel chromatography (EA/MeOH = 20:1) to afford the title compound as a colorless oil (94.5 mg, 76% yield). The identity of the product was confirmed by ^{1}H NMR, ^{13}C NMR, and HRMS.

^{1}H NMR (400 MHz, CDCl$_3$) δ 9.75 (s, 1H), 7.63 (d, J = 8.8 Hz, 2H), 7.41 (d, J = 8.9 Hz, 2H), 7.33 – 7.23 (m, 2H), 7.22 – 7.09 (m, 3H), 4.13 – 3.98 (m, 1H), 3.03 – 2.78 (m, 4H), 2.74 – 2.51 (m, 2H), 2.31 – 2.15 (m, 1H), 2.04 (s, 3H), 1.94 – 1.79 (m, 2H), 1.72 – 1.55 (m, 2H).
^{13}C NMR (101 MHz, CDCl$_3$) δ 173.4, 172.4, 141.6, 137.9, 131.7, 128.4, 128.4, 128.3, 125.9, 121.2, 116.0, 45.9, 43.4, 36.1, 34.4, 33.6, 31.4, 21.9.
HRMS (ESI-TOF) m/z: [M + H]+ Calcd for $C_{21}H_{26}BrN_2O_2$ 417.1172; Found: 417.1179.

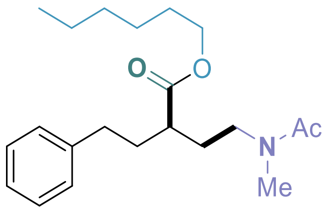

hexyl-4-(*N*-methylacetamido)-2-phenethylbutanoate (7j)

This reaction was conducted on a 0.3 mmol scale with the general procedure I. The crude product was purified by silica gel chromatography (PE/EA = 1:1) to afford the title compound as a colorless oil (85.4 mg, 82% yield). The identity of the product was confirmed by ^{1}H NMR, ^{13}C NMR, and HRMS. Analysis of NMR spectra revealed the presence of two amide bond rotamers. These conformational isomers have been observed and studied in similar compounds.[1] The minor distinguishable rotamer peak is denoted with *.

^{1}H NMR (400 MHz, CDCl$_3$) δ 7.31 – 7.23 (m, 2H), 7.24 – 7.13 (m, 3H), 4.31 – 4.04 (m, 2H), 3.51 – 3.19 (m, 2H), 2.95/2.88* (s, 3H), 2.79 – 2.50 (m, 2H), 2.47 – 2.32 (m, 1H), 2.06*/2.05 (s, 3H), 1.94 – 1.58 (m, 6H), 1.41 – 1.27 (m, 6H), 0.89 (t, J = 6.0 Hz, 3H).
^{13}C NMR (101 MHz, CDCl$_3$) δ 175.6/175.1*, 170.4/170.3*, 141.4/141.0*, 128.5*/128.4, 128.3, 126.1*/125.9, 64.9*/64.6, 49.1*/48.8, 42.7/42.5*, 36.2/34.3*, 34.1/33.4*, 33.4*/33.1, 31.4/31.4*, 30.6/29.4*, 28.6*/28.5, 25.6/25.5*, 22.5/22.5*, 21.8/21.3*, 21.1*/14.0.
HRMS (ESI-TOF) m/z: [M + H]+ Calcd for $C_{21}H_{34}NO_3$ 348.2533; Found: 348.2533.

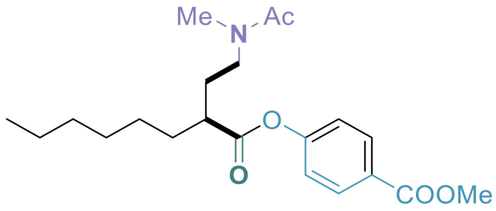

methyl 4-((2-(2-(*N*-methylacetamido)ethyl)octanoyl)oxy)benzoate (7k)

This reaction was conducted on a 0.3 mmol scale with the general procedure I. The crude product was purified by silica gel chromatography (PE/EA = 1:2) to afford the title compound as a colorless oil (71.3 mg, 63% yield). The identity of the product was confirmed by ^{1}H NMR, ^{13}C NMR, and HRMS. Analysis of NMR spectra revealed the presence of two amide bond rotamers. These conformational isomers have been observed and studied in similar compounds.[1] The minor distinguishable rotamer peak is denoted with *.

^{1}H NMR (400 MHz, CDCl$_3$) δ 8.06 (dd, J = 8.5, 6.0 Hz, 2H), 7.15 (dd, J = 18.7, 8.7 Hz, 2H), 3.90/3.90* (s, 3H), 3.59 – 3.27 (m, 2H), 2.98/2.92* (s, 3H), 2.75 – 2.52 (m, 1H), 2.19 – 1.95 (m, 4H), 1.91 – 1.71 (m, 2H), 1.71 – 1.59 (m, 1H), 1.48 – 1.05 (m, 8H), 0.87 (t, J = 3.6 Hz, 3H).

^{13}C NMR (101 MHz, CDCl$_3$) δ 173.8/173.4*, 170.6/170.3*, 166.3/166.1*, 154.4/154.0*, 131.2*/131.1, 127.9*/127.6, 121.6/121.4*, 52.2*/52.1, 48.9*/45.6, 43.2/43.0*, 36.1*/33.2, 32.6*/32.5, 31.6*/31.5, 30.2*/29.2, 29.1/29.1*, 27.1, 22.5/22.5*, 21.9/21.2*, 14.0/14.0*.

HRMS (ESI-TOF) m/z: [M + H]+ Calcd for $C_{21}H_{32}NO_5$ 378.2275; Found: 378.2279.

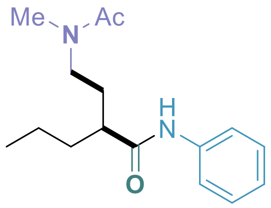

2-(2-(*N*-methylacetamido)ethyl)-*N*-phenylpentanamide (7l)

This reaction was conducted on a 0.3 mmol scale with the general procedure I. The crude product was purified by silica gel chromatography (EA/MeOH = 20:1) to afford the title compound as a colorless oil (61.3 mg, 74% yield). The identity of the product was confirmed by ^{1}H NMR, ^{13}C NMR, and HRMS. Analysis of NMR spectra revealed the presence of two amide bond rotamers. These conformational isomers have been observed and studied in similar compounds.[1] The minor distinguishable rotamer peak is denoted with *.

^{1}H NMR (400 MHz, CDCl$_3$) δ 9.35 (s, 1H), 7.69 (d, J = 7.4 Hz, 2H), 7.51 – 7.19 (m, 2H), 7.05 (t, J = 7.4 Hz, 1H), 4.18 – 3.60 (m, 1H), 3.42 – 2.77 (m, 4H), 2.27 – 2.15 (m, 1H), 2.10/2.05* (s, 3H), 1.90 – 1.75 (m, 2H), 1.69 – 1.57 (m, 1H), 1.40 – 1.21 (m, 3H), 0.90 (t, J = 7.1 Hz, 3H).

^{13}C NMR (101 MHz, CDCl$_3$) δ 173.8, 172.1, 138.7, 128.7, 123.5, 119.6, 46.0, 44.6, 36.1, 35.4, 31.3, 22.0, 20.7, 14.0.

HRMS (ESI-TOF) m/z: [M + H]+ Calcd for $C_{16}H_{25}N_2O_2$ 277.1911; Found: 277.1920.

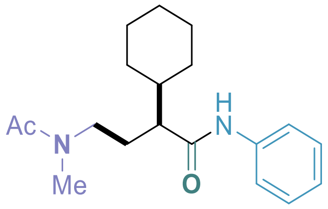

2-cyclohexyl-4-(*N*-methylacetamido)-*N*-phenylbutanamide (7m)

This reaction was conducted on a 0.3 mmol scale with the general procedure I. The crude product was purified by silica gel chromatography (EA/MeOH = 20:1) to afford the title compound as a colorless oil (50.2 mg, 53% yield). The identity of the product was confirmed by ^{1}H NMR, ^{13}C NMR, and HRMS. Analysis of NMR spectra revealed the presence of two amide bond rotamers. These conformational isomers have been observed and studied in similar compounds.[1] The minor distinguishable rotamer peak is denoted with *.

^{1}H NMR (400 MHz, CDCl$_3$) δ 8.87 (s, 1H), 7.66 (d, J = 7.1 Hz, 2H), 7.38 – 7.19 (m, 2H), 7.06 (t, J = 7.4 Hz, 1H), 3.94 – 3.63 (m, 1H), 3.16 – 3.03 (m, 1H), 2.97/2.91* (s, 3H), 2.05 (d, J = 4.8 Hz, 4H), 1.97 – 1.77 (m, 4H), 1.75 – 1.58 (m, 4H), 1.38 – 1.08 (m, 3H), 1.07 – 0.95 (m, 1H), 0.95 – 0.81 (m, 1H).

^{13}C NMR (101 MHz, CDCl$_3$) δ 173.8, 172.0, 138.5, 128.8, 123.6, 119.7, 51.6, 46.2, 39.7, 36.0, 31.2, 30.6, 27.3, 26.4, 26.3, 26.2, 22.0.

HRMS (ESI-TOF) m/z: [M + H]+ Calcd for $C_{19}H_{29}N_2O_2$ 317.2224; Found: 317.2226.

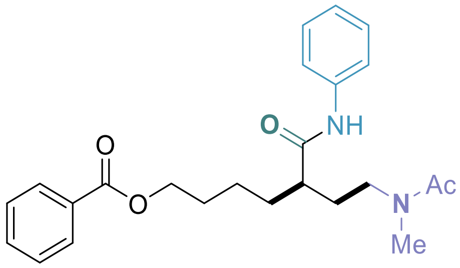

7-(*N*-methylacetamido)-5-(phenylcarbamoyl)heptyl benzoate (7n)

This reaction was conducted on a 0.3 mmol scale with the general procedure I. The crude product was purified by silica gel chromatography (EA/MeOH = 20:1) to afford the title compound as a colorless oil (83.6 mg, 68% yield). The identity of the product was confirmed by ^{1}H NMR, ^{13}C NMR, and HRMS. Analysis of NMR spectra revealed the presence of two amide bond rotamers. These conformational isomers have been

observed and studied in similar compounds.[1] The minor distinguishable rotamer peak is denoted with *.

^{1}H NMR (400 MHz, CDCl$_3$) δ 9.51 (s, 1H), 8.02 (dd, J = 8.4, 1.4 Hz, 2H), 7.69 (dd, J = 8.6, 1.2 Hz, 2H), 7.53 (t, J = 7.4 Hz, 1H), 7.40 (t, J = 7.7 Hz, 2H), 7.34 – 7.27 (m, 2H), 7.06 (t, J = 7.4 Hz, 1H), 4.38 – 4.19 (m, 2H), 4.10 – 3.91 (m, 1H), 3.06 – 2.84 (m, 4H), 2.32 – 2.16 (m, 1H), 2.11 (s, 3H), 1.97 – 1.84 (m, 2H), 1.82 – 1.72 (m, 2H), 1.68 – 1.58 (m, 1H), 1.57 – 1.27 (m, 3H).

^{13}C NMR (101 MHz, CDCl$_3$) δ 173.4, 172.2, 166.6, 138.7, 132.8, 130.3, 129.5, 128.7, 128.3, 128.3, 123.6, 119.6, 64.7, 46.0, 44.6, 36.1, 32.8, 31.5, 28.7, 24.0, 22.0.

HRMS (ESI-TOF) m/z: [M + H]+ Calcd for $C_{24}H_{31}N_2O_4$ 411.2278; Found: 411.2284.

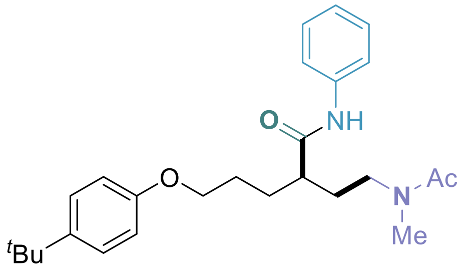

5-(4-(*tert*-butyl)phenoxy)-2-(2-(*N*-methylacetamido)ethyl)-*N*-phenylpentanamide (7o)

This reaction was conducted on a 0.3 mmol scale with the general procedure I. The crude product was purified by silica gel chromatography (EA/MeOH = 20:1) to afford the title compound as a colorless oil (80.1 mg, 63% yield). The identity of the product was confirmed by ^{1}H NMR, ^{13}C NMR, and HRMS. Analysis of NMR spectra revealed the presence of two amide bond rotamers. These conformational isomers have been observed and studied in similar compounds.[1] The minor distinguishable rotamer peak is denoted with *.

^{1}H NMR (400 MHz, CDCl$_3$) δ 9.57 (s, 1H), 7.70 (d, J = 7.4 Hz, 2H), 7.39 – 7.19 (m, 4H), 7.06 (t, J = 7.4 Hz, 1H), 6.82 (d, J = 8.8 Hz, 2H), 4.22 – 3.87 (m, 3H), 3.00/2.98* (s, 3H), 2.96 – 2.88 (m, 1H), 2.38 – 2.25 (m, 1H), 2.08/2.07* (s, 3H), 2.01 – 1.76 (m, 2H), 1.87 – 1.72 (m, 2H), 1.72 – 1.53 (m, 2H), 1.29 (s, 9H).

^{13}C NMR (101 MHz, CDCl$_3$) δ 173.4, 172.2, 156.6, 143.2, 138.7, 128.8, 126.2, 123.6, 119.6, 113.8, 67.7, 46.0, 44.2, 38.0, 36.1, 35.1, 34.0, 31.6, 31.5, 30.0, 27.2, 21.9, 21.5.

HRMS (ESI-TOF) m/z: [M + H]+ Calcd for $C_{26}H_{37}N_2O_3$ 425.2799; Found: 425.2807.

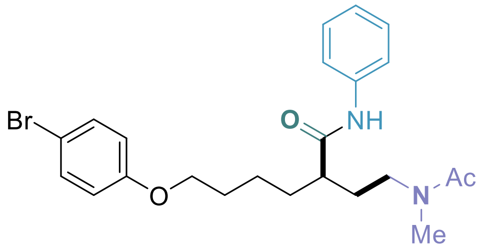

6-(4-bromophenoxy)-2-(2-(*N*-methylacetamido)ethyl)-*N*-phenylhexanamide (7p)

This reaction was conducted on a 0.3 mmol scale with the general procedure I. The crude product was purified by silica gel chromatography (EA/MeOH = 20:1) to afford the title compound as a colorless oil (80.0 mg, 58% yield). The identity of the product was confirmed by ^{1}H NMR, ^{13}C NMR, and HRMS. Analysis of NMR spectra revealed the presence of two amide bond rotamers. These conformational isomers have been observed and studied in similar compounds.[1] The minor distinguishable rotamer peak is denoted with *.

^{1}H NMR (400 MHz, CDCl$_3$) δ 9.47 (s, 1H), 7.69 (dd, J = 8.6, 1.2 Hz, 2H), 7.49 – 7.18 (m, 4H), 7.06 (t, J = 7.4 Hz, 1H), 6.88 – 6.64 (m, 2H), 4.19 – 3.95 (m, 1H), 4.01 – 3.72 (m, 2H), 3.00 (s, 3H), 2.96 – 2.84 (m, 1H), 2.29 – 2.16 (m, 1H), 2.12/2.08* (s, 3H), 2.00 – 1.83 (m, 2H), 1.82 – 1.73 (m, 2H), 1.73 – 1.59 (m, 1H), 1.54 – 1.43 (m, 2H), 1.42 – 1.32 (m, 1H).

^{13}C NMR (101 MHz, CDCl$_3$) δ 173.5, 172.3, 158.1, 138.7, 132.2, 128.8, 123.7, 119.6, 116.3, 112.6, 67.9, 46.1, 44.8, 36.2, 33.0, 31.6, 29.2, 24.1, 22.1.

HRMS (ESI-TOF) m/z: [M + H]+ Calcd for $C_{23}H_{30}BrN_2O_3$ 461.1434; Found: 461.1438.

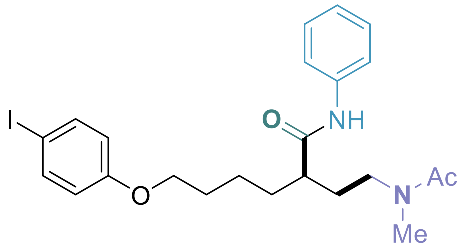

6-(4-iodophenoxy)-2-(2-(*N*-methylacetamido)ethyl)-*N*-phenylhexanamide (7q)

This reaction was conducted on a 0.3 mmol scale with the general procedure I. The crude product was purified by silica gel chromatography (EA/MeOH = 20:1) to afford the title compound as a colorless oil (91.4 mg, 60% yield). The identity of the product was confirmed by ^{1}H NMR, ^{13}C NMR, and HRMS. Analysis of NMR spectra revealed the presence of two amide bond rotamers. These conformational isomers have been

observed and studied in similar compounds.[1] The minor distinguishable rotamer peak is denoted with *.

^{1}H NMR (400 MHz, CDCl$_3$) δ 9.47 (s, 1H), 7.81 – 7.65 (m, 2H), 7.52 (d, J = 8.9 Hz, 2H), 7.43 – 7.27 (m, 2H), 7.06 (t, J = 7.4 Hz, 1H), 6.64 (d, J = 8.9 Hz, 2H), 4.11 – 3.96 (m, 1H), 4.01 – 3.79 (m, 2H), 3.09 – 2.82 (m, 4H), 2.23 – 2.15 (m, 1H), 2.12/2.08* (s, 3H), 1.96 – 1.84 (m, 2H), 1.80 – 1.70 (m, 2H), 1.69 – 1.58 (m, 1H), 1.54 – 1.43 (m, 2H), 1.43 – 1.32 (m, 1H).

^{13}C NMR (101 MHz, CDCl$_3$) δ 173.5, 172.3, 158.9, 138.7, 138.1, 128.8, 123.6, 119.6, 116.9, 82.4, 67.8, 46.1, 44.8, 36.2, 33.0, 31.6, 29.2, 24.1, 22.1.

HRMS (ESI-TOF) m/z: [M + H]+ Calcd for $C_{23}H_{30}IN_2O_3$ 509.1296; Found: 509.1297.

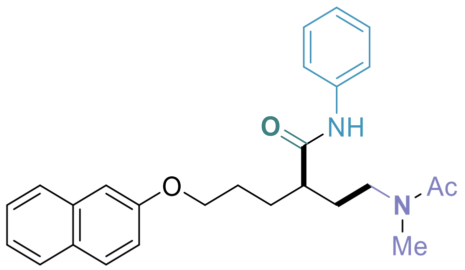

2-(2-(*N*-methylacetamido)ethyl)-5-(naphthalen-2-yloxy)-*N*-phenylpentanamide (7r)

This reaction was conducted on a 0.3 mmol scale with the general procedure I. The crude product was purified by silica gel chromatography (EA/MeOH = 20:1) to afford the title compound as a colorless oil (82.8 mg, 66% yield). The identity of the product was confirmed by ^{1}H NMR, ^{13}C NMR, and HRMS. Analysis of NMR spectra revealed the presence of two amide bond rotamers. These conformational isomers have been observed and studied in similar compounds.[1] The minor distinguishable rotamer peak is denoted with *.

^{1}H NMR (400 MHz, CDCl$_3$) δ 9.49 (s, 1H), 7.88 – 7.61 (m, 5H), 7.45 – 7.37 (m, 1H), 7.36 – 7.27 (m, 3H), 7.15 – 7.01 (m, 3H), 4.14 – 3.95 (m, 3H), 3.11 – 2.83 (m, 4H), 2.33 – 2.17 (m, 1H), 2.10/2.05* (s, 3H), 1.90 – 1.80 (m, 2H), 1.71 – 1.58 (m, 1H), 1.58 – 1.48 (m, 2H), 1.47 – 1.37 (m, 1H).

^{13}C NMR (101 MHz, CDCl$_3$) δ 173.6, 172.2, 156.9, 138.7, 134.5, 129.3, 128.8, 128.8, 127.6, 126.7, 126.3, 123.6, 123.4, 119.6, 118.9, 106.5, 67.6, 46.0, 44.8, 36.1, 33.0, 31.5, 29.3, 24.2, 22.0.

HRMS (ESI-TOF) m/z: [M + H]+ Calcd for $C_{26}H_{31}N_2O_3$ 419.2329; Found: 419.2335.

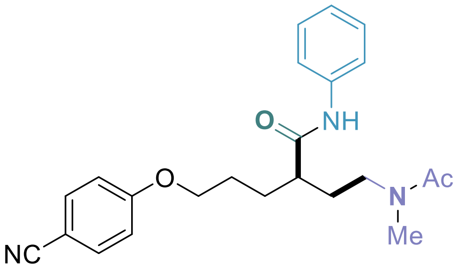

5-(4-cyanophenoxy)-2-(2-(*N*-methylacetamido)ethyl)-*N*-phenylpentanamide (7s)

This reaction was conducted on a 0.3 mmol scale with the general procedure I. The crude product was purified by silica gel chromatography (EA/MeOH = 10:1) to afford the title compound as a colorless oil (61.3 mg, 52% yield). The identity of the product was confirmed by ^{1}H NMR, ^{13}C NMR, and HRMS. Analysis of NMR spectra revealed the presence of two amide bond rotamers. These conformational isomers have been observed and studied in similar compounds.[1] The minor distinguishable rotamer peak is denoted with *.

^{1}H NMR (400 MHz, CDCl$_3$) δ 9.51 (s, 1H), 7.92 – 7.66 (m, 2H), 7.55 (d, J = 8.8 Hz, 2H), 7.30 (t, J = 7.9 Hz, 2H), 7.07 (t, J = 7.3 Hz, 1H), 6.89 (d, J = 8.8 Hz, 2H), 4.18 – 3.79 (m, 3H), 3.08 – 2.72 (m, 4H), 2.32 – 2.17 (m, 1H), 2.12/2.08* (s, 3H), 1.85 – 1.73 (m, 2H), 1.68 – 1.57 (m, 1H), 1.55 – 1.43 (m, 2H), 1.43 – 1.29 (m, 1H).
^{13}C NMR (101 MHz, CDCl$_3$) δ 173.4, 172.3, 162.3, 138.7, 133.9, 128.8, 123.7, 119.6, 119.3, 115.1, 103.6, 68.1, 46.1, 44.8, 36.2, 33.0, 31.7, 29.0, 24.1, 22.0.
HRMS (ESI-TOF) m/z: [M + H]+ Calcd for $C_{23}H_{28}N_3O_3$ 394.2125; Found: 394.2133.

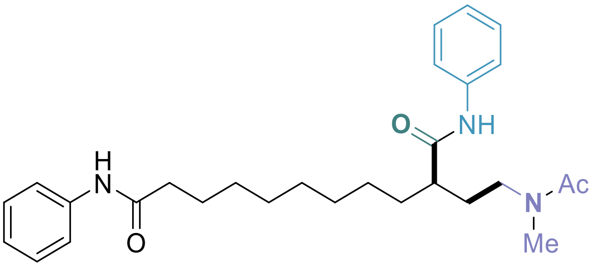

2-(2-(*N*-methylacetamido)ethyl)-*N*1,*N*11-diphenylundecanediamide (7t)

This reaction was conducted on a 0.3 mmol scale with the general procedure I. The crude product was purified by silica gel chromatography (EA/MeOH = 10:1) to afford the title compound as a colorless oil (99.0 mg, 71% yield). The identity of the product was confirmed by ^{1}H NMR, ^{13}C NMR, and HRMS. Analysis of NMR spectra revealed the presence of two amide bond rotamers. These conformational isomers have been

observed and studied in similar compounds.[1] The minor distinguishable rotamer peak is denoted with *.

^{1}H NMR (400 MHz, $CDCl_3$) δ 9.40 (s, 1H), 7.77 (s, 1H), 7.70 – 7.63 (m, 2H), 7.53 (d, J = 7.5 Hz, 2H), 7.37 – 7.25 (m, 4H), 7.16 – 7.01 (m, 2H), 4.10 – 3.91 (m, 1H), 3.13 – 2.84 (m, 4H), 2.33 (t, J = 7.4 Hz, 2H), 2.19 – 2.02 (m, 4H), 1.93 – 1.79 (m, 2H), 1.76 – 1.59 (m, 2H), 1.43 – 1.15 (m, 12H).

^{13}C NMR (101 MHz, $CDCl_3$) δ 173.9, 172.2, 171.7, 138.7, 138.2, 128.8, 128.8, 123.9, 123.7, 119.8, 119.7, 46.0, 44.9, 37.5, 36.1, 33.1, 31.4, 29.3, 28.9, 28.8, 28.8, 27.3, 25.4, 22.0.

HRMS (ESI-TOF) m/z: [M + H]+ Calcd for $C_{28}H_{40}N_3O_3$ 466.3064; Found: 466.3066.

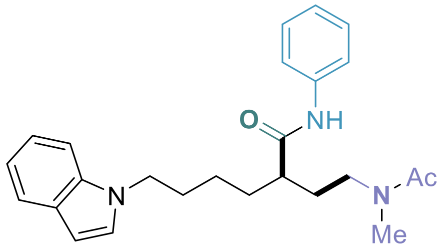

6-(1*H*-indol-1-yl)-2-(2-(*N*-methylacetamido)ethyl)-*N*-phenylhexanamide (7u)

This reaction was conducted on a 0.3 mmol scale with the general procedure I. The crude product was purified by silica gel chromatography (EA/MeOH = 10:1) to afford the title compound as a colorless oil (66.8 mg, 55% yield). The identity of the product was confirmed by ^{1}H NMR, ^{13}C NMR, and HRMS. Analysis of NMR spectra revealed the presence of two amide bond rotamers. These conformational isomers have been observed and studied in similar compounds.[1] The minor distinguishable rotamer peak is denoted with *.

^{1}H NMR (400 MHz, $CDCl_3$) δ 9.51 (s, 1H), 7.89 – 7.58 (m, 3H), 7.43 – 7.26 (m, 3H), 7.22 – 7.13 (m, 1H), 7.15 – 6.86 (m, 3H), 6.46 (dd, J = 3.2, 0.8 Hz, 1H), 4.39 – 3.87 (m, 3H), 3.13 – 2.76 (m, 4H), 2.22 – 1.95 (m, 4H), 1.94 – 1.72 (m, 4H), 1.61 – 1.47 (m, 1H), 1.44 – 1.16 (m, 3H).

^{13}C NMR (101 MHz, $CDCl_3$) δ 173.4, 172.3, 138.7, 135.8, 128.8, 128.5, 127.9, 123.6, 121.3, 120.9, 119.6, 119.1, 109.4, 100.8, 46.2, 45.9, 44.4, 36.0, 32.7, 31.5, 30.3, 25.1, 22.0.

HRMS (ESI-TOF) m/z: [M + H]+ Calcd for $C_{25}H_{32}N_3O_2$ 406.2489; Found: 406.2494.

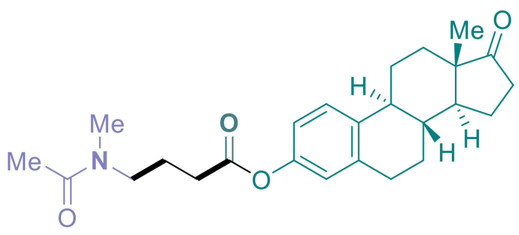

(8R,9S,13S,14S)-13-methyl-17-oxo-7,8,9,11,12,13,14,15,16,17-decahydro-6*H*-cyclopenta[a]phenanthren-3-yl 4-(*N*-methylacetamido)butanoate (8a)

This reaction was conducted on a 0.3 mmol scale with the general procedure II. The crude product was purified by silica gel chromatography (PE/EA = 1:2) to afford the title compound as a colorless oil (95.9 mg, 77% yield). The identity of the product was confirmed by ^{1}H NMR, ^{13}C NMR, and HRMS. Analysis of NMR spectra revealed the presence of two amide bond rotamers. These conformational isomers have been observed and studied in similar compounds.[1] The minor distinguishable rotamer peak is denoted with *.

^{1}H NMR (400 MHz, CDCl$_3$) δ 7.28 (t, J = 7.6 Hz, 1H), 6.97 – 6.76 (m, 2H), 3.48/3.41* (t, J = 7.2 Hz, 2H), 3.02/2.95* (s, 3H), 2.94 – 2.84 (m, 2H), 2.72 – 2.44 (m, 3H), 2.44 – 2.32 (m, 1H), 2.32 – 2.22 (m, 2H), 2.21 – 2.06 (m, 4H), 2.06 – 1.89 (m, 4H), 1.73 – 1.34 (m, 6H), 0.93*/0.90 (s, 3H).
^{13}C NMR (101 MHz, CDCl$_3$) δ 220.6/220.5*, 171.9/171.4*, 170.6/170.4*, 148.4/148.2*, 138.0*/137.8, 137.4*/137.2, 126.3*/126.2, 121.4/121.2*, 118.6/118.4*, 50.2/49.6*, 47.7/47.6*, 46.5/46.4*, 44.0, 37.8*/37.8, 36.0*/35.7, 33.0*/31.4, 30.7*/29.2, 26.1/26.1*, 25.6/23.1*, 22.3*/21.7, 21.4/21.1*, 14.3*/13.6.
HRMS (ESI-TOF) m/z: [M + H]+ Calcd for $C_{25}H_{34}NO_4$ 412.2482; Found: 412.2490.

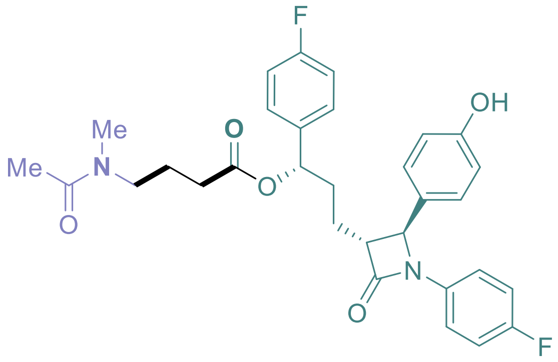

(S)-1-(4-fluorophenyl)-3-((2S,3R)-1-(4-fluorophenyl)-2-(4-hydroxyphenyl)-4-oxoazetidin-3-yl)propyl 4-(*N*-methylacetamido)butanoate (8b)

This reaction was conducted on a 0.3 mmol scale with the general procedure II. The crude product was purified by silica gel chromatography (PE/EA = 1:2) to afford the

title compound as a colorless oil (104.0 mg, 63% yield). The identity of the product was confirmed by ^{1}H NMR, ^{13}C NMR, and HRMS. Analysis of NMR spectra revealed the presence of two amide bond rotamers. These conformational isomers have been observed and studied in similar compounds.[1] The minor distinguishable rotamer peak is denoted with *.

^{1}H NMR (400 MHz, CDCl$_3$) δ 7.85*/7.82 (s, 1H), 7.51 – 7.39 (m, 2H), 7.40 – 7.32 (m, 2H), 7.28 – 7.18 (m, 2H), 7.07 – 6.97 (m, 4H), 6.91 (t, J = 8.7 Hz, 2H), 4.87 – 4.74 (m, 1H), 4.60 (d, J = 11.4 Hz, 1H), 3.45/3.38* (t, J = 7.2 Hz, 2H), 3.00/2.99* (s, 3H), 2.92/2.91* (s, 3H), 2.63 – 2.46 (m, 3H), 2.13 – 2.01 (m, 4H), 2.01 – 1.86 (m, 2H).

^{13}C NMR (101 MHz, CDCl$_3$) δ 171.5/170.8*, 171.1/170.7*, 171.1/170.5*, 162.0/159.3* (d, J = 272.7 Hz), 160.5/158.1*, 150.2*/150.0, 138.7/138.4*, 138.2/138.2*, 133.4*/133.4, 127.7*/127.6, 127.3*/127.2, 122.5/122.4*, 121.4/121.3*, 115.3*/115.1, 114.9, 81.1/79.0*, 52.3/52.3*, 49.7*/46.4, 37.9*/36.0, 35.1/33.1*, 32.5*/31.3, 30.8*/27.7, 23.1/22.3*, 21.7, 21.4/21.1*.

^{19}F NMR (376 MHz, CDCl$_3$) δ -115.1*/-115.2, -118.0*/-118.0.

HRMS (ESI-TOF) m/z: [M + H]+ Calcd for $C_{31}H_{33}F_2N_2O_5$ 551.2352; Found: 551.2356.

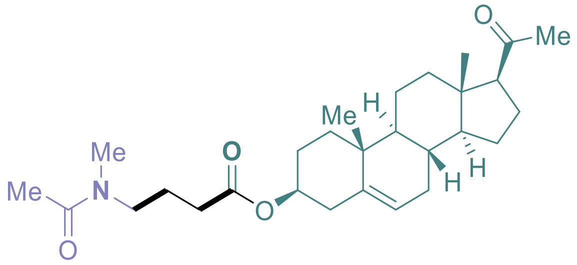

(3S,8S,9S,10R,13S,14S,17S)-17-acetyl-10,13-dimethyl 2,3,4,7,8,9,10,11,12,13,14,15, 16,17-tetradecahydro-1*H* cyclopenta[a]phenanthren-3-yl 4-(*N*-methylacetamido) butanoate (8c)

This reaction was conducted on a 0.3 mmol scale with the general procedure II. The crude product was purified by silica gel chromatography (PE/EA = 1:3) to afford the title compound as a colorless oil (93.2 mg, 68% yield). The identity of the product was confirmed by ^{1}H NMR, ^{13}C NMR, and HRMS. Analysis of NMR spectra revealed the presence of two amide bond rotamers. These conformational isomers have been observed and studied in similar compounds.[1] The minor distinguishable rotamer peak is denoted with *.

^{1}H NMR (400 MHz, CDCl$_3$) δ 5.51 – 5.24 (m, 1H), 5.04 – 4.50 (m, 1H), 4.12 – 3.23 (m, 2H), 3.00/2.92* (s, 3H), 2.54 (t, J = 8.9 Hz, 1H), 2.41 – 2.26 (m, 4H), 2.22 – 1.96

(m, 9H), 1.96 – 1.80 (m, 4H), 1.77 – 1.55 (m, 5H), 1.53 – 1.41 (m, 3H), 1.32 – 1.09 (m, 3H), 1.02 (t, J = 2.7 Hz, 4H), 0.69*/0.63 (s, 3H).

^{13}C NMR (101 MHz, CDCl$_3$) δ 209.5/209.4*, 172.5/172.0*, 170.5/170.4*, 139.6/139.4*, 122.5*/122.3, 74.1*/73.8, 63.6/63.6*, 56.7/56.7*, 49.8/46.7*, 43.9, 38.7*/38.0, 36.9*/36.9, 36.5/36.1*, 33.1, 31.7/31.7*, 31.5/31.1*, 27.7, 24.4/23.3*, 22.7/22.5*, 21.8/21.1*, 20.9/19.2*, 13.1.

HRMS (ESI-TOF) m/z: [M + H]+ Calcd for $C_{28}H_{44}NO_4$ 458.3265; Found: 458.3260.

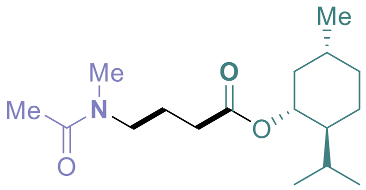

(1R,2S,5R)-2-isopropyl-5-methylcyclohexyl 4-(*N*-methylacetamido)butanoate (8d)

This reaction was conducted on a 0.3 mmol scale with the general procedure II. The crude product was purified by silica gel chromatography (PE/EA = 1:1) to afford the title compound as a colorless oil (75.7 mg, 85% yield). The identity of the product was confirmed by ^{1}H NMR, ^{13}C NMR, and HRMS. Analysis of NMR spectra revealed the presence of two amide bond rotamers. These conformational isomers have been observed and studied in similar compounds.[1] The minor distinguishable rotamer peak is denoted with *.

^{1}H NMR (400 MHz, CDCl$_3$) δ 4.77 – 4.62 (m, 1H), 3.48 – 3.26 (m, 2H), 2.98/2.90* (s, 3H), 2.36 – 2.19 (m, 2H), 2.08*/2.06 (s, 3H), 2.00 – 1.76 (m, 5H), 1.73 – 1.55 (m, 2H), 1.51 – 1.27 (m, 1H), 1.10 – 0.80 (m, 9H), 0.74 (dd, J = 7.0, 3.7 Hz, 3H).

^{13}C NMR (101 MHz, CDCl$_3$) δ 172.8/172.2*, 170.6/170.5*, 74.5*/74.2, 49.8/46.9*, 46.9/46.8*, 40.9*/40.9, 36.1*/34.2, 34.1/33.1*, 31.8/31.1*, 31.3*/26.3, 26.2*/23.4, 23.3/22.6*, 22.0/22.05*, 21.8*/21.1, 20.7/20.7*, 16.2.

HRMS (ESI-TOF) m/z: [M + H]+ Calcd for $C_{17}H_{32}NO_3$ 298.2377; Found: 298.2374.

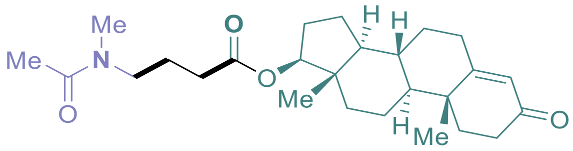

(8R,9S,10R,13S,14S,17S)-10,13-dimethyl-3-oxo-2,3,6,7,8,9,10,11,12,13,14,15,16,17-tetradecahydro-1*H*-cyclopenta[a]phenanthren-17-yl 4-(*N*-methylacetamido) butanoate (8e)

This reaction was conducted on a 0.3 mmol scale with the general procedure II. The crude product was purified by silica gel chromatography (PE/EA = 1:3) to afford the title compound as a colorless oil (105.5 mg, 82% yield). The identity of the product was

confirmed by ^{1}H NMR, ^{13}C NMR, and HRMS. Analysis of NMR spectra revealed the presence of two amide bond rotamers. These conformational isomers have been observed and studied in similar compounds.[1] The minor distinguishable rotamer peak is denoted with *.

^{1}H NMR (400 MHz, $CDCl_3$) δ 5.68 (s, 1H), 4.65 – 4.41 (m, 1H), 3.85 – 3.19 (m, 2H), 2.95/2.87* (s, 3H), 2.40 – 2.19 (m, 6H), 2.19 – 2.08 (m, 1H), 2.05*/2.02 (s, 3H), 2.02 – 1.93 (m, 1H), 1.89 – 1.75 (m, 3H), 1.78 – 1.66 (m, 1H), 1.68 – 1.43 (m, 5H), 1.40 – 1.25 (m, 2H), 1.14 (d, J = 1.5 Hz, 4H), 1.08 – 0.84 (m, 3H), 0.79 (s, 3H).

^{13}C NMR (101 MHz, $CDCl_3$) δ 199.3/199.3*, 173.1/172.5*, 170.9/170.7*, 170.5/170.4*, 123.8*/123.8, 82.7*/82.4, 53.6/53.5*, 50.1/50.1*, 49.7*/46.6, 42.4, 38.5/38.5*, 36.5/36.0*, 35.6/35.2*, 33.8/33.0*, 32.6/32.6*, 31.5/31.3*, 31.3/30.8*, 27.4, 23.3/23.3*, 23.3*/22.5, 21.8*/21.1, 20.4*/17.3, 12.0*/12.0.

HRMS (ESI-TOF) m/z: [M + H]+ Calcd for $C_{26}H_{40}NO_4$ 430.2952; Found: 430.2954.

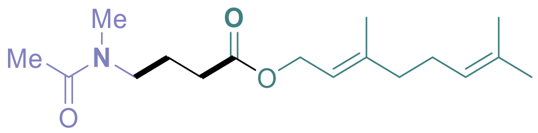

3,7-dimethylocta-2,6-dien-1-yl 4-(*N*-methylacetamido)butanoate (8f)

This reaction was conducted on a 0.3 mmol scale with the general procedure II. The crude product was purified by silica gel chromatography (PE/EA = 1:1) to afford the title compound as a colorless oil (63.7 mg, 72% yield). The identity of the product was confirmed by ^{1}H NMR, ^{13}C NMR, and HRMS. Analysis of NMR spectra revealed the presence of two amide bond rotamers. These conformational isomers have been observed and studied in similar compounds.[1] The minor distinguishable rotamer peak is denoted with *.

^{1}H NMR (400 MHz, $CDCl_3$) δ 5.32 (t, J = 7.0 Hz, 1H), 5.21 – 4.86 (m, 1H), 4.55 (t, J = 7.2 Hz, 2H), 3.68 – 3.21 (m, 2H), 2.96/2.88* (s, 3H), 2.30 (q, J = 7.1 Hz, 2H), 2.14 – 1.97 (m, 7H), 1.95 – 1.78 (m, 2H), 1.73 (s, 3H), 1.65 (s, 3H), 1.57 (s, 3H).

^{13}C NMR (101 MHz, $CDCl_3$) δ 173.1/172.6, 170.5/170.4, 142.9*/142.5, 132.2*/132.1, 123.5/123.4*, 119.1/118.8*, 61.2*/61.0, 49.8*/46.6, 36.0/33.1*, 32.1/31.4*, 30.8, 26.6, 25.6/23.4*, 23.3/22.5*, 21.8/21.1*, 17.6.

HRMS (ESI-TOF) m/z: [M + H]+ Calcd for $C_{17}H_{30}NO_3$ 296.2220; Found: 296.2225.

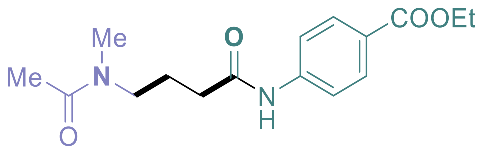

ethyl 4-(4-(*N*-methylacetamido)butanamido)benzoate (8g)

This reaction was conducted on a 0.3 mmol scale with the general procedure II. The crude product was purified by silica gel chromatography (EA/MeOH = 10:1) to afford the title compound as a colorless oil (79.9 mg, 87% yield). The identity of the product was confirmed by ^{1}H NMR, ^{13}C NMR, and HRMS. Analysis of NMR spectra revealed the presence of two amide bond rotamers. These conformational isomers have been observed and studied in similar compounds.[1] The minor distinguishable rotamer peak is denoted with *.

^{1}H NMR (400 MHz, CDCl$_3$) δ 9.81/9.09* (s, 1H), 7.94 (d, J = 8.6 Hz, 2H), 7.70 (d, J = 8.5 Hz, 2H), 4.30 (q, J = 7.1 Hz, 2H), 3.40 (dt, J = 44.8, 6.6 Hz, 2H), 2.98/2.88* (s, 3H), 2.48 – 2.26 (m, 2H), 2.08/2.06* (s, 3H), 2.01 – 1.84 (m, 2H), 1.34 (t, J = 7.1 Hz, 3H).

^{13}C NMR (101 MHz, CDCl$_3$) δ 172.1, 171.6, 166.2, 142.8, 130.5, 130.5, 125.2, 118.7, 60.7*/60.6, 49.7*/46.4, 36.0, 34.5/33.0*, 23.5/23.2*, 21.8/21.1*, 14.2.

HRMS (ESI-TOF) m/z: [M + H]+ Calcd for $C_{16}H_{23}N_2O_4$ 307.1652; Found: 307.1652.

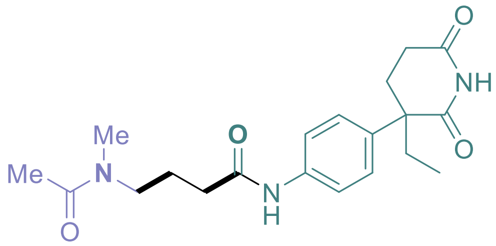

***N*-(4-(3-ethyl-2,6-dioxopiperidin-3-yl)phenyl)-4-(*N*-methylacetamido)butanamide (8h)**

This reaction was conducted on a 0.3 mmol scale with the general procedure II. The crude product was purified by silica gel chromatography (EA/MeOH = 10:1) to afford the title compound as a colorless oil (81.7 mg, 73% yield). The identity of the product was confirmed by ^{1}H NMR, ^{13}C NMR, and HRMS. Analysis of NMR spectra revealed the presence of two amide bond rotamers. These conformational isomers have been

observed and studied in similar compounds.[1] The minor distinguishable rotamer peak is denoted with *.

^{1}H NMR (700 MHz, CDCl$_3$) δ 9.45/8.62* (s, 1H), 8.55/8.36* (s, 1H), 7.62 (d, J = 8.7 Hz, 2H), 7.17 (d, J = 8.8 Hz, 2H), 3.49 – 3.27 (m, 2H), 3.00/2.91* (s, 3H), 2.64 – 2.50 (m, 1H), 2.44 – 2.28 (m, 3H), 2.26 – 2.14 (m, 1H), 2.09 (s, 3H), 2.05 – 1.96 (m, 2H), 1.95 – 1.84 (m, 3H), 0.84 (t, J = 7.5 Hz, 3H).

^{13}C NMR (176 MHz, CDCl$_3$) δ 175.4, 172.6, 172.2, 171.4, 138.0, 133.7, 126.8*/126.6, 120.1*/120.1, 50.6, 46.5, 36.1, 34.5, 32.8, 29.3, 27.0, 23.5, 21.9, 9.0.

HRMS (ESI-TOF) m/z: [M + H]+ Calcd for $C_{20}H_{28}N_3O_4$ 374.2074; Found: 374.2083.

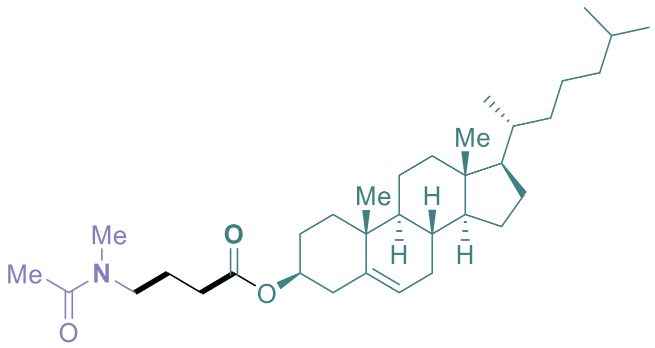

(3S,8S,9S,10R,13R,14S,17R)-10,13-dimethyl-17-((R)-6-methylheptan-2-yl)-2,3,4,7,8,9,10,11,12,13,14,15,16,17-tetradecahydro-1*H*-cyclopenta[a]phenanthren-3-yl 4-(*N*-methylacetamido)butanoate (8i)

This reaction was conducted on a 0.3 mmol scale with the general procedure II. The crude product was purified by silica gel chromatography (PE/EA = 1:1) to afford the title compound as a colorless oil (112.3 mg, 71% yield). The identity of the product was confirmed by ^{1}H NMR, ^{13}C NMR, and HRMS. Analysis of NMR spectra revealed the presence of two amide bond rotamers. These conformational isomers have been observed and studied in similar compounds.[1] The minor distinguishable rotamer peak is denoted with *.

^{1}H NMR (700 MHz, CDCl$_3$) δ 5.45 – 5.25 (m, 1H), 5.00 – 4.37 (m, 1H), 3.46 – 3.25 (m, 2H), 2.98/2.91* (s, 3H), 2.40 – 2.24 (m, 4H), 2.09/2.06* (s, 3H), 2.02 – 1.96 (m, 1H), 1.93 – 1.78 (m, 7H), 1.62 – 1.49 (m, 4H), 1.44/1.43* (s, 3H), 1.39 – 1.25 (m, 4H), 1.18 – 1.05 (m, 7H), 1.05 – 0.95 (m, 5H), 0.91/0.90* (s, 3H), 0.85 (dd, J = 6.6, 3.2 Hz, 6H), 0.66 (s, 3H).

^{13}C NMR (176 MHz, CDCl$_3$) δ 172.6/172.0*, 170.6*/170.5, 139.6/139.4*, 122.8*/122.6, 74.3*/74.0, 56.7*/56.6, 56.1*/50.0, 49.8*/46.7, 42.3/39.7*, 39.7*/39.5, 38.1, 36.9/36.9*,

36.6*/36.1, 36.1*/35.8, 33.1, 31.9/31.8*, 31.8/31.1*, 28.2*/28.1, 28.0, 27.8/27.8*, 24.2*/23.8, 23.4*/22.8, 22.5/21.9*, 21.2*/21.0, 19.3/19.3*, 18.7, 11.8.
HRMS (ESI-TOF) m/z: [M + H]+ Calcd for $C_{34}H_{58}NO_3$ 528.4411; Found: 528.4409.

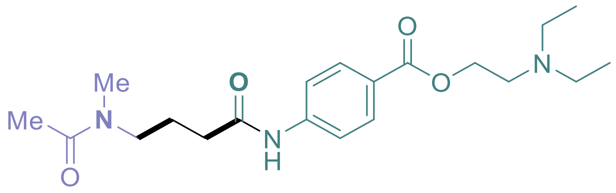

2-(diethylamino)ethyl 4-(4-(*N*-methylacetamido)butanamido)benzoate (8j)

This reaction was conducted on a 0.3 mmol scale with the general procedure II. The crude product was purified by silica gel chromatography (EA/MeOH = 10:1) to afford the title compound as a colorless oil (57.7 mg, 51% yield). The identity of the product was confirmed by ^{1}H NMR, ^{13}C NMR, and HRMS. Analysis of NMR spectra revealed the presence of two amide bond rotamers. These conformational isomers have been observed and studied in similar compounds.[1] The minor distinguishable rotamer peak is denoted with *.

^{1}H NMR (400 MHz, $CDCl_3$) δ 7.78 (d, J = 7.0 Hz, 2H), 6.60 (d, J = 8.7 Hz, 2H), 4.46 – 4.26 (m, 2H), 3.69 – 3.53 (m, 2H), 3.48 – 3.17 (m, 5H), 3.00 – 2.79 (m, 4H), 2.79 – 2.58 (m, 1H), 2.40 – 2.24 (m, 2H), 2.16 – 1.96 (m, 4H), 1.96 – 1.74 (m, 2H), 1.44 – 1.01 (m, 5H).
^{13}C NMR (101 MHz, $CDCl_3$) δ 172.2/172.0*, 171.4*/170.6, 166.4/166.2*, 151.2*/151.1, 131.6*/131.6, 119.1/118.9*, 113.7*/113.6, 62.1/61.5*, 47.3*/46.7, 44.7/ 44.7*, 43.5/43.3*, 35.9/33.0*, 29.9/29.0*, 23.3*/22.6, 21.8/21.1*, 14.1/11.2*.
HRMS (ESI-TOF) m/z: [M + H]+ Calcd for $C_{20}H_{32}N_3O_4$ 378.2387; Found: 378.2381.

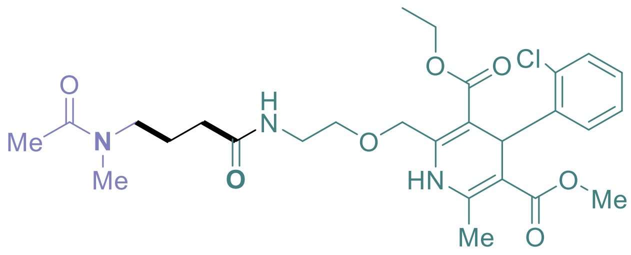

3-ethyl 5-methyl 4-(2-chlorophenyl)-6-methyl-2-((2-(4-(*N*-methylacetamido) butanamido)ethoxy)methyl)-1,4-dihydropyridine-3,5-dicarboxylate (8k)

This reaction was conducted on a 0.3 mmol scale with the general procedure II. The crude product was purified by silica gel chromatography (EA/MeOH = 8:1) to afford

the title compound as a colorless oil (88.9 mg, 54% yield). The identity of the product was confirmed by ^{1}H NMR, ^{13}C NMR, and HRMS. Analysis of NMR spectra revealed the presence of two amide bond rotamers. These conformational isomers have been observed and studied in similar compounds.[1] The minor distinguishable rotamer peak is denoted with *.

^{1}H NMR (700 MHz, CDCl$_3$) δ 7.72 (s, 1H), 7.58 (s, 1H), 7.38 (d, J = 7.8 Hz, 1H), 7.22 (d, J = 6.6 Hz, 1H), 7.12 (t, J = 6.8 Hz, 1H), 7.04 (d, J = 5.9 Hz, 1H), 5.40 (s, 1H), 4.77 (d, J = 15.7 Hz, 1H), 4.66 (d, J = 15.7 Hz, 1H), 4.04 (p, J = 7.1 Hz, 2H), 3.74 – 3.56 (m, 5H), 3.51 – 3.40 (m, 3H), 3.14 – 2.85 (m, 4H), 2.37/2.36* (s, 3H), 2.26 – 2.17 (m, 2H), 2.12/2.09* (s, 3H), 1.98 – 1.81 (m, 2H), 1.18 (t, J = 7.1 Hz, 3H).

^{13}C NMR (176 MHz, CDCl$_3$) δ 173.3, 172.0, 168.2, 167.2, 145.9, 145.4, 144.8, 132.3, 131.5/131.5*, 129.2, 127.4*/127.3, 126.8*/126.8, 103.5, 101.4, 70.5, 68.0, 59.8, 50.7, 46.3, 39.2, 37.1, 36.1, 33.3, 23.6, 21.9, 19.2, 14.3.

HRMS (ESI-TOF) m/z: [M + H]+ Calcd for $C_{27}H_{37}ClN_3O_7$ 550.2315; Found: 550.2321.

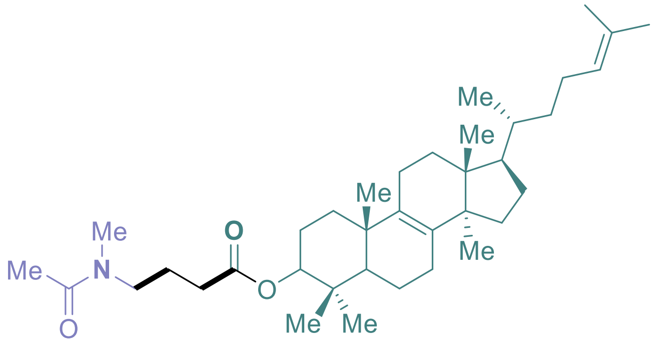

(10S,13R,14R,17R)-4,4,10,13,14-pentamethyl-17-((R)-6-methylhept-5-en-2-yl)-2,3,4,5,6,7,10,11,12,13,14,15,16,17-tetradecahydro-1*H*-cyclopenta[a]phenanthren-3-yl 4-(*N*-methylacetamido)butanoate (8l)

This reaction was conducted on a 0.3 mmol scale with the general procedure II. The crude product was purified by silica gel chromatography (PE/EA = 1:1) to afford the title compound as a colorless oil (79.9 mg, 47% yield). The identity of the product was confirmed by ^{1}H NMR, ^{13}C NMR, and HRMS. Analysis of NMR spectra revealed the presence of two amide bond rotamers. These conformational isomers have been observed and studied in similar compounds.[1] The minor distinguishable rotamer peak is denoted with *.

^{1}H NMR (400 MHz, CDCl$_3$) δ 5.44 – 5.20 (m, 1H), 5.13 – 4.91 (m, 1H), 4.64 – 4.27 (m, 1H), 3.50 – 3.14 (m, 2H), 2.93/2.85* (s, 3H), 2.44 – 2.20 (m, 2H), 2.09 – 1.87 (m,

8H), 1.81 (dt, J = 15.0, 7.6 Hz, 3H), 1.72 – 1.51 (m, 10H), 1.46 – 1.16 (m, 6H), 1.14 – 0.99 (m, 3H), 0.99 – 0.88 (m, 5H), 0.88 – 0.76 (m, 13H), 0.62/0.49* (s, 3H).
^{13}C NMR (101 MHz, CDCl$_3$) δ 173.0/172.4*, 170.6/170.5*, 134.6, 134.2, 130.9, 125.2, 81.4*/81.0, 50.5, 50.4*/50.3, 49.9*/49.8, 49.2*/46.8, 44.4/43.7*, 39.5, 37.8/37.6*, 37.2*/36.9, 36.4*/36.3, 36.2/36.1*, 35.2, 33.1, 31.9, 31.5*/31.2, 30.9*/30.8, 28.2*/28.0, 27.9/27.9*, 26.3*/5.7, 25.5*/4.9, 24.2/4.1*, 23.5*/2.8, 22.7*/2.7, 22.5*/1.9, 21.2*/1.0, 19.2/18.7*, 18.6*/18.4, 18.1*/17.6, 17.0*/16.6, 15.7/15.6*.
HRMS (ESI-TOF) m/z: [M + H]+ Calcd for $C_{37}H_{62}NO_3$ 568.4724; Found: 568.1337

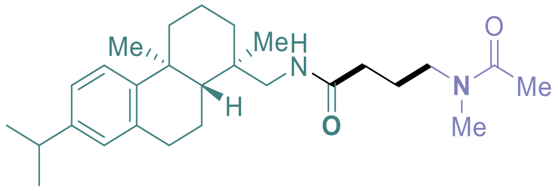

N-(((1R,4aS,10aR)-7-isopropyl-1,4a-dimethyl-1,2,3,4,4a,9,10,10a-octahydro phenanthren-1-yl)methyl)-4-(*N*-methylacetamido)butanamide (8m)

This reaction was conducted on a 0.3 mmol scale with the general procedure II. The crude product was purified by silica gel chromatography (EA/MeOH = 20:1) to afford the title compound as a colorless oil (80.5 mg, 63% yield). The identity of the product was confirmed by ^{1}H NMR, ^{13}C NMR, and HRMS. Analysis of NMR spectra revealed the presence of two amide bond rotamers. These conformational isomers have been observed and studied in similar compounds.[1] The minor distinguishable rotamer peak is denoted with *.

^{1}H NMR (400 MHz, CDCl$_3$) δ 7.14 (d, J = 8.2 Hz, 1H), 7.08 – 6.60 (m, 2H), 3.60 – 3.24 (m, 4H), 3.07 (dd, J = 13.6, 6.3 Hz, 1H), 2.99 (s, 1H), 2.94/2.91* (s, 3H), 2.88 – 2.75 (m, 2H), 2.44 – 2.25 (m, 2H), 2.22 – 2.12 (m, 2H), 2.11 – 2.01 (m, 5H), 1.96 – 1.73 (m, 4H), 1.56 – 1.29 (m, 4H), 1.25 – 1.12 (m, 8H), 0.92 (s, 3H).
^{13}C NMR (101 MHz, CDCl$_3$) δ 175.6*/173.0, 171.6/171.3*, 147.2, 147.0/145.4*, 134.9, 126.8, 124.1, 123.8*/123.7, 50.0/49.9*, 46.7*/46.5, 45.2, 38.3, 37.4/37.3*, 36.1*/36.0, 36.0*/33.5, 33.4/33.2*, 33.1/32.6*, 31.3/30.7*, 30.1, 25.3/25.2*, 23.9*/23.9, 23.4/23.3*, 21.8/21.7*, 21.1*/21.0, 18.9/18.7*, 18.6/18.5*.
HRMS (ESI-TOF) m/z: [M + H]+ Calcd for $C_{27}H_{43}N_2O_2$ 427.3319; Found: 427.3320

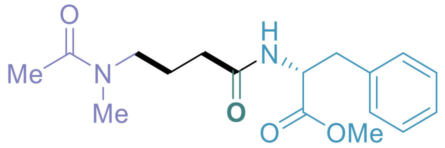

methyl (4-(*N*-methylacetamido)butanoyl)-D-phenylalaninate (9a)

This reaction was conducted on a 0.3 mmol scale with the general procedure II. The crude product was purified by silica gel chromatography (EA/MeOH = 20:1) to afford the title compound as a colorless oil (69.1 mg, 72% yield). The identity of the product was confirmed by ^{1}H NMR, ^{13}C NMR, and HRMS. Analysis of NMR spectra revealed the presence of two amide bond rotamers. These conformational isomers have been observed and studied in similar compounds.[1] The minor distinguishable rotamer peak is denoted with *.

^{1}H NMR (700 MHz, CDCl$_3$) δ 7.32 – 7.25 (m, 2H), 7.26 – 7.21 (m, 1H), 7.16 (d, J = 6.8 Hz, 1H), 7.13 – 7.04 (m, 1H), 4.93 – 4.76 (m, 1H), 3.73*/(s, 3H), 3.47 – 3.29 (m, 2H), 3.27 – 3.13 (m, 2H), 3.05 (dd, J = 14.0, 7.5 Hz, 1H), 2.96/2.88* (s, 3H), 2.24 – 2.15 (m, 2H), 2.08/2.06* (s, 3H), 1.97 – 1.74 (m, 2H).

^{13}C NMR (176 MHz, CDCl$_3$) δ 175.1/174.7*, 172.4/172.2*, 171.3*/171.3, 136.2/135.8*, 129.1/129.0*, 128.5*/128.4, 127.1*/126.8, 53.4/53.0*, 52.3*/52.1, 46.7*/46.4, 37.7/36.0*, 33.1*/32.9, 32.2/31.2*, 23.5*/23.1, 21.7/21.0*.

HRMS (ESI-TOF) m/z: [M + H]+ Calcd for $C_{17}H_{25}N_2O_4$ 321.1809; Found: 321.1816.

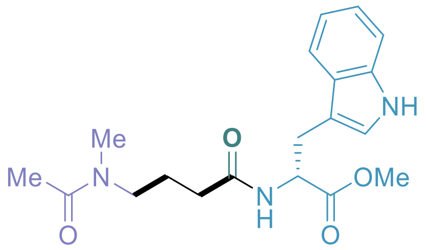

methyl (4-(*N*-methylacetamido)butanoyl)-D-tryptophanate (9b)

This reaction was conducted on a 0.3 mmol scale with the general procedure II. The crude product was purified by silica gel chromatography (EA/MeOH = 10:1) to afford the title compound as a colorless oil (84.0 mg, 78% yield). The identity of the product was confirmed by ^{1}H NMR, ^{13}C NMR, and HRMS. Analysis of NMR spectra revealed the presence of two amide bond rotamers. These conformational isomers have been observed and studied in similar compounds.[1] The minor distinguishable rotamer peak is denoted with *.

^{1}H NMR (700 MHz, CDCl$_3$) δ 8.67*/8.56 (s, 1H), 7.52 (dd, J = 21.3, 8.0 Hz, 1H), 7.34 (dd, J = 11.3, 8.1 Hz, 1H), 7.20 – 7.14 (m, 1H), 7.14 – 7.06 (m, 1H), 7.02/6.97* (s, 1H) 6.78/6.10* (d, J = 7.8 Hz, 1H), 4.99 – 4.73 (m, 1H), 3.71*/3.67 (s, 3H), 3.50 – 3.15 (m, 4H), 2.92/2.84* (s, 3H), 2.30 – 2.08 (m, 2H), 2.05/2.01* (s, 3H), 1.93 – 1.63 (m, 2H).

^{13}C NMR (176 MHz, CDCl$_3$) δ 172.6/172.4*, 172.4/171.4*, 171.1/170.7*, 136.2*/136.1, 127.6*/127.5, 123.0/122.8*, 122.2*/122.0, 119.6*/119.4, 118.5/118.3*, 111.4*/111.3, 110.0/109.7*, 53.0, 52.4*/52.3, 49.8*/46.5, 36.0/33.1*, 33.2/32.4*, 27.5/27.4*, 23.5*/23.1, 21.8/21.1*.

HRMS (ESI-TOF) m/z: [M + H]+ Calcd for $C_{19}H_{26}N_3O_4$ 360.1918; Found: 360.1820

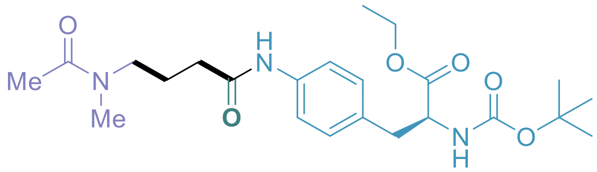

ethyl (S)-2-((tert-butoxycarbonyl)amino)-3-(4-(4-(*N*-methylacetamido)butanamido)phenyl)propanoate (9c)

This reaction was conducted on a 0.3 mmol scale with the general procedure II. The crude product was purified by silica gel chromatography (EA/MeOH = 10:1) to afford the title compound as a colorless oil (90.2 mg, 67% yield). The identity of the product was confirmed by ^{1}H NMR, ^{13}C NMR, and HRMS. Analysis of NMR spectra revealed the presence of two amide bond rotamers. These conformational isomers have been observed and studied in similar compounds.[1] The minor distinguishable rotamer peak is denoted with *.

^{1}H NMR (700 MHz, CDCl$_3$) δ 9.42/8.72* (s, 1H), 7.58/7.48* (d, J = 8.3 Hz, 2H), 7.14 – 6.96 (m, 2H), 5.23 – 4.42 (m, 1H), 4.32 – 4.06 (m, 2H), 3.44 (dt, J = 76.7, 6.9 Hz, 2H), 3.11 – 2.84 (m, 5H), 2.50 – 2.27 (m, 2H), 2.11/2.08* (s, 3H), 1.97 – 1.82 (m, 2H), 1.42 (s, 9H), 1.24 (t, J = 7.1 Hz, 3H).

^{13}C NMR (176 MHz, CDCl$_3$) δ 172.0*/171.8, 171.2/170.7*, 170.8/170.4*, 155.2, 137.7, 131.2, 129.8*/129.7, 119.9*/119.7, 79.8, 61.3*/61.3, 54.5/49.9*, 46.5, 38.0/37.5*, 36.1/35.1*, 34.4/33.1*, 28.3, 23.6*/21.9, 21.5/21.2*, 14.1.

HRMS (ESI-TOF) m/z: [M + H]+ Calcd for $C_{23}H_{36}N_3O_6$ 450.2599; Found: 450.2597

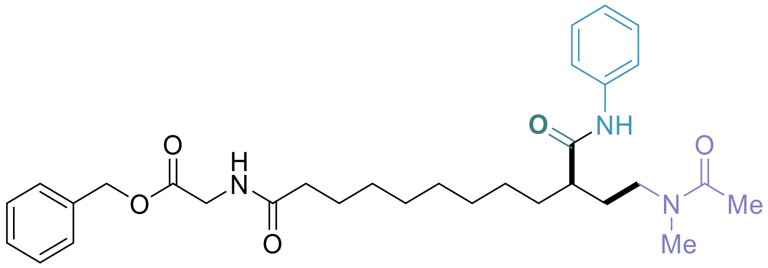

benzyl-(12-(*N*-methylacetamido)-10-(phenylcarbamoyl)dodecanoyl)glycinate (9d)

This reaction was conducted on a 0.3 mmol scale with the general procedure II. The crude product was purified by silica gel chromatography (EA/MeOH = 8:1) to afford

the title compound as a colorless oil (101.5 mg, 63% yield). The identity of the product was confirmed by ^{1}H NMR, ^{13}C NMR, and HRMS.

^{1}H NMR (400 MHz, CDCl$_3$) δ 9.35 (s, 1H), 7.69 (d, J = 8.0 Hz, 2H), 7.55 – 7.21 (m, 9H), 7.05 (t, J = 7.4 Hz, 1H), 5.18 (s, 2H), 4.55 – 3.78 (m, 2H), 3.23 – 2.76 (m, 5H), 2.21 (t, J = 7.4 Hz, 2H), 2.14 – 2.01 (m, 4H), 1.89 – 1.77 (m, 2H), 1.69 – 1.54 (m, 4H), 1.27 (d, J = 8.2 Hz, 9H).

^{13}C NMR (101 MHz, CDCl$_3$) δ 173.8, 173.3, 172.2, 170.0, 138.7, 135.1, 128.8, 128.6, 128.5, 128.3, 123.6, 119.6, 67.1, 46.1, 44.9, 41.3, 36.2, 36.1, 33.2, 31.4, 29.4, 29.0, 29.0, 27.4, 25.4, 22.0.

HRMS (ESI-TOF) m/z: [M + H]+ Calcd for $C_{31}H_{44}N_3O_5$ 538.3275; Found: 538.3277

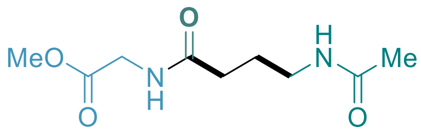

methyl (4-acetamidobutanoyl)glycinate (9e)

This reaction was conducted on a 0.3 mmol scale with the general procedure V. The crude product was purified by silica gel chromatography (EA/MeOH = 5:1) to afford the title compound as a white solid (30.5 mg, 47% yield). The identity of the product was confirmed by ^{1}H NMR, ^{13}C NMR, and HRMS.

^{1}H NMR (400 MHz, DMSO-d$_6$) δ 8.26 (s, 1H), 7.81 (s, 1H), 3.81 (d, J = 5.9 Hz, 2H), 3.62 (s, 3H), 3.01 (q, J = 6.9 Hz, 2H), 2.12 (t, J = 7.5 Hz, 2H), 1.78 (s, 3H), 1.76 – 1.48 (m, 2H).

^{13}C NMR (101 MHz, DMSO-d$_6$) δ 172.9, 171.0, 169.5, 52.1, 41.0, 38.6, 33.0, 25.8, 23.1.

HRMS (ESI-TOF) m/z: [M + H]+ Calcd for $C_9H_{17}N_2O_4$ 217.1183; Found: 217.1191

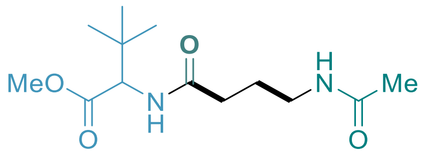

methyl 2-(4-acetamidobutanamido)-3,3-dimethylbutanoate (9f)

This reaction was conducted on a 0.3 mmol scale with the general procedure V. The crude product was purified by silica gel chromatography (EA/MeOH = 5:1) to afford the title compound as a white solid (51.4 mg, 63% yield). The identity of the product was confirmed by ^{1}H NMR, ^{13}C NMR, and HRMS.

^{1}H NMR (400 MHz, DMSO-d$_6$) δ 8.05 (s, 1H), 7.81 (s, 1H), 4.16 (d, J = 8.5 Hz, 1H), 3.62 (s, 3H), 3.23 – 2.84 (m, 2H), 2.46 – 2.03 (m, 2H), 1.79 (s, 3H), 1.73 – 1.33 (m, 2H), 0.93 (s, 9H).
^{13}C NMR (101 MHz, DMSO-d$_6$) δ 172.7, 172.1, 169.5, 60.8, 51.8, 38.6, 33.9, 32.9, 27.0, 26.2, 23.1.
HRMS (ESI-TOF) m/z: [M + H]+ Calcd for $C_{13}H_{25}N_2O_4$ 273.1089; Found: 273.1081

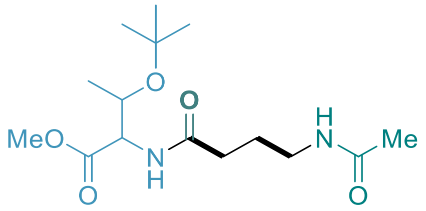

methyl 2-(4-acetamidobutanamido)-3-(*tert*-butoxy)butanoate (9g)

This reaction was conducted on a 0.3 mmol scale with the general procedure V. The crude product was purified by silica gel chromatography (EA/MeOH = 5:1) to afford the title compound as a white solid (48.3 mg, 51% yield). The identity of the product was confirmed by ^{1}H NMR, ^{13}C NMR, and HRMS. Analysis of NMR spectra revealed the presence of two amide bond rotamers. These conformational isomers have been observed and studied in similar compounds.[1] The minor distinguishable rotamer peak is denoted with *.

^{1}H NMR (400 MHz, DMSO-d$_6$) δ 7.84 (s, 1H), 7.82 (s, 1H), 4.39 (dd, J = 8.8, 3.0 Hz, 1H), 4.11 (dd, J = 6.2, 3.1 Hz, 1H), 3.62 (s, 3H), 3.12 – 2.88 (m, 2H), 2.39 – 2.18 (m, 2H), 1.79 (s, 3H), 1.61 (q, J = 7.3, 6.7 Hz, 2H), 1.20 – 0.92 (m, 12H).
^{13}C NMR (101 MHz, DMSO-d$_6$) δ 173.1, 171.5, 169.4, 73.9, 67.5, 57.9, 52.1, 38.6, 32.9, 28.6, 26.1, 23.1, 20.6.
HRMS (ESI-TOF) m/z: [M + H]+ Calcd for $C_{15}H_{29}N_2O_5$ 317.2071; Found: 317.2080

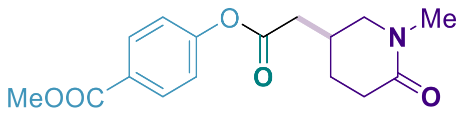

methyl-4-(2-(1-methyl-6-oxopiperidin-3-yl)acetoxy)benzoate (10)

This reaction was conducted on a 0.3 mmol scale with the general procedure 4. The crude product was purified by silica gel chromatography (PE/EA = 1:3) to afford the title compound as a colorless oil (45.8 mg, 50% yield). The identity of the product was confirmed by ^{1}H NMR, ^{13}C NMR, and HRMS.

¹H NMR (400 MHz, $CDCl_3$) δ 8.08 (d, J = 8.7 Hz, 2H), 7.17 (d, J = 8.7 Hz, 2H), 3.92 (s, 3H), 3.62 – 3.35 (m, 1H), 3.24 – 3.07 (m, 1H), 3.03 – 2.98 (m, 1H), 2.96 (s, 3H), 2.76 – 2.62 (m, 1H), 2.59 – 2.36 (m, 3H), 2.12 – 1.93 (m, 1H), 1.70 – 1.54 (m, 1H).
¹³C NMR (101 MHz, $CDCl_3$) δ 169.5, 169.1, 166.1, 153.8, 131.1, 127.8, 121.3, 121.3, 54.3, 52.1, 37.7, 34.7, 30.9, 30.7, 27.3.
HRMS (ESI-TOF) m/z: [M + H]+ Calcd for $C_{16}H_{20}NO_5$ 306.1336; Found: 306.1339

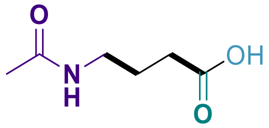

4-acetamidobutanoic acid (11)

This reaction was conducted on a 1 mmol scale with the general procedure 5. The crude product was purified by recrystallization (MeOH) to afford the title compound as a white solid (134.9 mg, 93% yield). The identity of the product was confirmed by ^{1}H NMR, ^{13}C NMR, and HRMS.

¹H NMR (400 MHz, Methanol-d₄) δ2.84 (d, J = 6.9 Hz, 2H), 1.98 (t, J = 7.4 Hz, 2H), 1.58 (s, 3H), 1.52 – 1.21 (m, 2H).
¹³C NMR (101 MHz, Methanol-d₄) δ176.9, 173.4, 39.8, 32.2, 25.8, 22.5.
HRMS (ESI-TOF) m/z: [M + H]+ Calcd for $C_6H_{12}NO_3$ 146.0812; Found: 146.0812

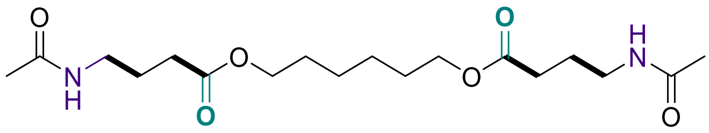

hexane-1,6-diyl bis(4-acetamidobutanoate) (13)

This reaction was conducted on a 0.3 mmol scale with the general procedure 6 The crude product was purified by silica gel chromatography (EA/MeOH = 10:1) to afford the title compound as a white solid (45.8 mg, 41% yield). The identity of the product was confirmed by ^{1}H NMR, ^{13}C NMR, and HRMS.

¹H NMR (400 MHz, $CDCl_3$) δ 6.30 (s, 2H), 4.01 (t, J = 6.6 Hz, 4H), 3.37 – 2.91 (m, 4H), 2.31 (t, J = 7.3 Hz, 4H), 1.91 (s, 6H), 1.83 – 1.65 (m, 4H), 1.65 – 1.41 (m, 4H), 1.42 – 1.04 (m, 4H).
¹³C NMR (101 MHz, $CDCl_3$) δ 173.3, 170.3, 64.3, 38.9, 31.6, 28.3, 25.4, 24.6, 23.0.
HRMS (ESI-TOF) m/z: [M + H]+ Calcd for $C_{18}H_{33}N_2O_6$ 373.2333; Found: 373.2340

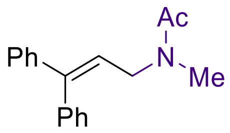

***N*-(3,3-diphenylallyl)-*N*-methylacetamide (15)**

This reaction was conducted on a 0.3 mmol scale with the general procedure 7. The crude product was purified by silica gel chromatography (PE/EA = 1:1) to afford the title compound as a colorless oil (35.8 mg, 45% yield). The identity of the product was confirmed by ^{1}H NMR and HRMS. Analysis of NMR spectra revealed the presence of two amide bond rotamers. These conformational isomers have been observed and studied in similar compounds.[1] The minor distinguishable rotamer peak is denoted with *.

^{1}H NMR (400 MHz, CDCl$_3$) δ 7.49 – 7.31 (m, 3H), 7.32 – 7.19 (m, 5H), 7.22 – 7.10 (m, 2H), 6.15 – 5.92 (m, 1H), 4.08*/3.95 (d, J = 6.6 Hz, 2H), 2.91*/2.87 (s, 3H), 2.08/1.97* (s, 3H).

HRMS (ESI-TOF) m/z: [M + H]+ Calcd for $C_{18}H_{20}NO$ 266.1539; Found: 266.1542

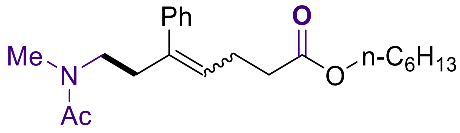

hexyl-7-(*N*-methylacetamido)-5-phenylhept-4-enoate (16)

This reaction was conducted on a 0.3 mmol scale with the general procedure 8. The crude product was purified by silica gel chromatography (PE/EA = 1:3) to afford the title compound as a colorless oil (44.2 mg, 41% yield). The identity of the product was confirmed by ^{1}H NMR, ^{13}C NMR, and HRMS. Analysis of NMR spectra revealed the presence of two amide bond rotamers. These conformational isomers have been observed and studied in similar compounds.[1] The minor distinguishable rotamer peak is denoted with *.

^{1}H NMR (400 MHz, CDCl$_3$) δ 7.55 – 7.25 (m, 4H), 7.25 – 6.89 (m, 1H), 6.21 – 5.55 (m, 1H), 4.49 – 3.80 (m, 2H), 3.36 (dd, J = 8.3, 6.5 Hz, 1H), 3.31 – 3.12 (m, 1H), 2.94 – 2.70 (m, 5H), 2.70 – 2.50 (m, 3H), 2.34 – 2.23 (m, 1H), 1.99/1.95* (s, 3H), 1.79 – 1.53 (m, 2H), 1.47 – 1.20 (m, 6H), 1.06 – 0.67 (m, 3H).

^{13}C NMR (101 MHz, CDCl$_3$) δ173.2/172.9*, 170.4, 142.2, 138.3/137.5*, 129.2*/128.6, 128.6*/128.4, 127.4*/127.0, 126.2*/126.2, 64.8*/64.7, 49.5*/47.5, 37.0*/34.3, 34.2/33.3*, 31.4/28.9*, 28.6/27.7*, 25.6/24.2*, 24.1*/22.5, 21.9/21.0*, 14.0.

HRMS (ESI-TOF) m/z: [M + H]+ Calcd for $C_{22}H_{34}NO_3$ 360.2533; Found: 360.2538

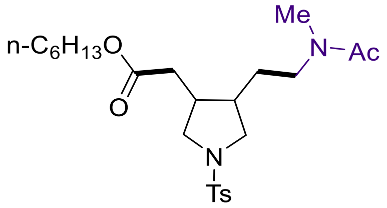

hexyl 2-(4-(2-(*N*-methylacetamido)ethyl)-1-tosylpyrrolidin-3-yl)acetate (17)

This reaction was conducted on a 0.3 mmol scale with the general procedure 8. The crude product was purified by silica gel chromatography (PE/EA = 1:5) to afford the title compound as a colorless oil (75.5 mg, 54% yield). The identity of the product was confirmed by ^{1}H NMR, ^{13}C NMR, and HRMS. Analysis of NMR spectra revealed the presence of two amide bond rotamers. These conformational isomers have been observed and studied in similar compounds.[1] The minor distinguishable rotamer peak is denoted with *.

^{1}H NMR (400 MHz, CDCl$_3$) δ 7.71 (d, J = 8.3 Hz, 2H), 7.52 – 7.14 (m, 2H), 4.60 – 3.78 (m, 2H), 3.75 – 3.25 (m, 2H), 3.23 – 3.10 (m, 2H), 3.10 – 2.96 (m, 1H), 2.96 – 2.81 (m, 4H), 2.52 – 2.28 (m, 4H), 2.18 – 1.93 (m, 5H), 1.58 (q, J = 6.5 Hz, 3H), 1.41 – 1.18 (m, 8H), 1.02 – 0.75 (m, 3H).

^{13}C NMR (101 MHz, CDCl$_3$) δ 172.1, 171.8, 143.5*/143.5, 133.8/133.3*, 129.8*/129.7, 127.6/127.4*, 65.1*/64.9, 52.7, 52.4*/52.3, 52.1/51.3*, 51.1*/49.3, 46.0/45.9*, 41.5*/40.4, 38.7*/38.4, 37.6/37.4*, 37.0/36.1*, 36.0, 33.2*/32.6, 32.4*/31.4, 31.4, 29.9/28.5*, 28.5, 26.3/25.5*, 25.2*/22.5, 21.8/21.8*, 21.5/21.2*.

HRMS (ESI-TOF) m/z: [M + H]+ Calcd for $C_{24}H_{39}N_2O_5S$ 360.2533; Found: 360.2529.

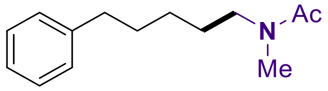

***N*-methyl-*N*-(5-phenylpentyl)acetamide (19)**

This reaction was conducted on a 0.3 mmol scale with the general procedure 10. The crude product was purified by silica gel chromatography (PE/EA = 1:1) to afford the title compound as a colorless oil (34.7 mg, 53% yield). The identity of the product was confirmed by ^{1}H NMR, ^{13}C NMR, and HRMS. Analysis of NMR spectra revealed the presence of two amide bond rotamers. These conformational isomers have been observed and studied in similar compounds.[1] The minor distinguishable rotamer peak is denoted with *.

^{1}H NMR (700 MHz, $CDCl_3$) δ 7.32 – 7.24 (m, 2H), 7.21 – 7.12 (m, 3H), 3.34/3.23* (t, J = 7.7 Hz, 2H), 2.95*/2.89 (s, 3H), 2.73 – 2.56 (m, 2H), 2.06/2.06* (s, 3H), 1.71 – 1.61 (m, 2H), 1.61 – 1.50 (m, 2H), 1.39 – 1.28 (m, 2H).
^{13}C NMR (176 MHz, $CDCl_3$) δ 170.3/170.3*, 142.5*/142.1, 128.4, 128.3/128.2*, 125.8/125.6*, 50.7*/47.4, 36.0*/35.8, 35.8*/33.2, 31.2/31.1*, 28.2/27.1*, 26.4*/26.3, 21.9*/21.2.
HRMS (ESI-TOF) m/z: [M + H]+ Calcd for $C_{14}H_{22}NO$ 220.1696; Found: 220.1699.

4.3 The NMR Spectrum

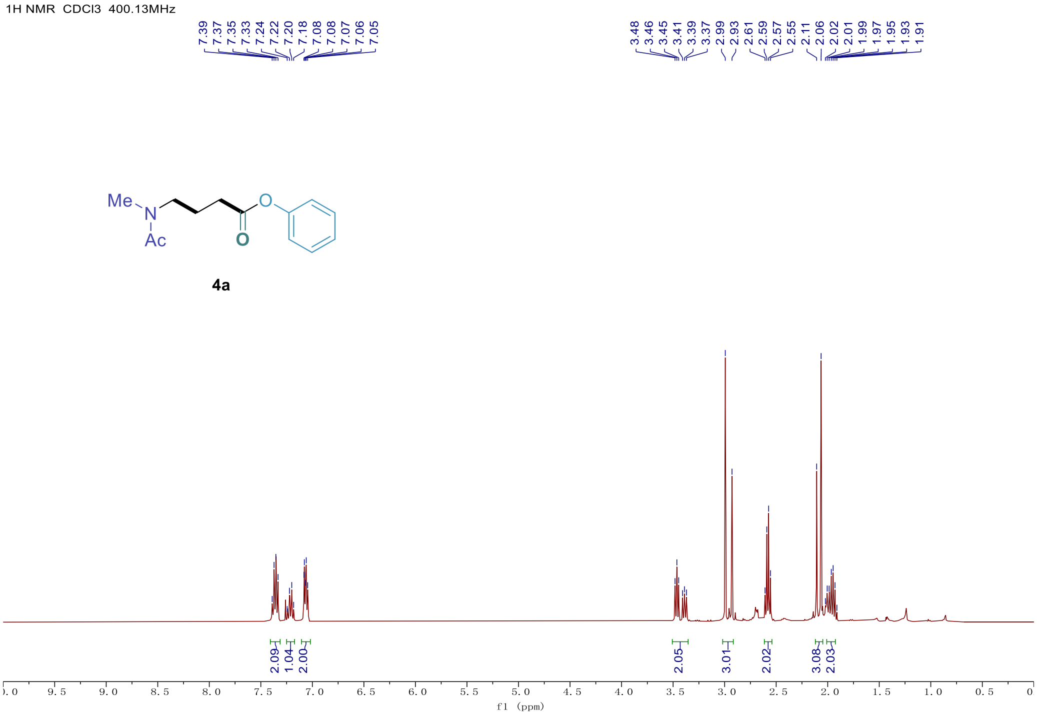

Supplementary Figure 3. ^{1}H NMR of compound **4a** (400 MHz, $CDCl_3$)

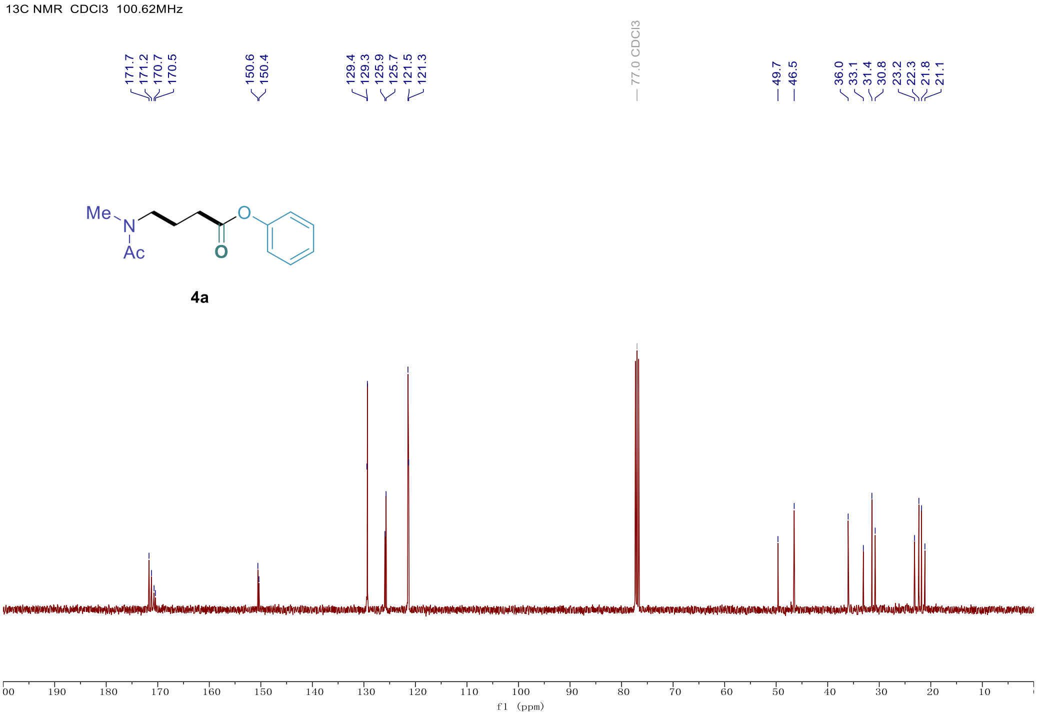

Supplementary Figure 4. ^{13}C NMR of compound **4a** (100 MHz, $CDCl_3$)

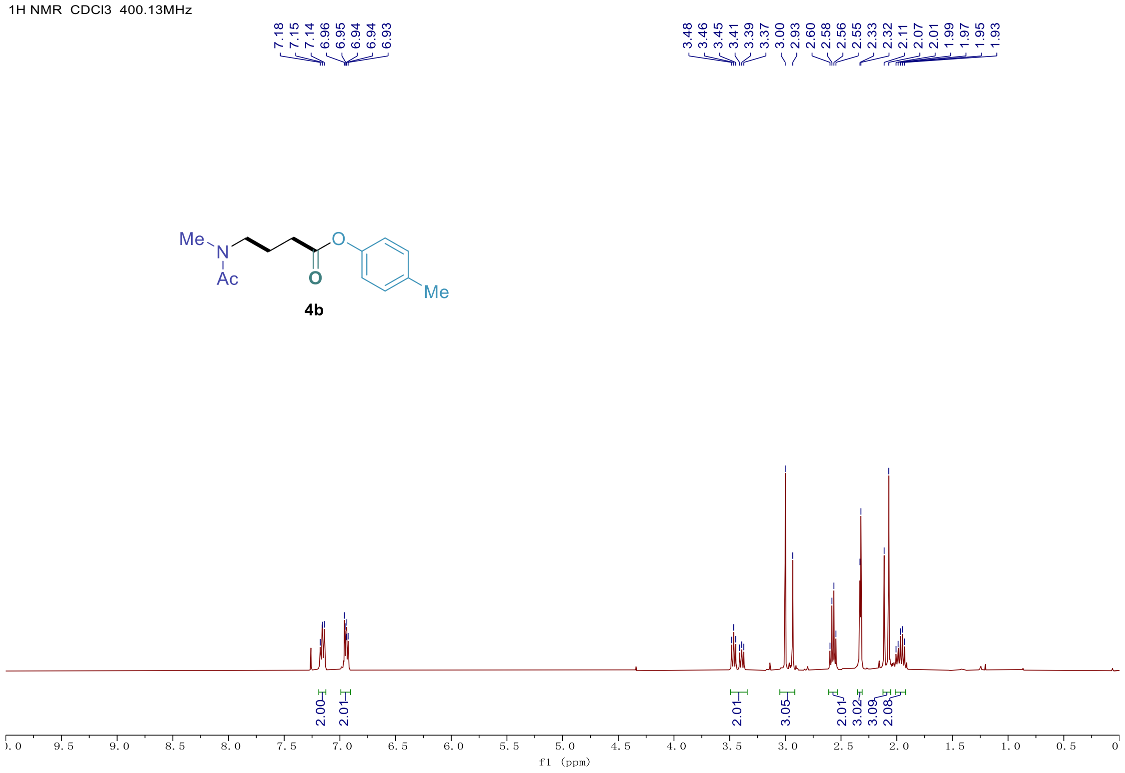

Supplementary Figure 5. ^{1}H NMR of compound **4b** (400 MHz, $CDCl_3$)

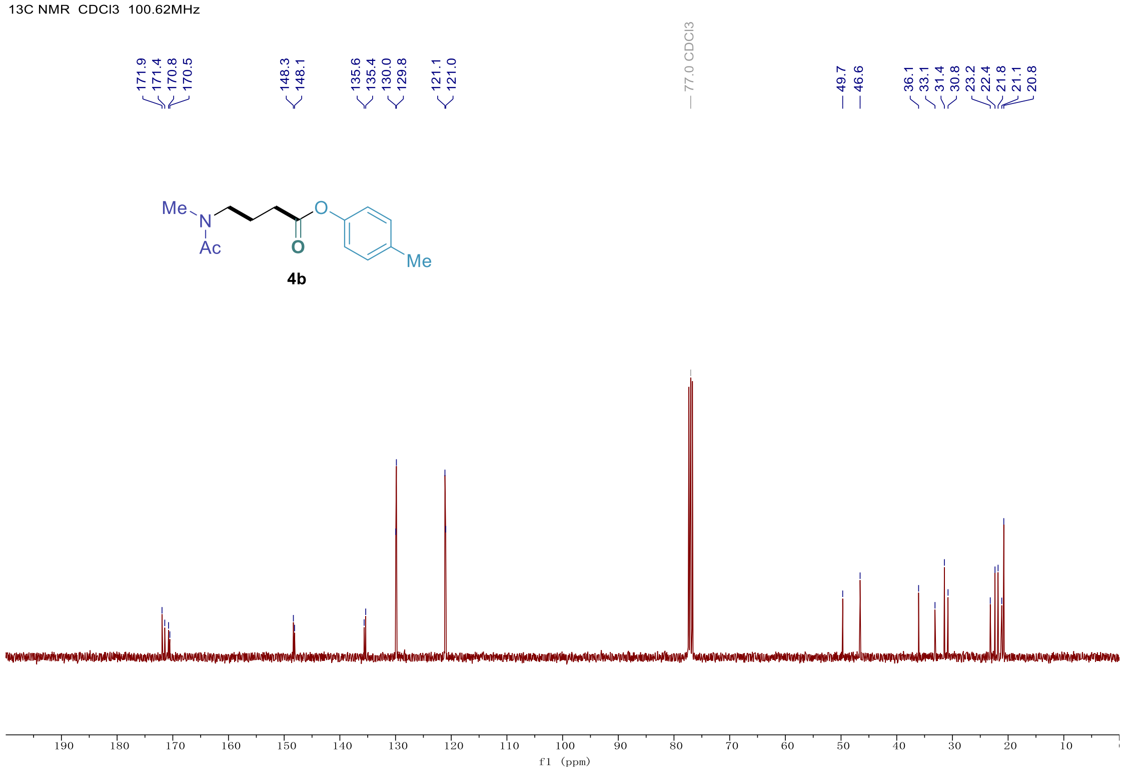

Supplementary Figure 6. ^{13}C NMR of compound **4b** (100 MHz, $CDCl_3$)

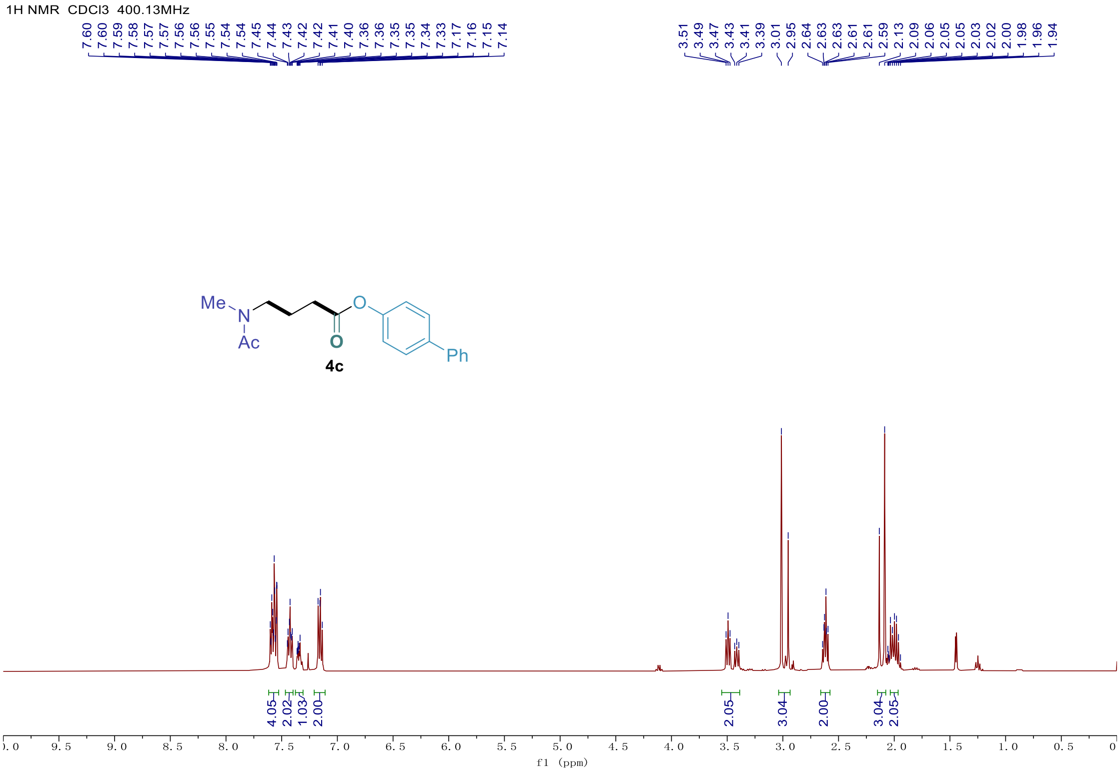

Supplementary Figure 7. ^{1}H NMR of compound **4c** (400 MHz, $CDCl_3$)

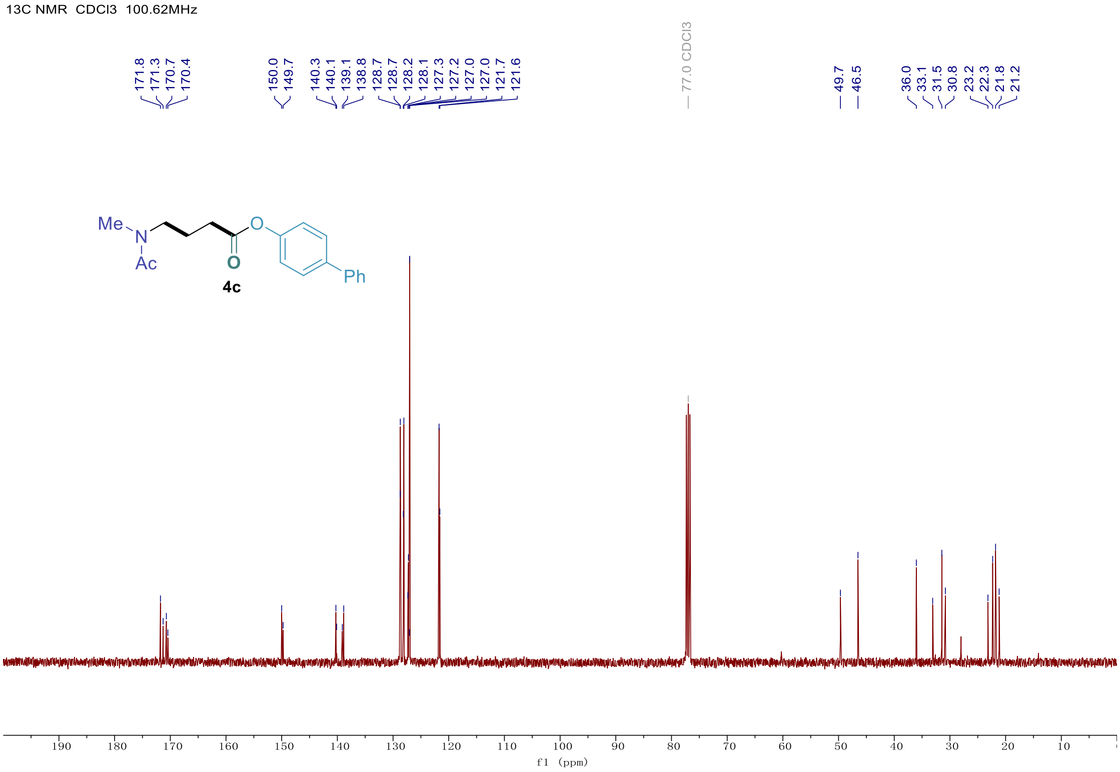

Supplementary Figure 8. ^{13}C NMR of compound **4c** (100 MHz, $CDCl_3$)

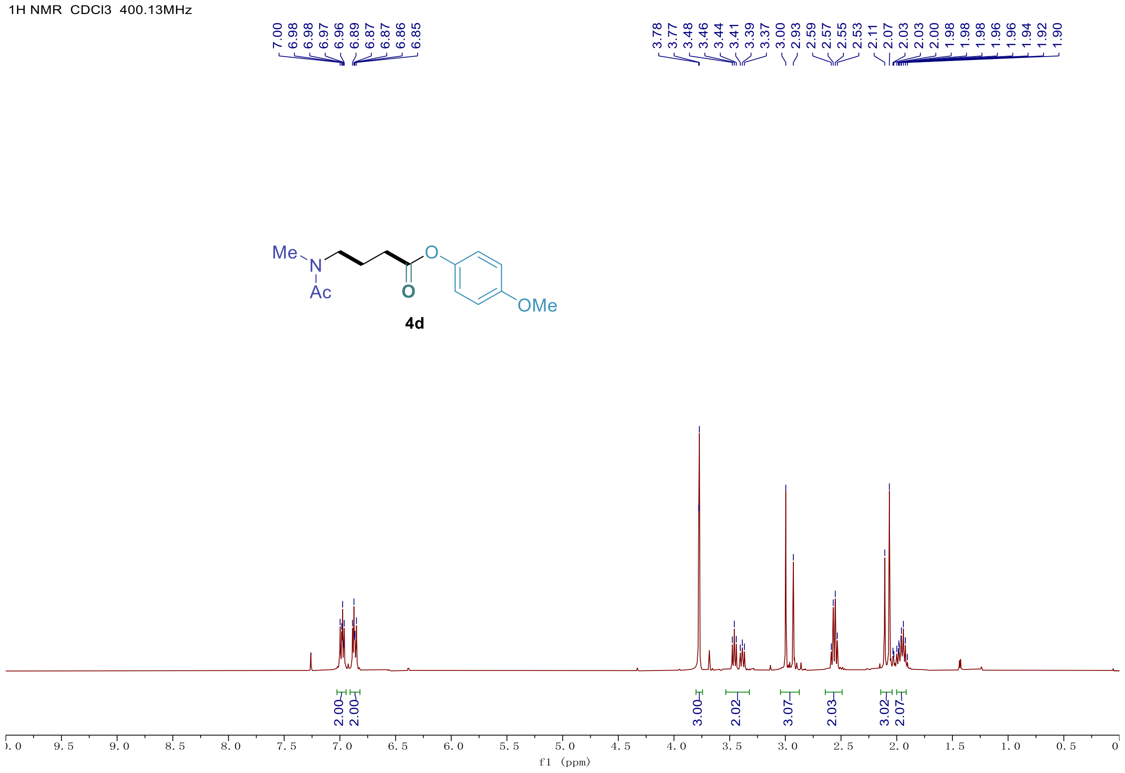

Supplementary Figure 9. ^{1}H NMR of compound **4d** (400 MHz, $CDCl_3$)

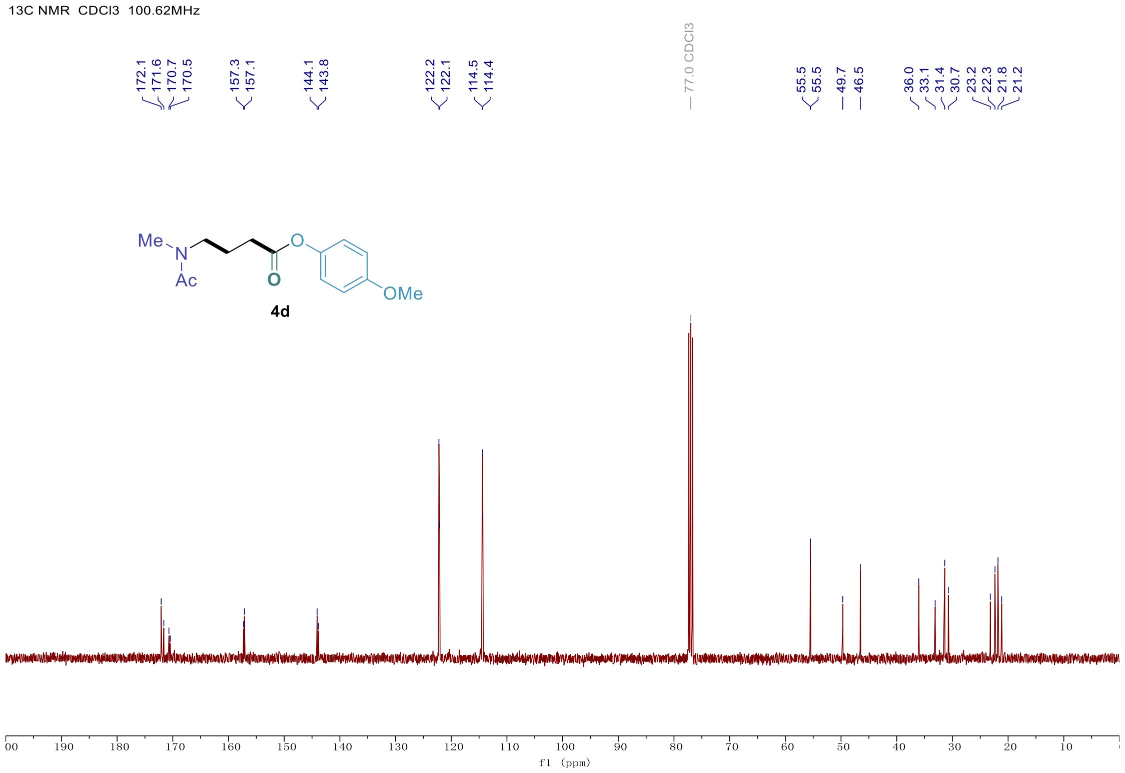

Supplementary Figure 10. ^{13}C NMR of compound **4d** (100 MHz, $CDCl_3$)

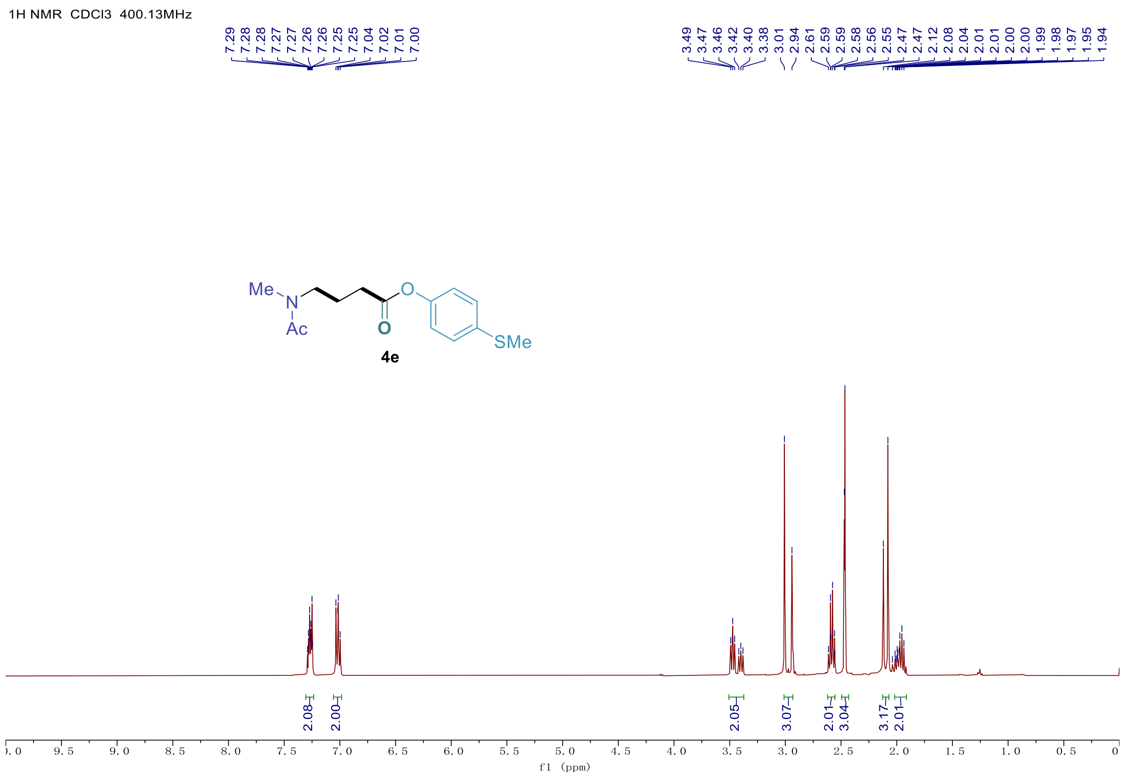

Supplementary Figure 11. ^{1}H NMR of compound **4e** (400 MHz, $CDCl_3$)

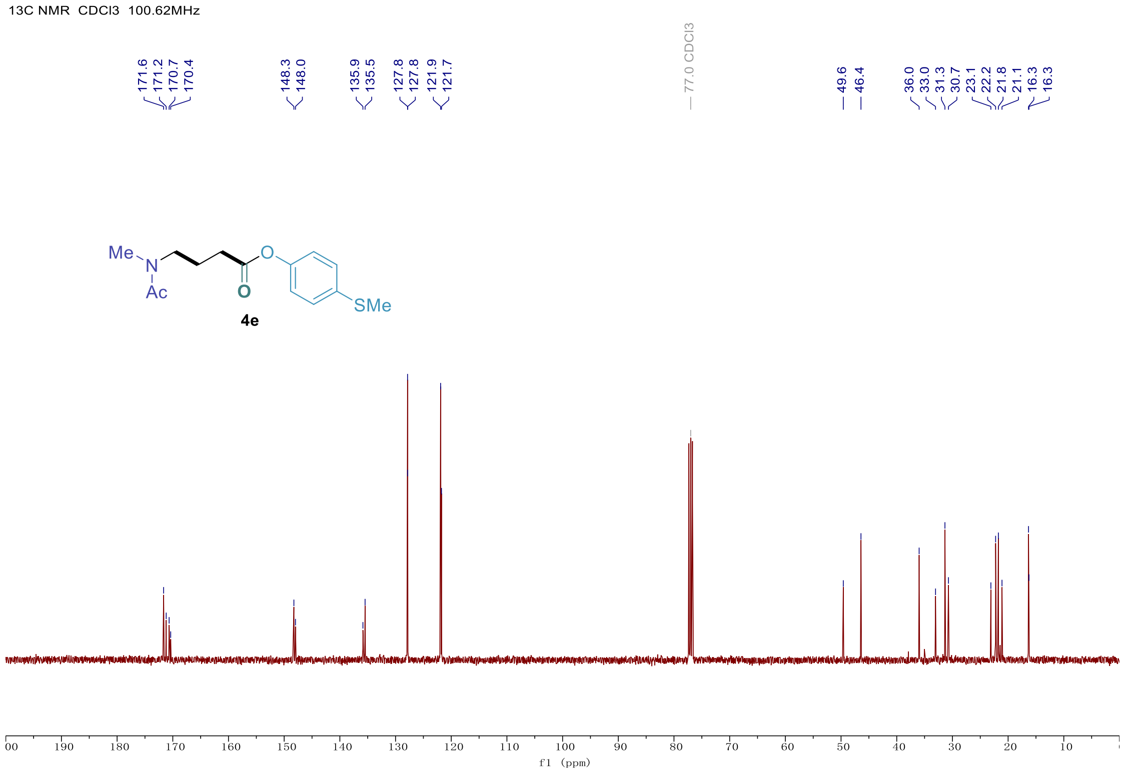

Supplementary Figure 12. ^{13}C NMR of compound **4e** (100 MHz, $CDCl_3$)

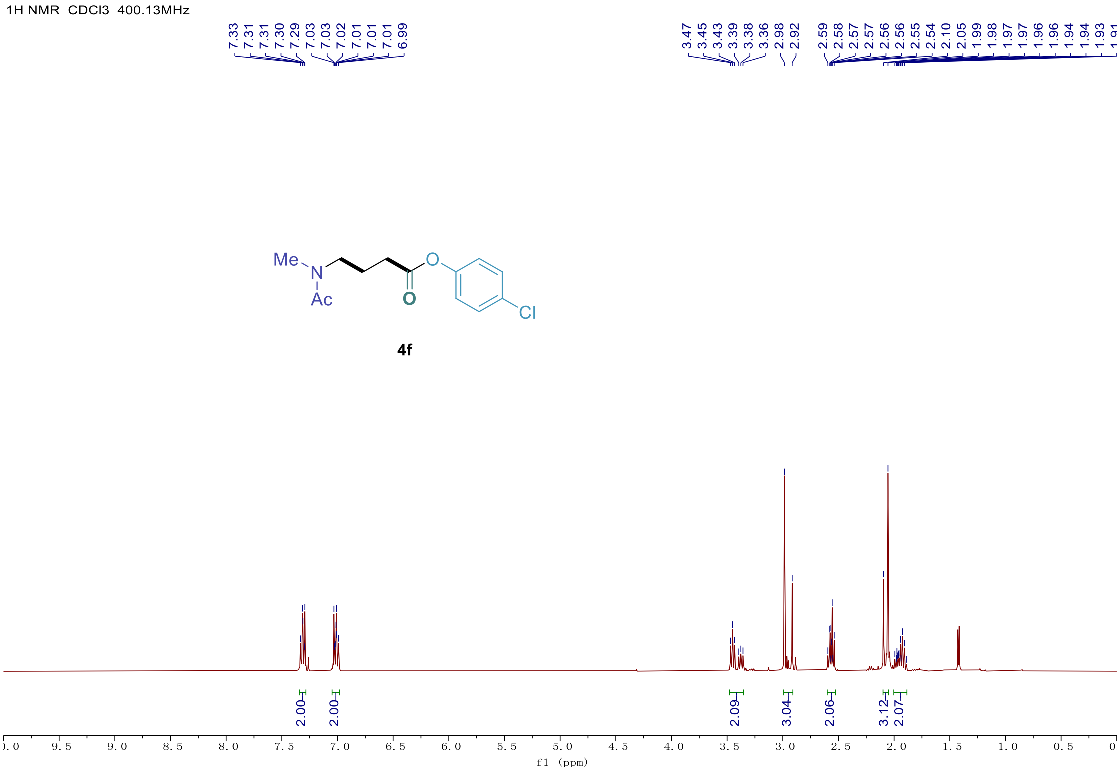

Supplementary Figure 13. ^{1}H NMR of compound **4f** (400 MHz, $CDCl_3$)

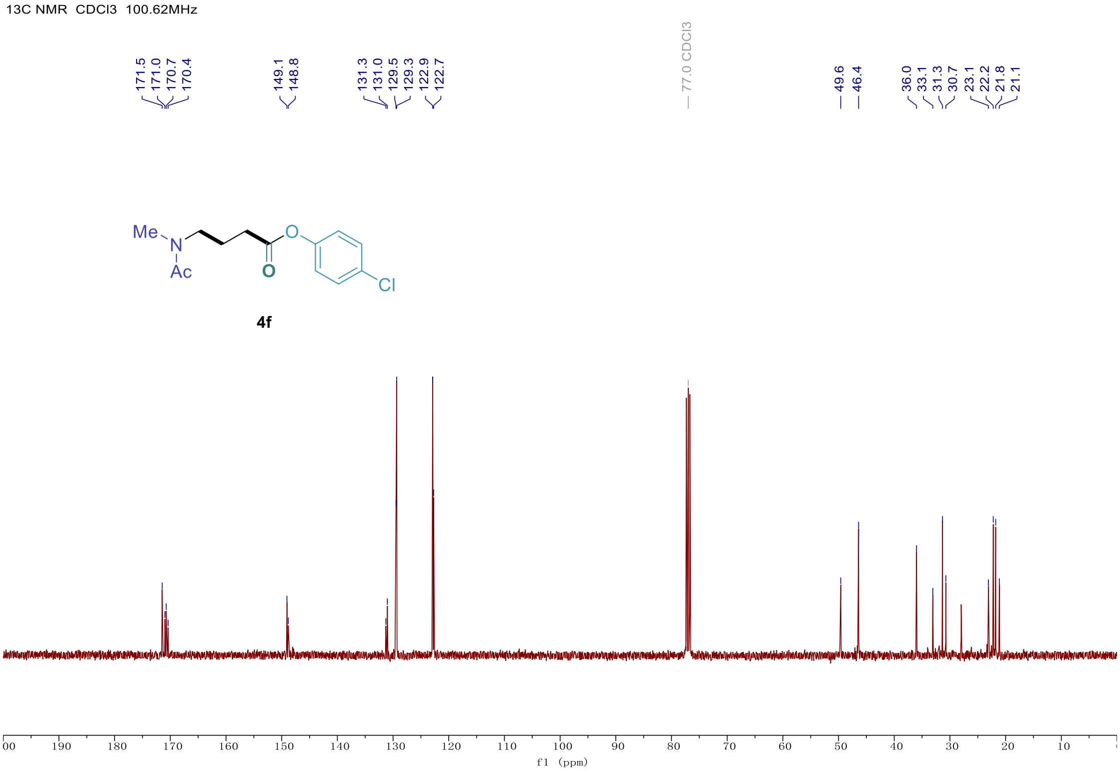

Supplementary Figure 14. ^{13}C NMR of compound **4f** (100 MHz, $CDCl_3$)

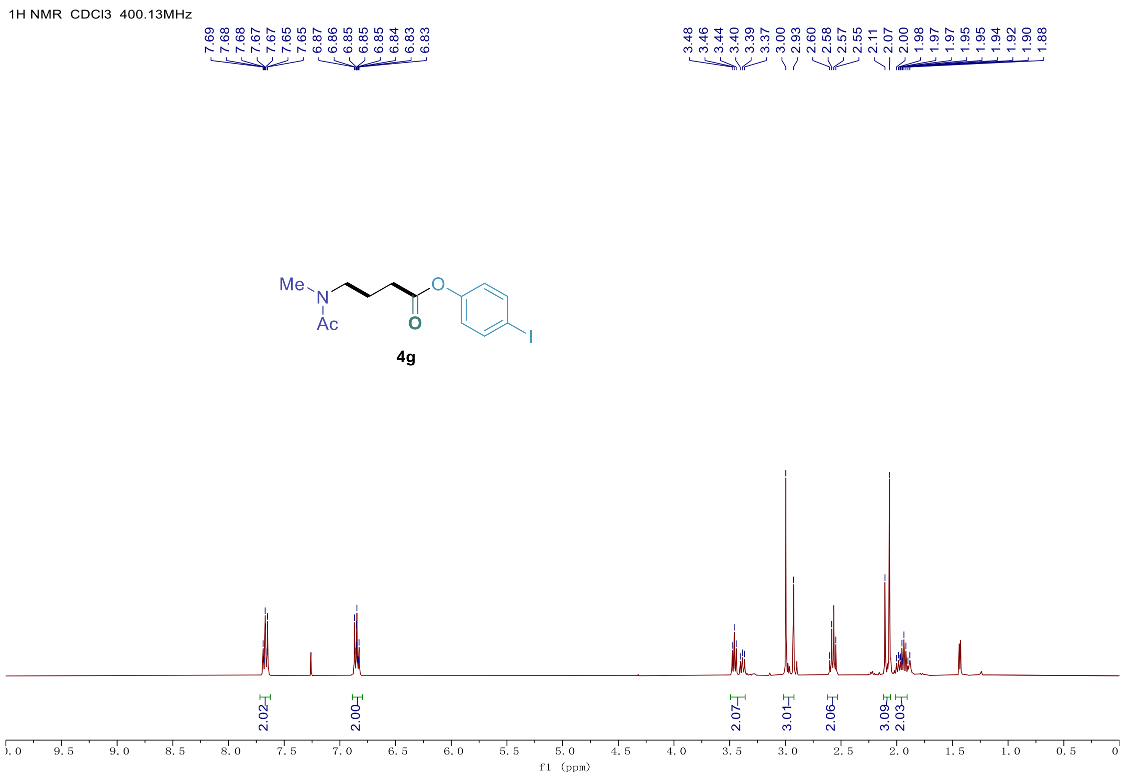

Supplementary Figure 15. ^{1}H NMR of compound **4g** (400 MHz, $CDCl_3$)

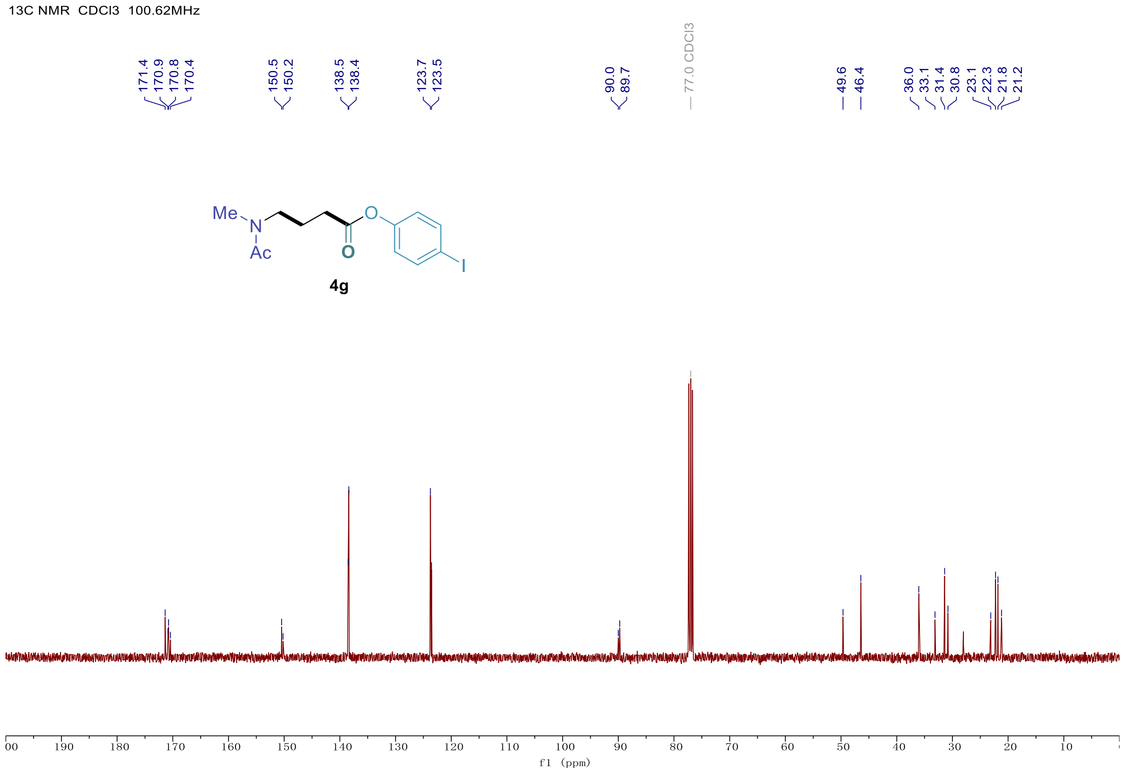

Supplementary Figure 16. ^{13}C NMR of compound **4g** (100 MHz, $CDCl_3$)

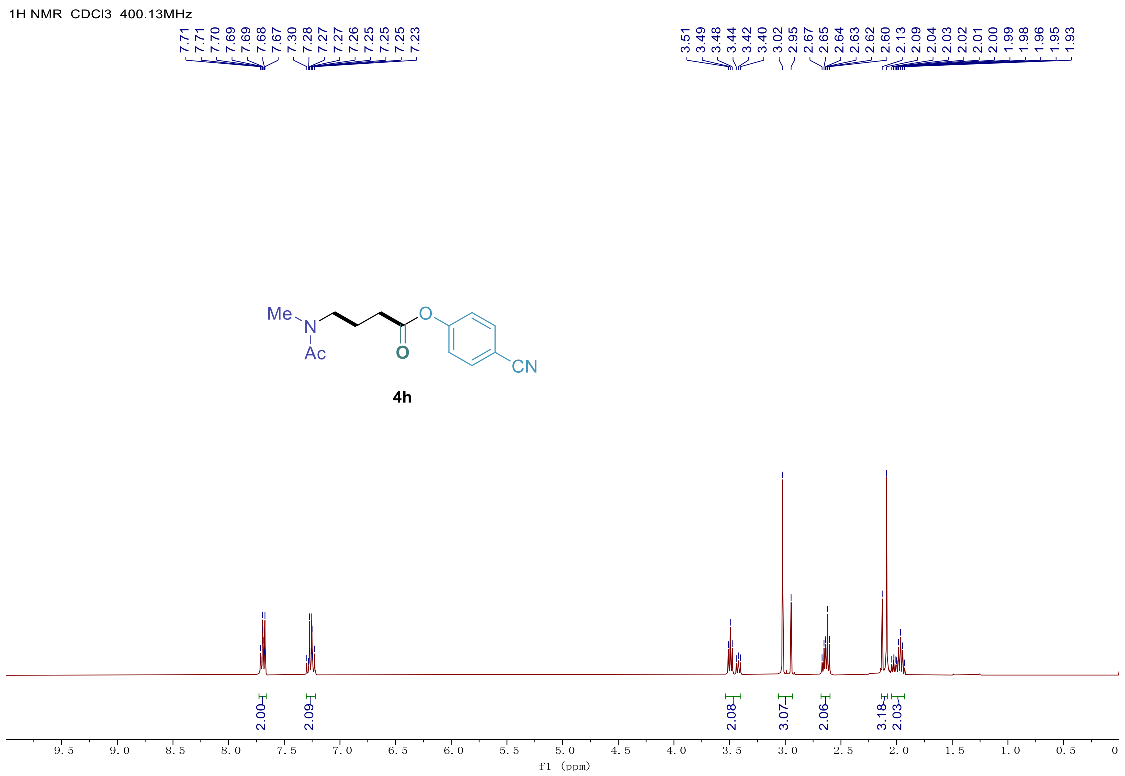

Supplementary Figure 17. ^{1}H NMR of compound **4h** (400 MHz, $CDCl_3$)

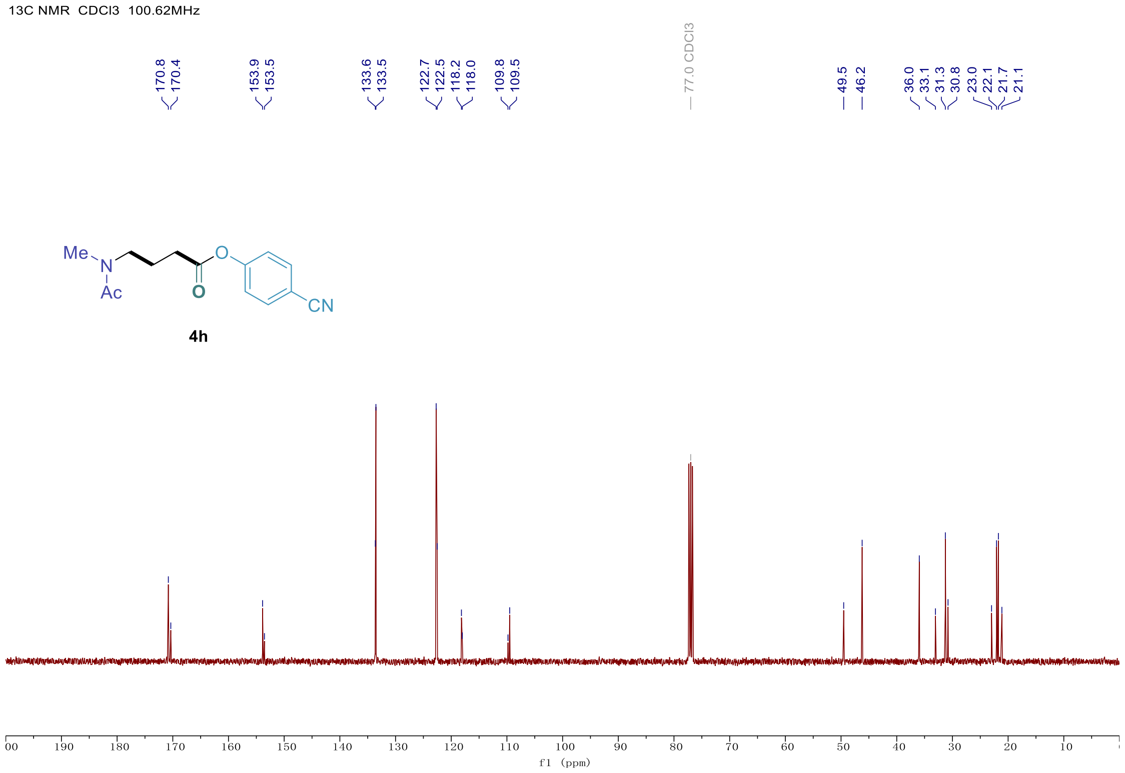

Supplementary Figure 18. ^{13}C NMR of compound **4h** (100 MHz, $CDCl_3$)

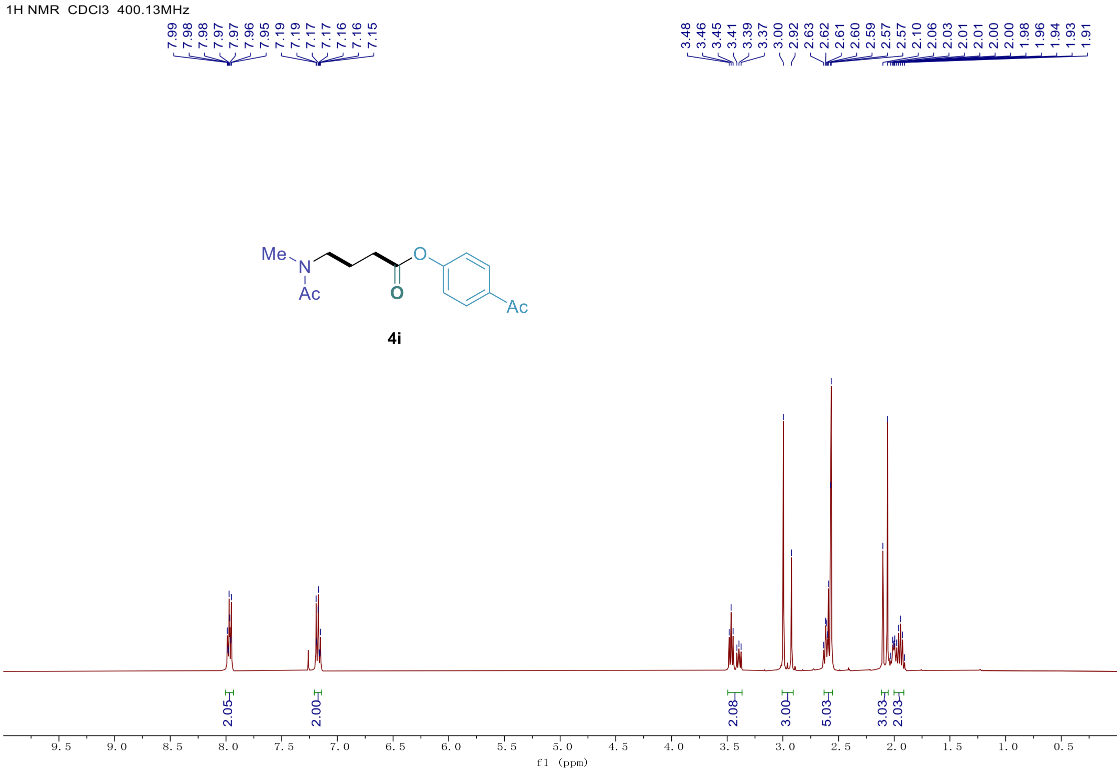

Supplementary Figure 19. ^{1}H NMR of compound **4i** (400 MHz, $CDCl_3$)

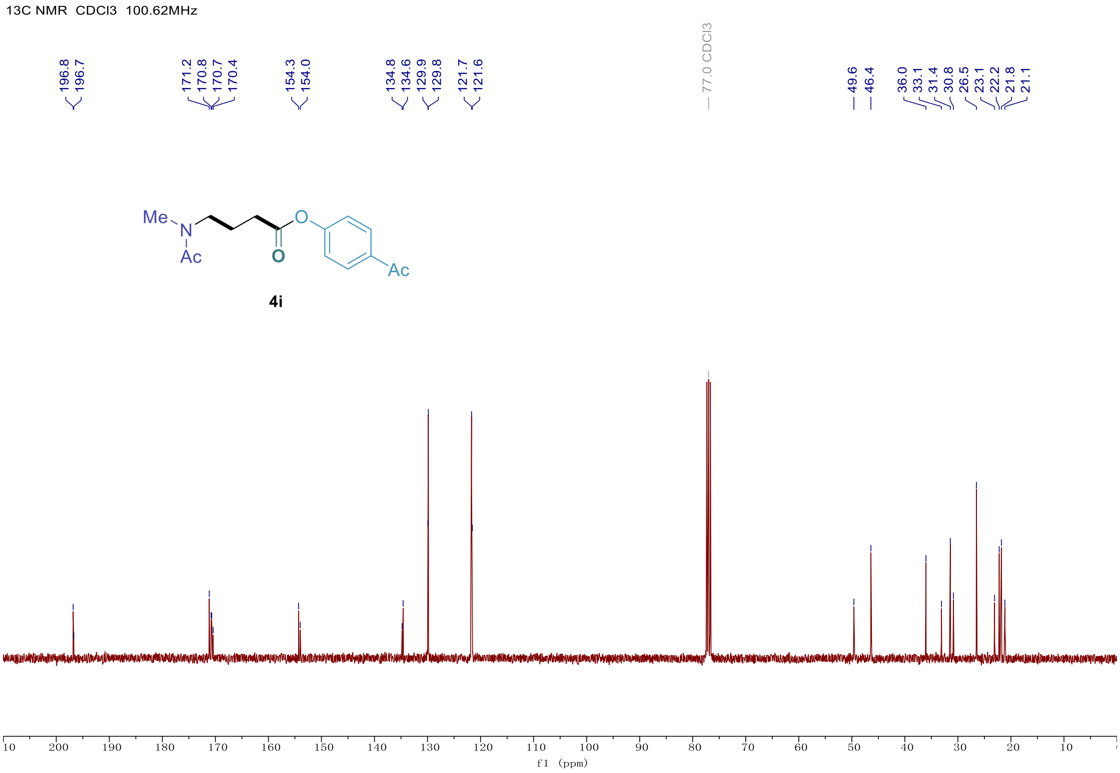

Supplementary Figure 20. ^{13}C NMR of compound **4i** (100 MHz, $CDCl_3$)

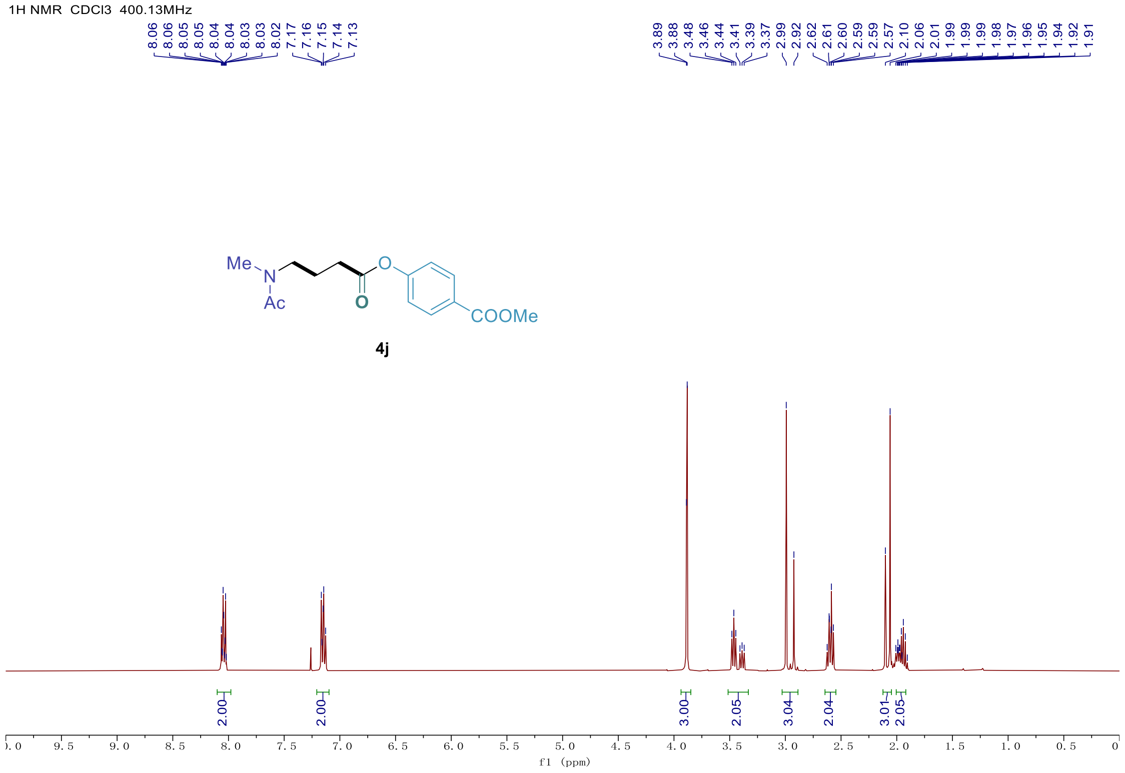

Supplementary Figure 21. ^{1}H NMR of compound **4j** (400 MHz, $CDCl_3$)

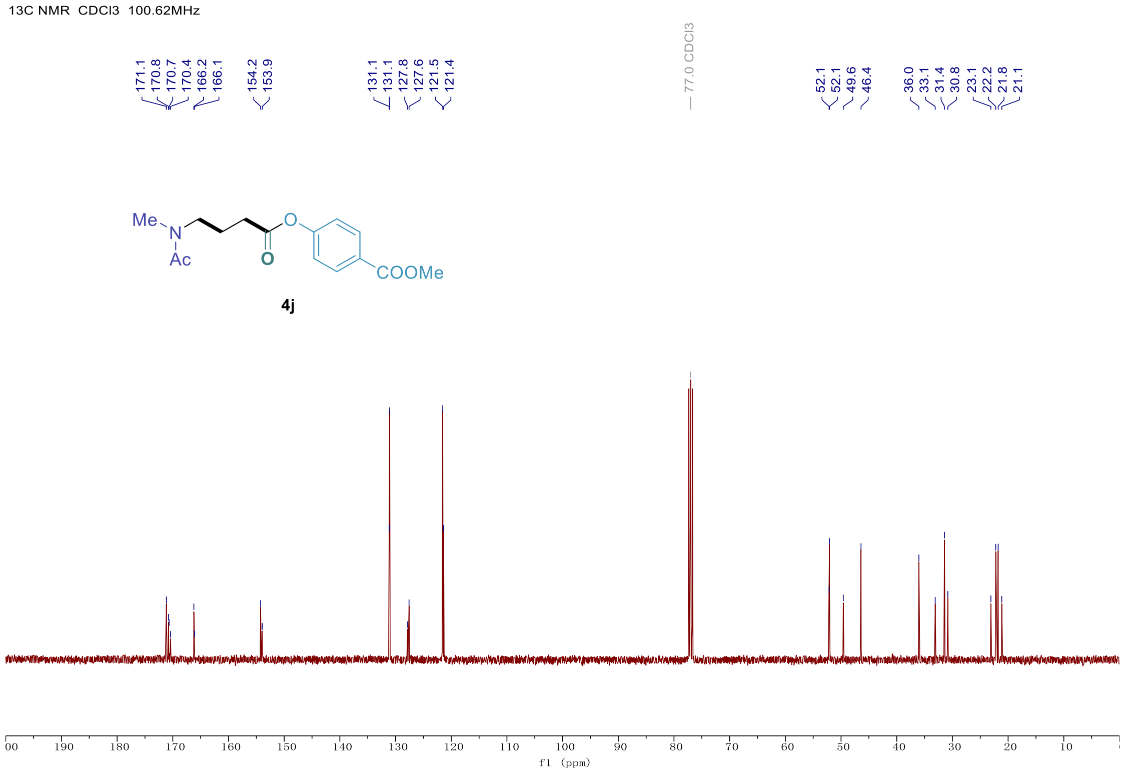

Supplementary Figure 22. ^{13}C NMR of compound **4j** (100 MHz, $CDCl_3$)

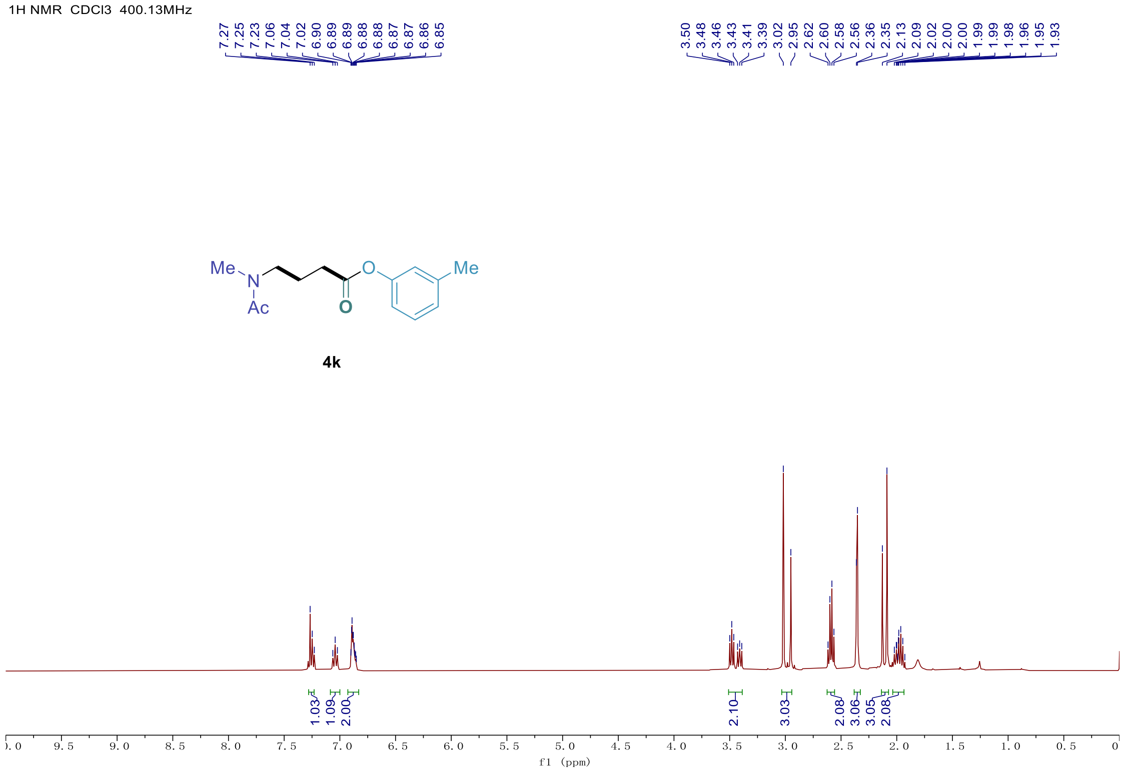

Supplementary Figure 23. ^{1}H NMR of compound **4k** (400 MHz, $CDCl_3$)

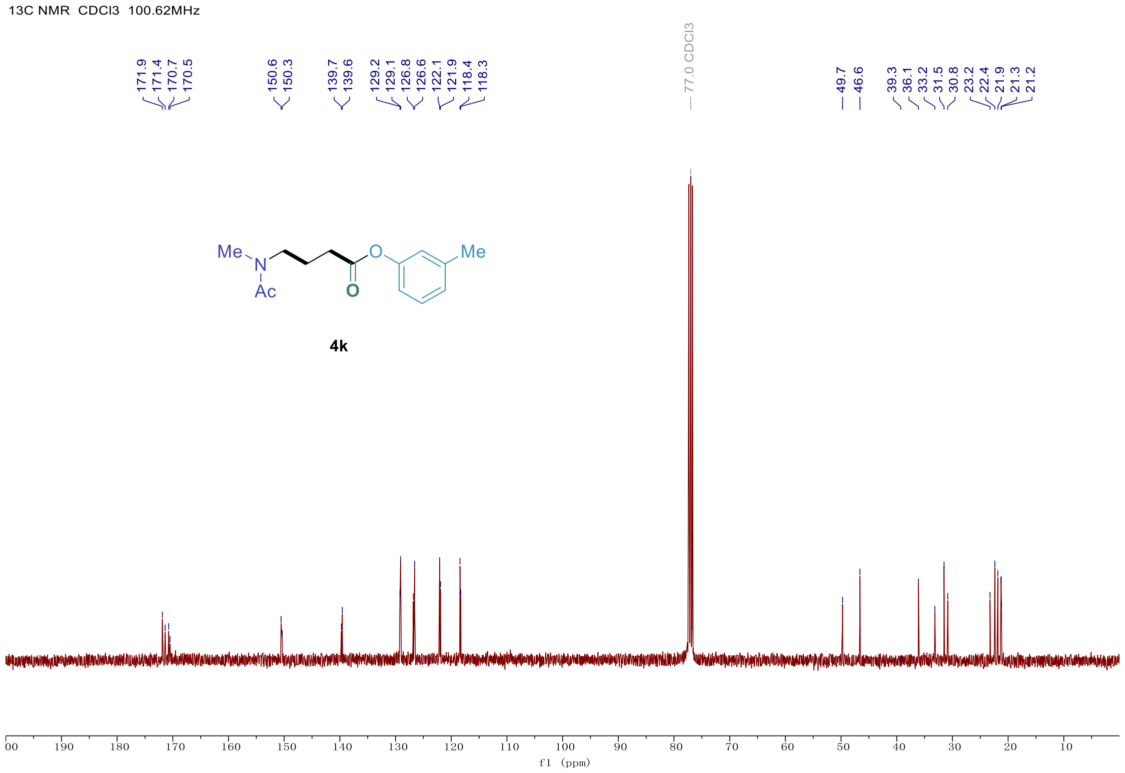

Supplementary Figure 24. ^{13}C NMR of compound **4k** (100 MHz, $CDCl_3$)

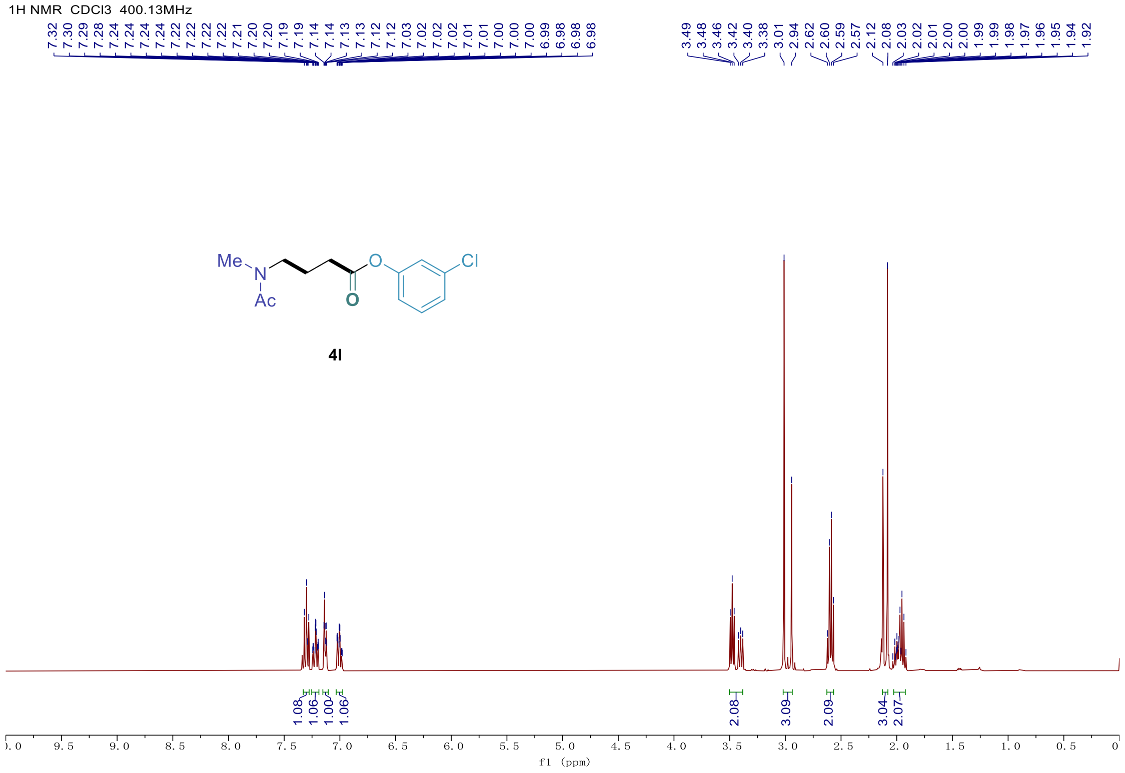

Supplementary Figure 25. ^{1}H NMR of compound **4l** (400 MHz, $CDCl_3$)

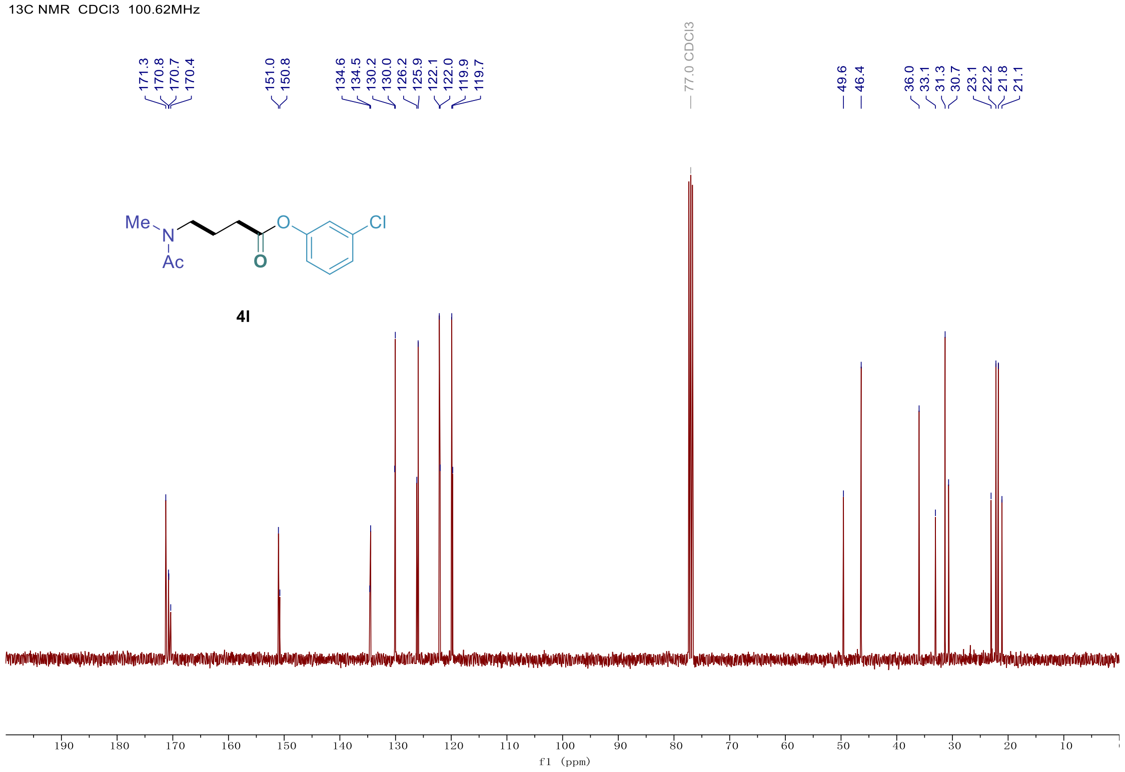

Supplementary Figure 26. ^{13}C NMR of compound **4l** (100 MHz, $CDCl_3$)

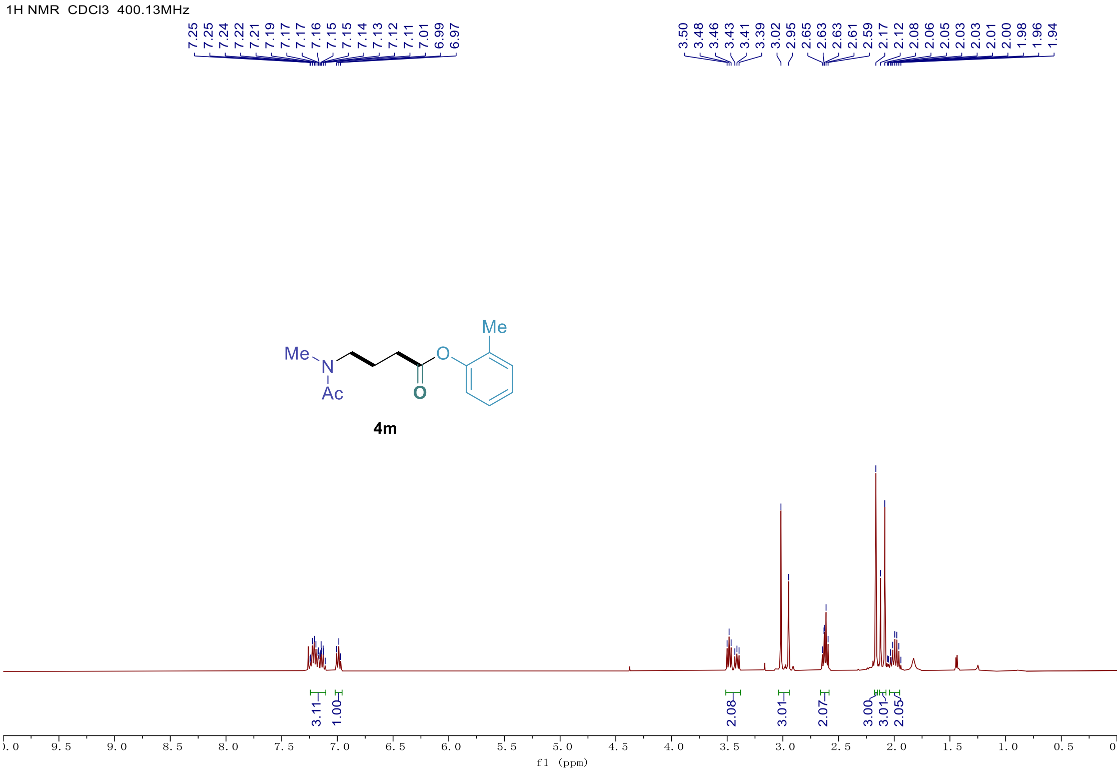

Supplementary Figure 27. ^{1}H NMR of compound **4m** (400 MHz, $CDCl_3$)

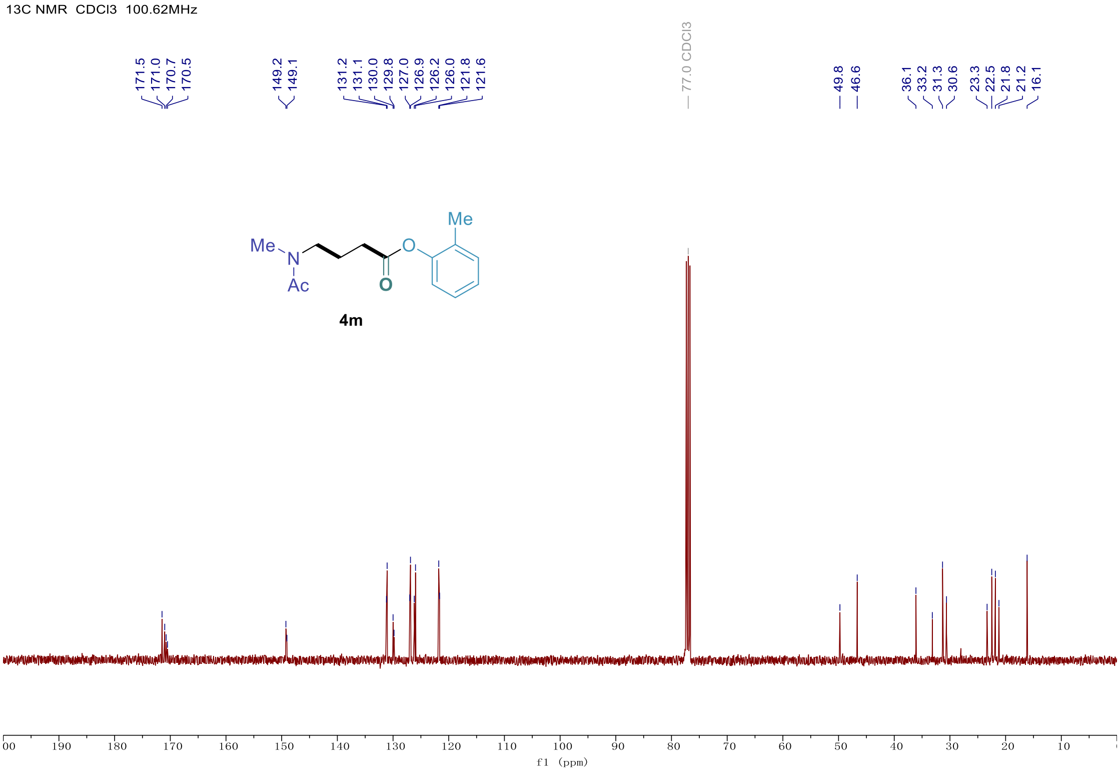

Supplementary Figure 28. ^{13}C NMR of compound **4m** (100 MHz, $CDCl_3$)

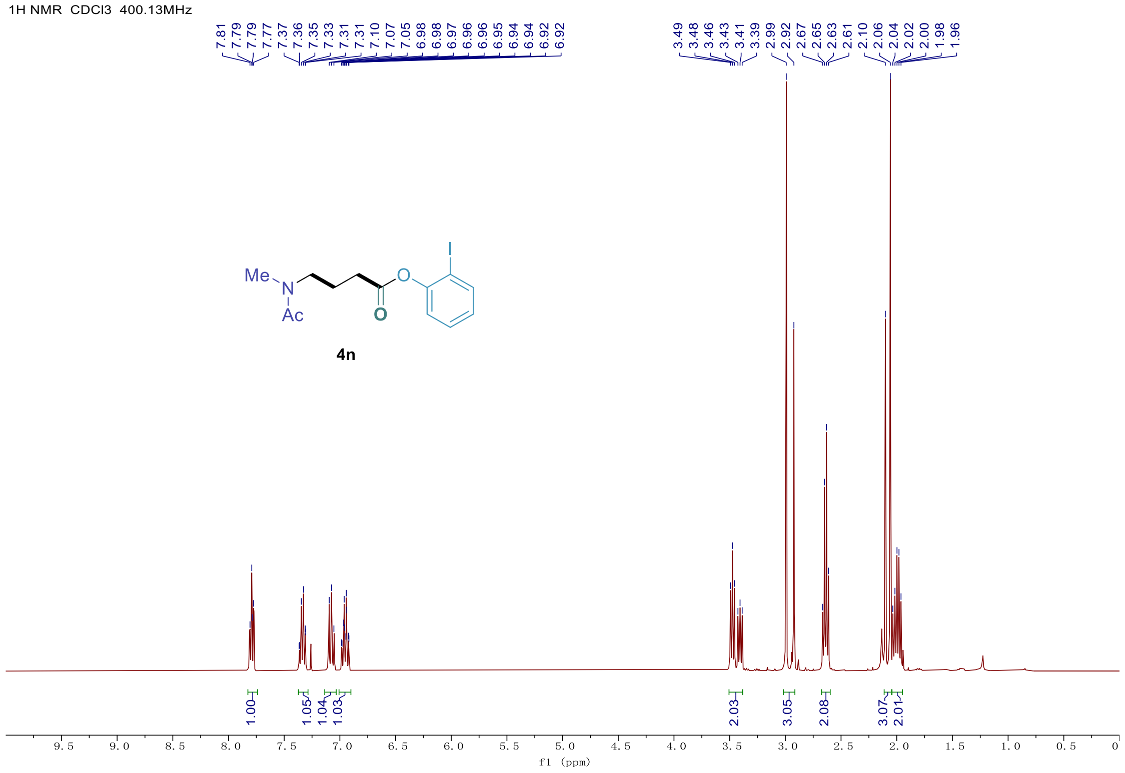

Supplementary Figure 29. ^{1}H NMR of compound **4n** (400 MHz, $CDCl_3$)

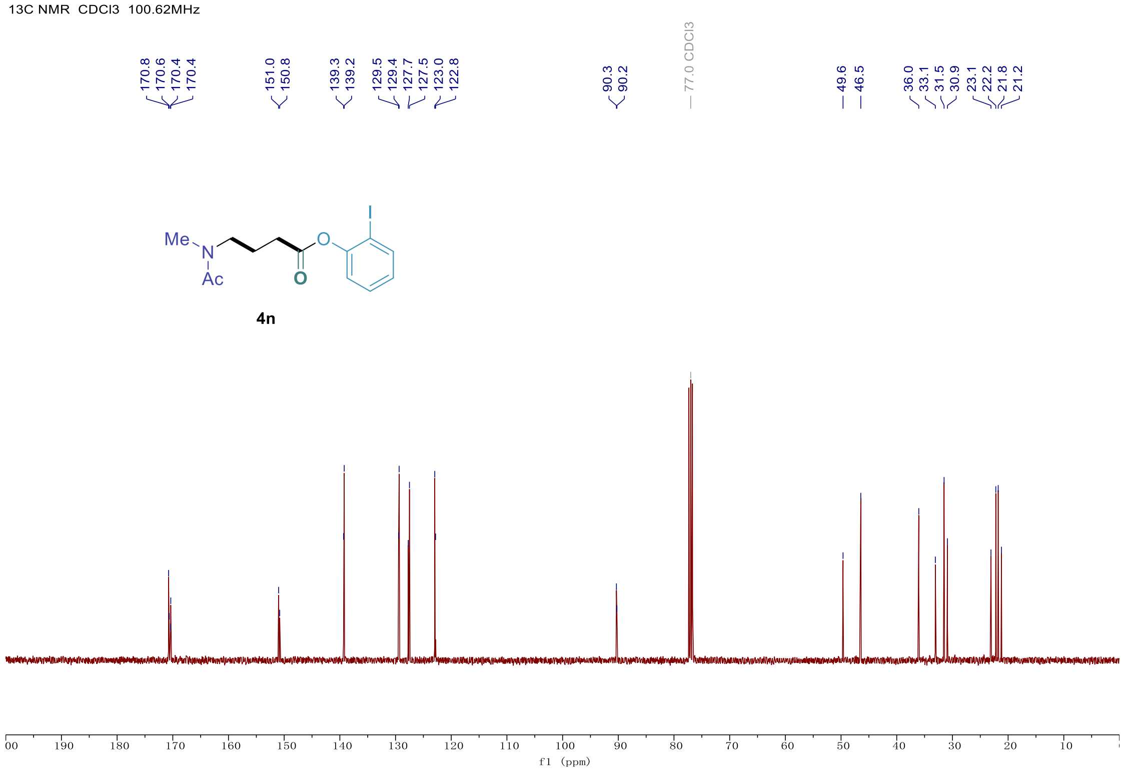

Supplementary Figure 30. ^{13}C NMR of compound **4n** (100 MHz, $CDCl_3$)

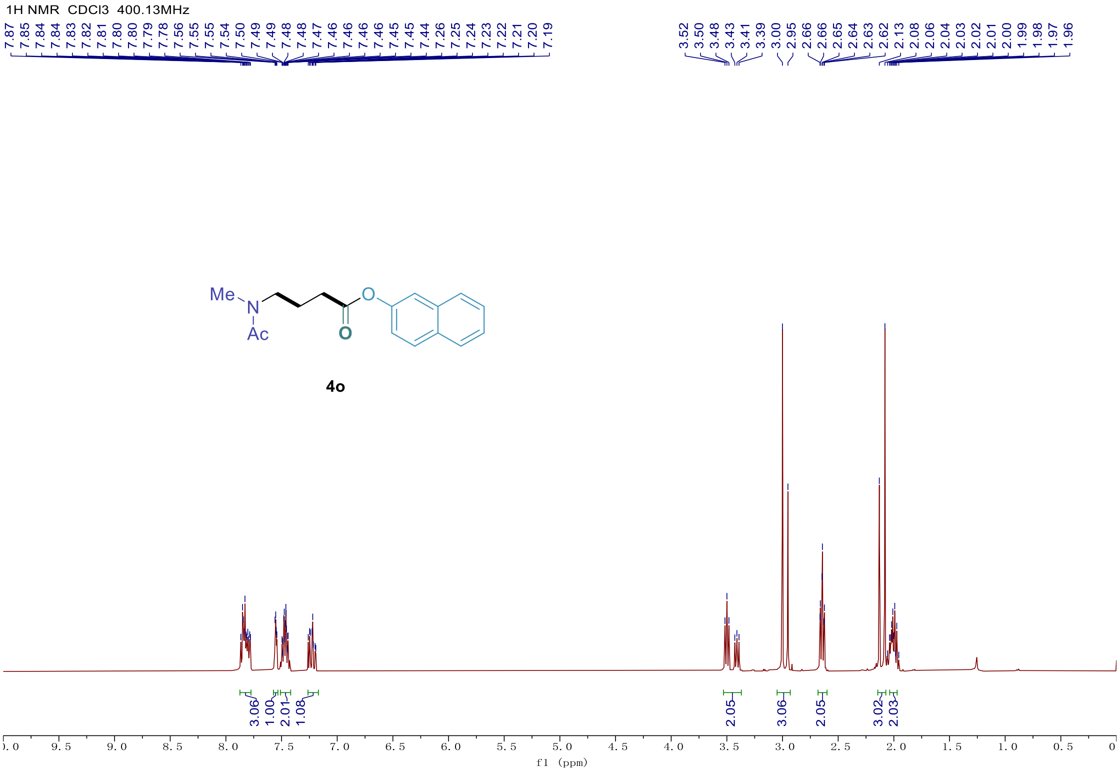

Supplementary Figure 31. ^{1}H NMR of compound **4o** (400 MHz, $CDCl_3$)

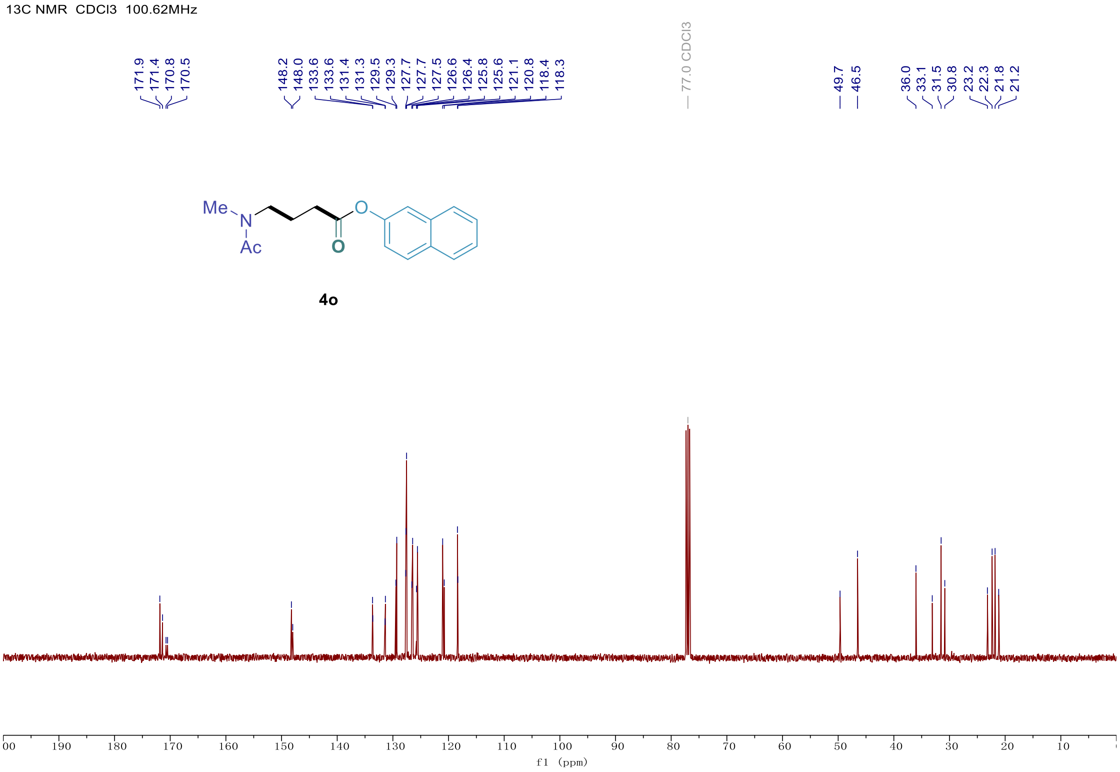

Supplementary Figure 32. ^{13}C NMR of compound **4o** (100 MHz, $CDCl_3$)

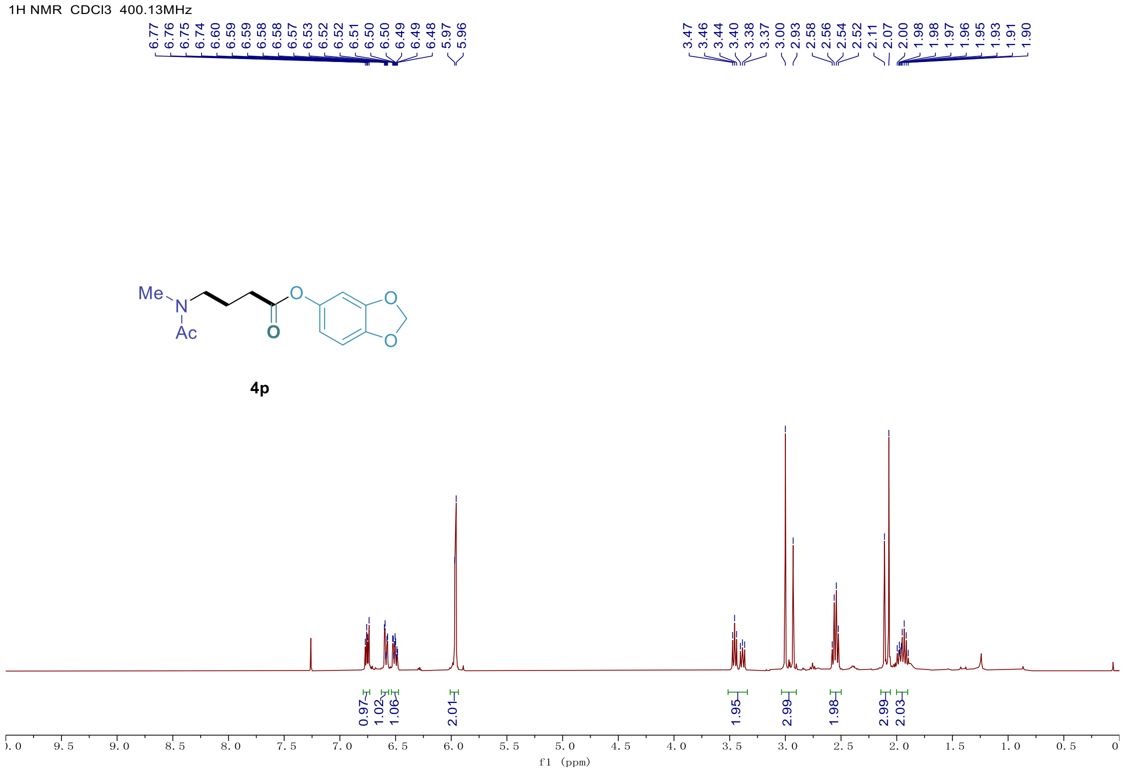

Supplementary Figure 33. ^{1}H NMR of compound **4p** (400 MHz, $CDCl_3$)

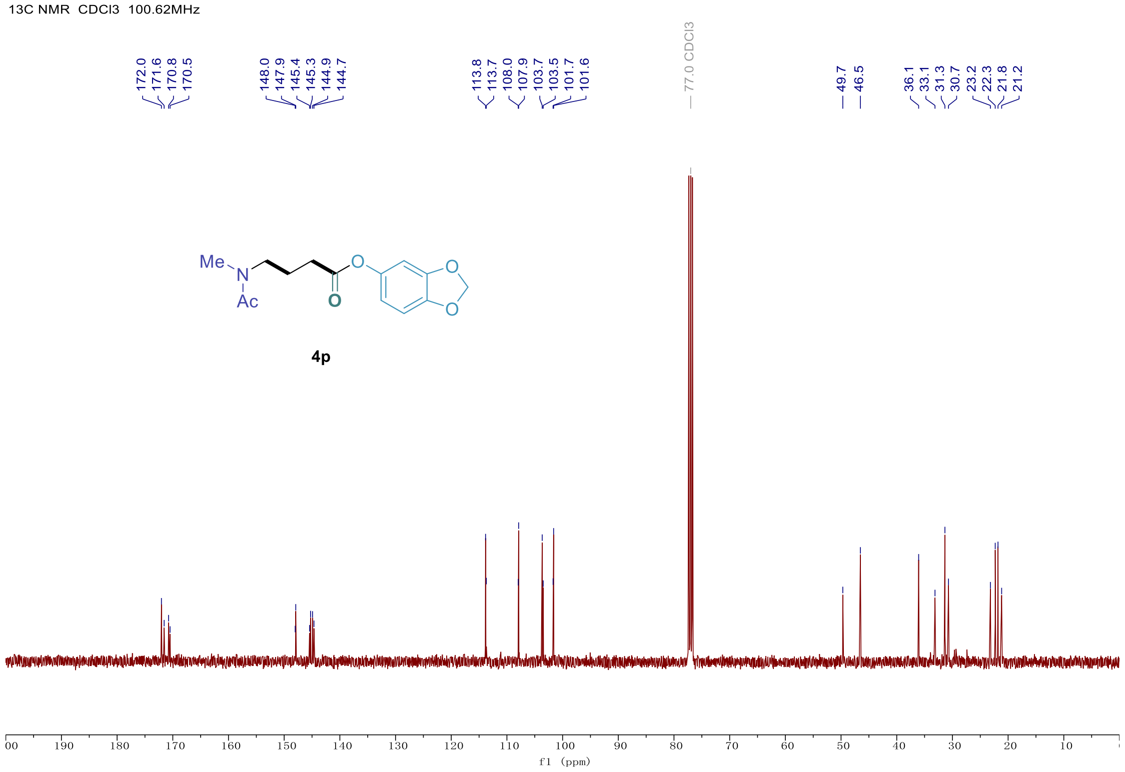

Supplementary Figure 34. ^{13}C NMR of compound **4p** (100 MHz, $CDCl_3$)

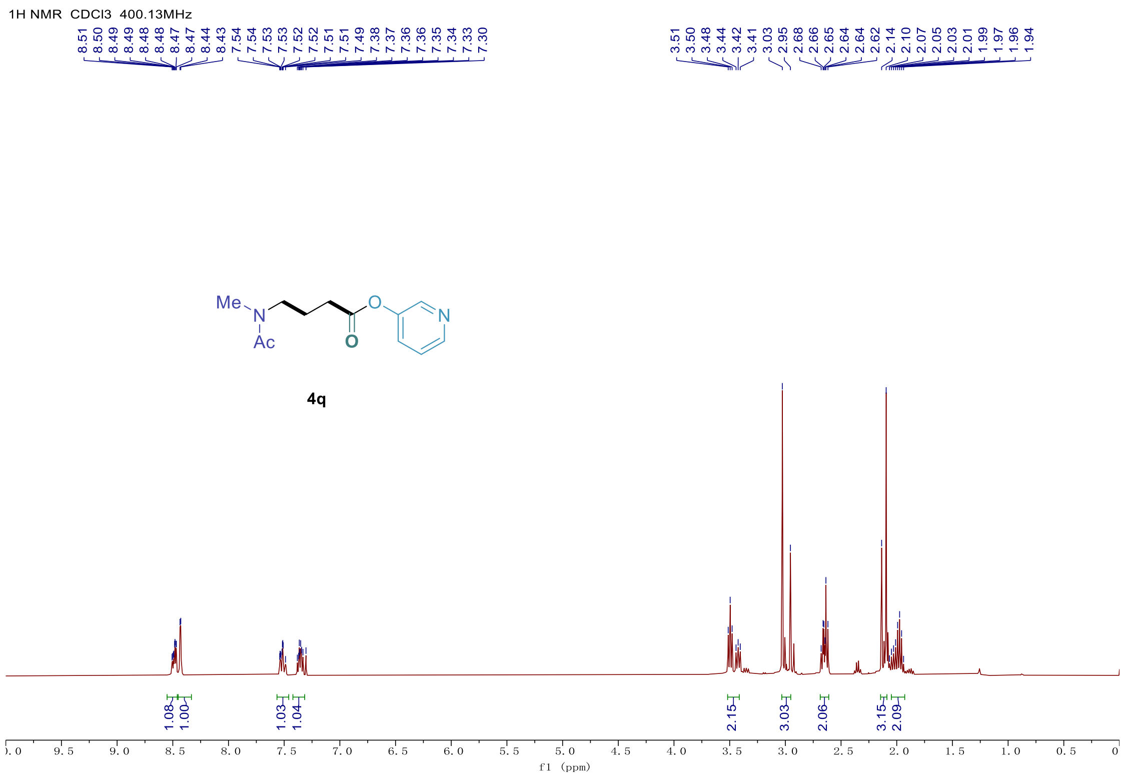

Supplementary Figure 35. ^{1}H NMR of compound **4q** (400 MHz, $CDCl_3$)

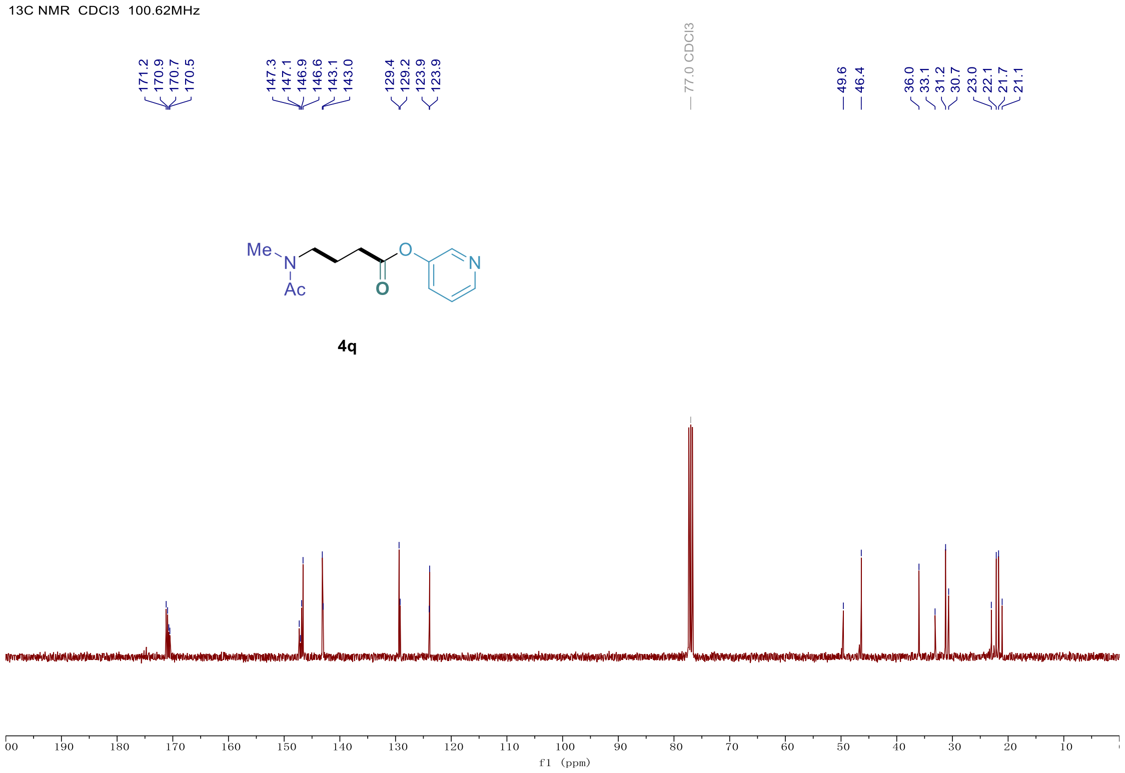

Supplementary Figure 36. ^{13}C NMR of compound **4q** (100 MHz, $CDCl_3$)

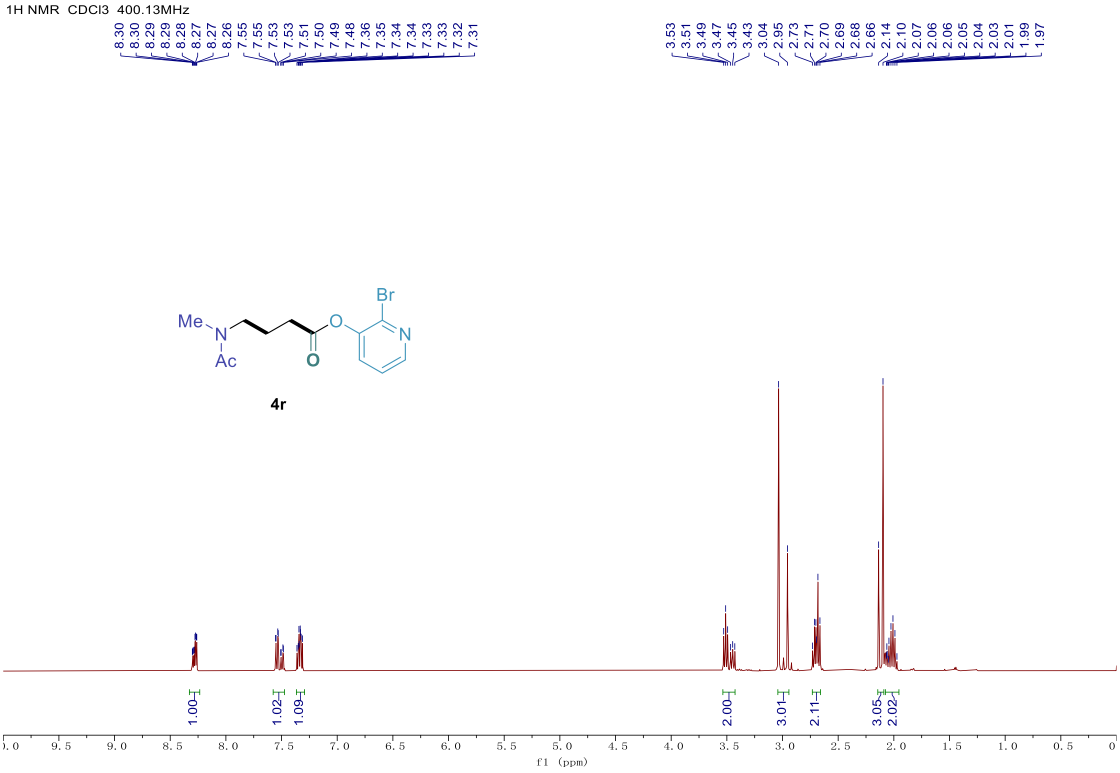

Supplementary Figure 37. ^{1}H NMR of compound **4r** (400 MHz, $CDCl_3$)

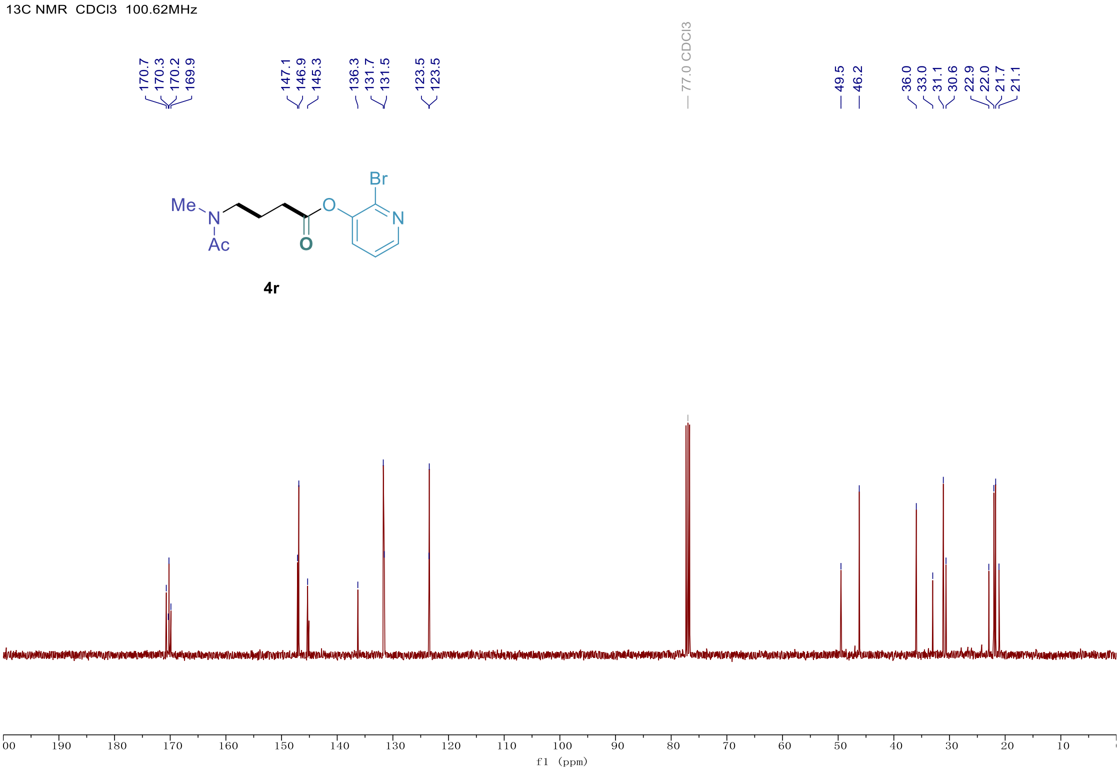

Supplementary Figure 38. ^{13}C NMR of compound **4r** (100 MHz, $CDCl_3$)

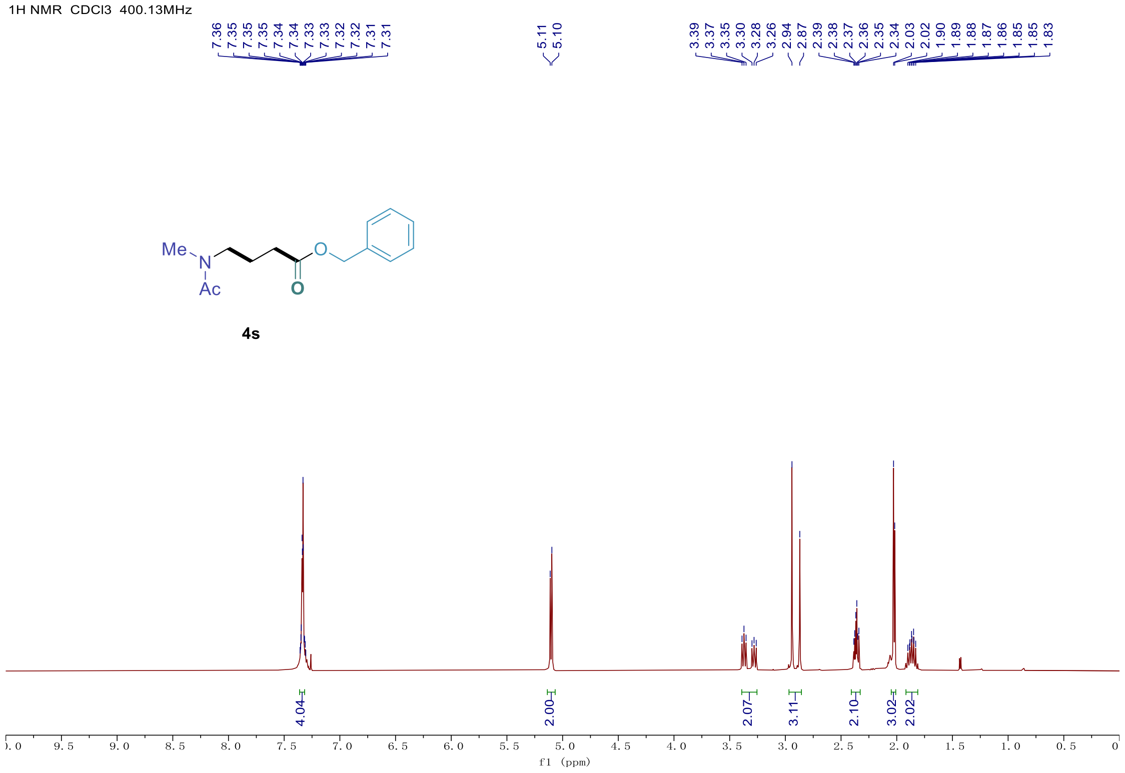

Supplementary Figure 39. ^{1}H NMR of compound **4s** (400 MHz, $CDCl_3$)

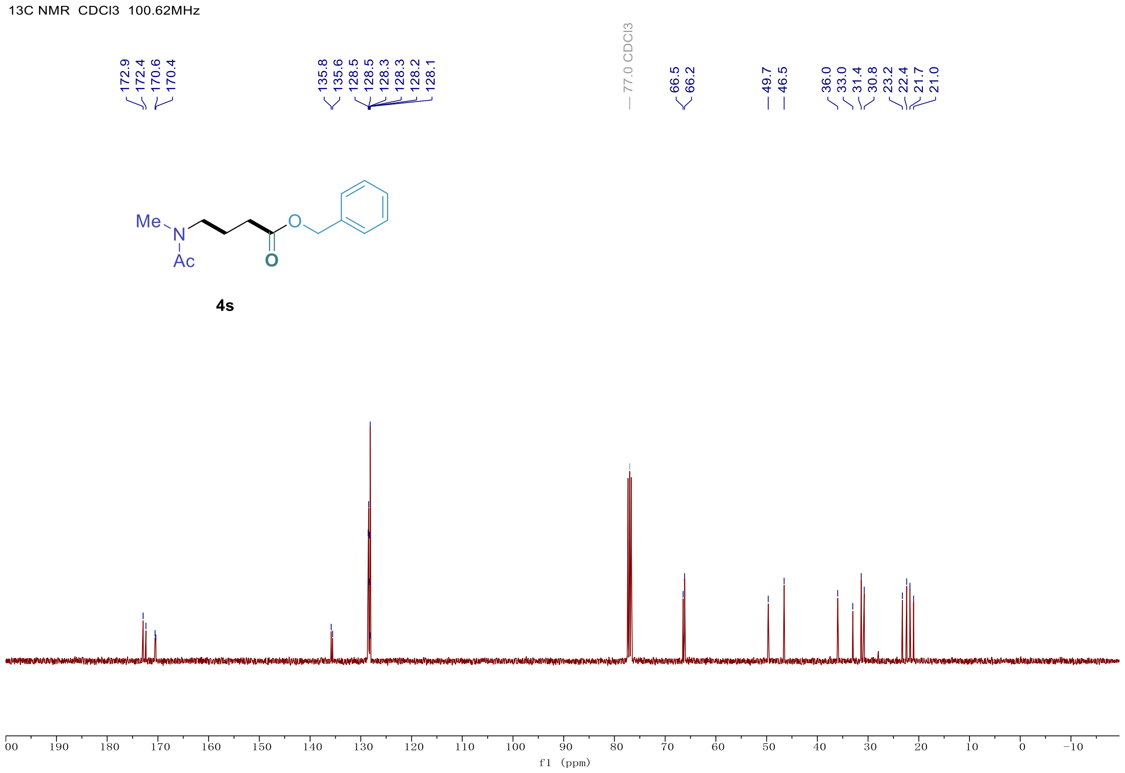

Supplementary Figure 40. ^{13}C NMR of compound **4s** (100 MHz, $CDCl_3$)

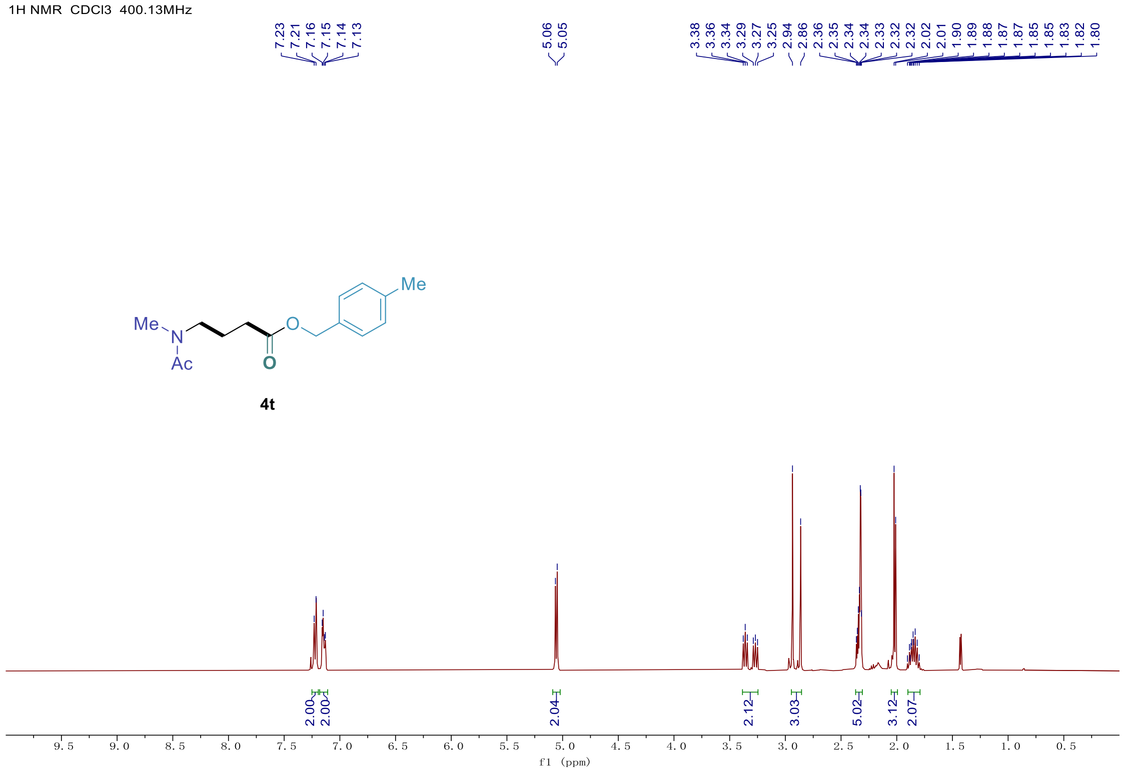

Supplementary Figure 41. ^{1}H NMR of compound **4t** (400 MHz, $CDCl_3$)

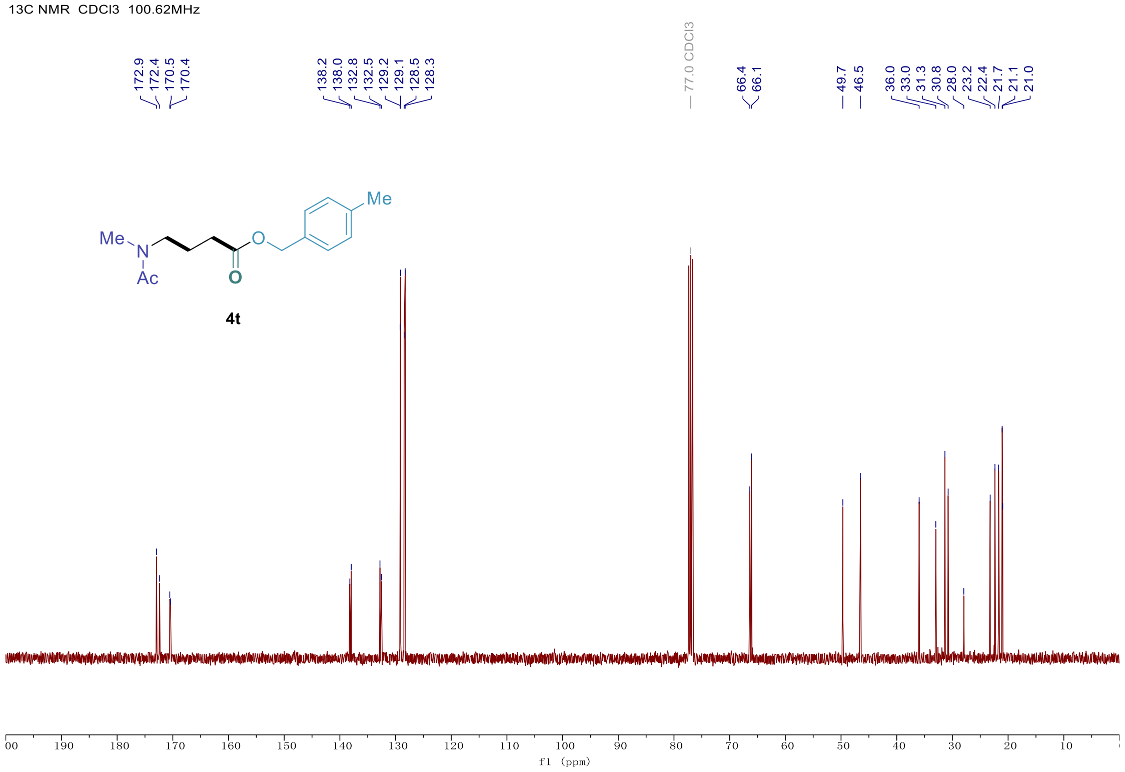

Supplementary Figure 42. ^{13}C NMR of compound **4t** (100 MHz, $CDCl_3$)

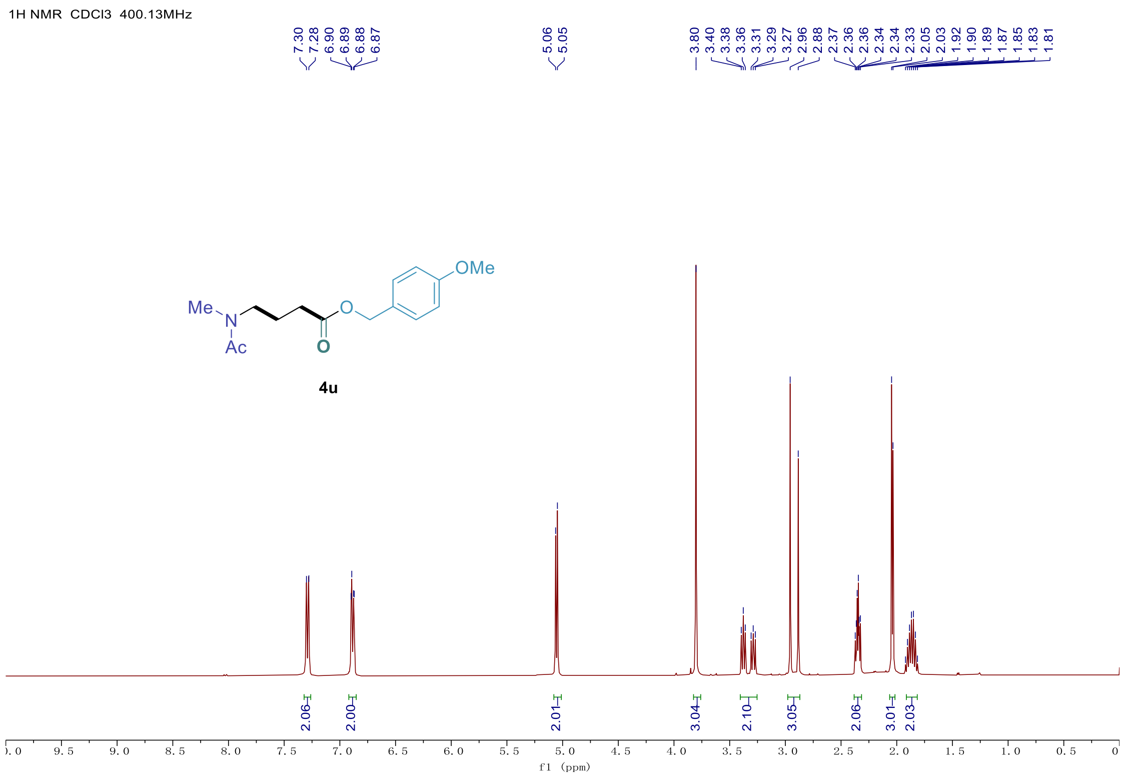

Supplementary Figure 43. ^{1}H NMR of compound **4u** (400 MHz, $CDCl_3$)

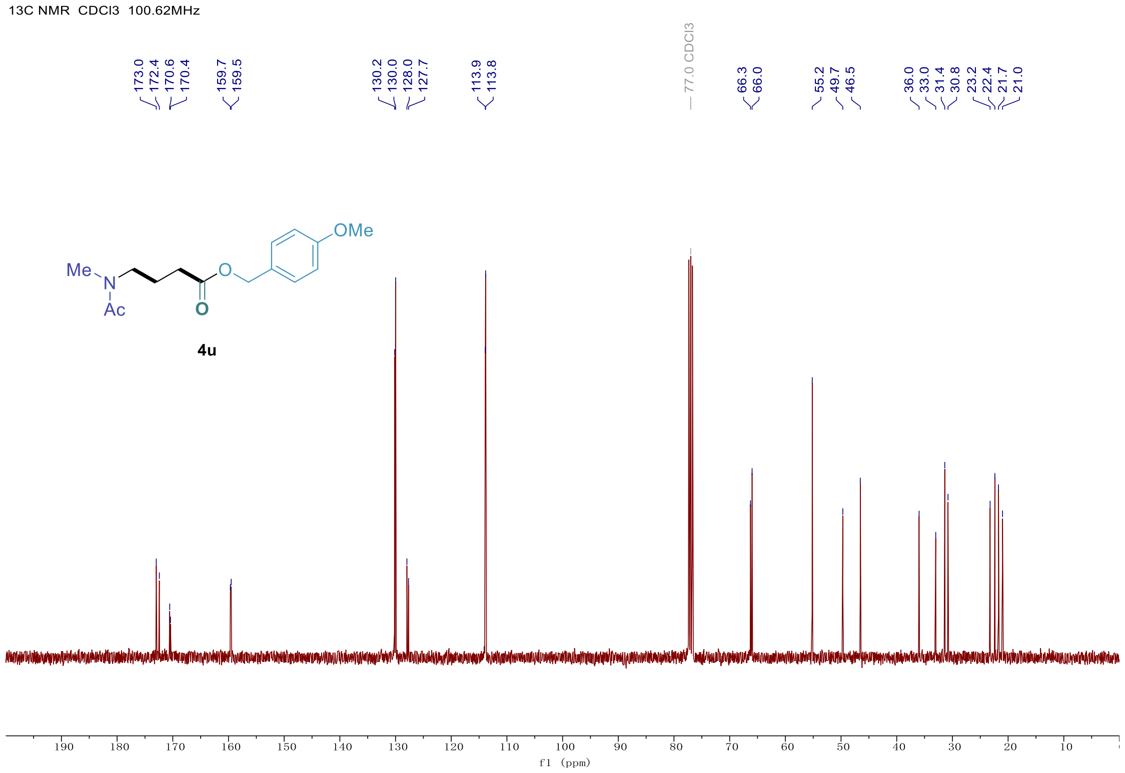

Supplementary Figure 44. ^{13}C NMR of compound **4u** (100 MHz, $CDCl_3$)

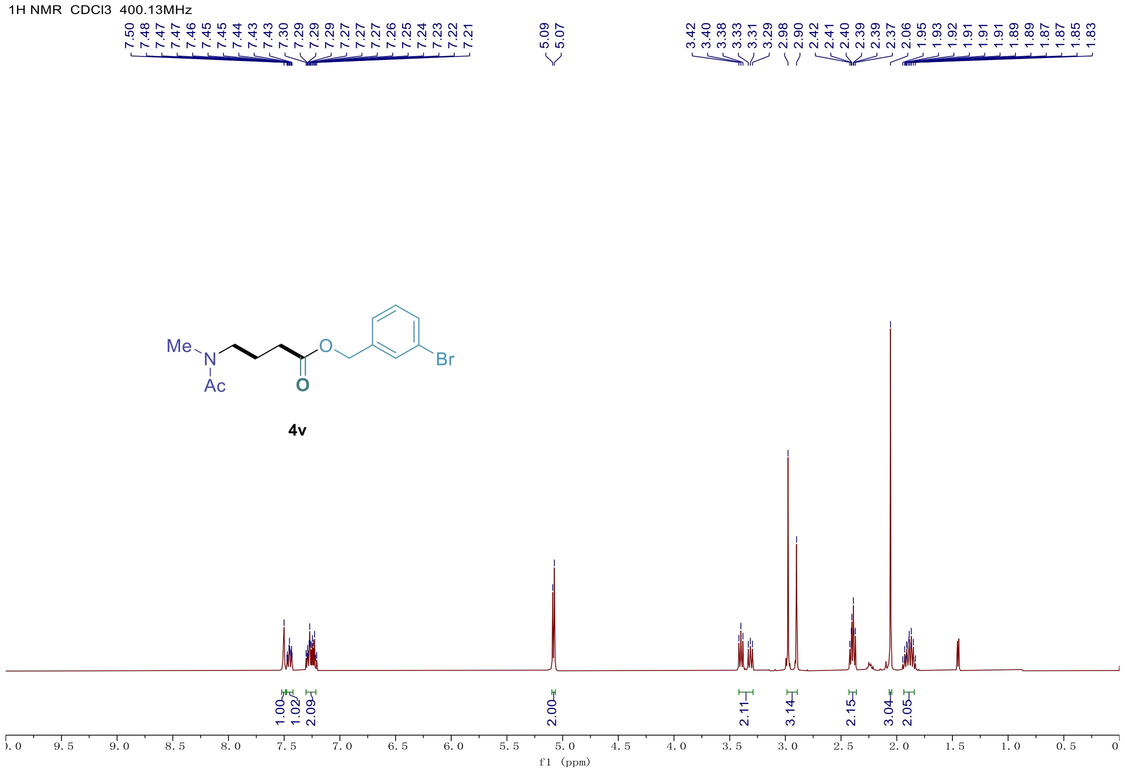

Supplementary Figure 45. ^{1}H NMR of compound **4v** (400 MHz, $CDCl_3$)

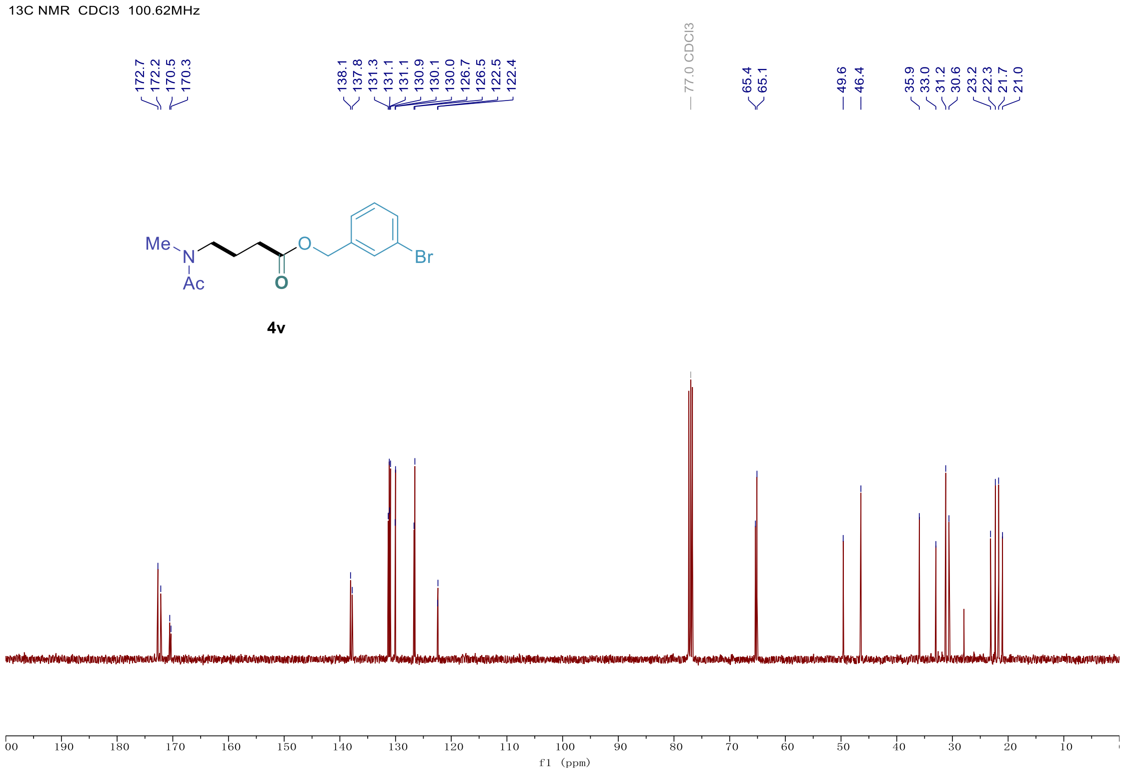

Supplementary Figure 46. ^{13}C NMR of compound **4v** (100 MHz, $CDCl_3$)

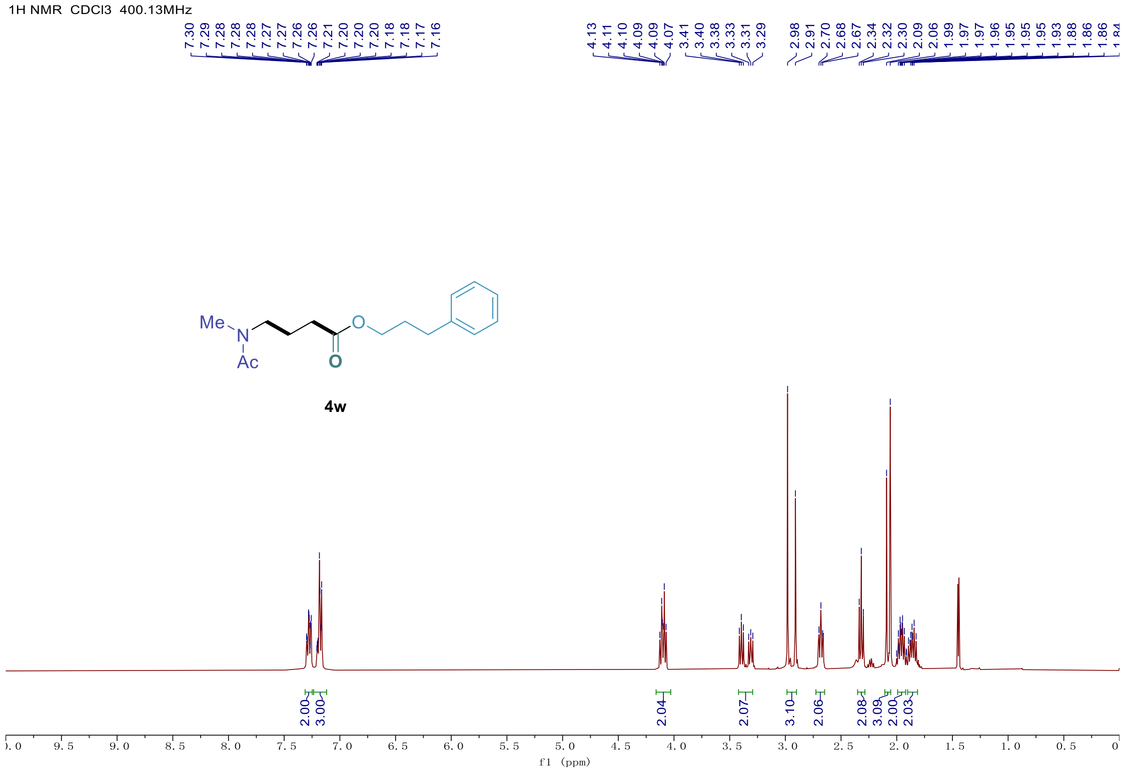

Supplementary Figure 47. ^{1}H NMR of compound **4w** (400 MHz, $CDCl_3$)

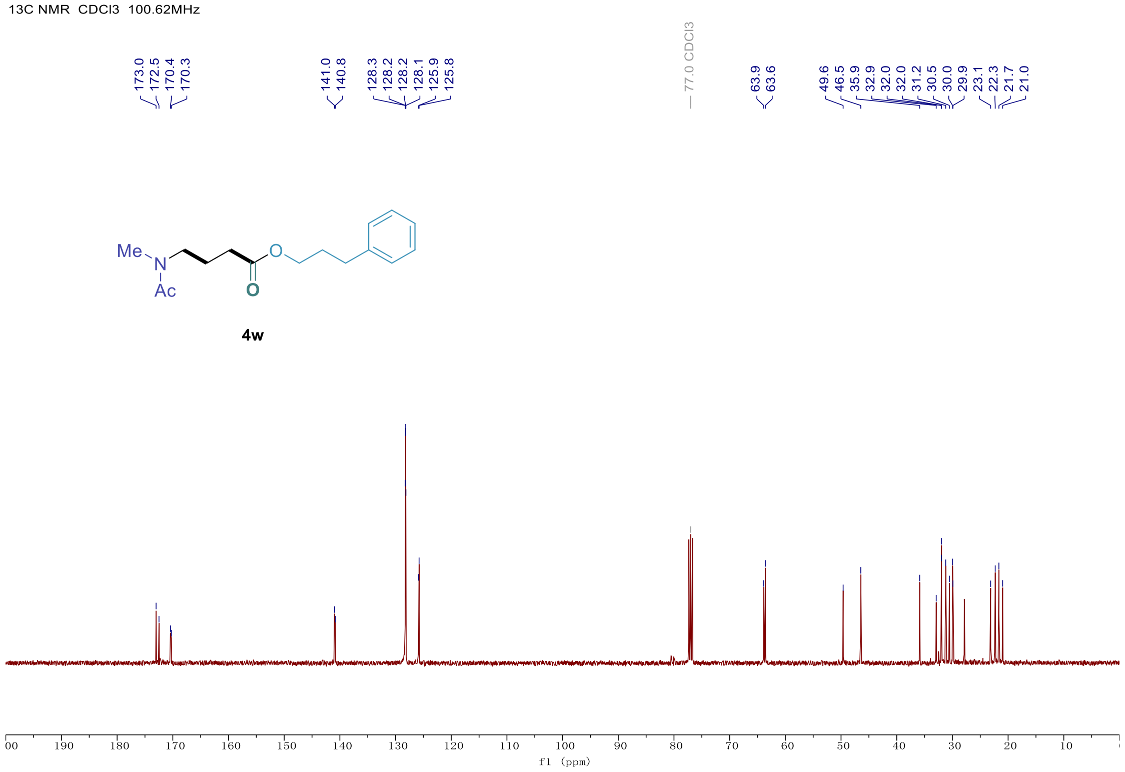

Supplementary Figure 48. ^{13}C NMR of compound **4w** (100 MHz, $CDCl_3$)

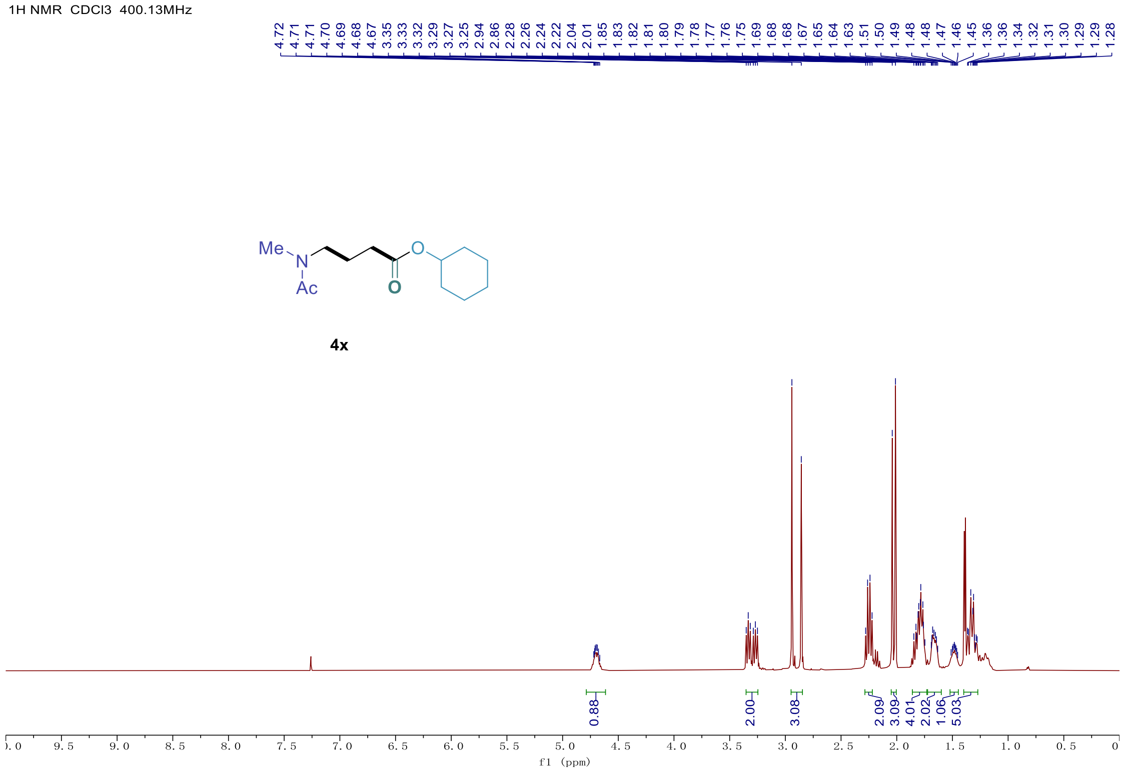

Supplementary Figure 49. ^{1}H NMR of compound **4x** (400 MHz, $CDCl_3$)

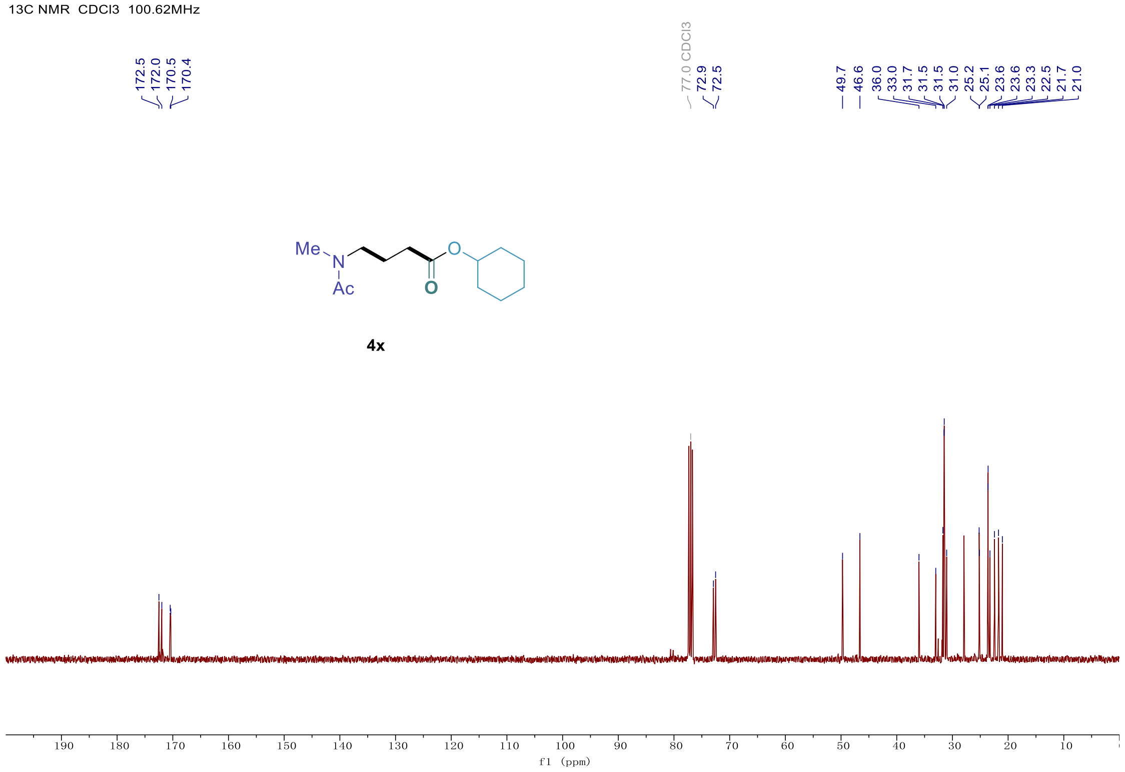

Supplementary Figure 50. ^{13}C NMR of compound **4x** (100 MHz, $CDCl_3$)

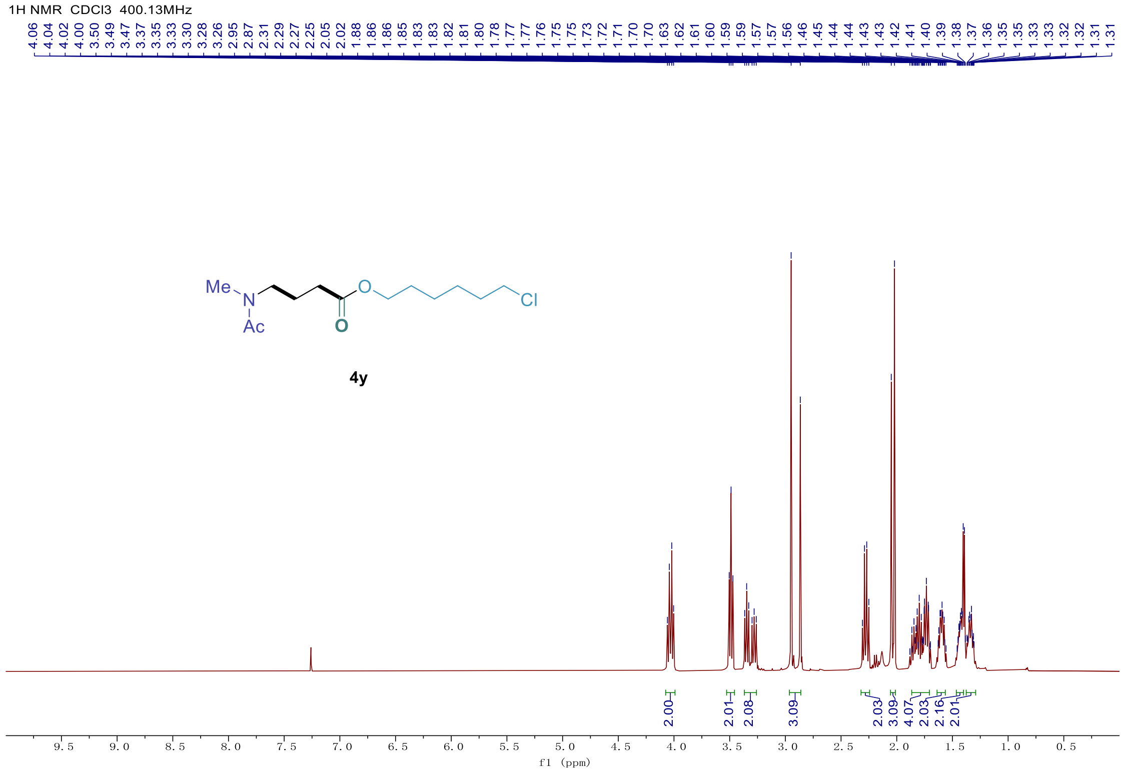

Supplementary Figure 51. ^{1}H NMR of compound **4y** (400 MHz, $CDCl_3$)

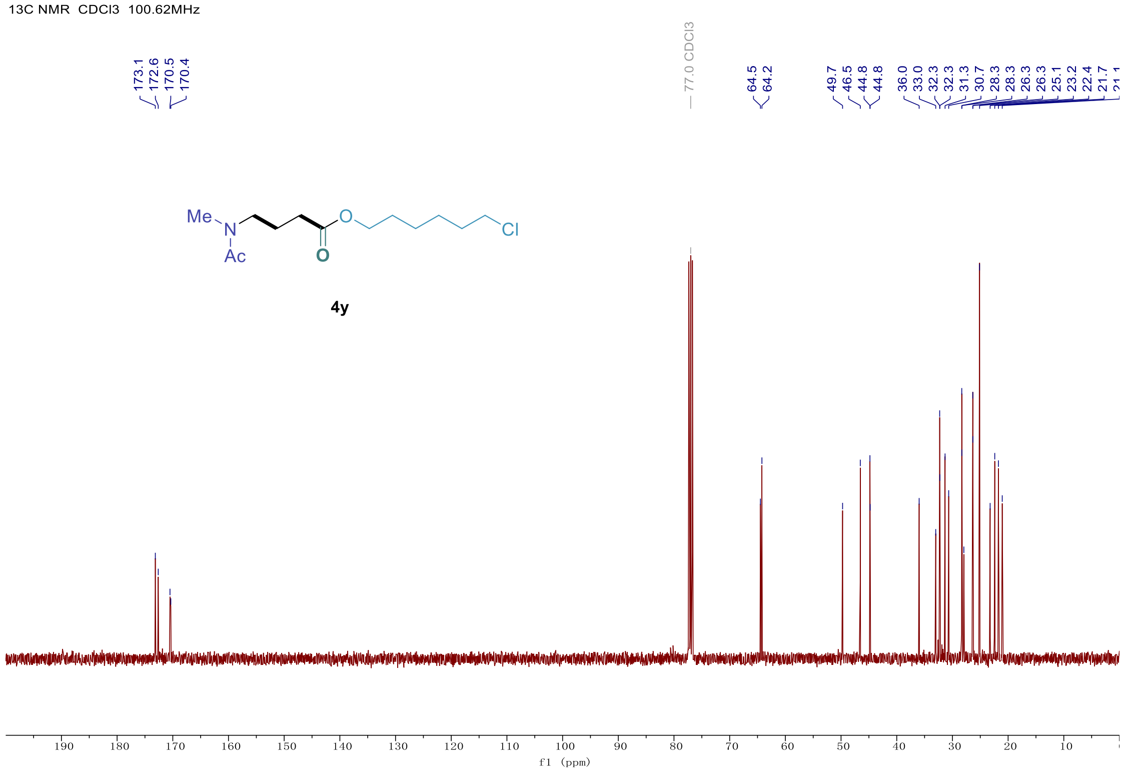

Supplementary Figure 52. ^{13}C NMR of compound **4y** (100 MHz, $CDCl_3$)

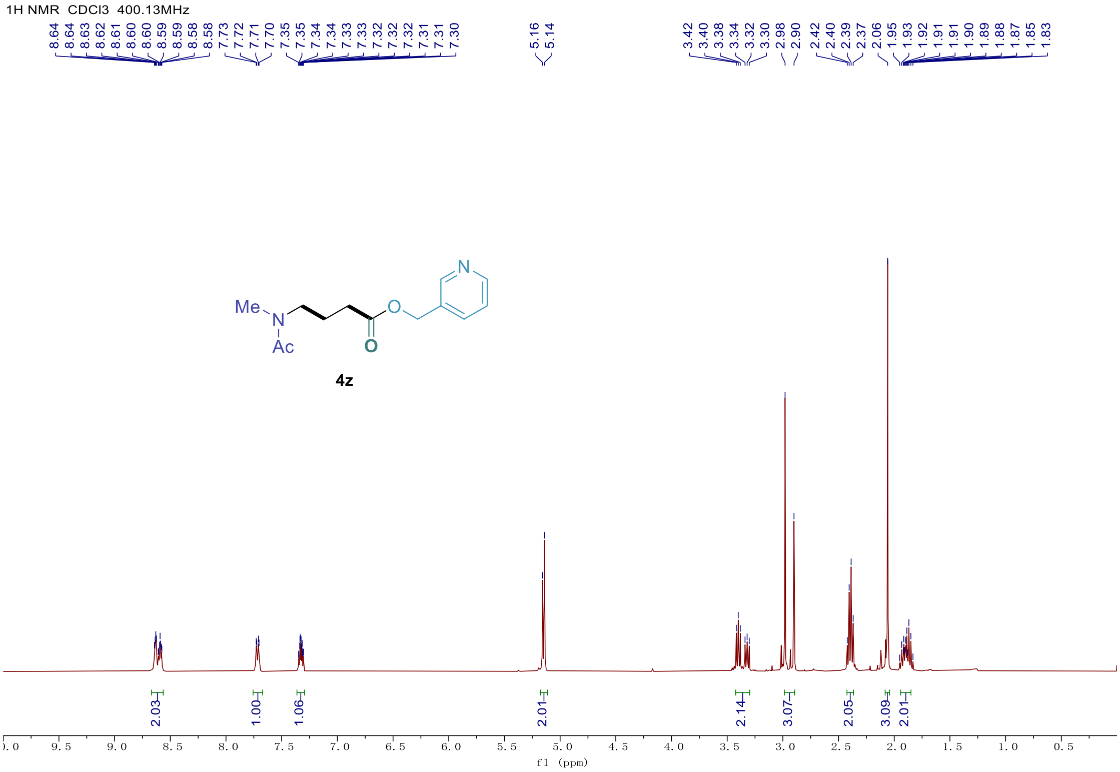

Supplementary Figure 53. ^{1}H NMR of compound **4z** (400 MHz, $CDCl_3$)

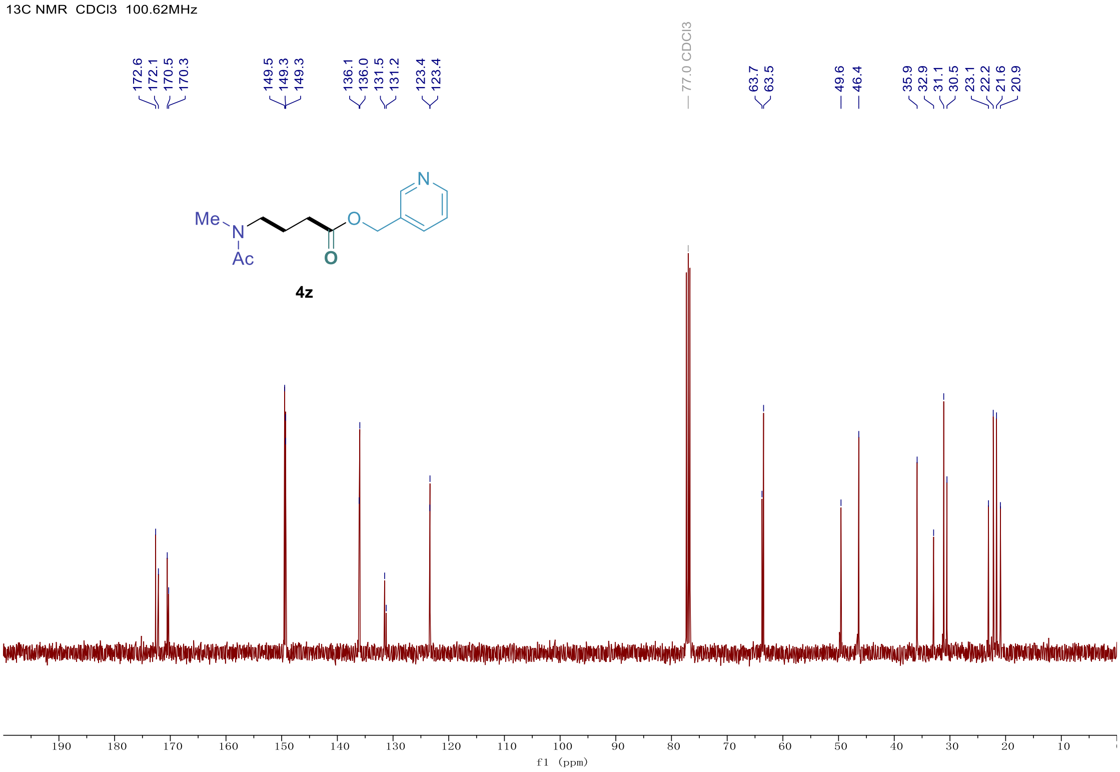

Supplementary Figure 54. ^{13}C NMR of compound **4z** (100 MHz, $CDCl_3$)

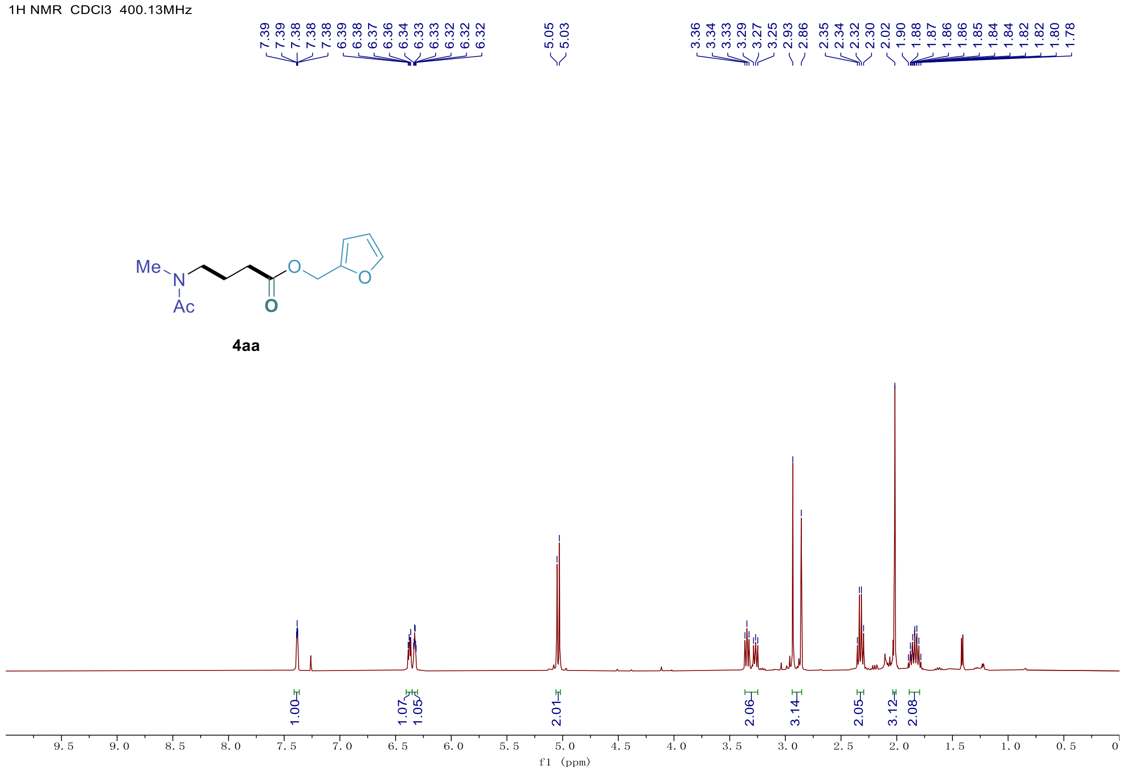

Supplementary Figure 55. ^{1}H NMR of compound **4aa** (400 MHz, $CDCl_3$)

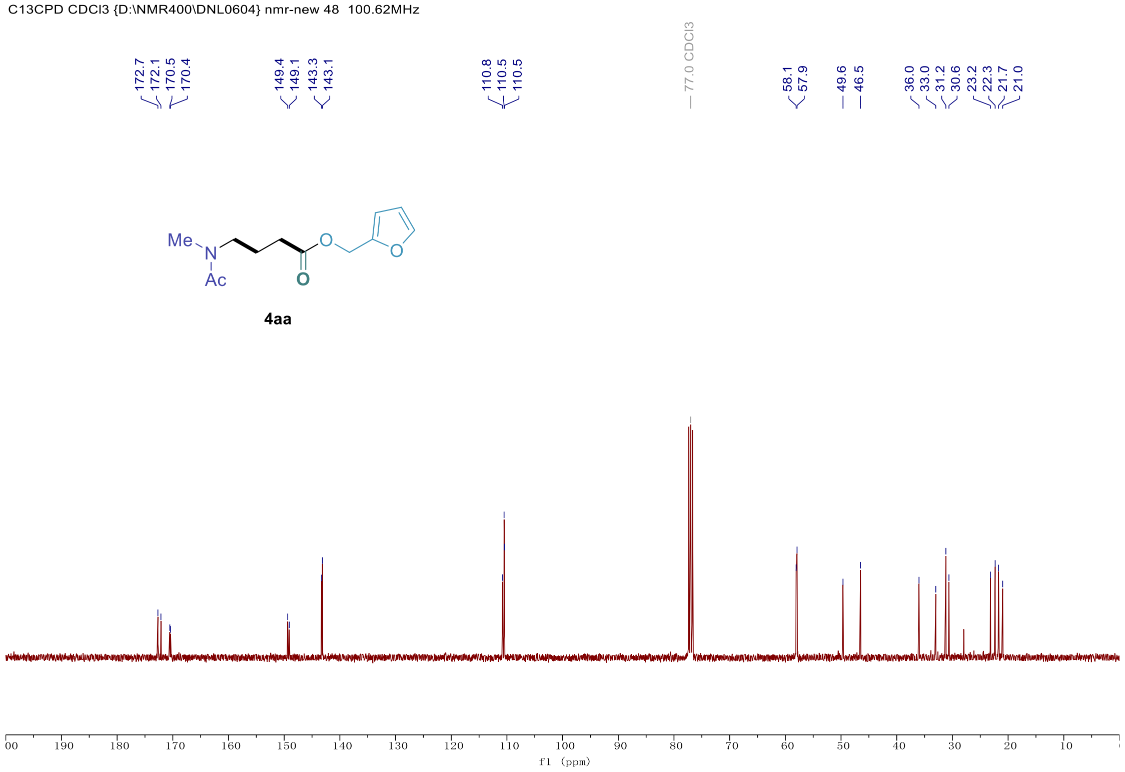

Supplementary Figure 56. ^{13}C NMR of compound **4aa** (100 MHz, $CDCl_3$)

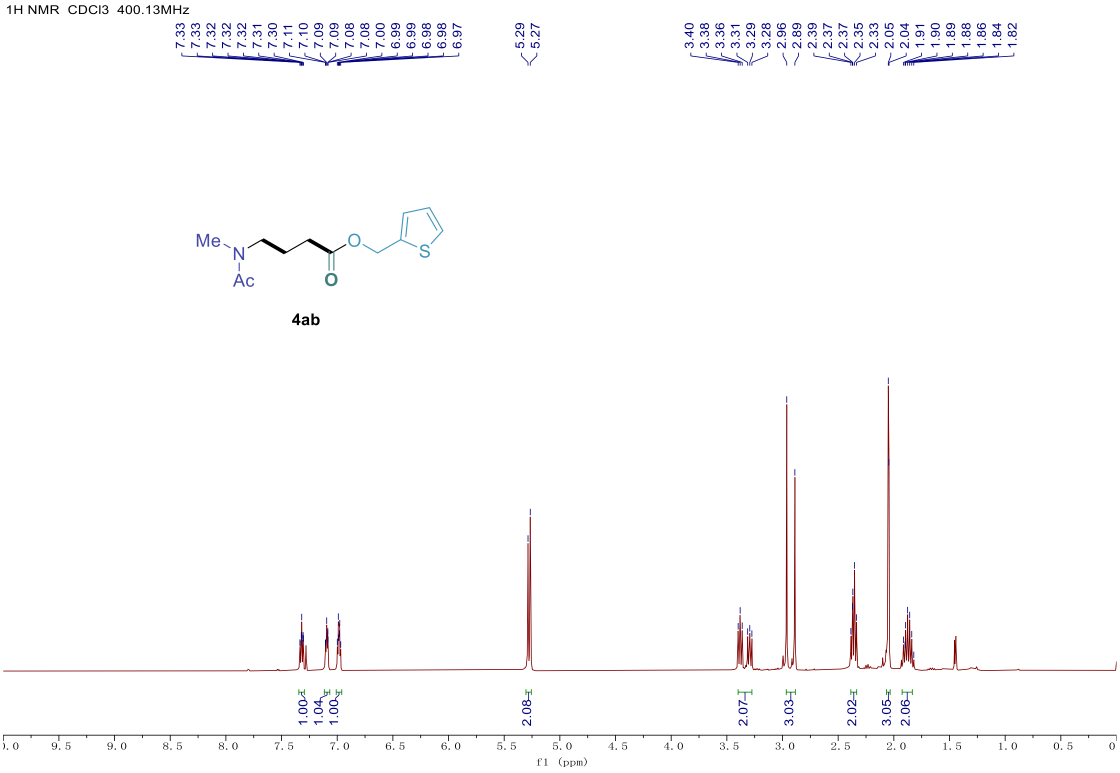

Supplementary Figure 57 ^{1}H NMR of compound **4ab** (400 MHz, $CDCl_3$)

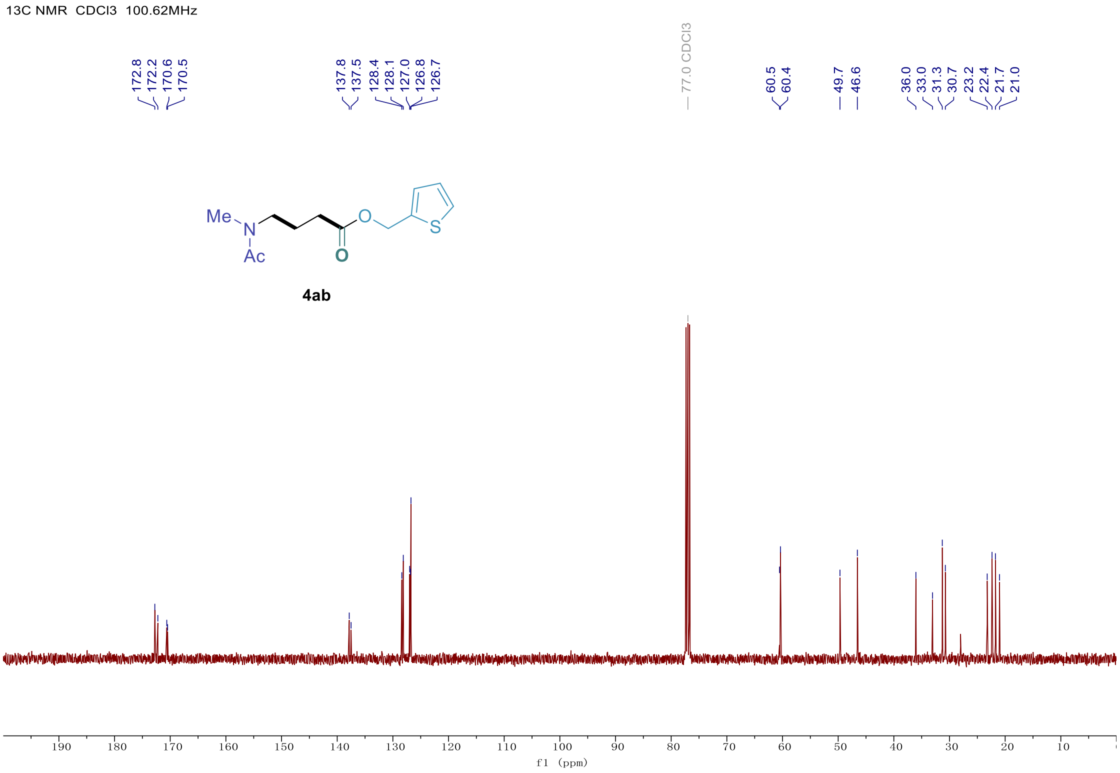

Supplementary Figure 58. ^{13}C NMR of compound **4ab** (100 MHz, $CDCl_3$)

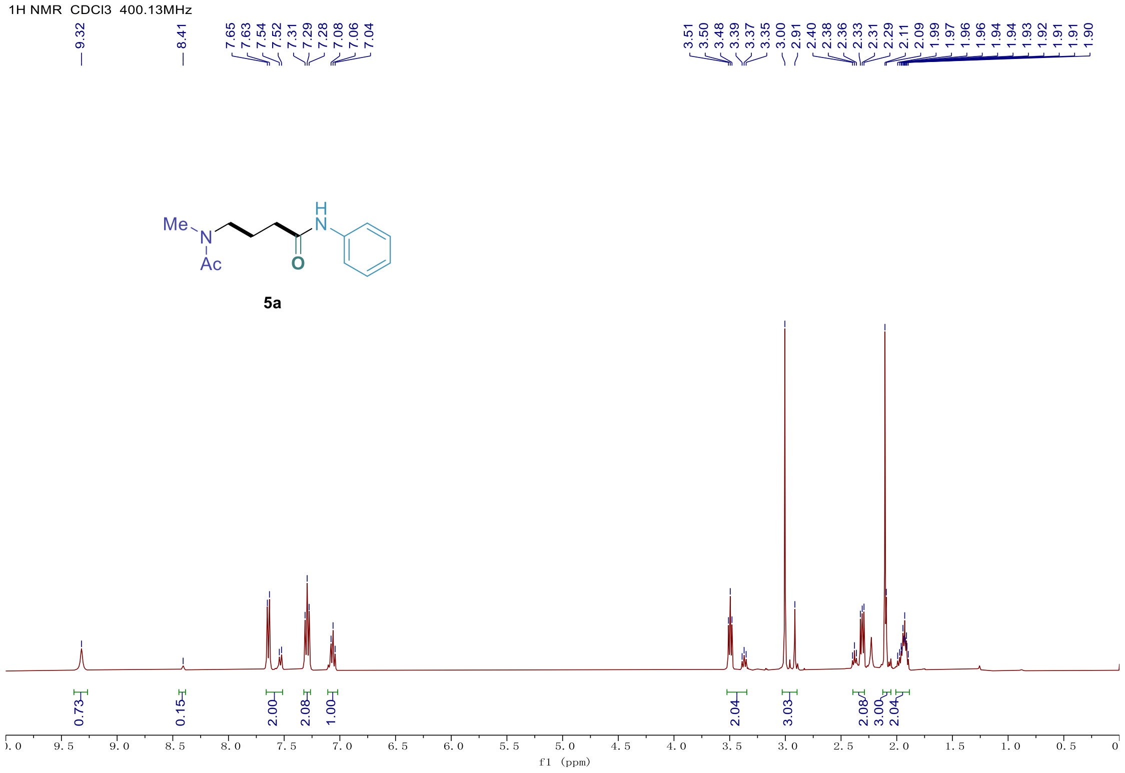

Supplementary Figure 59. ^{1}H NMR of compound **5a** (400 MHz, $CDCl_3$)

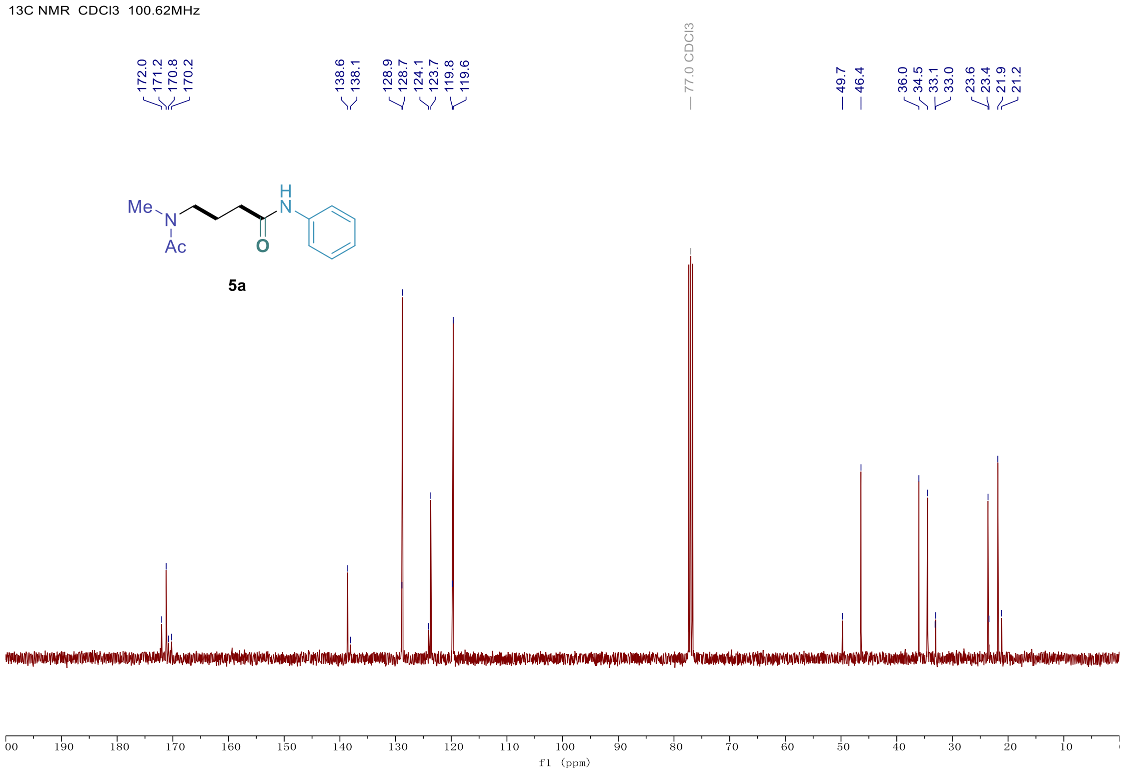

Supplementary Figure 60. ^{13}C NMR of compound **5a** (100 MHz, $CDCl_3$)

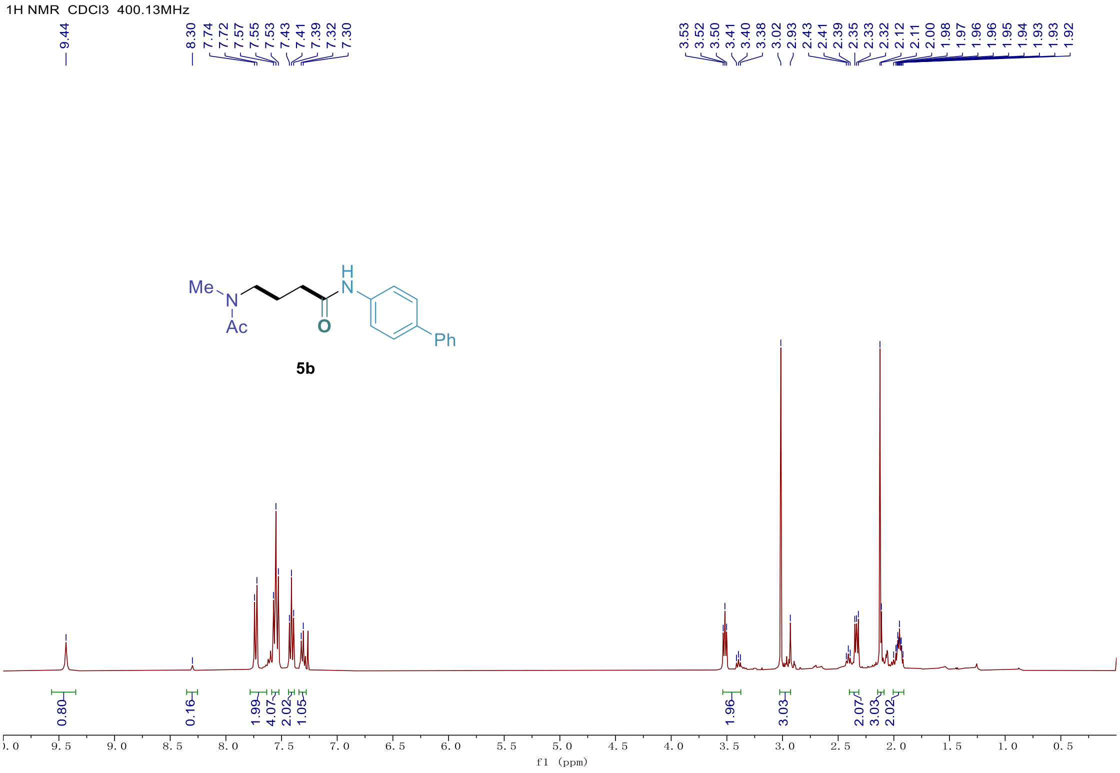

Supplementary Figure 61. ^{1}H NMR of compound **5b** (400 MHz, $CDCl_3$)

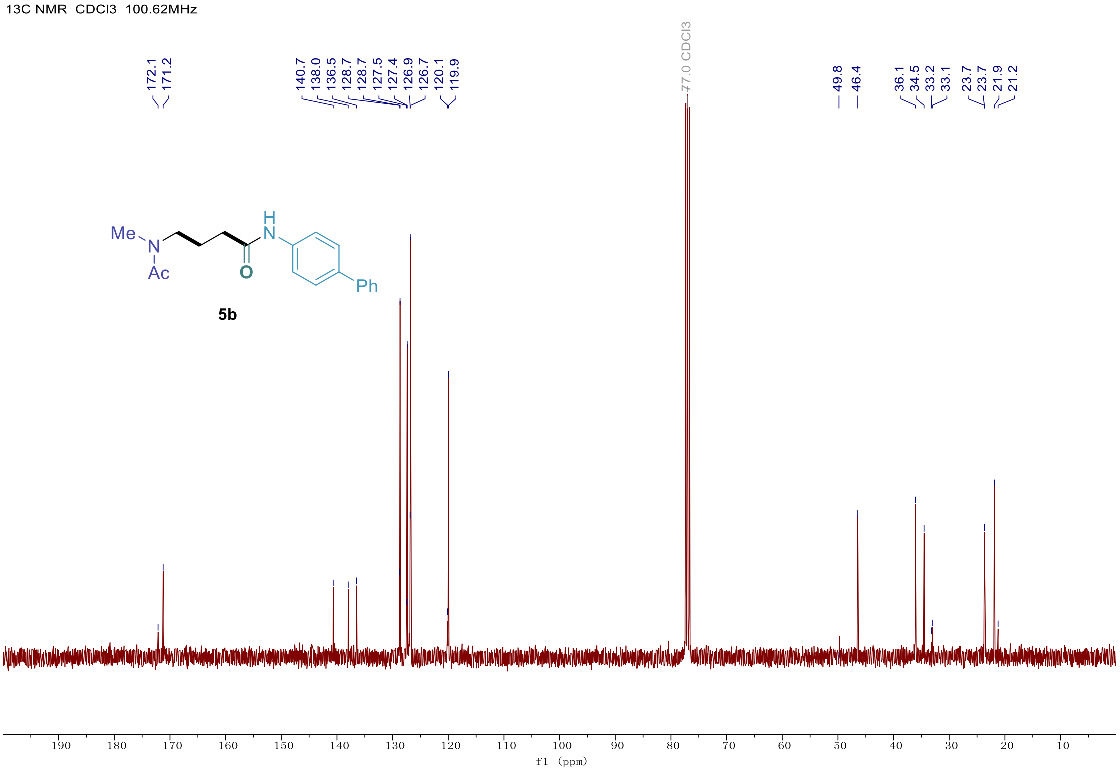

Supplementary Figure 62. ^{13}C NMR of compound **5b** (100 MHz, $CDCl_3$)

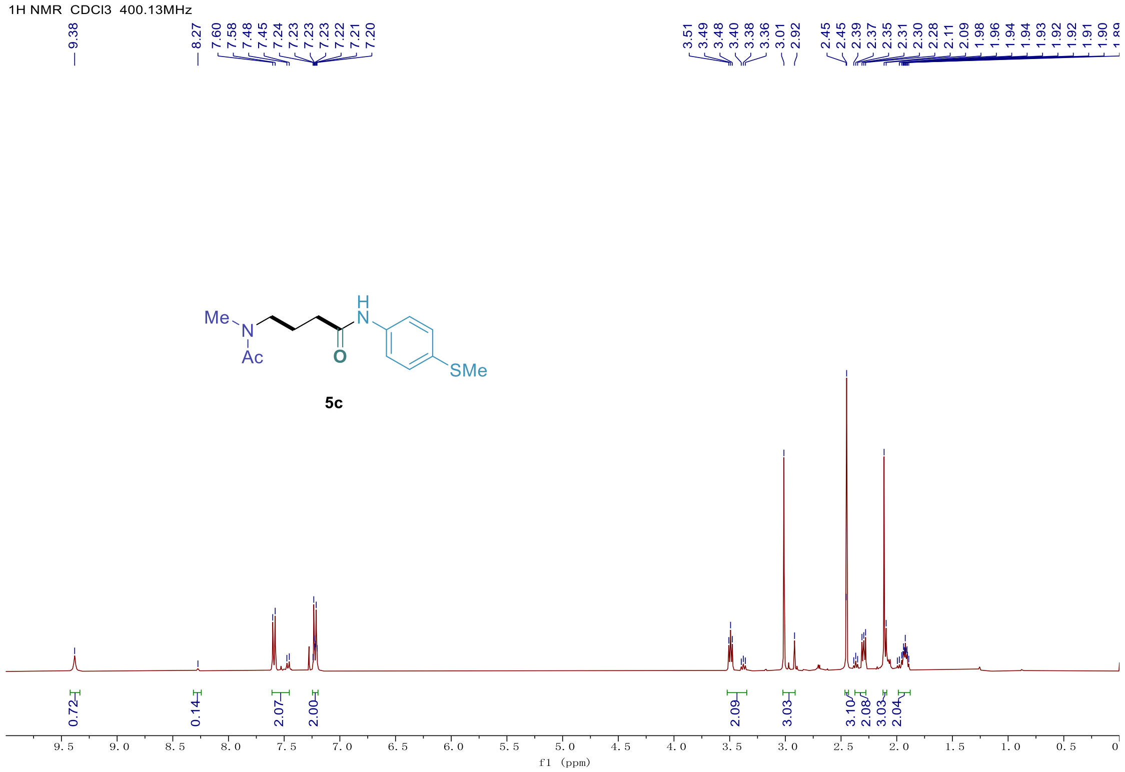

Supplementary Figure 63. ^{1}H NMR of compound **5c** (400 MHz, $CDCl_3$)

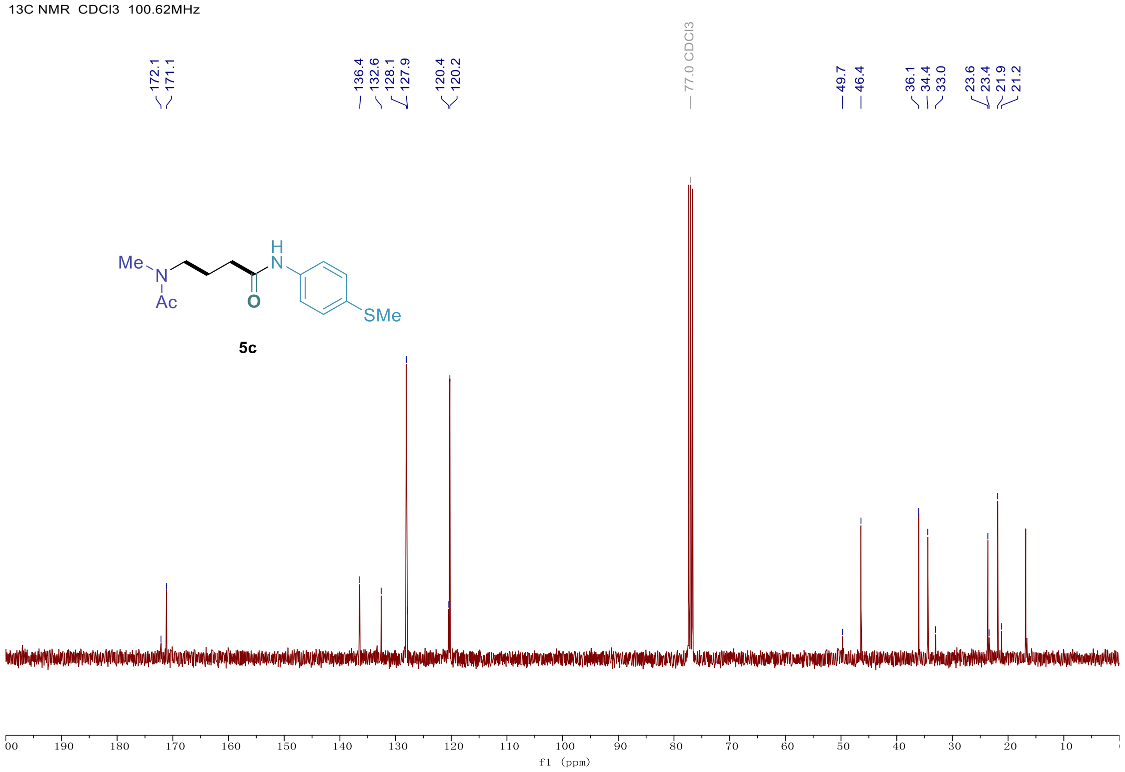

Supplementary Figure 64. ^{13}C NMR of compound **5c** (100 MHz, $CDCl_3$)

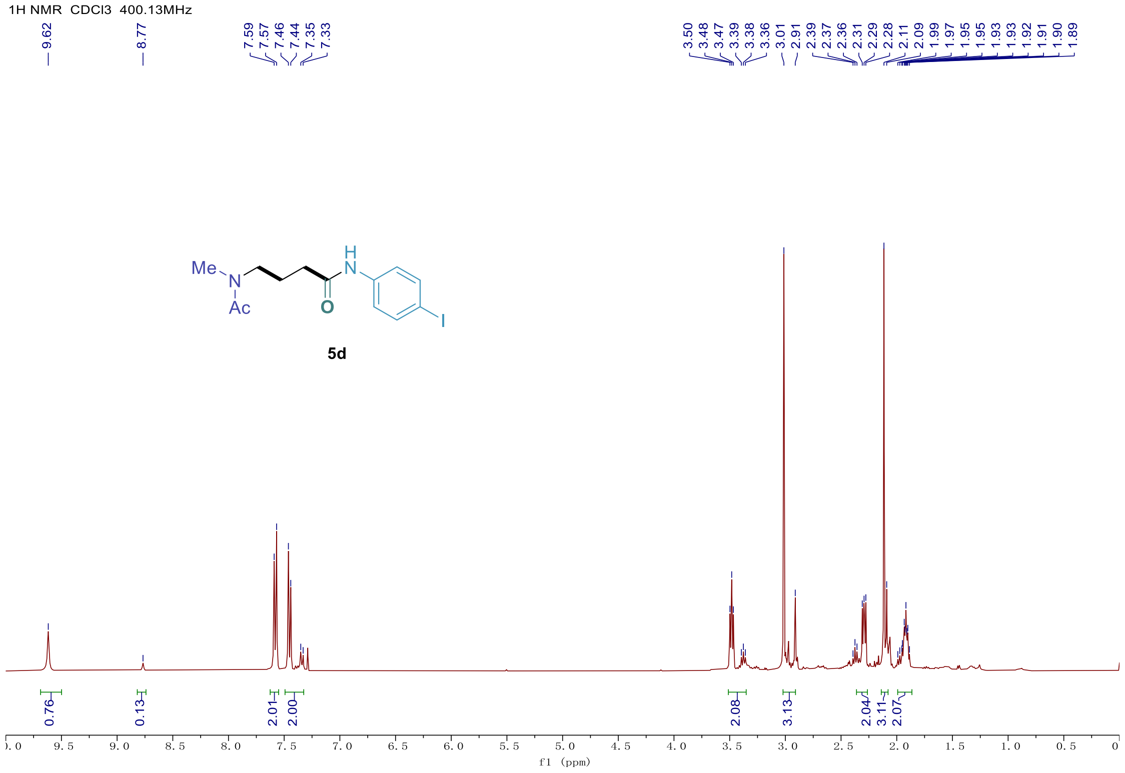

Supplementary Figure 65. ^{1}H NMR of compound **5d** (400 MHz, $CDCl_3$)

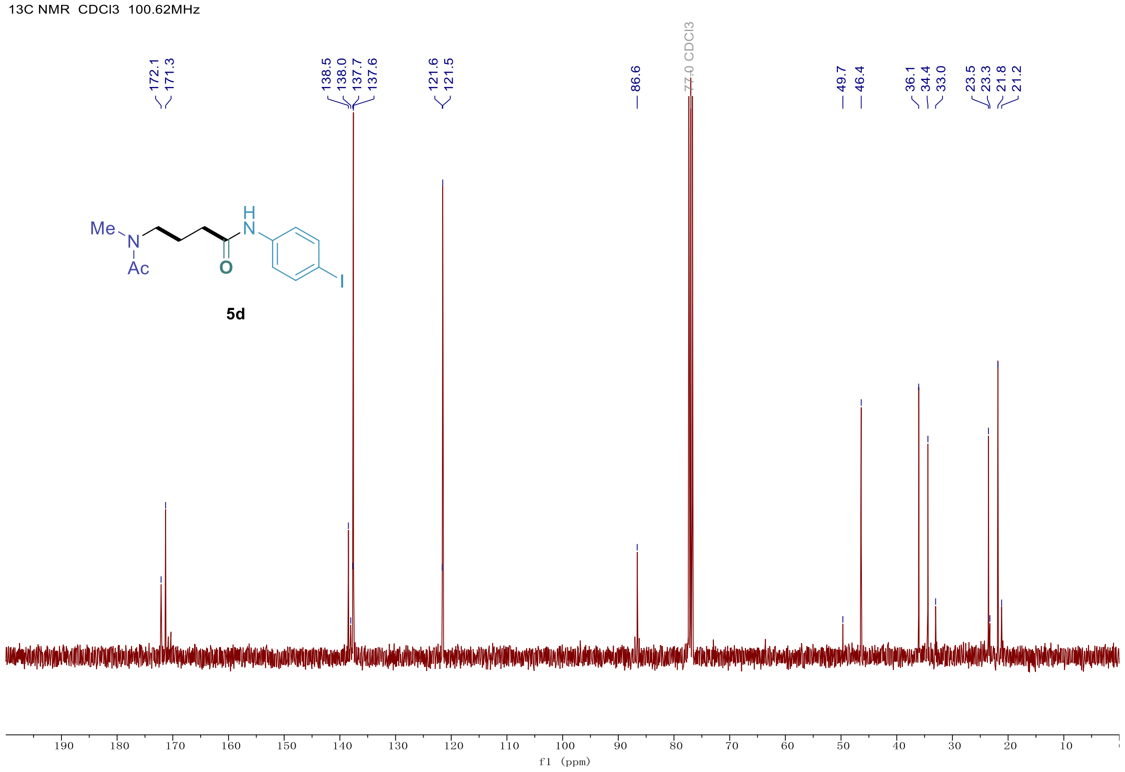

Supplementary Figure 66. ^{13}C NMR of compound **5d** (100 MHz, $CDCl_3$)

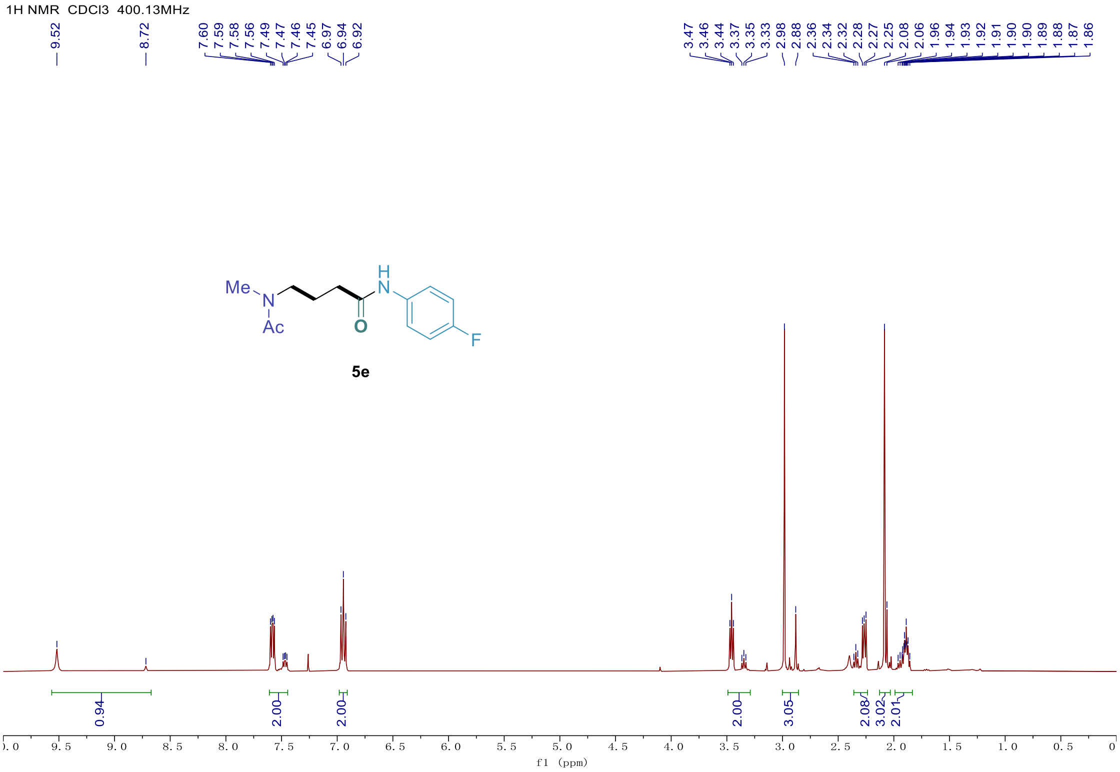

Supplementary Figure 67. ^{1}H NMR of compound **5e** (400 MHz, $CDCl_3$)

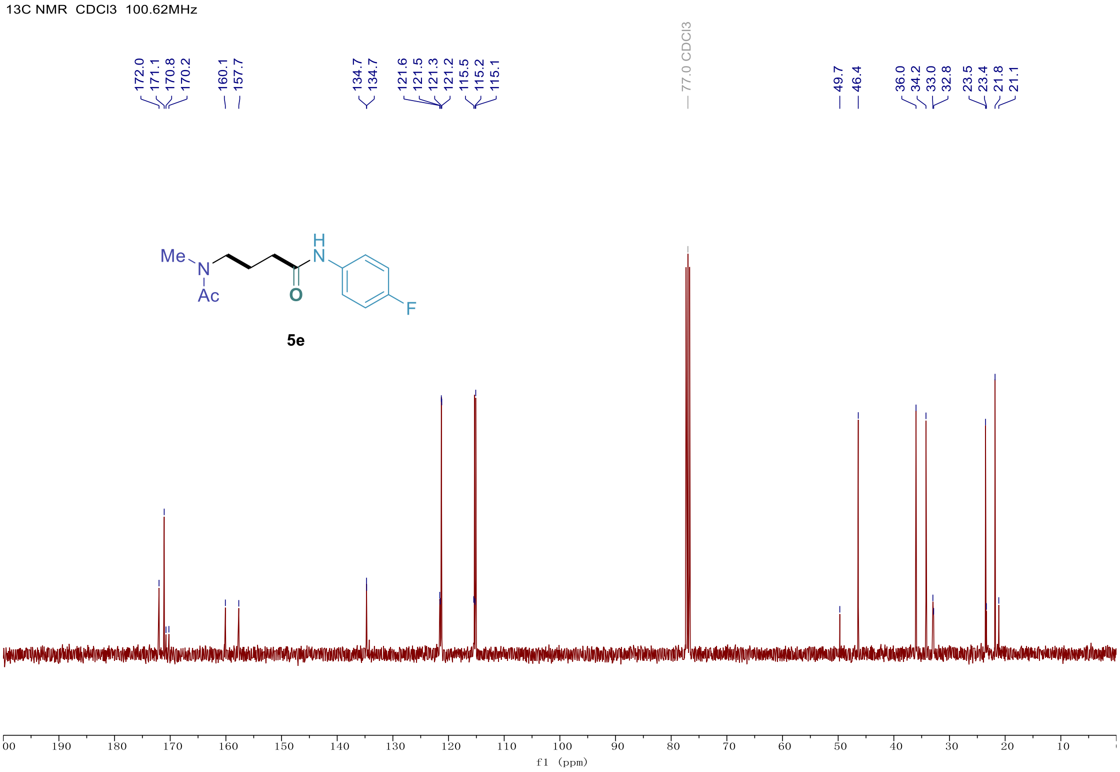

Supplementary Figure 68. ^{13}C NMR of compound **5e** (100 MHz, $CDCl_3$)

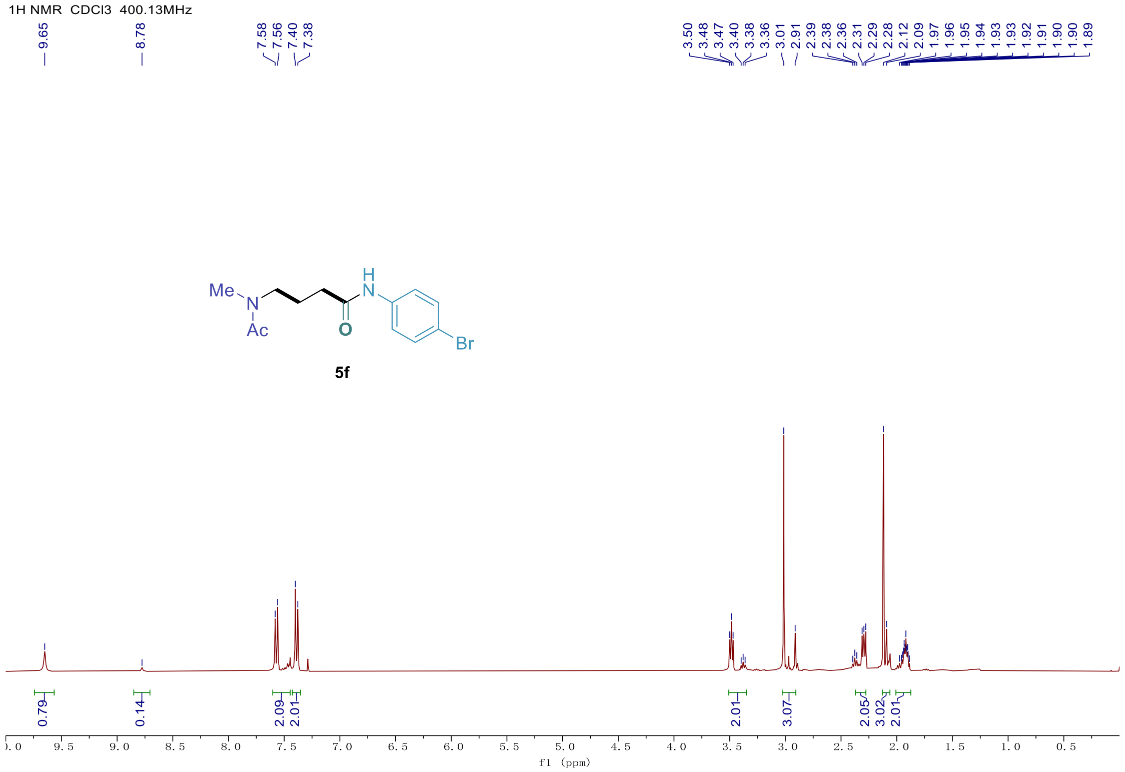

Supplementary Figure 69. ^{1}H NMR of compound **5f** (400 MHz, $CDCl_3$)

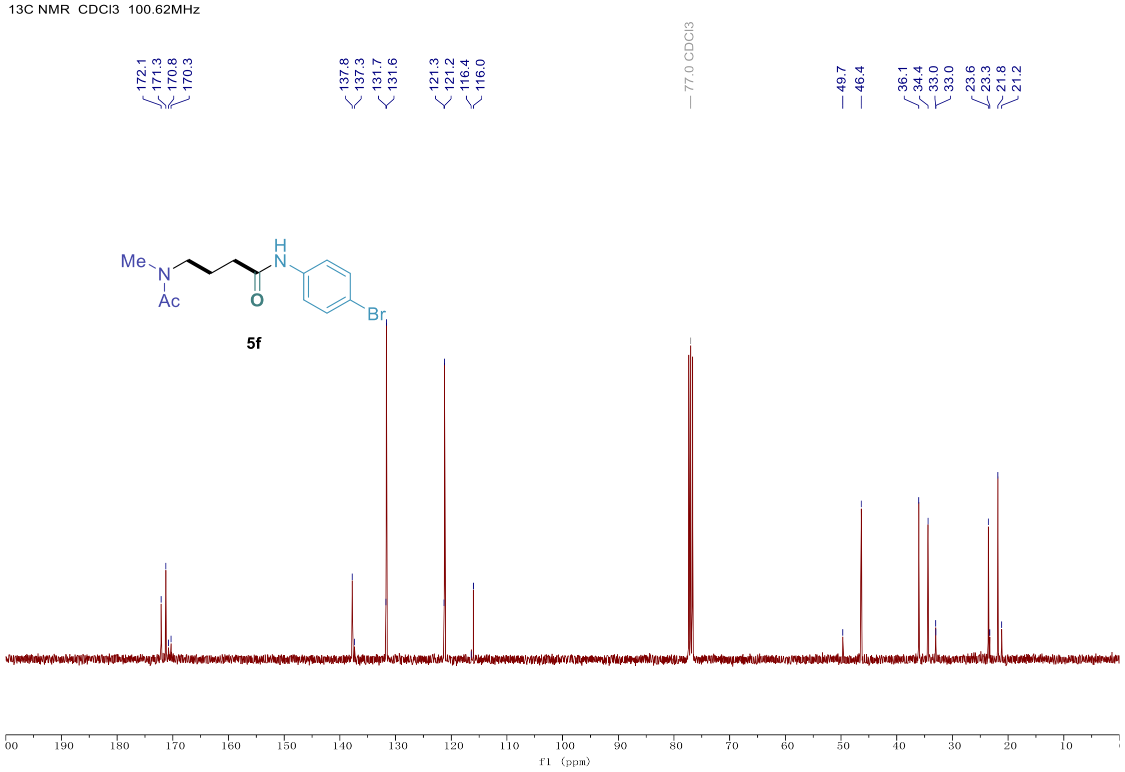

Supplementary Figure 70. ^{13}C NMR of compound **5f** (100 MHz, $CDCl_3$)

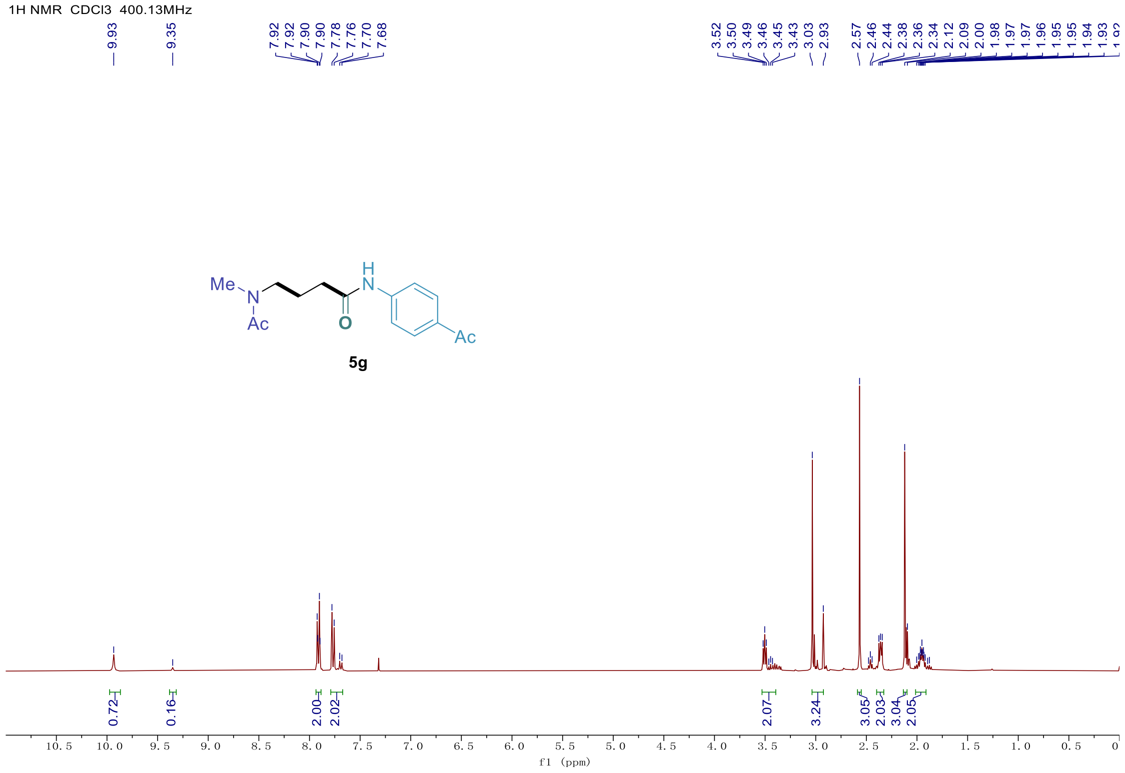

Supplementary Figure 71. ^{1}H NMR of compound **5g** (400 MHz, $CDCl_3$)

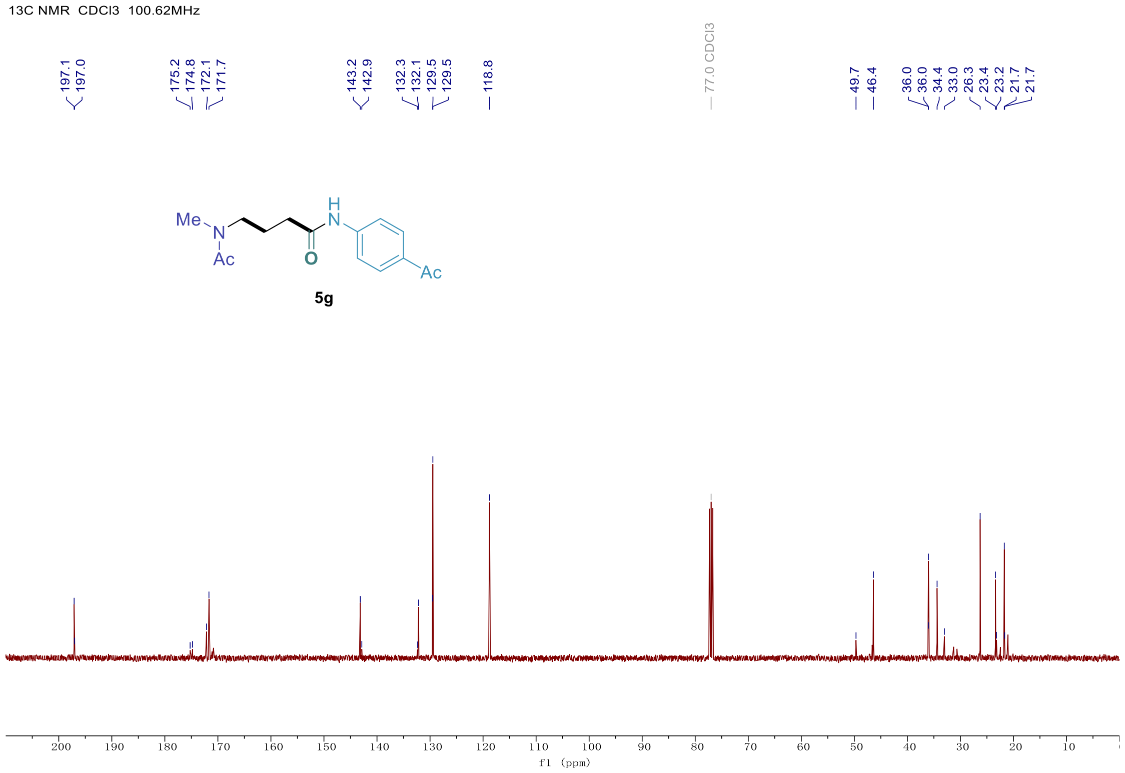

Supplementary Figure 72. ^{13}C NMR of compound **5g** (100 MHz, $CDCl_3$)

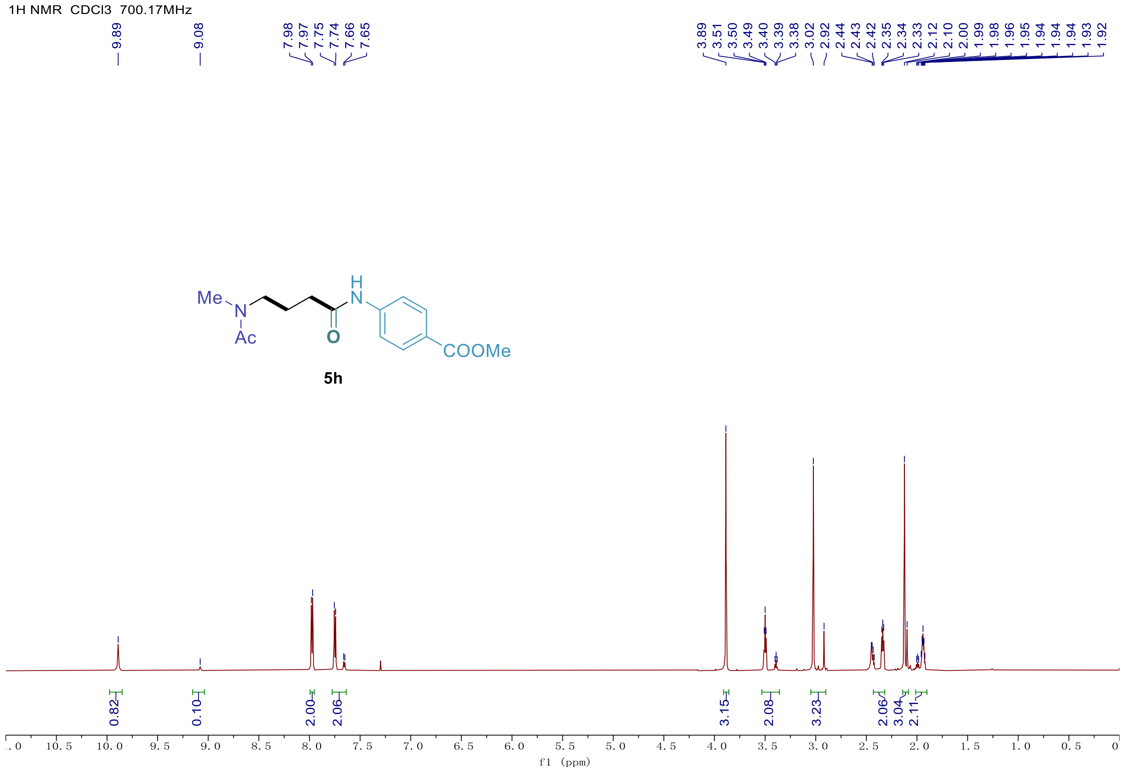

Supplementary Figure 73. ^{1}H NMR of compound **5h** (400 MHz, $CDCl_3$)

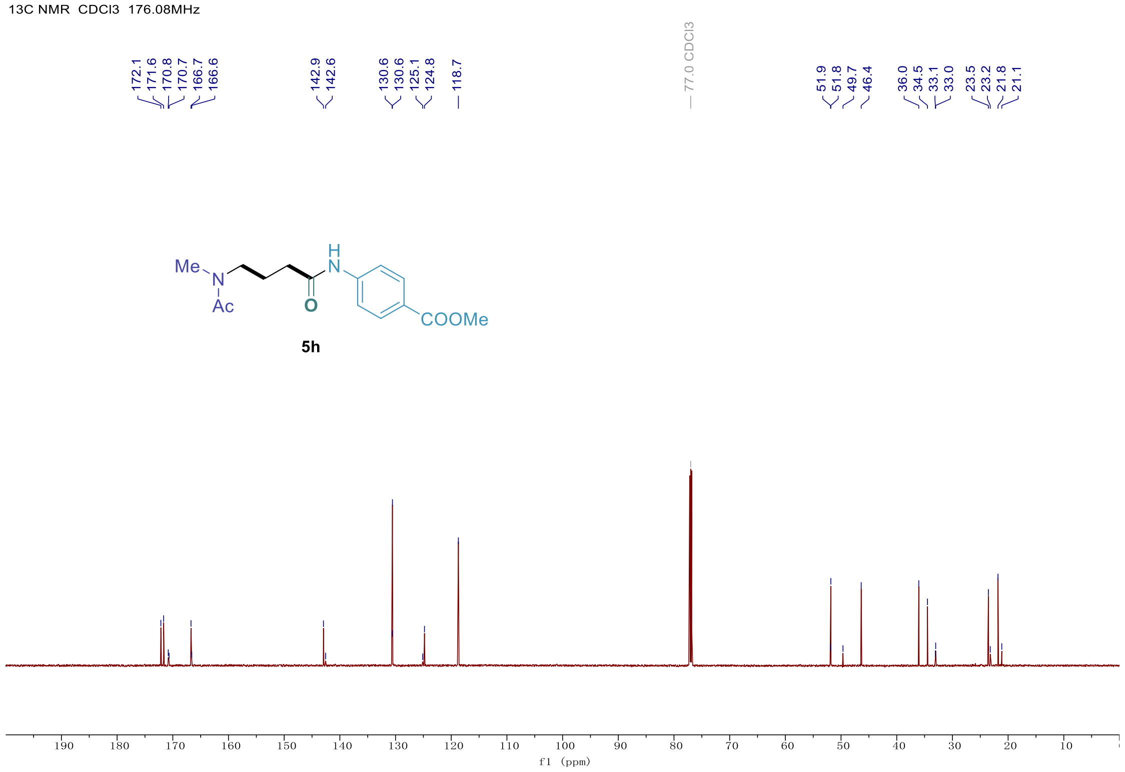

Supplementary Figure 74. ^{13}C NMR of compound **5h** (100 MHz, $CDCl_3$)

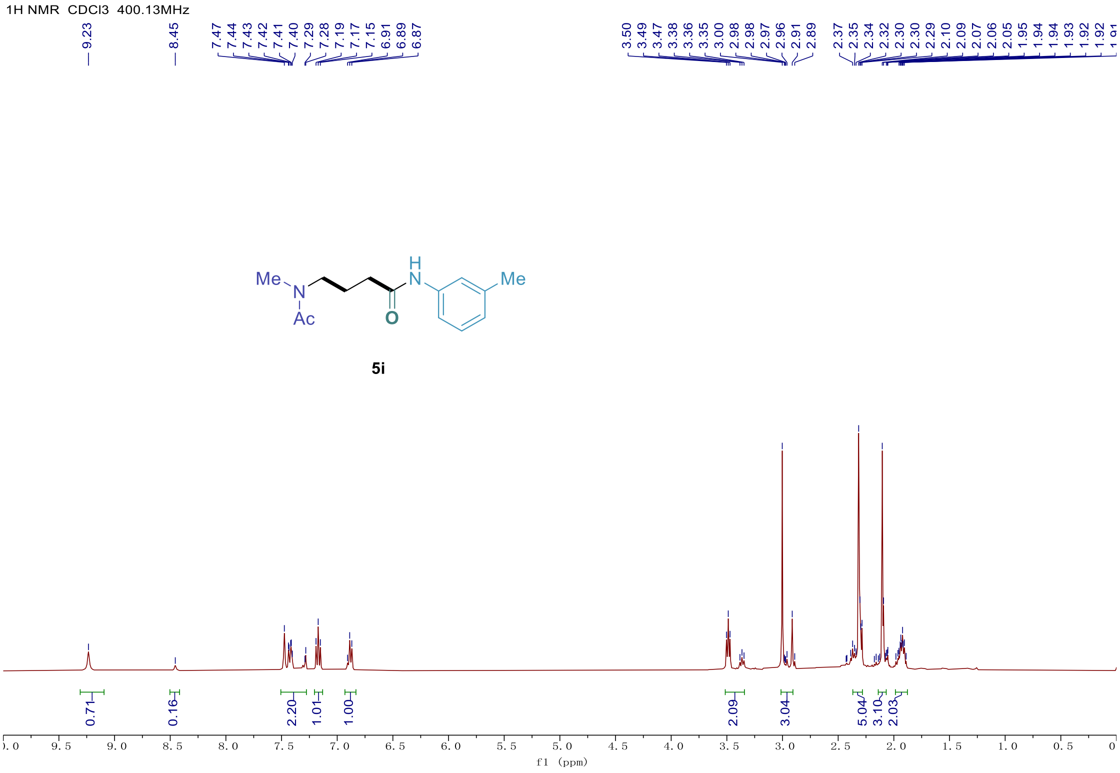

Supplementary Figure 75. ^{1}H NMR of compound **5i** (400 MHz, $CDCl_3$)

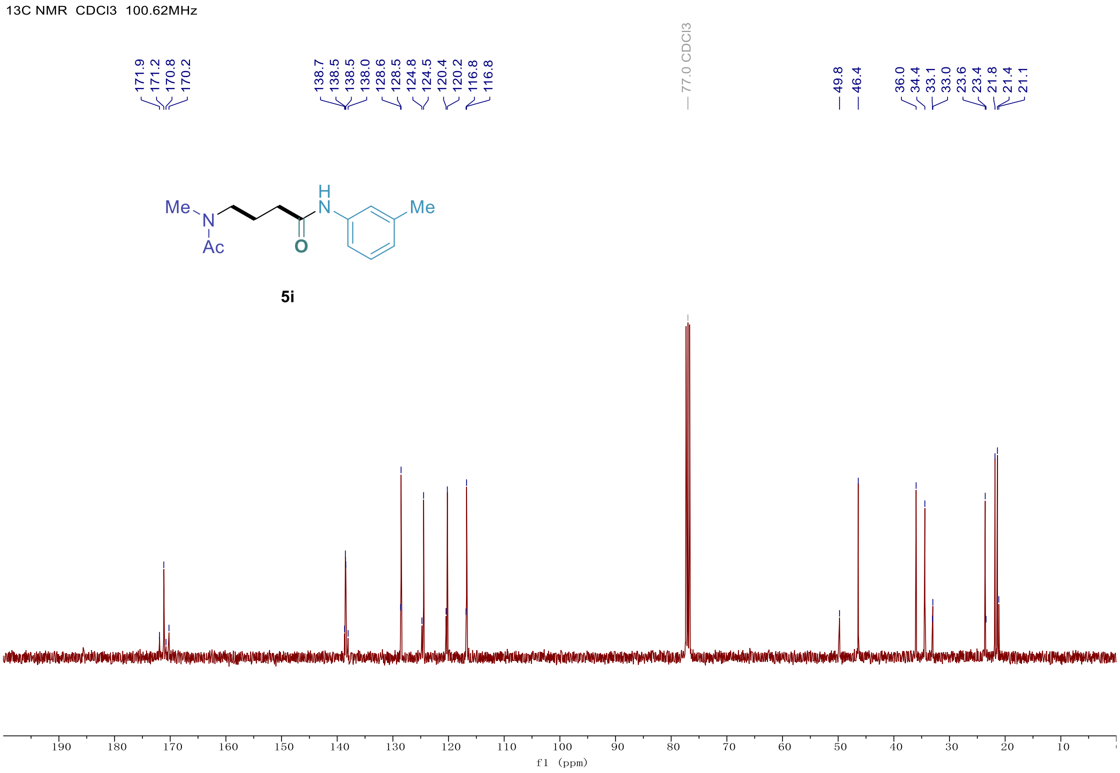

Supplementary Figure 76. ^{13}C NMR of compound **5i** (100 MHz, $CDCl_3$)

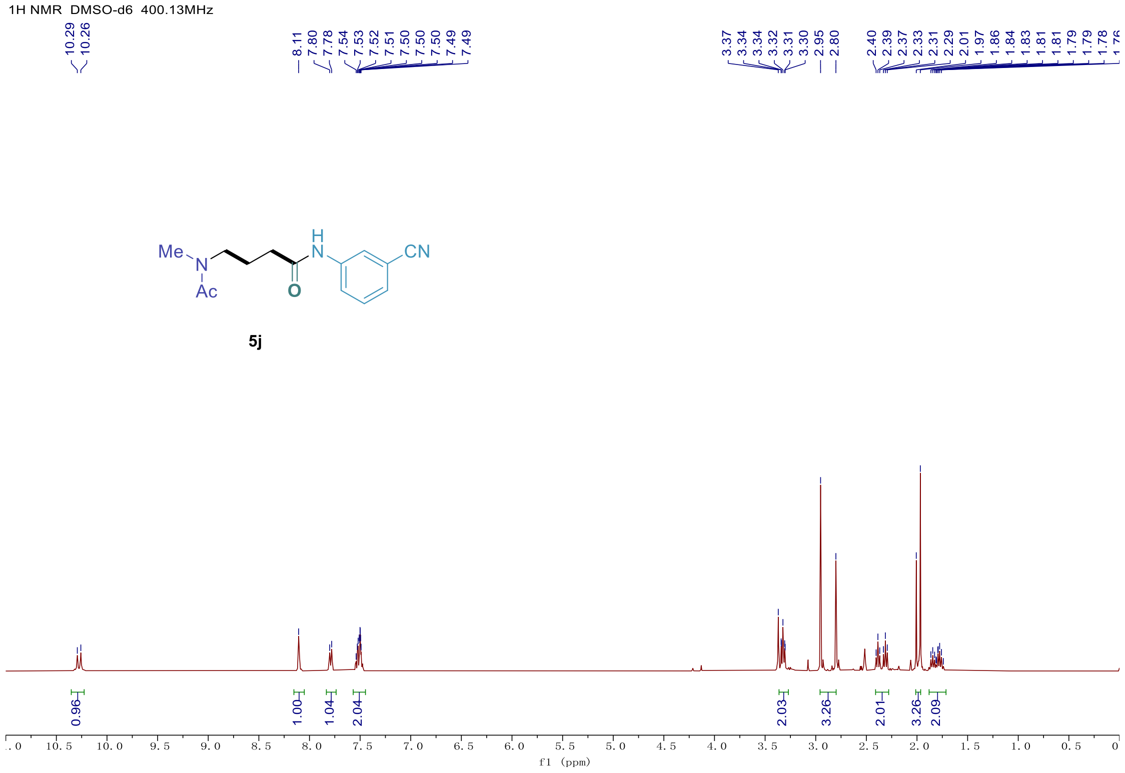

Supplementary Figure 77. ^{1}H NMR of compound **5j** (400 MHz, DMSO-*d6*)

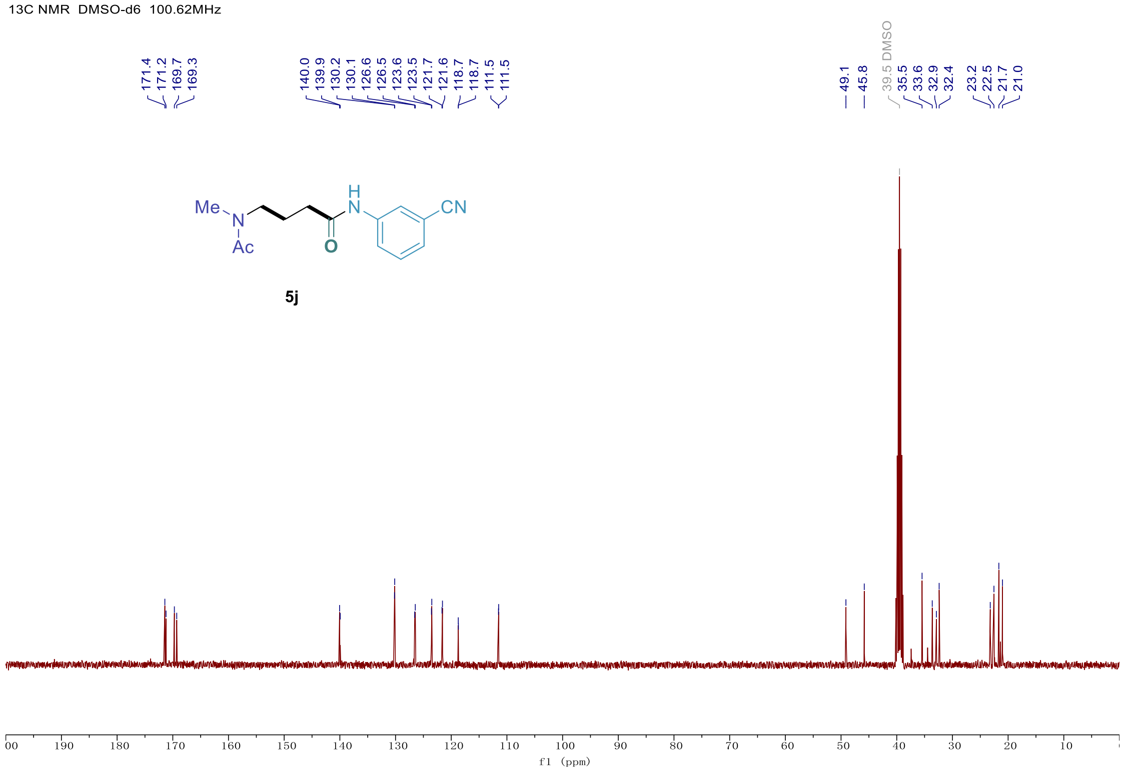

Supplementary Figure 78. ^{13}C NMR of compound **5j** (100 MHz, DMSO-*d6*)

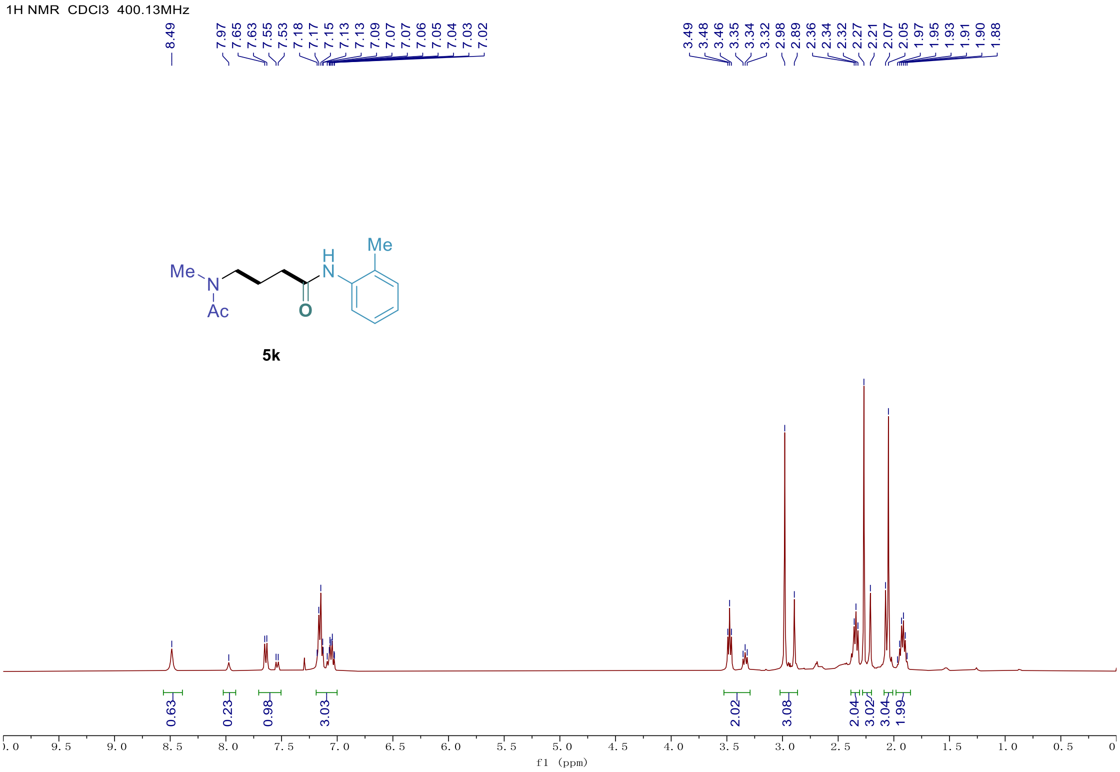

Supplementary Figure 79. ^{1}H NMR of compound **5k** (400 MHz, $CDCl_3$)

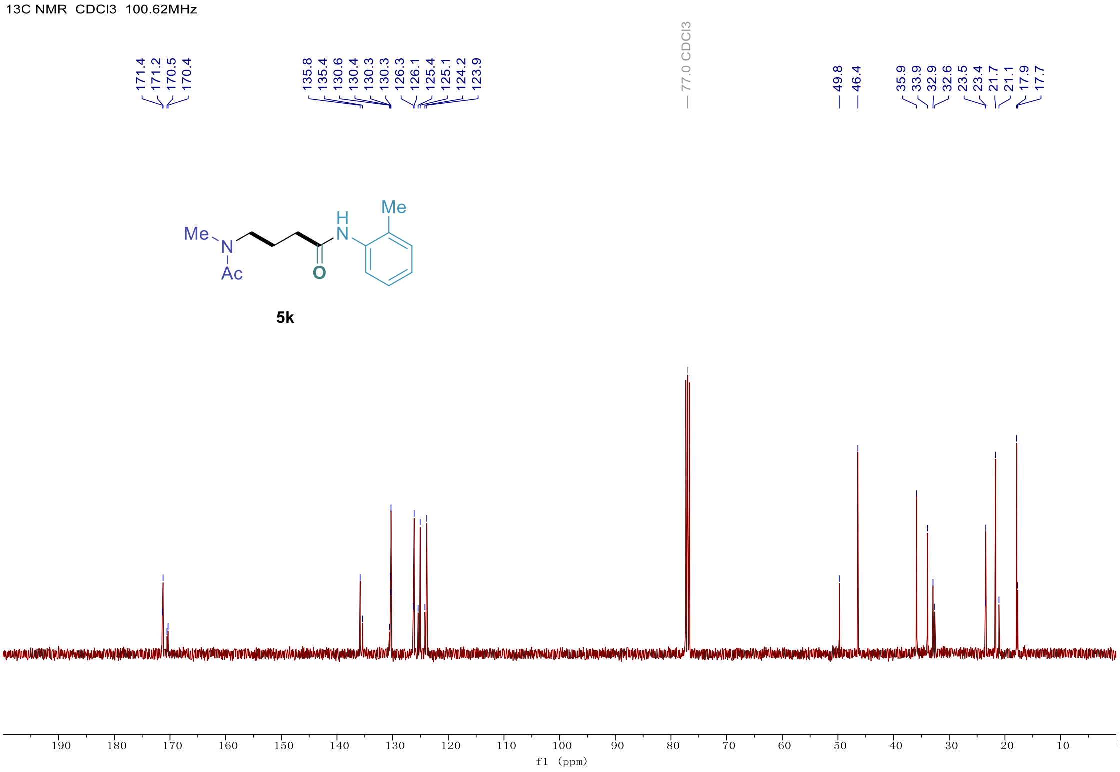

Supplementary Figure 80. ^{13}C NMR of compound **5k** (100 MHz, $CDCl_3$)

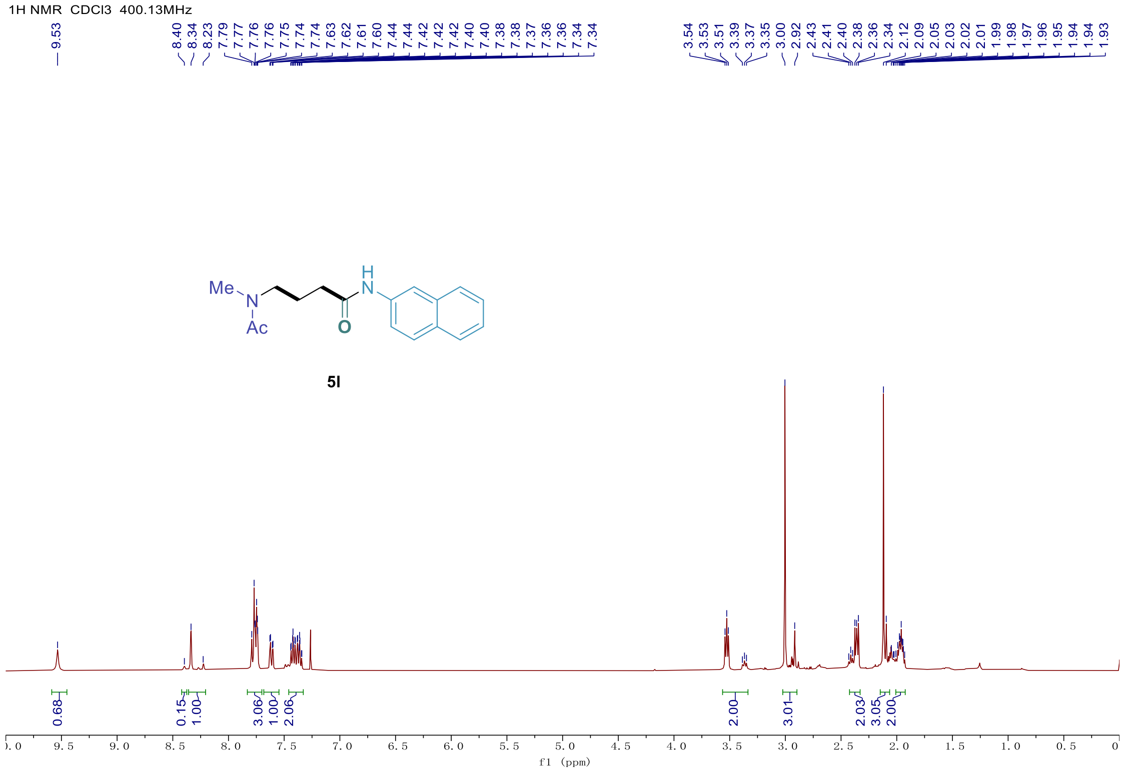

Supplementary Figure 81. ^{1}H NMR of compound **5l** (400 MHz, $CDCl_3$)

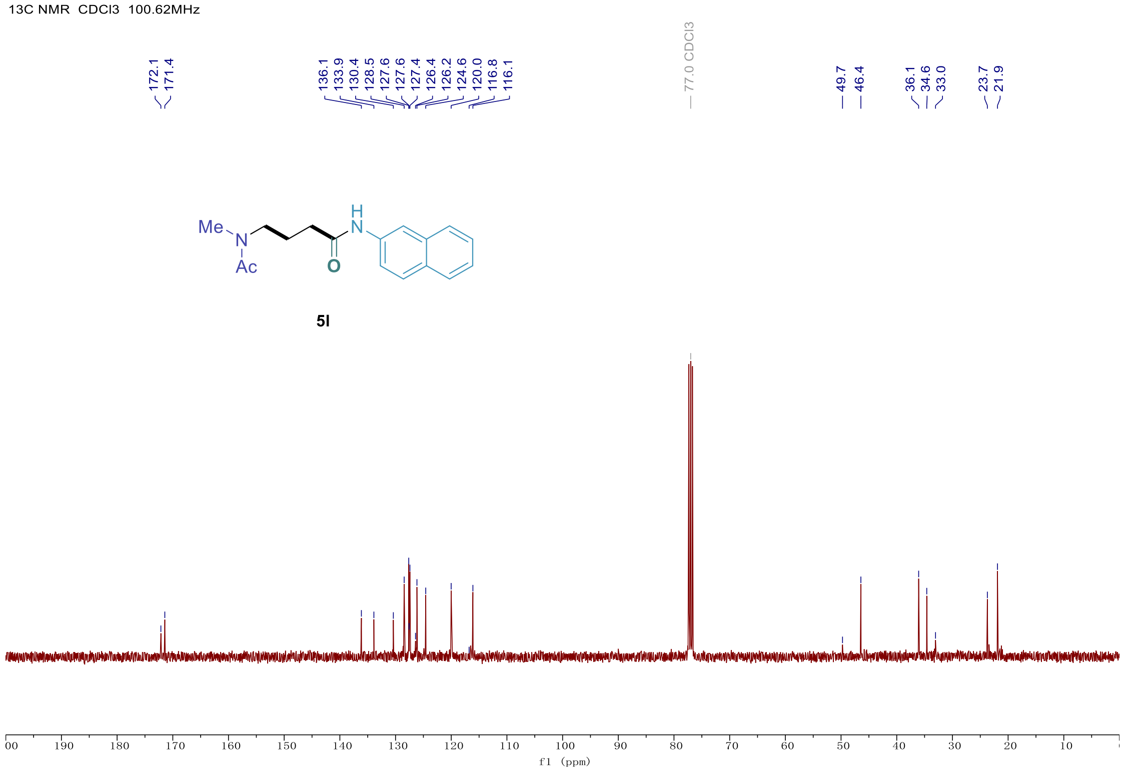

Supplementary Figure 82. ^{13}C NMR of compound **5l** (100 MHz, $CDCl_3$)

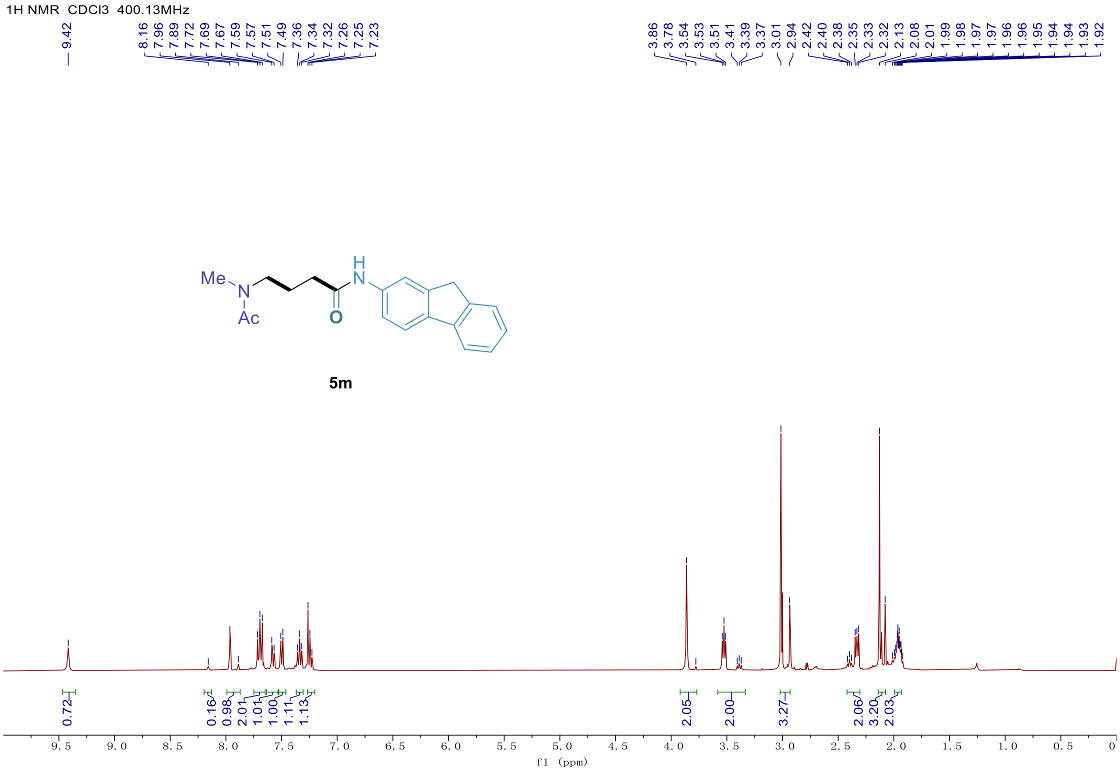

Supplementary Figure 83. ^{1}H NMR of compound **5m** (400 MHz, $CDCl_3$)

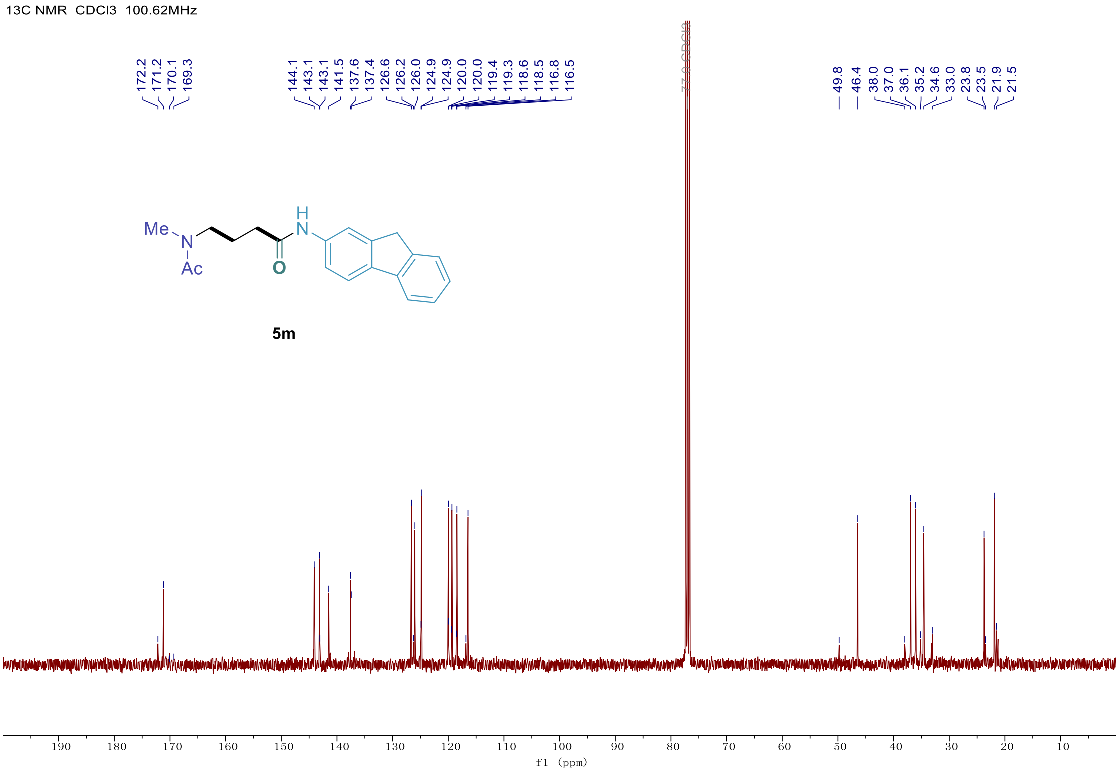

Supplementary Figure 84. ^{13}C NMR of compound **5m** (100 MHz, $CDCl_3$)

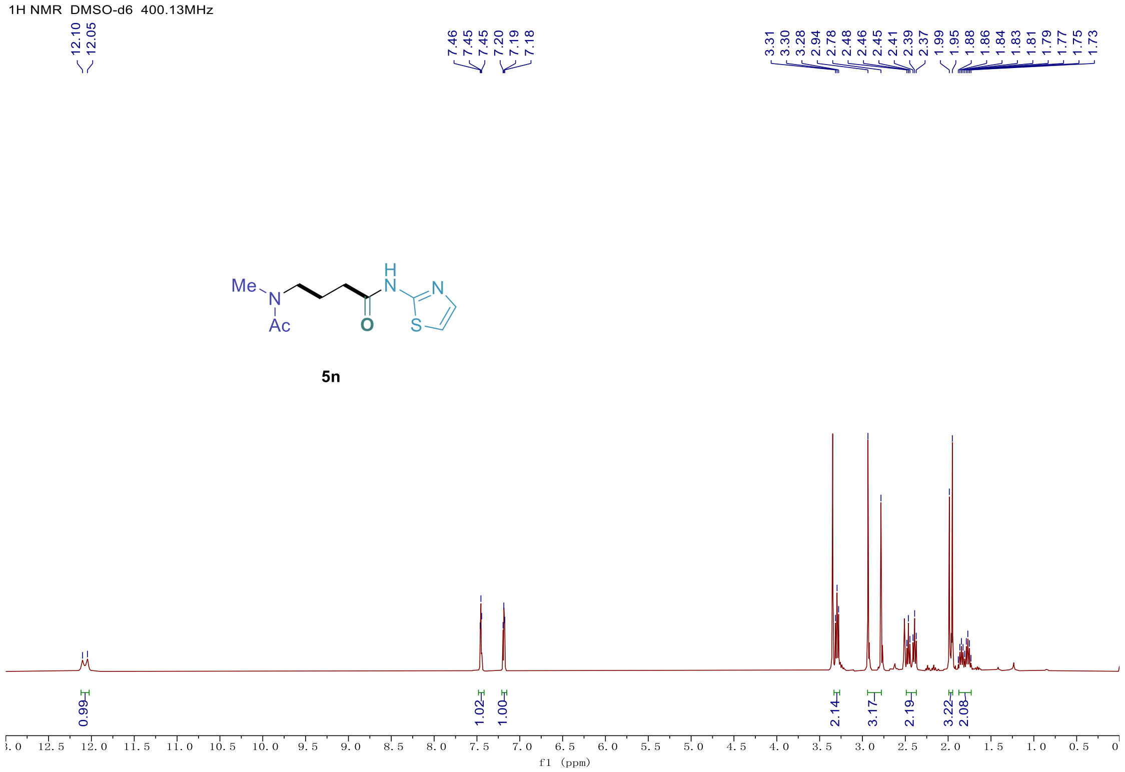

Supplementary Figure 85. ^{1}H NMR of compound **5n** (400 MHz, DMSO-*d6*)

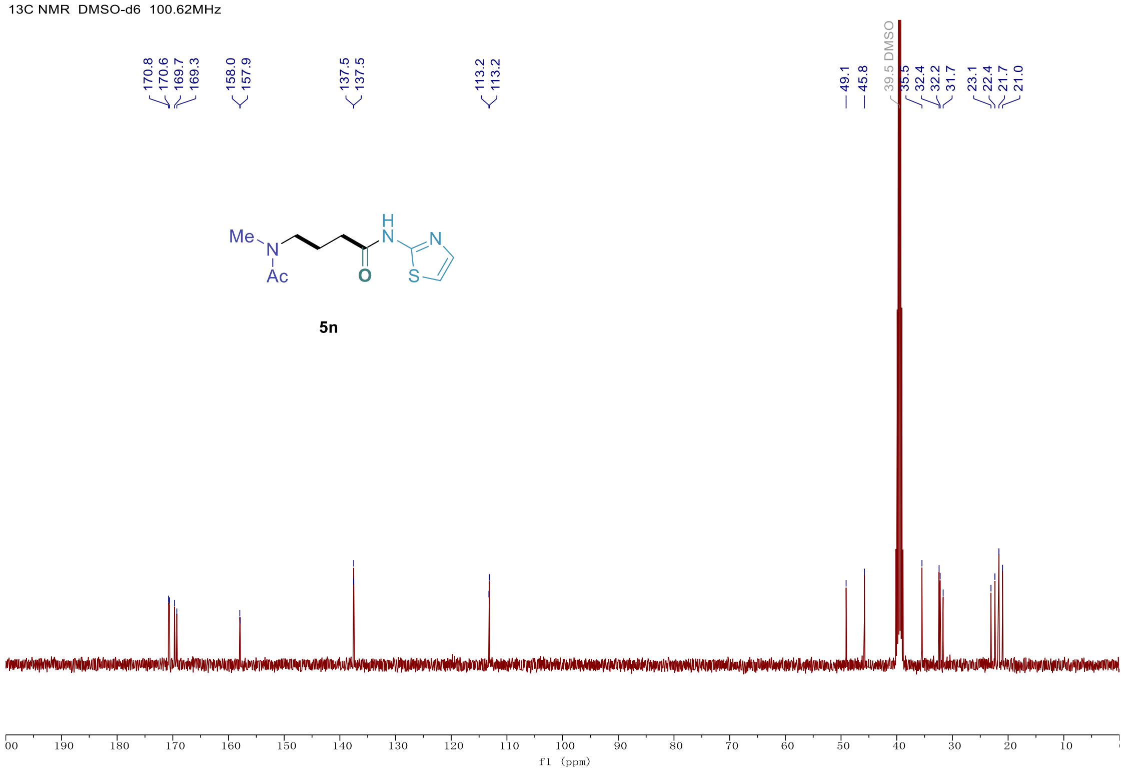

Supplementary Figure 86. ^{13}C NMR of compound **5n** (100 MHz, DMSO-*d6*)

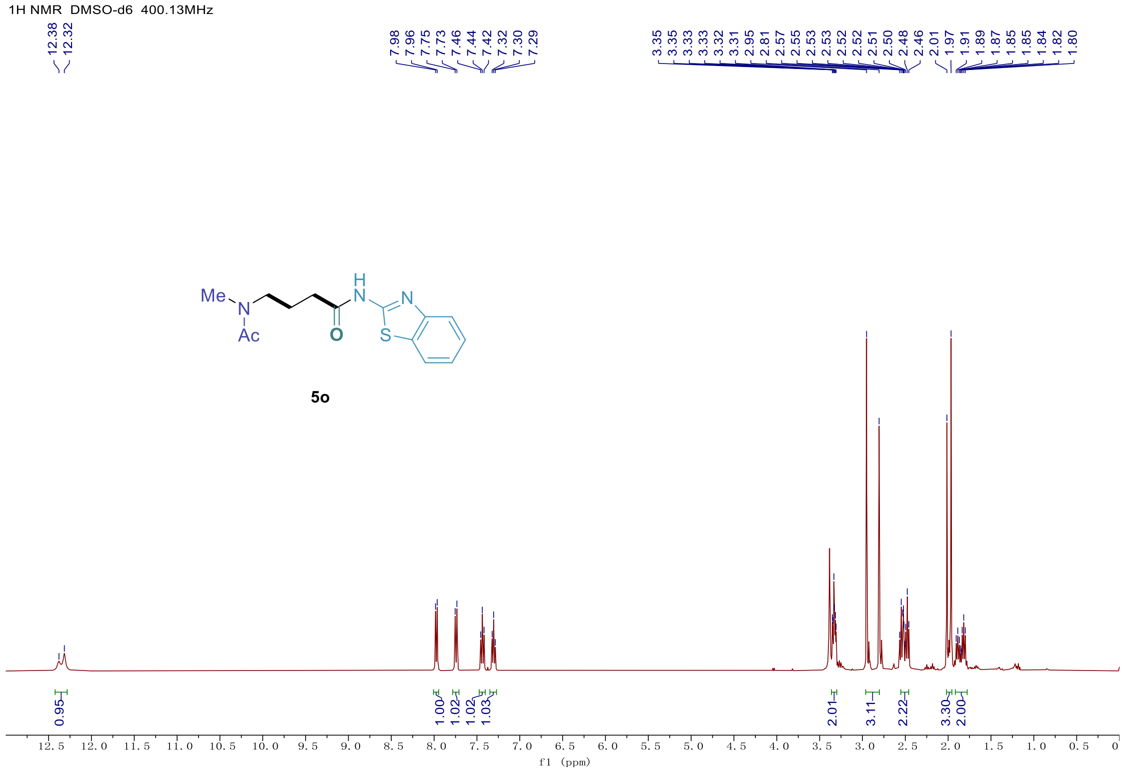

Supplementary Figure 87. ^{1}H NMR of compound **5o** (400 MHz, DMSO-*d6*)

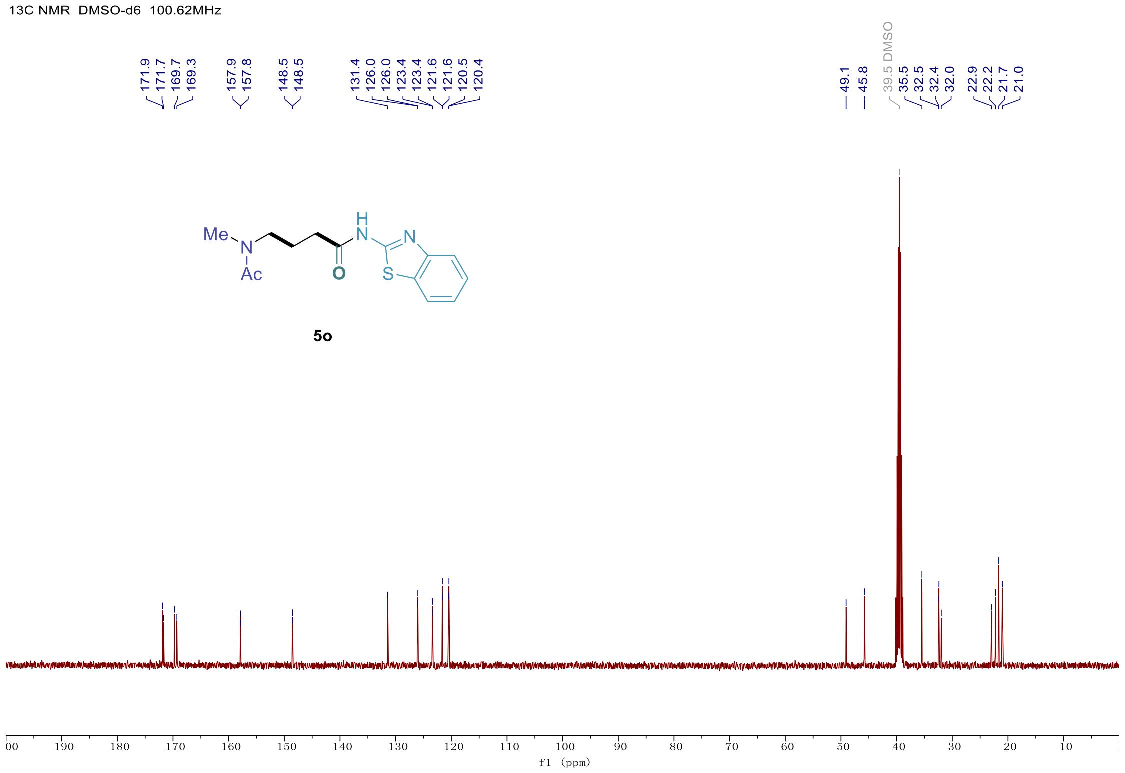

Supplementary Figure 88. ^{13}C NMR of compound **5o** (100 MHz, DMSO-*d6*)

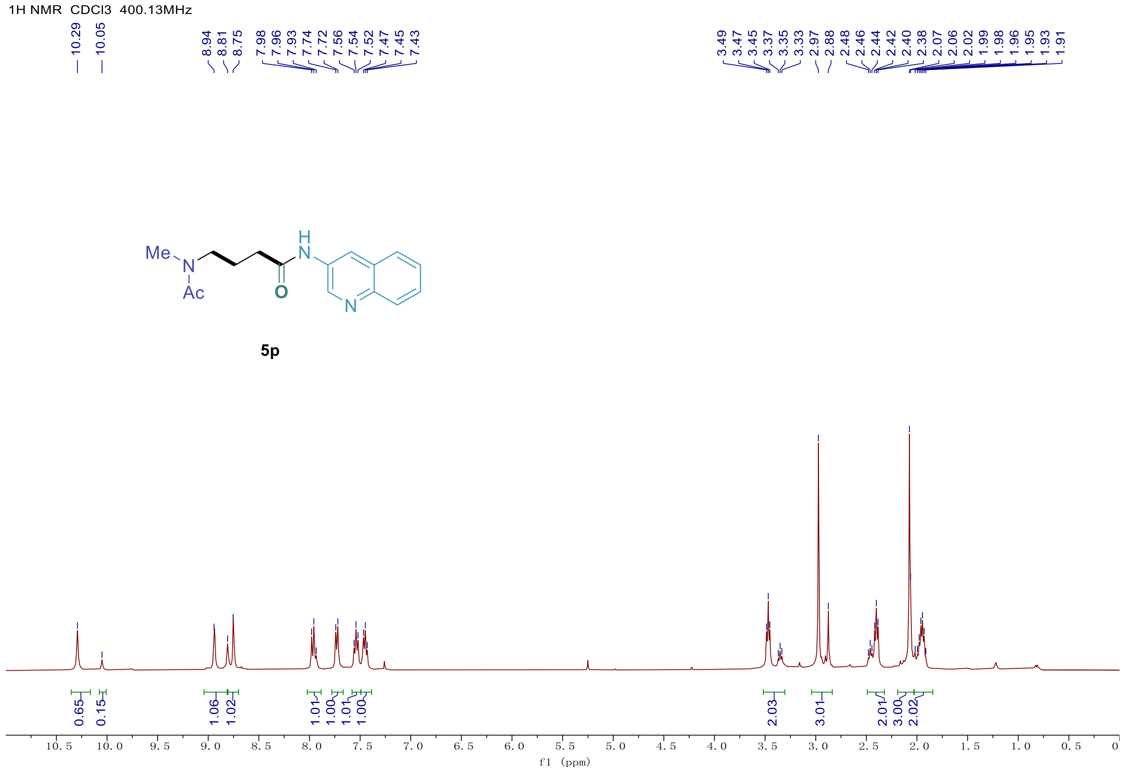

Supplementary Figure 89. ^{1}H NMR of compound **5p** (400 MHz, $CDCl_3$)

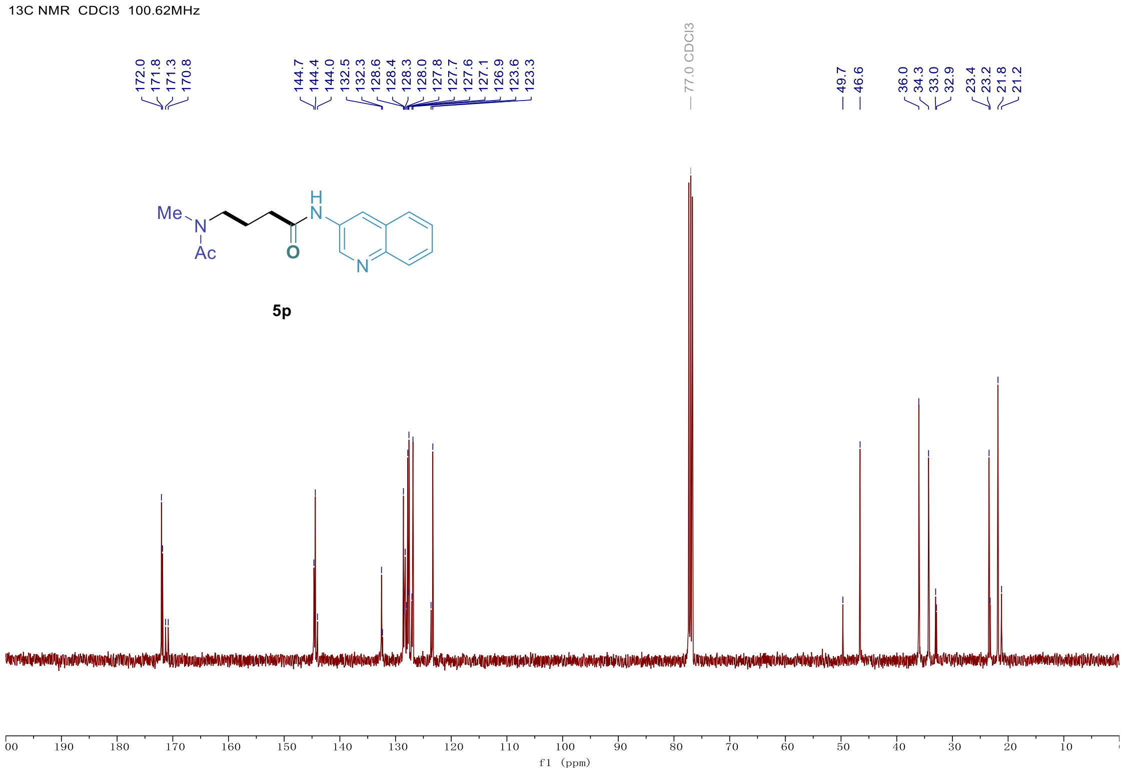

Supplementary Figure 90. ^{13}C NMR of compound **5p** (100 MHz, $CDCl_3$)

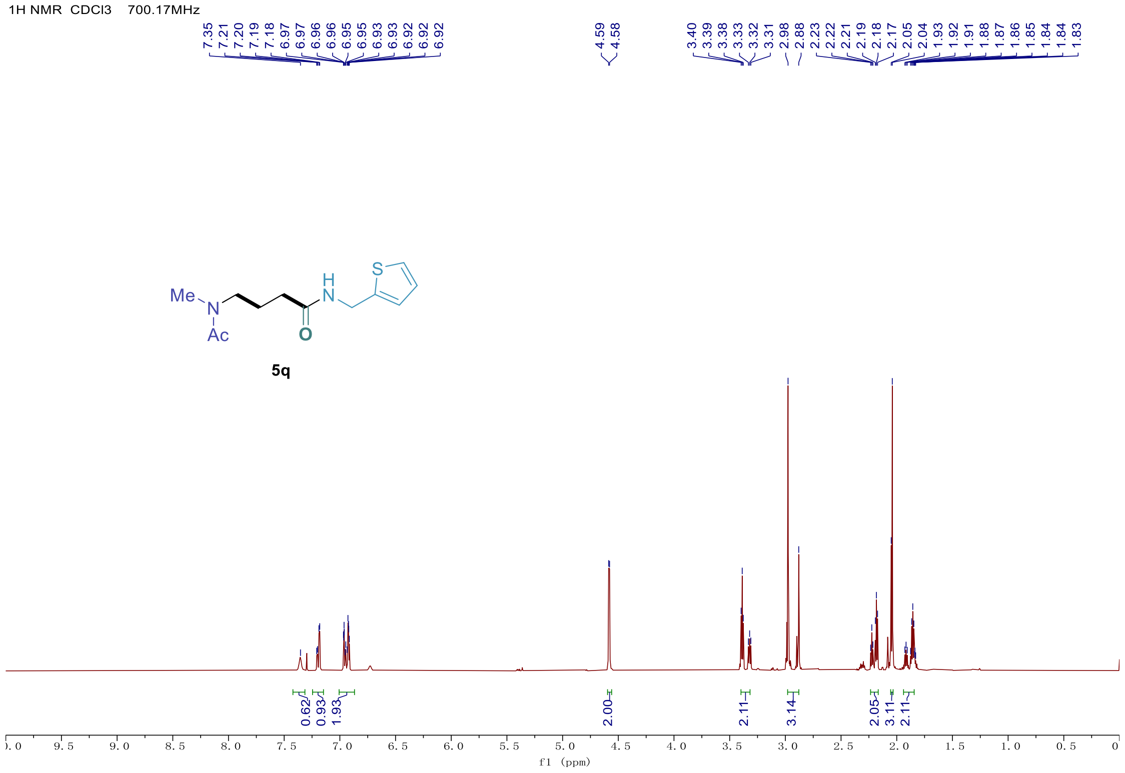

Supplementary Figure 91. ^{1}H NMR of compound **5q** (700 MHz, $CDCl_3$)

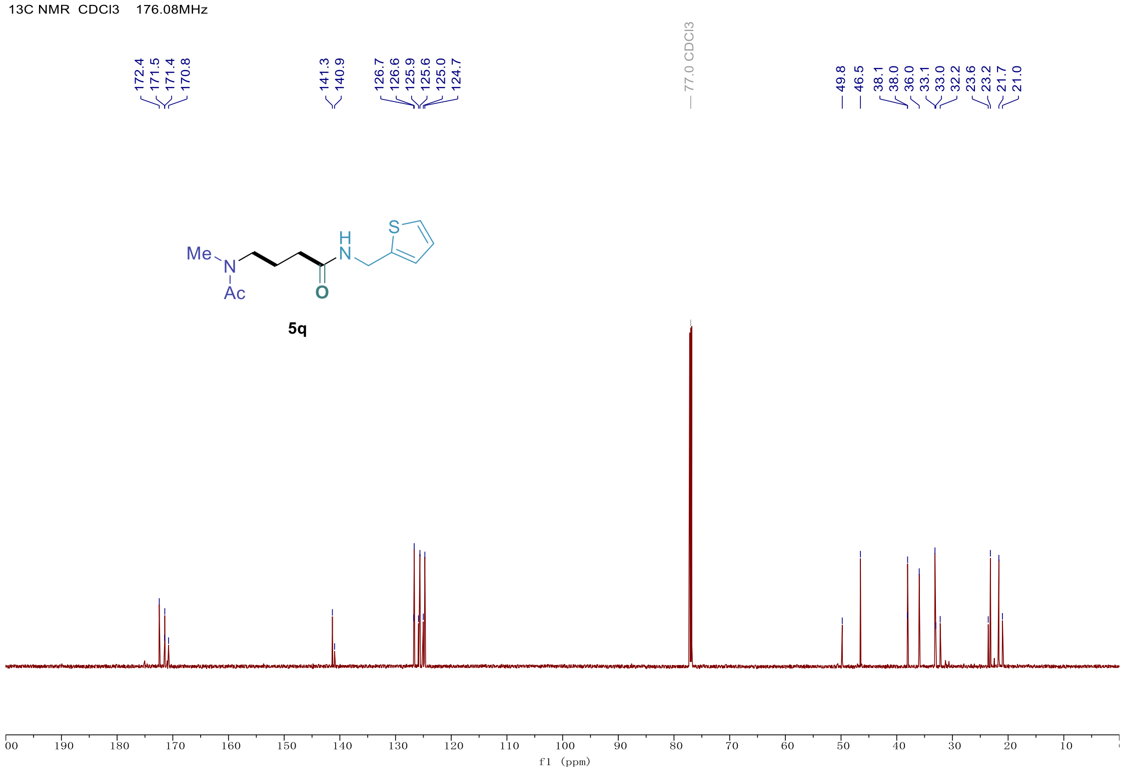

Supplementary Figure 92. ^{13}C NMR of compound **5q** (176 MHz, $CDCl_3$)

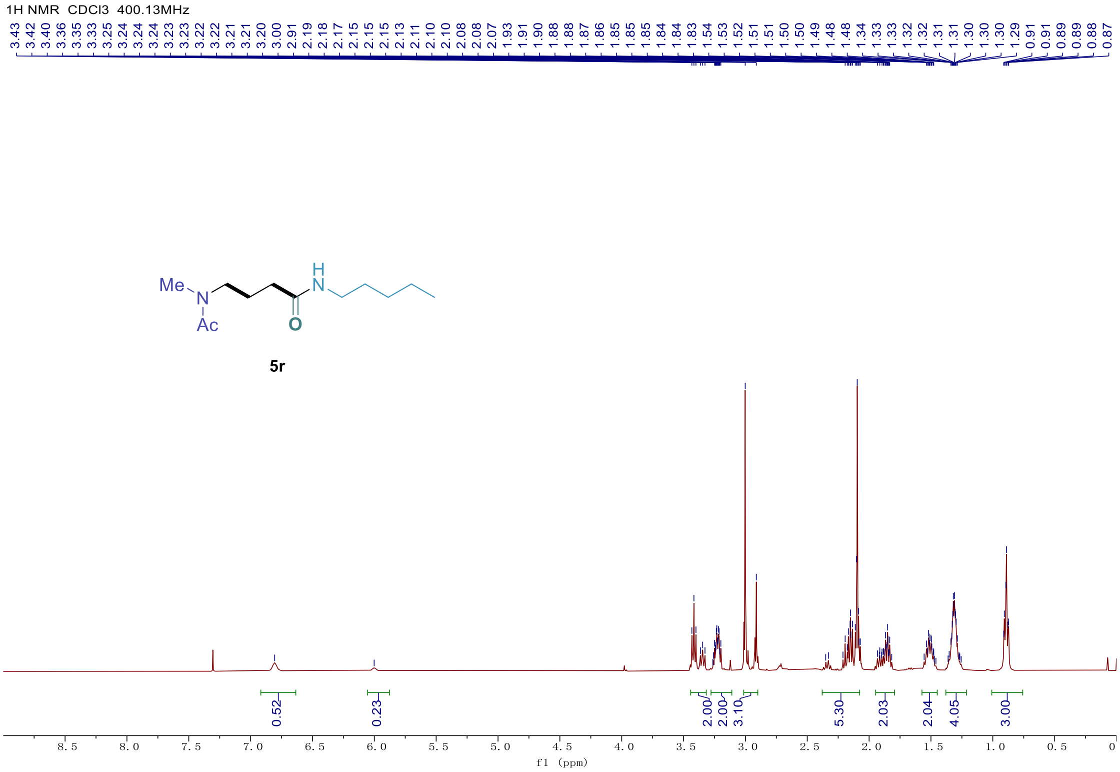

Supplementary Figure 93. ^{1}H NMR of compound **5r** (400 MHz, $CDCl_3$)

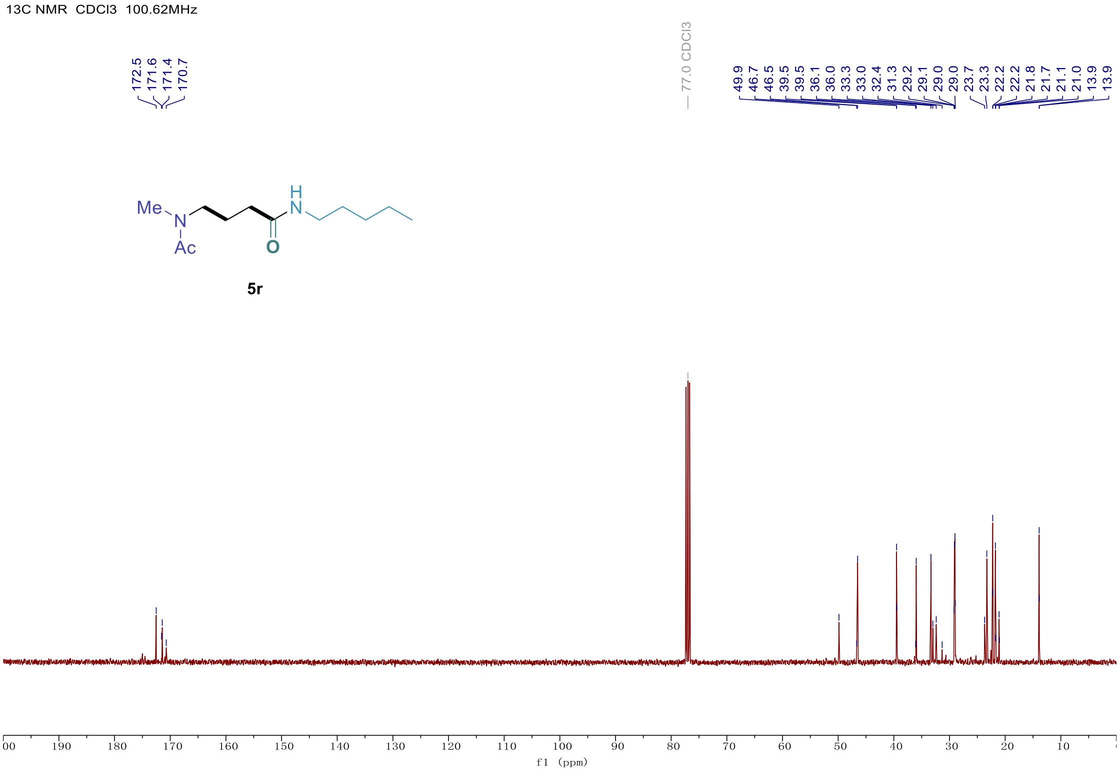

Supplementary Figure 94. ^{13}C NMR of compound **5r** (100 MHz, $CDCl_3$)

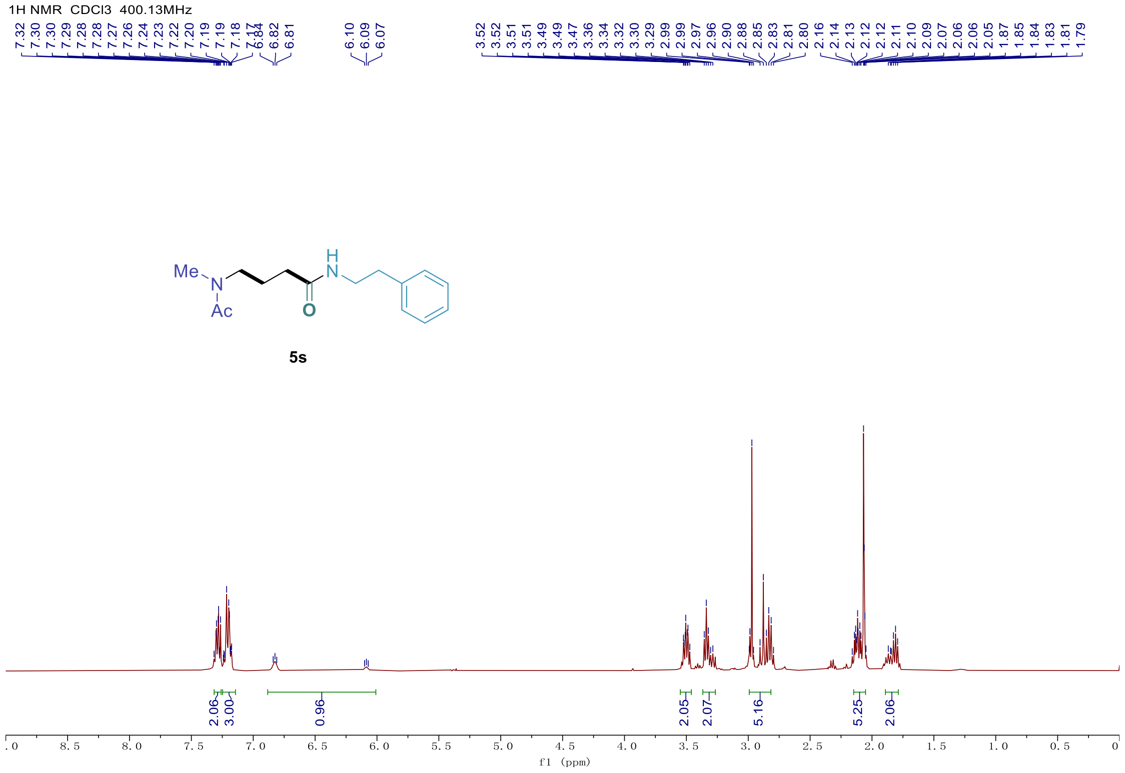

Supplementary Figure 95. ^{1}H NMR of compound **5s** (400 MHz, $CDCl_3$)

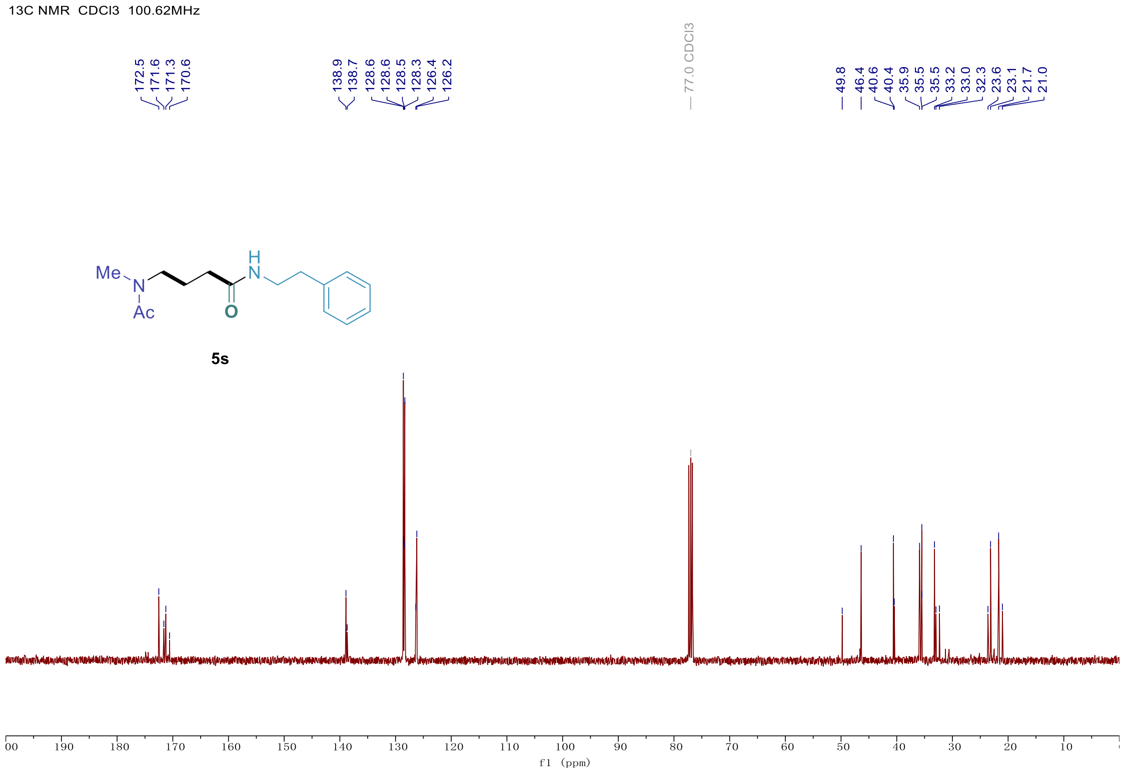

Supplementary Figure 96. ^{13}C NMR of compound **5s** (100 MHz, $CDCl_3$)

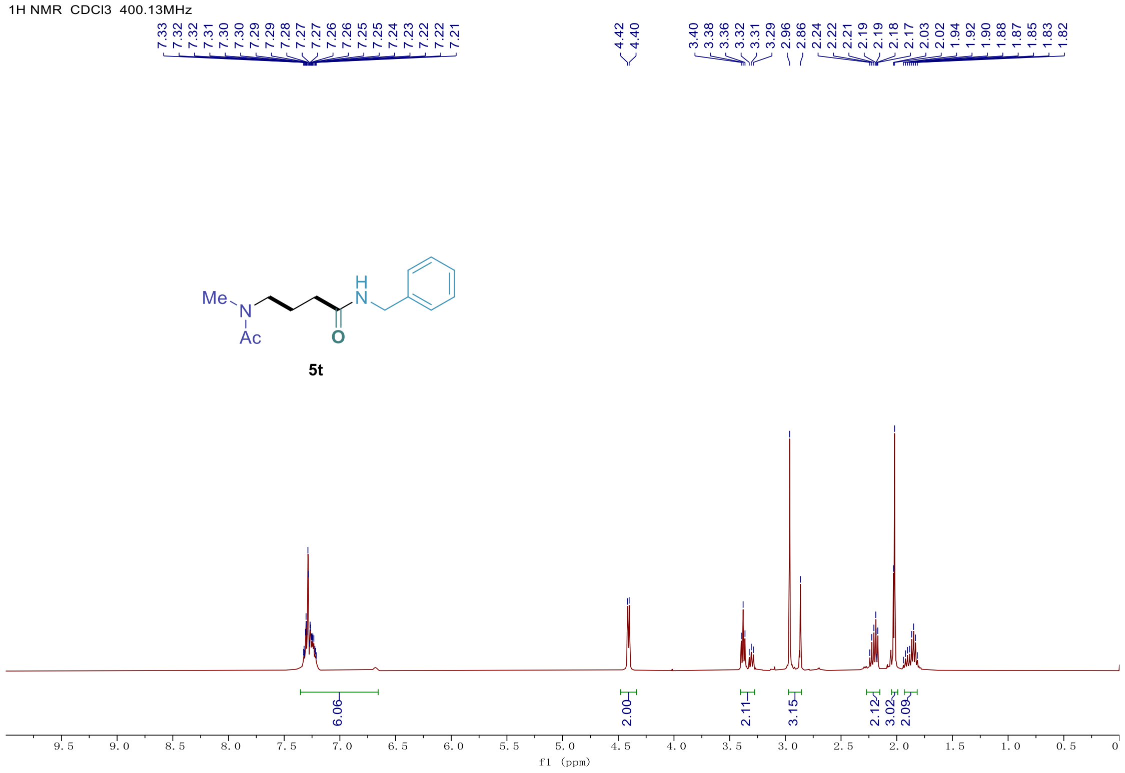

Supplementary Figure 97. ^{1}H NMR of compound **5t** (400 MHz, $CDCl_3$)

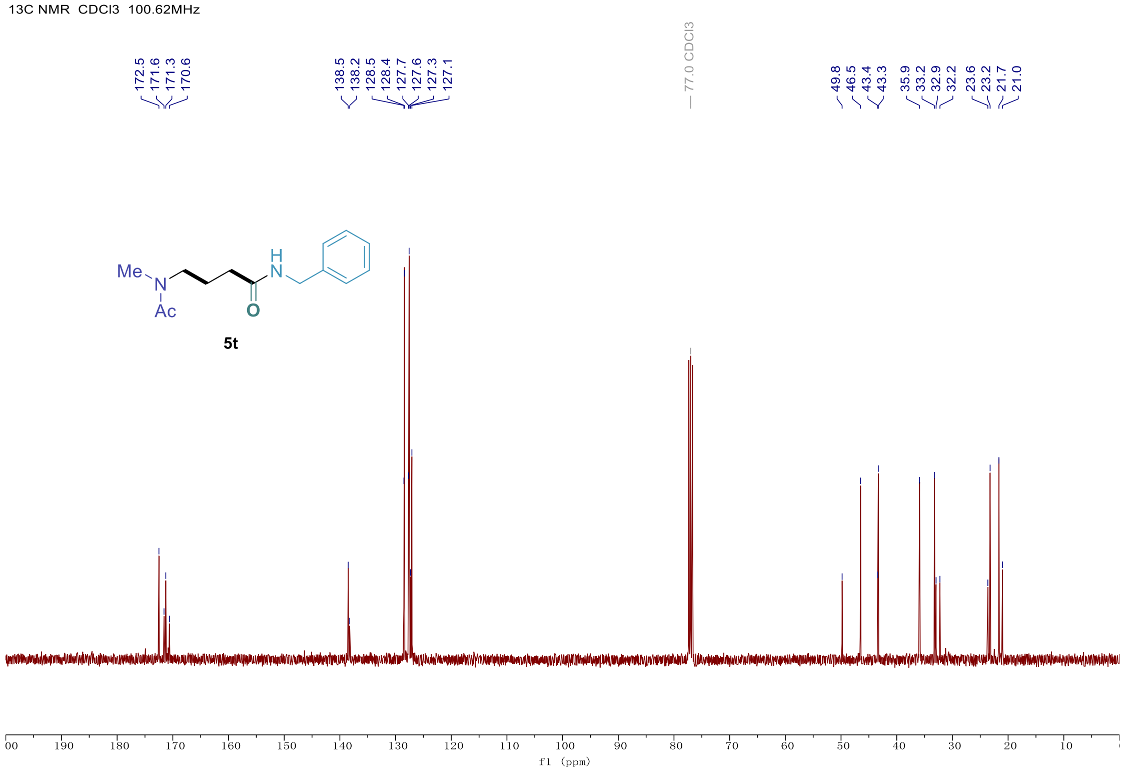

Supplementary Figure 98. ^{13}C NMR of compound **5t** (100 MHz, $CDCl_3$)

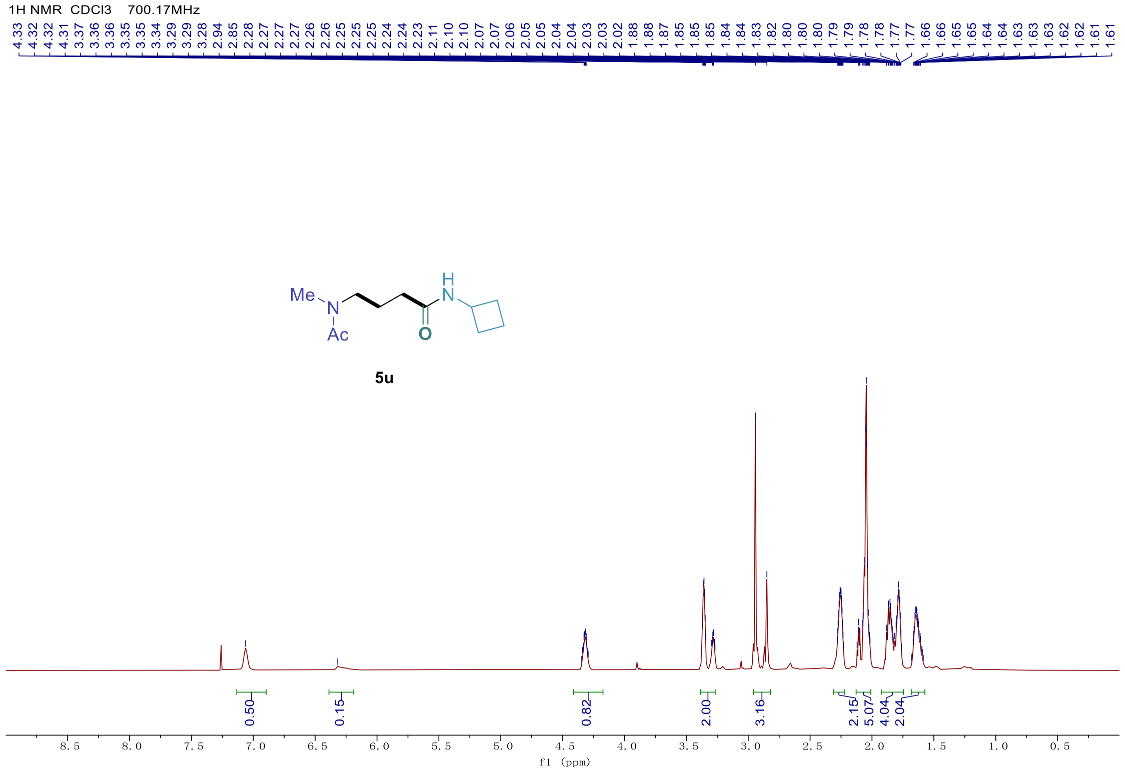

Supplementary Figure 99. ^{1}H NMR of compound **5u** (700 MHz, $CDCl_3$)

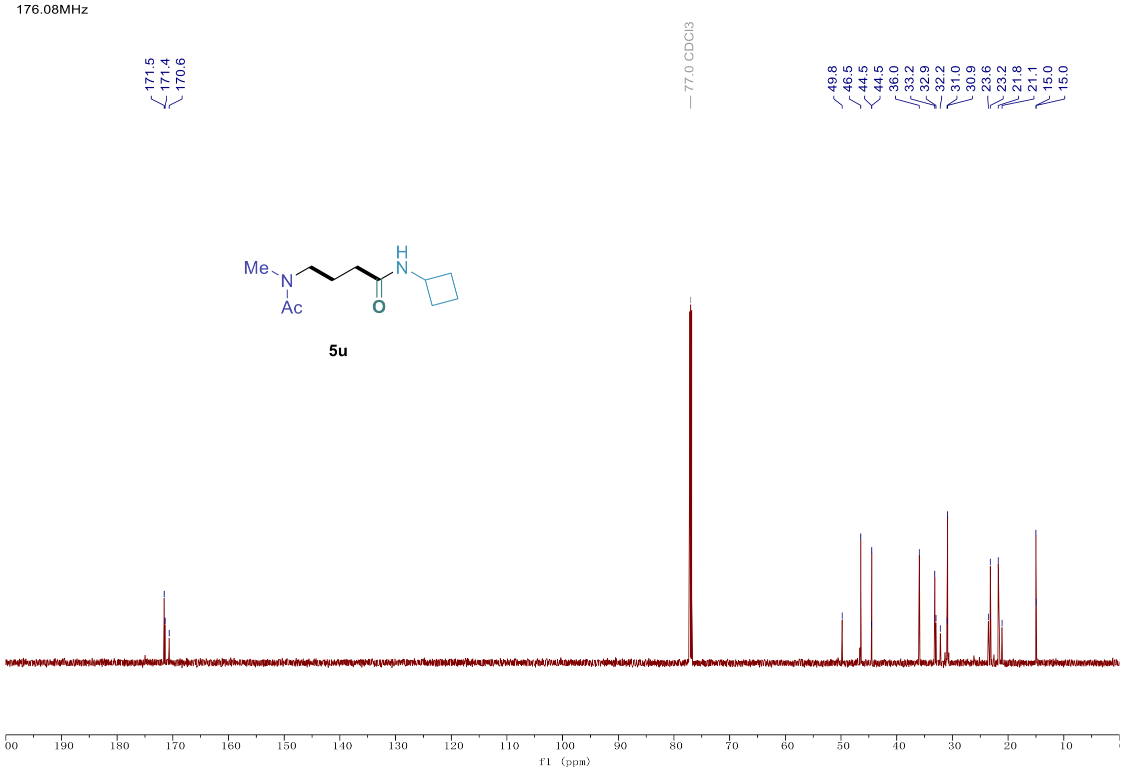

Supplementary Figure 100. ^{13}C NMR of compound **5u** (176 MHz, $CDCl_3$)

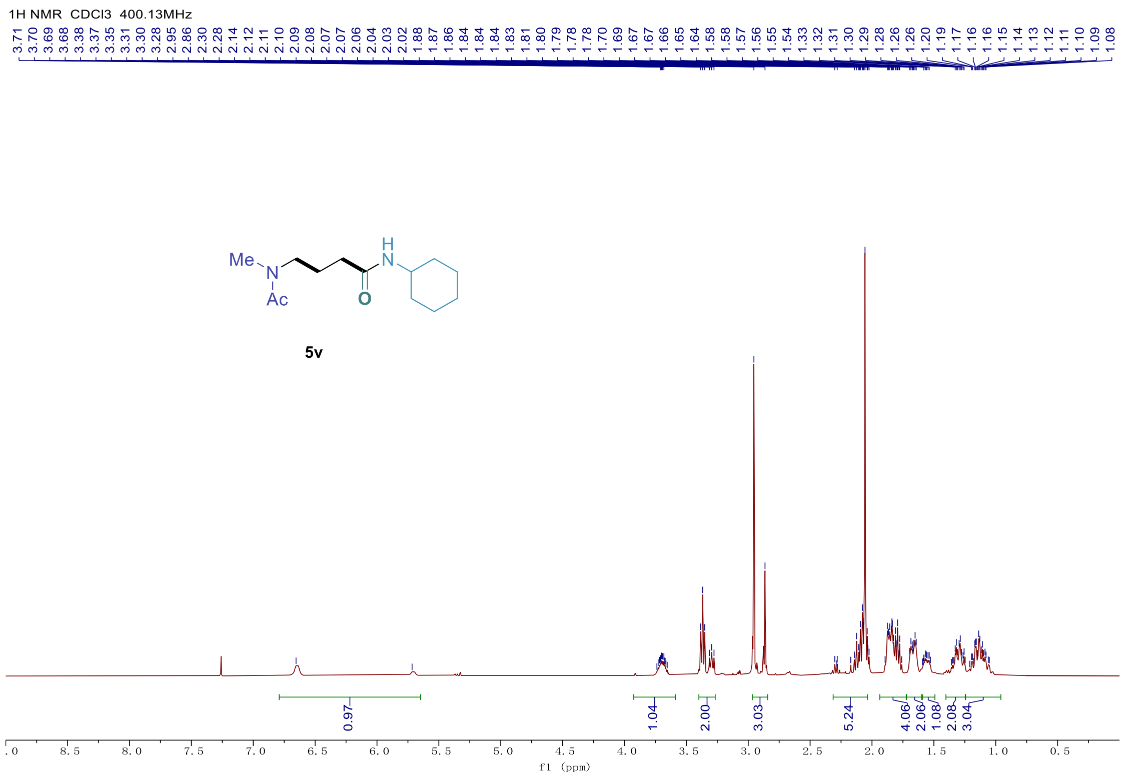

Supplementary Figure 101. ^{1}H NMR of compound **5v** (400 MHz, $CDCl_3$)

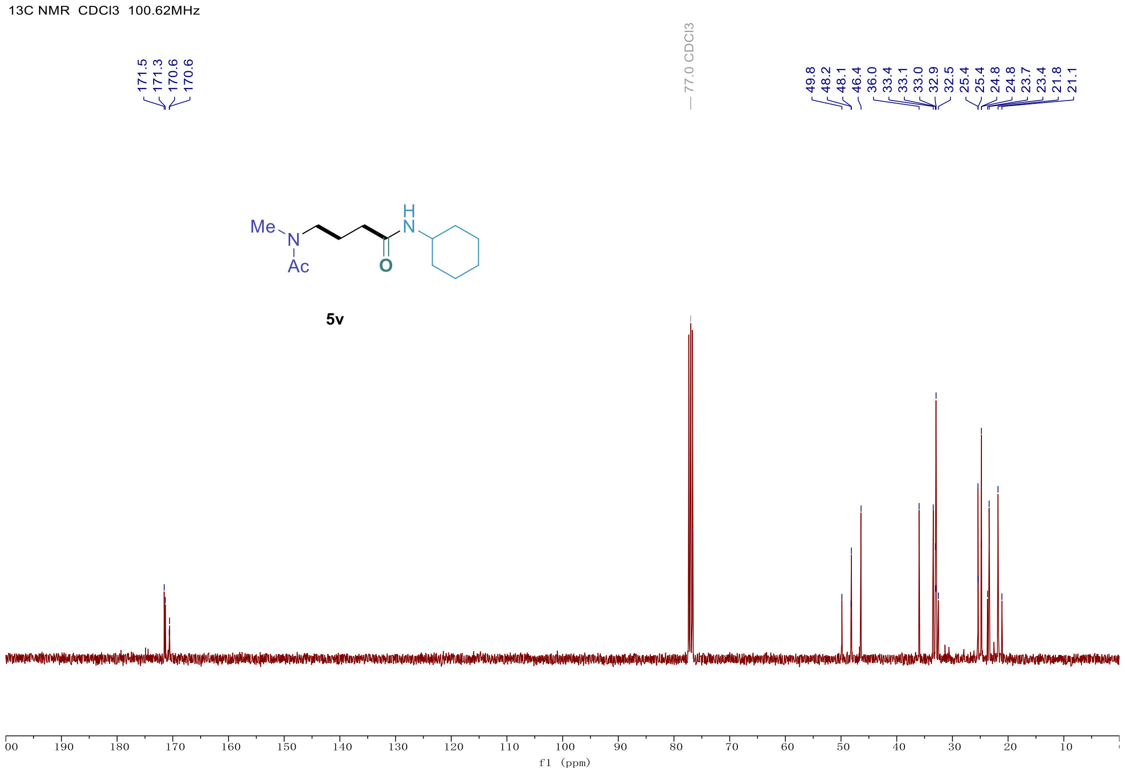

Supplementary Figure 102. ^{13}C NMR of compound **5v** (100 MHz, $CDCl_3$)

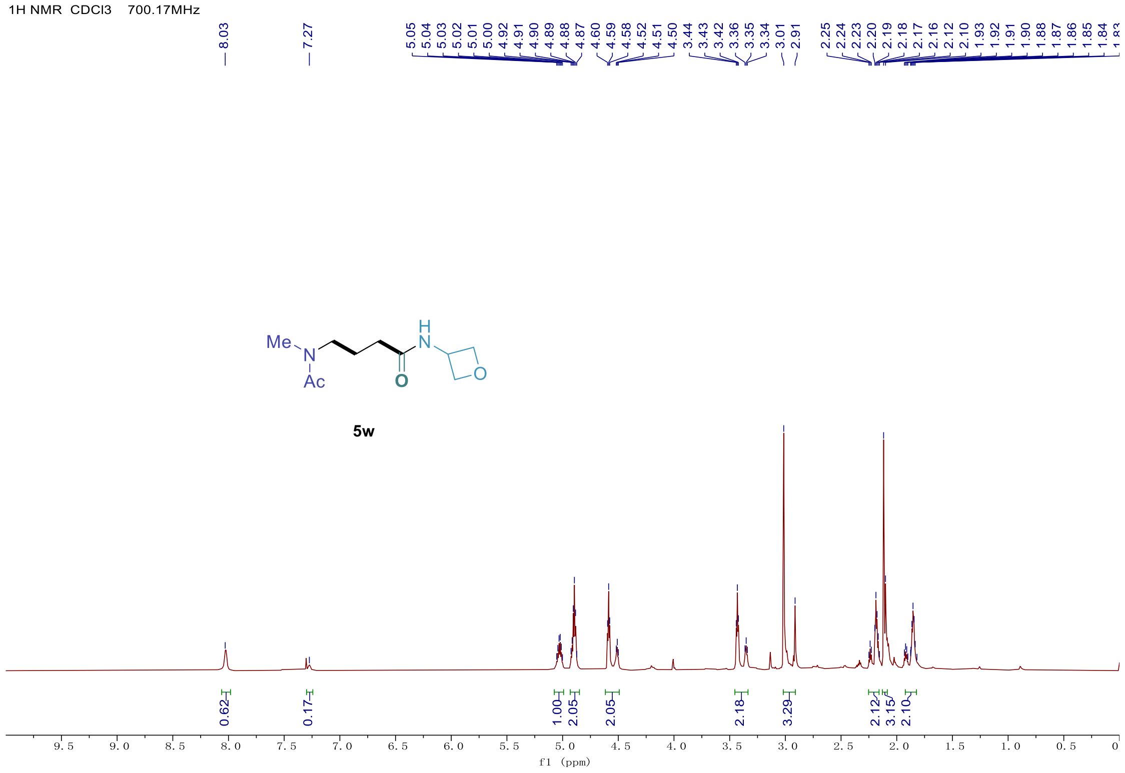

Supplementary Figure 103. ^{1}H NMR of compound **5w** (700 MHz, $CDCl_3$)

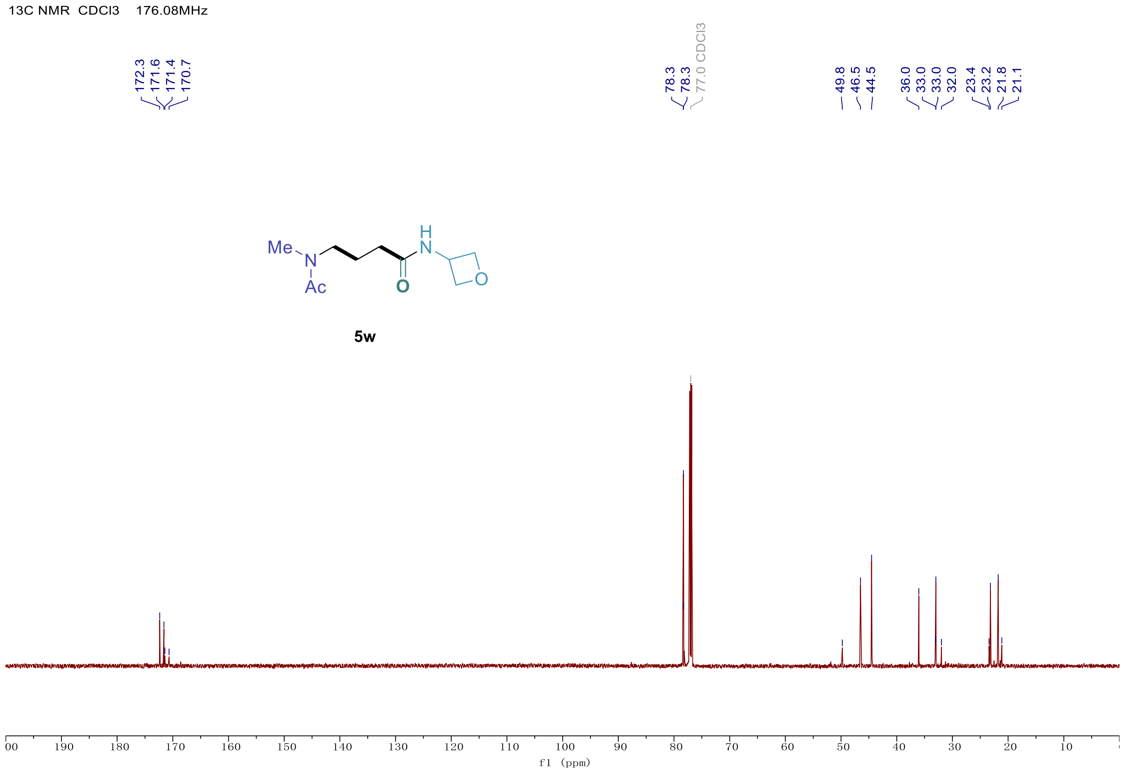

Supplementary Figure 104. ^{13}C NMR of compound **5w** (176 MHz, $CDCl_3$)

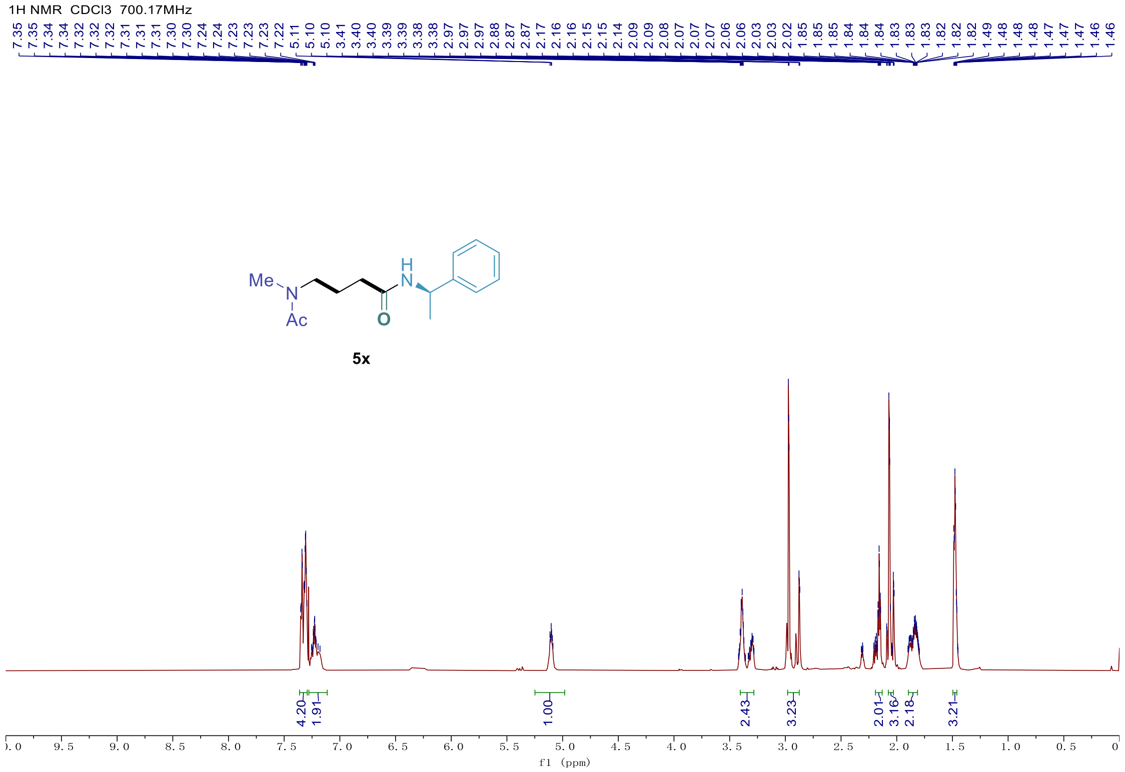

Supplementary Figure 105. ^{1}H NMR of compound **5x** (700 MHz, $CDCl_3$)

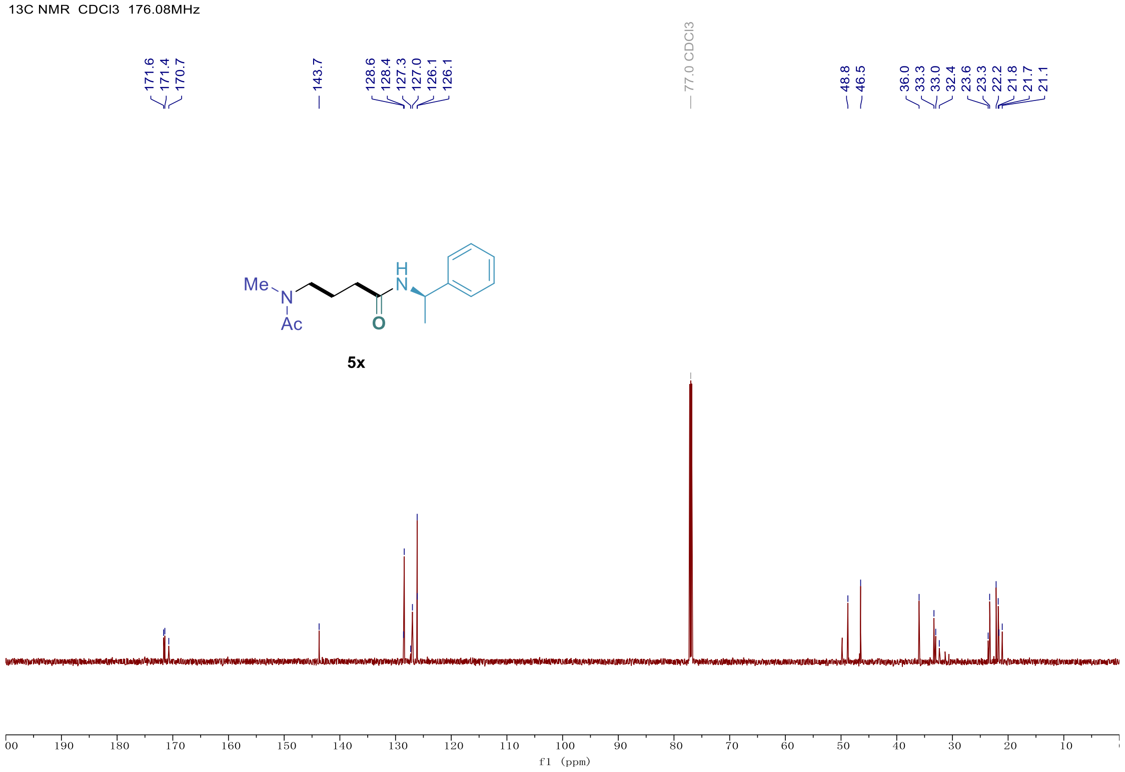

Supplementary Figure 106. ^{13}C NMR of compound **5x** (176 MHz, $CDCl_3$)

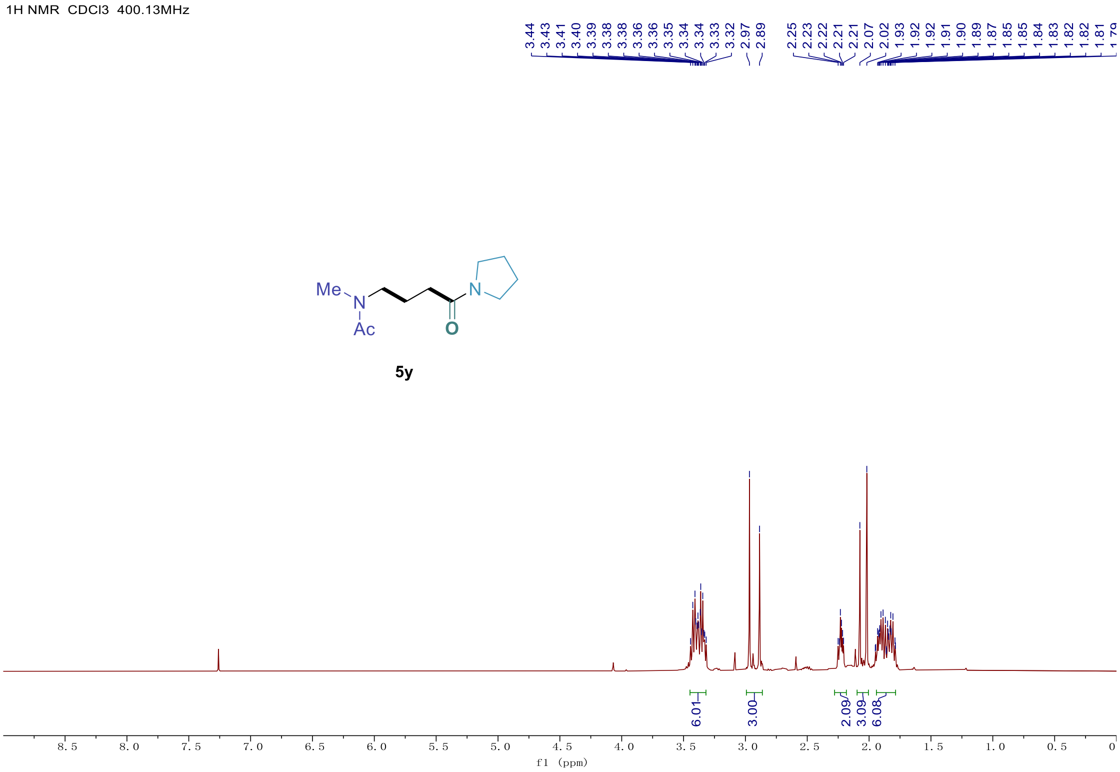

Supplementary Figure 107. ^{1}H NMR of compound **5y** (400 MHz, $CDCl_3$)

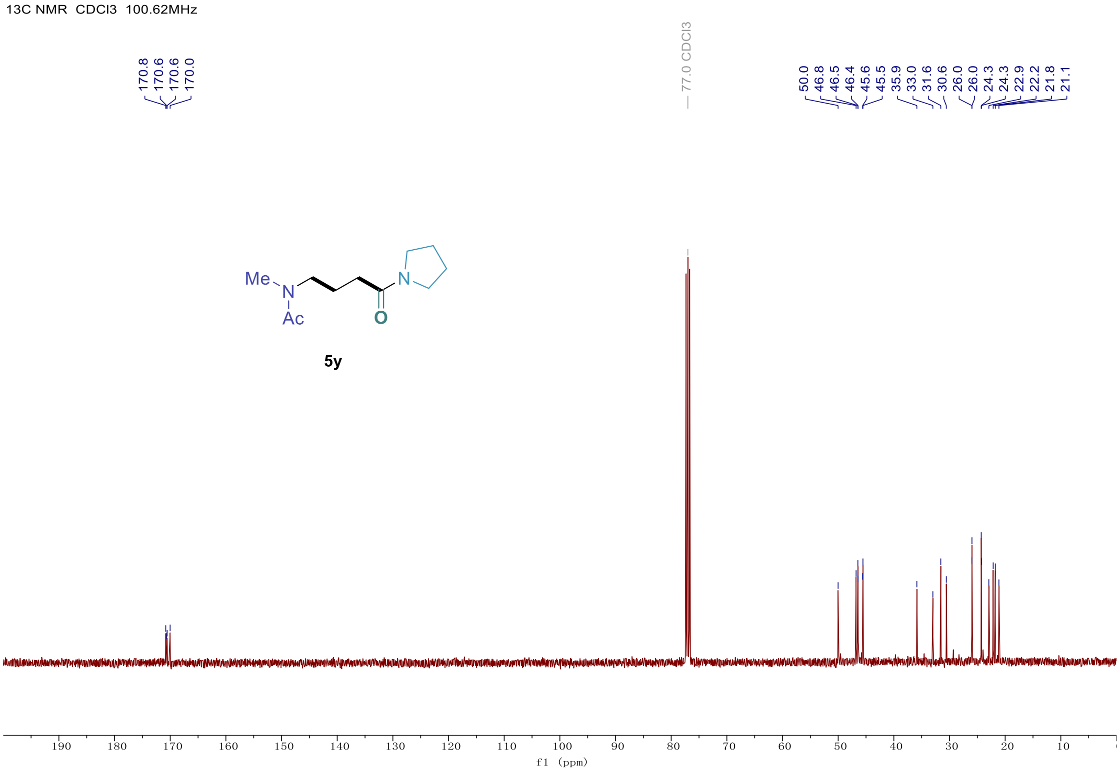

Supplementary Figure 108. ^{13}C NMR of compound **5y** (100 MHz, $CDCl_3$)

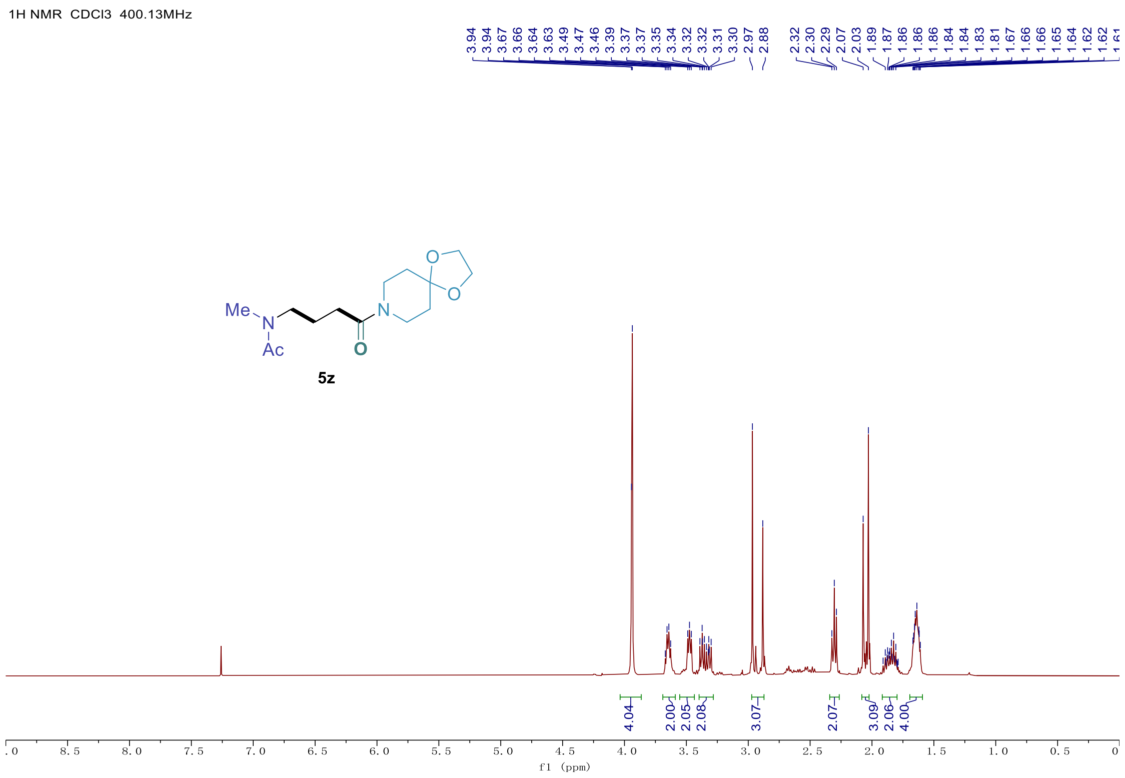

Supplementary Figure 109. ^{1}H NMR of compound **5z** (400 MHz, $CDCl_3$)

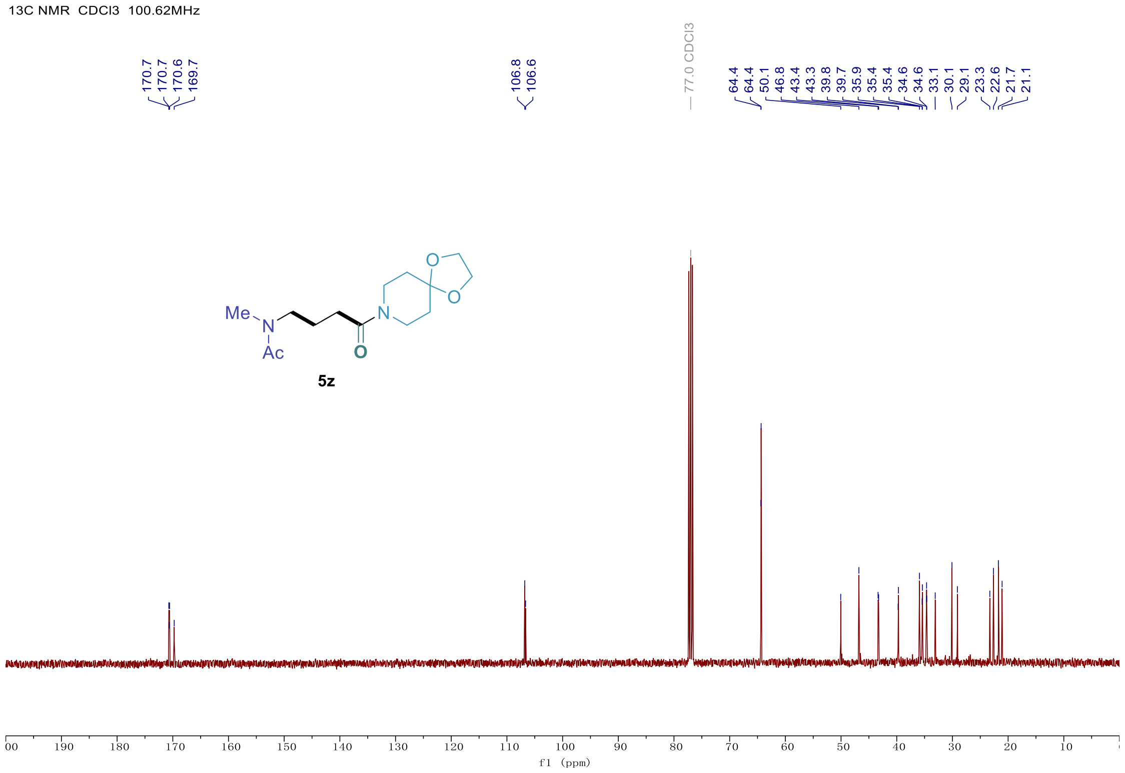

Supplementary Figure 110. ^{13}C NMR of compound **5z** (100 MHz, $CDCl_3$)

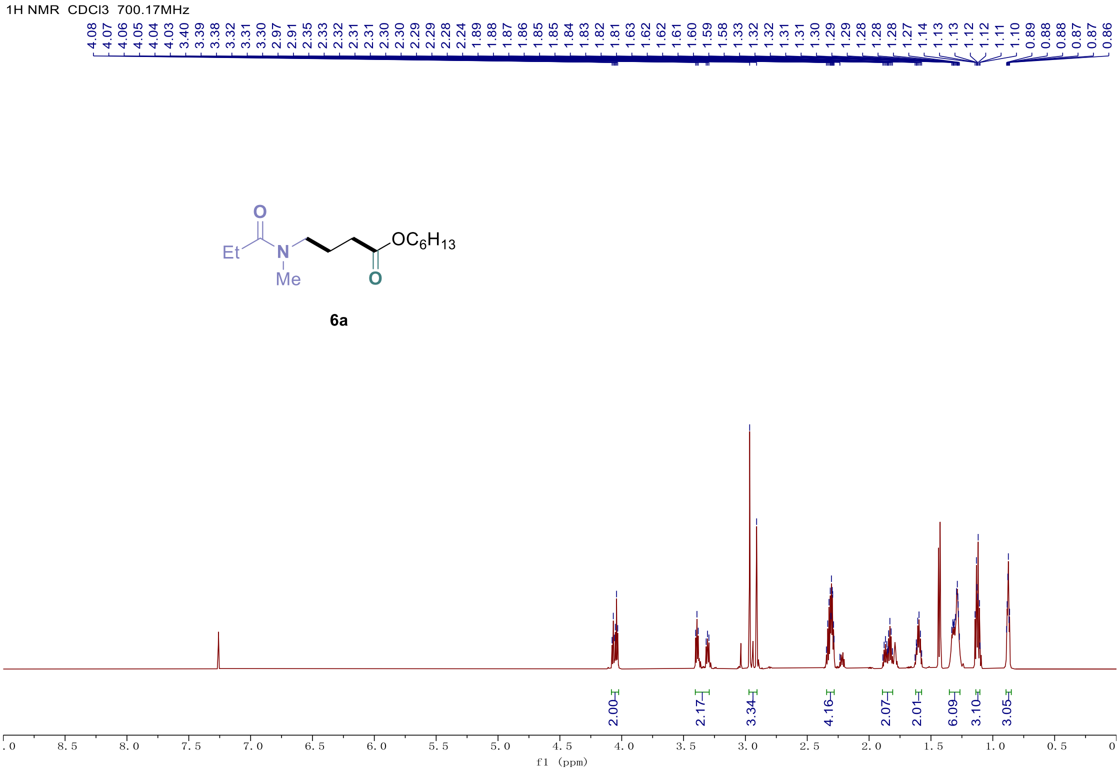

Supplementary Figure 111. ^{1}H NMR of compound **6a** (700 MHz, $CDCl_3$)

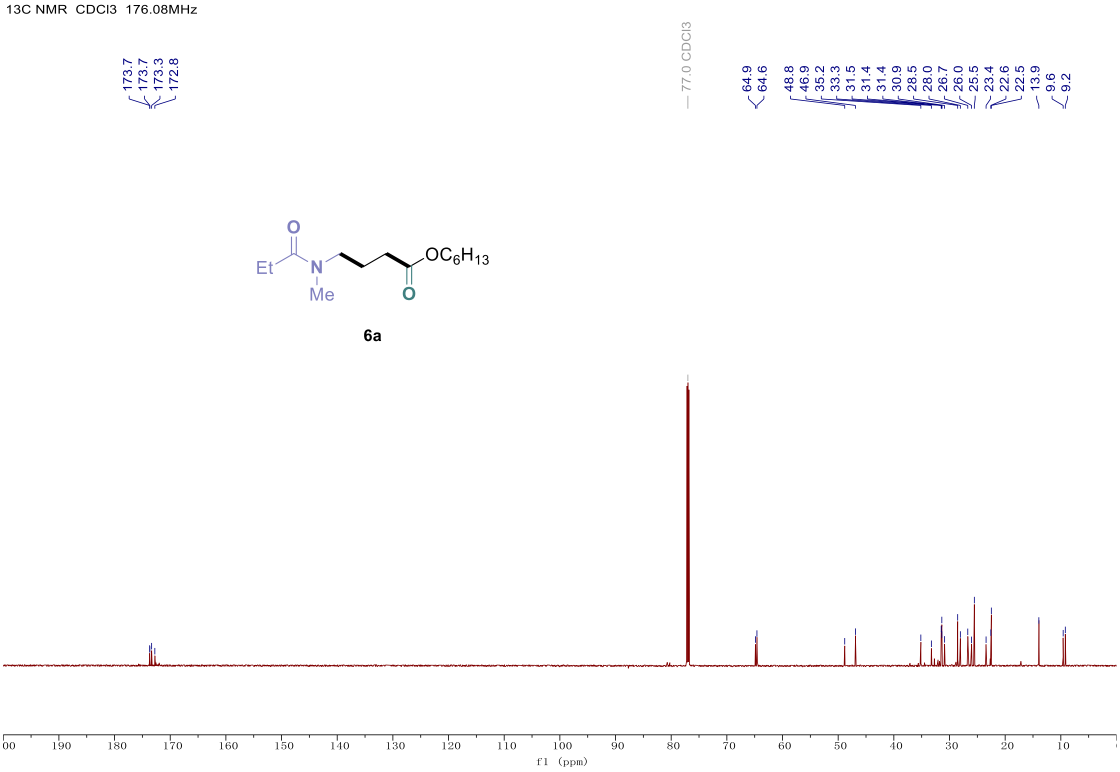

Supplementary Figure 112. ^{13}C NMR of compound **6a** (176 MHz, $CDCl_3$)

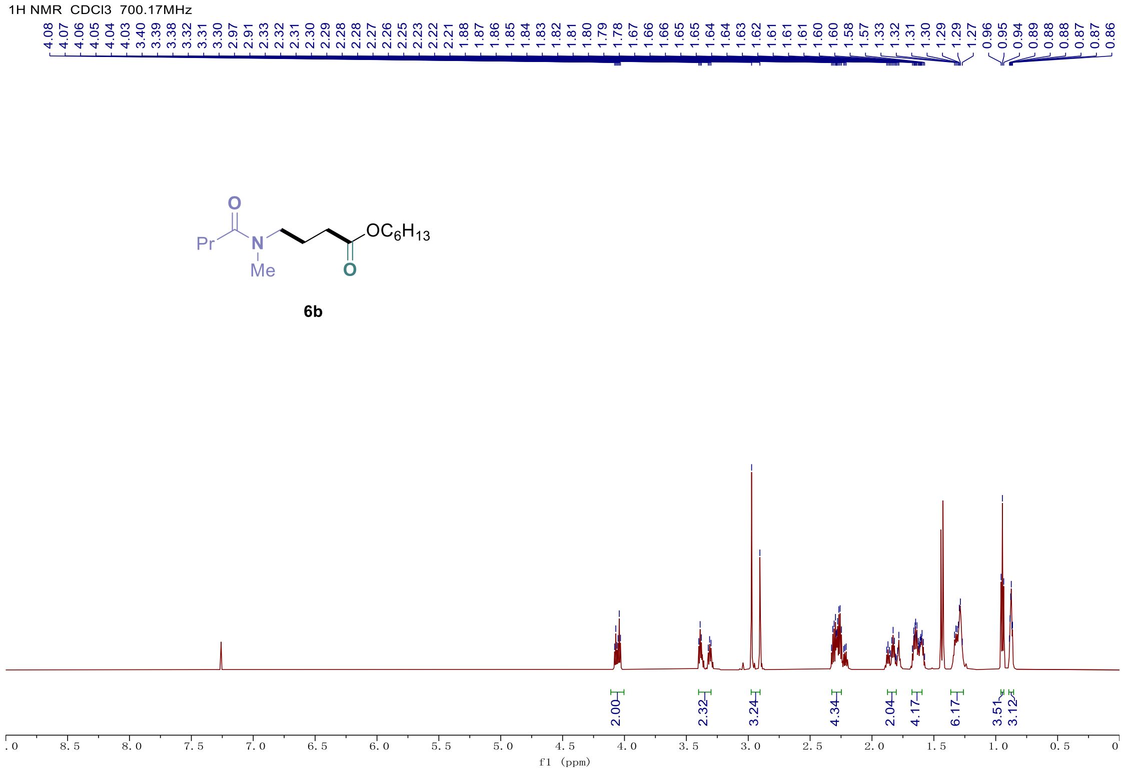

Supplementary Figure 113. ^{1}H NMR of compound **6b** (700 MHz, $CDCl_3$)

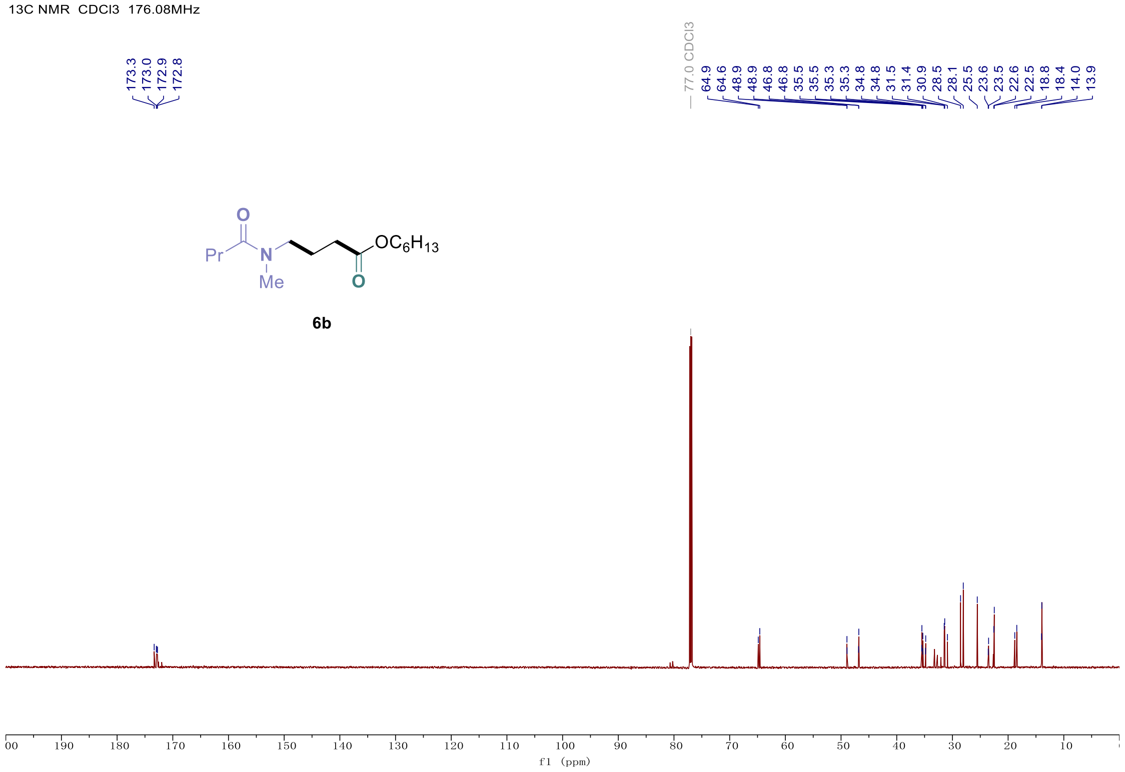

Supplementary Figure 114. ^{13}C NMR of compound **6b** (176 MHz, $CDCl_3$)

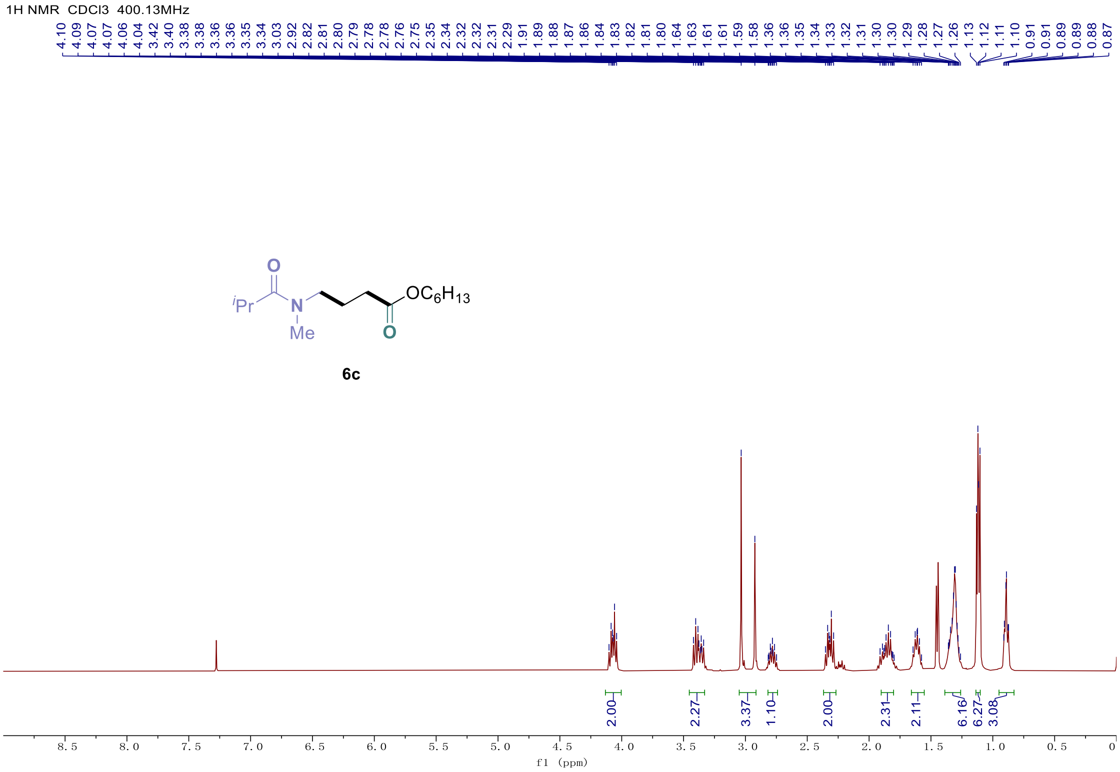

Supplementary Figure 115. ^{1}H NMR of compound **6c** (400 MHz, $CDCl_3$)

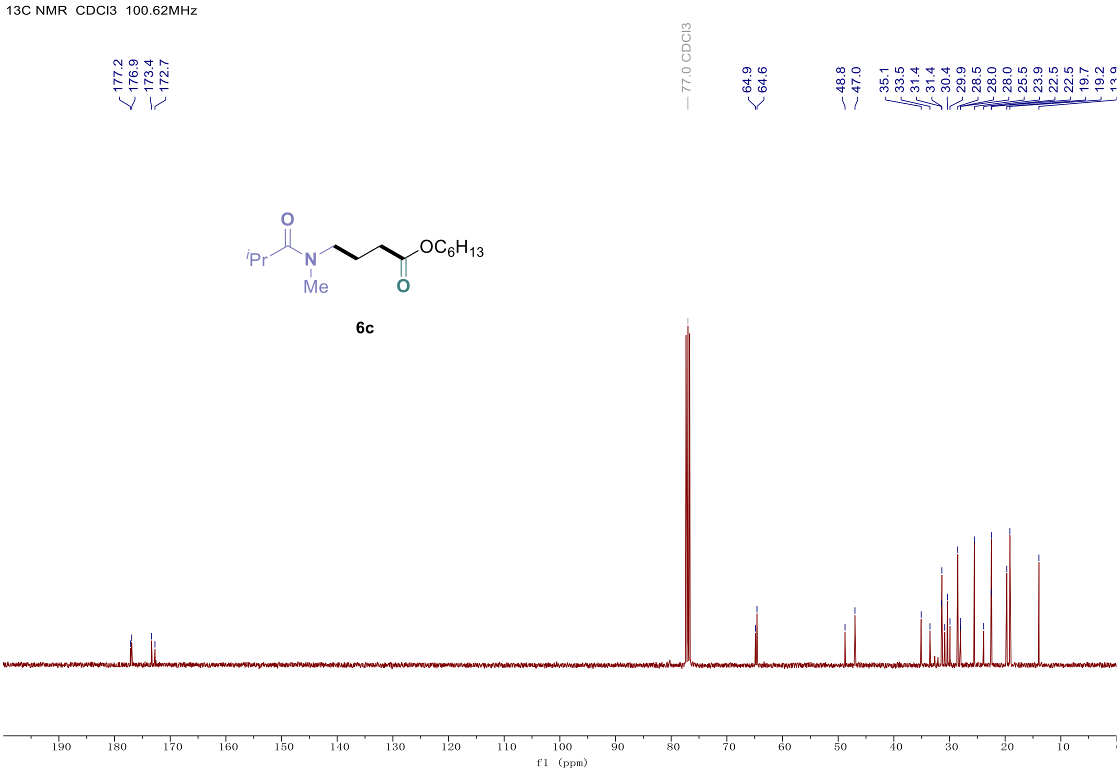

Supplementary Figure 116. ^{13}C NMR of compound **6c** (100 MHz, $CDCl_3$)

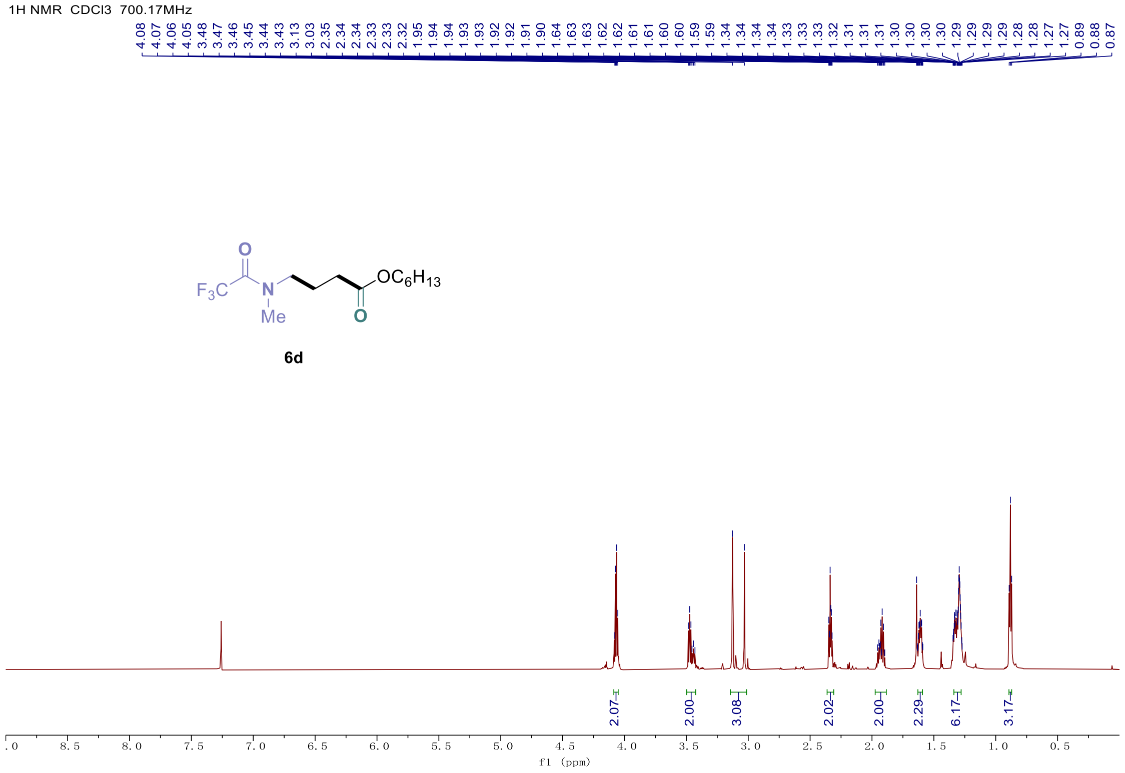

Supplementary Figure 117. ^{1}H NMR of compound **6d** (700 MHz, $CDCl_3$)

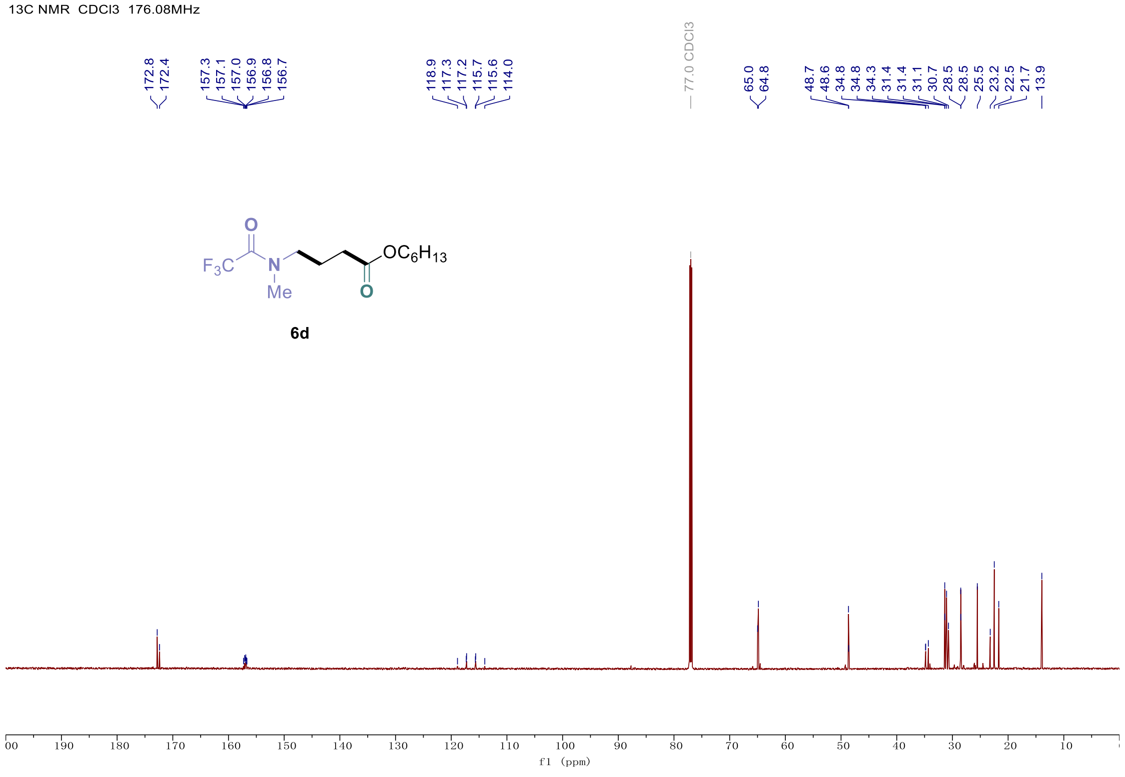

Supplementary Figure 118. ^{13}C NMR of compound **6d** (176 MHz, $CDCl_3$)

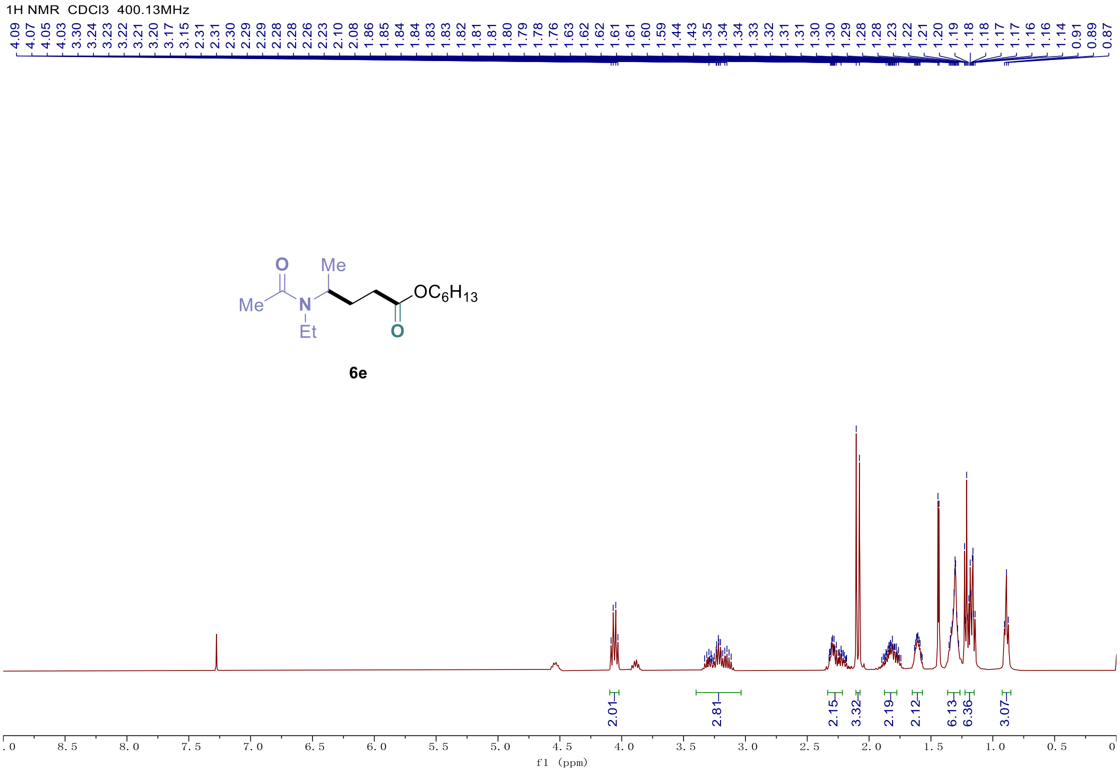

Supplementary Figure 119. ^{1}H NMR of compound **6e** (400 MHz, $CDCl_3$)

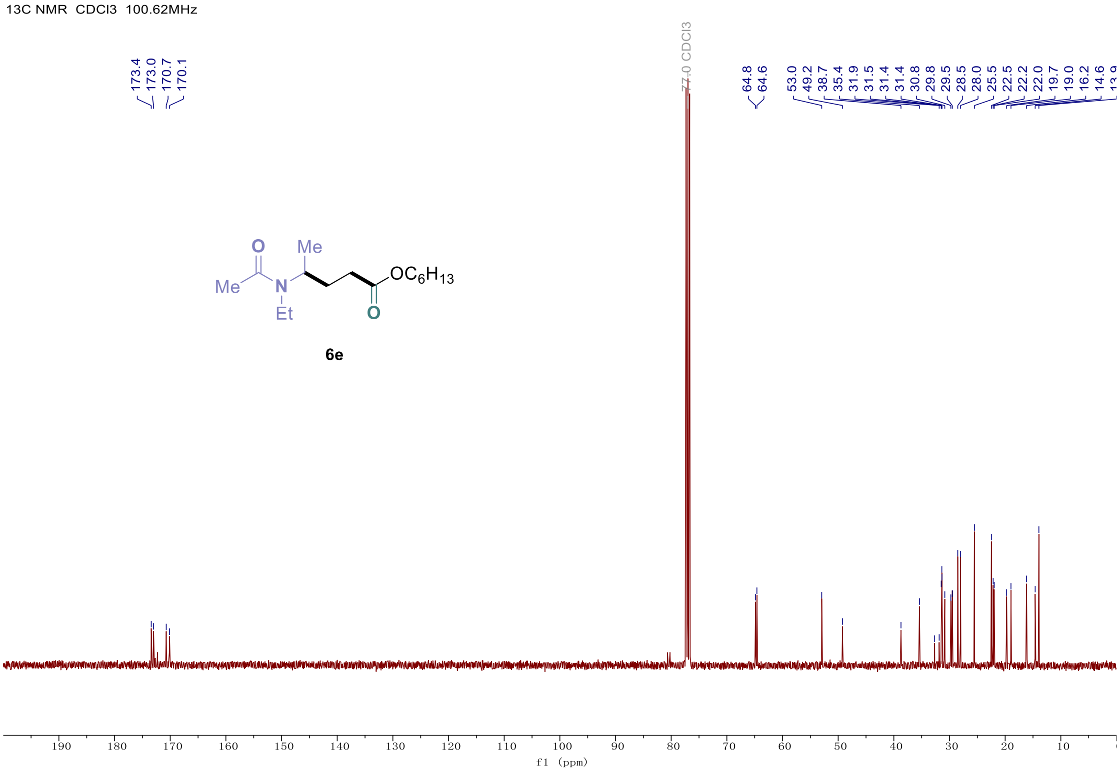

Supplementary Figure 120. ^{13}C NMR of compound **6e** (100 MHz, $CDCl_3$)

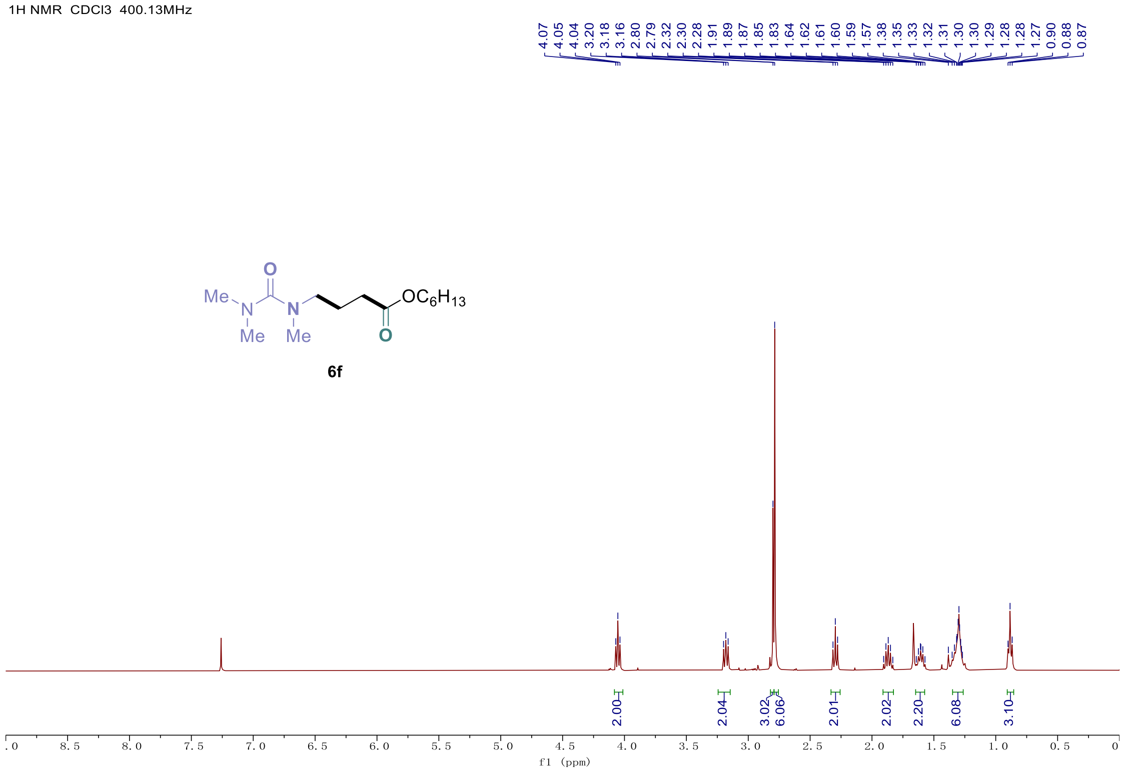

Supplementary Figure 121. ^{1}H NMR of compound **6f** (400 MHz, $CDCl_3$)

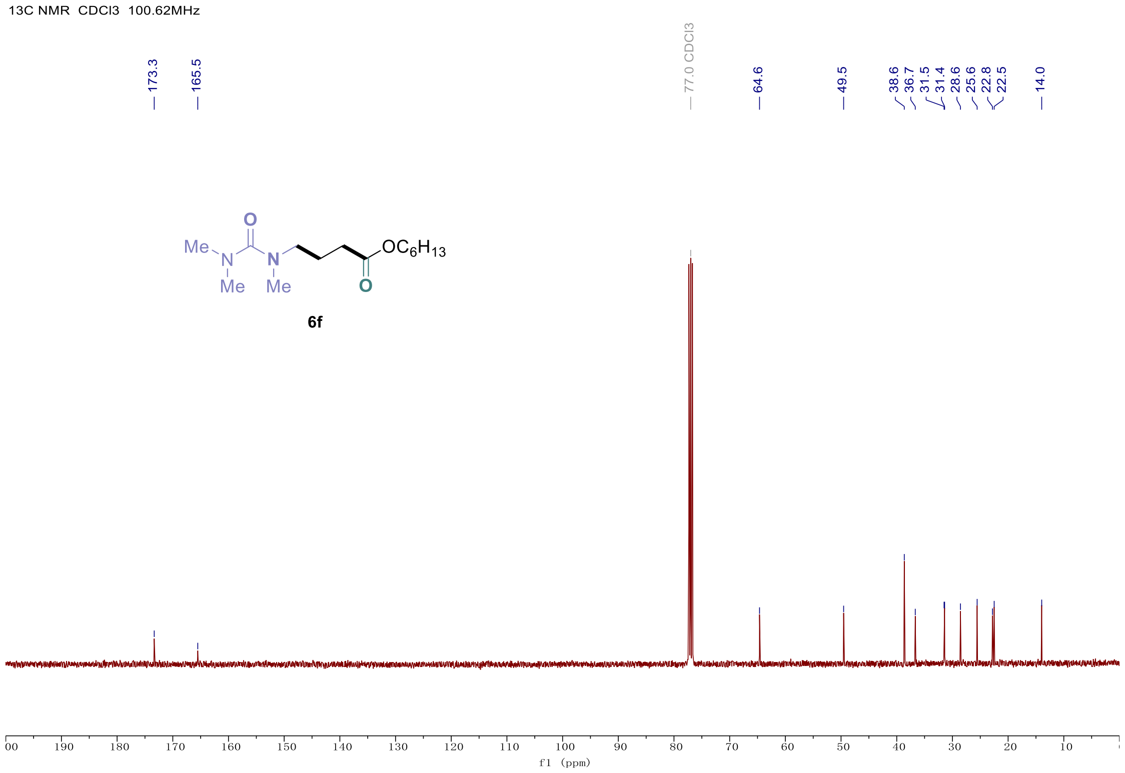

Supplementary Figure 122. ^{13}C NMR of compound **6f** (100 MHz, $CDCl_3$)

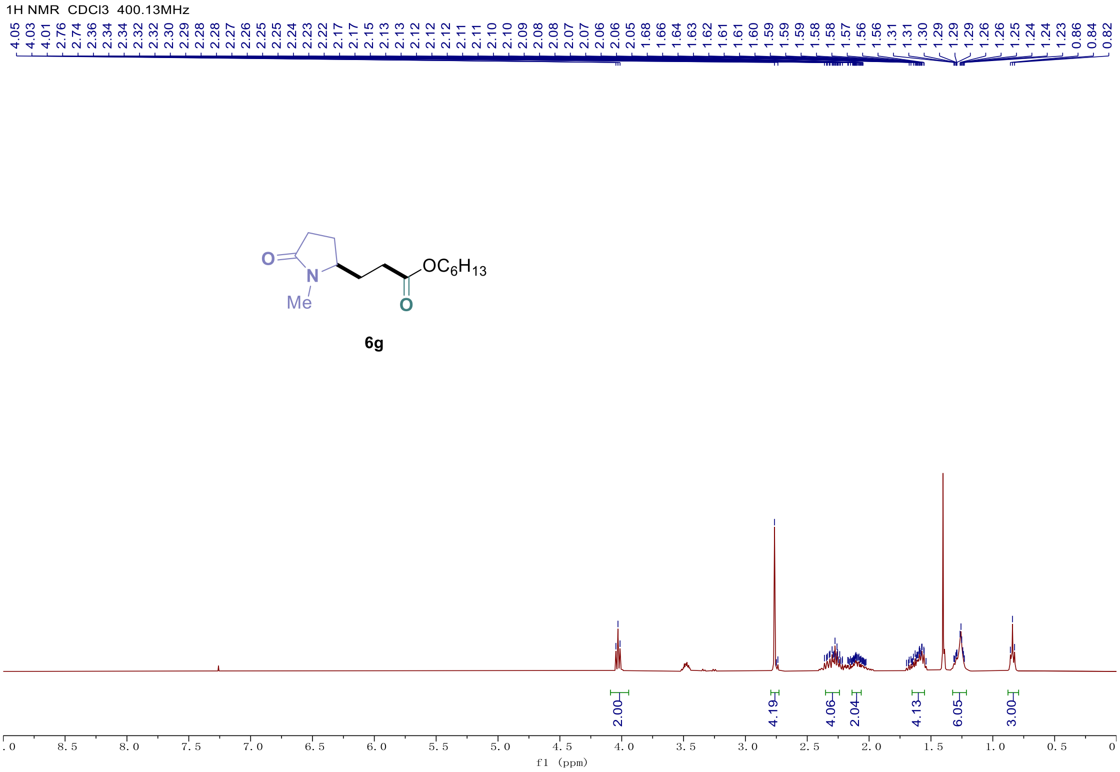

Supplementary Figure 123. ^{1}H NMR of compound **6g** (400 MHz, $CDCl_3$)

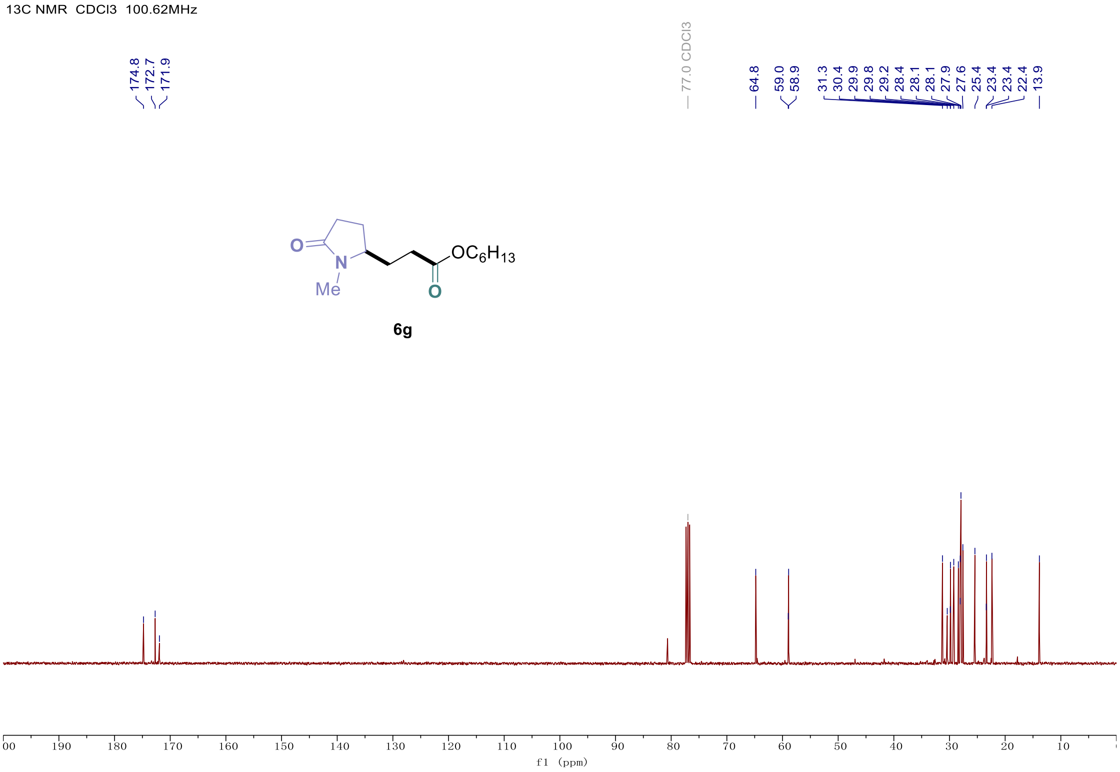

Supplementary Figure 124. ^{13}C NMR of compound **6g** (100 MHz, $CDCl_3$)

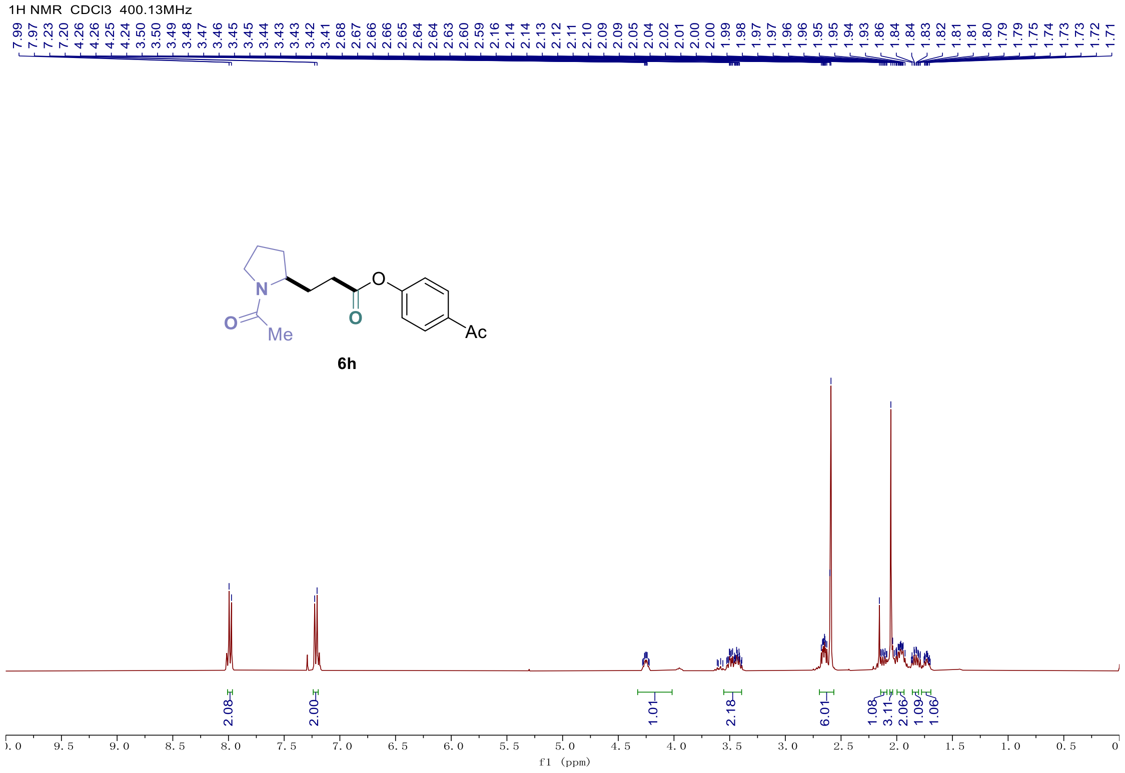

Supplementary Figure 125. ^{1}H NMR of compound **6h** (400 MHz, $CDCl_3$)

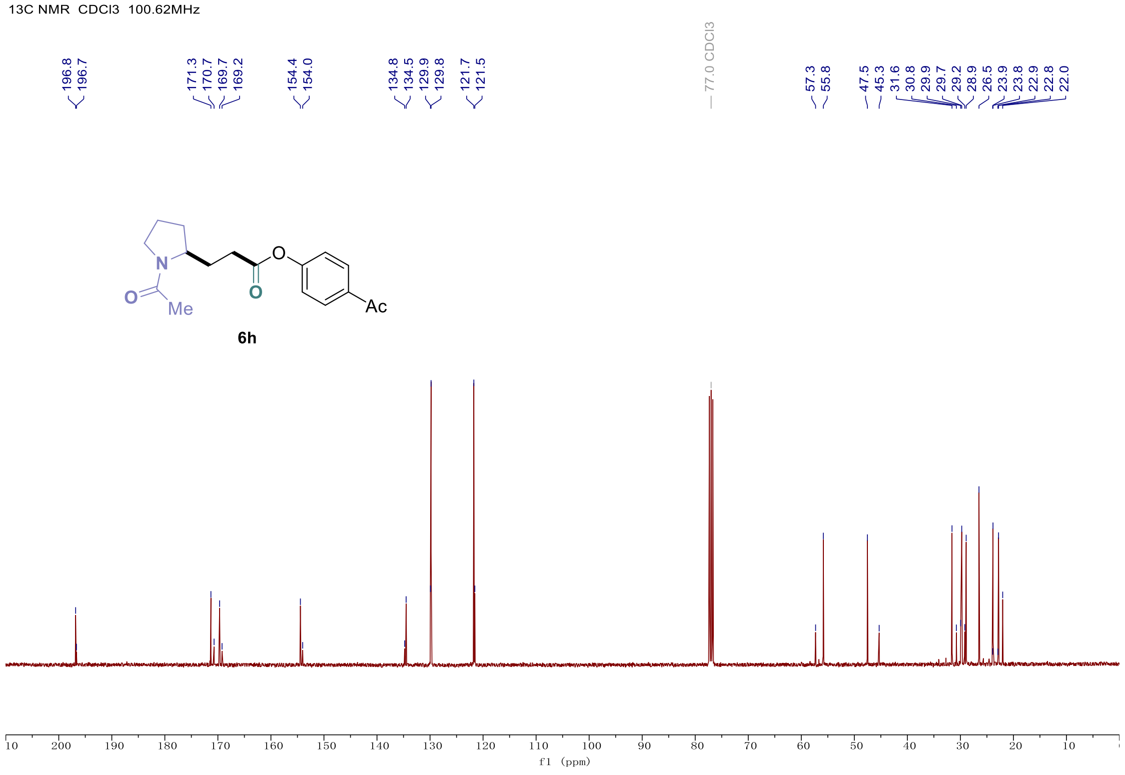

Supplementary Figure 126. ^{13}C NMR of compound **6h** (100 MHz, $CDCl_3$)

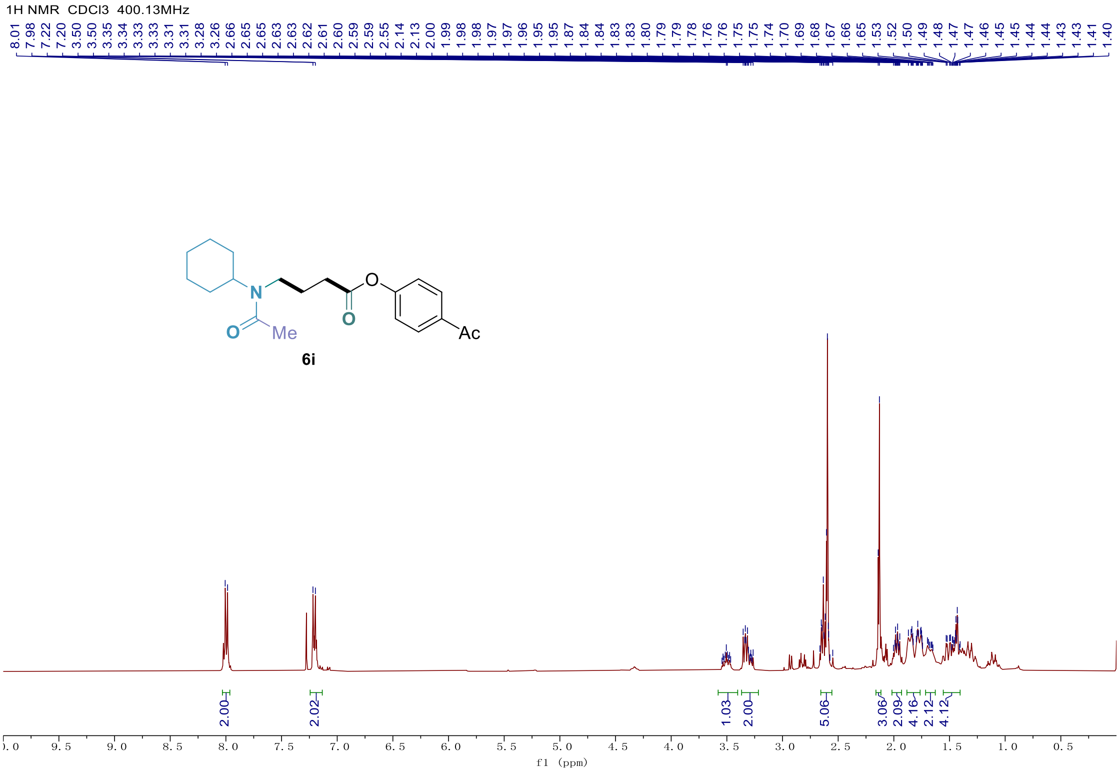

Supplementary Figure 127. ^{1}H NMR of compound **6i** (400 MHz, $CDCl_3$)

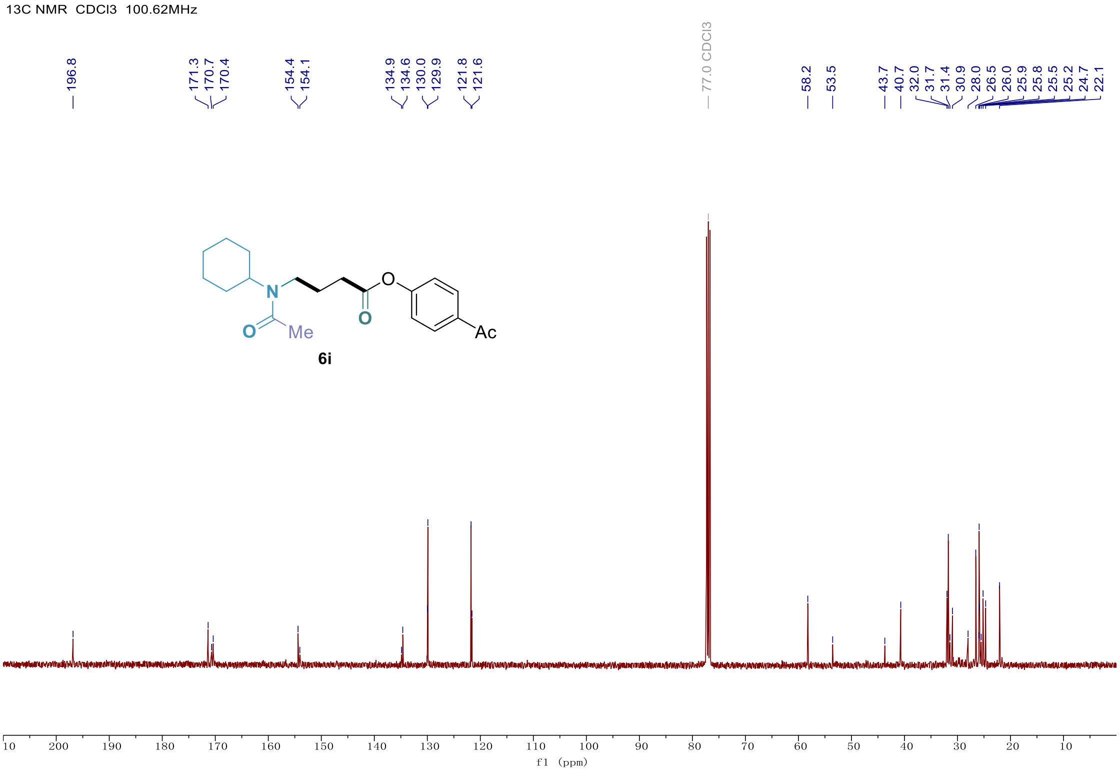

Supplementary Figure 128. ^{13}C NMR of compound **6i** (100 MHz, $CDCl_3$)

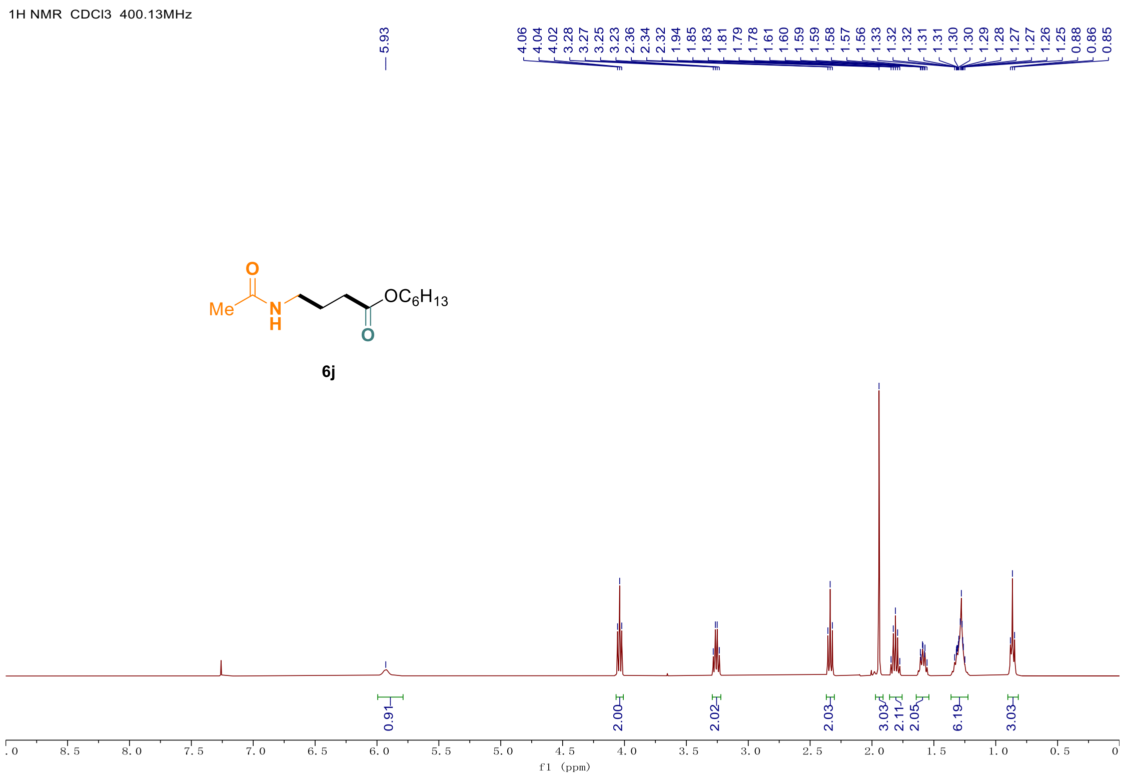

Supplementary Figure 129. ^{1}H NMR of compound **6j** (400 MHz, $CDCl_3$)

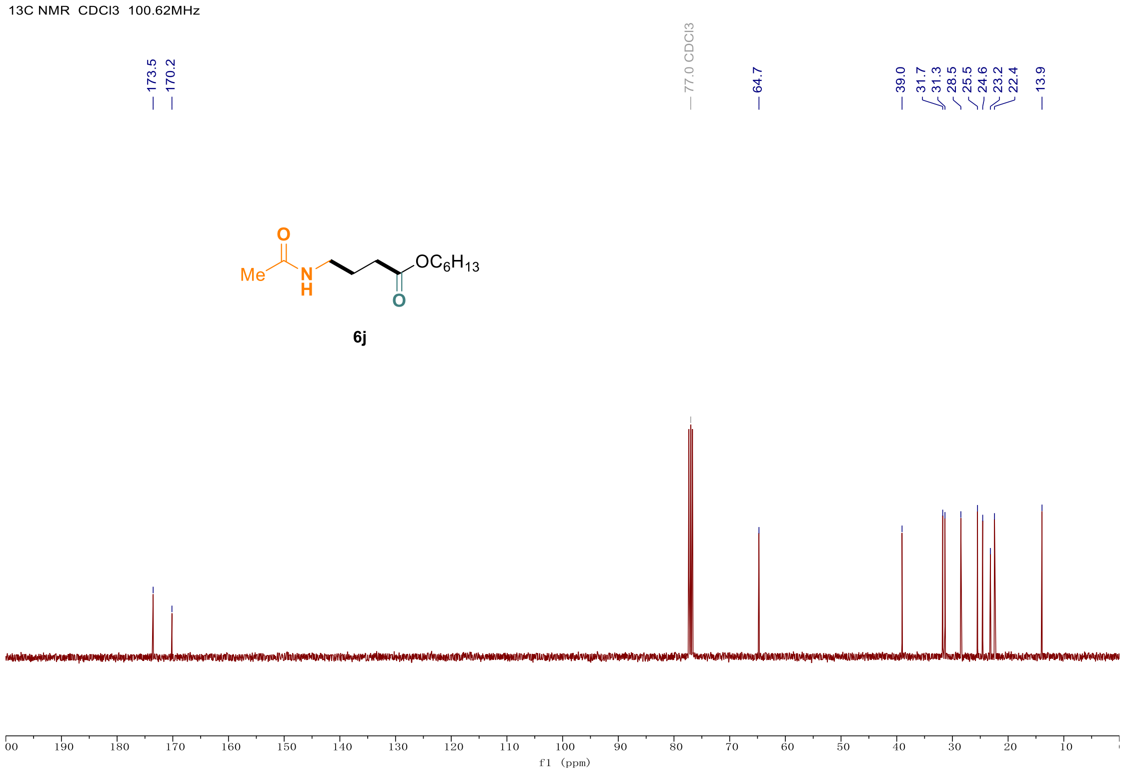

Supplementary Figure 130. ^{13}C NMR of compound **6j** (100 MHz, $CDCl_3$)

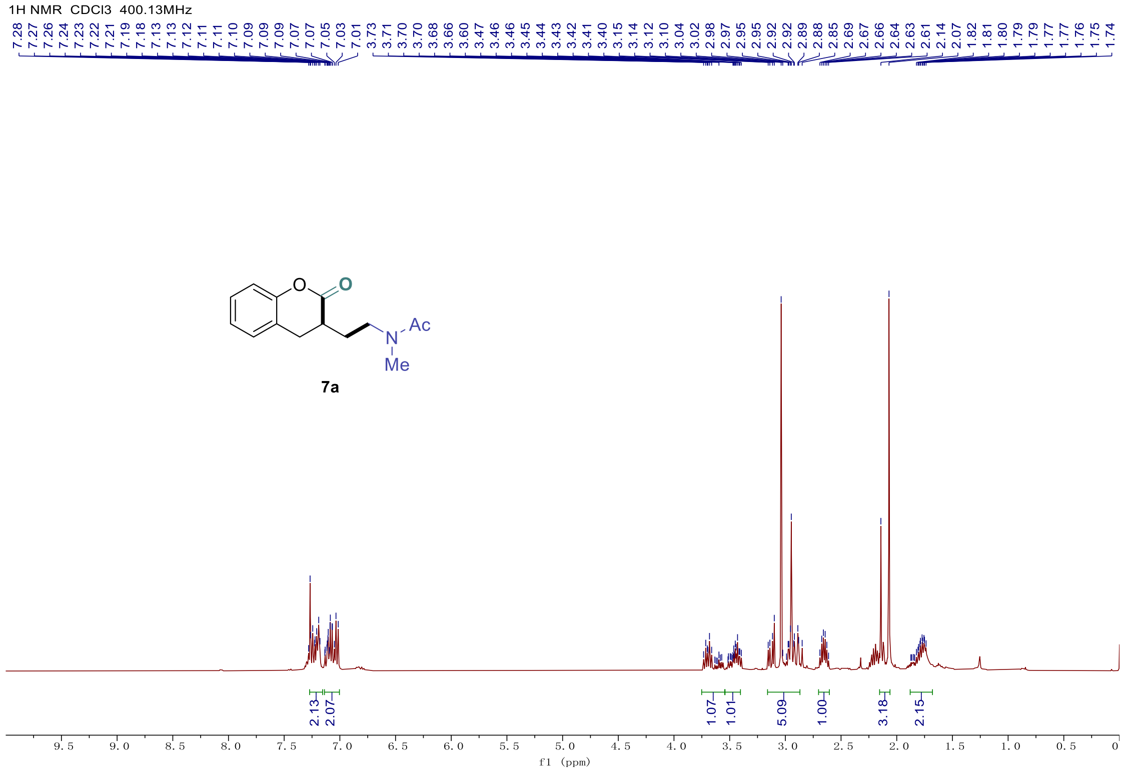

Supplementary Figure 131. ^{1}H NMR of compound **7a** (400 MHz, $CDCl_3$)

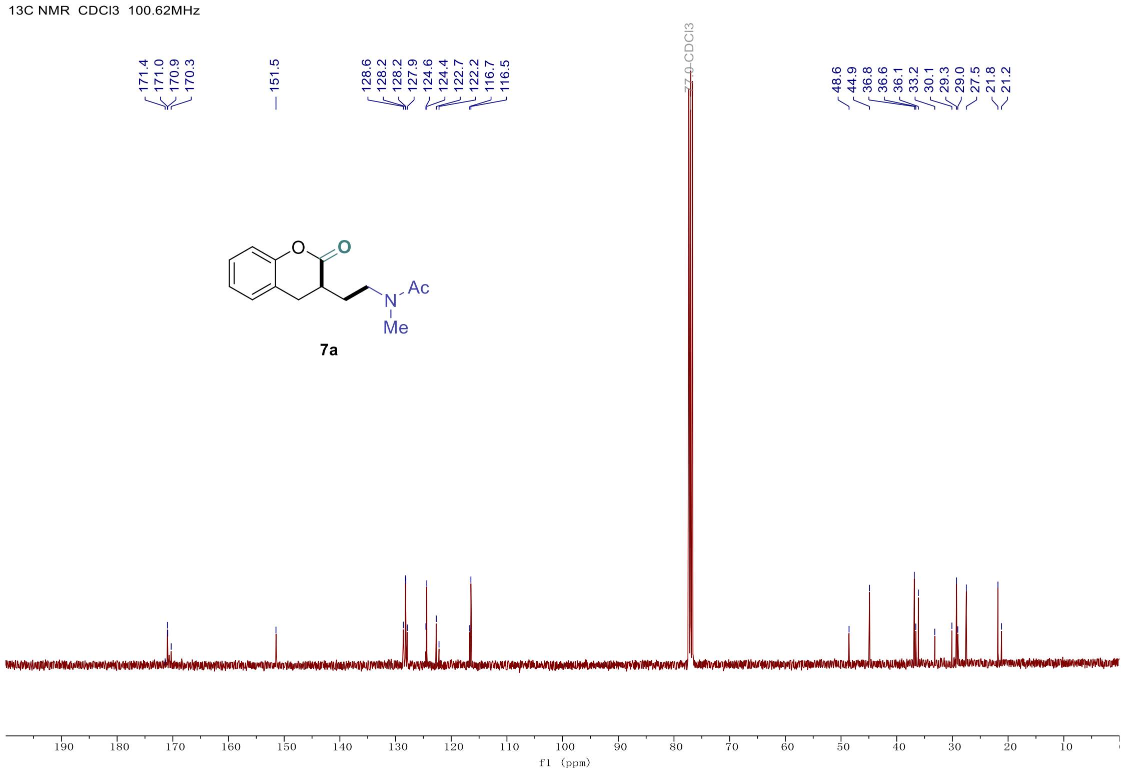

Supplementary Figure 132. ^{13}C NMR of compound **7a** (100 MHz, $CDCl_3$)

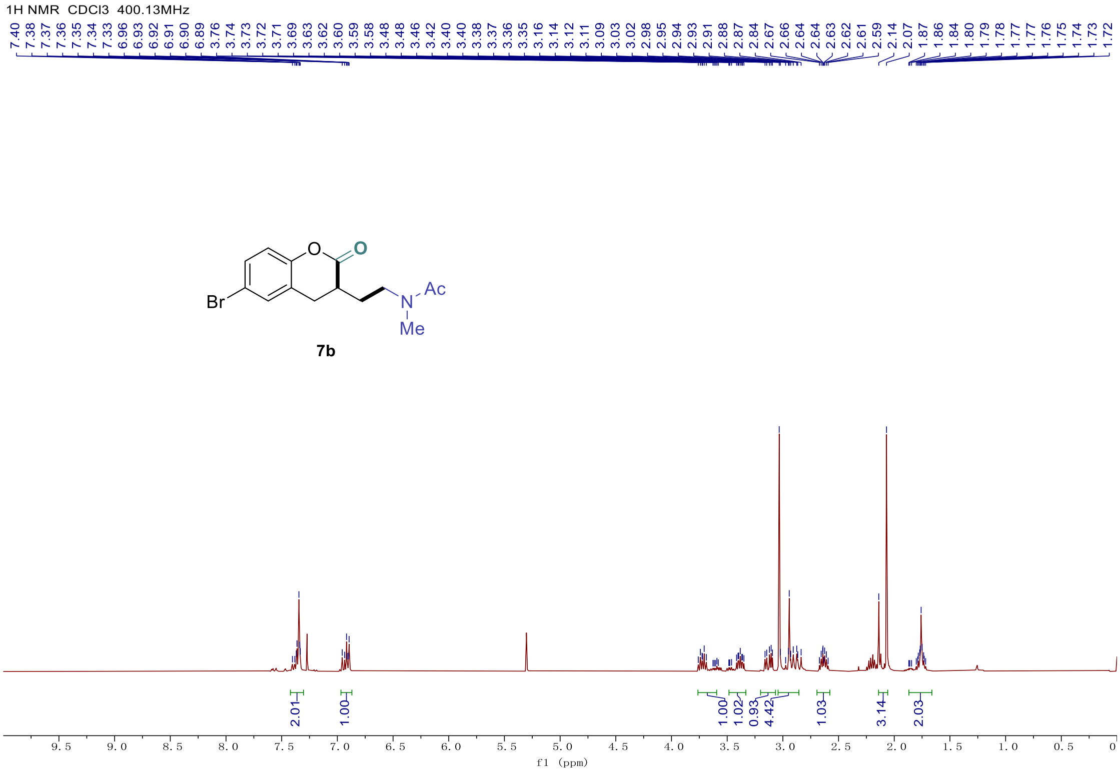

Supplementary Figure 133. ^{1}H NMR of compound **7b** (400 MHz, $CDCl_3$)

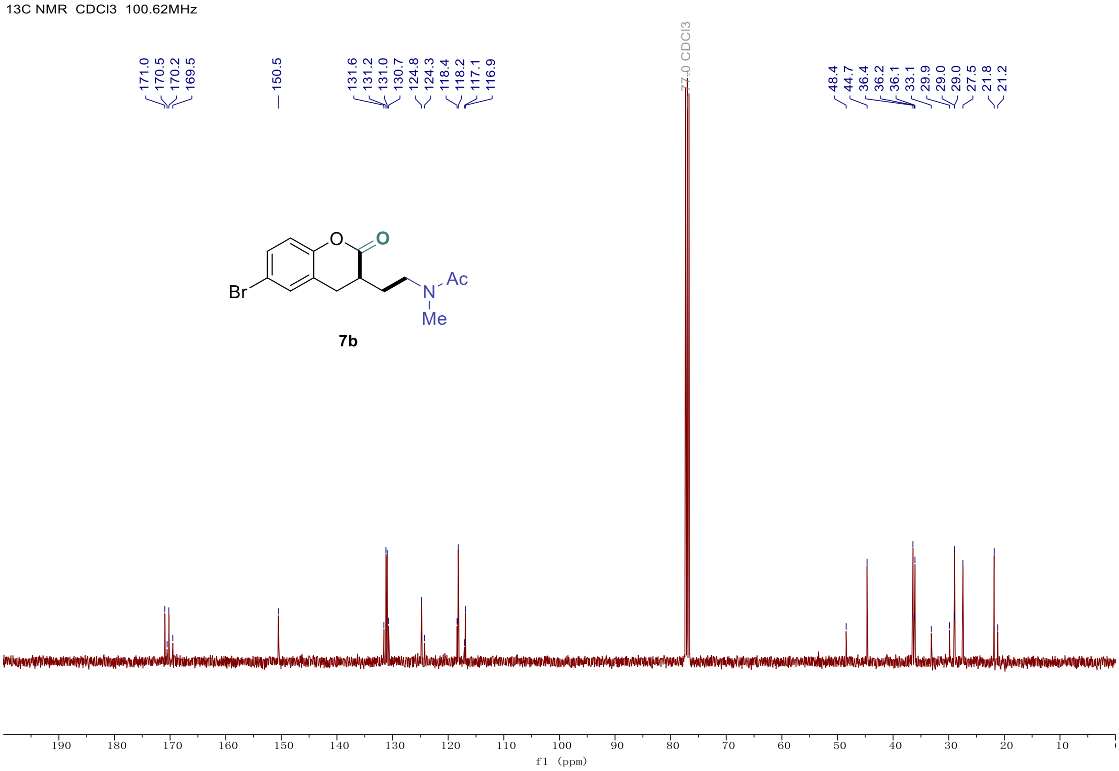

Supplementary Figure 134. ^{13}C NMR of compound **7b** (100 MHz, $CDCl_3$)

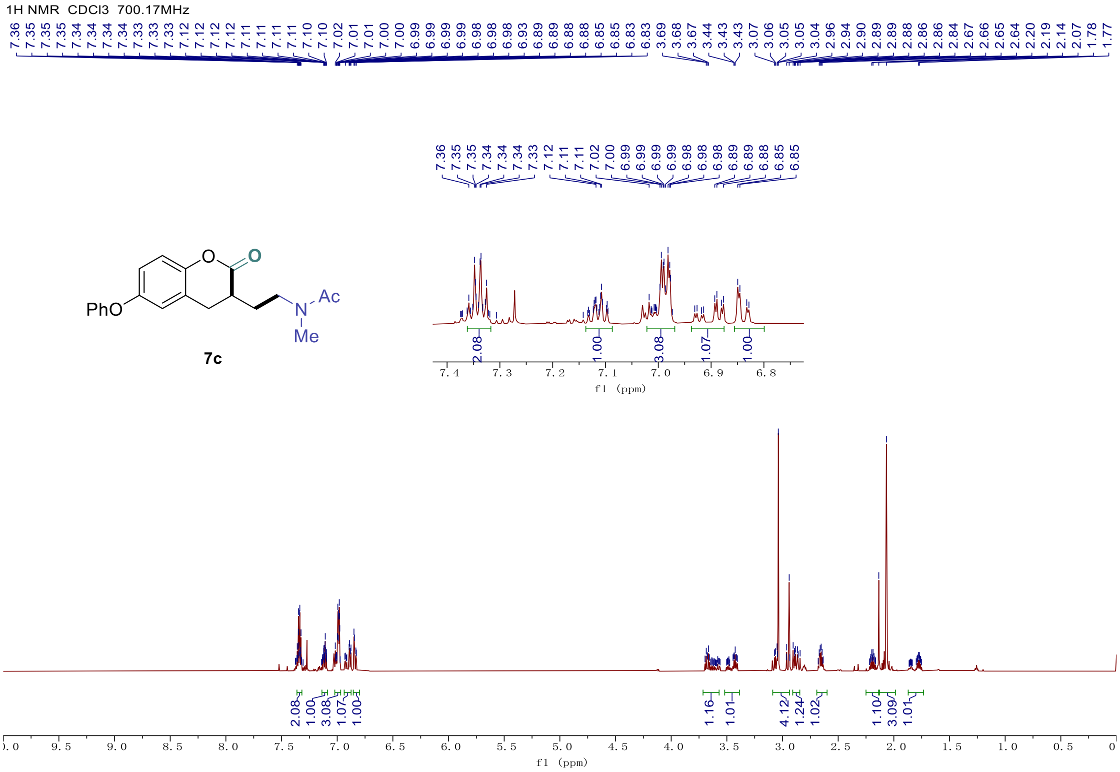

Supplementary Figure 135. ^{1}H NMR of compound **7c** (700 MHz, $CDCl_3$)

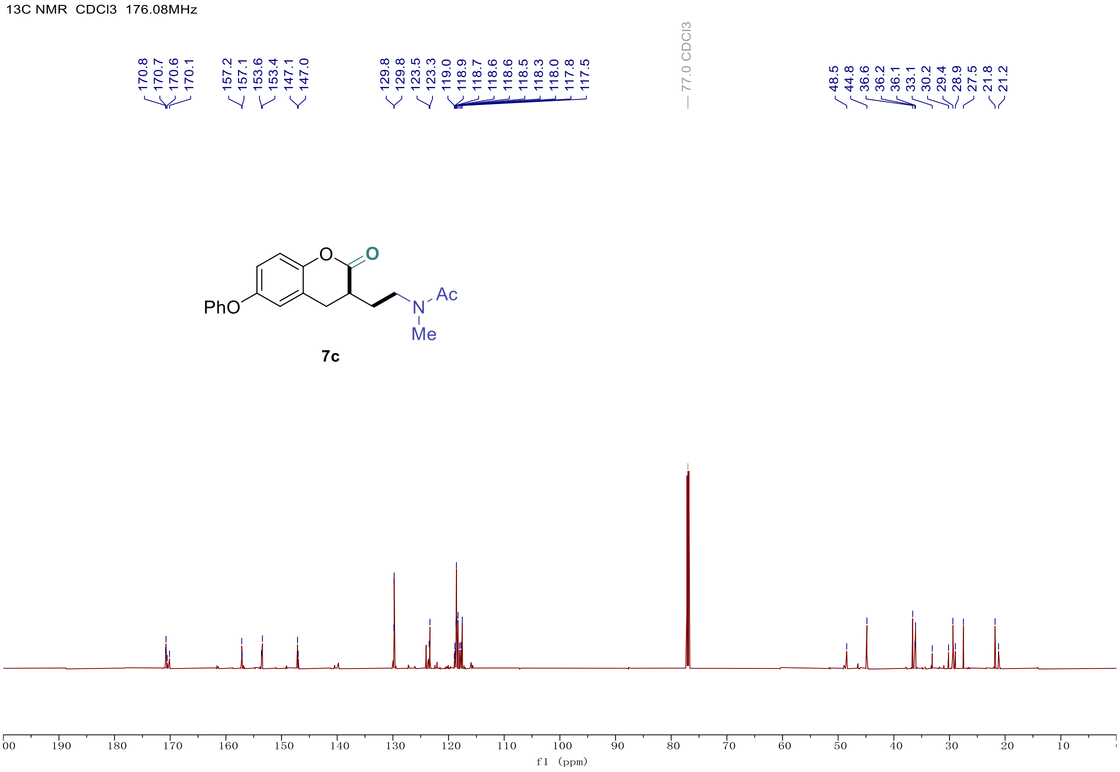

Supplementary Figure 136. ^{13}C NMR of compound **7c** (176 MHz, $CDCl_3$)

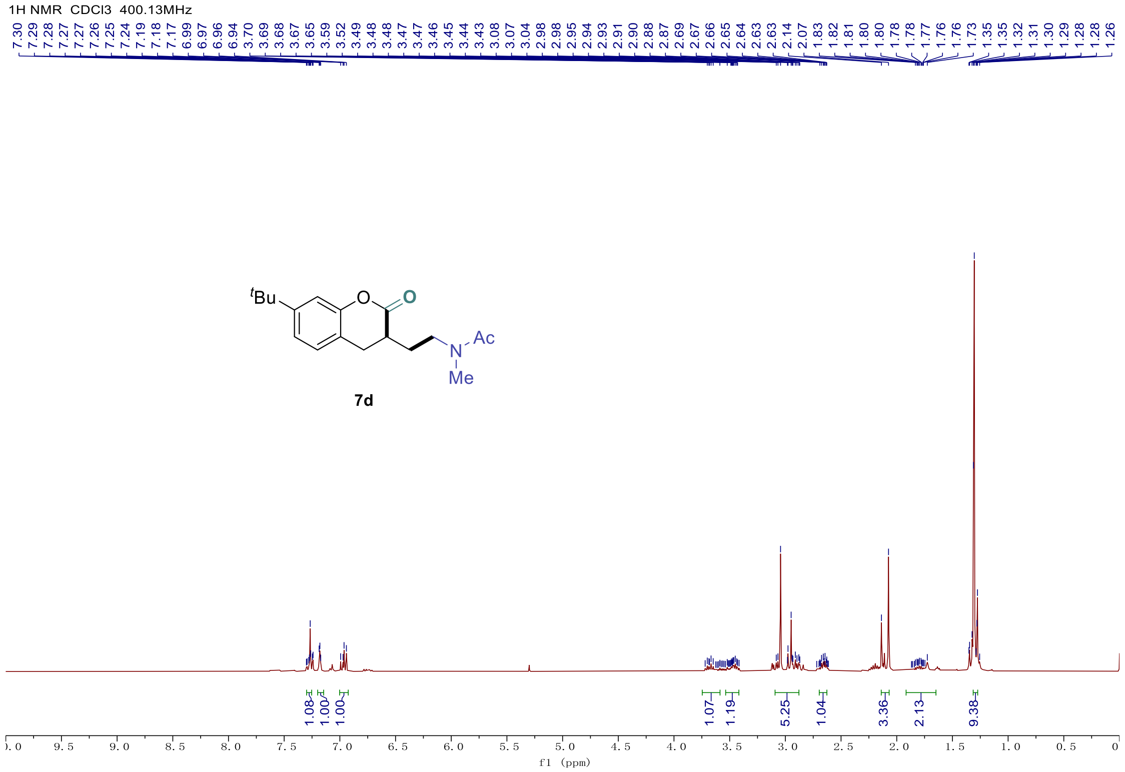

Supplementary Figure 137. ^{1}H NMR of compound **7d** (400 MHz, $CDCl_3$)

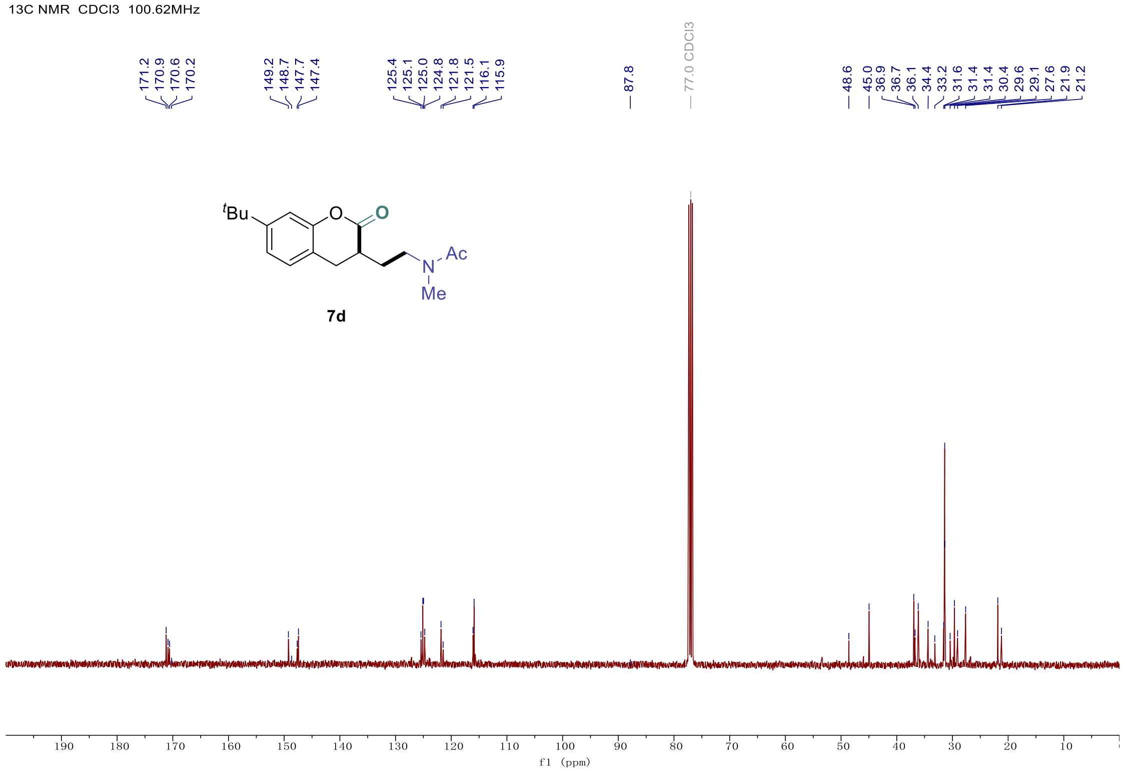

Supplementary Figure 138. ^{13}C NMR of compound **7d** (100 MHz, $CDCl_3$)

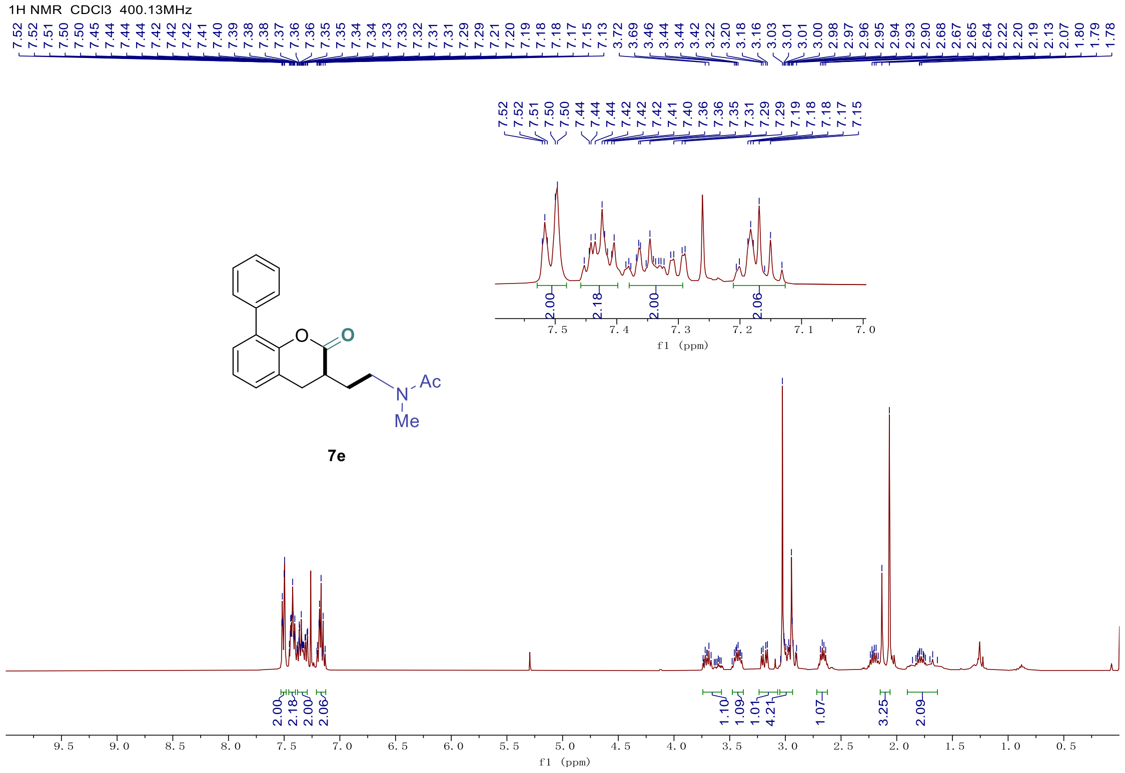

Supplementary Figure 139. ^{1}H NMR of compound **7e** (400 MHz, $CDCl_3$)

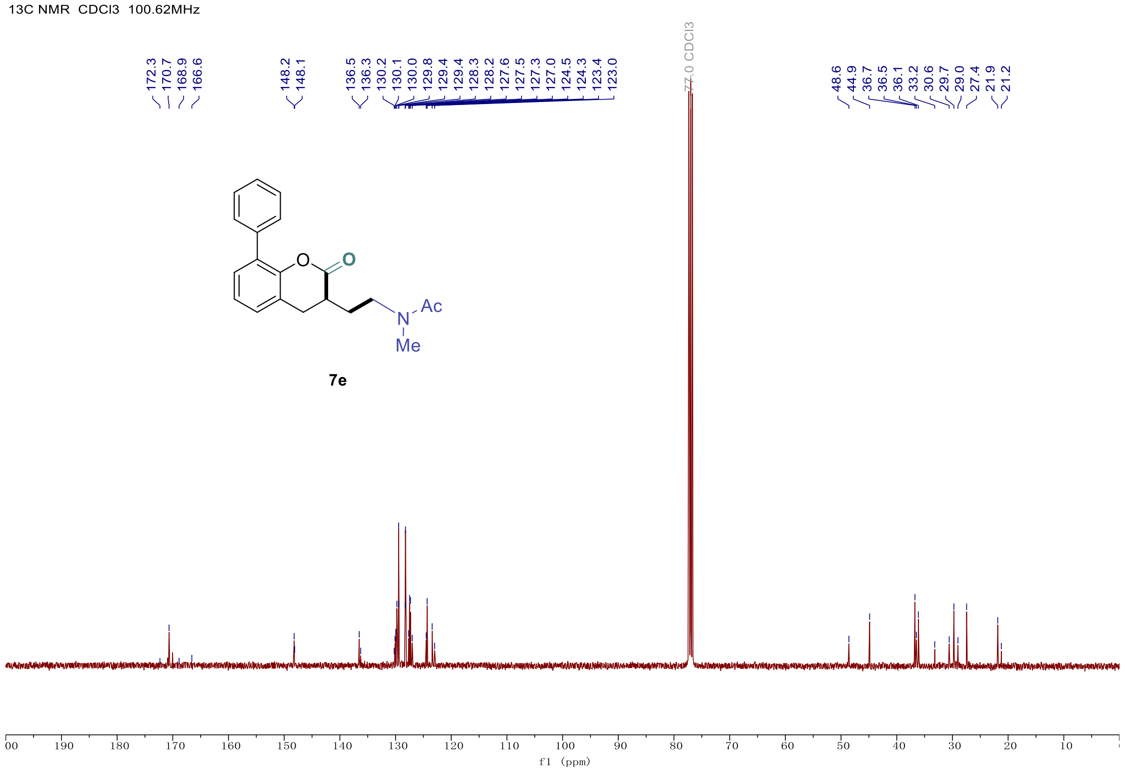

Supplementary Figure 140. ^{13}C NMR of compound **7e** (100 MHz, $CDCl_3$)

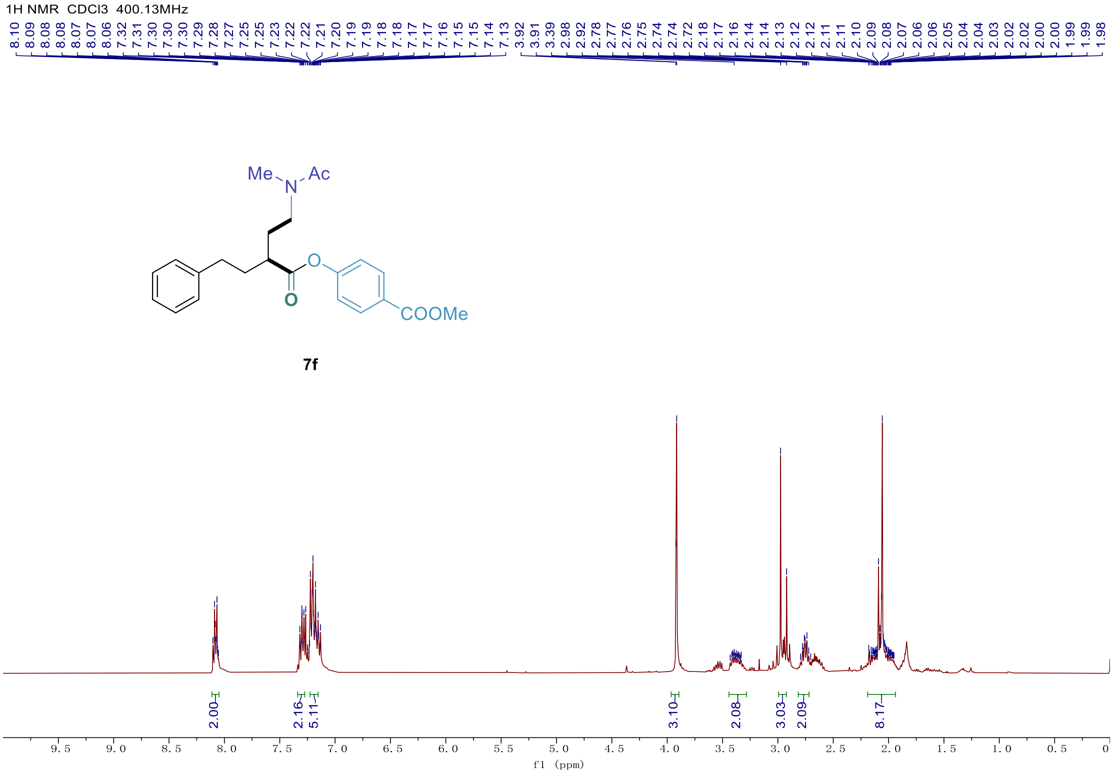

Supplementary Figure 141. ^{1}H NMR of compound **7f** (400 MHz, $CDCl_3$)

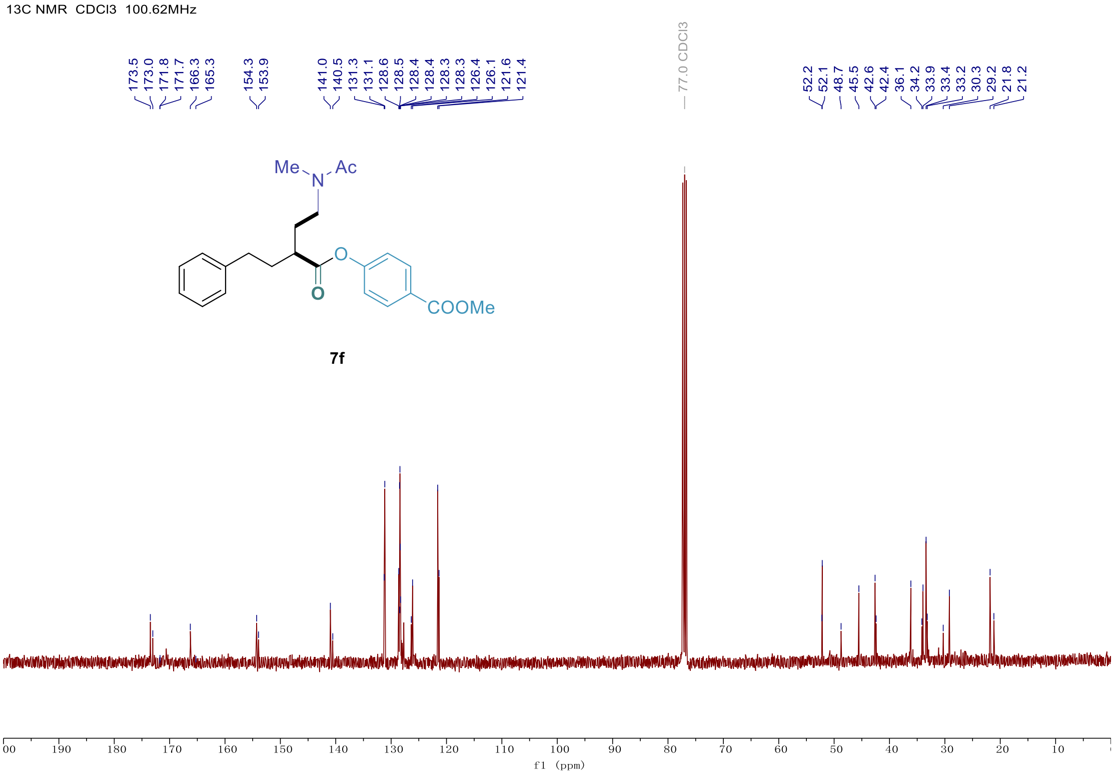

Supplementary Figure 142. ^{13}C NMR of compound **7f** (100 MHz, $CDCl_3$)

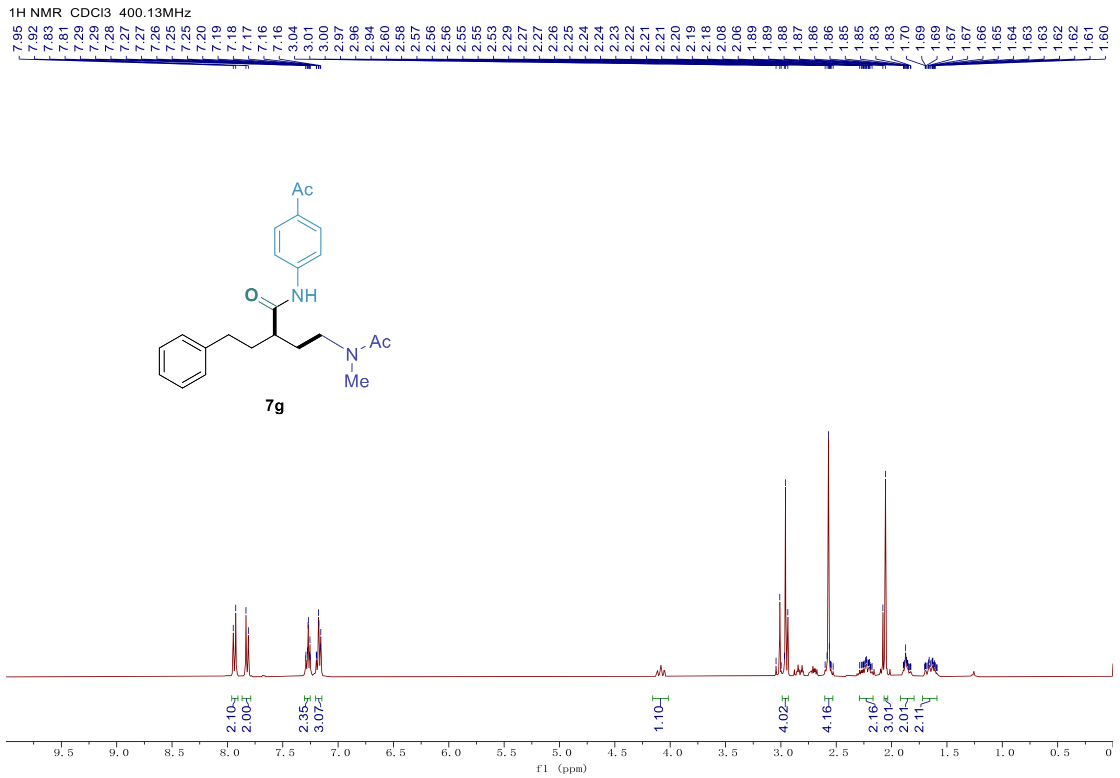

Supplementary Figure 143. ^{1}H NMR of compound **7g** (400 MHz, $CDCl_3$)

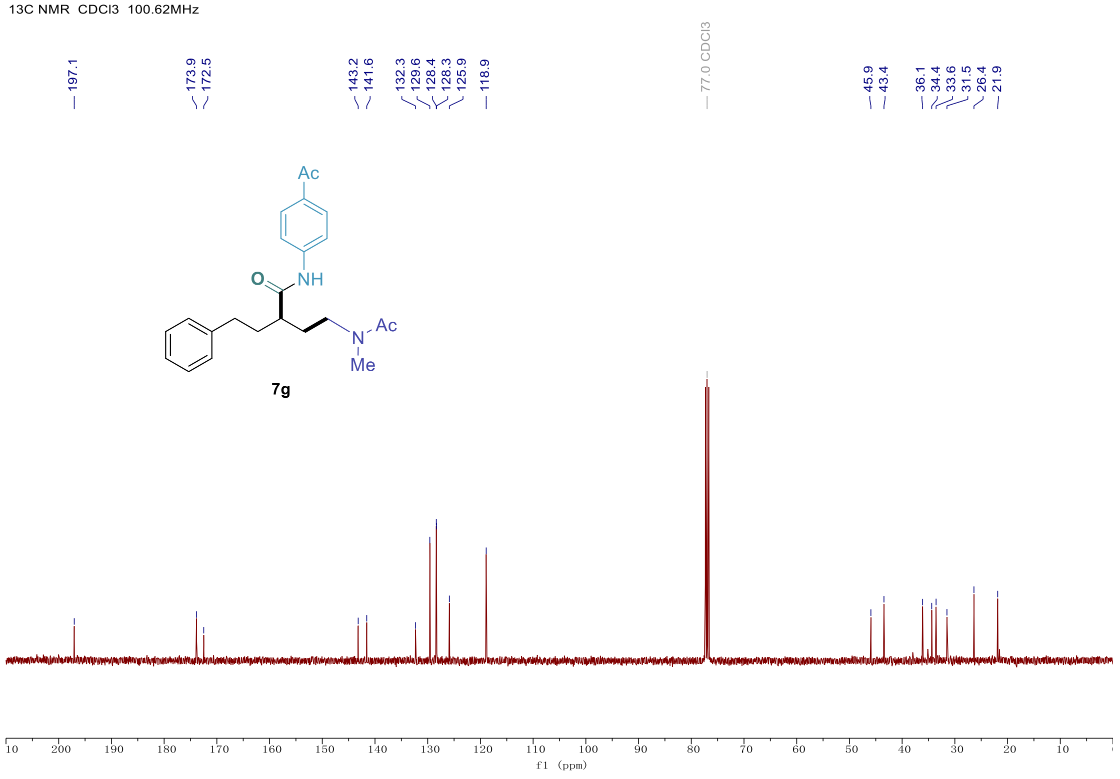

Supplementary Figure 144. ^{13}C NMR of compound **7g** (100 MHz, $CDCl_3$)

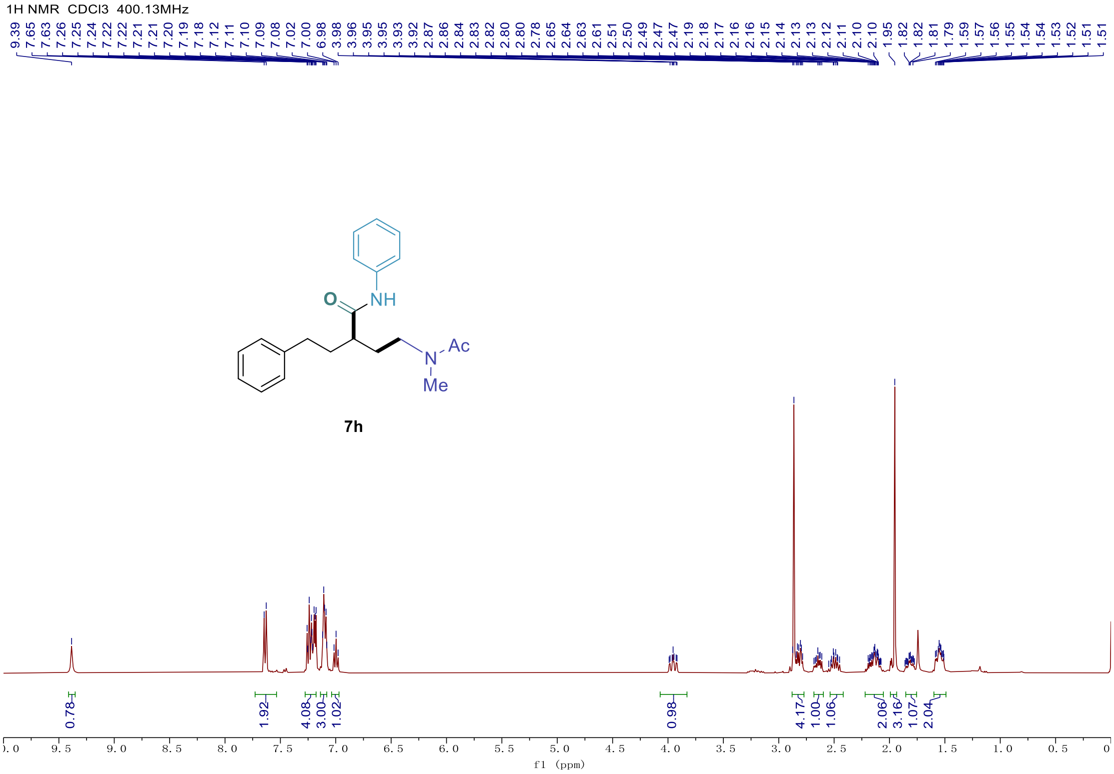

Supplementary Figure 145. ^{1}H NMR of compound **7h** (400 MHz, $CDCl_3$)

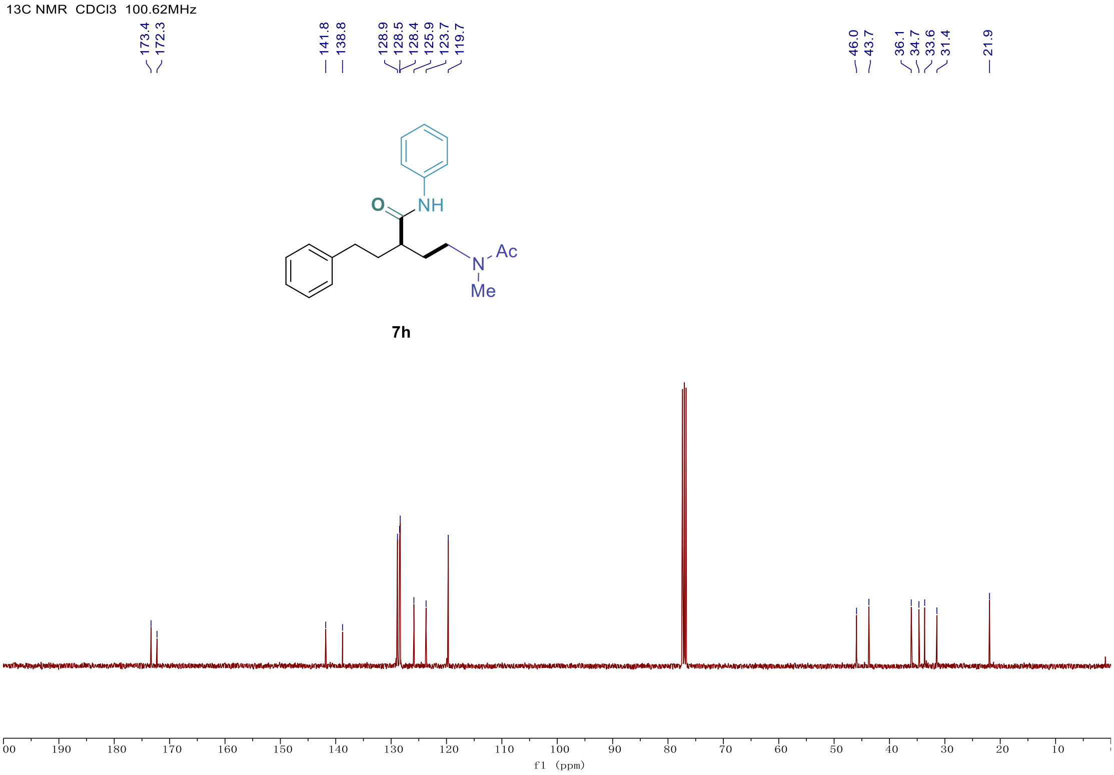

Supplementary Figure 146. ^{13}C NMR of compound **7h** (100 MHz, $CDCl_3$)

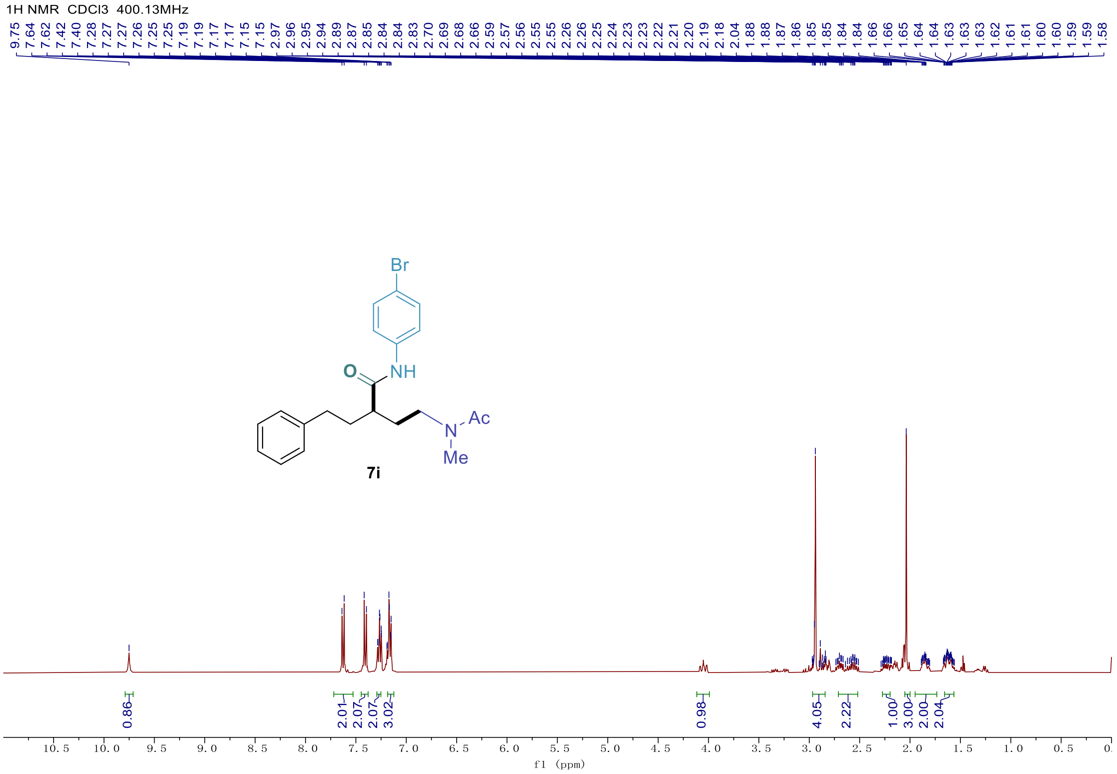

Supplementary Figure 147. ^{1}H NMR of compound **7i** (400 MHz, $CDCl_3$)

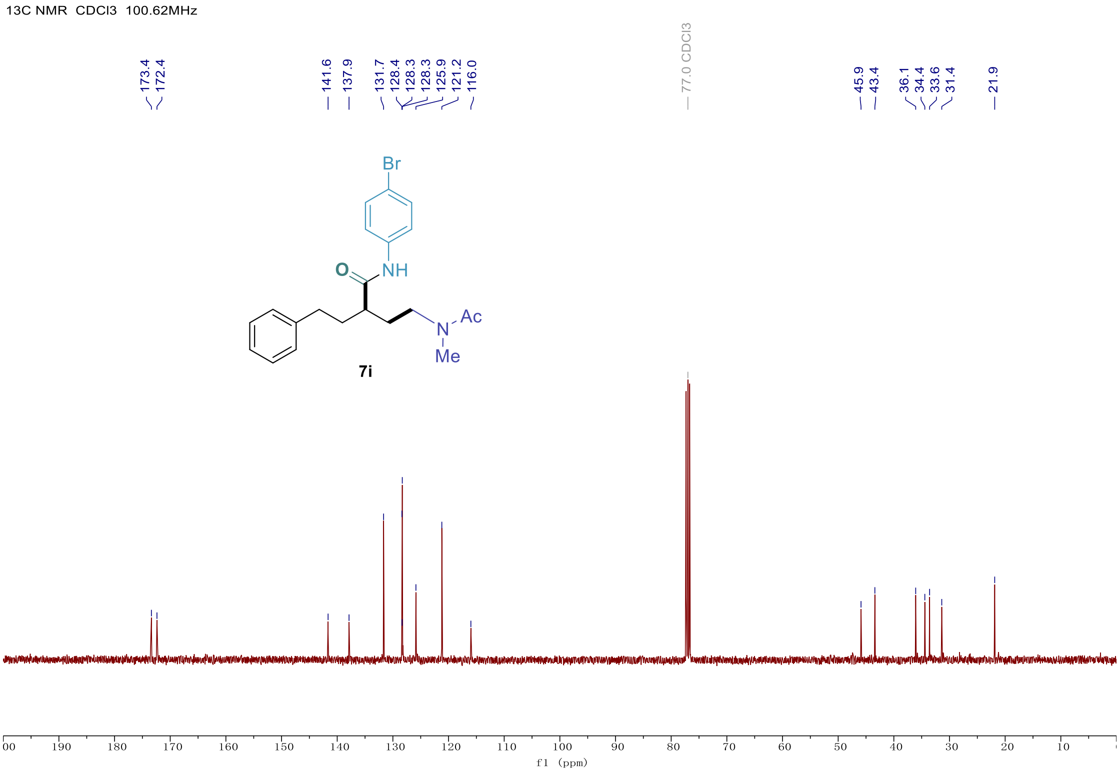

Supplementary Figure 148. ^{13}C NMR of compound **7i** (100 MHz, $CDCl_3$)

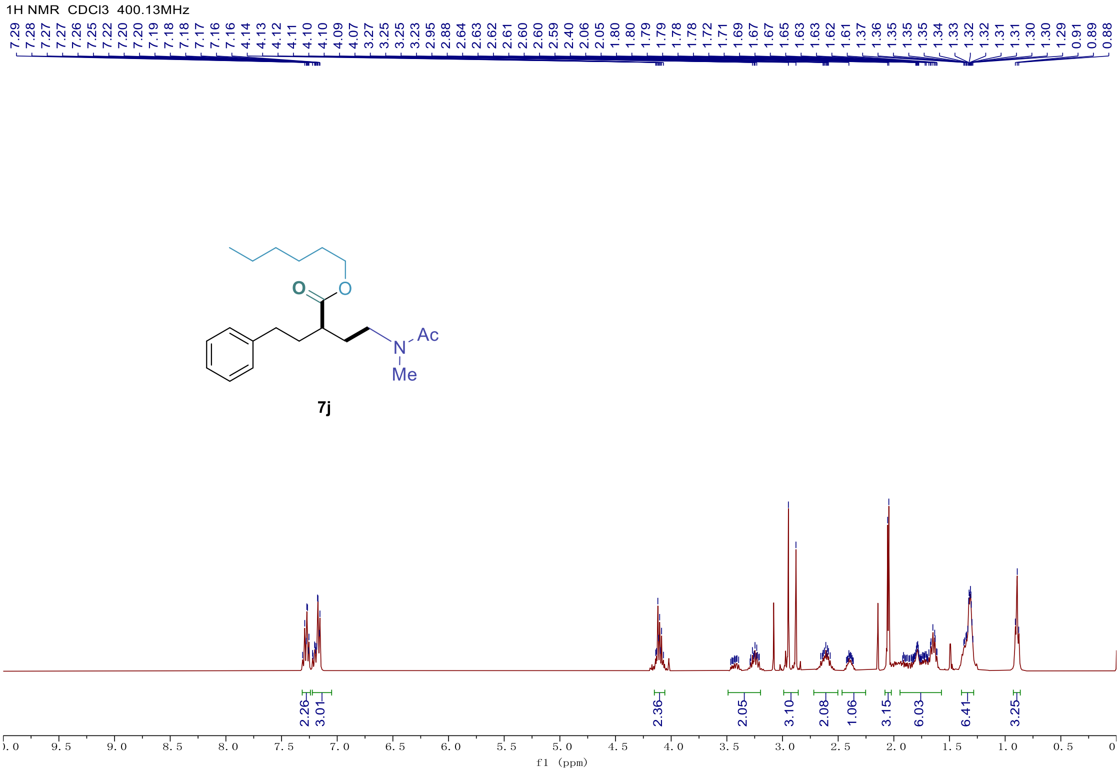

Supplementary Figure 149. ^{1}H NMR of compound **7j** (400 MHz, $CDCl_3$)

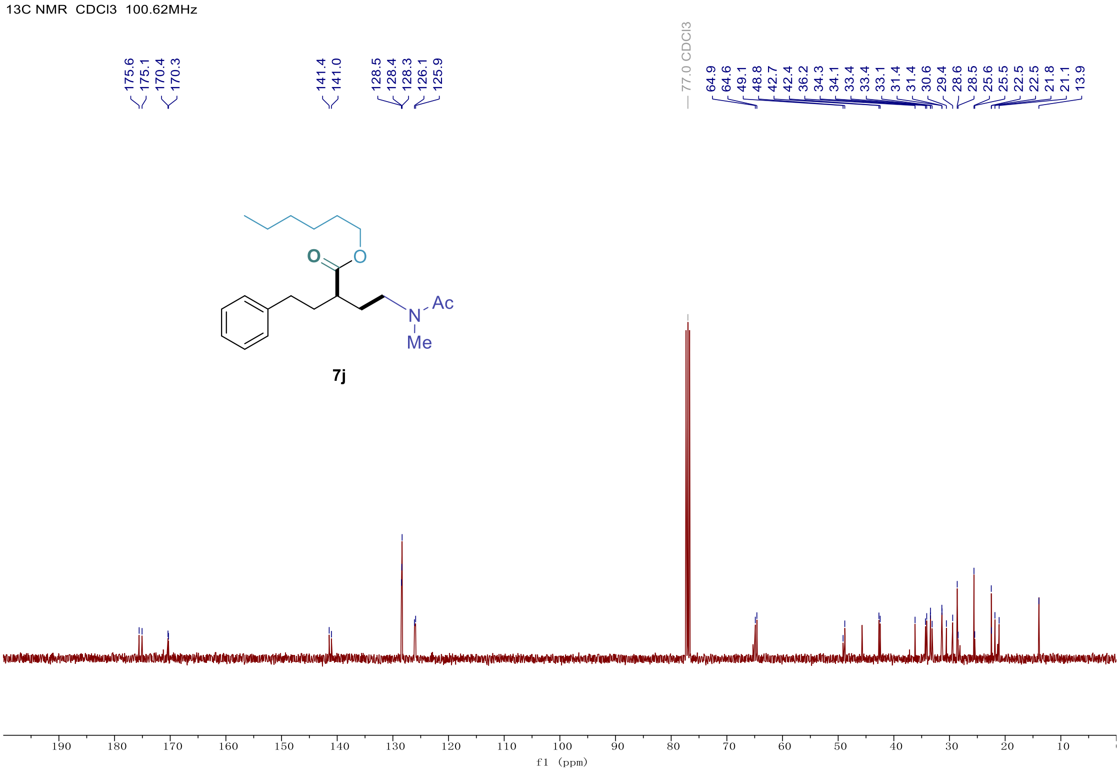

Supplementary Figure 150. ^{13}C NMR of compound **7j** (100 MHz, $CDCl_3$)

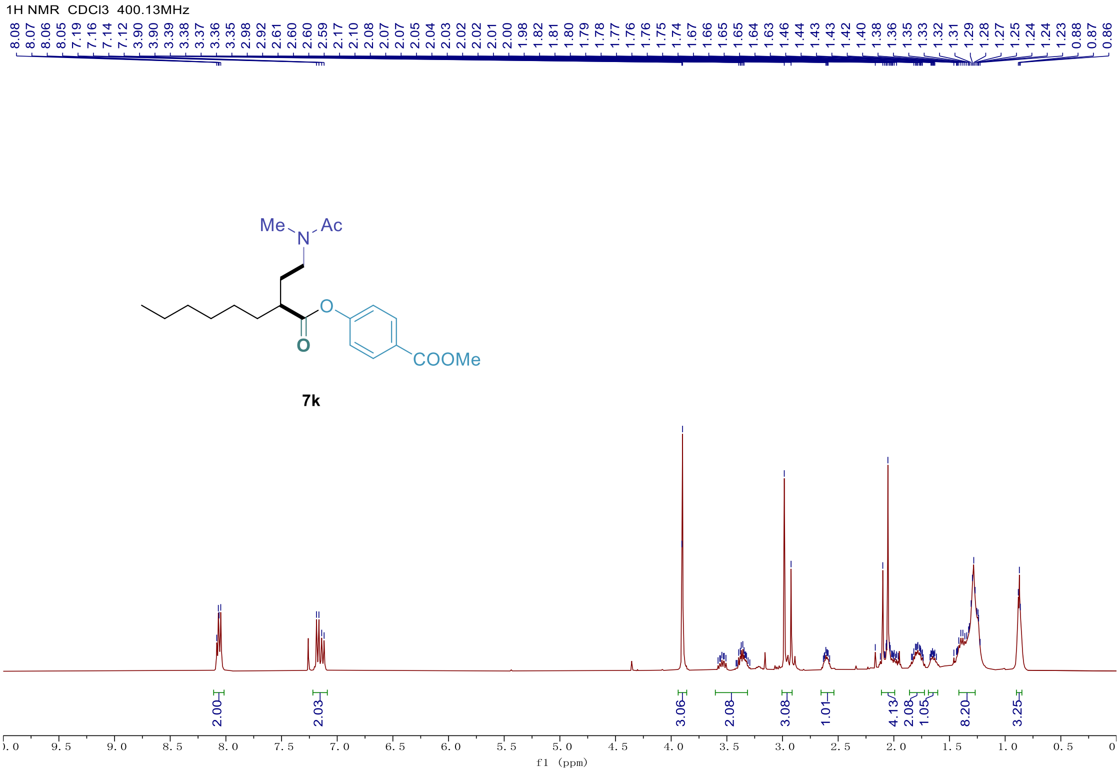

Supplementary Figure 151. ^{1}H NMR of compound **7k** (400 MHz, $CDCl_3$)

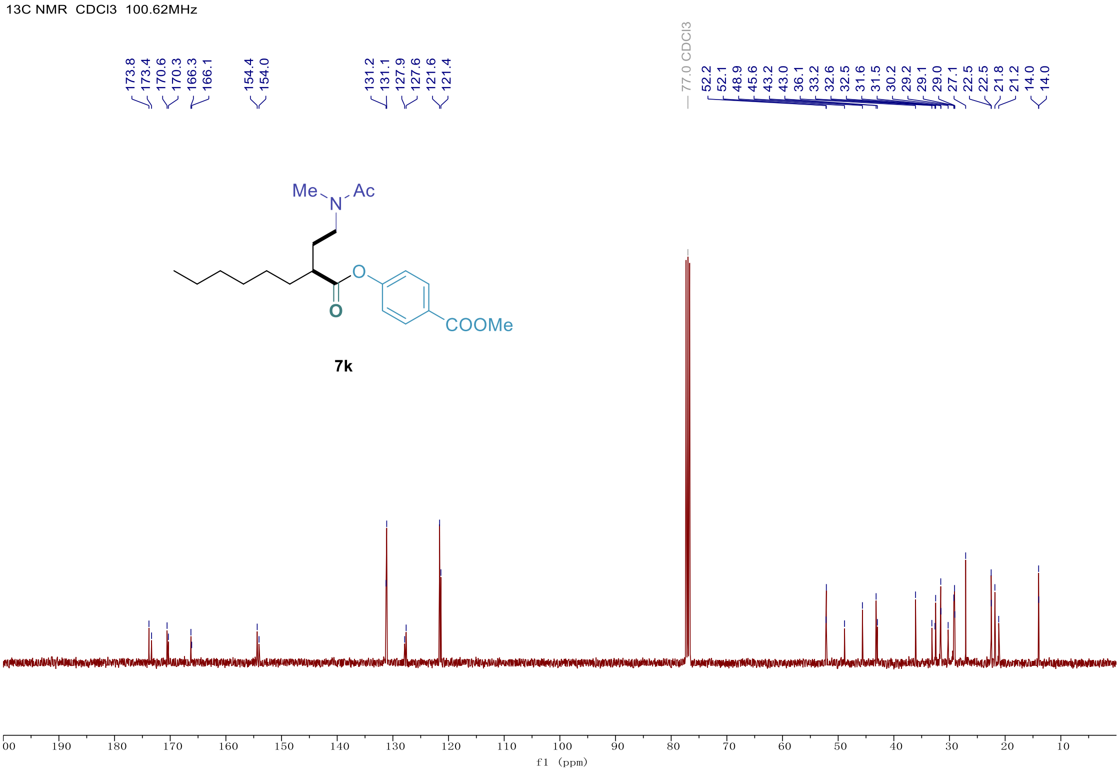

Supplementary Figure 152. ^{13}C NMR of compound **7k** (100 MHz, $CDCl_3$)

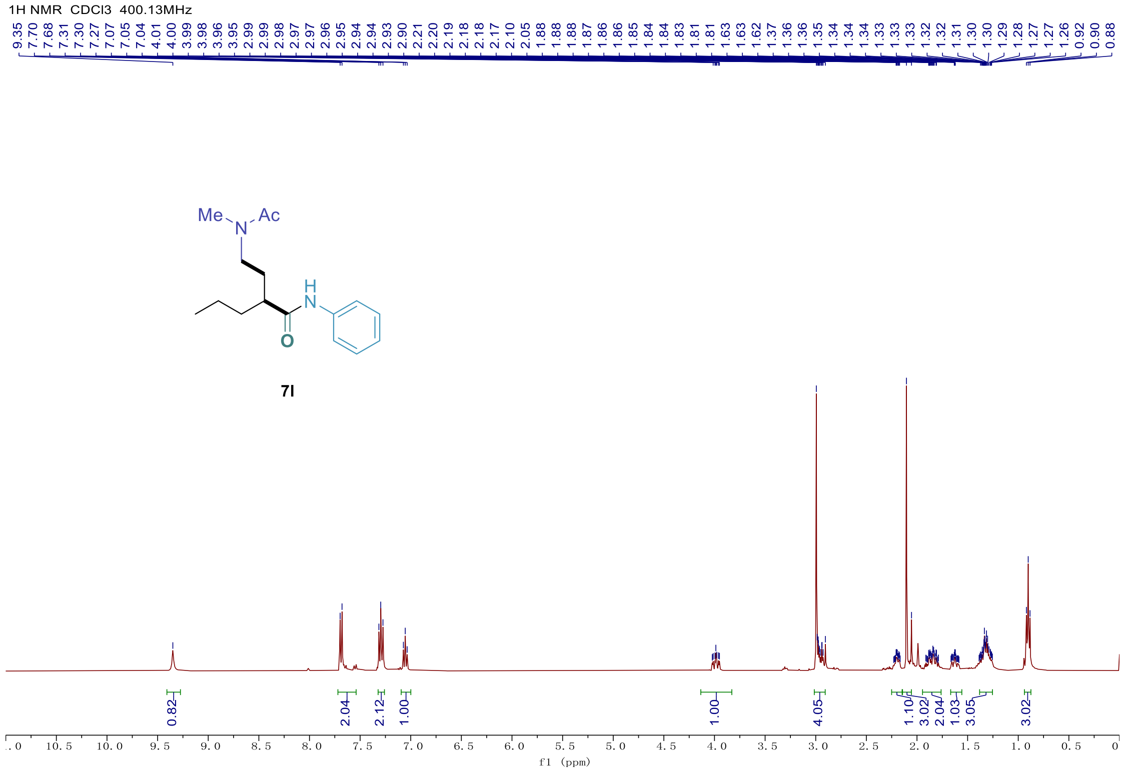

Supplementary Figure 153. ^{1}H NMR of compound **7l** (400 MHz, $CDCl_3$)

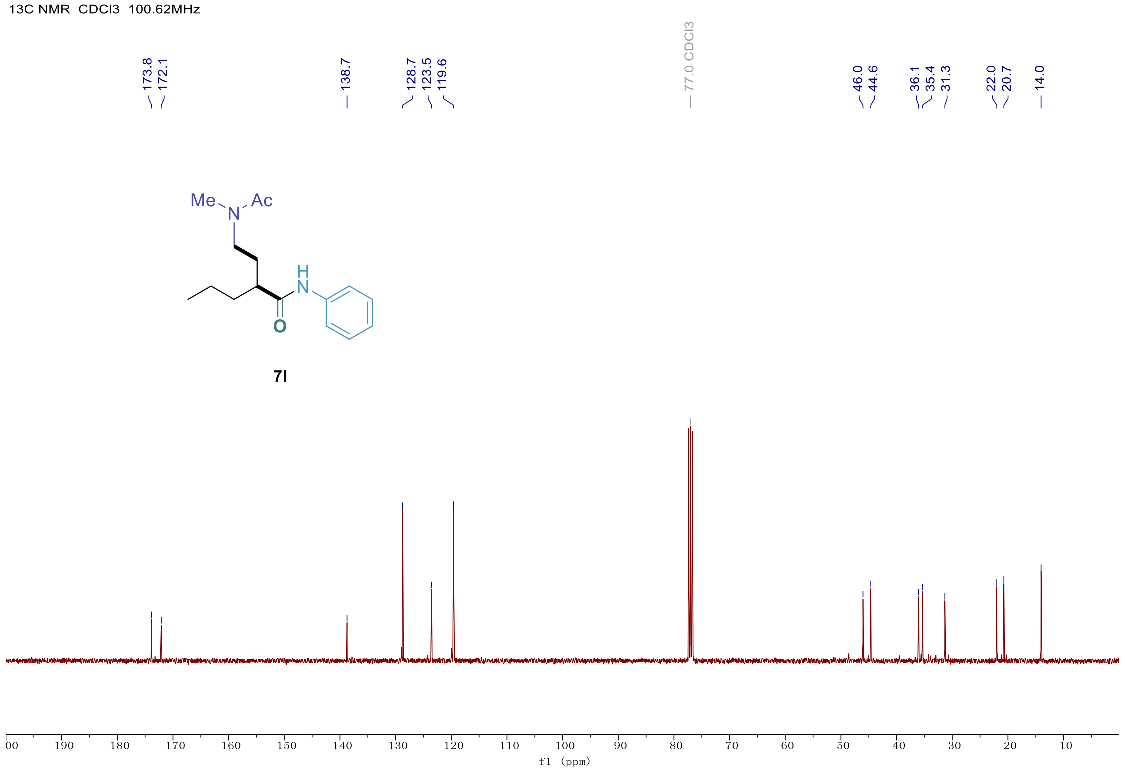

Supplementary Figure 154. ^{13}C NMR of compound **7l** (100 MHz, $CDCl_3$)

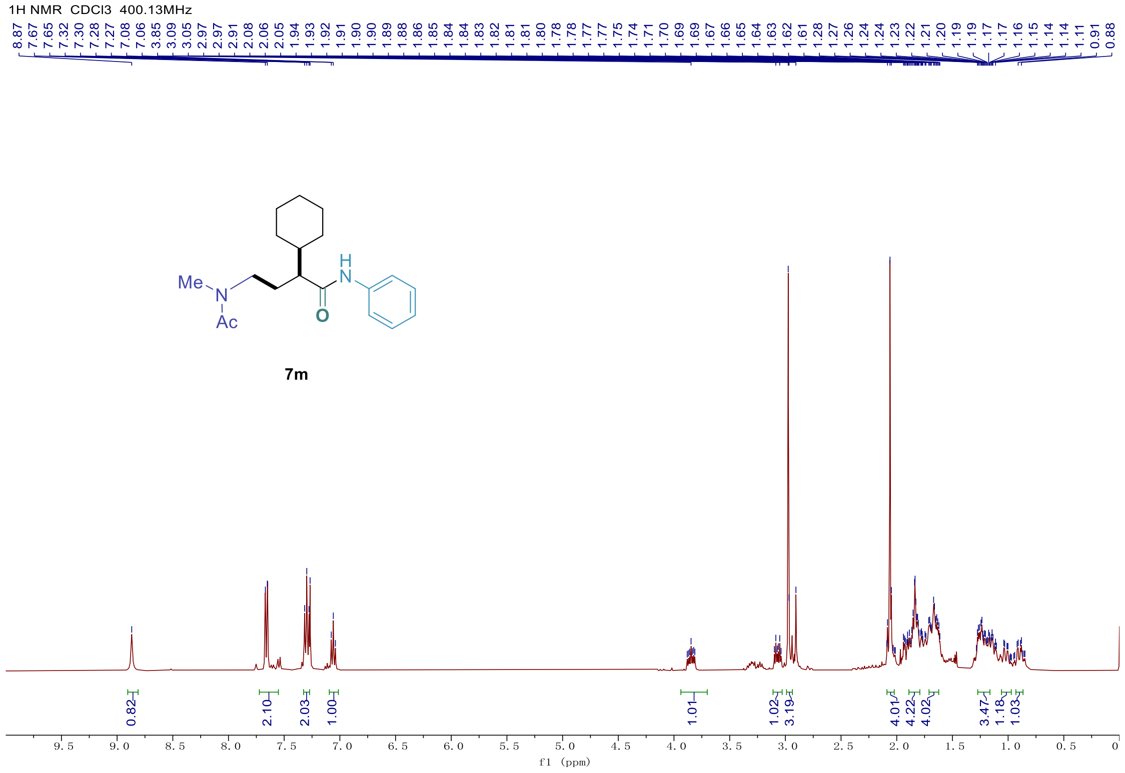

Supplementary Figure 155. ^{1}H NMR of compound **7m** (400 MHz, $CDCl_3$)

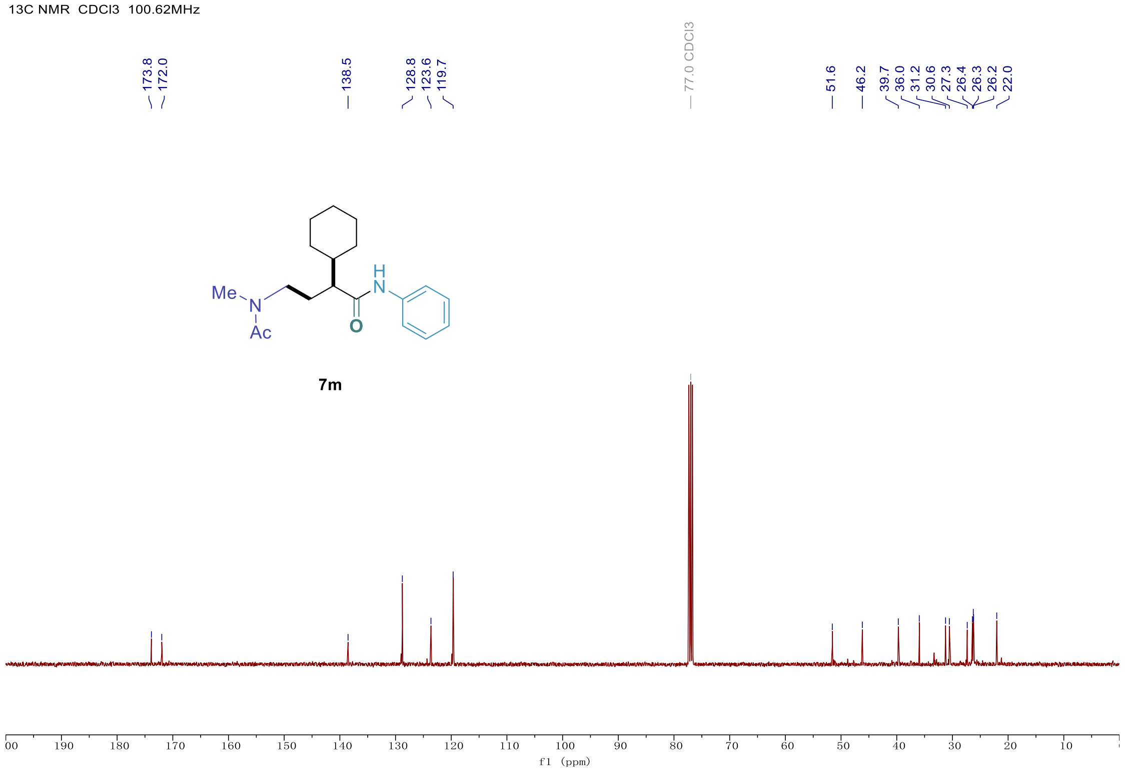

Supplementary Figure 156. ^{13}C NMR of compound **7m** (100 MHz, $CDCl_3$)

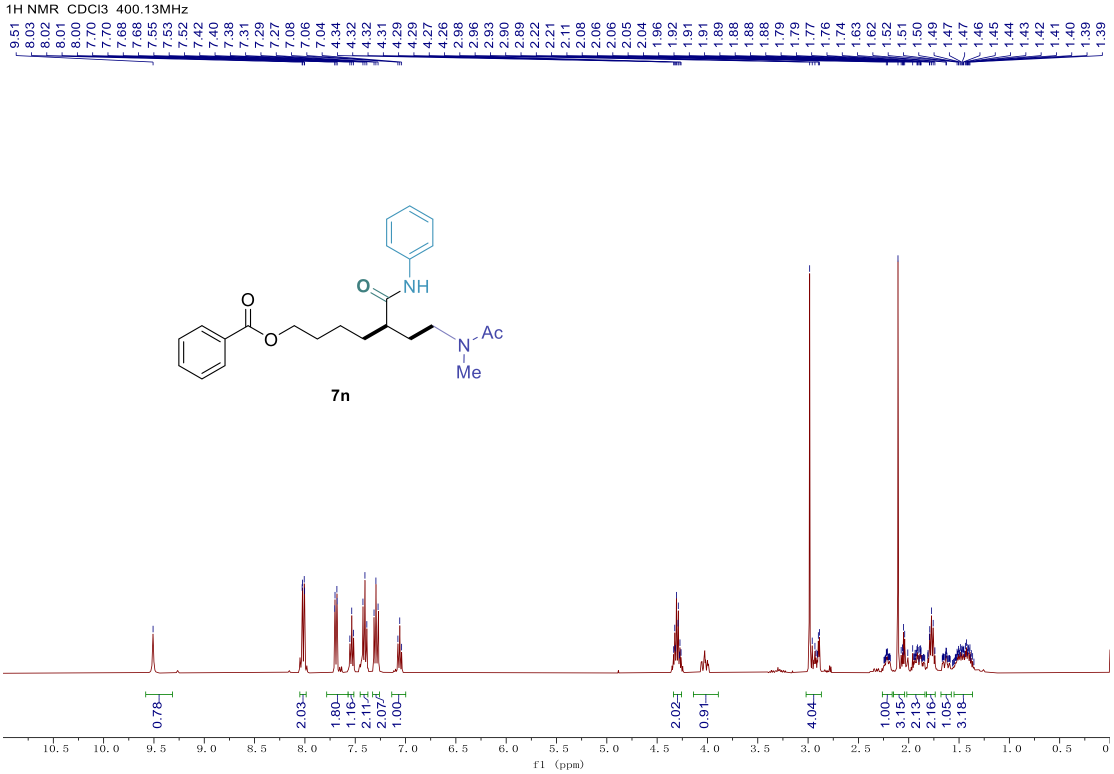

Supplementary Figure 157. ^{1}H NMR of compound **7n** (400 MHz, $CDCl_3$)

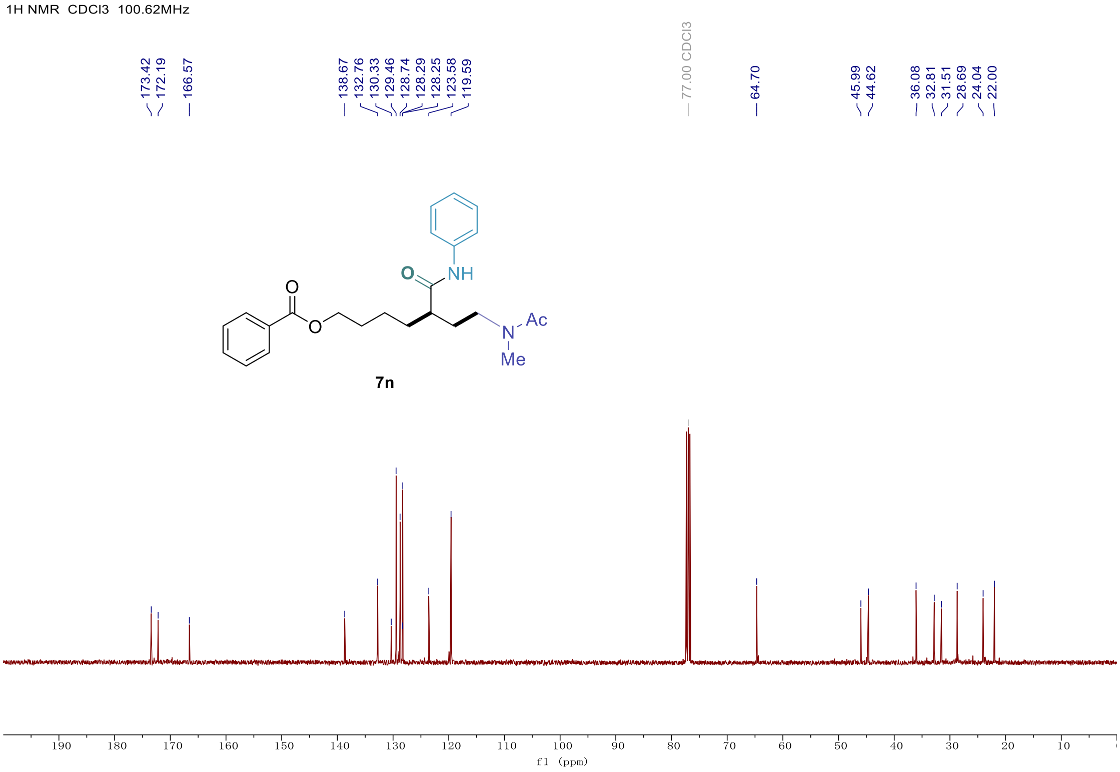

Supplementary Figure 158. ^{13}C NMR of compound **7n** (100 MHz, $CDCl_3$)

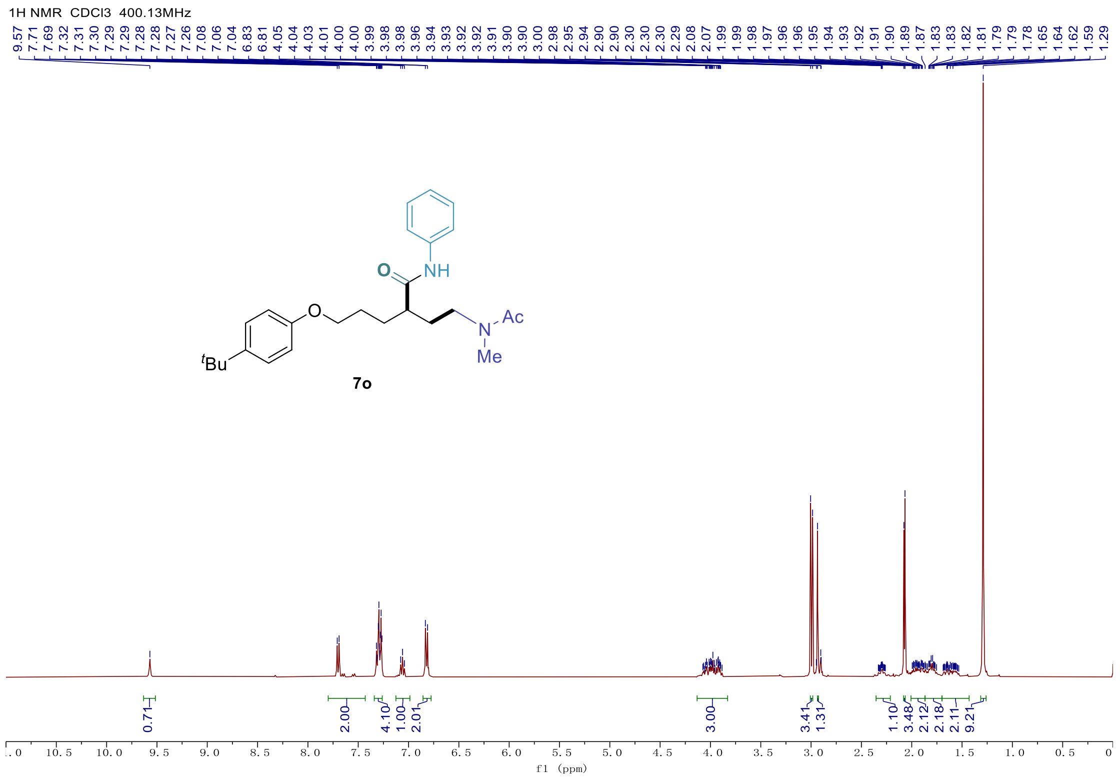

Supplementary Figure 159. ^{1}H NMR of compound **7o** (400 MHz, $CDCl_3$)

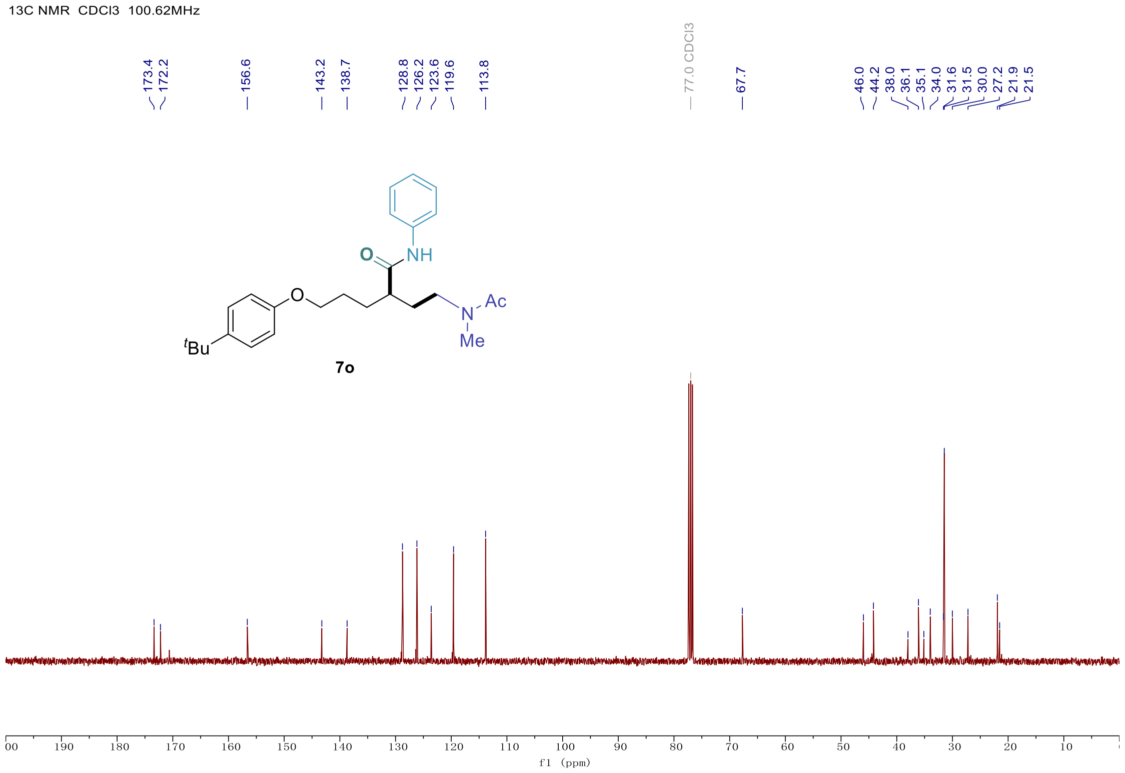

Supplementary Figure 160. ^{13}C NMR of compound **7o** (100 MHz, $CDCl_3$)

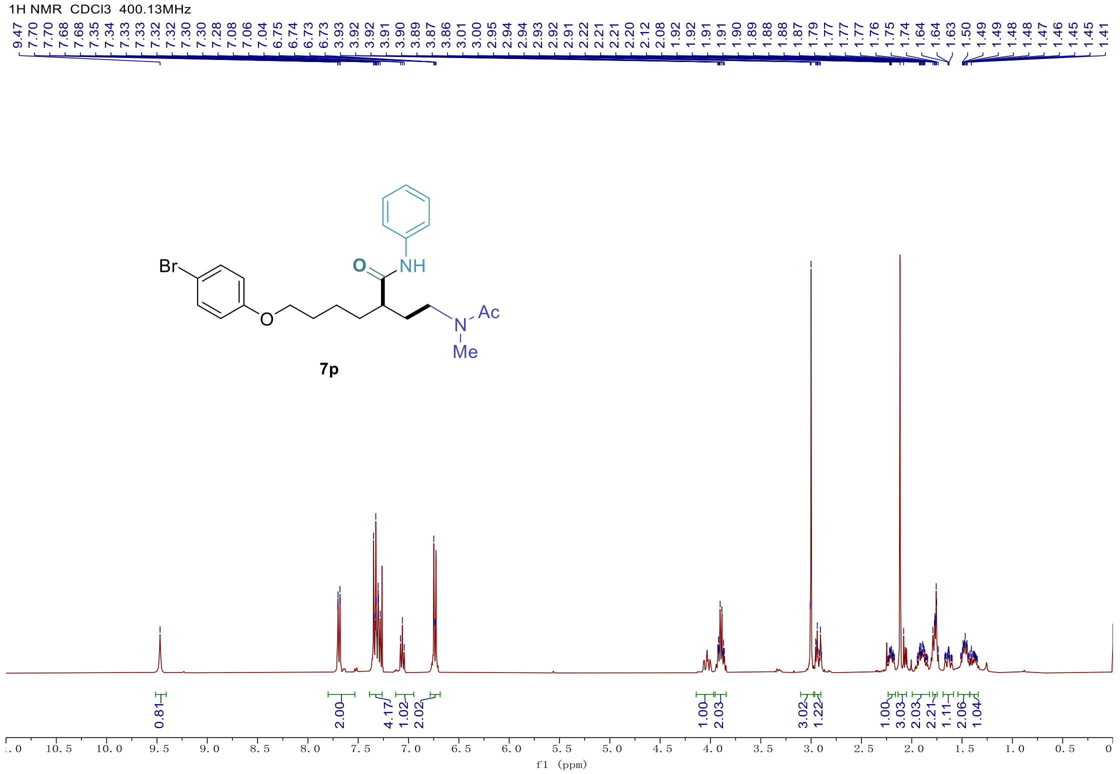

Supplementary Figure 161. ^{1}H NMR of compound **7p** (400 MHz, $CDCl_3$)

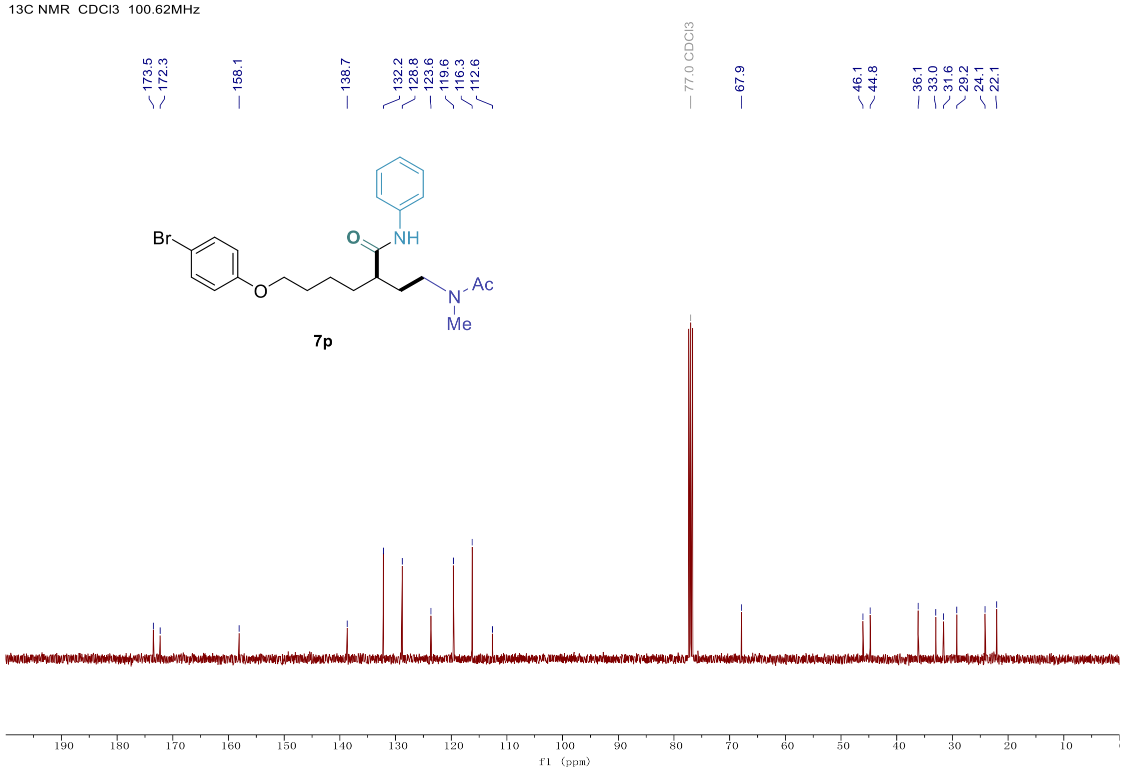

Supplementary Figure 162. ^{13}C NMR of compound **7p** (100 MHz, $CDCl_3$)

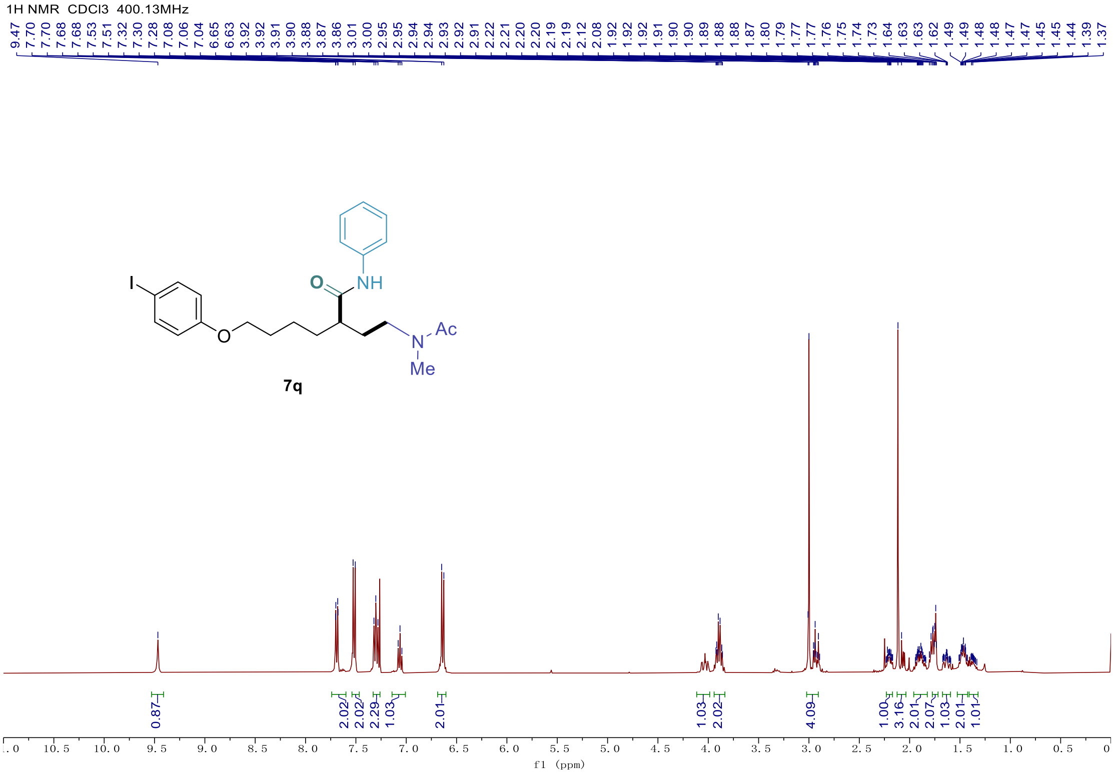

Supplementary Figure 163. ^{1}H NMR of compound **7q** (400 MHz, $CDCl_3$)

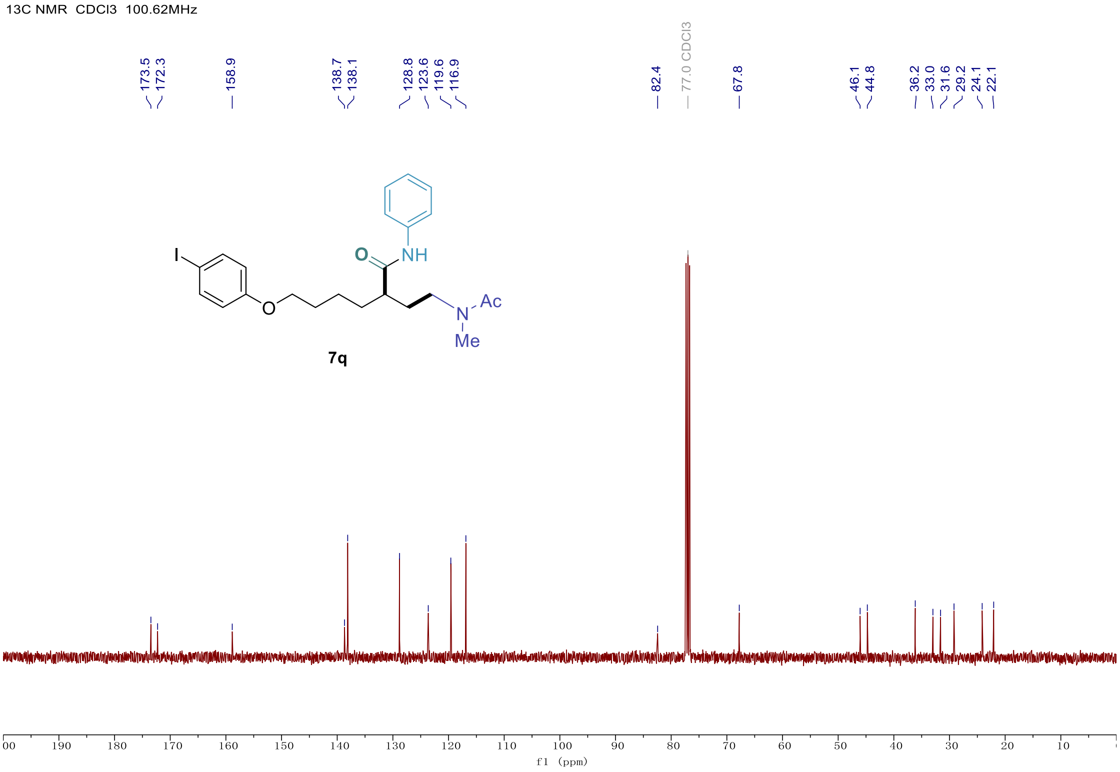

Supplementary Figure 164. ^{13}C NMR of compound **7q** (100 MHz, $CDCl_3$)

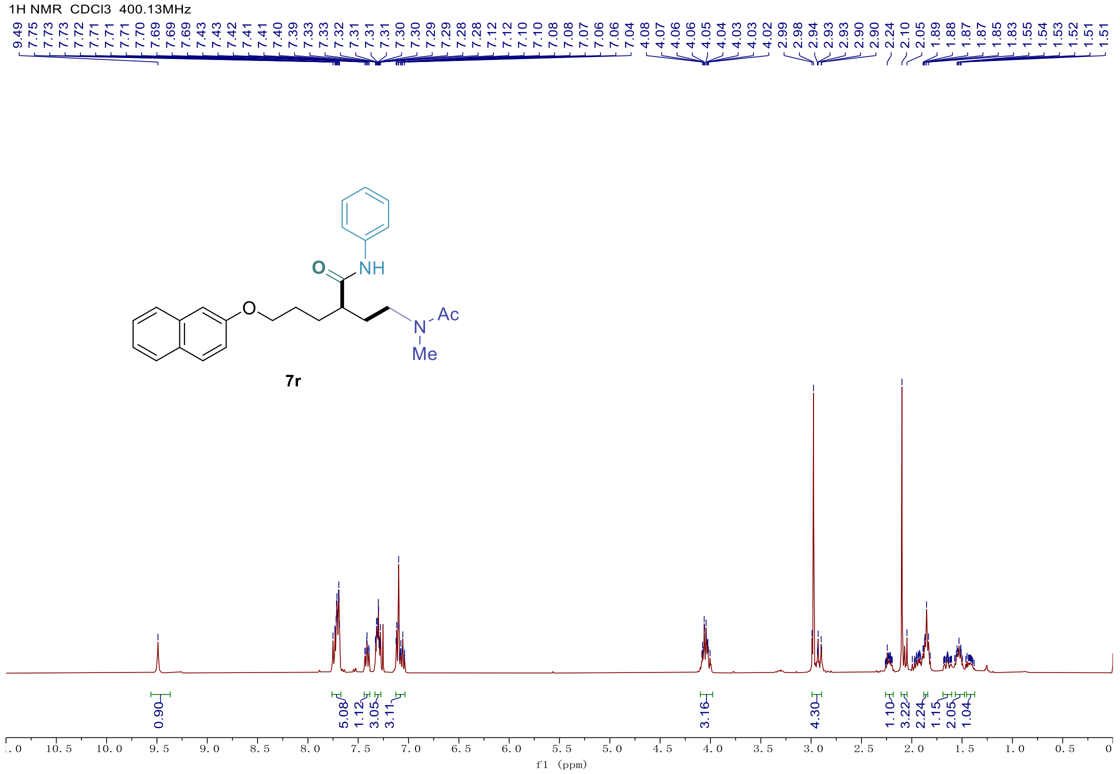

Supplementary Figure 165. ^{1}H NMR of compound **7r** (400 MHz, $CDCl_3$)

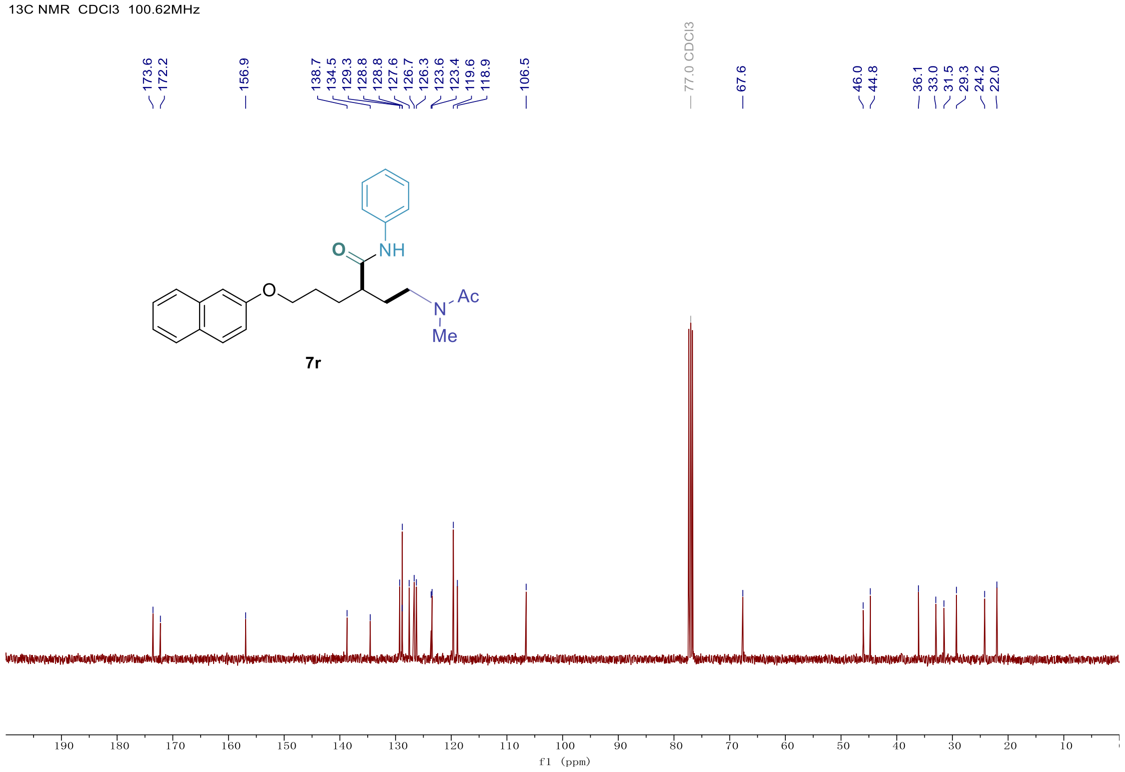

Supplementary Figure 166. ^{13}C NMR of compound **7r** (100 MHz, $CDCl_3$)

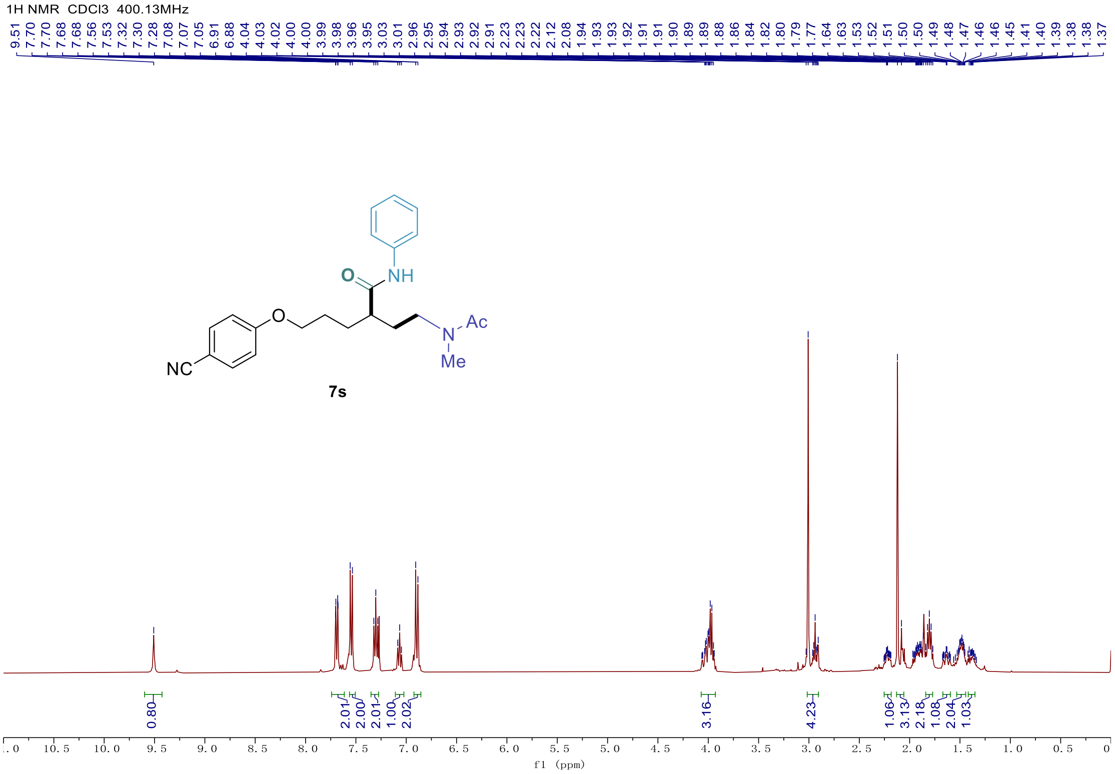

Supplementary Figure 167. ^{1}H NMR of compound **7s** (400 MHz, $CDCl_3$)

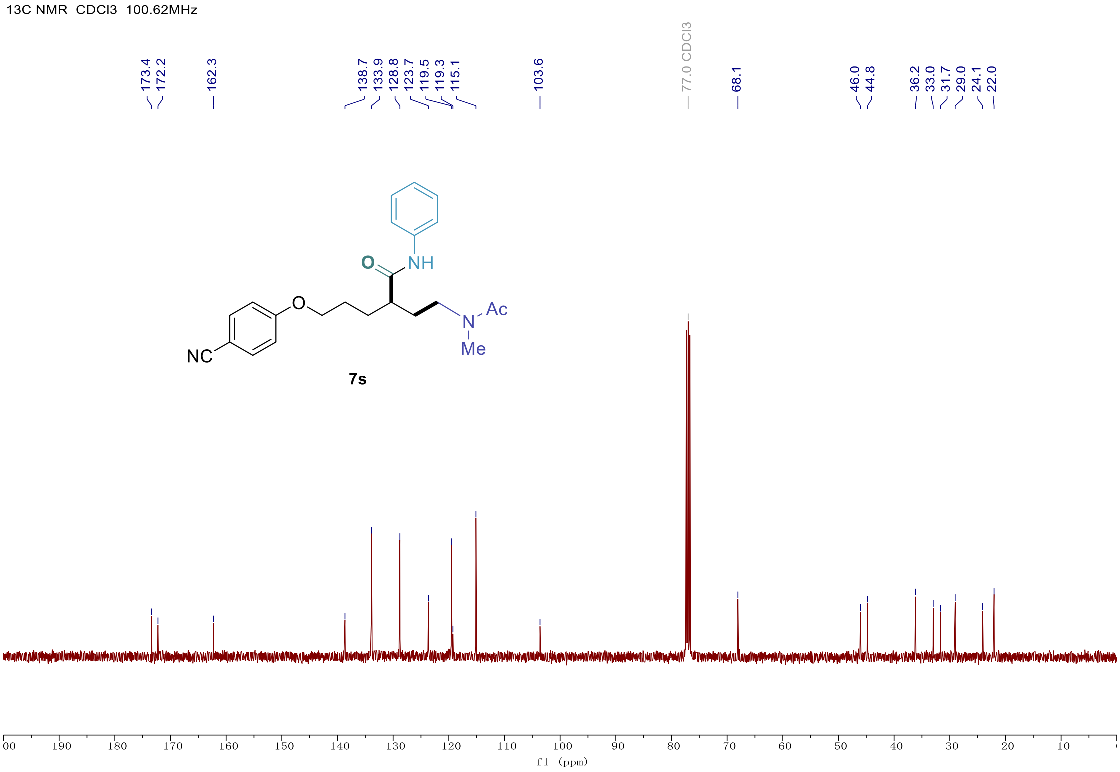

Supplementary Figure 168. ^{13}C NMR of compound **7s** (100 MHz, $CDCl_3$)

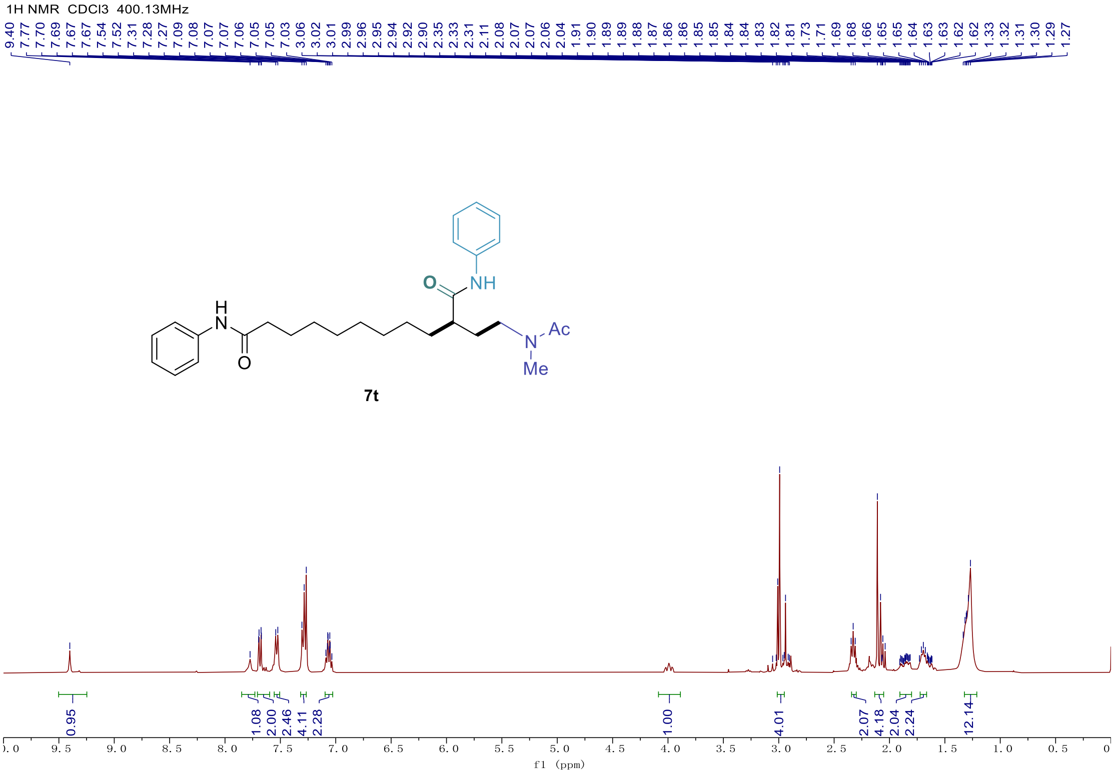

Supplementary Figure 169. ^{1}H NMR of compound **7t** (400 MHz, $CDCl_3$)

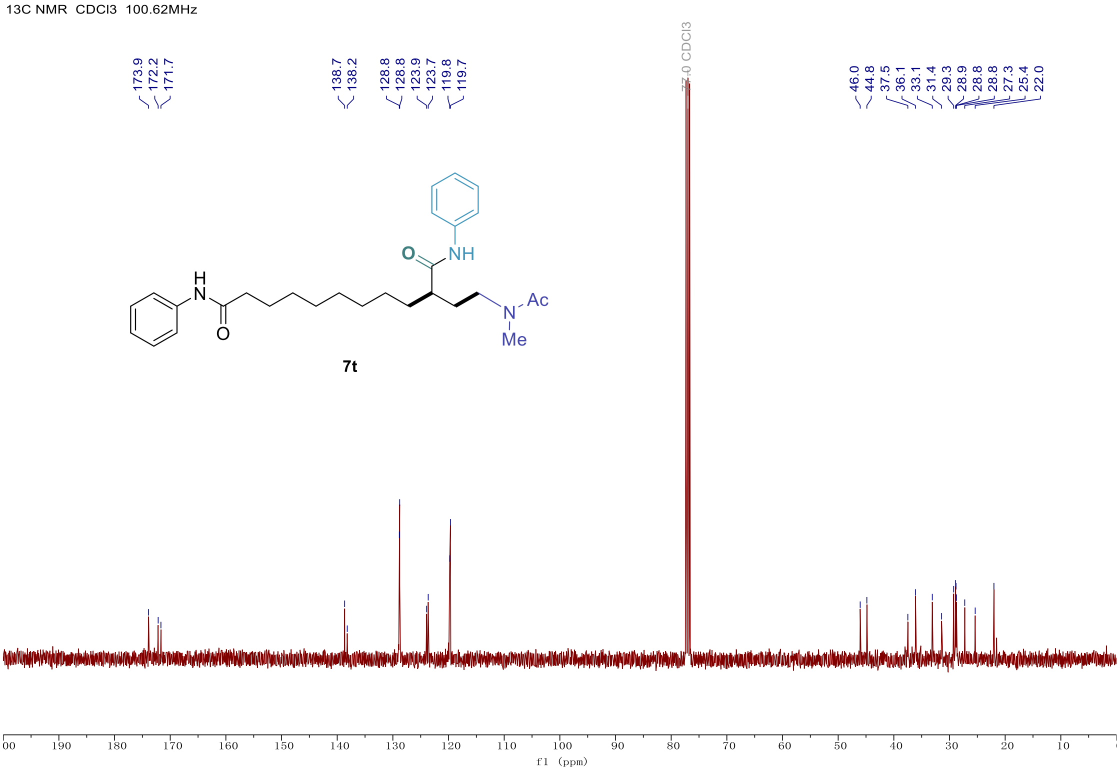

Supplementary Figure 170. ^{13}C NMR of compound **7t** (100 MHz, $CDCl_3$)

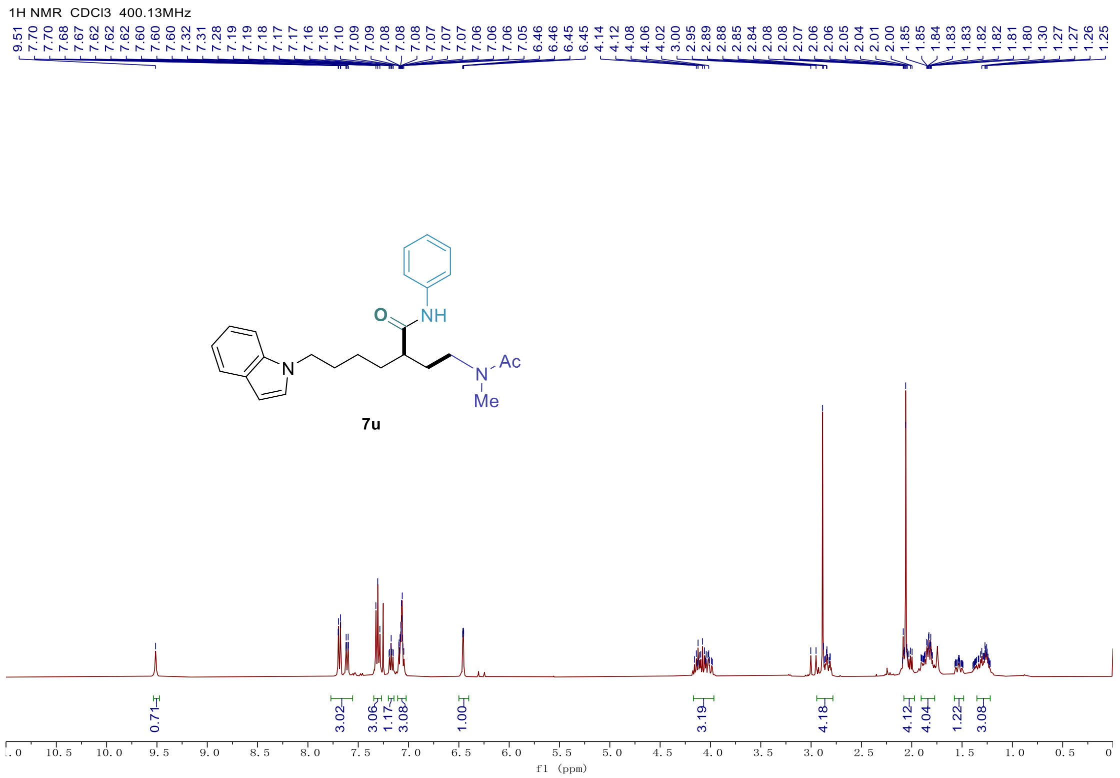

Supplementary Figure 171. ^{1}H NMR of compound **7u** (400 MHz, $CDCl_3$)

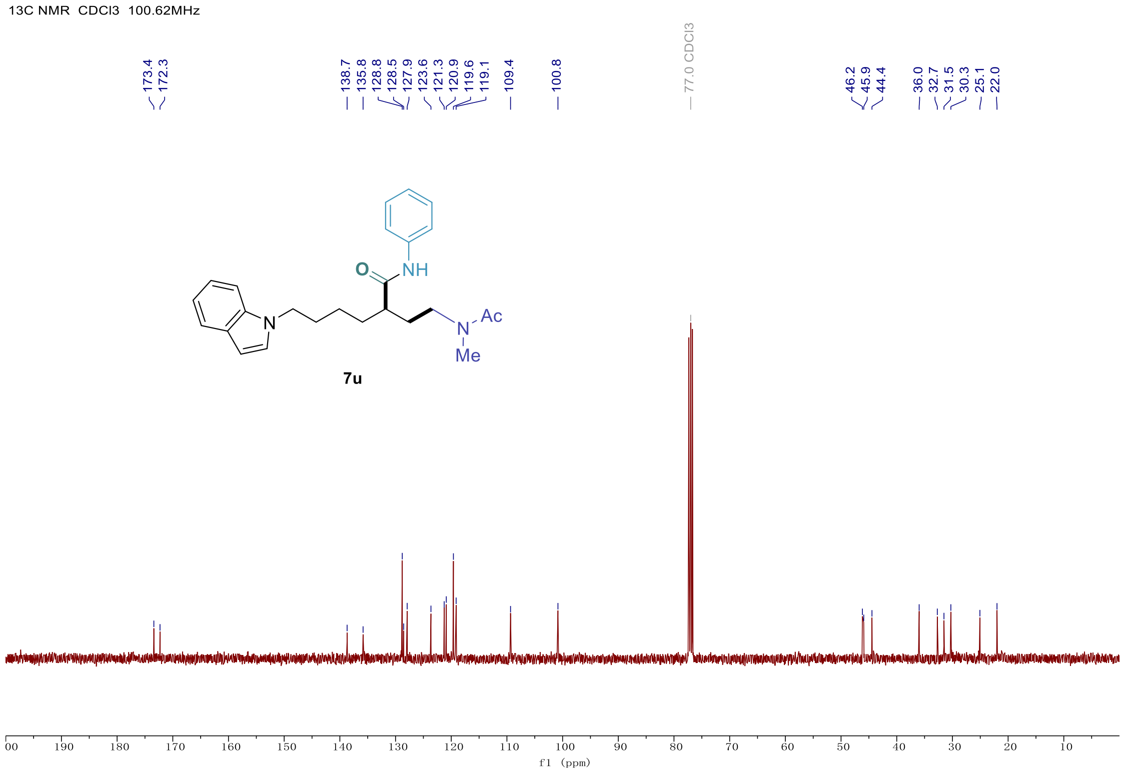

Supplementary Figure 172. ^{13}C NMR of compound **7u** (100 MHz, $CDCl_3$)

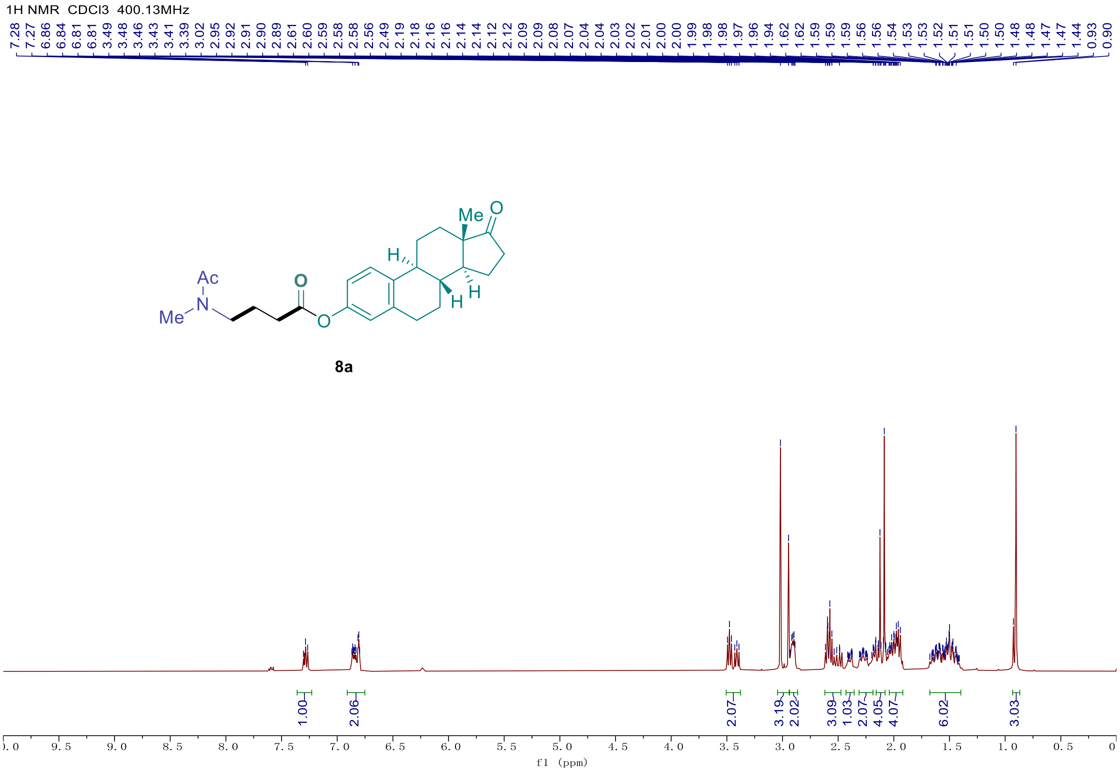

Supplementary Figure 173. ^{1}H NMR of compound **8a** (400 MHz, $CDCl_3$)

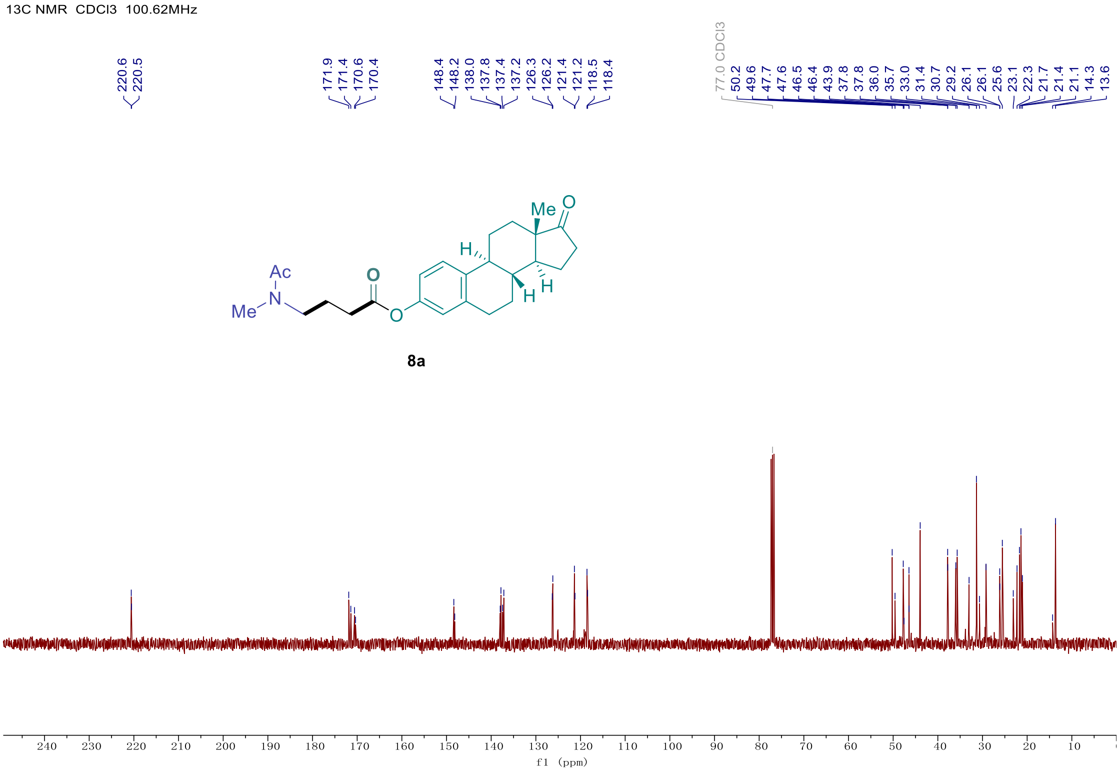

Supplementary Figure 174. ^{13}C NMR of compound **8a** (100 MHz, $CDCl_3$)

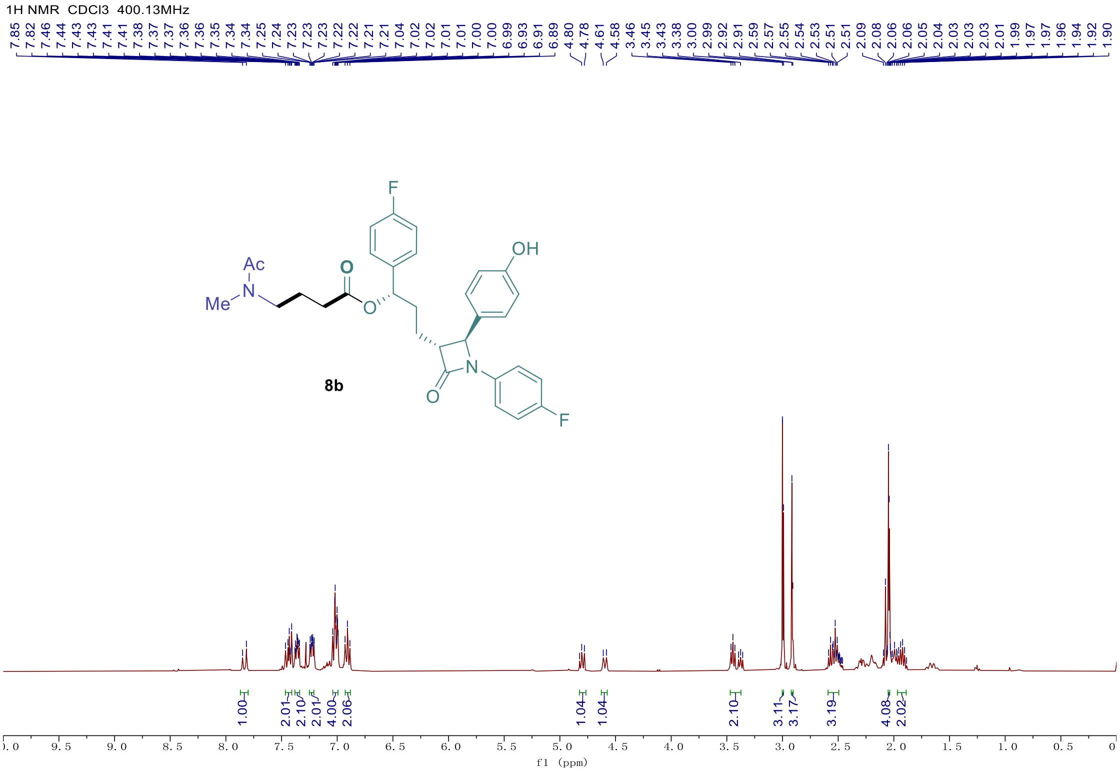

Supplementary Figure 175. ^{1}H NMR of compound **8b** (400 MHz, $CDCl_3$)

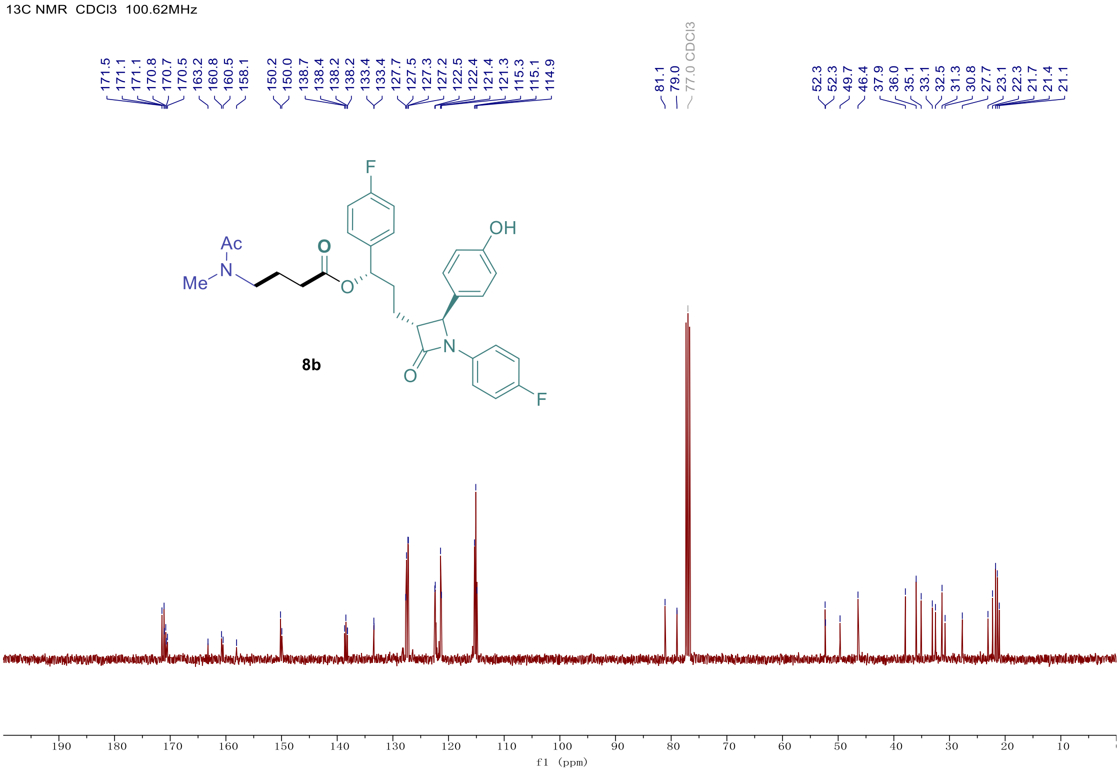

Supplementary Figure 176. ^{13}C NMR of compound **8b** (100 MHz, $CDCl_3$)

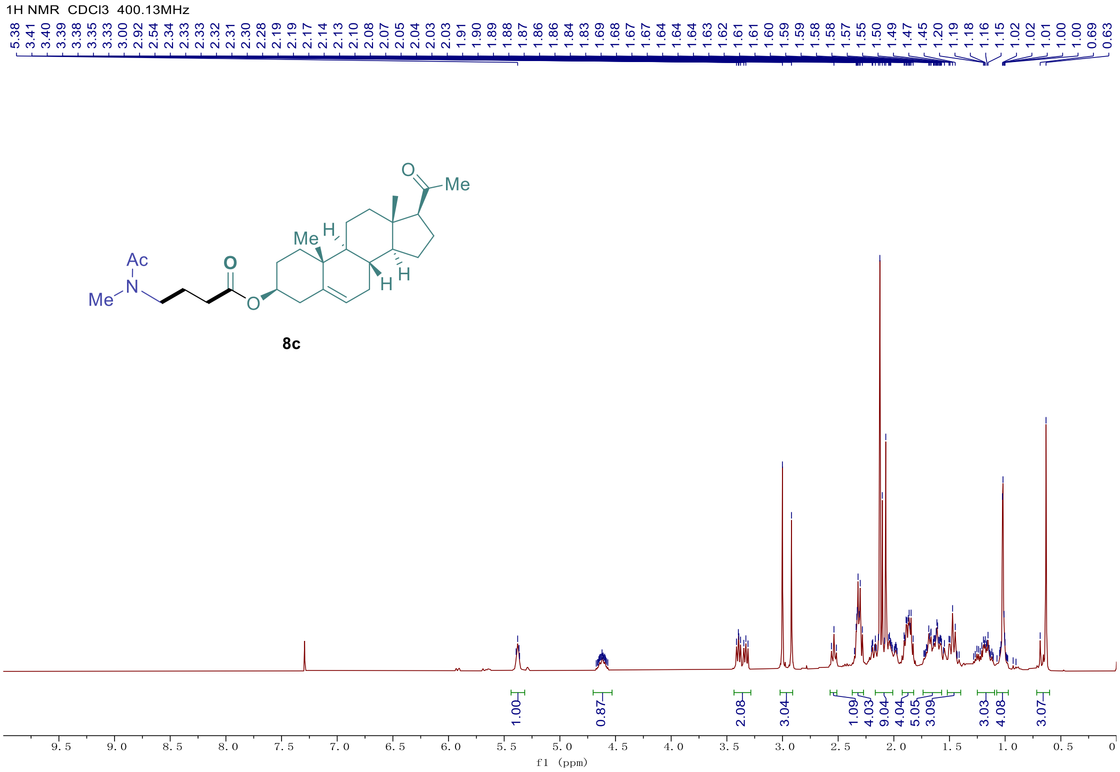

Supplementary Figure 177. ^{1}H NMR of compound **8c** (400 MHz, $CDCl_3$)

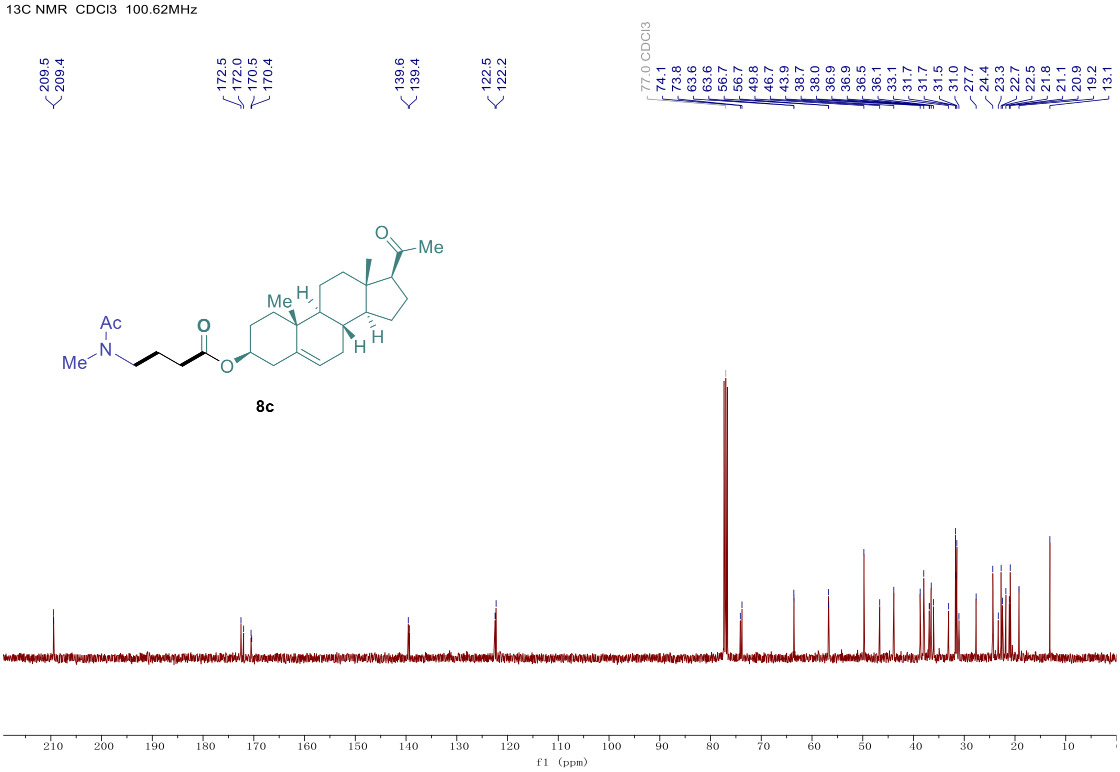

Supplementary Figure 178. ^{13}C NMR of compound **8c** (100 MHz, $CDCl_3$)

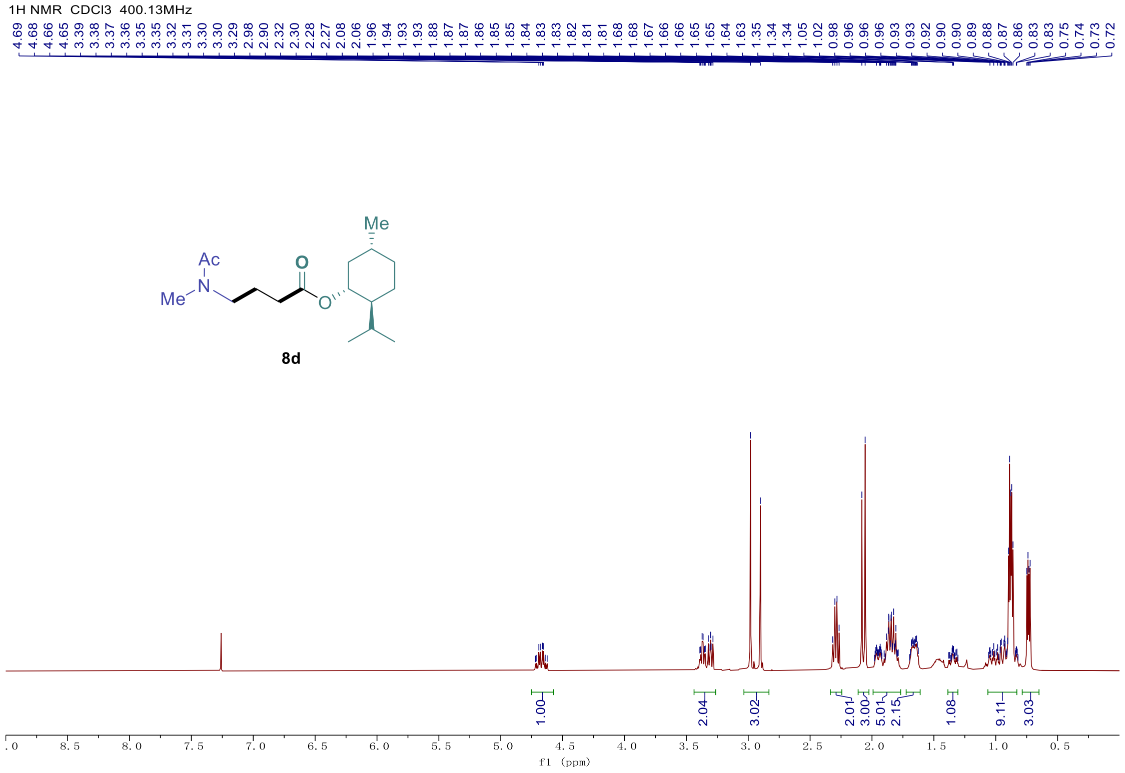

Supplementary Figure 179. ^{1}H NMR of compound **8d** (400 MHz, $CDCl_3$)

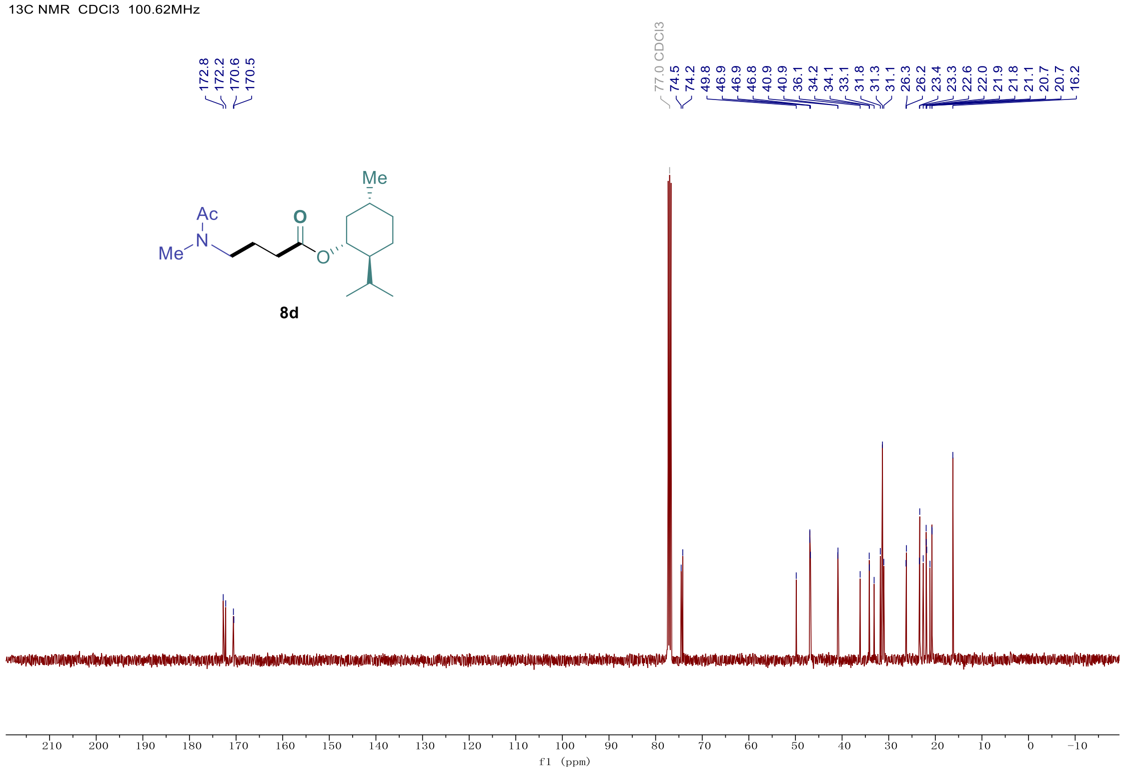

Supplementary Figure 180. ^{13}C NMR of compound **8d** (100 MHz, $CDCl_3$)

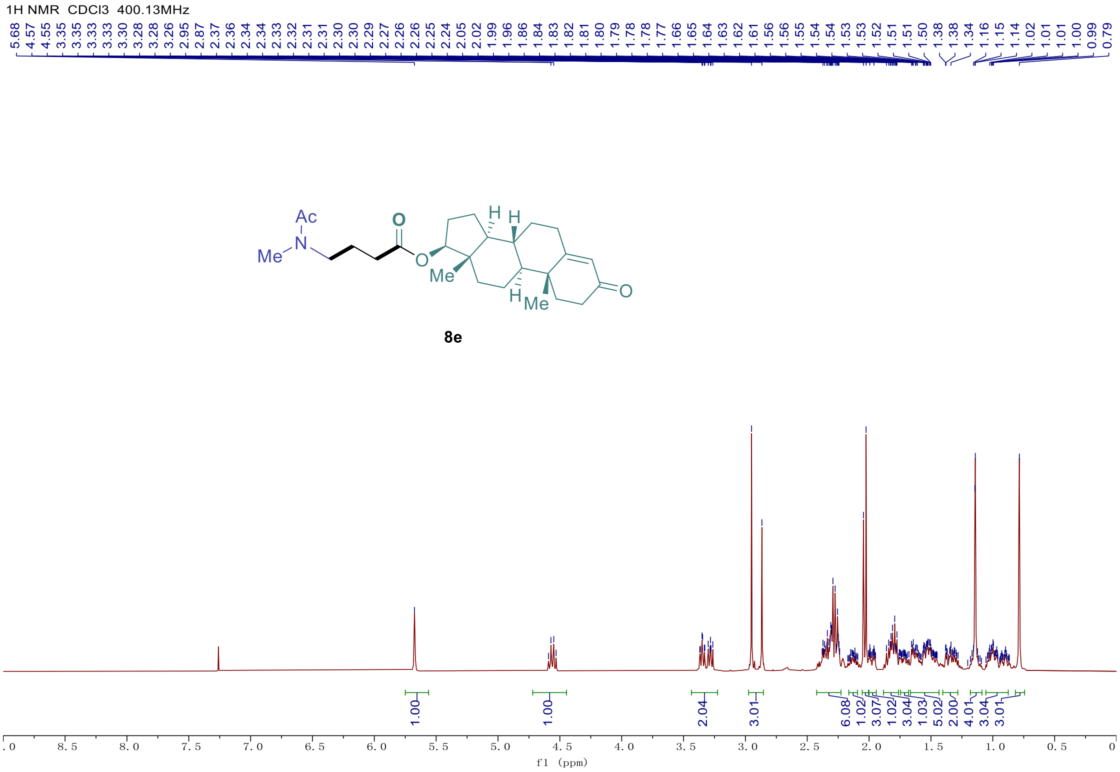

Supplementary Figure 181. ^{1}H NMR of compound **8e** (400 MHz, $CDCl_3$)

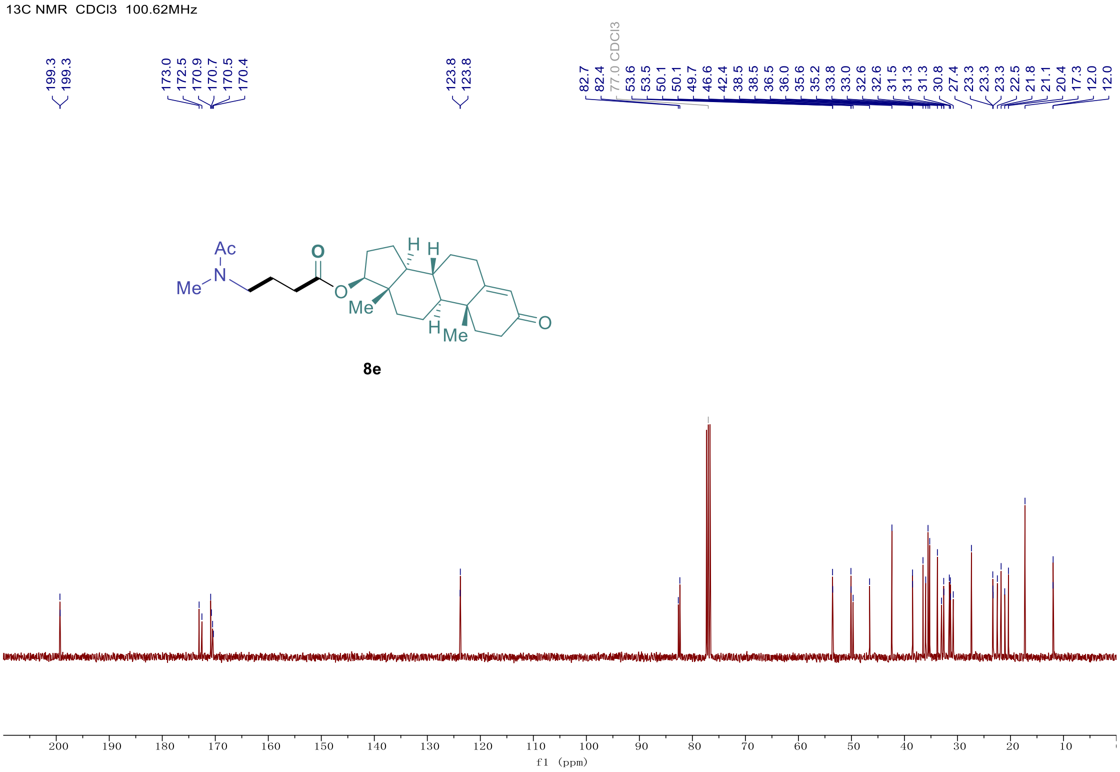

Supplementary Figure 182. ^{13}C NMR of compound **8e** (100 MHz, $CDCl_3$)

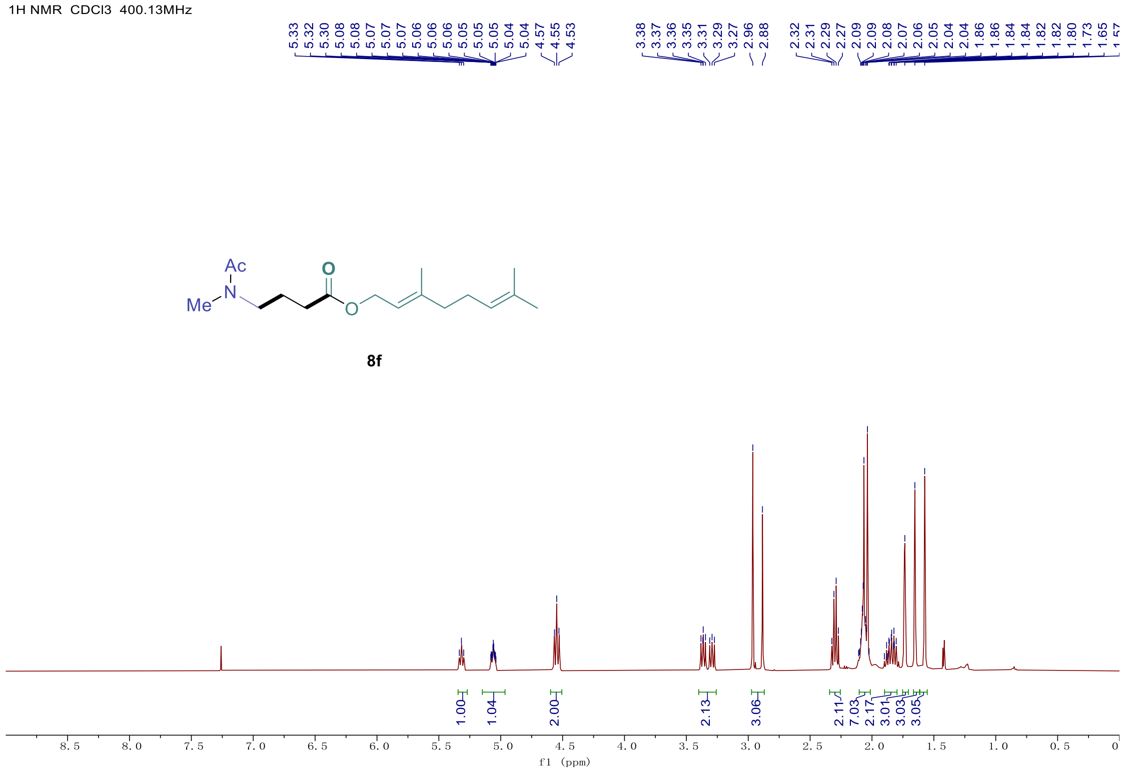

Supplementary Figure 183. ^{1}H NMR of compound **8f** (400 MHz, $CDCl_3$)

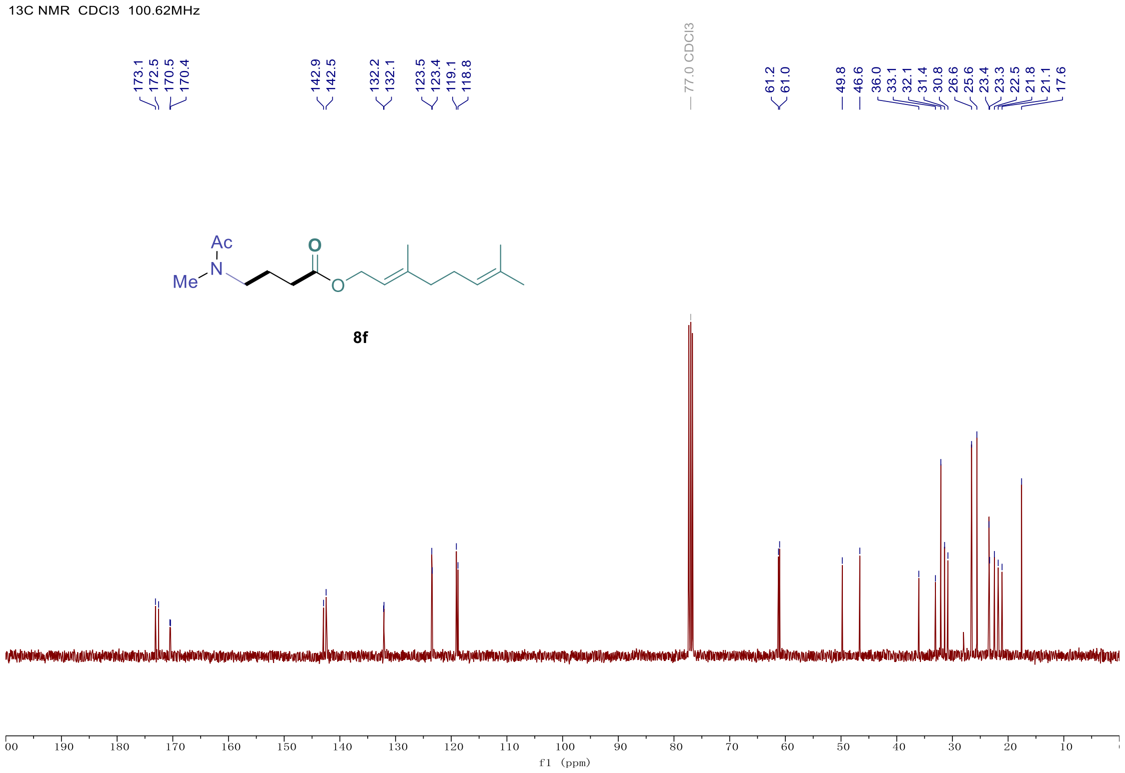

Supplementary Figure 184. ^{13}C NMR of compound **8f** (100 MHz, $CDCl_3$)

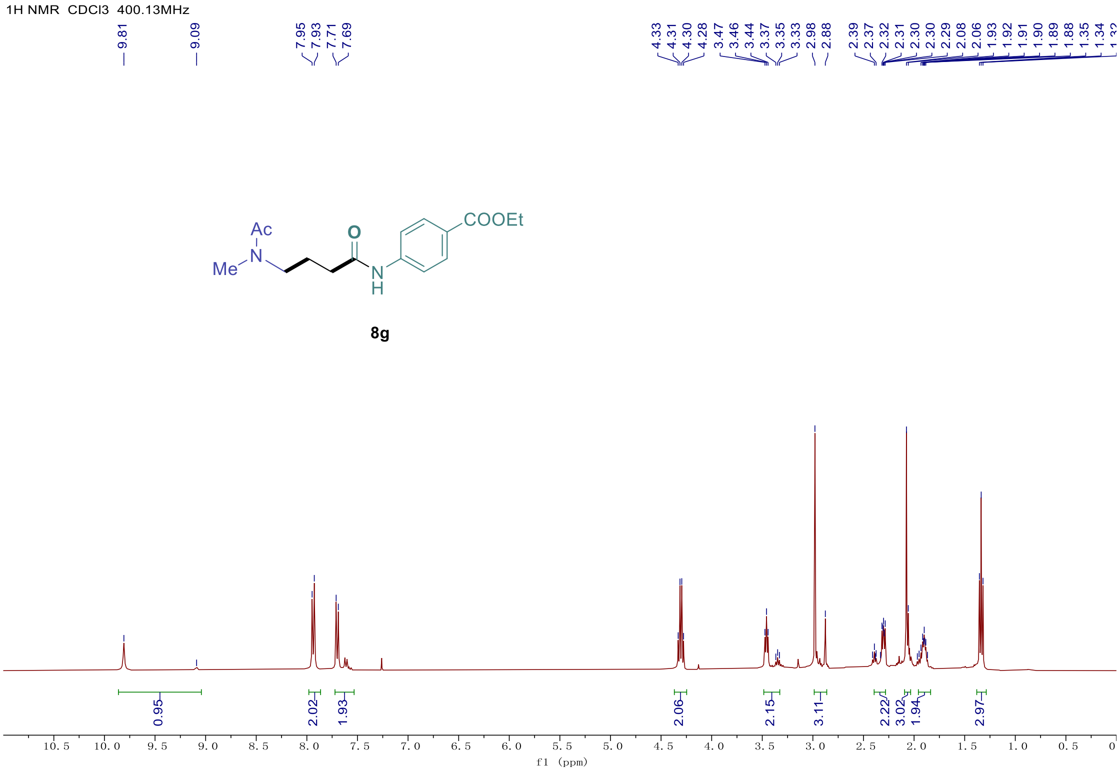

Supplementary Figure 185. ^{1}H NMR of compound **8g** (400 MHz, $CDCl_3$)

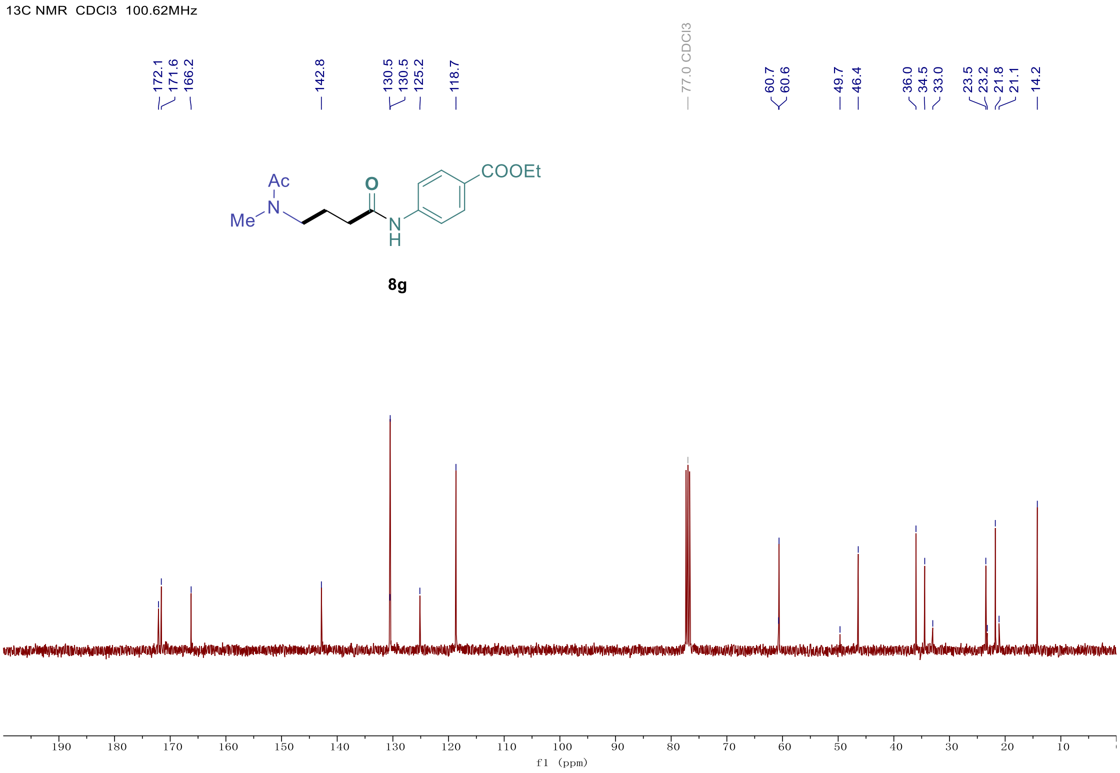

Supplementary Figure 186. ^{13}C NMR of compound **8g** (100 MHz, $CDCl_3$)

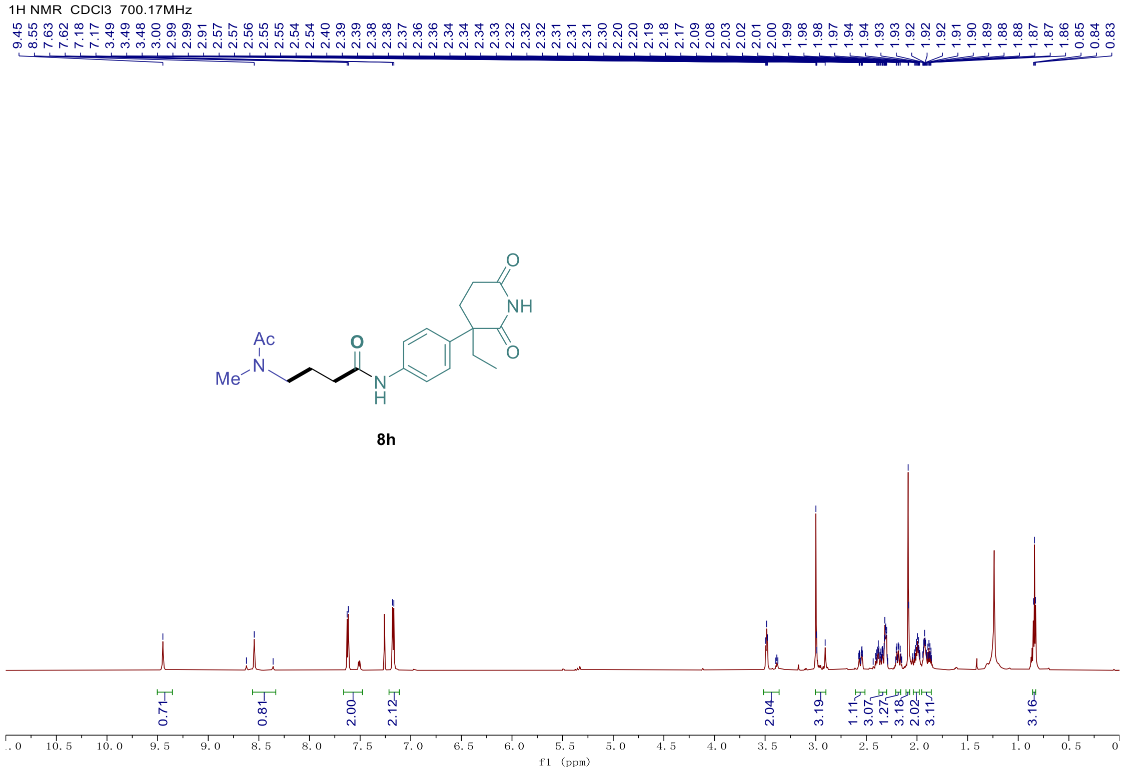

Supplementary Figure 187. ^{1}H NMR of compound **8h** (700 MHz, $CDCl_3$)

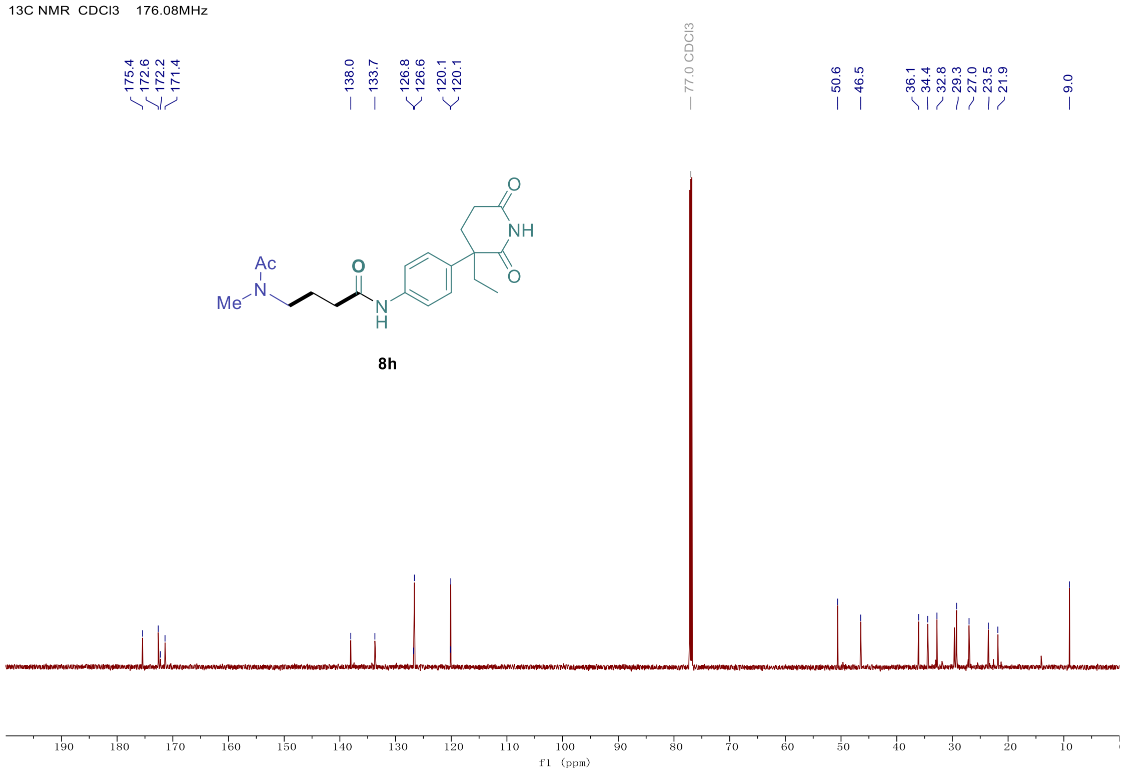

Supplementary Figure 188. ^{13}C NMR of compound **8h** (176 MHz, $CDCl_3$)

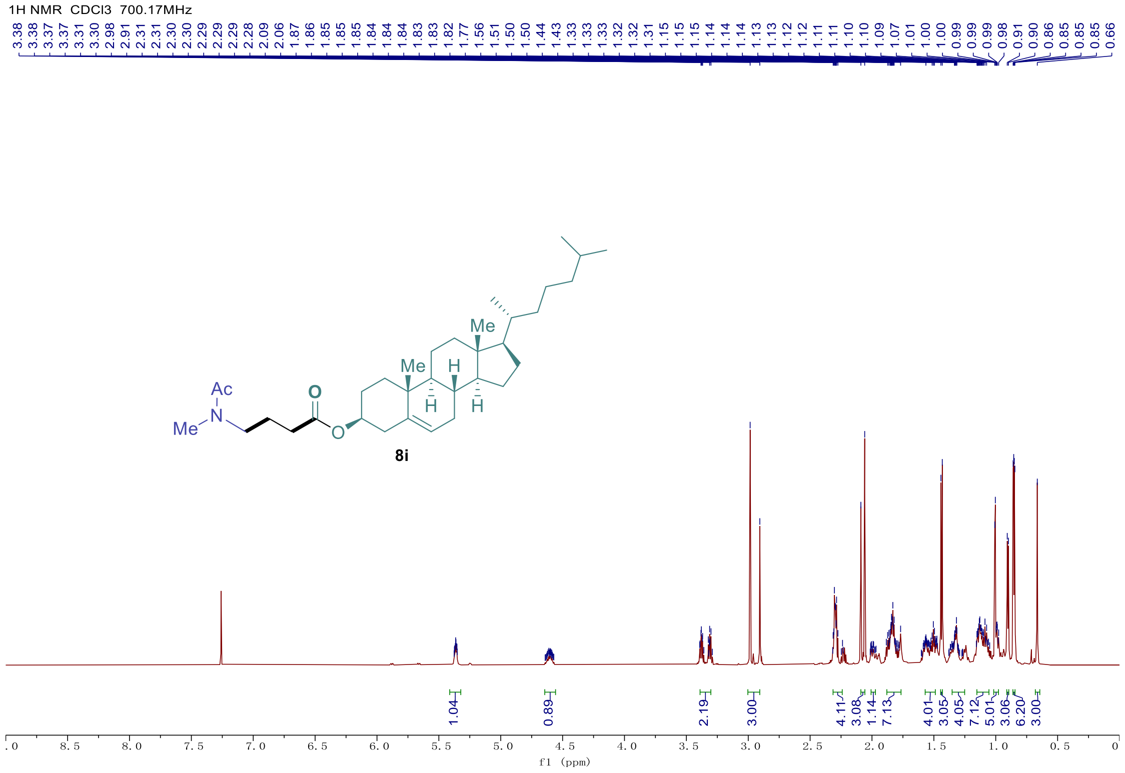

Supplementary Figure 189. ^{1}H NMR of compound **8i** (700 MHz, $CDCl_3$)

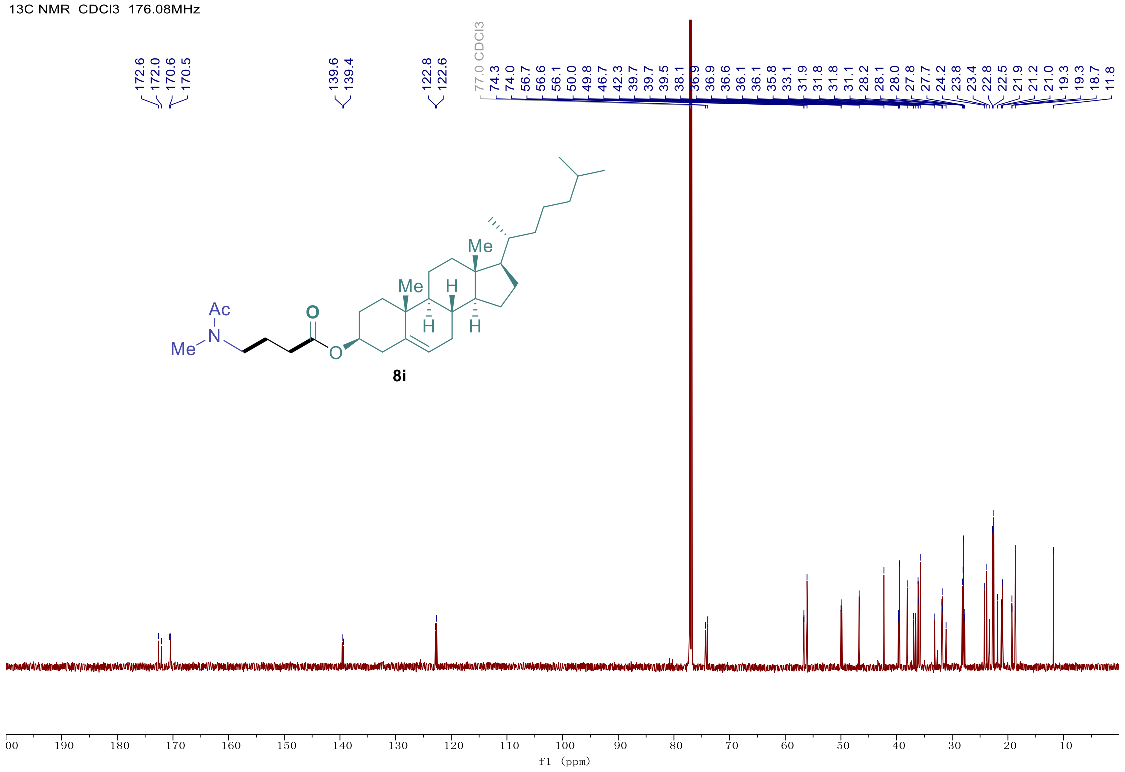

Supplementary Figure 190. ^{13}C NMR of compound **8i** (176 MHz, $CDCl_3$)

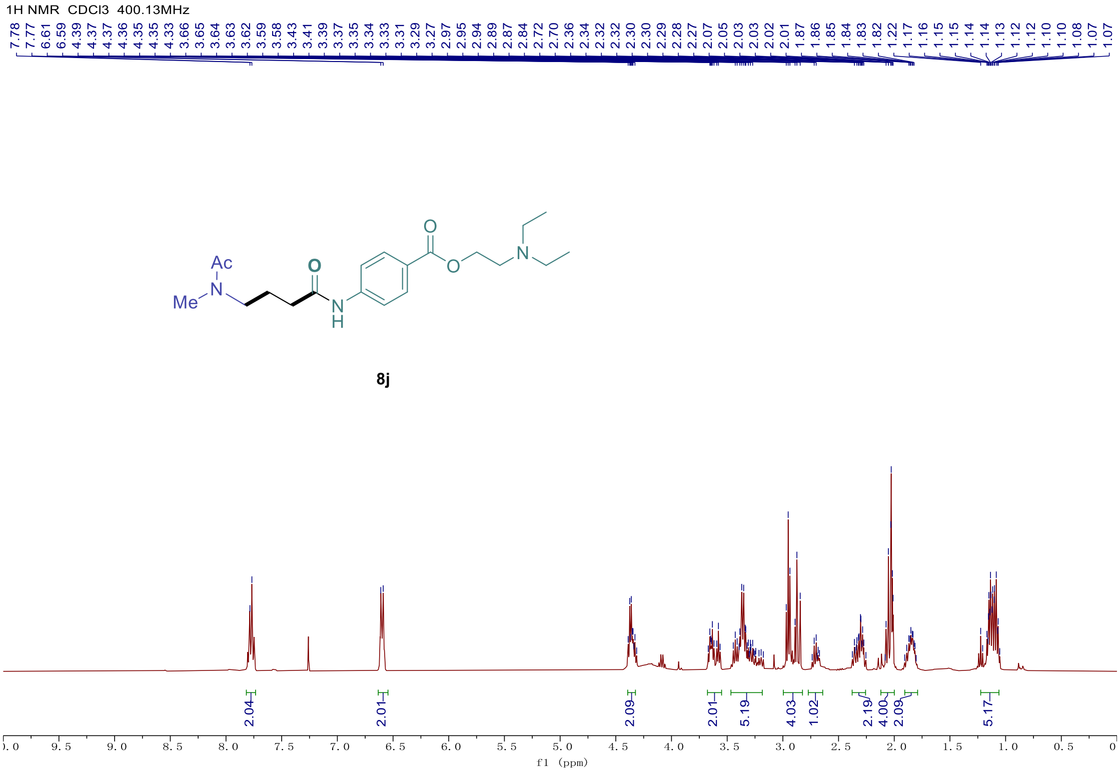

Supplementary Figure 191. ^{1}H NMR of compound **8j** (400 MHz, $CDCl_3$)

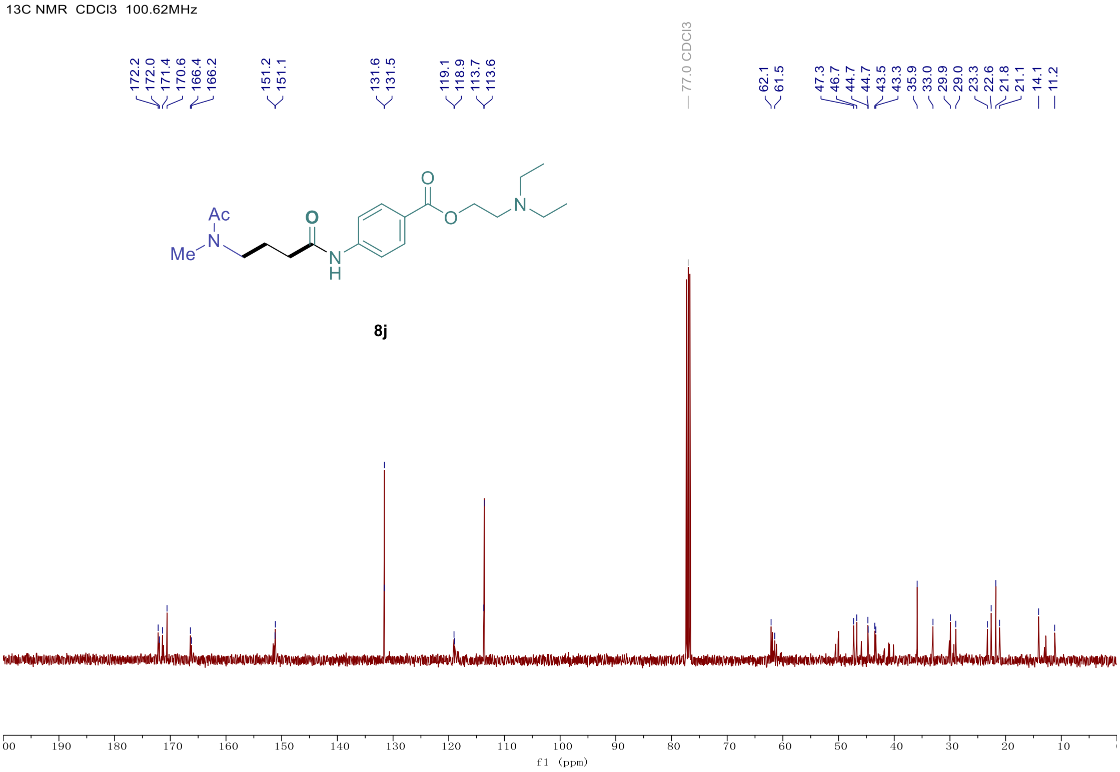

Supplementary Figure 192. ^{13}C NMR of compound **8j** (100 MHz, $CDCl_3$)

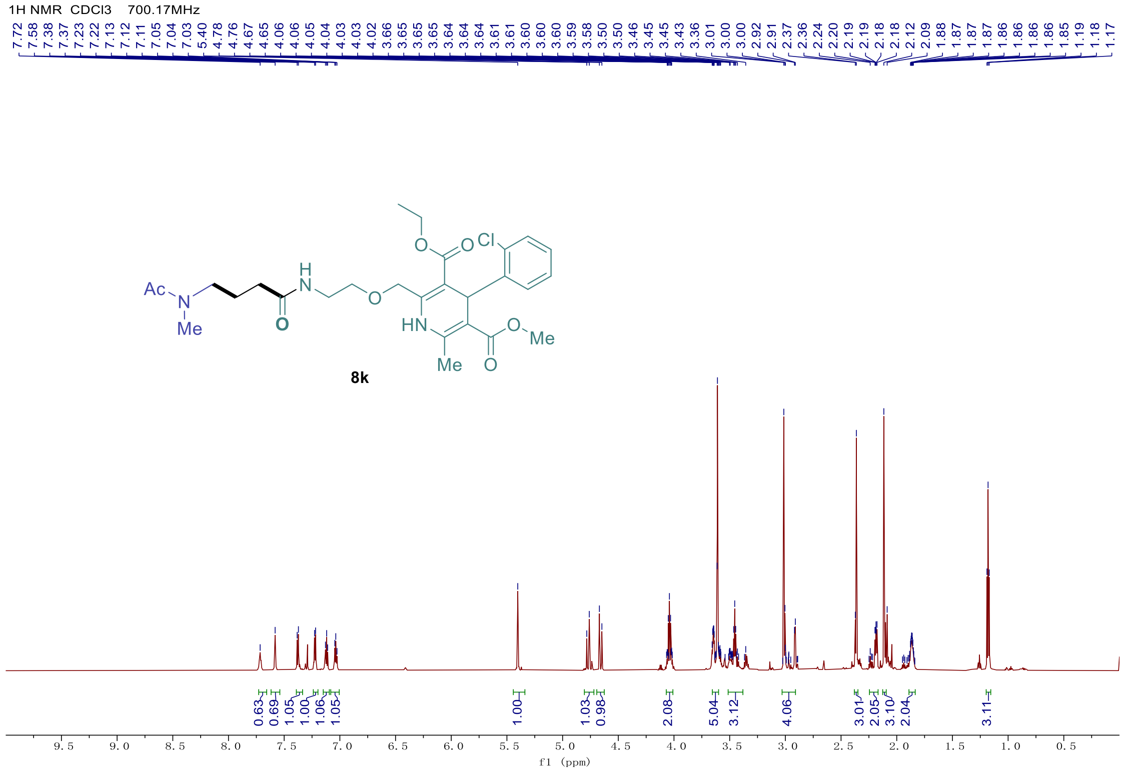

Supplementary Figure 193. ^{1}H NMR of compound **8k** (700 MHz, $CDCl_3$)

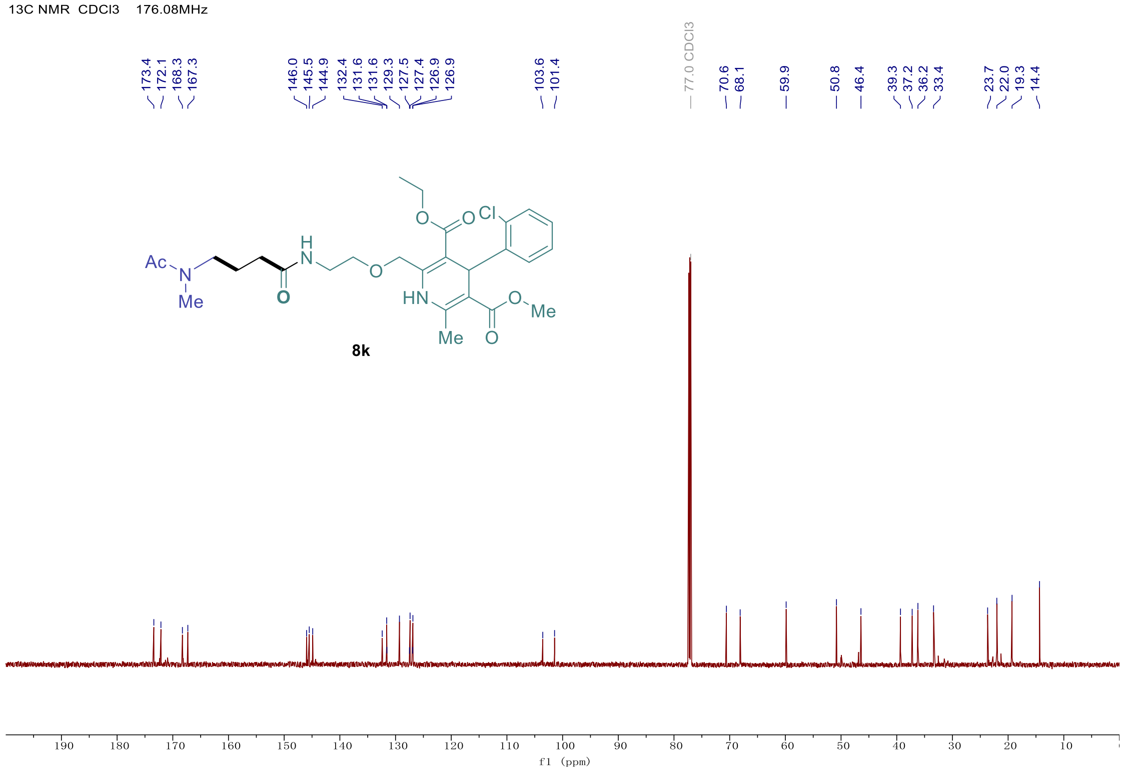

Supplementary Figure 194. ^{13}C NMR of compound **8k** (176 MHz, $CDCl_3$)

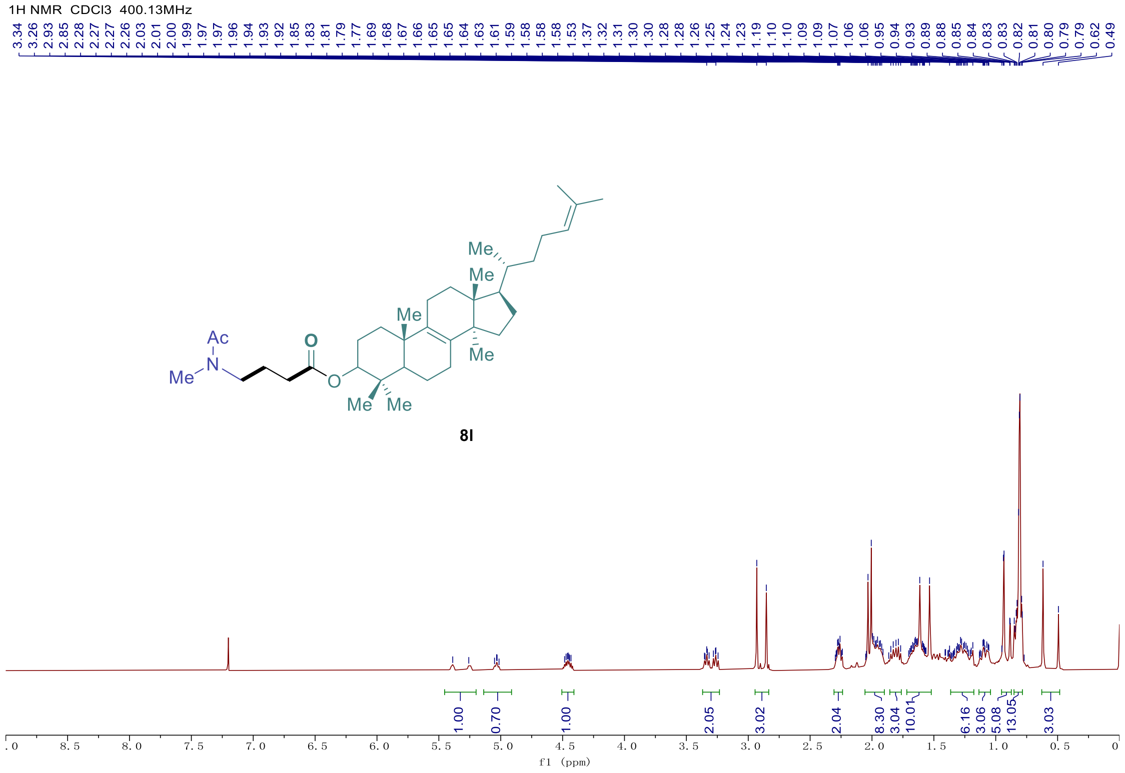

Supplementary Figure 195. ^{1}H NMR of compound **8l** (400 MHz, $CDCl_3$)

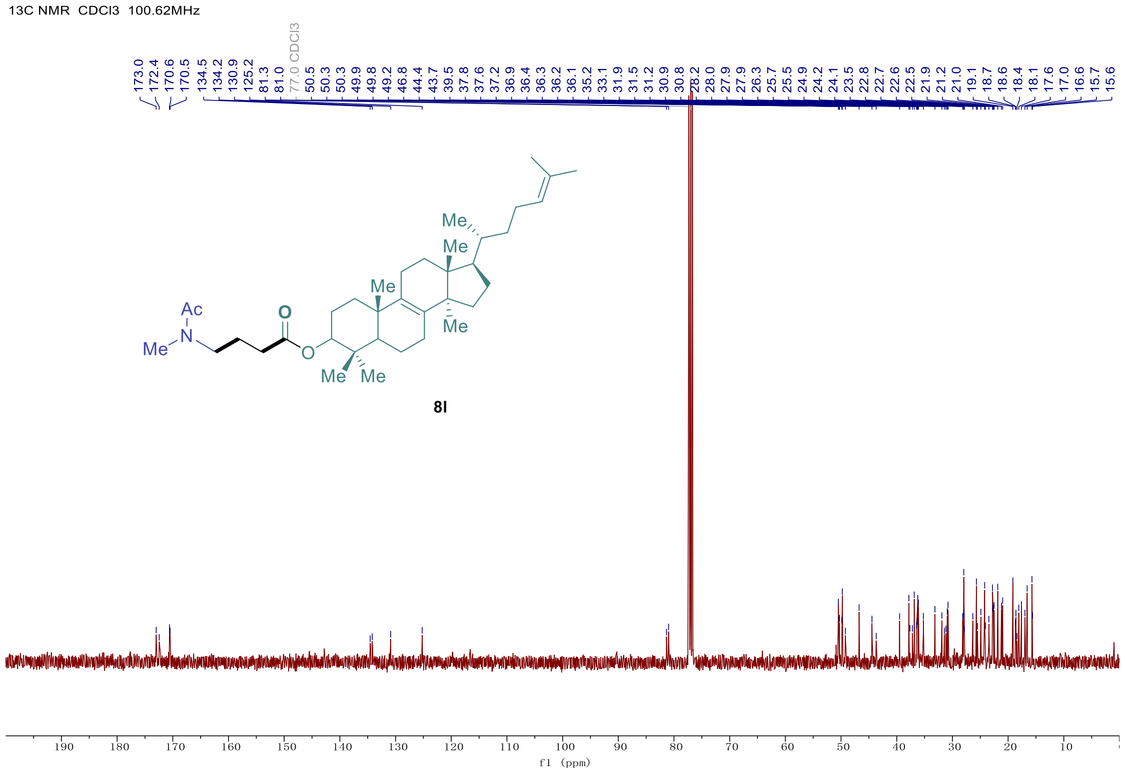

Supplementary Figure 196. ^{13}C NMR of compound **8l** (100 MHz, $CDCl_3$)

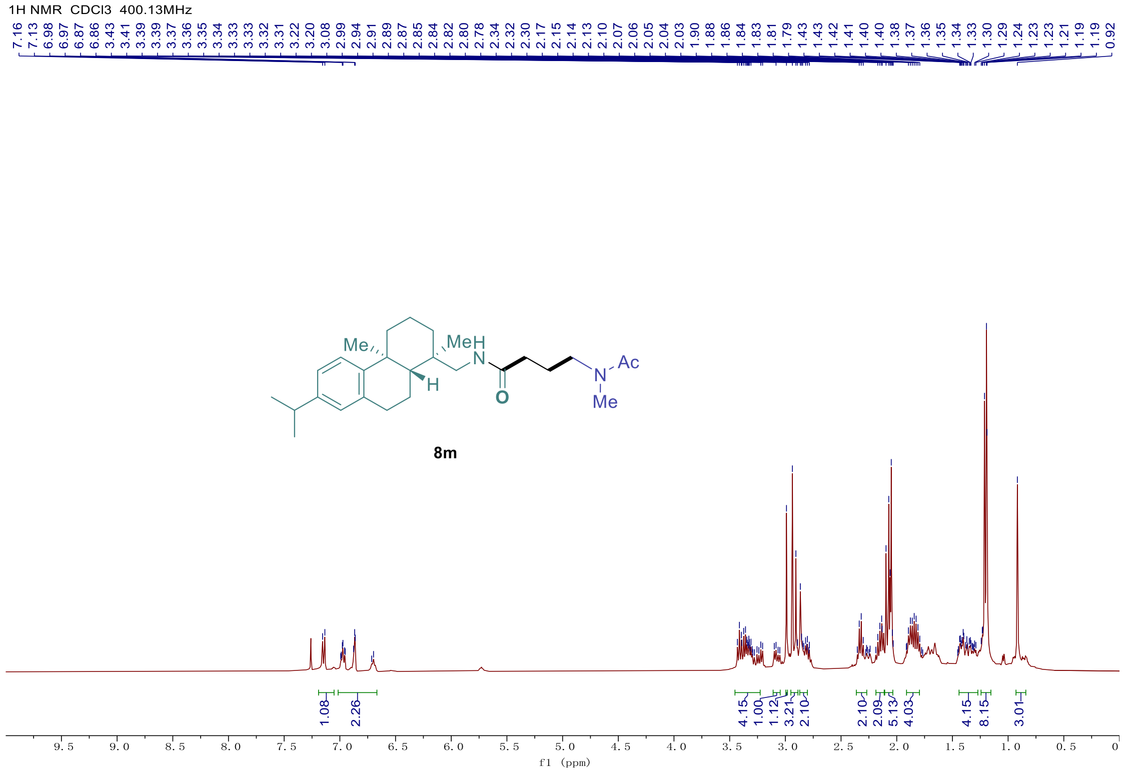

Supplementary Figure 197. ^{1}H NMR of compound **8m** (400 MHz, $CDCl_3$)

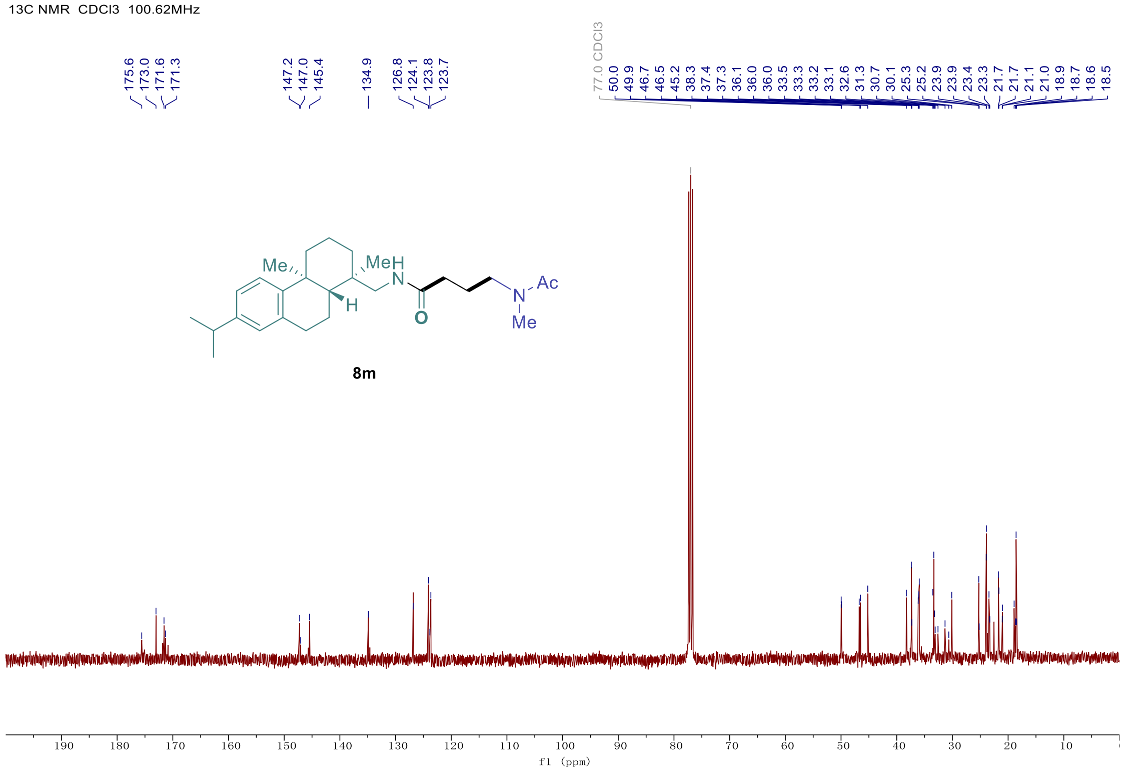

Supplementary Figure 198. ^{13}C NMR of compound **8m** (100 MHz, $CDCl_3$)

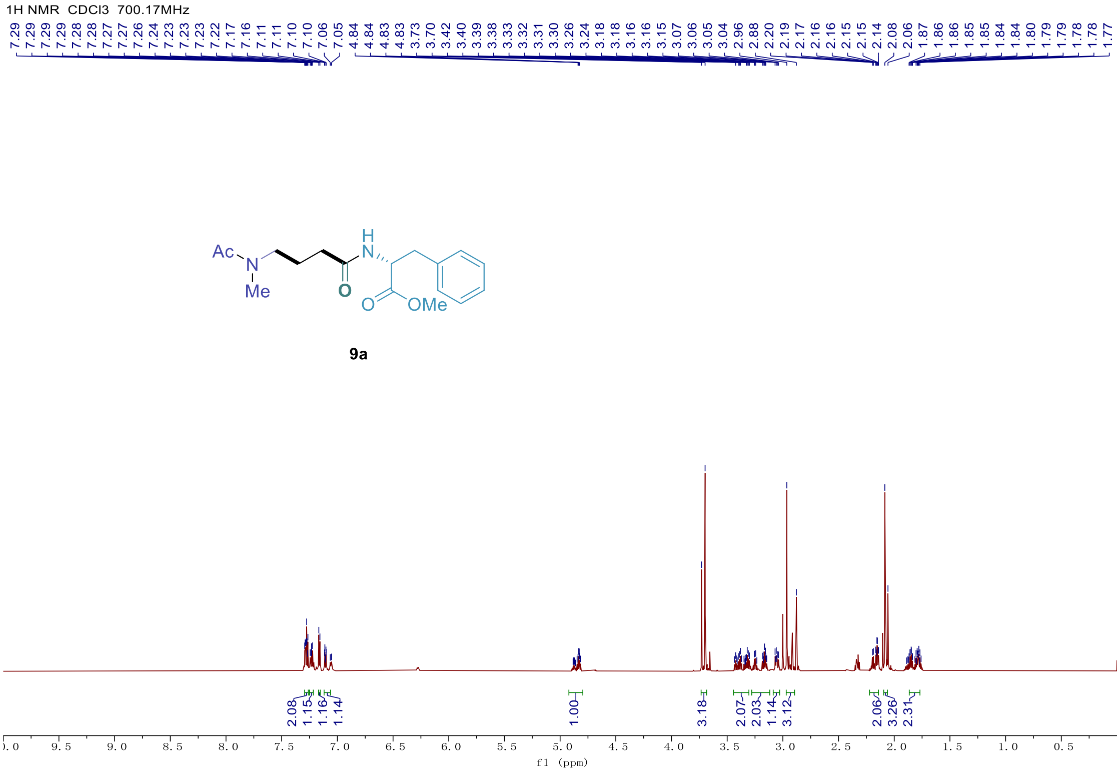

Supplementary Figure 199. ^{1}H NMR of compound **9a** (700 MHz, $CDCl_3$)

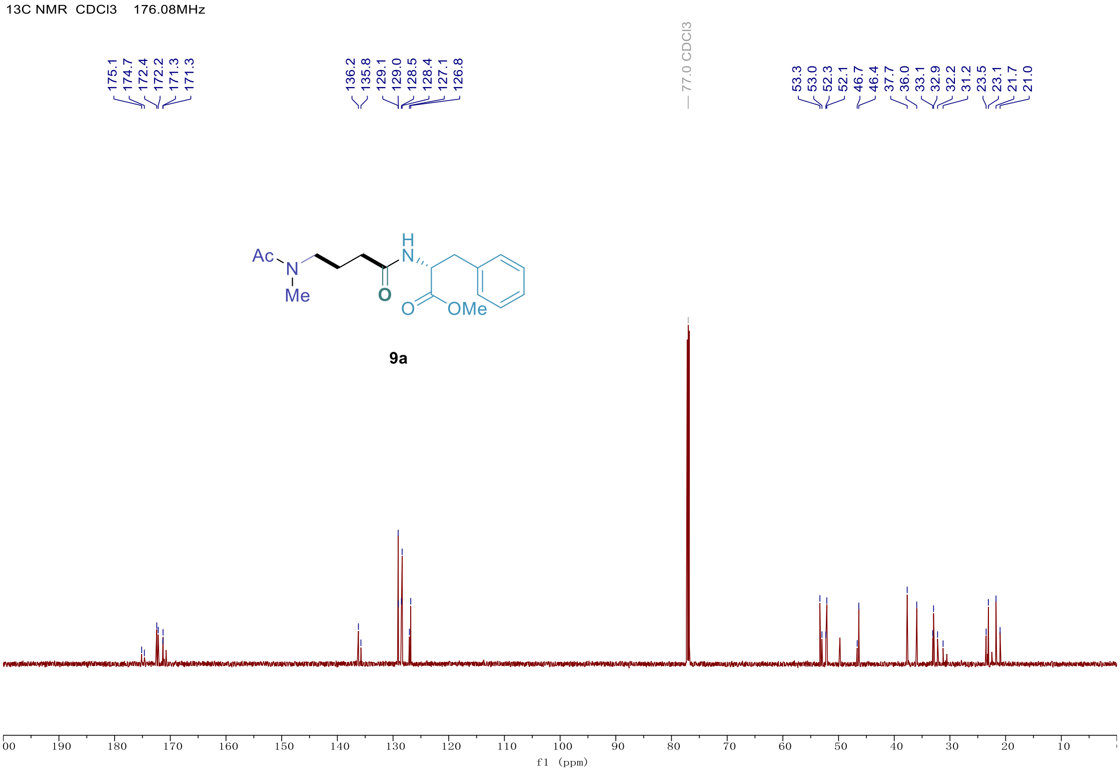

Supplementary Figure 200. ^{13}C NMR of compound **9a** (176 MHz, $CDCl_3$)

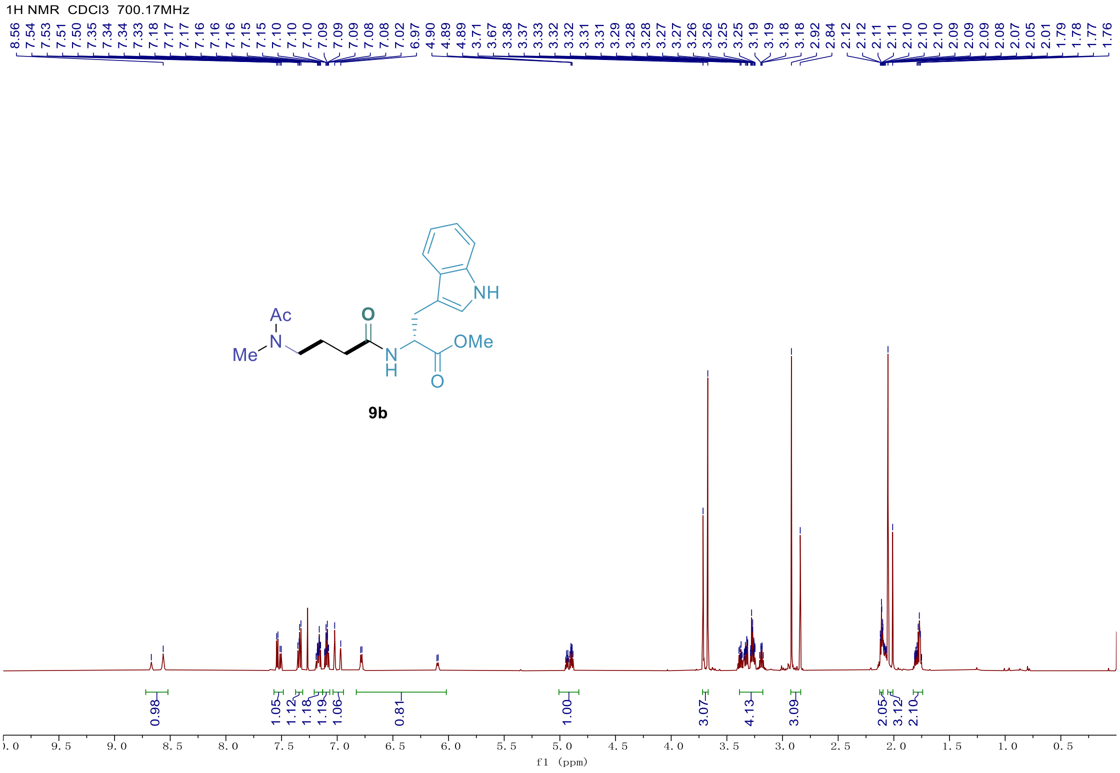

Supplementary Figure 201. ^{1}H NMR of compound **9b** (700 MHz, $CDCl_3$)

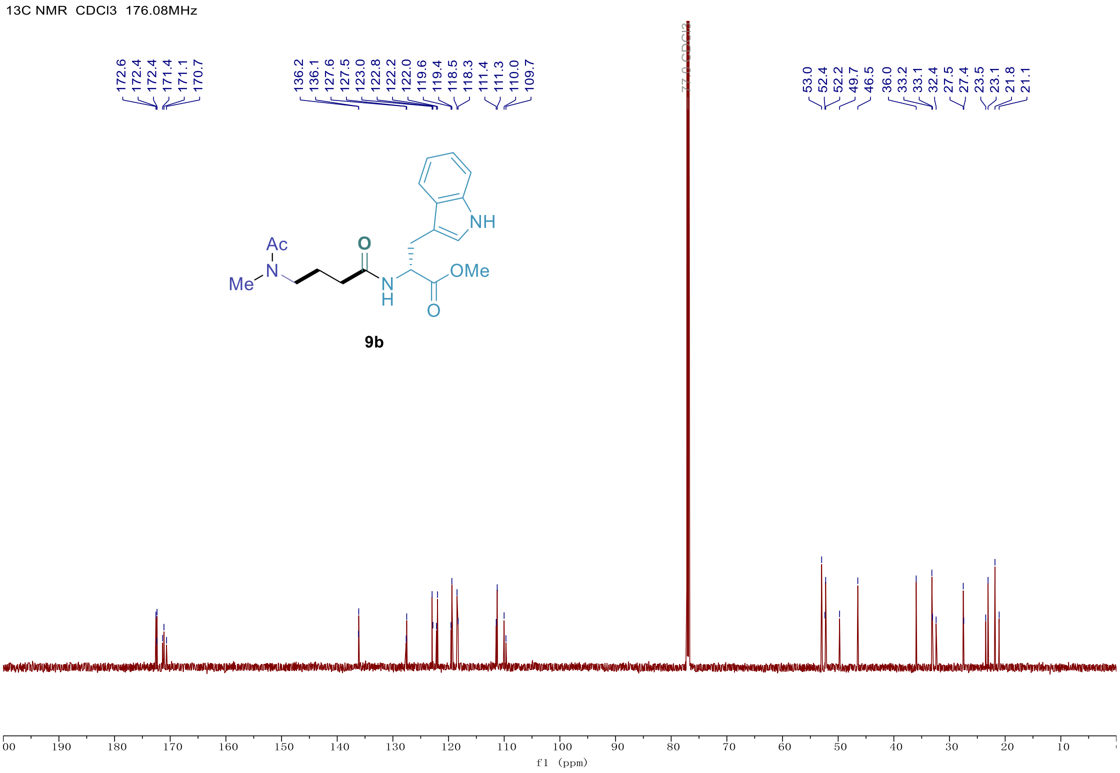

Supplementary Figure 202. ^{13}C NMR of compound **9b** (176 MHz, $CDCl_3$)

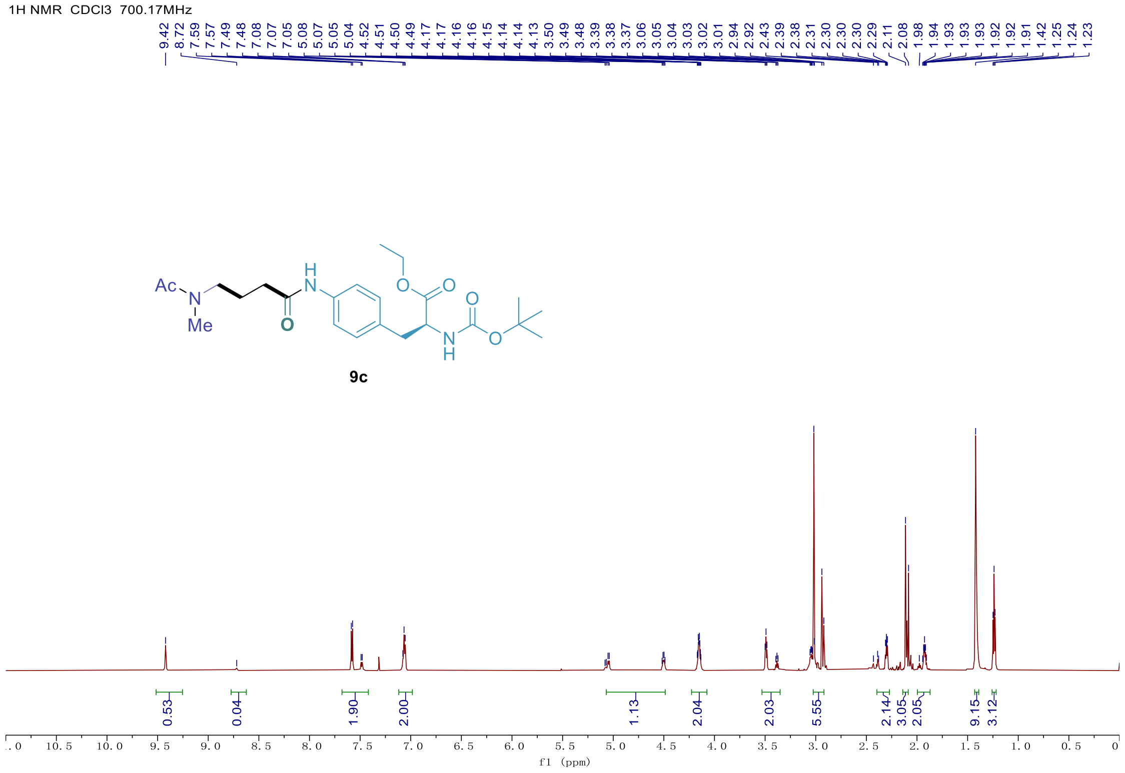

Supplementary Figure 203. ^{1}H NMR of compound **9c** (700 MHz, $CDCl_3$)

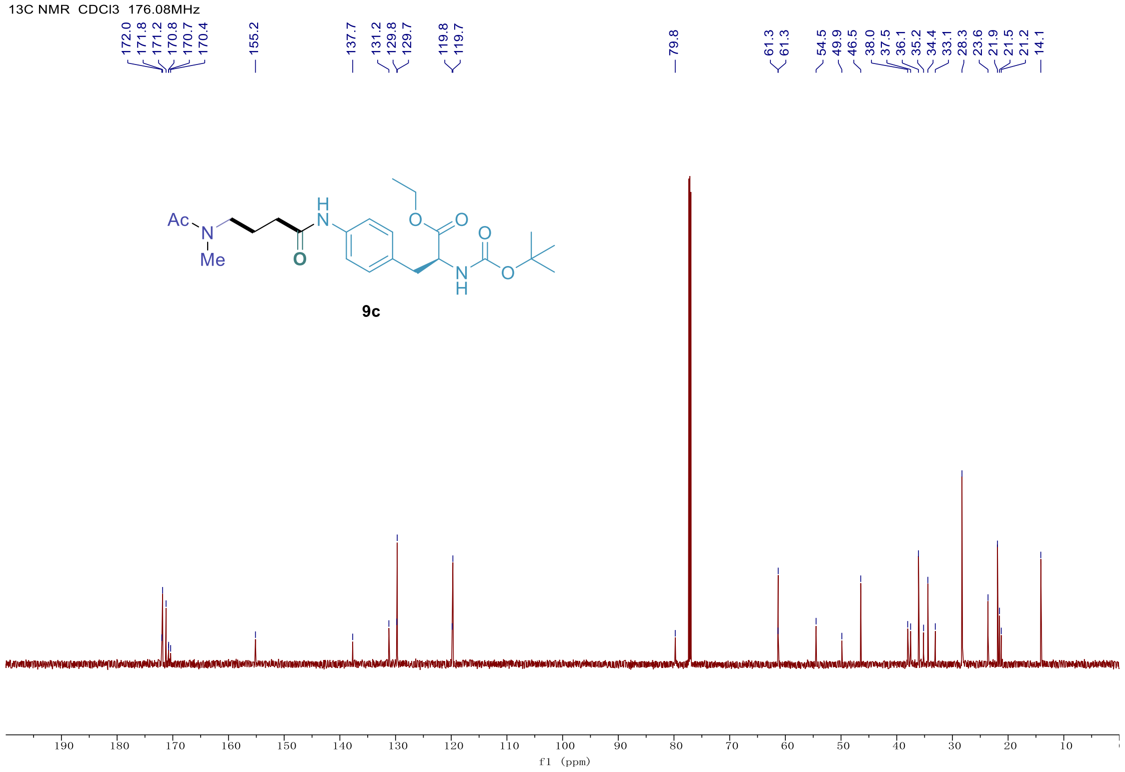

Supplementary Figure 204. ^{13}C NMR of compound **9c** (176 MHz, $CDCl_3$)

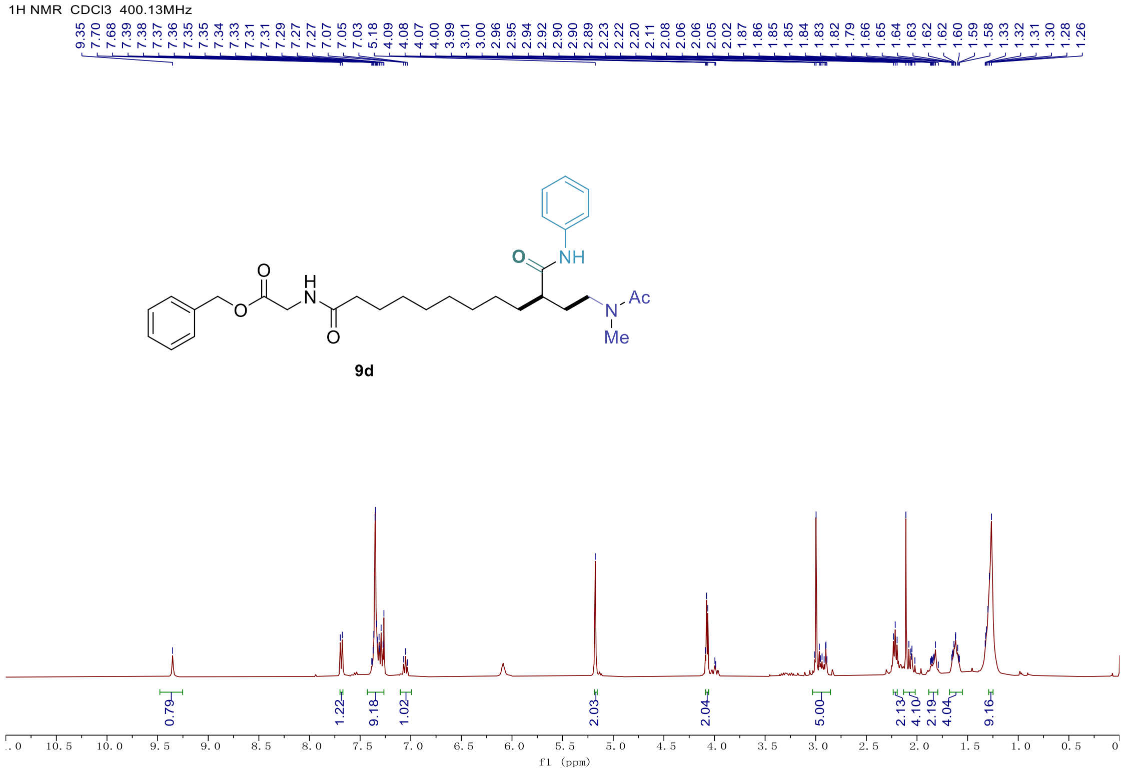

Supplementary Figure 205. ^{1}H NMR of compound **9d** (400 MHz, $CDCl_3$)

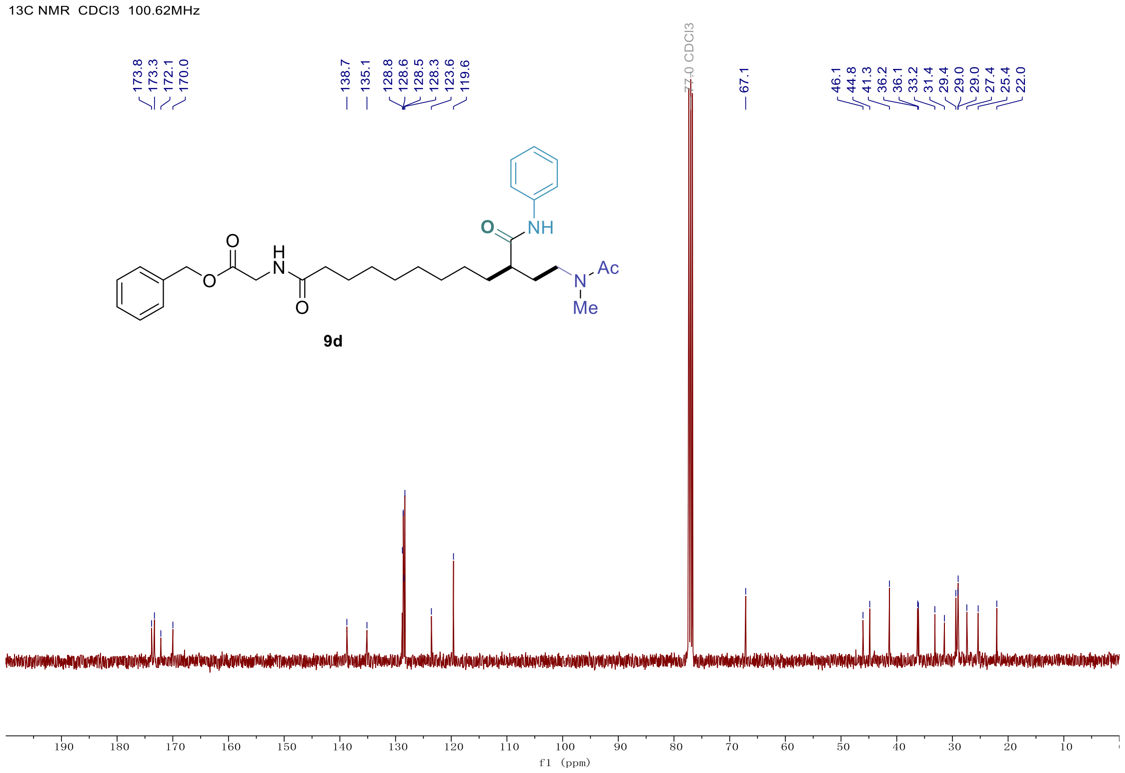

Supplementary Figure 206. ^{13}C NMR of compound **9d** (100 MHz, $CDCl_3$)

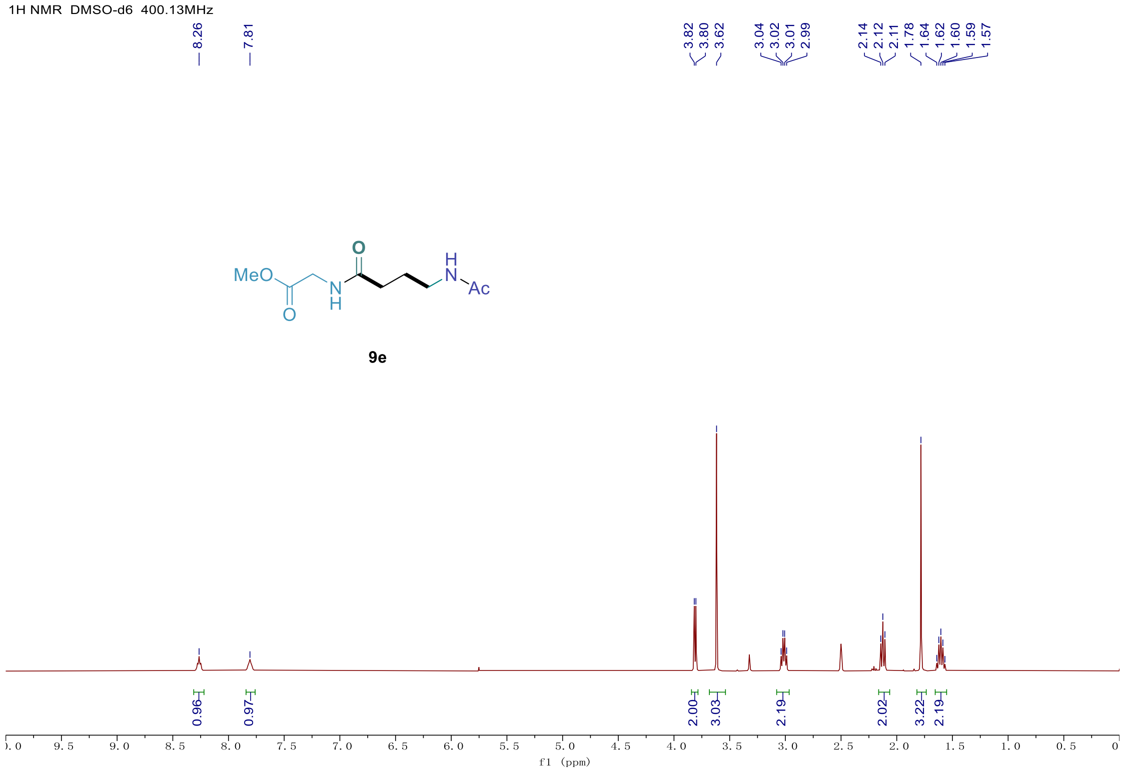

Supplementary Figure 207. ^{1}H NMR of compound **9e** (400 MHz, DMSO-*d6*)

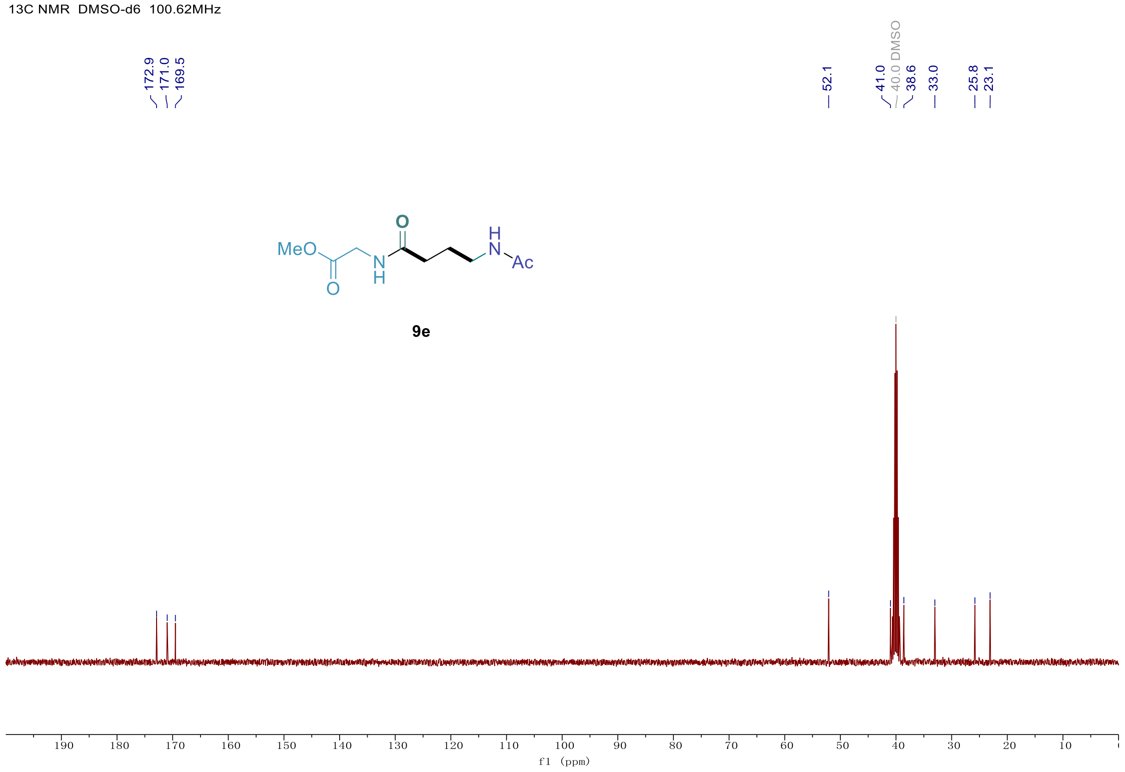

Supplementary Figure 208. ^{13}C NMR of compound **9e** (100 MHz, DMSO-*d6*)

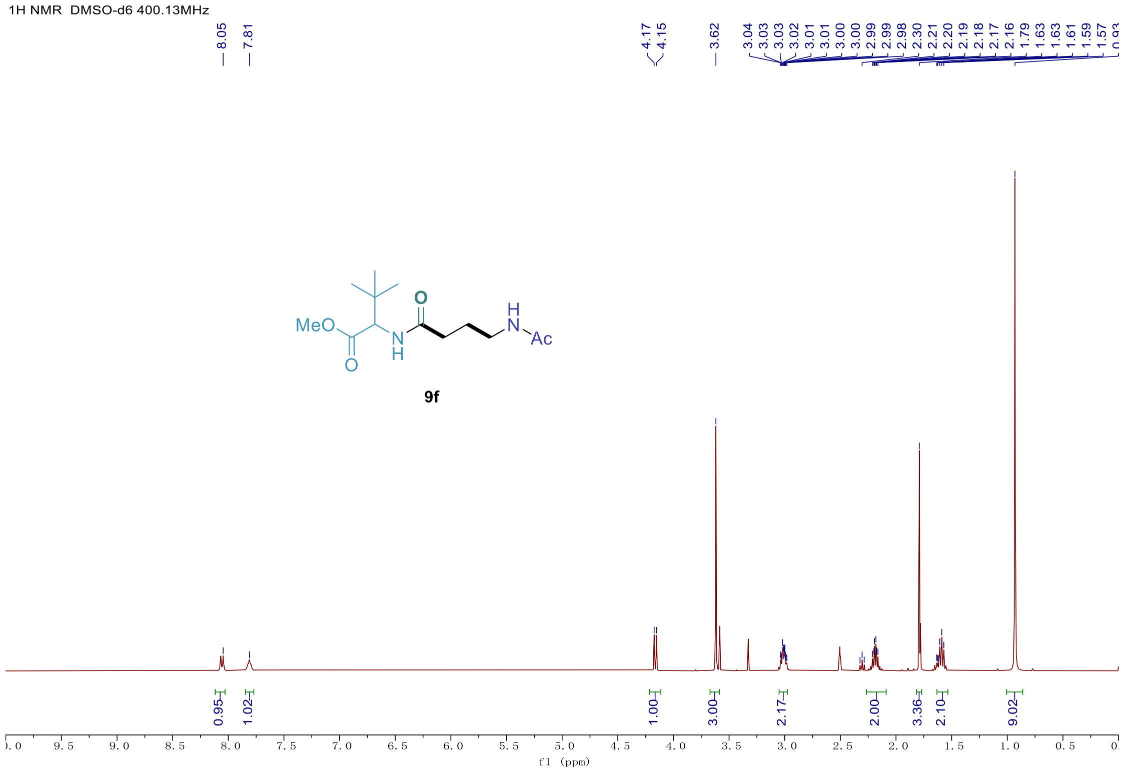

Supplementary Figure 209. ^{1}H NMR of compound **9f** (400 MHz, DMSO-*d6*)

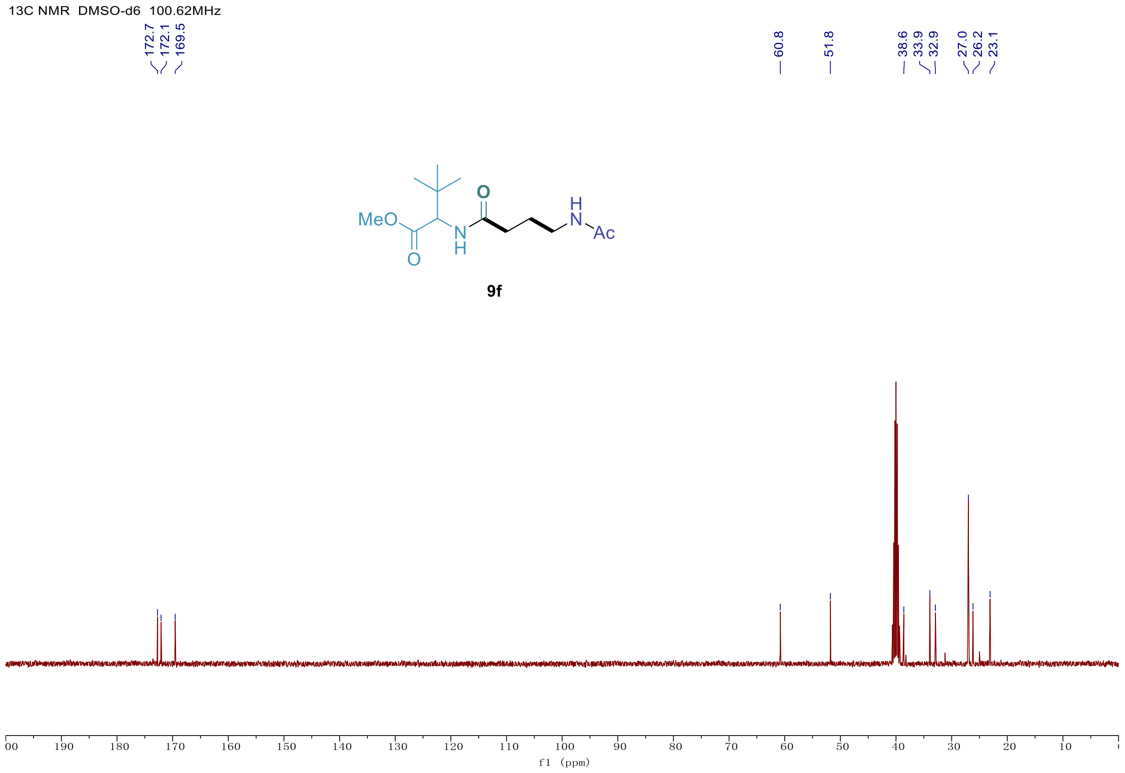

Supplementary Figure 210. ^{13}C NMR of compound **9f** (100 MHz, DMSO-*d6*)

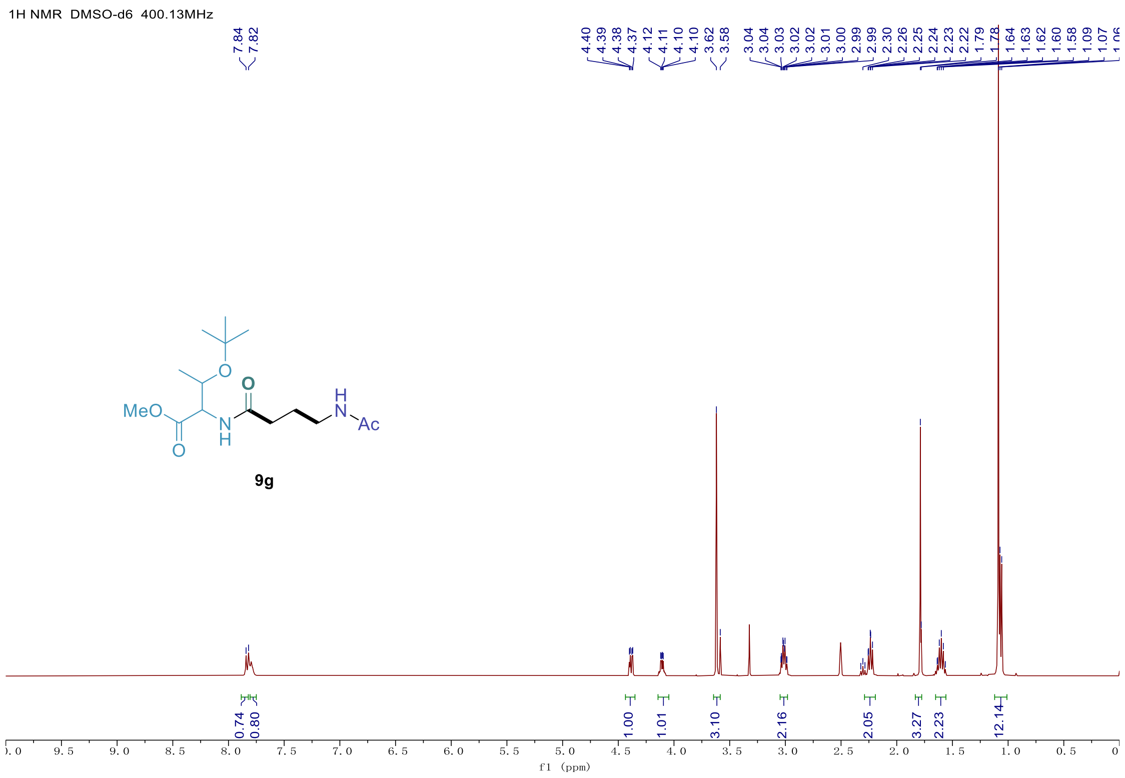

Supplementary Figure 211. ^{1}H NMR of compound **9g** (400 MHz, DMSO-*d6*)

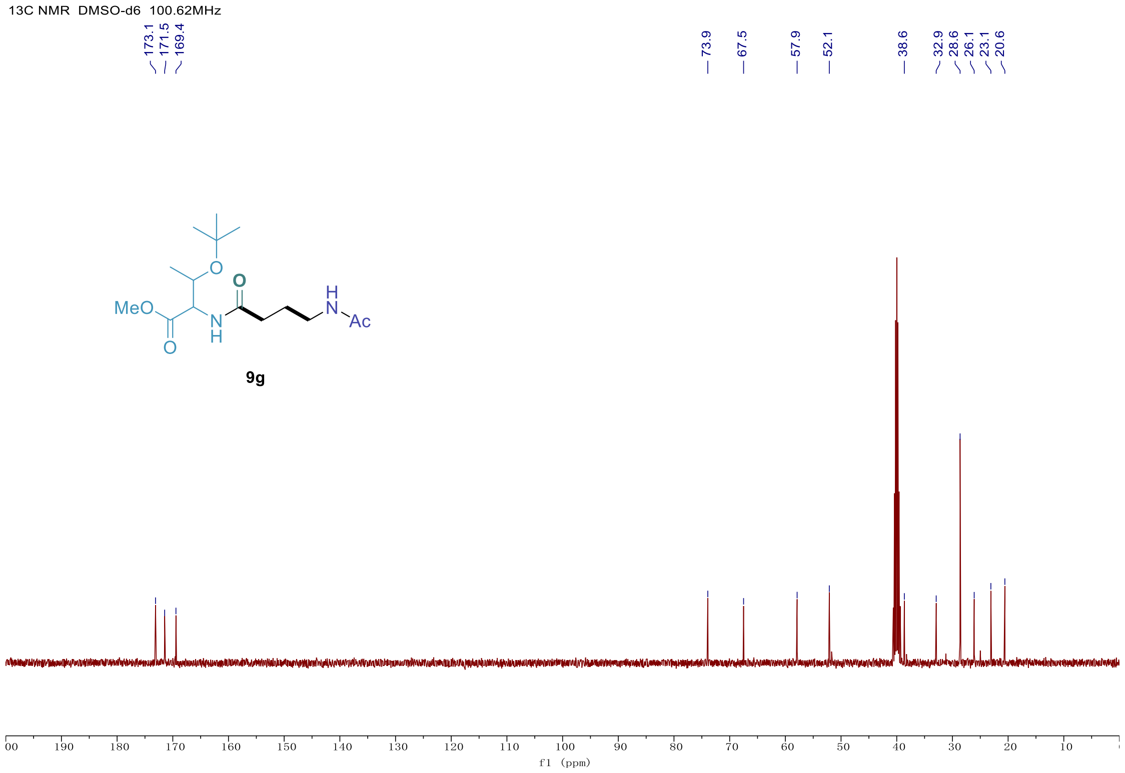

Supplementary Figure 212. ^{13}C NMR of compound **9g** (100 MHz, DMSO-*d6*)

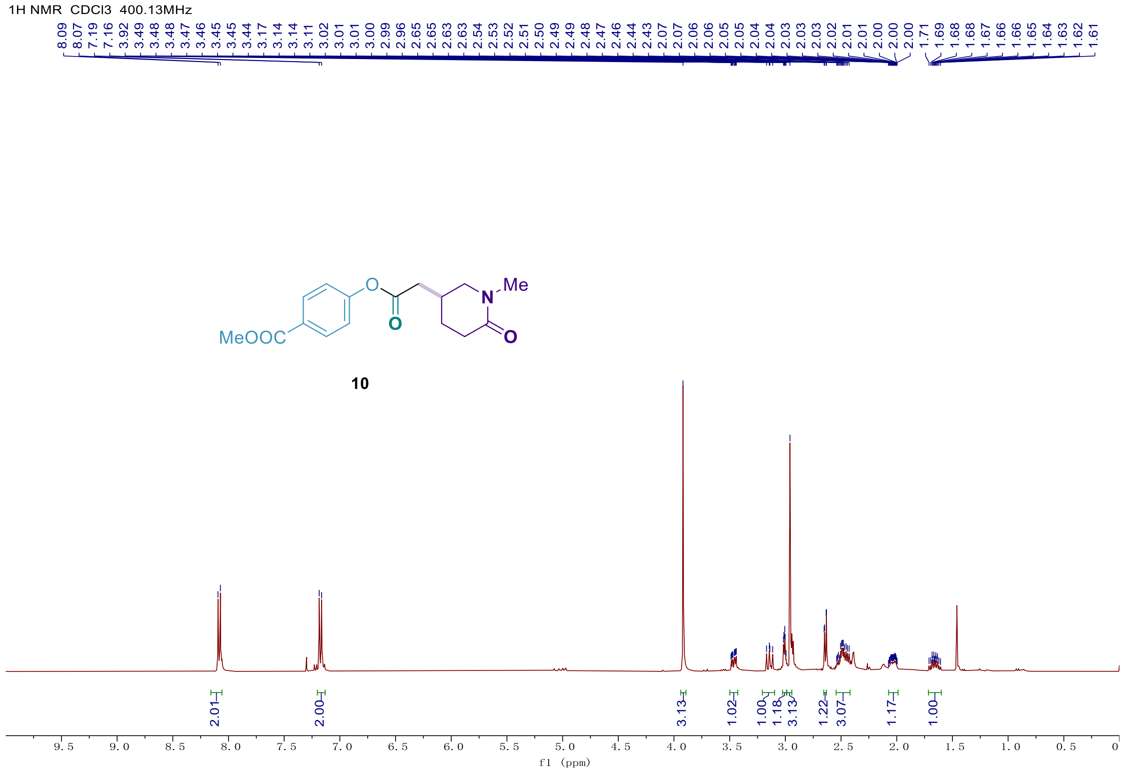

Supplementary Figure 213. ^{1}H NMR of compound **9h** (400 MHz, $CDCl_3$)

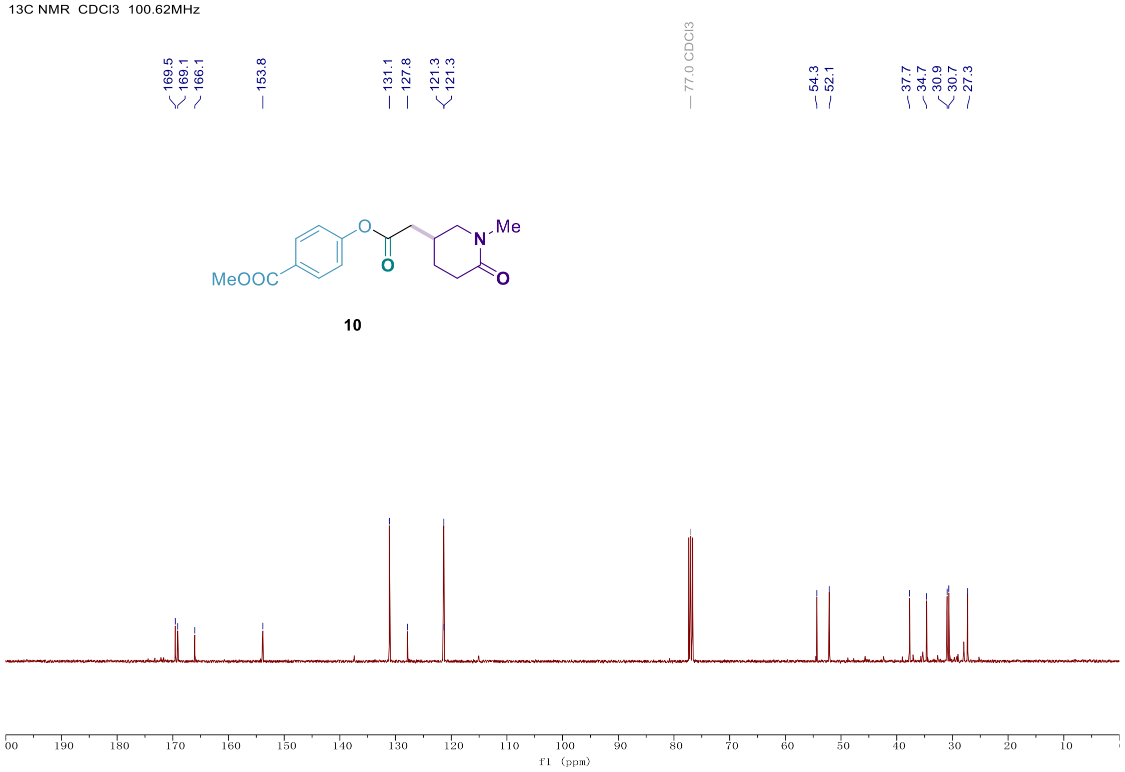

Supplementary Figure 214. ^{13}C NMR of compound **9h** (100 MHz, $CDCl_3$)

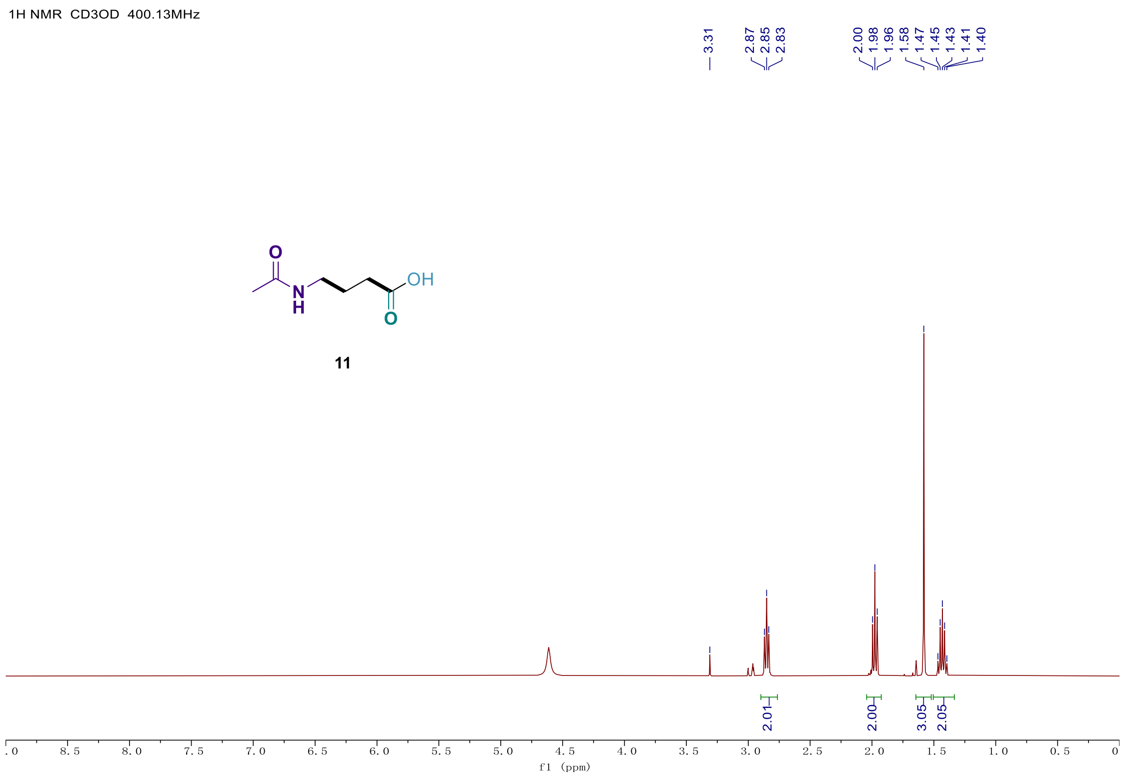

Supplementary Figure 215. ^{1}H NMR of compound **11** (400 MHz, CD_3OD)

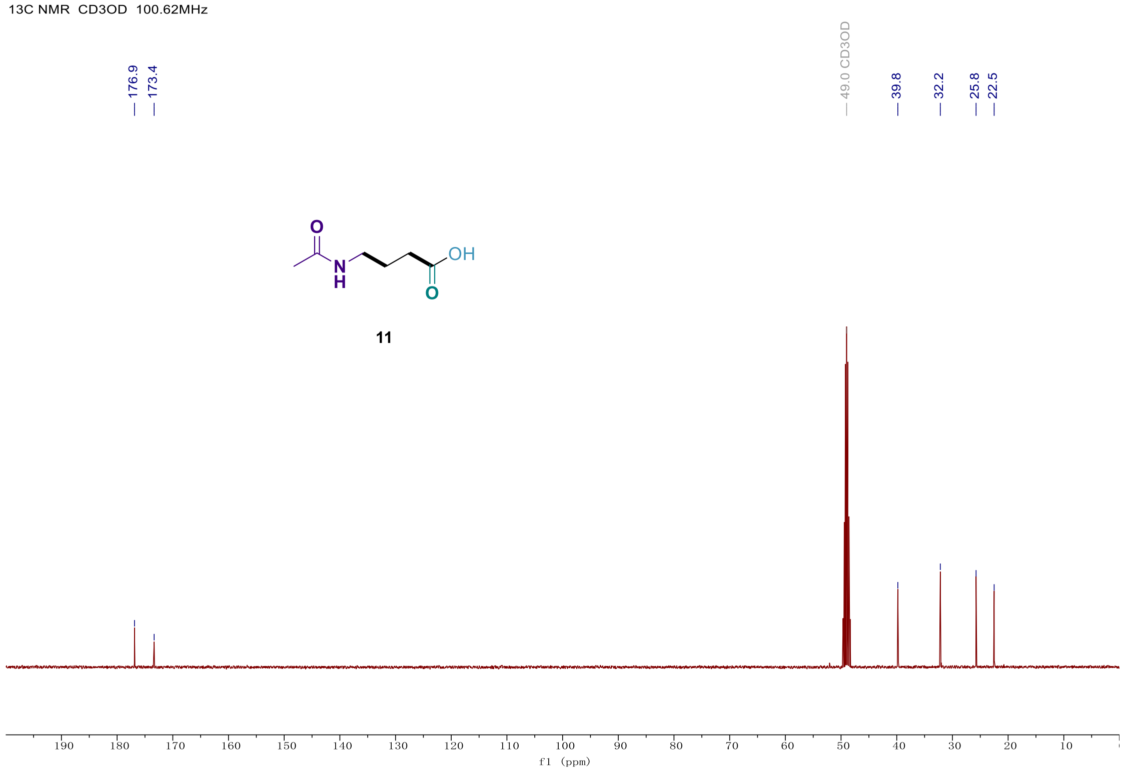

Supplementary Figure 216. ^{13}C NMR of compound **11** (100 MHz, CD_3OD)

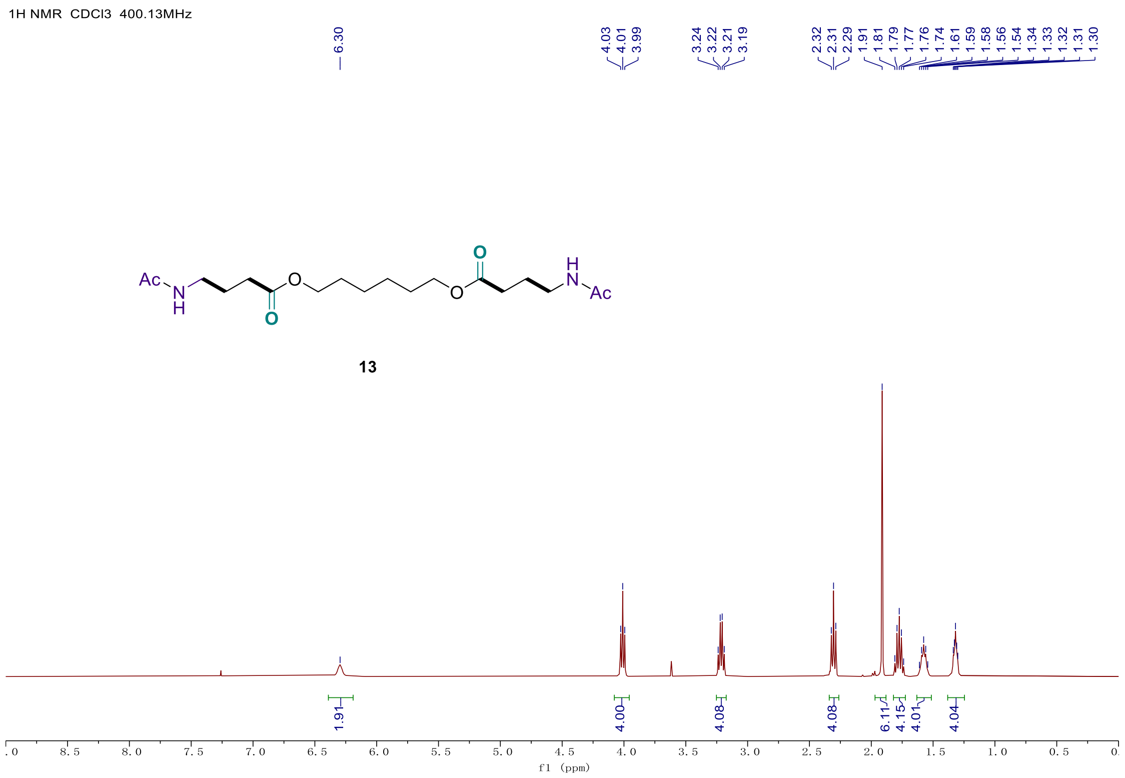

Supplementary Figure 217. ^{1}H NMR of compound **13** (400 MHz, $CDCl_3$)

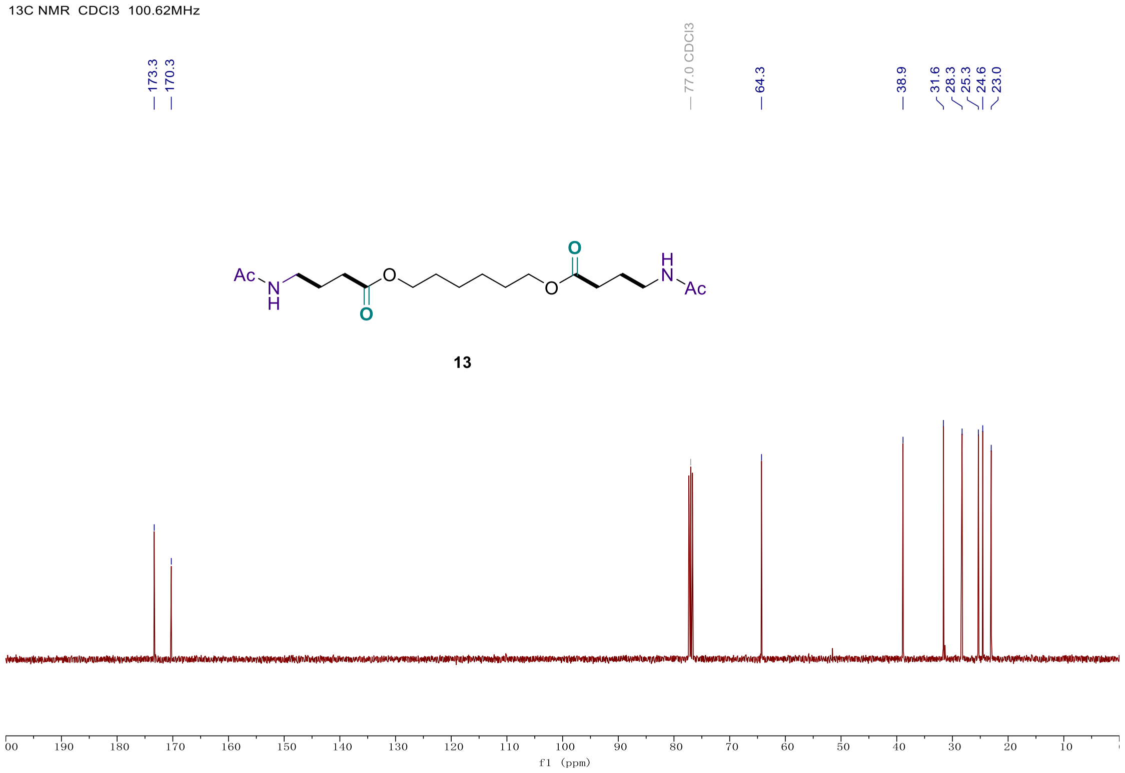

Supplementary Figure 218. ^{13}C NMR of compound **13** (100 MHz, $CDCl_3$)

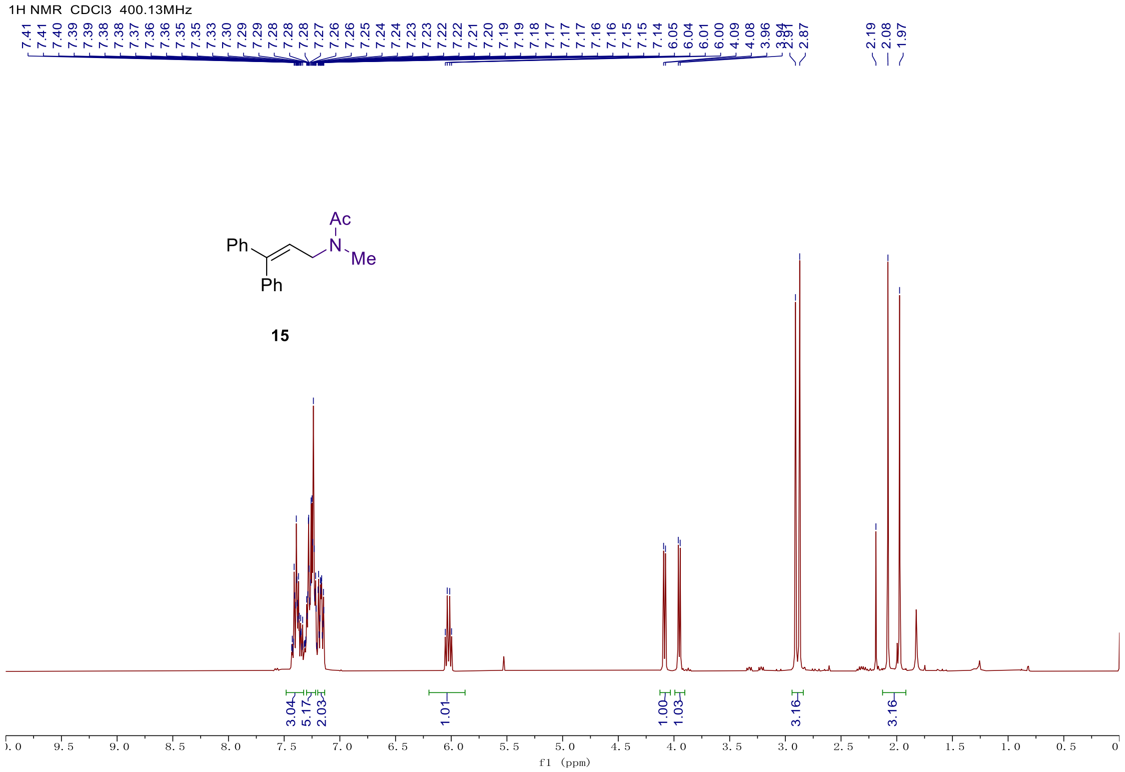

Supplementary Figure 219. ^{1}H NMR of compound **15** (400 MHz, $CDCl_3$)

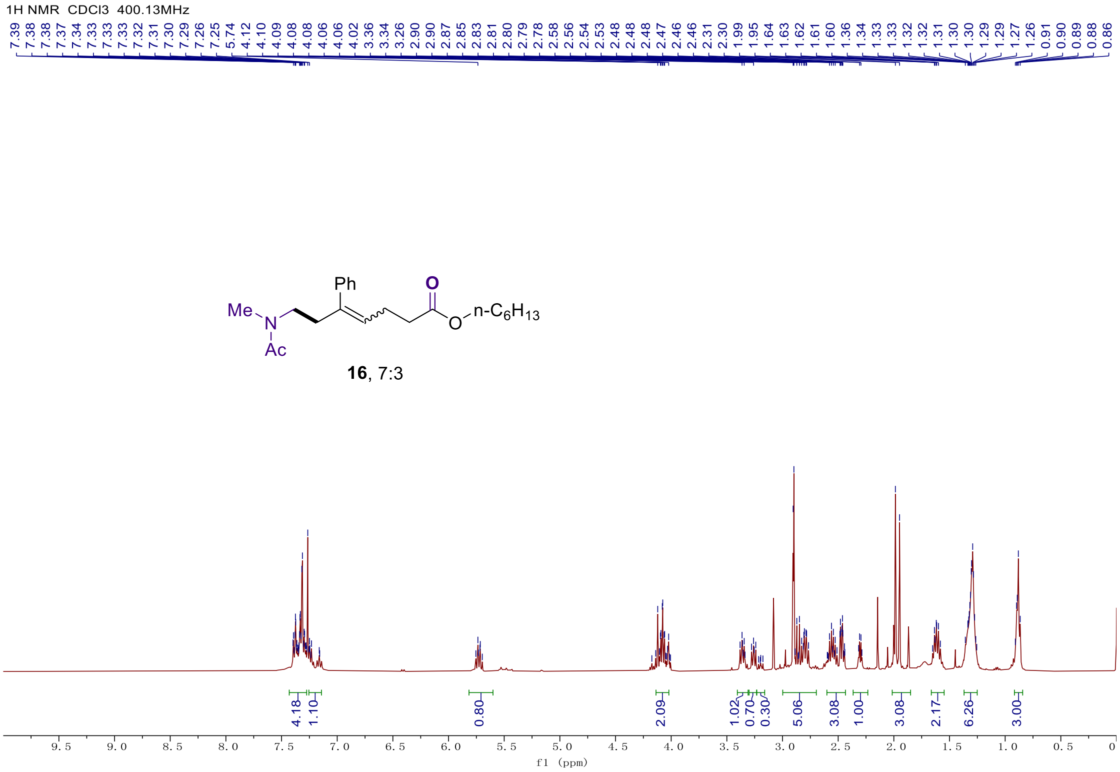

Supplementary Figure 220. ^{1}H NMR of compound **16** (400 MHz, $CDCl_3$)

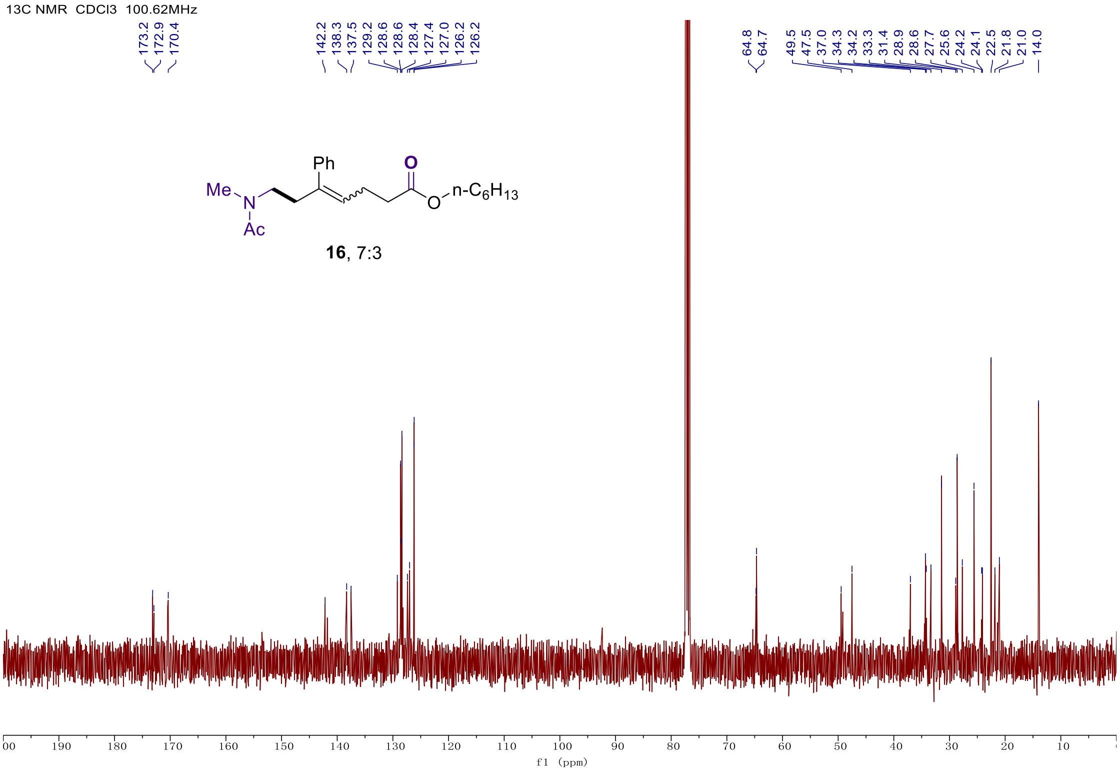

Supplementary Figure 221. ^{13}C NMR of compound **16** (100 MHz, $CDCl_3$)

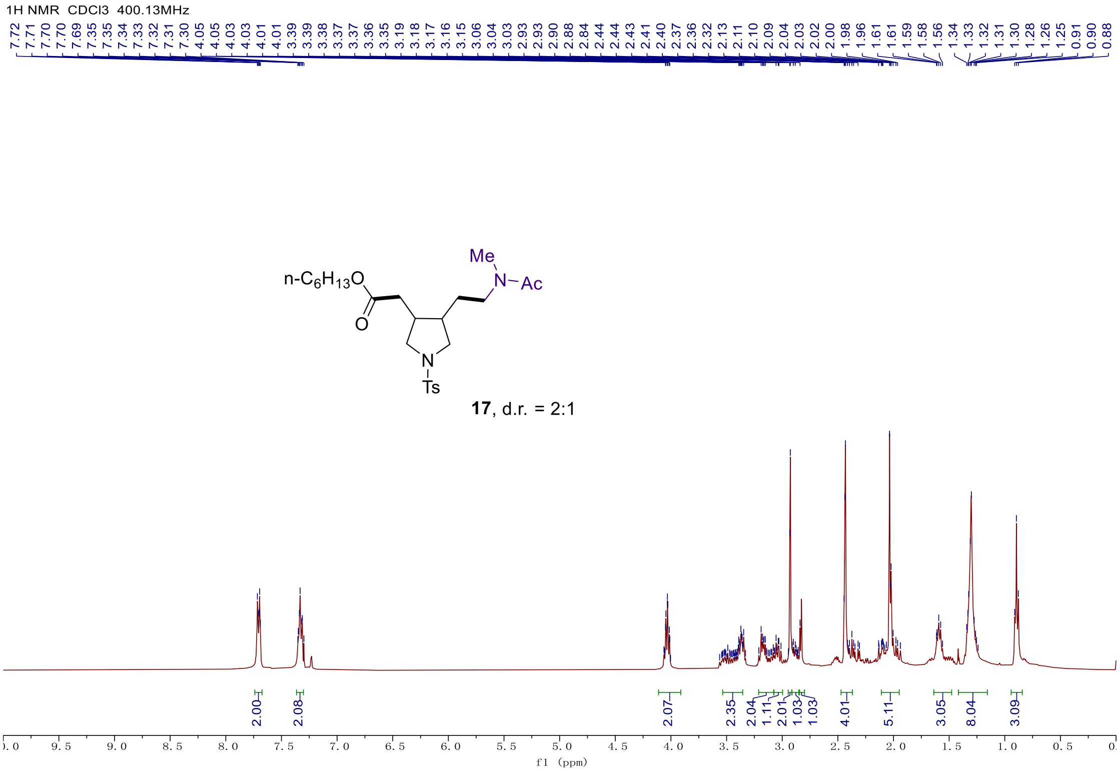

Supplementary Figure 222. ^{1}H NMR of compound **17** (400 MHz, $CDCl_3$)

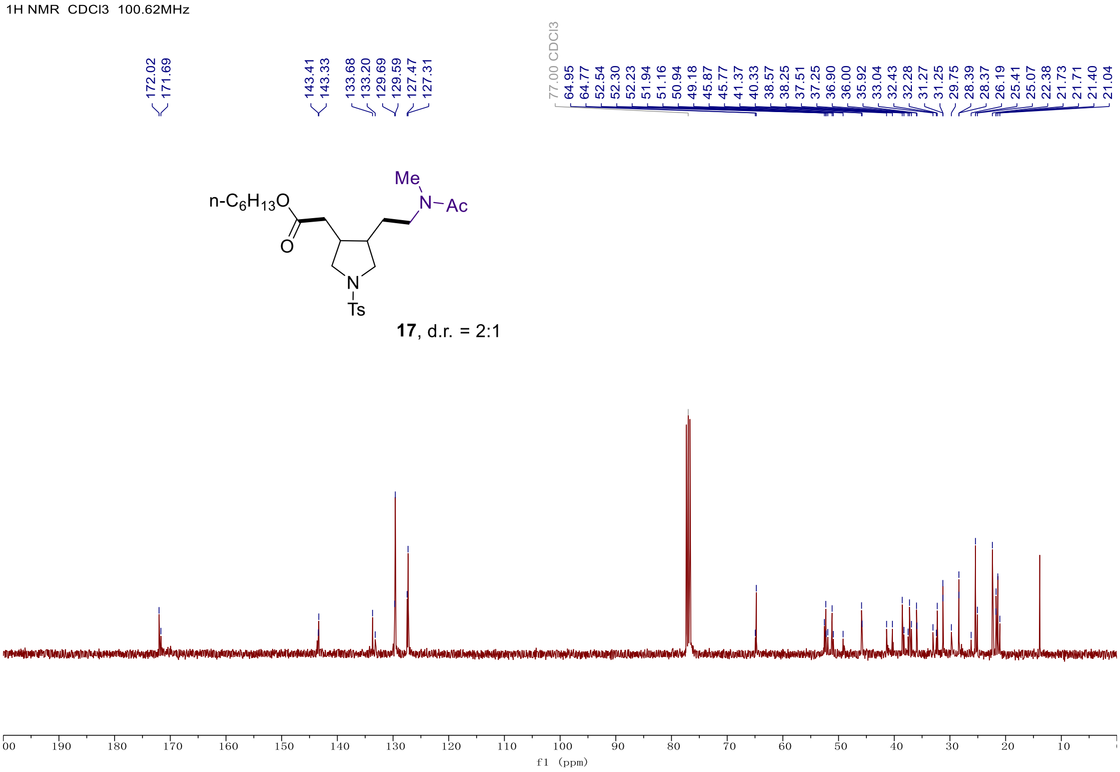

Supplementary Figure 223. ^{13}C NMR of compound **17** (100 MHz, $CDCl_3$)

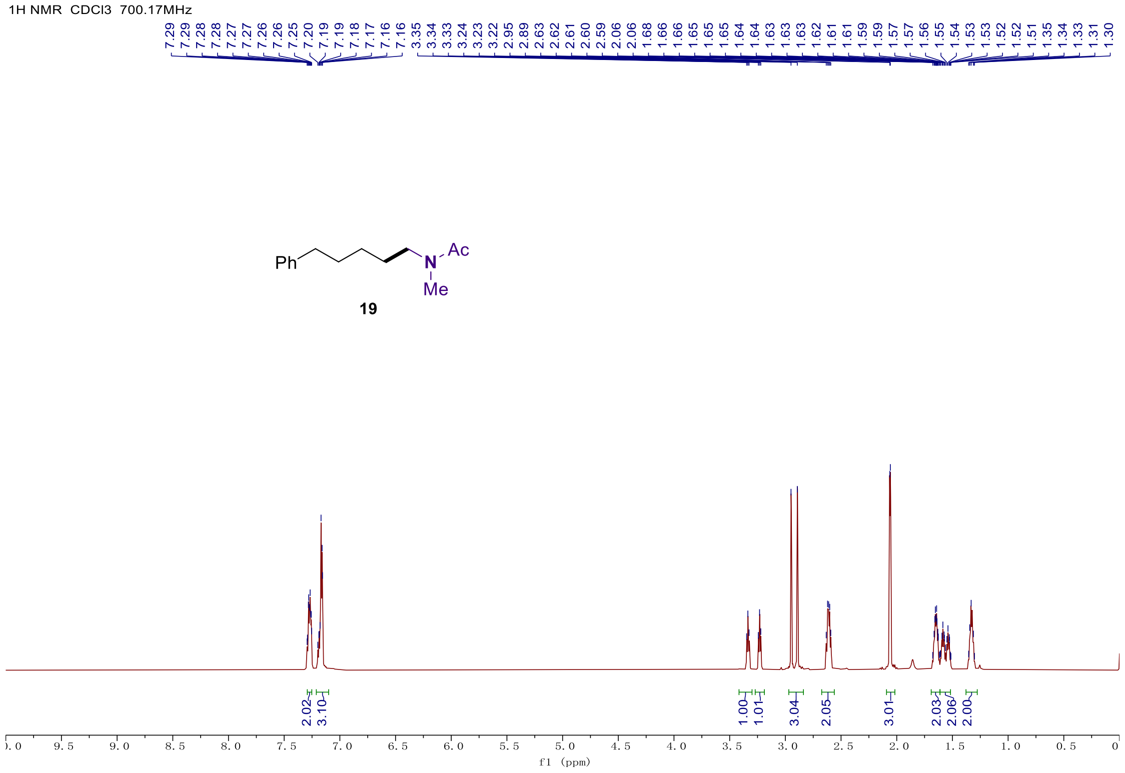

Supplementary Figure 224. ^{1}H NMR of compound **19** (700 MHz, $CDCl_3$)

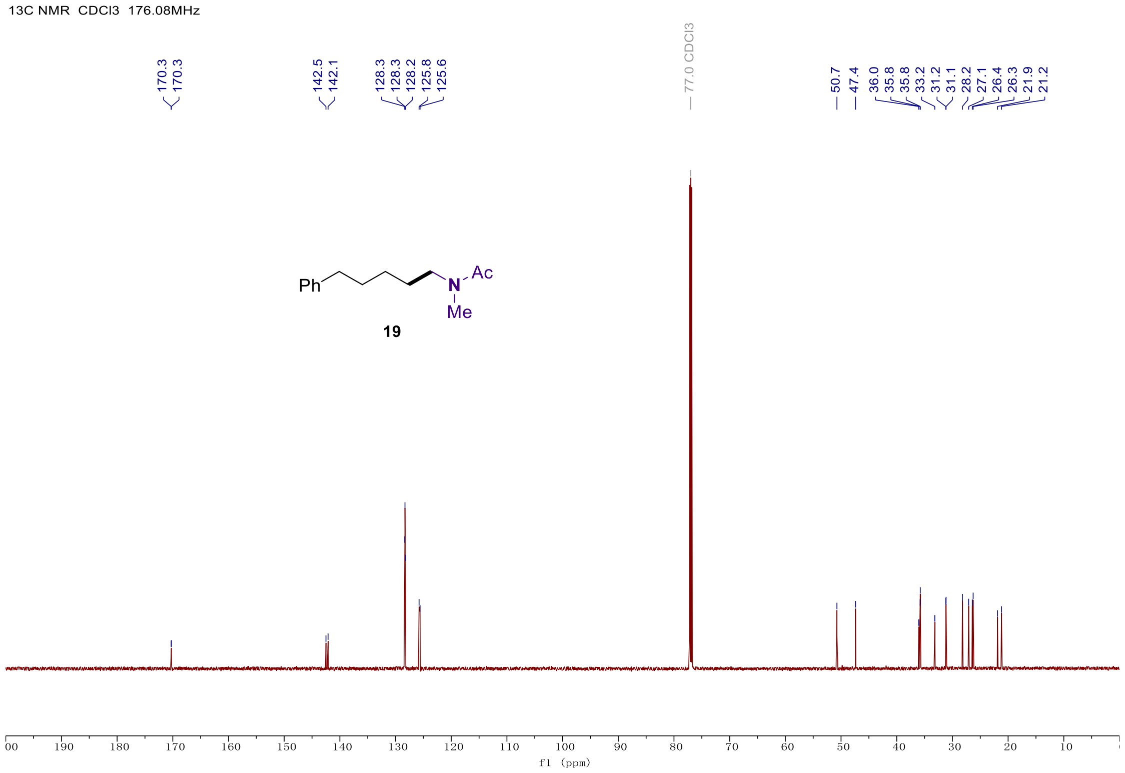

Supplementary Figure 225. ^{13}C NMR of compound 1**9** (176 MHz, $CDCl_3$)

5. Supplementary References

[1] Laursen, J. S., Engel-Andreasen, J., Fristrup, P., Harris,P. & Olsen, C. A. *J. Am. Chem. Soc.* **135**, 2835–2844 (2013).

[2] Sultane, P. R., Mete, T. B. & Bhat, R. G. Chemoselective *N*-deacetylation under mild conditions. *Org. Biomol. Chem.* **12**, 261-264 (2014).
